# Asymmetric Transfer Hydrogenation as a Key Step in the Synthesis of the Phosphonic Acid Analogs of Aminocarboxylic Acids

**DOI:** 10.1002/chem.202302171

**Published:** 2023-09-20

**Authors:** Tamara Dinhof, Thomas Kalina, Toda Stanković, Kristóf Braunsteiner, Philipp Rohrbach, Ertan Turhan, Andreas Gradwohl, Artur Königshofer, Jeannie Horak, Katharina Pallitsch

**Affiliations:** ^1^ Institute of Organic Chemistry Faculty of Chemistry University of Vienna Währingerstraße 38 1090 Vienna Austria; ^2^ Vienna Doctoral School in Chemistry (DoSChem) University of Vienna Währingerstraße 42 1090 Vienna Austria; ^3^ Institute of Inorganic Chemistry Faculty of Chemistry University of Vienna Josef-Holaubek-Platz 2 1090 Vienna Austria; ^4^ Division of Metabolic and Nutritional Medicine Dr. von Hauner Children's Hospital Ludwig Maximilians University Munich Medical Center Lindwurmstraße 4 80337 Munich Germany

**Keywords:** asymmetric transfer hydrogenation, deuteration, aminophosphonates, hydroxyphosphonates

## Abstract

α‐Aminophosphonic acids have a remarkably broad bioactivity spectrum. They can function as highly efficient transition state mimics for a variety of hydrolytic and angiotensin‐converting enzymes, which makes them interesting target structures for synthetic chemists. In particular, the phosphonic acid analogs to α‐aminocarboxylic acids (P^a^AAs) are potent enzyme inhibitors, but many of them are only available by chiral or enzymatic resolution; sometimes only one enantiomer is accessible, and several have never been prepared in enantiopure form at all. Today, a variety of methods to access enantiopure α‐aminophosphonic acids is known but none of the reported approaches can be generally applied for the synthesis of P^a^AAs. Here we show that the phosphonic acid analogs of many (proteinogenic) α‐amino acids become accessible by the catalytic, stereoselective asymmetric transfer hydrogenation (ATH) of α‐oxo‐phosphonates. The highly enantioenriched (enantiomeric excess (*ee*) ≥ 98 %) α‐hydroxyphosphonates obtained are important pharmaceutical building blocks in themselves and could be easily converted to α‐aminophosphonic acids in most studied cases. Even stereoselectively deuterated analogs became easily accessible from the same α‐oxo‐phosphonates using deuterated formic acid (DCO_2_H).

## Graphical abstract

The asymmetric transfer hydrogenation (ATH) of 17 diisopropyl α‐oxophosphonates was accomplished by using a commercially available Noyori‐type catalyst. The highly enantioenriched products (*ee* >98 % in all cases but one) were further converted to the phosphonic acid analogs of 15 aminocarboxylic acids. The established method can also be used for the asymmetric transfer deuteration (ATD) of the starting α‐oxo‐phosphonates.

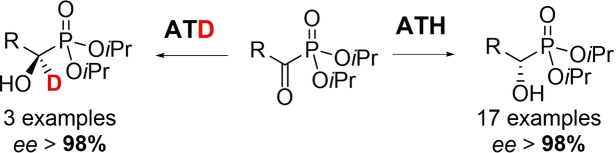

## Introduction

**Figure 1:**
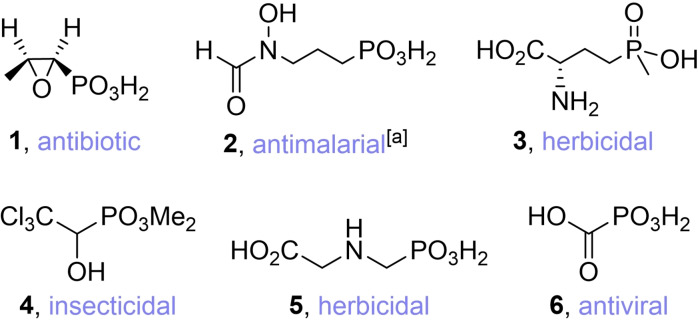
*Upper row*: marketed phosphonates of natural origin and their biological activity, [a] in clinical trials; *lower row*: selected examples of marketed, synthetic phosphonates and their bioactivity.

Biogenic phosphonates (Pn)[^1^, ^2^, ^3^] have a commercialization rate far above the average for all isolated natural products (15 % compared to 0.1 %).^[4]^ Unsurprisingly, the remarkable bioactivity spectrum^[3]^ of biogenic Pns led to the development of many phosphonate‐based drugs for pharmaceutical and agricultural use, some of which are extensively used today (Figure 1).

Among the different types of synthetic Pn‐based drug candidates, α‐aminophosphonates and the related phosphonopeptides soon stood out considerably.[^5^, ^6^, ^7^] Their bioactivity can be attributed to fundamental structural and electronic properties making them excellent bioisosteres to α‐aminocarboxylic acids.[^8^, ^9^] The tetrahedral geometry of the phosphonate moiety in combination with the high intrinsic stability of the P−C bond, makes them ideal inhibitors of many hydrolytic and angiotensin‐converting enzymes by mimicking tightly bound transition states.[^5^, ^10^] The useful properties of α‐aminophosphonates range from antihypertensive, to osteoarthritic effects,^[11]^ they are further promising agents against Leishmaniasis,^[12]^ Alzheimer's disease, and kidney stone formation.^[13]^

Thus, there is an increased scientific interest in the development of stereoselective methods for the synthesis of α‐aminophosphonates. Among the varied approaches, stereoinduction by asymmetric hydrogenation (AH)^[14]^ is equally known as the reduction^[15]^ of α‐oxo‐ and α‐iminophosphonates in the presence of complex metal hydrides as the key step. While hydride reductions are known to furnish products of modest *ee*s,[^16^, ^17^] AH‐based approaches proceed with good to excellent enantioselectivities but only starting from α‐enamidophosphonates,^[18]^ or α‐enolphosphonates.^[19]^ Studies on the AH of α‐oxo‐[^20^, ^21^] or α‐iminophosphonates[^15^, ^22^, ^23^, ^24^] in the presence of varied transition metal‐based catalysts[^17^, ^20^, ^25^] are often limited to α‐aryl‐substituted substrates.

Despite the significant general advantages of asymmetric transfer hydrogenation (ATH)[^26^, ^27^] over AH and its particularly good performance in the hydrogenation of challenging substrates,[^28^, ^29^] the ATH of α‐oxo‐phosphonates was never considered as a key strategy for the synthesis of α‐aminophosphonates. The ATH of α‐oxo‐phosphonates is generally underexplored with only two literature reported examples.[^30^, ^31^]

## Results and Discussion

In 2013, Corbett and Johnson elaborated an elegant dynamic kinetic resolution (DKR) process of α‐oxo‐β‐aryl phosphonates by ATH using one of the most common, commercially available Noyori‐type catalysts: (R*,R*)‐RuCl[(*p*‐cymene)TsDPEN] [(R*,R*)‐**7**].^[32]^ In a side note, they could show that β‐aryl substituents are not required for excellent *ee*s and that even fairly small, aliphatic α‐oxophosphonates can be efficiently hydrogenated by this method. Inspired by their work, we investigated the potential of this transformation for the synthesis of varied α‐aminophosphonic acids by ATH of varied α‐oxo‐phosphonates as central transformation. We planned to integrate an ATH as the key step during the synthesis of highly enantioenriched phosphonic acid analogs to the 19 chiral, proteinogenic α‐aminocarboxylic acids. The latter and their peptidic derivatives are known to be potent enzyme inhibitors.^[33]^ Prominent examples include (*R*)‐phosphaleucine,^[34]^ (*R*)‐ and (*S*)‐phosphaalanine (as a component of the drugs alafosfalin and fotemustine),^[35]^ (*R*)‐phosphatyrosine (as component of the bioactive compound K26),^[36]^ and phosphaphenylalanine, to name but a few.^[37]^

Due to their interesting properties, today stereoselective syntheses for some phospha‐analogs to proteinogenic α‐aminocarboxylic acids are known, but they have never been prepared by the same strategy. Usually, completely different approaches are needed for each target compound, and often both enantiomers have to be synthesized by different sequences. Even today, diastereoselective methods^[38]^ are frequently chosen, or complex, chiral starting materials and chiral auxiliaries are needed.[^39^, ^40^, ^41^, ^42^] Many methods (additionally) rely on chiral or enzymatic[^43^, ^44^, ^45^] resolution techniques.

**Scheme 1:**
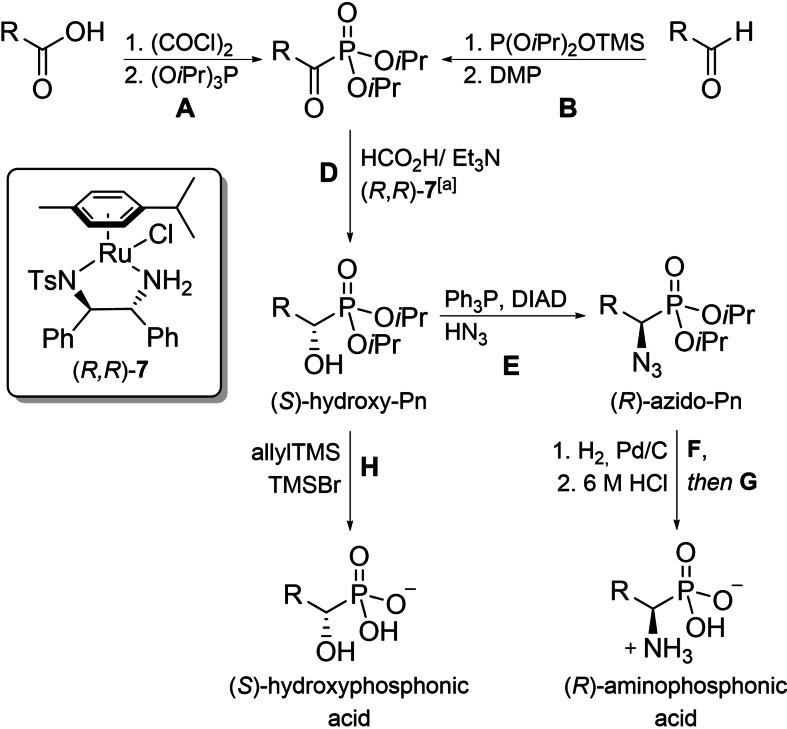
Outline of the general synthetic sequence; general procedures are named with capital letters (A–H), as they appear in the experimental section of the manuscript; [a] activated by general procedure C; TMS=trimethyl silyl, DMP=Dess Martin periodinane, DIAD=diisopropylazodicarboxylate.

We intended to overcome those drawbacks by relying on an unaltered sequence of 4 transformations in combination with suitable protecting groups for the synthesis of as many phosphonic acid analogs to the proteinogenic α‐aminocarboxylic acids as possible (Scheme 1).

The planned steps are: 1) α‐oxo‐phosphonate formation (by general procedure **A** or **B**, see experimental section), 2) ATH in the presence of activated (*R*,*R*)‐**7** (catalyst activation by general procedure **C**, ATH by general procedure **D**), 3) conversion of the resulting (*S*)‐α‐hydroxyphosphonates to the corresponding azides (by general procedure **E**), and 4) hydrogenation and global deprotection (by general procedure **F**, then **G**).

By producing highly enantioenriched α‐hydroxyphosphonates as intermediates, chiral α‐hydroxyphosphonic acids become accessible too (by deprotection following general procedure **H**). They are often bioactive themselves and play crucial roles in the global phosphorus cycle.[^3^, ^46^] Enantiopure samples of these α‐hydroxyphosphonic acids are of particular interest for enzyme mechanistic studies (see below). Furthermore, if 1‐[^2^H]formic acid is used as a deuterium source during the ATH‐process, deuterated analogs of these compounds become available.

### α‐Oxo‐phosphonate formation

α‐Oxophosphonates are known to have a low overall stability and are thus often delicate to handle. For the planned ATH we chose diisopropyl acyl‐phosphonates as substrates. They tend to be relatively stable (compared to dimethyl or diethyl acyl‐phosphonates), are usually obtained in sufficient purity for the intended ATH and can in some cases even be purified by crystallization, distillation or chromatography. Most of them were obtained by reacting the corresponding acyl chlorides with triisopropyl phosphite in an Arbuzov‐reaction at 0 °C (general procedure **A**, Scheme 1). Short reaction times and low temperatures were shown to be of particular importance for acyl chloride formation during the synthesis of oxo‐phosphonate **11** (Table 1), where racemization of the β‐stereogenic center next to the phosphorus was observed otherwise, while slightly higher temperatures proved beneficial for the formation of **24**.

**Table 1:** Tested substrate scope for ATH.

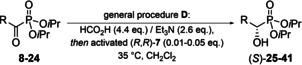				
Residue (R)	α‐oxo‐ Pn	α‐OH‐ Pn	Isolated yield [%]	*ee* [%]
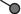	**8**	**25**	99	≥98^[a,b]^
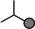	**9**	**26**	95	≥99^[b]^
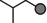	**10**	**27**	96	≥99^[b]^
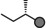	**11**	**28**	96	≥99^[a,b,c]^
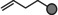	**12**	**29**	90	≥98^[b]^
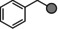	**13** ^[c]^	**30**	78	≥98^[a,b]^
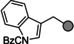	**14** ^[c]^	**31**	65	≥98^[a,b,d]^
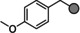	**15**	**32**	76	≥99^[a,b]^
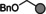	**16**	**33**	84	≥99^[b]^
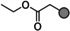	**17**	**34 a**	82	≥99^[b]^
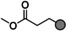	**18**	**35**	82	≥99^[b]^
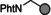	**19**	**36**	81	≥99^[a,b,e]^
, 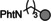	**20**	**37**	74	≥99^[a]^
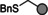	**21**	**38**	73	≥98^[b]^
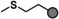	**22**	**39**	94	≥80^[b]^
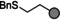	**23**	**40**	98	≥98^[b]^
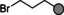	**24**	**41**	75^[d]^	≥ 99^[b,f]^

*ee* values of all shown compounds were determined by ^[a]^ chiral HPLC or ^[b]^ by NMR spectroscopy using chiral solvating agent (*R*)‐**71**; ^[c]^ *de* ≥97 %; ^[d]^ diethyl phosphonate; ^[e]^ the *R*‐enantiomer of **36** was prepared using (*S*,*S*)‐**7**; ^[f]^ in inseparable admixture with diisopropyl (tetrahydrofuran‐2‐yl)phosphonate (4 mol %).

If the respective acyl chloride cannot be obtained without considerable decomposition of the starting acid, the use of racemic α‐hydroxyphosphonates (as obtained by Pudovik or Abramov reactions) is a versatile work‐around. Racemic α‐hydroxyphosphonates can be easily oxidized to the corresponding α‐oxo‐phosphonates in the presence of DMP (Dess–Martin periodinane, general procedure **B**, Scheme 1). The reaction proceeds smoothly between 0 °C and room temperature and is usually finished within 30–60 min. The obtained α‐oxo‐phosphonates can be directly used after extractive removal of the remaining oxidant. Noteworthy, we did not aim for the preparation of a suitable α‐oxo‐phosphonate for the synthesis of phospha‐threonine, as it cannot be accessed in the desired configuration by the outlined method and is available in a simple one‐step procedure from commercially available Fosfomycin.^[47]^

The preparation of suitable α‐oxo‐phosphonates for the synthesis of all other phospha‐analogs to the proteinogenic α‐aminocarboxylic acids was attempted. However, we did not succeed to prepare a suitable precursor for the synthesis of phosphahistidine by the outlined procedures (**A** or **B**).

### General features and limitations of the ATH

The α‐oxo‐phosphonates obtained by either method were directly subjected to ATH using a classic mixture of formic acid and Et_3_N as the hydrogen source (general procedure **D**) and commercially available (*R*,*R*)‐RuCl[(*p*‐cymene)‐TsDPEN] in an activated form (general procedure **C**) as the catalyst. Noteworthy, catalyst activation is not mandatory, but produces significantly higher yields. No intermediate purification step is needed, and by‐products are usually not formed in significant amounts. While (*R*,*R*)‐**7** reliably produced (*S*)‐hydroxyphosphonates, (*S*,*S*)‐**7** was shown to give (*R*)‐hydroxyphosphonates with the same enantiopurity in all studied cases.

As we focused on the synthesis of phosphonic acid analogs to proteinogenic α‐aminocarboxylic acids, only the synthesis of the respective (*S*)‐hydroxyphosphonates [finally leading to (*R*)‐aminophosphonic acids] is shown here, but changing the catalyst configuration produces the opposite enantiomers. Thus, both enantiomers of the same compound can be obtained with equal efficiency and enantiopurity, starting from a shared intermediate.

In most cases, very low catalyst loadings (1 %) were sufficient, except for sulfur‐containing substrates. These required up to 5 mol % catalyst, due to a significant amount of inactivation. The ATH proceeded smoothly with good to excellent yields for all tested substrates and was usually finished within 2–3 h, but could be left unattended for longer periods (24‐72 h) without observable decomposition or loss in *ee*. The *ee*s were excellent (≥98 %, Table 1) in all, but one investigated case (ATH of **22** gave **39** with 80 % *ee*). Reaction temperatures from room temperature to 35 °C made dichloromethane the solvent of choice, which can be easily removed after completion of the reaction. The reaction can be performed under air and is not moisture sensitive. No aqueous work‐up is necessary and the reaction mixtures can be purified by column chromatography directly after concentration *in vacuo*. We found the reaction to be scalable with high reproducibility in the range of 0.1 to 50 mmol as tested for the synthesis of **25** (in the presence of 0.8 mol % catalyst).

Simple alkyl chains, whether branched or linear, are equally tolerated as β‐(hetero)aryl residues, ethers, esters, phthalimides, thioethers, or halogen substituents. All studied substance classes were accessible with excellent enantioselectivity and yield.

**Scheme 2:**
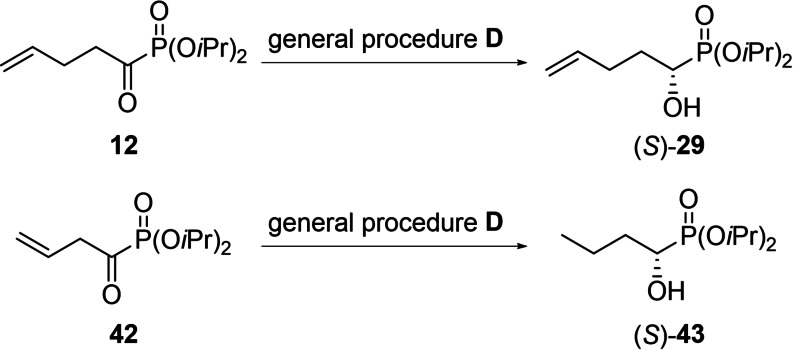
Limitations of the performed ATH for unsaturated α‐oxo‐phosphonates.

While thioethers were generally accepted as substrates, methylthioethers were found to lead to significantly lower *ee*s than the corresponding benzylthioethers (compare reduction of **22** and **23**, Table 1). Alkenes remain intact as long as they are at least in γ‐position to the carbonyl group. If the double bond is closer, the corresponding hydroxyalkanyl‐phosphonate is obtained (Scheme 2).

This limitation is well exemplified by the fact that the ATH of **12** yields the desired diisopropyl 1‐hydroxy‐pent‐4‐enylphosphonate (**29**), while the reduction of diisopropyl‐1‐oxobut‐3‐enyl phosphonate (**42**) yields diisopropyl 1‐hydroxybutylphosphonate (**43**).

Note that β‐hydroxyphosphonates are accessible too, albeit in slightly lower enantiopurity,^[48]^ possibly because of competing directing effects in combination with a decreased stability during transformations after ATH.

While a significant amount of enolization was observed for β‐aryl‐α‐oxo‐phosphonates (such as **13**, **16**, or **19**), β‐alkyl‐α‐oxophosphonates did not show a similar behavior. Thus, the stereogenic center in β‐position to the phosphorus in **11** remained intact under the conditions used for ATH. In case (±)‐**11** was used as substrate for ATH, a 1 : 1 mixture of diastereomers (1*S*,2*R*)‐ (*ee*≥99 %) and (1*S*,2*S*)‐**28** (*ee*≥99 %) resulted.

The obtained (*S*)‐hydroxyphosphonates can be either converted to the corresponding (*R*)‐aminophosphonic acids (general procedure **E** to **G**), or directly deprotected to give (*S*)‐hydroxyphosphonic acids (general procedure **H**).

### Asymmetric transfer deuterations

**Figure 2:**
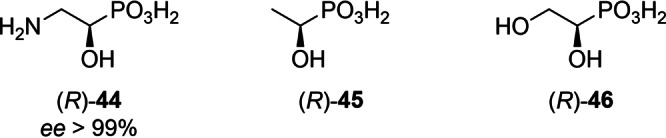
Environmentally important α‐hydroxyphosphonic acids that can be obtained by general method **H**.

Direct deprotection of the obtained α‐hydroxyphosphonates produces highly enantioenriched α‐hydroxyphosphonic acids, many of which play an important biochemical role^[3]^ such as **44**,^[46]^ **45** ^[48]^ and **46** ^[49]^ (Figure 2).

As their metabolism has a direct impact on the global phosphorus cycle, deuterated substrate analogs are often needed to study the involved enzymatic transformations. Thus, we tested the possibility to synthesize enantiopure α‐deutero‐α‐hydroxyphosphonates by the same method as described above. By simply using 1‐[^2^H]formic acid (DCO_2_H) and Et_3_N instead of HCO_2_H/Et_3_N as hydrogen source, the deuterated analogs were accessible from the same α‐oxophosphonates as the respective *protio* compounds. Deuterium incorporation rates were ≥92 % in all three studied case (Table 2).

**Table 2:** Tested substrates and conditions for asymmetric transfer deuteration of α‐oxo‐phosphonates.

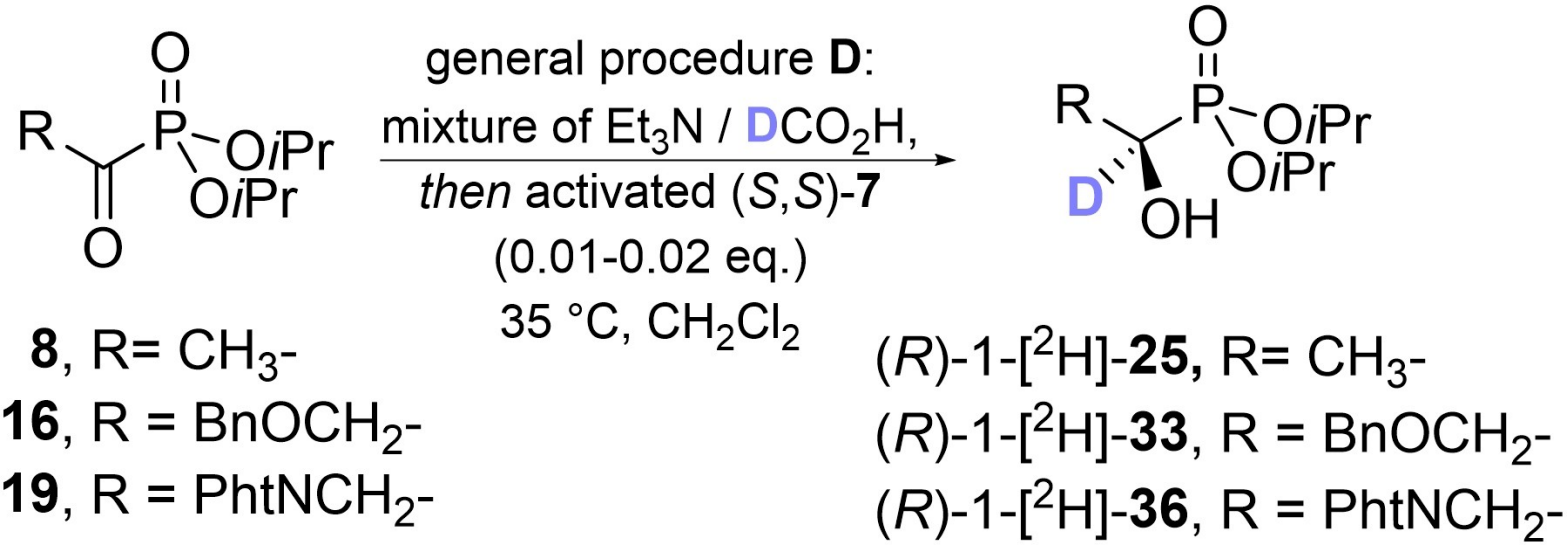				
Residue (R)	Equiv. DCO_2_H	Equiv. Et_3_N	Isolated yield [%]	Degree of deuteration [%]
CH_3_‐	4.4	2.6	99	≥99
CH_3_‐	2.6	2.6	99	≥99
CH_3_‐	1.5	1.5	98	≥99
BnOCH_2_‐	2.6	2.6	79	≥92
PhtNCH_2_‐	2.6	2.6	72	≥99

It is established standard to use 4.4 equivalents of formic acid and 2.6 equivalents of Et_3_N with respect to the substrate during ATHs with Noyori‐type catalysts. As both reagents are cheap and easily available, the high excess of both reagents is usually not questioned. However, significantly lower amounts of reagents are desirable, if 1‐[^2^H]formic acid is used. Thus, the effects of lower amounts of formic acid on the reaction outcome were investigated. It was found that an equimolar mixture of Et_3_N and deuterated formic acid in a slight molar excess (1.5 equiv. relative to the substrate) has the same efficiency as the established 4.4 : 2.6 ratio in terms of *ee*, yield, and degree of deuteration.

This cheap (1‐[^2^H]formic acid: € 3 per mmol in 2023) and convenient transformation allows the synthesis of both enantiomers of isotopically labeled, enantiopure hydroxyphosphonates from the same intermediates as the *protio* compounds. Like this, many key substances for enzyme mechanistic studies become available that were formerly only accessible by chemoenzymatic syntheses.[^50^, ^51^] These include both enantiomers each of 1‐[^2^H_1_]‐2‐amino‐1‐hydroxyethylphosphonic acid {(*R*)‐ and (*S*)‐[^2^H]‐**44**}, 1‐[^2^H_1_]‐1‐hydroxyethylphosphonic acid {(*R*)‐ and (*S*)‐[^2^H]‐**45**}, and 1,2‐dihydroxy‐1‐[^2^H_1_]‐ethylphosphonic acid {(*R*)‐ and (*S*)‐[^2^H]‐**46**}.^[49]^ Similarly, both enantiomers of 1‐[^2^H_1_]‐1‐[^13^C]‐**44** were obtained in a multigram scale starting from ^13^C‐labeled glycine as substrates for enzyme mechanistic studies.

In principle, [^2^H_2_]formic acid (DCO_2_D) can be used too, except for the asymmetric transfer deuteration of α‐oxophosphonates prone to tautomerize to the respective enol‐form such as **16** or **19**. In both cases, a significant amount of deuterium incorporation was observed in the β‐position to the phosphorus (60–70 % respectively) regardless of the reaction temperature (−20 to +35 °C), time (1 to 24 h) and used amount of [^2^H_2_]‐formic acid (1.5 to 4.4 equiv.).

### Conversion of α‐hydroxyphosphonates to α‐aminophosphonic acids

In most cases, the enantioenriched (*S*)‐hydroxyphosphonates obtained by ATH, were subjected to Mitsunobu reactions with HN_3_ under standard conditions (general procedure **E**) yielding (*R*)‐azidophosphonates. However, other types of nucleophiles (such as tetrachlorophthalimide) can be used as well (data not shown). In some cases, purification problems made the use of D*t*BAD (di‐*tert*‐butylazodicarboxylate) or DEAD (diethylazodicarboxylate) instead of DIAD (diisopropylazodicarboxylate) for the Mitsunobu reactions necessary. This often resulted in a lower isolated yield.

However, remaining traces of hydrazoesters do not necessarily need to be removed, as they do not interfere with the subsequent reaction steps and can be easily separated from the final α‐aminophosphonic acids. These intermediately obtained azides can be easily converted to the corresponding aminophosphonates by hydrogenolysis under neutral or acidic conditions (as specified for each compound), which is compatible with a broad variety of functional groups (general procedure **F**). Both the Mitsunobu reaction and the final deprotection steps can be performed on a gram scale^[2]^ and were already used to produce gram quantities of several mentioned target compounds for biochemical studies [such as (*R*)‐**55**, (*R*)‐**59**, (*R*)‐**67**].

**Scheme 3:**
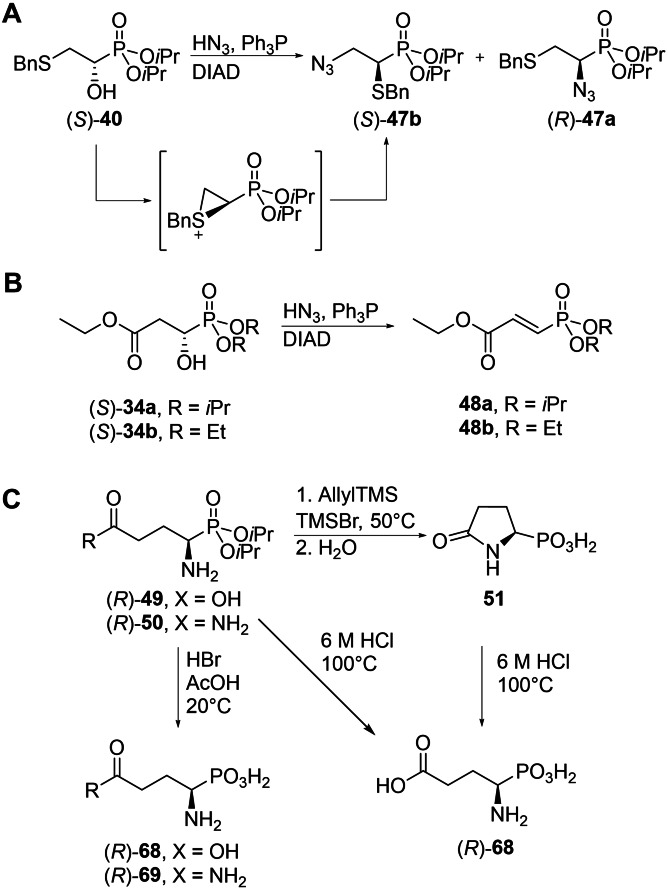
Observed limitations of general procedures **E** and **G**. A: Assumed mechanism for the formation of by‐product (*S*)‐**47 b** during the synthesis of (*R*)‐**47 a**. B: Elimination of the hydroxy group in (*S*)‐**34 a** and **b**. C: Tested conditions for deprotection of (*R*)‐**49** and (*R*)‐**50**, including the obtained products.

The Mitsunobu reaction typically proceeded smoothly, except for a few studied cases. We observed a partial rearrangement during the Mitsunobu reaction of (*S*)‐**40**, to give a mixture of 2‐azido‐1‐benzylthioethyl phosphonate **47 b** and the desired product **47 a** (Scheme 3). The obtained ratio of **47 a** to **47 b** was highly dependent on the used solvent mixture and can be shifted to favour the formation of either product (for details see the Supporting Information). While hydroxyphosphonate **35** was smoothly converted to azide **49**, subjecting **34 a** to the same conditions exclusively gave the elimination product 3‐(diisopropoxyphosphoryl)acrylic acid (**48 a**). Using the spatially less demanding diethyl phosphonate exclusively gave the elimination product **48 b** too. Thus, phosphaasparagine could not be obtained from **34 a** as intended.^[52]^ Alternatively, the replacement of the hydroxy group in diisopropyl (*R*)‐1‐amino‐2‐hydroxyethylphosphonate (**52**, an intermediate obtained during the synthesis of phosphaserine) in the presence of potassium cyanide/Ph_3_P/DIAD followed by acidic hydrolysis was attempted. Unfortunately, this did not produce the desired product either.^[53]^

In all other cases, the desired azides were obtained in satisfying yields. Hydrogenation of the obtained azidophosphonates was typically finished within 2–3 h at room temperature in a Parr apparatus at 3.5 atm H_2_‐pressure using Pd on activated charcoal as catalyst (general procedure **F**). In some cases, the addition of small amounts (1‐2 drops) of conc. HCl proved beneficial.

Final deprotection of the intermediate α‐aminophosphonates was usually performed by acidic hydrolysis in the presence of 6 M HCl (general procedure **G**), followed by ion exchange chromatographic purification and crystallisation of the product if possible. In some cases, additional transformations were necessary before final deprotection as outlined in the Supporting Information.

It should be noted that in most cases, both general procedure **H** and **G** can be used for the final deprotection of the intermediately obtained α‐aminophosphonates. Deprotection of (*R*)‐**49** and (*R*)‐**50** is however not possible using general procedure **H** (TMSBr/allylTMS, Scheme 3C) which resulted in the exclusive formation of the cyclic product (*R*)‐**51**.^[54]^ This intermediate can also serve as an alternative substrate for the synthesis of enantiopure phosphaproline (**55**).^[55]^ In agreement with the literature, using general procedure **G** (6 M HCl, 100°C) can be either used to hydrolyze (*R*)‐**51** or to directly form (*R*)‐**68** is. (*R*)‐**69** can only be obtained at low temperatures, therefore, stirring in HBr/AcOH (33% HBr, 5.7 M) at room temperature was the method of choice.^[56]^

**Figure 3:**
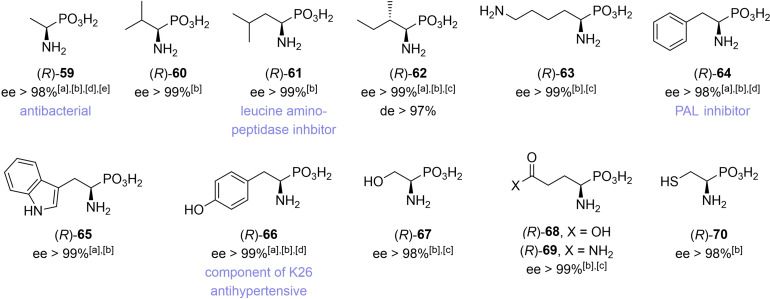
Structures of α‐aminophosphonic acids **56**–**67**; *ee* was determined either at the α‐hydroxyphosphonate stage by [a] chiral HPLC or by [b] NMR spectroscopy; at the [c] α‐azidophosphonate stage by NMR spectroscopy, or at the [d] α‐aminophosphonic acid stage by chiral HPLC. [e] *ee* determination after derivatization as indicated in the Experimental Section.

**Scheme 4:**
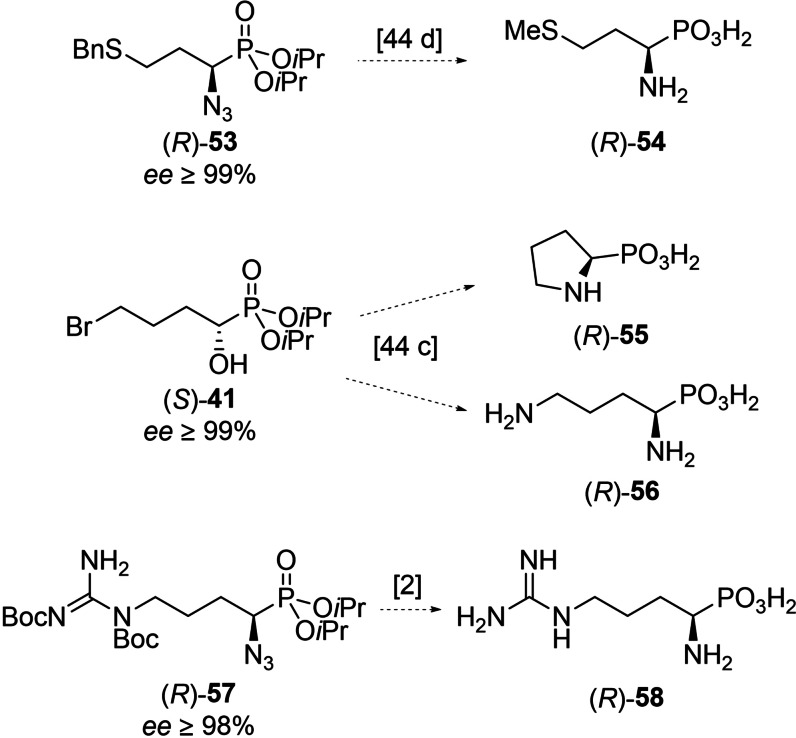
Obtained late‐stage precursors for the synthesis of aminophosphonic acids **54**, **55**, **56** and **58**. Numbers in square brackets indicate known literature references for the anticipated conversions.

Like this we were able to synthesize highly enantioenriched samples of the phosphonic acid analogs to 12 α‐aminocarboxylic acids (**59**‐**70**, Figure 3) and late‐stage intermediates for the synthesis of 3 other proteinogenic α‐aminophosphonic acids {phosphamethionine [(*R*)‐**54**], phosphaproline [(*R*)‐**56**], phosphaarginine [(*R*)‐**58**]}. Additionally, many of the intermediately obtained α‐hydroxyphosphonates can be converted to other α‐aminophosphonates too. Hydroxyphosphonate (*S*)‐**41** can, for example, be easily converted to phosphaornithine [(*R*)‐**56**] (Scheme 4).

### ee determination

The enantiomeric excess of all synthesized α‐hydroxy‐, and some synthesized α‐azidophosphonates was determined by a combination of chromatographic and spectroscopic methods (Figure 3). While chiral stationary phase HPLC provides very exact results, optimization of the separation parameters and conditions can be very time‐consuming and the whole process uses high solvent amounts. In many cases, only UV‐active compounds can be easily detected, creating the need to further derivatize aliphatic compounds to facilitate detection, as in the case of hydroxyphosphonate **25**. Thus, we resorted to a known, easy to handle, quick and reliable spectroscopic method for *ee* determination on a daily basis. Addition of an excess of (+)‐(*R*)‐*tert*‐butylphenylphosphinothioic acid [(+)‐(*R*)‐**71**] as chiral solvating agent to the respective NMR samples can directly provide the *ee* of the synthesized compounds.^[57]^ This easy method allows to distinguish between both enantiomers of all obtained intermediate α‐hydroxyphosphonates and many described α‐azidophosphonates as indicated (compare Figure 3). The *ee* of α‐functionalized phosphonates can be determined by direct comparison of signal integrals of the formed complexes of (*R*)‐**71** with each enantiomer of the studied compound by ^31^P and/or ^1^H NMR spectroscopy. In cases with significant signal overlap, the use of *d*_8_‐toluene often led to better results than using CDCl_3_ as solvent. The *ee* of several selected compounds was double‐checked by chiral stationary phase HPLC (at the α‐hydroxyphosphonate stage) to verify the reliability of the used spectroscopic method. Both provided the same results in all studied cases (maximum deviation±0.3 % *ee*). Ultimately, the *ee* of several deprotected α‐aminophosphonic acids was checked by chiral stationary phase HPLC to proof the configurational stability of the stereogenic centers during deprotection.

## Conclusions

In summary, 17 highly enantioenriched α‐hydroxyphosphonates, as well as several isotopically labeled analogs have been synthesized by a general, and reliable asymmetric transfer hydrogenation (ATH) process. Easily accessible α‐oxo‐phosphonates were reduced in the presence of a commercially available Noyori‐type catalyst; this made both enantiomers of these compounds accessible by simply changing the catalyst configuration. The obtained α‐hydroxyphosphonates were subsequently converted to environmentally and pharmaceutically important α‐hydroxyphosphonic acids, or to highly enantioenriched (*ee* ≥98 %) phosphonic acid analogs to proteinogenic α‐aminocarboxylic acids. Some of these compounds were produced as pure enantiomers for the first time, such as (*R*)‐phosphatryptophane, (*R*)‐phosphacysteine, (*R*)‐phosphaglutamine or (*R*)‐1‐[^2^H]‐1,2‐dihydroxyethylphosphonic acid. The synthetic sequence consists of four robust key steps in combination with a suitable protecting‐group strategy. It does not rely on enzymatic resolution and is the first generally applicable method for the synthesis of 15 out of 19 chiral phospha‐analogs to the proteinogenic α‐amino acids. Additionally, deuterated analogs to several of the described compounds were synthesized with high rates of deuteration and with excellent *ee*s by using the cheap and easily available deuterium source 1‐[^2^H]formic acid.

## Experimental Section

**General experimental details**: ^1^H, ^13^C and ^31^P NMR spectra were recorded on either a Bruker AV III HD 700 (^1^H: 700.40 MHz, ^13^C: 176.12 MHz), AV III 600 (^1^H: 600.25 MHz, ^13^C: 150.93 MHz, ^31^P: 242.99 MHz), AV NEO 500 (^1^H: 500.32 MHz, ^13^C: 125.81 MHz), AV III 400 (^1^H: 400.27 MHz, ^13^C: 100.65 MHz, ^31^P: 162.03 MHz) or DRX 400 (^1^H: 400.13 MHz, ^13^C: 100.61 MHz, ^31^P: 161.98 MHz) spectrometer. Chemical shifts (*δ*) are given in parts per million (ppm) and were referenced to (residual) solvent signals as follows: ^1^H NMR spectra: CDCl_3_: *δ*_H_ (CHCl_3_) 7.26, *d_6_‐*DMSO: *δ*_H_ [(CD_2_H)SO(CD_3_)] 2.50, CD_3_OD: *δ*_H_ (CD_3_OH) 3.31, *d_8_*‐toluene: *δ*_H_ (CHD_2_/C_6_D_5_) 2.08, and D_2_O: *δ*_H_ (HDO) 4.79; ^13^C NMR spectra:CDCl_3_ (*δ*_C_ 77.16), *d_6_*‐DMSO (*δ*_C_ 39.52), and CD_3_OD (*δ*_C_ 49.00). H_3_PO_4_ (85 %) (*δ*_P_ 0.00 ppm) served as external reference for ^31^P NMR spectra. Coupling constants (*J*) are reported in Hz. ^13^C spectra were recorded *j*‐modulated. The chemical shift of the two parts of AB‐systems are given separately as unweighted mean value of the single signals. “A” is used to denote the down‐field part and “B” to denote the high‐field part of the AB‐system.

High‐resolution mass spectrometry (HRMS) was conducted on a Bruker maXis ultra‐high‐resolution time‐of‐flight (UHR‐TOF) instrument with electrospray ionization (ESI) in the positive‐ion mode. Optical rotations were measured on a Schmidt‐Haensch Digital Polarimeter Unipol L 2000 and are given in 10^−1^ deg cm^2^ g^−1^.

Chromatographic separations (medium pressure liquid chromatography, MPLC) were either carried out on a Biotage Isolera Prime (Biotage, Uppsala, Sweden) flash purification system or manually as indicated. The stationary phase was Macherey–Nagel silica gel 60 (0.04‐0.063 mm) in all cases, cartridges for MPLC were self‐packed. Thin layer chromatography (TLC) was carried out on precoated Merck silica gel 60 F_254_ glass plates. Compounds were visualized with UV light (254 nm) and/or by dipping the plate in one of the following solutions, followed by heating with a heat gun: Cerium ammonium molybdate solution (CAM, 46 g (NH_4_)_6_Mo_7_O_24_⋅4 H_2_O, 2 g Ce(SO_4_)_2_⋅4 H_2_O) in 1 L 10 % (*w*/*w*) aq. H_2_SO_4_), ninhydrin solution (0.5 % (*w*/*w*) ninhydrin in EtOH abs.) or KMnO_4_ solution (9 g KMnO_4_, 60 g K_2_CO_3_ in 900 mL H_2_O and 15 mL 5 % (*w*/*w*) aq. NaOH).

All used chemicals and solvents were purchased from commercial sources and used without further purification. Diethylazodicarboxylate (DEAD) was used as a commercially available solution containing 40 wt.% DEAD (approx.. 2.2 M) in dry toluene; diisopropylazodicarboxylate (DIAD) was used neat, 98 % purity; di‐*tert*‐butylazodicarboxylate (D*t*BAD) was dissolved in the reaction solvent (final concentration 1.0 M) prior to addition.

(+)‐(*R*)‐*tert*‐Butylphenylphosphinothioic acid [(+)‐(*R*)‐**71**] – used as chiral solvating agent for *ee* determination of the synthesized compounds – was obtained according to a literature known procedure.^[57]^

1‐[^2^H]formic acid was obtained as follows: Deuterated sodium formate (3 g, 44.11 mmol, 1 equiv.) was melted together with phosphoric acid (99 %, 25 g, 257.73 mmol, 5.8 equiv.) at 45 °C in a distillation apparatus. Afterwards the reaction mixture was heated to 70 °C in vacuo (0.3 mbar) to evaporate 1‐[^2^H]formic acid, which could be collected at −196 °C. Heating was continued for 2 h until no further distillation of deuterated formic acid was observed. The pure deuterated formic acid (1.4 g, 30.43 mmol, 69 %) was obtained as colorless liquid; ^1^H NMR (600.25 MHz, D_2_O): *δ*=8.19 ppm (s, DC(O)OH); ^13^C NMR (150.93 MHz, D_2_O): *δ*=165.46 ppm (t, ^1^ *J*_CD_=33.5 Hz, DC(O)OH).

Hydrazoic acid (HN_3_, *always to be handled in a fume hood!* ) was prepared from sodium azide (13 g, 200 mmol) dissolved in water (13 mL) in a three‐necked flask (equipped with a dropping funnel and a thermometer) at 40 °C. Toluene (80 mL) was added to the resulting sludge and the turbid mixture was stirred in an ice bath. Then, concentrated H_2_SO_4_ (5.6 mL, 100 mmol) was added slowly to keep the reaction temperature below 10 °C. Finally, the reaction mixture was stirred at 0 °C for 30 min and the organic layer was decanted. The obtained hydrazoic acid solution was dried (Na_2_SO_4_) and stored at 4 °C. The obtained solution typically contains 1.0 to 1.8 mol L^−1^ HN_3_ and the exact concentration was determined by titration with an aqueous NaOH solution in the presence of phenolphthalein. For this purpose, 1 mL of the prepared HN_3_ solution was diluted with water (25 mL).

Aminophosphonic acids [(*R*)‐**59**–(*R*)‐**70**] were prepared from α‐oxophosphonates **8**–**24** following the general procedures **A**–**H** described below. α‐Oxo‐phosphonate **19** ^[58]^ was obtained using a literature known procedure. Specific experimental details and analytical data for each compound can be found in the Supporting Information.

Compounds (*R*)‐1‐[^2^H]‐**44**, (*R*)‐1‐[^13^C]‐**44**, (*R*)‐1‐[^2^H]‐1‐[^13^C]‐**44**, (*R*)‐**52**, (*R*)‐**53**, (*R*)‐**70**, (*S*)‐**76**, (*R*)‐**79**, (*R*)‐**80**, (*R*)‐**86**, (*S*)‐**87**, (*R*)‐**88** and (*R*)‐**89** were not prepared according to the described general procedures. Experimental procedures for their synthesis can be found in the Supporting Information.

The numbering of compounds in the Supporting Information is in agreement with the numbering in the main manuscript.

### General procedures

**General procedure A – ketophosphonates from acyl chlorides**: The required carboxylic acid (1 equiv.) is dissolved in dry CH_2_Cl_2_ (1.5 mL mmol^−1^, final concentration: 0.66 M) under Ar and oxalyl chloride (1.1 equiv.) is added dropwise at 0 °C. The cooling bath is removed and stirring is continued at 25–40 °C for 2–15 h as specified for each compound. All volatiles are removed in vacuo, the crude acyl chloride is redissolved in dry CH_2_Cl_2_ (1.2–1.5 mL mmol^−1^, final concentration: 0.66‐0.83 M) and (*i*PrO)_3_P (1.05 equiv.) is added dropwise at 0 °C. The resulting solution is stirred at 0 °C for 1.5‐3 h before evaporation of the solvent at room temperature (!); product formation can be easily monitored by ^31^P NMR. This provides the crude keto‐phosphonates in sufficient purity for the next step, which can be stored at −20 °C for several months, if necessary.

This general procedure was used for the synthesis of compounds **8**, **9**, **10**, **11**, **12**, **13**, **14**, **15**, **17**, **18**, **20**, **21**, **22** and **23**.

**General procedure B – ketophosphonates from aldehydes**: Diisopropyltrimethylsilyl phosphite (3 equiv.)^[59]^ is added to the needed aldehyde (1 equiv.) under argon in dry toluene (3 mL mmol^−1^, final concentration: 0.33 M) and afterwards the resulting mixture is heated to 80 °C. After 16 h, the solvent is removed in vacuo, the residue is dissolved in a mixture of HCl (2 M, 2 mL mmol^−1^) and THF (tetrahydrofuran, 2 mL mmol^−1^) and stirring is continued for 5 h at room temperature. The phases are separated, and the aqueous layer is then extracted with ethyl acetate (4×3 mL mmol^−1^). The combined organic portions are dried (MgSO_4_) and the residue was purified as described for each substance to yield the racemic α‐hydroxyphosphonates of interest. These substances are dissolved in dry CH_2_Cl_2_ (6 mL mmol^−1^) and cooled to 0 °C. DMP (Dess–Martin periodinane, 1.5 equiv.) is added and stirring is continued for 30 min. Then, the cooling bath is removed, and the reaction mixture is allowed to stir at room temperature for 2–4 h (product formation can be easily monitored by ^31^P NMR) after which the solvent is removed in vacuo without external heating and the remaining residue is taken up in Et_2_O (diethyl ether, 4 mL mmol^−1^). Excess DMP can be easily removed by extraction using a 1 : 1 mixture of sat. aqueous NaHCO_3_ and Na_2_S_2_O_3_ solutions (3×4 mL mmol^−1^). The organic layer is dried (Na_2_SO_4_) and the solvent removed in vacuo at room temperature (!) to yield the desired ketophosphonates in sufficient purity for the next step; they can be stored at −20 °C for several months if necessary.

This general procedure was used for the synthesis of compounds **16** and **24**.

**General procedure C – catalyst activation**: (*R,R*)‐ or (*S,S*)‐RuCl[(*p*‐cymene TsDPEN] (1 equiv.) is dissolved in CH_2_Cl_2_ (1 mL/100 mg, final concentration of 0.16 M; for ≤100 mg of **7** we recommend to still use a minimum of 1 mL CH_2_Cl_2_ in order to facilitate the handling) and an aqueous solution of potassium hydroxide (preferable concentration range: 0.16‐0.20 M, 1 equiv.) is added. The resulting biphasic mixture is vigorously stirred for 1–2 min whereupon the organic layer turns from bright orange to deep purple. The phases are separated, the aqueous layer is extracted with CH_2_Cl_2_ (2×1 mL; small scale extractions of this type can conveniently be done in single‐use 2 mL syringes) and the combined organic layers are dried (CaH_2_, stirring is continued until no further gas evolution is observed. The catalyst solution is filtered over a plug of cotton wool and the residual drying agent washed again with CH_2_Cl_2_ (1‐2 mL). The combined and filtered organic fractions can either be directly used as described in **general procedure D**, or the solvent can be removed in vacuo. The activated catalyst (*R,R*)‐ or (*S*,*S*)‐Ru[(*p*‐cymene)‐TsDPEN] [(*R*,*R*)‐ or (*S*,*S*)‐**7**] can be stored at 4 °C under argon for approximately 10 days.

**General procedure D** – **catalytic transfer hydrogenation**: Formic acid (4.4 equiv., respective to the α‐oxophosphonate) is added dropwise to Et_3_N (2.6 equiv., respective to the α‐oxophosphonate) under Ar at 0 °C and the resulting mixture is stirred for 5 min. The desired crude α‐oxo‐phosphonate (1 equiv.) is dissolved in dry CH_2_Cl_2_ (2 mL mmol^−1^, final concentration 0.5 M) under Ar, and the formic acid/Et_3_N mixture is added, followed by a solution of either (*R,R*)‐ or (*S*,*S*)‐Ru[(*p*‐cymene TsDPEN] [(*R*,*R*)‐**7** or (*S*,*S*)‐**7**, 0.01‐0.05 equiv. as specified for the respective compound] obtained by **general procedure C**. The reaction mixture is stirred at 35 °C for 1–20 h before evaporation of the solvent. *Note*: (*R,R*)‐**7** is used to obtain (*S*)‐hydroxyphosphonates, while (*S*,*S*)‐**7** produces (*R*)‐hydroxyphosphonates. Purification of the products can be performed by flash chromatography as described for each substance. Remaining traces of HP(O)(O*i*Pr)_2_ from ketophosphonate formation can be easily removed at this stage by drying the sample in vacuo (0.3 mbar) at 50 °C for several hours.

This general procedure was used for the synthesis of compounds (*S*)‐ and (*R*)‐**25**, (*S*)‐**26**, (*S*)‐**27**, (1*S*,2*S*)‐**28**, (*S*)‐**29**, (*S*)‐**30**, (*S*)‐**31**, (*S*)‐**32**, (*S*)‐**33**, (*S*)‐**34**, (*S*)‐**35**, (*R*)‐**36**, (*S*)‐**37**, (*S*)‐**38**, (*S*)‐**39**, (*S*)‐**40**, (*S*)‐**41** as well as for the synthesis of (*R*)‐1‐[^2^H]‐**25**, (*R*)‐1‐[^2^H]‐**33**, (*R*)‐1‐[^2^H_1_]‐**36**, (*R*)‐1‐[^13^C]‐**36** and (*R*)‐1‐[^13^C]‐1‐[^2^H]‐**36**.

**General procedure E – Mitsunobu reaction with HN_3_**: The enantiopure hydroxyphosphonate (1 equiv.) and Ph_3_P (1.5 equiv.) are dissolved in the specified solvent mixture (3–5 mL mmol^−1^ hydroxyphosphonates, final concentration: 0.20–0.33 M) under Ar at 0 °C. In case ≤1 mmol of hydroxyphosphonates was used, improved yields were observed after including an additional drying step by coevaporation of residual water from the Ph_3_P and hydroxyphosphonate mixture with dry toluene.

Hydrazoic acid (1.8 equiv. of a solution in dry toluene), followed by the specified dialkyl azodicarboxylate (1.5 equiv, see general experimental details) is added and the reaction mixture is stirred at the specified temperature between 0 and 40 °C until completion of the reaction (monitored by TLC and ^31^P NMR). MeOH (1‐3 mL mmol^−1^ starting material) is added and stirring is continued for another 10 min before evaporation of the solvent in vacuo at room temperature (!). Purification of the products can be performed by flash chromatography as described for each substance.

This general procedure was used for the synthesis of compounds (*R*)‐**47 a** and **b**, (*R*)‐**53**, (*R*)‐**72**, (*R*)‐**73**, (*R*)‐**74**, (1*R*,2*S*)‐**75**, (*R*)‐**77**, (*R*)‐**78**, (*R*)‐**81**, (*R*)‐**82**, (*R*)‐**83**, (*R*)‐**84**, (*R*)‐**85** and (*R*)‐**89**.

**General procedure F – Reduction of azides to amines**: The azide (1 equiv.) obtained by **general procedure E** is dissolved in EtOH (5‐10 mL mmol^−1^, final concentration 0.02‐0.01 M) and conc. HCl (1 drop) is added (unless stated differently), followed by Pd on activated charcoal (10 % Pd, 20 mg mmol^−1^). Hydrogenation is performed at 3.5 atm H_2_ pressure in a Parr apparatus under constant shaking for 3 h. The catalyst is removed by filtration over Celite® (moistened with EtOH) and the solvent is removed in vacuo to give the crude amine of interest. The thus obtained amines are very polar and are directly used for the next reaction steps without further purification.

**General procedure G – deprotection of α‐aminophosphonates**: Protecting groups (diethyl of diisopropyl) can be removed by refluxing the crude amines (1 equiv.), obtained from **general procedure F**, in aqueous HCl (6 M) for 5–15 h as specified for each compound. Then, the solvent is removed in vacuo, the residue taken up in water (2 mL mmol^−1^) and again concentrated to dryness. Ion exchange chromatography over Dowex W50×8/H^+^ form (2 mL moist resin per mmol substance, water or formic acid (5 % in H_2_O) as eluent is used for purification. Ninhydrin positive fractions are pooled and concentrated to give the desired α‐aminophosphonic acids. These can be either crystallized or lyophilized, as specified.

A sequence of general procedures **F** and **G** was used for the synthesis of compounds (*R*)‐**59**, (*R*)‐**60**, (*R*)‐**61**, (1*R*,2*S*)‐**62**, (*R*)‐**63**, (*R*)‐**64**, (*R*)‐**65**, (*R*)‐**66**, (*R*)‐**67**, (*R*)‐**68** and (*R*)‐**69**.

**General procedure H – deprotection of α‐hydroxyphosphonates**: The desired α‐hydroxy‐ or aminophosphonic acid (obtained by **general procedure D** and **F**, respectively) is dissolved in dry DCE (1,2‐dichloroethane, 2 mL mmol^−1^, final concentration: 0.5 M) under argon atmosphere. Subsequently, allyltrimethylsilane (4 equiv.), followed by TMSBr (6 equiv.) is added and the mixture is stirred at 60 °C for 14 h. All volatiles are removed under reduced pressure, the residue is redissolved in DCE (2 mL mmol^−1^) and again concentrated to dryness. The product is taken up in H_2_O (2 mL mmol^−1^), and the pH value is adjusted to 6.5–7.5 (pH electrode for determination) with aqueous NaOH (0.5–1.0 M). The product is obtained as its sodium‐salt after lyophilization.

This general procedure was used to synthesize compound (*R*)‐**45**.

## Conflict of interests

The authors declare no conflict of interest.

1

## Supporting information

As a service to our authors and readers, this journal provides supporting information supplied by the authors. Such materials are peer reviewed and may be re‐organized for online delivery, but are not copy‐edited or typeset. Technical support issues arising from supporting information (other than missing files) should be addressed to the authors.

Supporting Information

Supporting Information

## Data Availability

Pictures to all recorded NMR spectra are available in the Additional Materials to the manuscript. Original data files are available from the authors upon request.
