# Supplementary material for: Asymmetric Transfer Hydrogenation as a Key Step in the Synthesis of the Phosphonic Acid Analogs of Aminocarboxylic Acids
Source: Chemistry. 2023 Sep 20;29(72):e202302171. doi: 10.1002/chem.202302171 (PMC10947287; doi:10.1002/chem.202302171)

Additional Material – Pictures of Recorded NMR Spectra

Asymmetric Transfer Hydrogenation as a Key Step in the Synthesis of the Phosphonic Acid Analogues to Aminocarboxylic Acids

Tamara Dinhof, Thomas Kalina, Toda Stanković, Kristóf Braunsteiner, Philipp Rohrbach, Ertan Turhan, Andreas Gradwohl, Artur Königshofer, Jeannie Horak and Katharina Pallitsch

Pictures of the recorded ^{1}H, ^{13}C and ^{31}P NMR spectra of all synthetized compounds are available as additional material. All assignments, solvents and device parameters are given in the **Experimental Section** of the publication, as well as on top of each picture. The first spectrum shown in each series is the full ^{1}H NMR spectrum. Expansions are depicted where they were regarded as necessary. Then the ^{13}C NMR spectrum and the ^{31}P NMR spectrum are depicted in the same manner.The x-axes is in ppm and the peak labels are in Hertz (Hz) for all shown spectra. Structures are always given on top of the full ^{1}H NMR spectrum. Integrals are denoted below the x-axes where they were regarded as necessary and the integration range is marked. The numbering and order of compounds is in accordance with the numbering and order of substances in the main text and Supporting Information.

^{1}H NMR of 1-[$^{2}H_1$]-formic acid (600.25 MHz, $CDCl_3$):

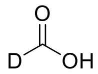

[$^{2}H_1$]-**formic acid**

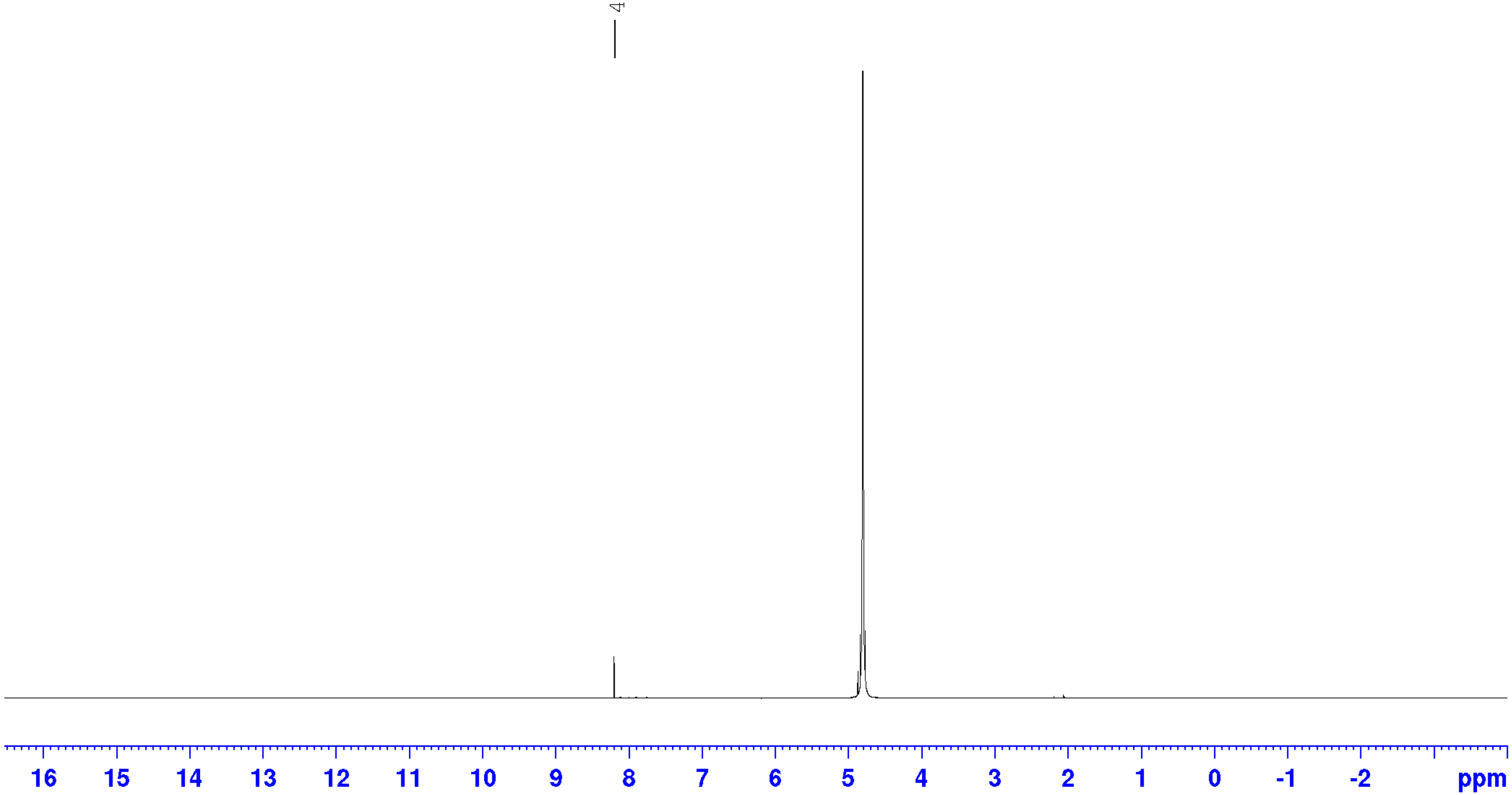

^{13}C NMR of 1-[$^{2}H_1$]-formic acid (150.93 MHz, $CDCl_3$):

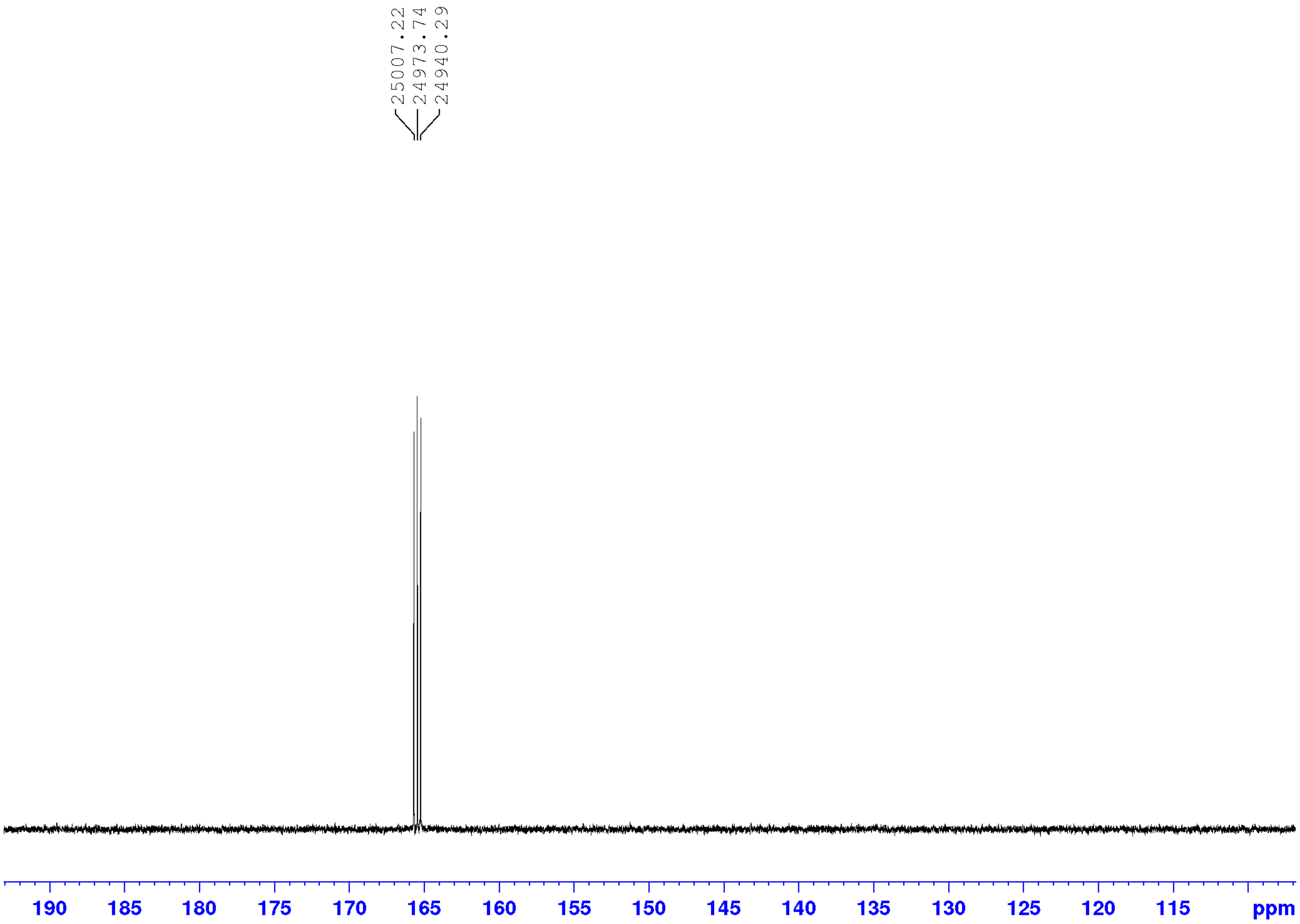

^{1}H NMR of diisopropyl 1-oxo-ethylphosphonate (400.13 MHz, $CDCl_3$) (8):

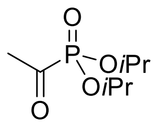

8

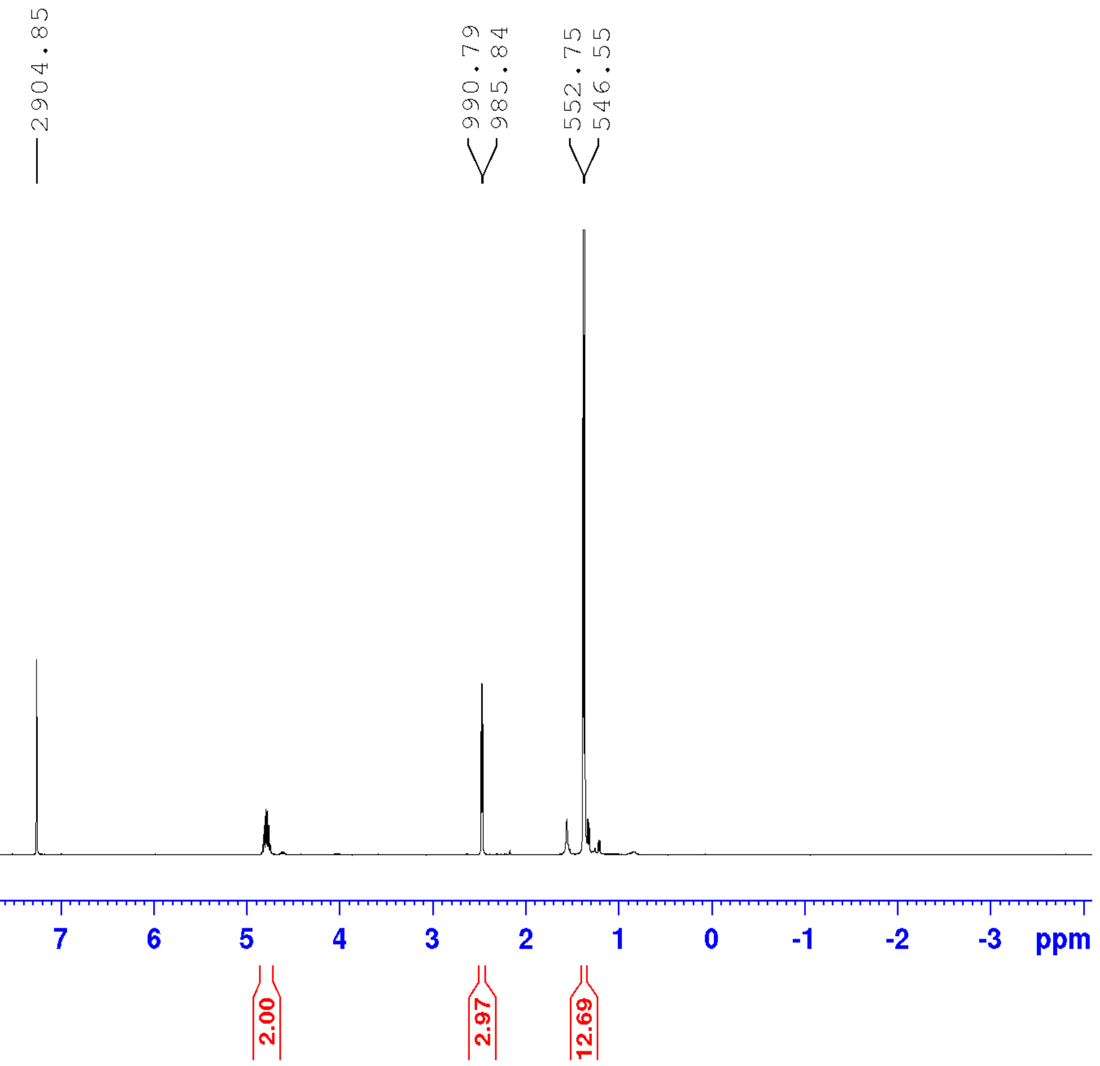

^{31}P NMR of diisopropyl 1-oxo-ethylphosphonate (400.13 MHz, $CDCl_3$) (8):

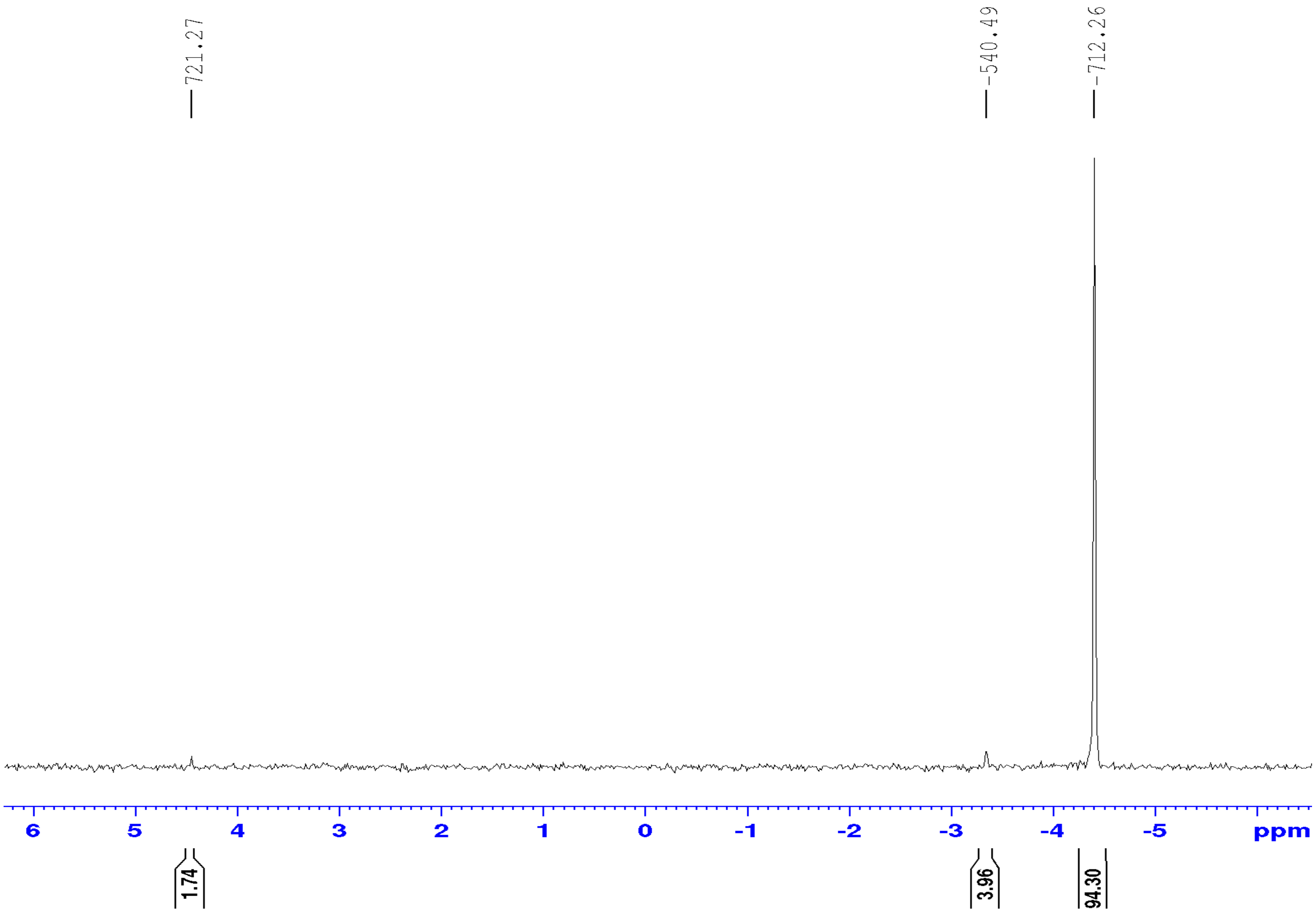

^{1}H NMR of (*S*)-diisopropyl 1-hydroxy-ethylphosphonate (400.27 MHz, $CDCl_3$) [(*S*)-25]:

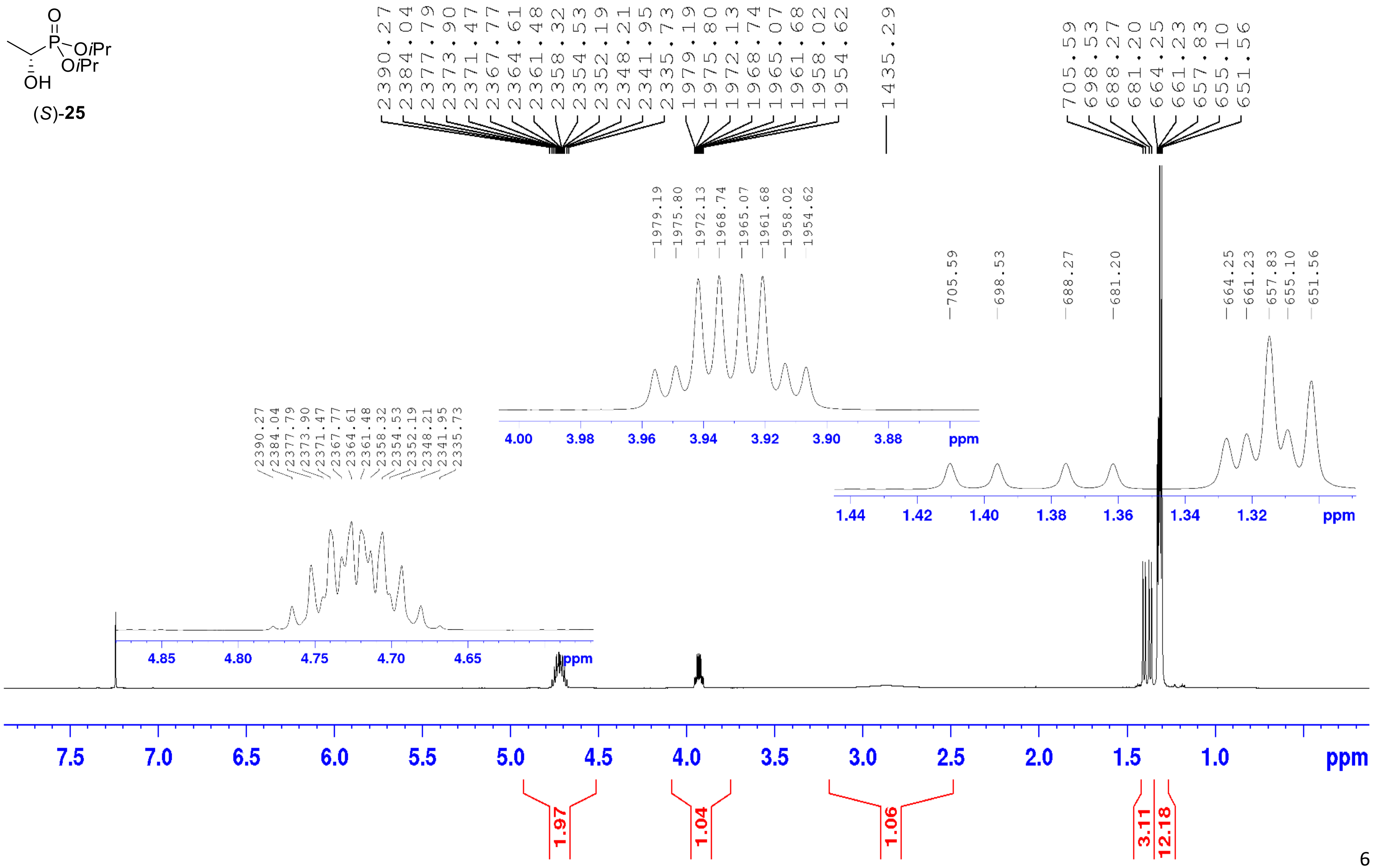

^{13}C NMR of (*S*)-diisopropyl 1-hydroxy-ethylphosphonate (176.12 MHz, $CDCl_3$) [(*S*)-25]:

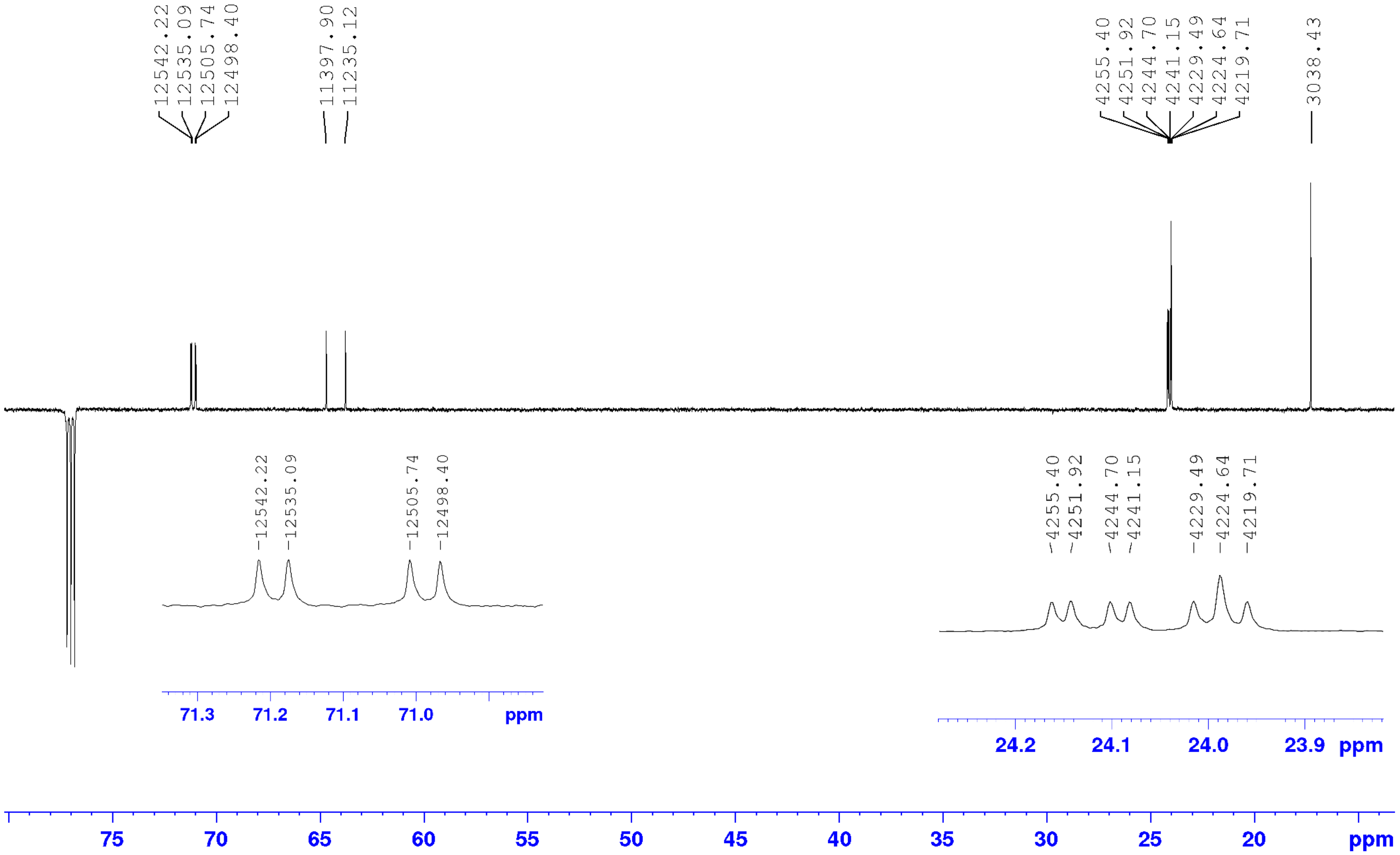

^{31}P NMR of (*S*)-diisopropyl 1-hydroxy-ethylphosphonate (161.98 MHz, $CDCl_3$) [(*S*)-25]:

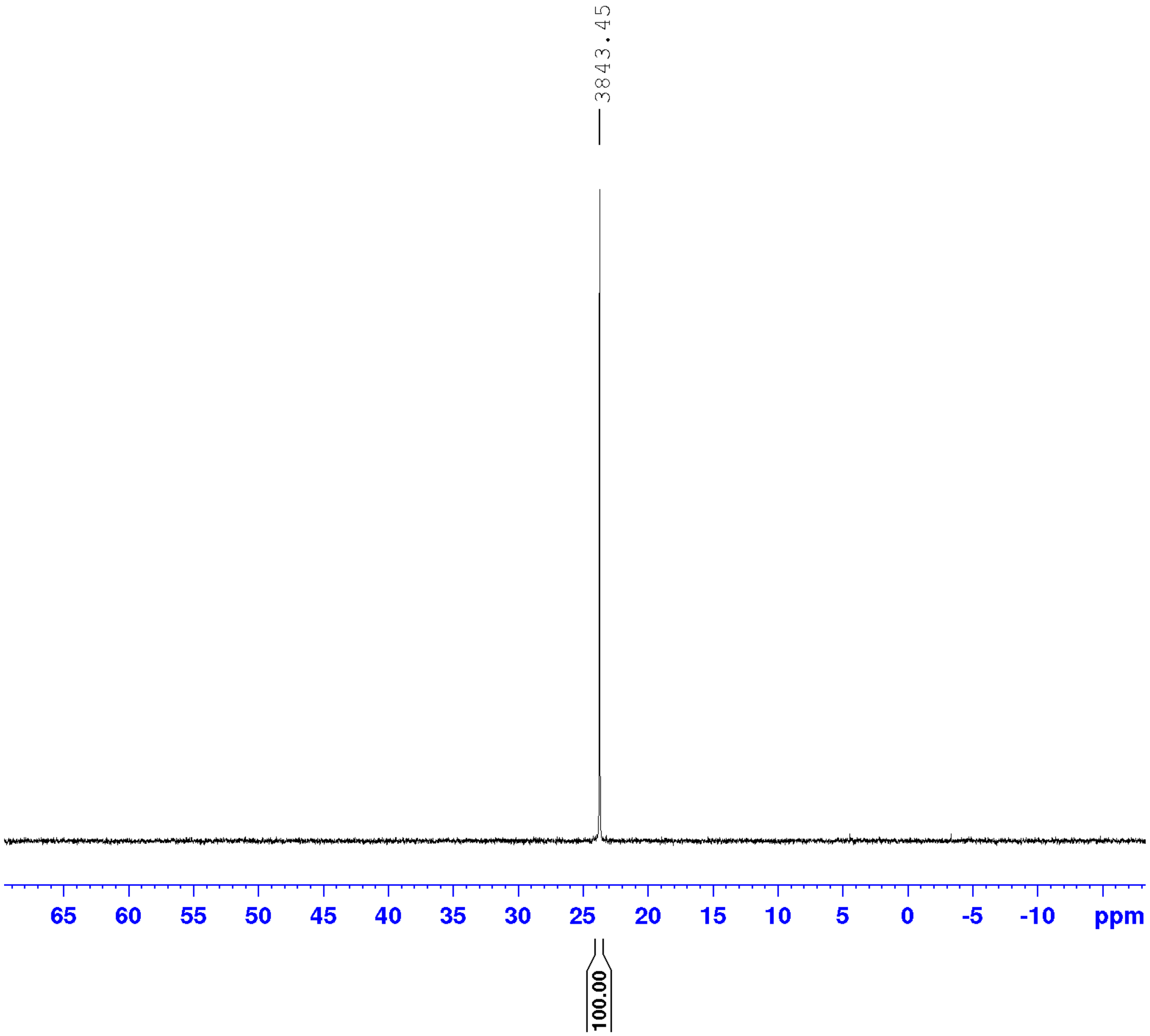

^{1}H NMR of (*R*)-1-hydroxy-ethylphosphonic acid sodium salt (700.40 MHz, $CDCl_3$) [(*R*)-45]:

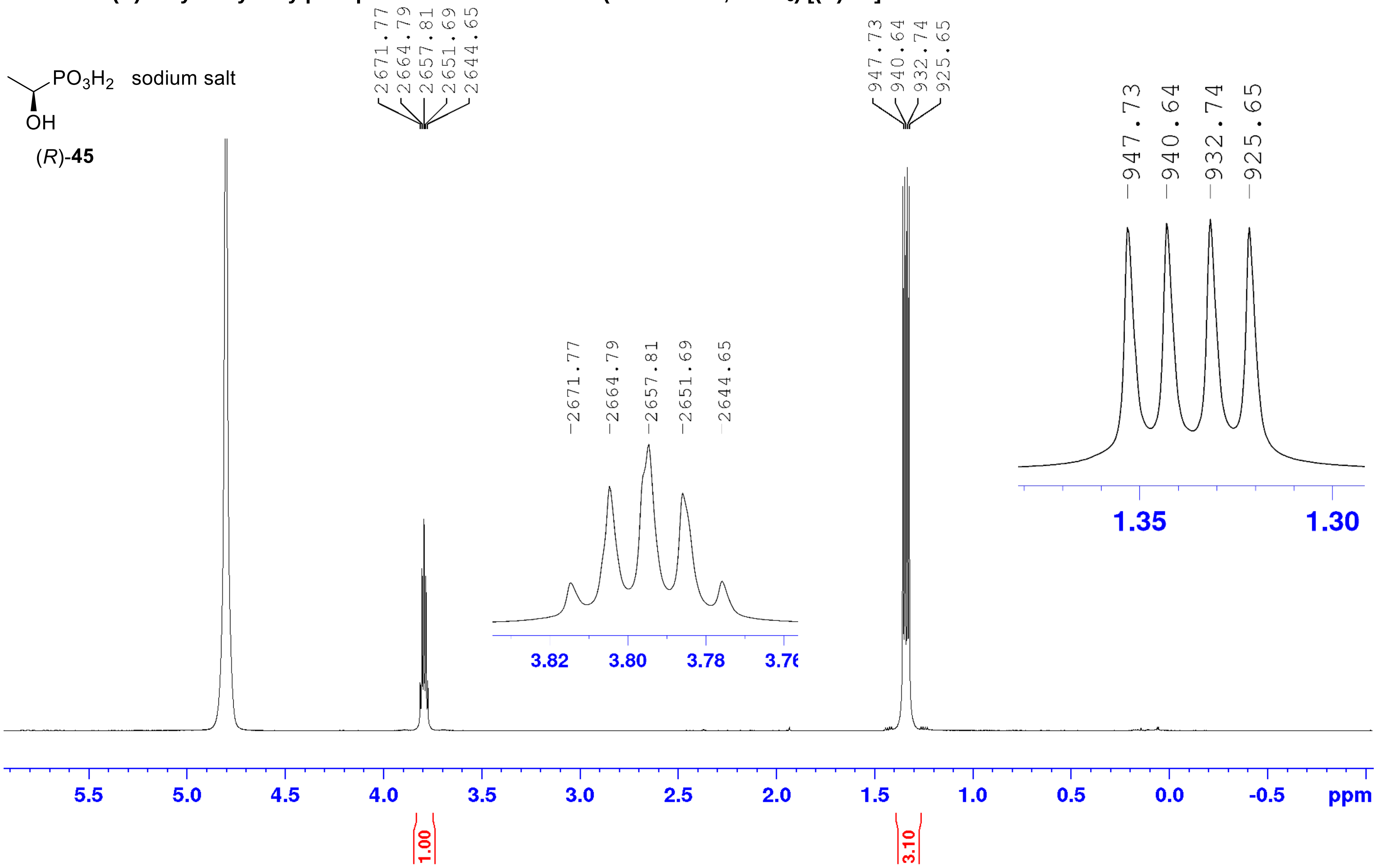

^{31}P NMR of (*R*)-1-hydroxy-ethylphosphonic acid sodium salt (162.03 MHz, $CDCl_3$) [(*R*)-45]:

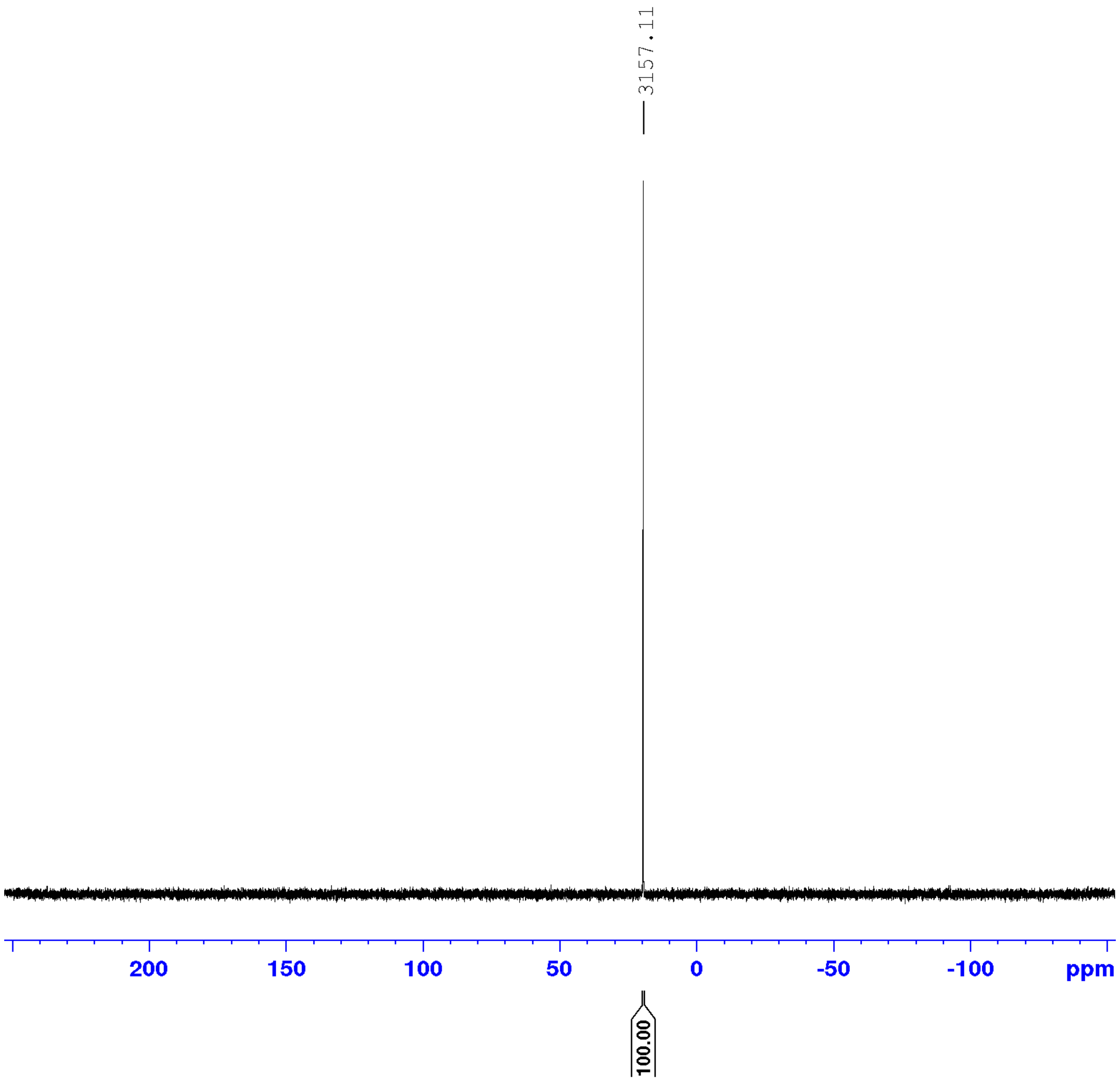

^{13}C NMR of (*R*)-1-hydroxy-ethylphosphonic acid sodium salt (176.12 MHz, $CDCl_3$) [(*R*)-45]:

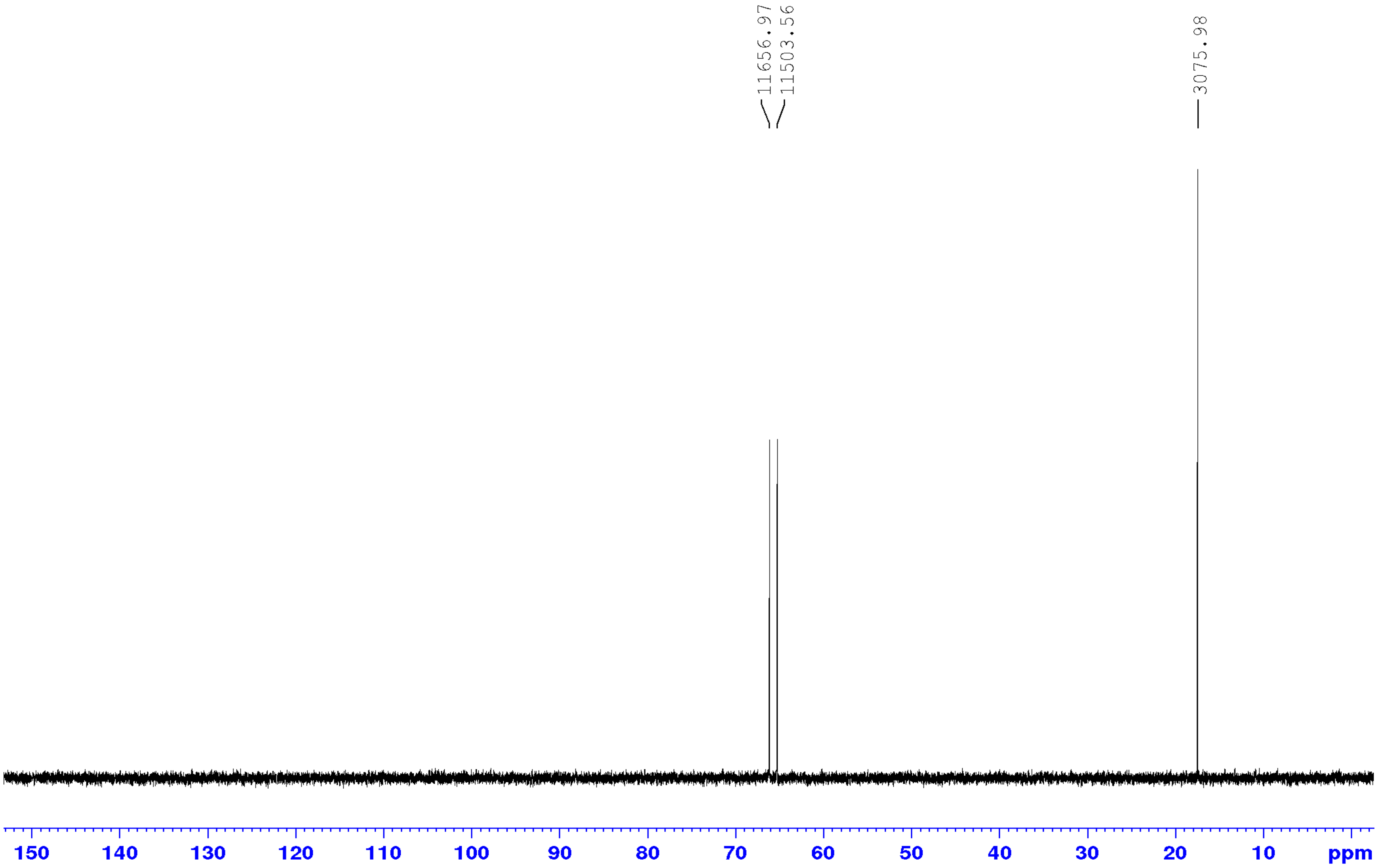

^{1}H NMR of (*R*)-1-[^{2}H]-diisopropyl 1-hydroxy-ethylphosphonate (400.27 MHz, $CDCl_3$) [(*R*)-1-[^{2}H]-45]:

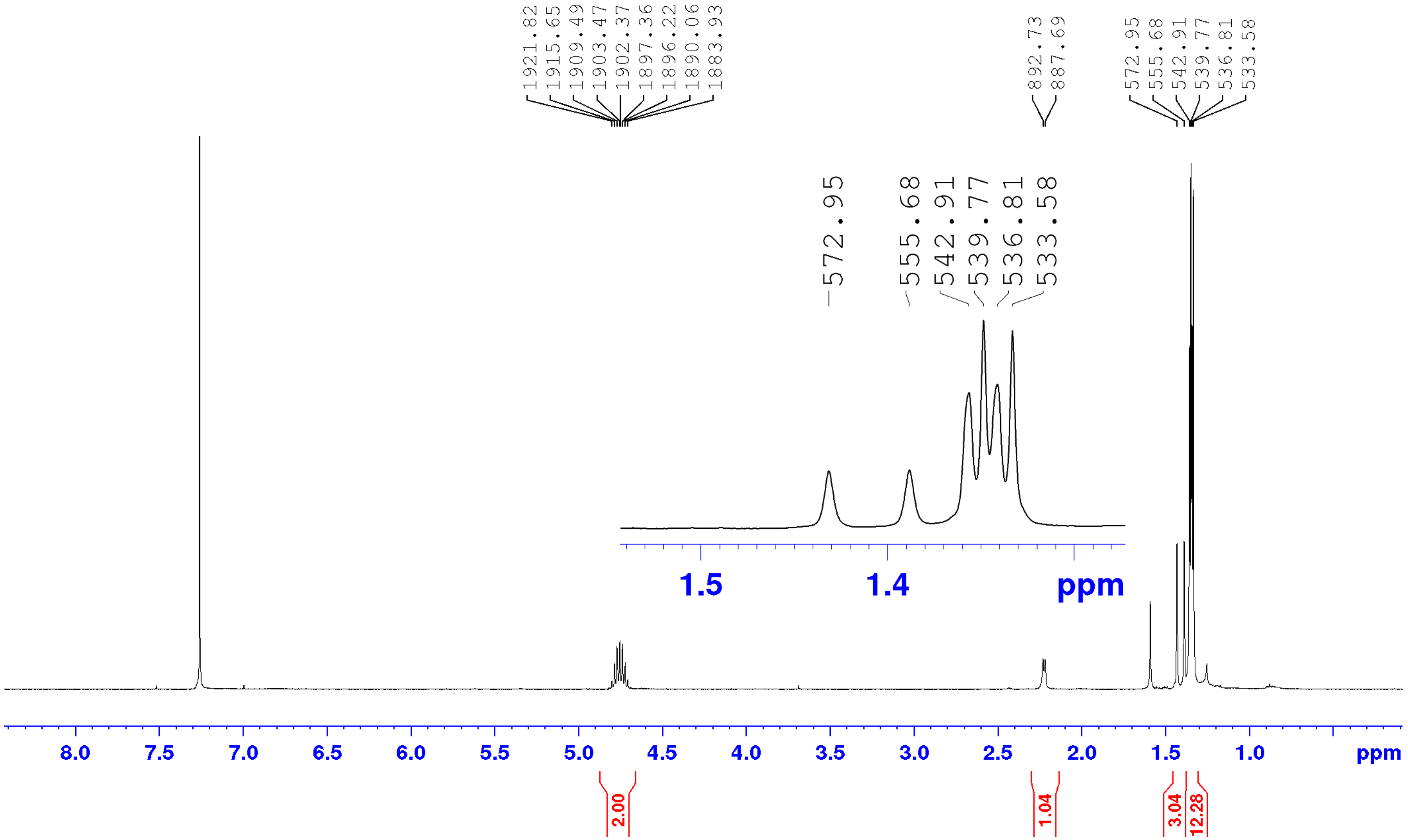

^{31}P NMR of (*R*)-1-[^{2}H]-diisopropyl 1-hydroxy-ethylphosphonate (162.03 MHz, $CDCl_3$) [(*R*)-45]:

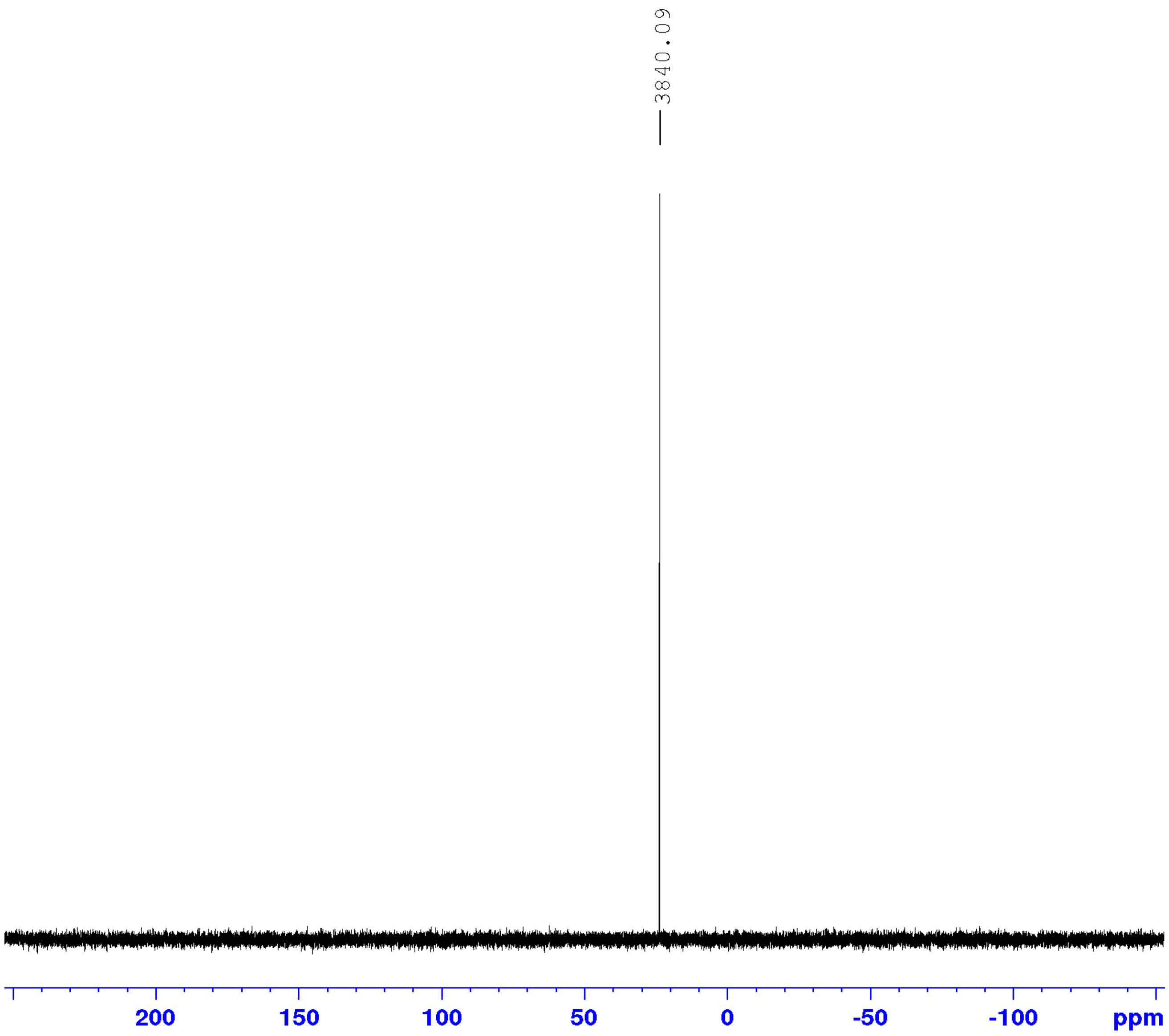

^{13}C NMR of (*R*)-1-[^{2}H]-diisopropyl 1-hydroxy-ethylphosphonate (150.93 MHz, CDCl$_3$) [(*R*)-45]:

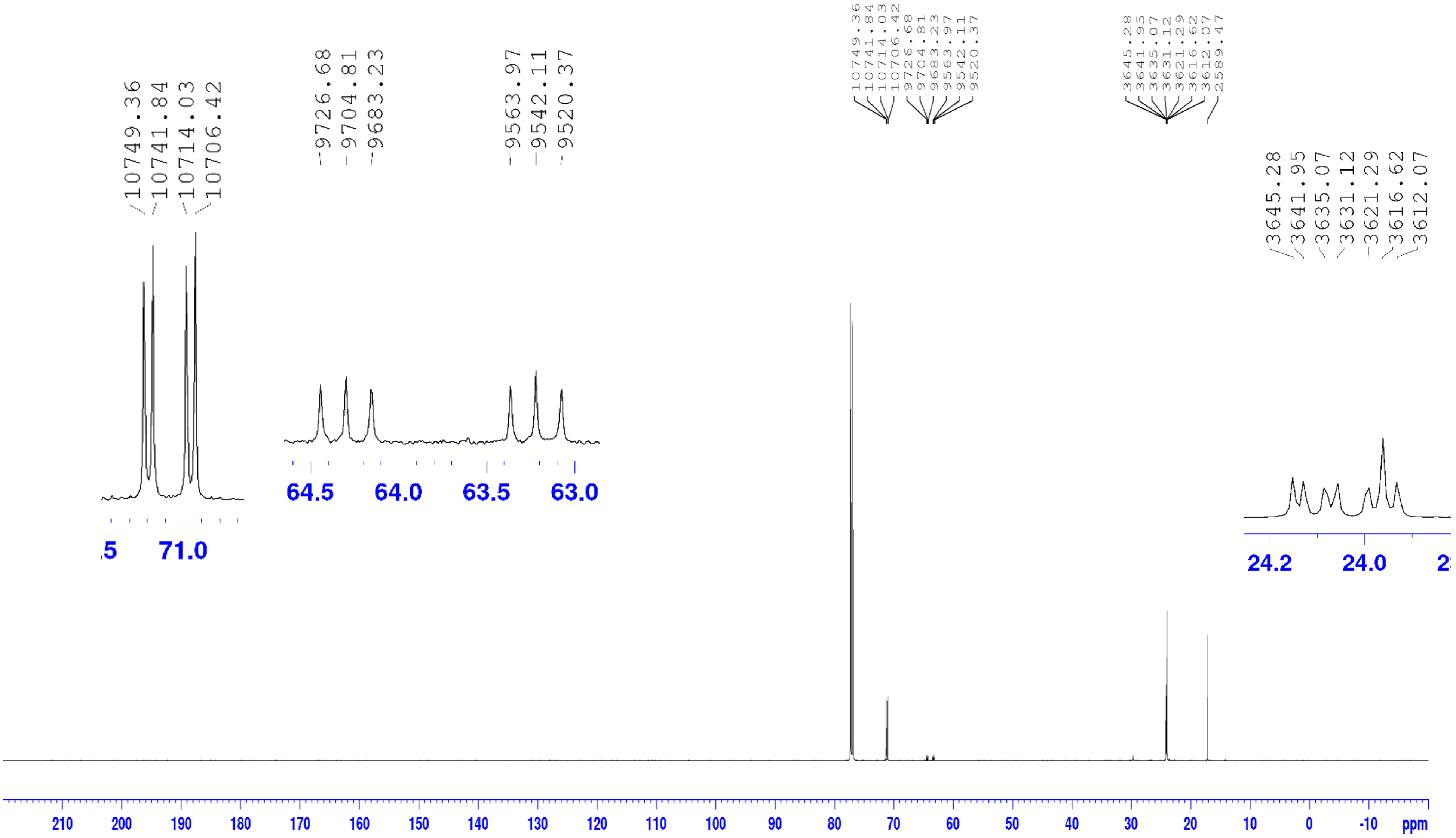

^{1}H NMR of (*R*)-diisopropyl 1-azido-ethylphosphonate (400.27 MHz, $CDCl_3$) [(*R*)-72]:

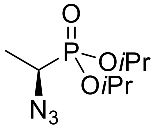

(*R*)-**72**

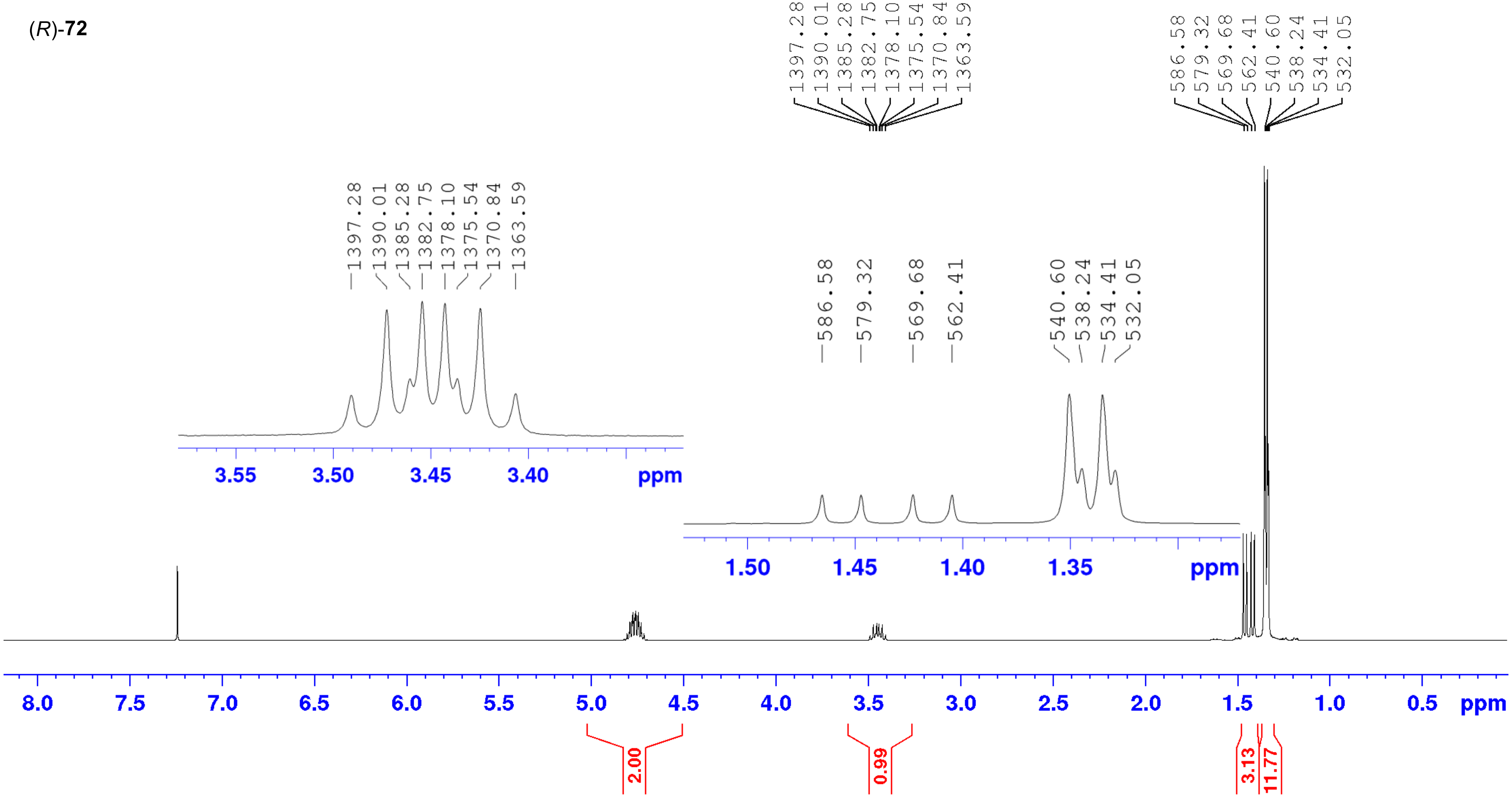

^{13}C NMR of (*R*)-diisopropyl 1-azido-ethylphosphonate [(*R*)-72] (150.93 MHz, $CDCl_3$):

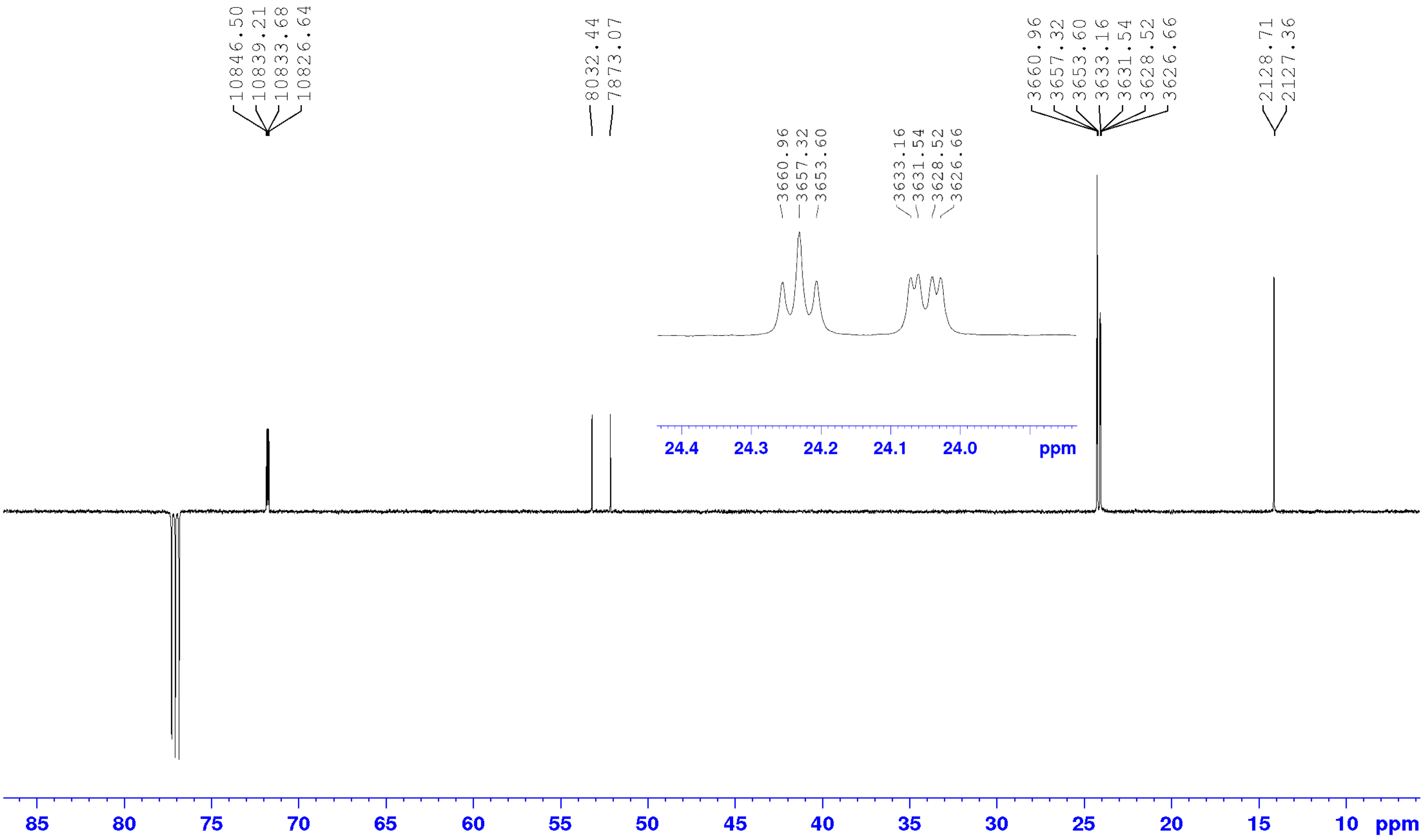

^{31}P NMR of (*R*)-diisopropyl 1-azido-ethylphosphonate (162.03 MHz, $CDCl_3$) [(*R*)-72]:

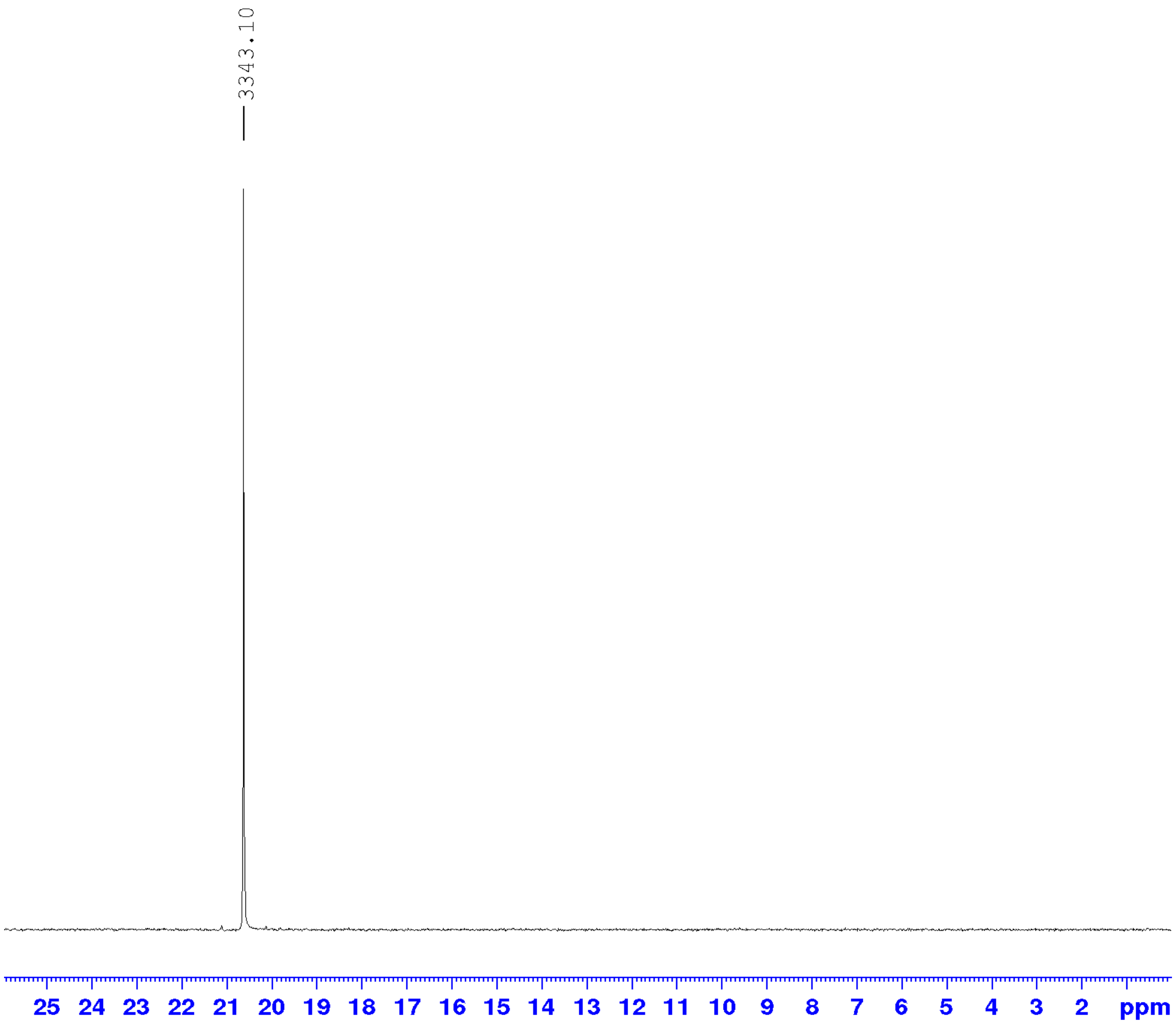

^{1}H NMR of (*R*)-1-amino-ethylphosphonic acid, (*R*)-phosphaalanine (400.27 MHz, D_2O) [(*R*)-59]:

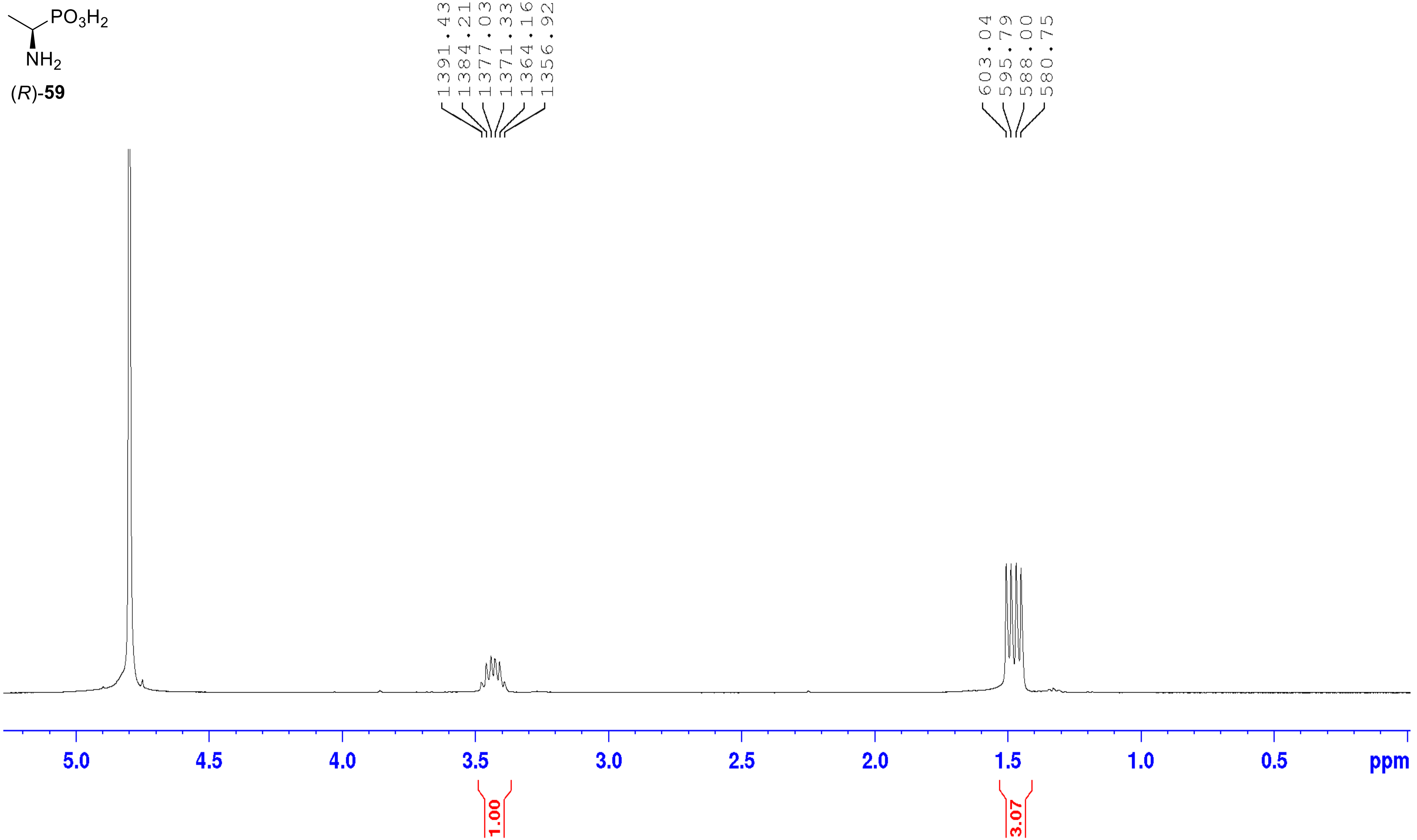

^{13}C NMR of (*R*)-1-amino-ethylphosphonic acid, (*R*)-phosphaalanine (100.65 MHz, D_2O) [(R)-59]:

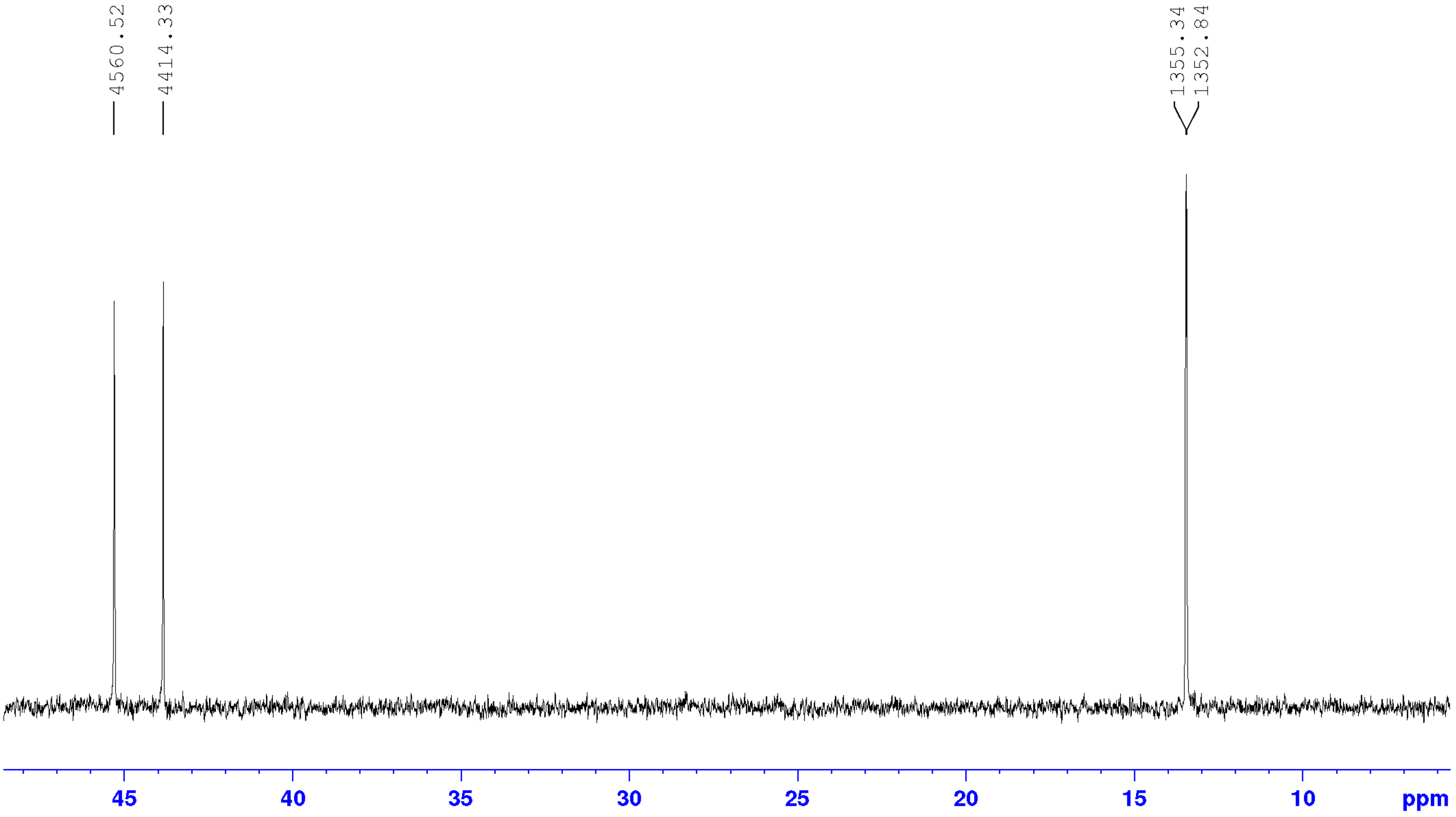

^{31}P NMR of (*R*)-1-amino-ethylphosphonic acid, (*R*)-phosphaalanine (162.03 MHz, D_2O) [(*R*)-59]:

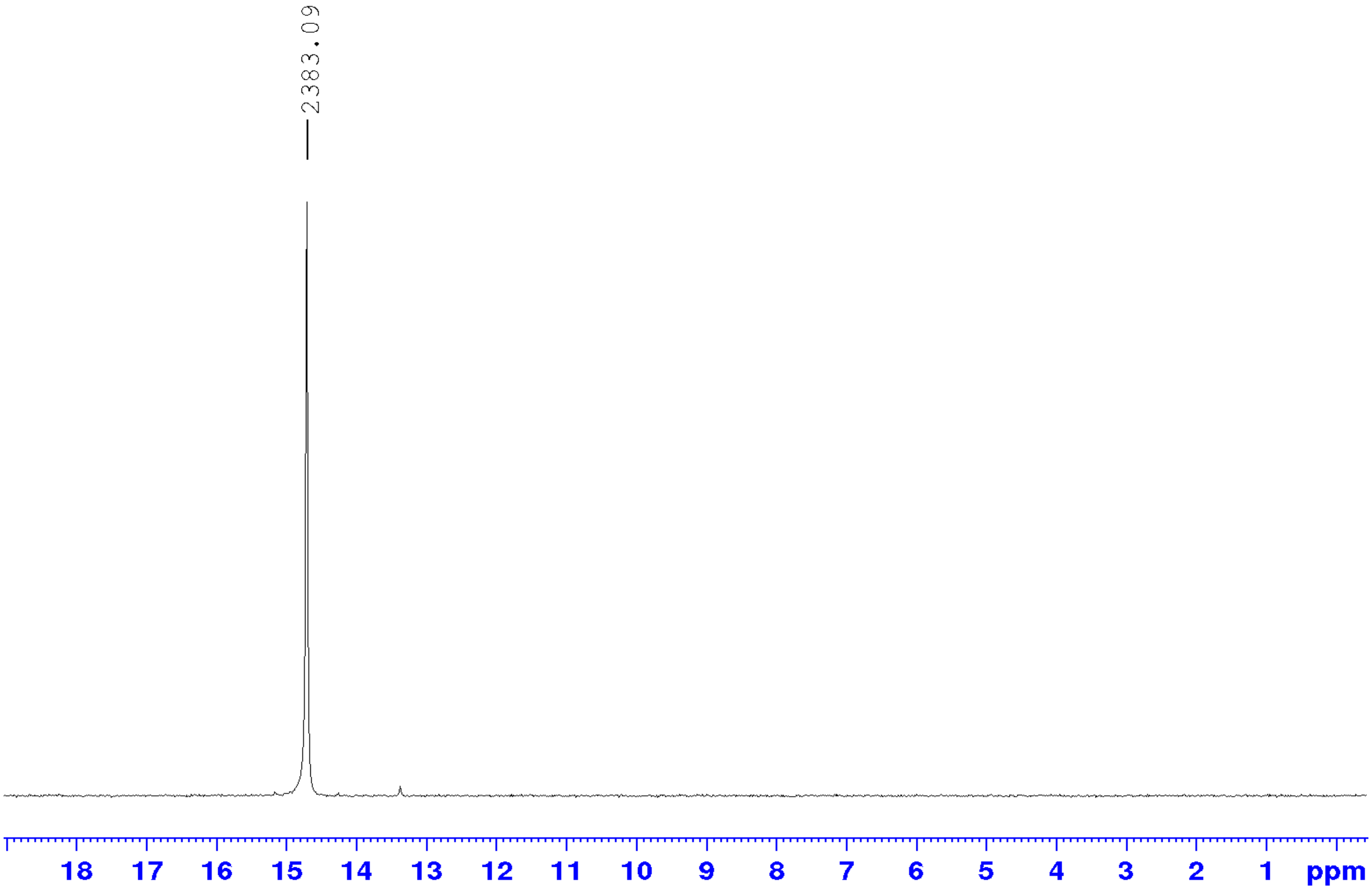

ee-determination of (*R*)-59 by chiral stationary phase HPLC after derivatization as shown below

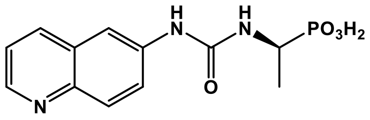

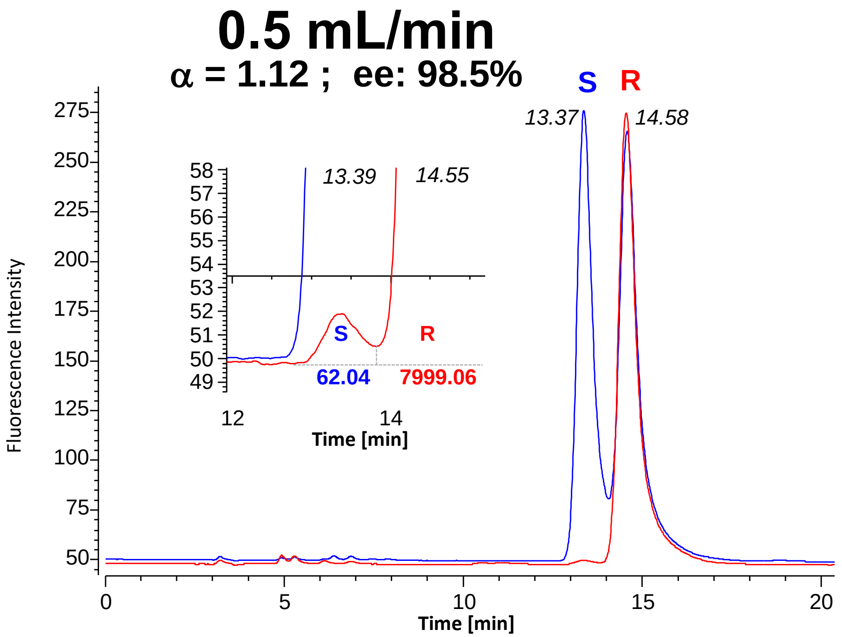

Column: OH-QNAX (150 x 4 mm, 5µm)

Mobile Phase: 2M H_3PO_4 : MeOH (1:9, v/v), pH 4.00, adjusted with TEA, 40°C

^{1}H NMR of diisopropyl 1-oxo-2-methylpropylphosphonate (400.27 MHz, $CDCl_3$) (9):

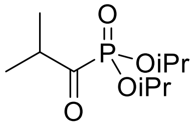

9

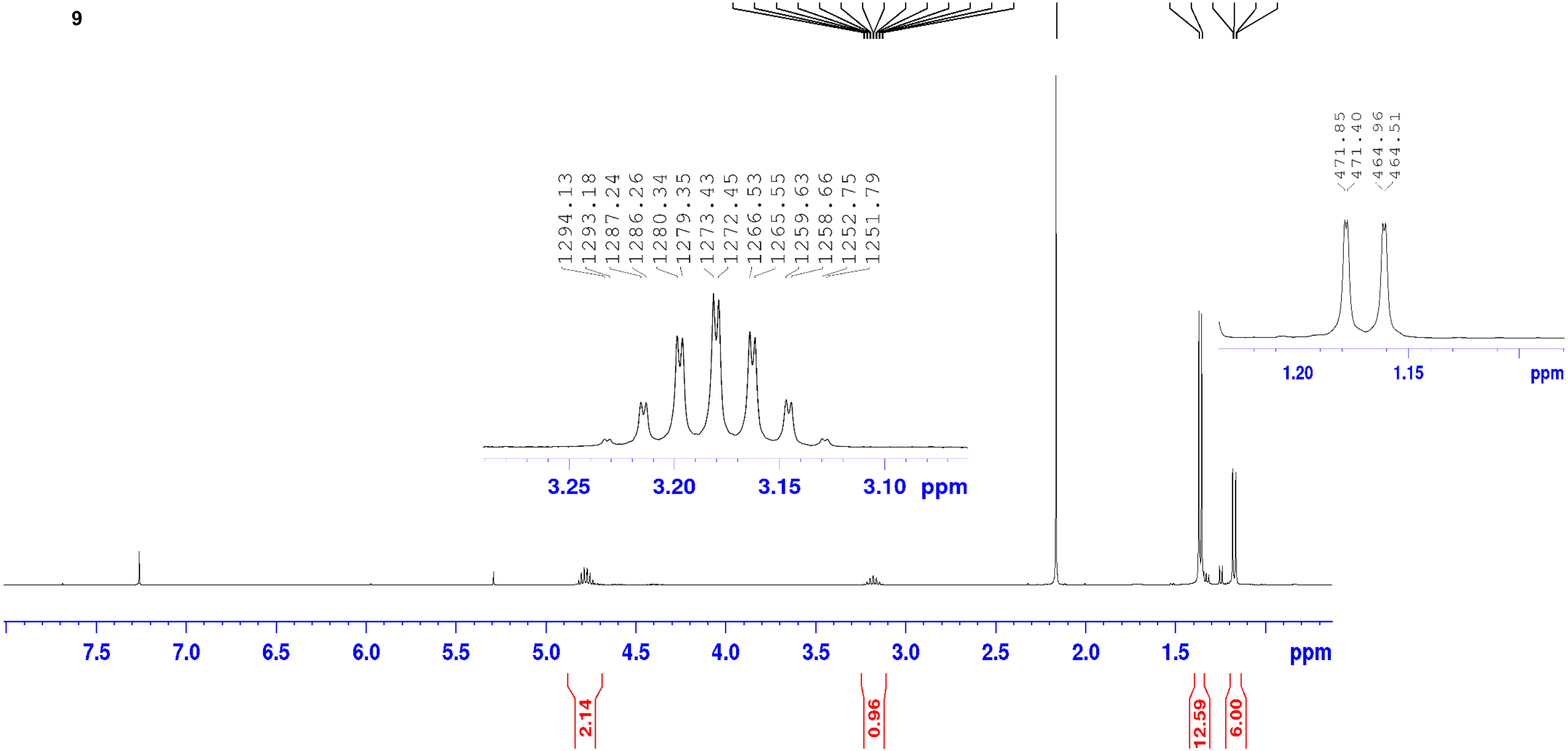

^{31}P NMR of diisopropyl 1-oxo-2-methylpropylphosphonate (162.03 MHz, $CDCl_3$) (9):

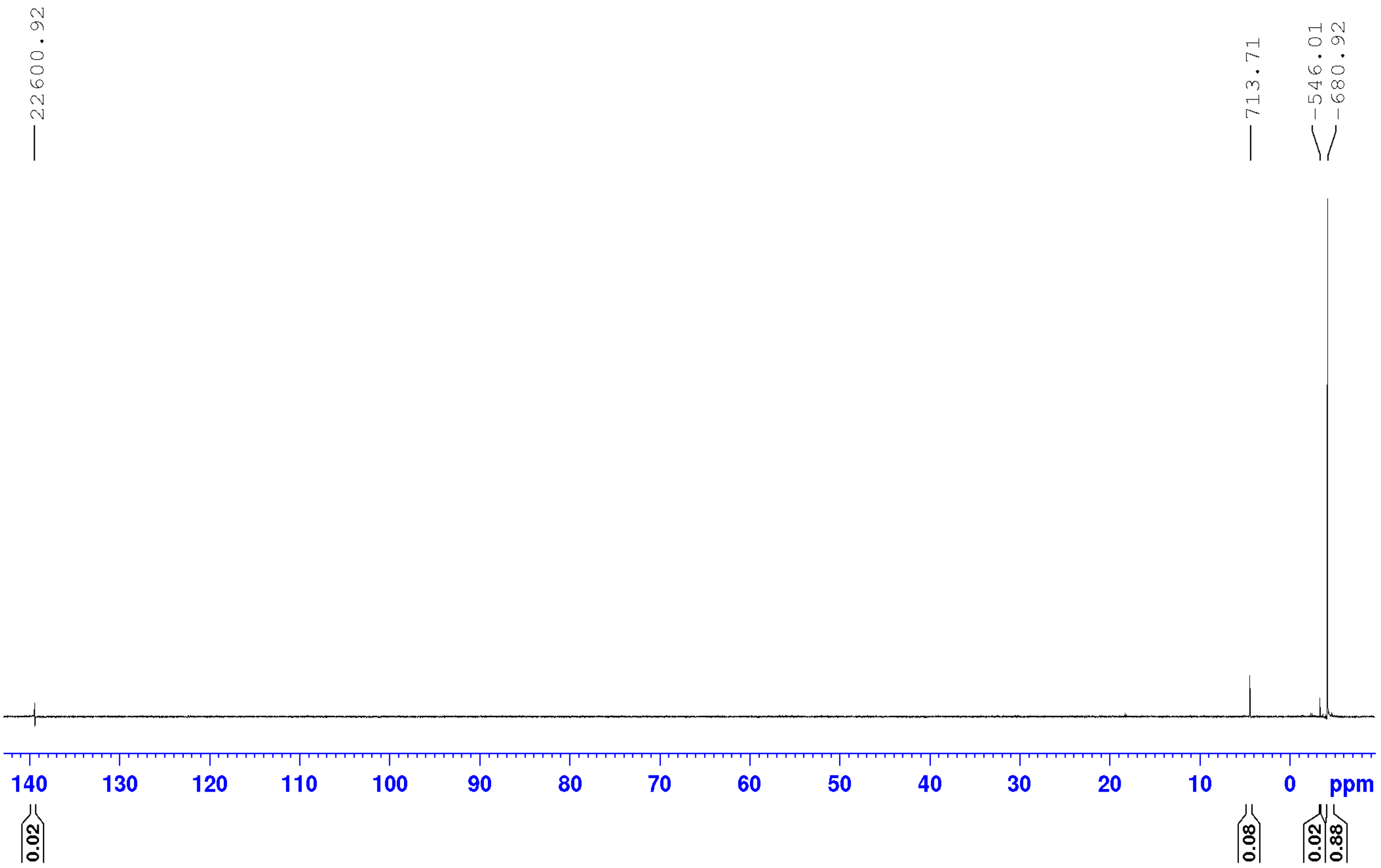

^{1}H NMR of (*S*)-diisopropyl 1-hydroxy-2-methylpropylphosphonate (400.27 MHz, CDCl$_3$) [(*S*)-26]:

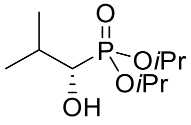

(*S*)-**26**

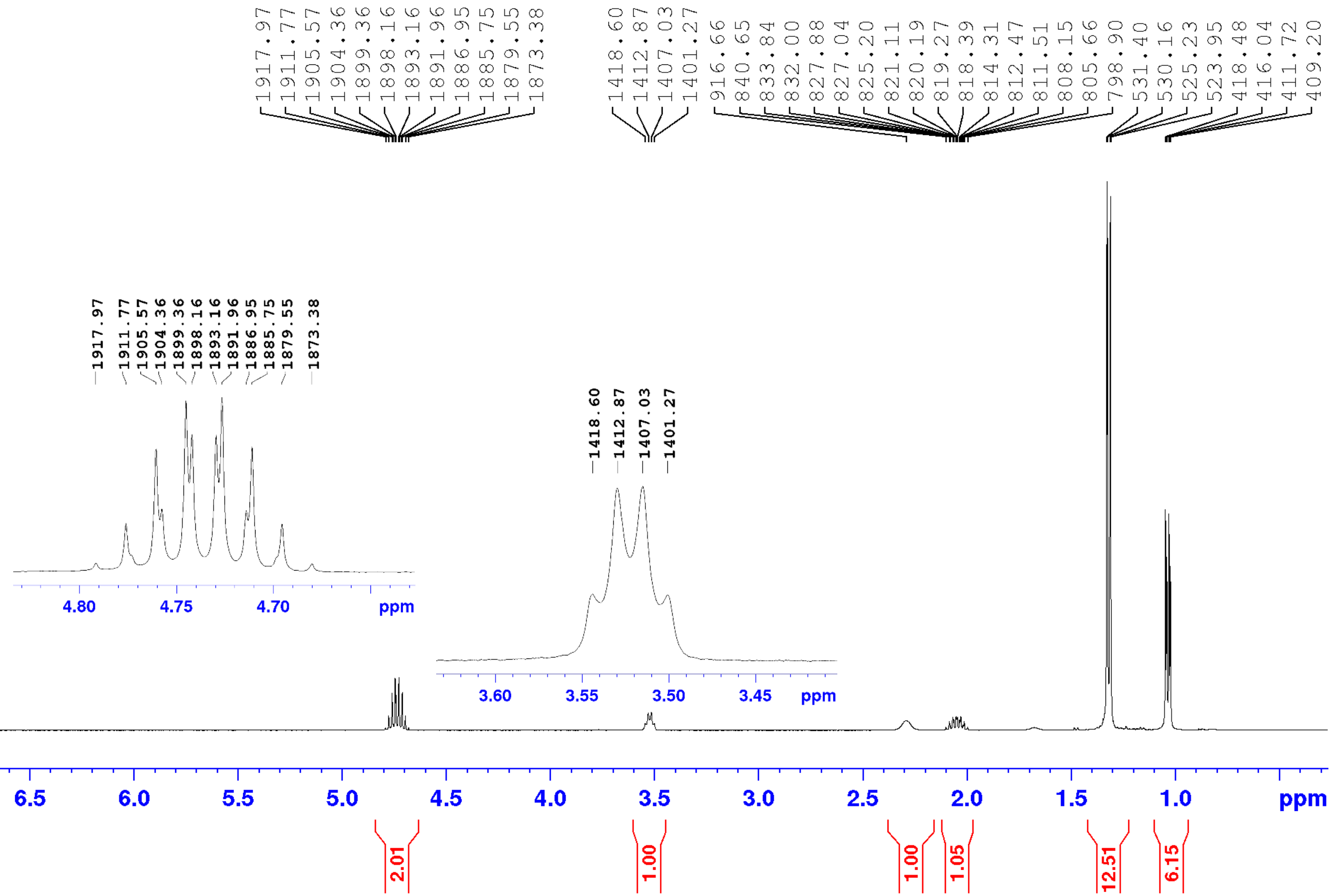

^{13}C NMR of (*S*)-diisopropyl 1-hydroxy-2-methylpropylphosphonate (176.12 MHz, $CDCl_3$) [(*S*)-26]:

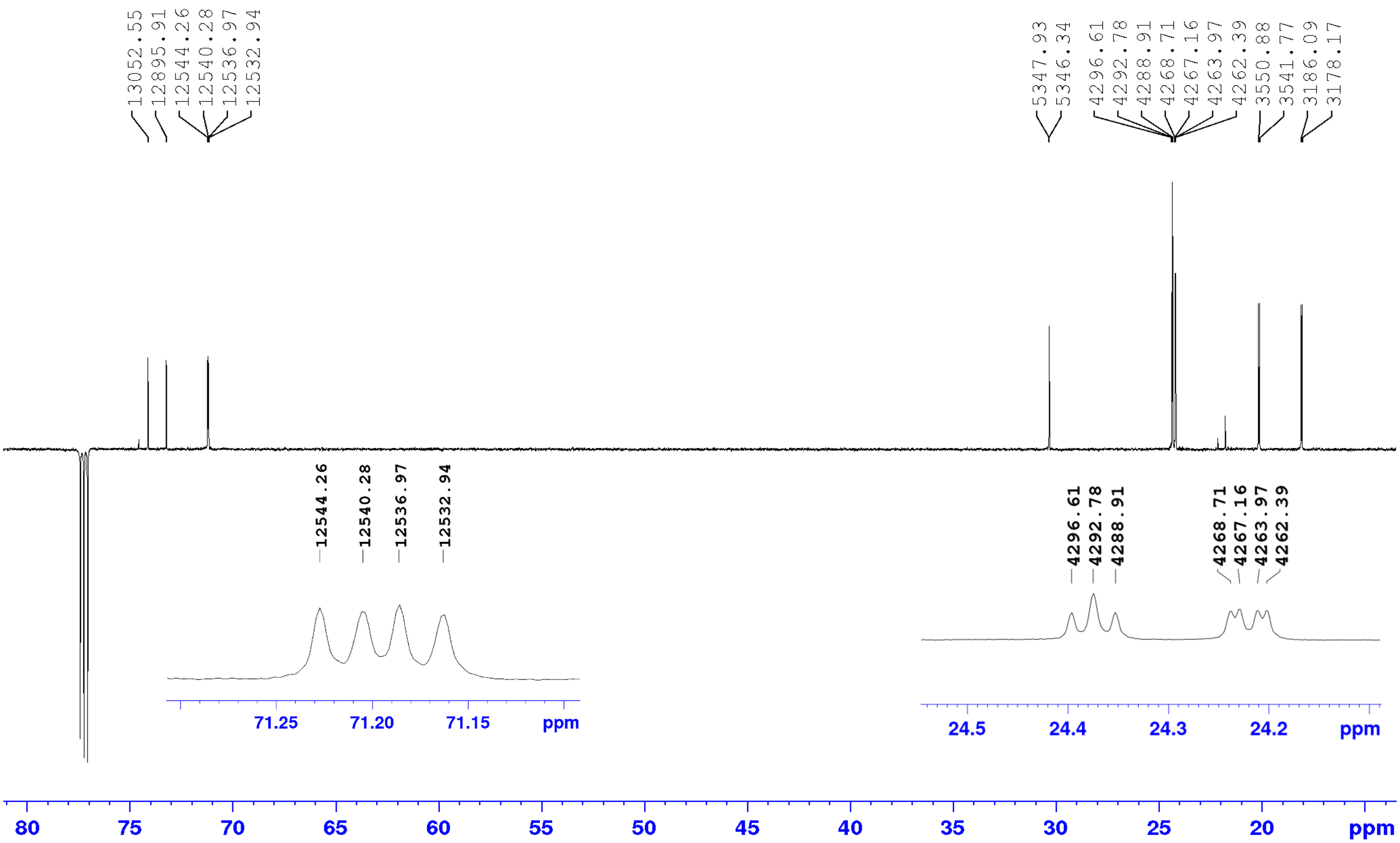

^{31}P NMR of (*S*)-diisopropyl 1-hydroxy-2-methylpropylphosphonate (162.03 MHz, $CDCl_3$) [(*S*)-26]:

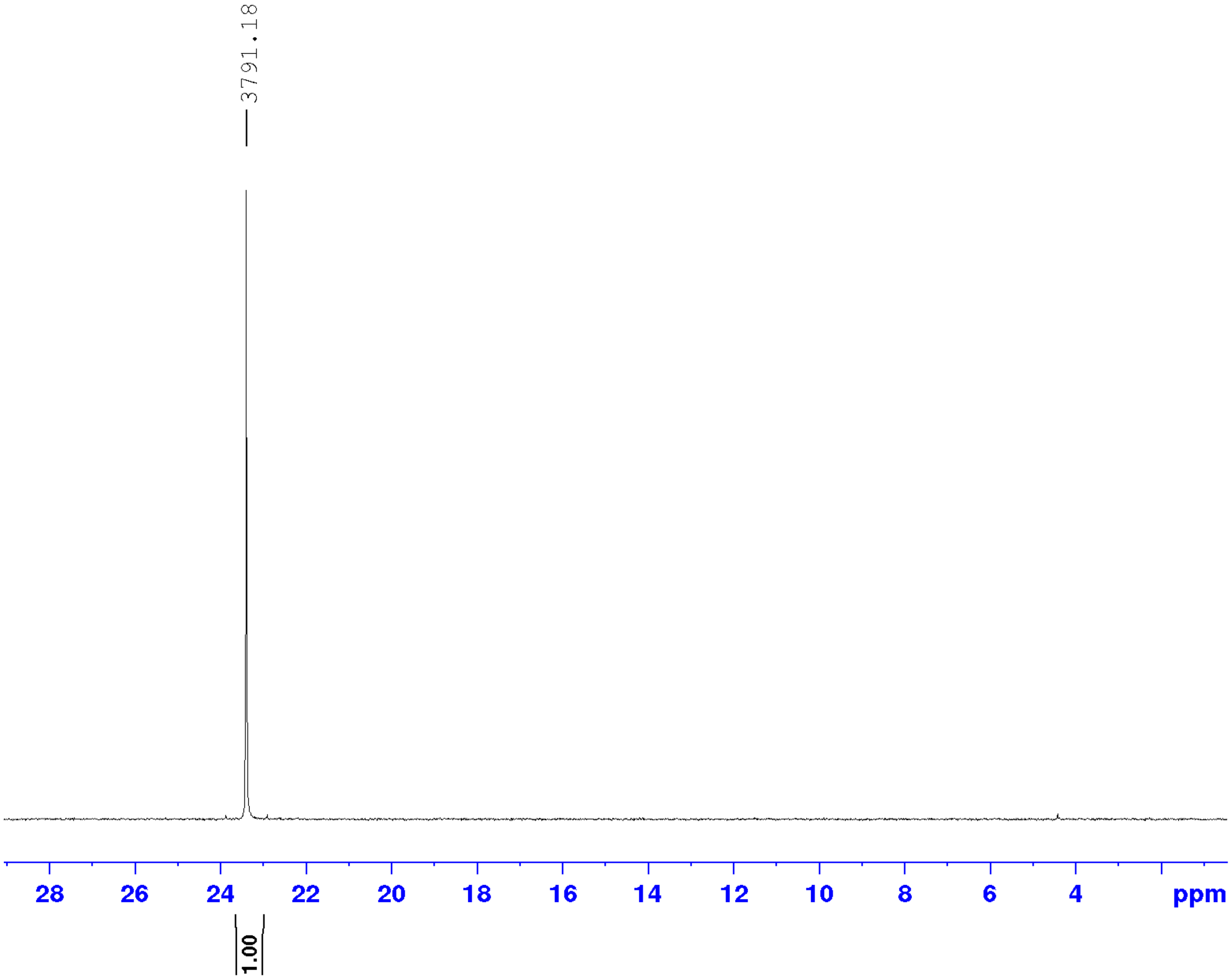

^{1}H NMR of (*R*)-diisopropyl 1-azido-2-methylpropylphosphonate (400.27 MHz, $CDCl_3$) [(*R*)-73]:

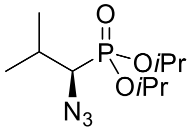

(*R*)-**73**

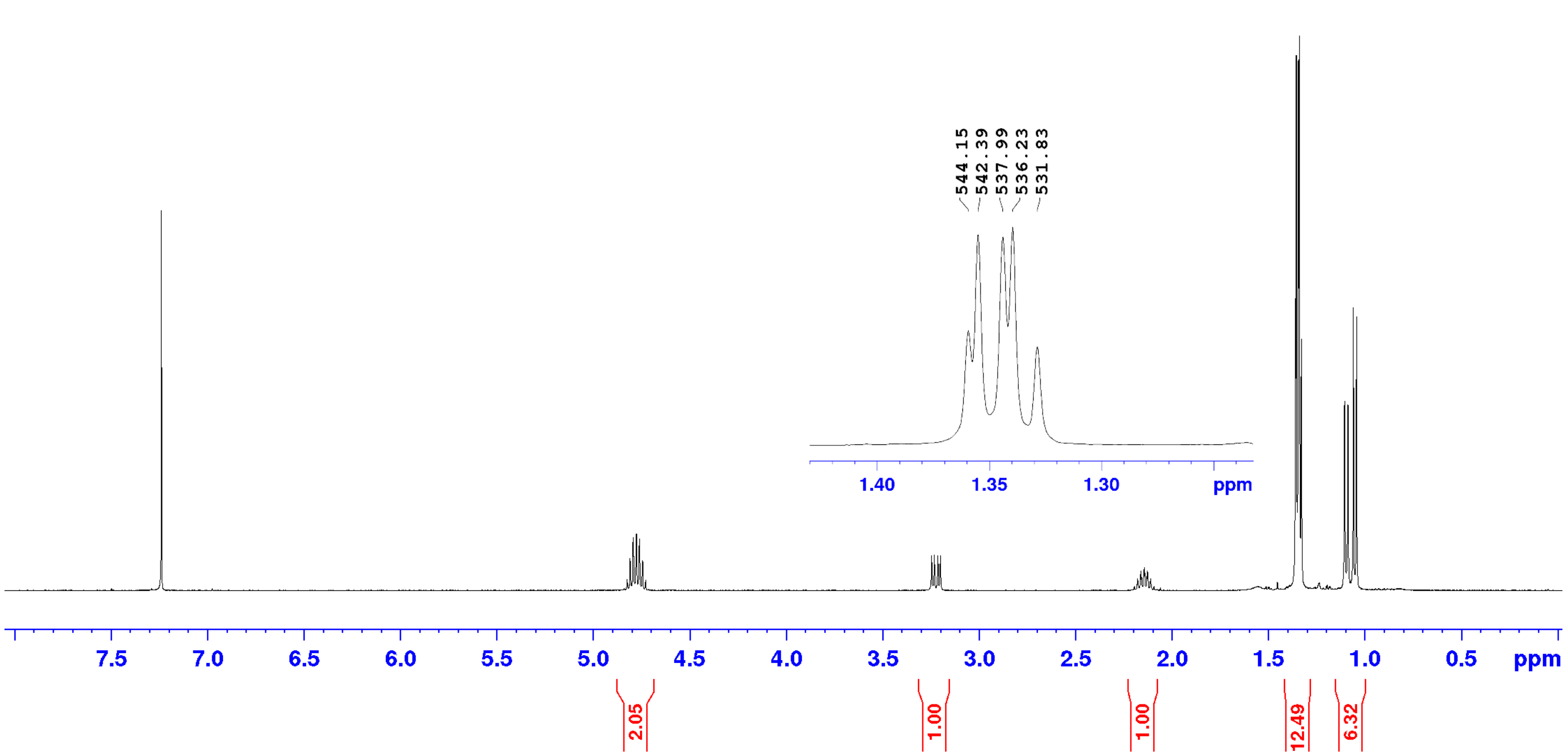

^{13}C NMR of (*R*)-diisopropyl 1-azido-2-methylpropylphosphonate (150.93 MHz, CDCl$_3$) [(*R*)-73]:

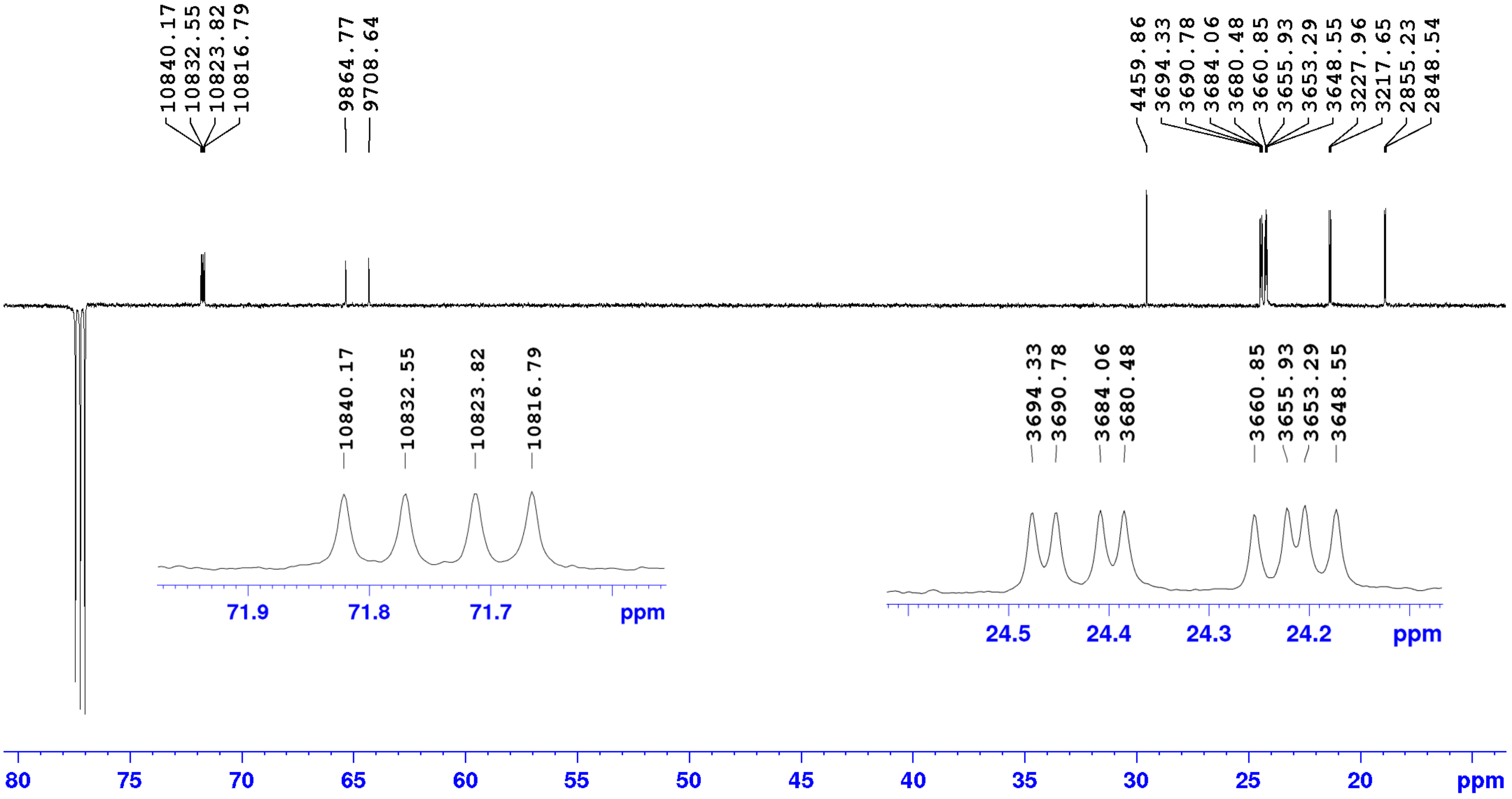

^{31}P NMR of (*R*)-diisopropyl 1-azido-2-methylpropylphosphonate (162.03 MHz, $CDCl_3$) [(*R*)-73]:

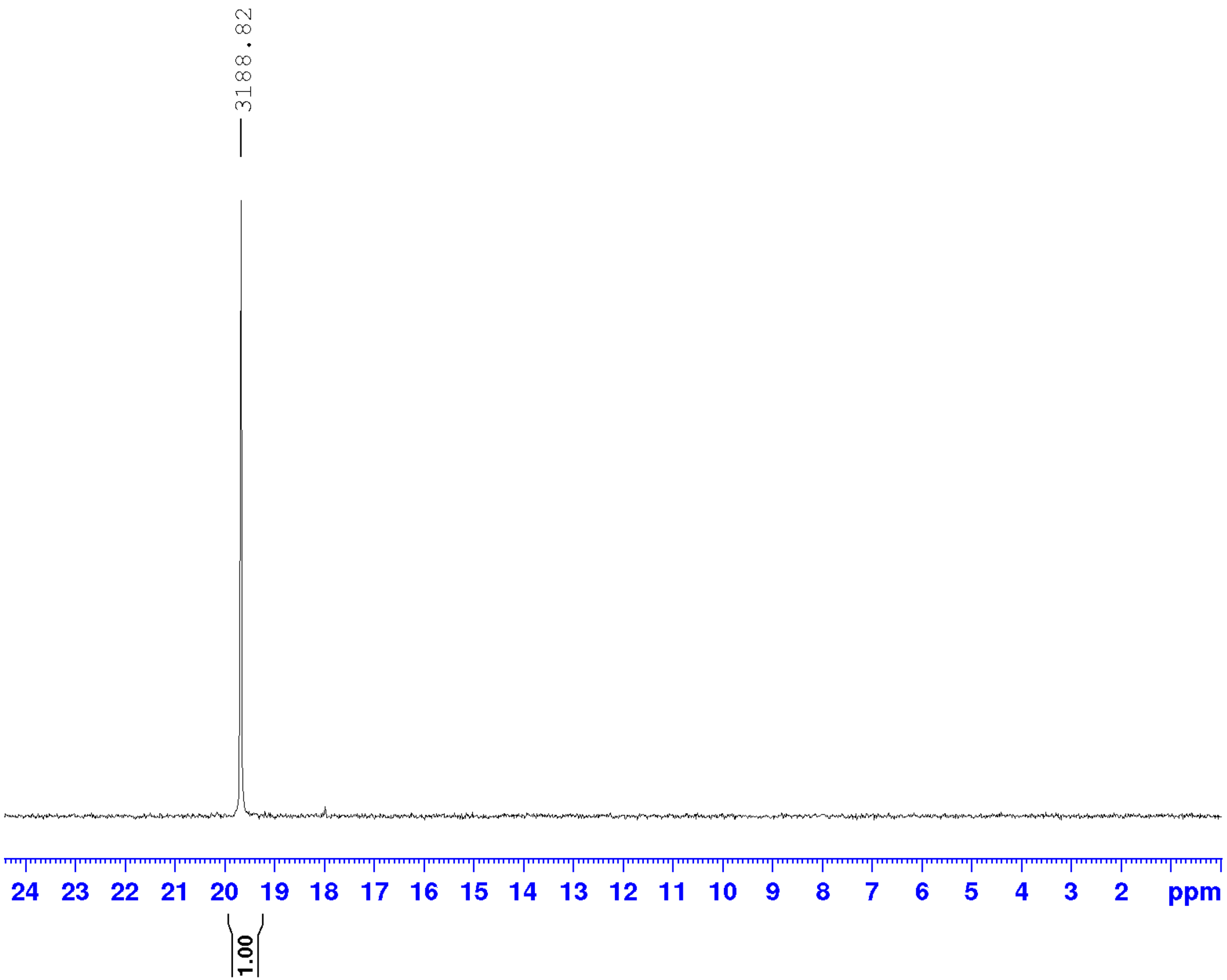

^{1}H NMR of (*R*)-1-amino-2-methylpropylphosphonic acid, (*R*)-phosphavaline (400.27 MHz, D_2O) [(*R*)-60]:

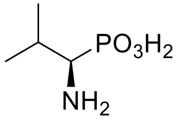

(*R*)-**60**

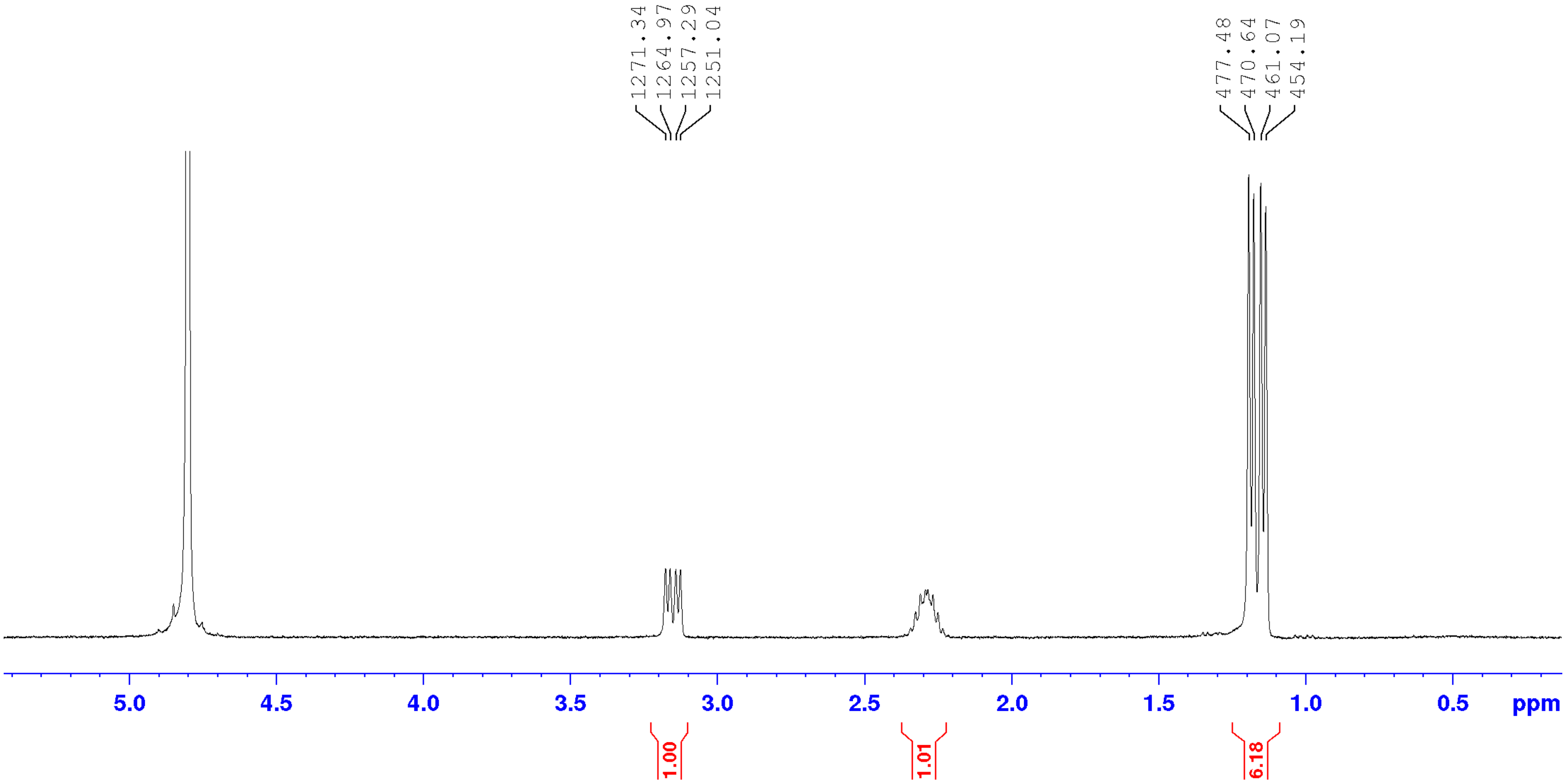

^{13}C NMR of (*R*)-1-amino-2-methylpropylphosphonic acid, (*S*)-phosphavaline (100.65 MHz, D_2O) [(*R*)-60]:

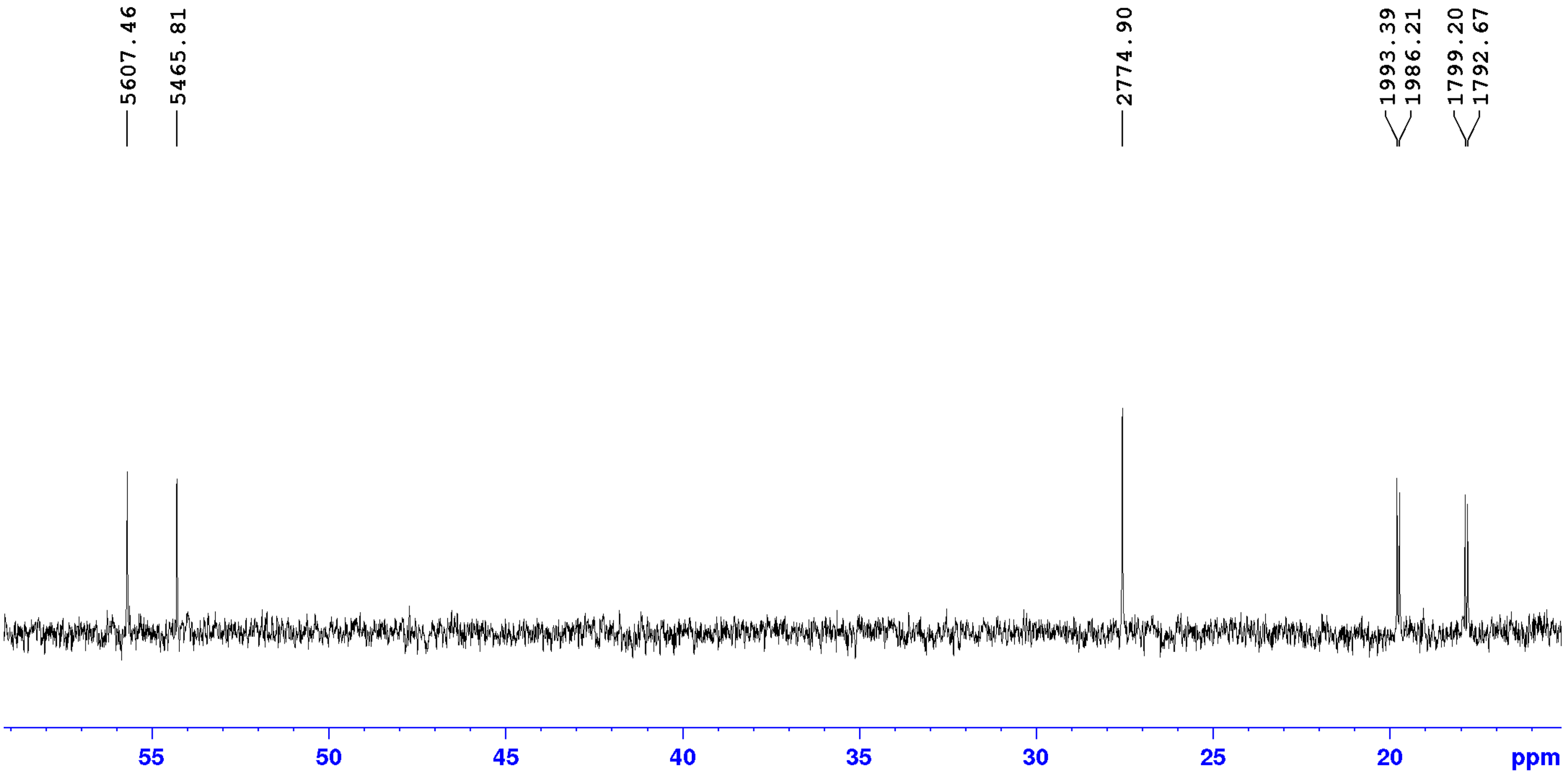

^{31}P NMR of (*R*)-1-amino-2-methylpropylphosphonic acid, (*R*)-phosphavaline (162.03 MHz, D_2O) [(*R*)-60]:

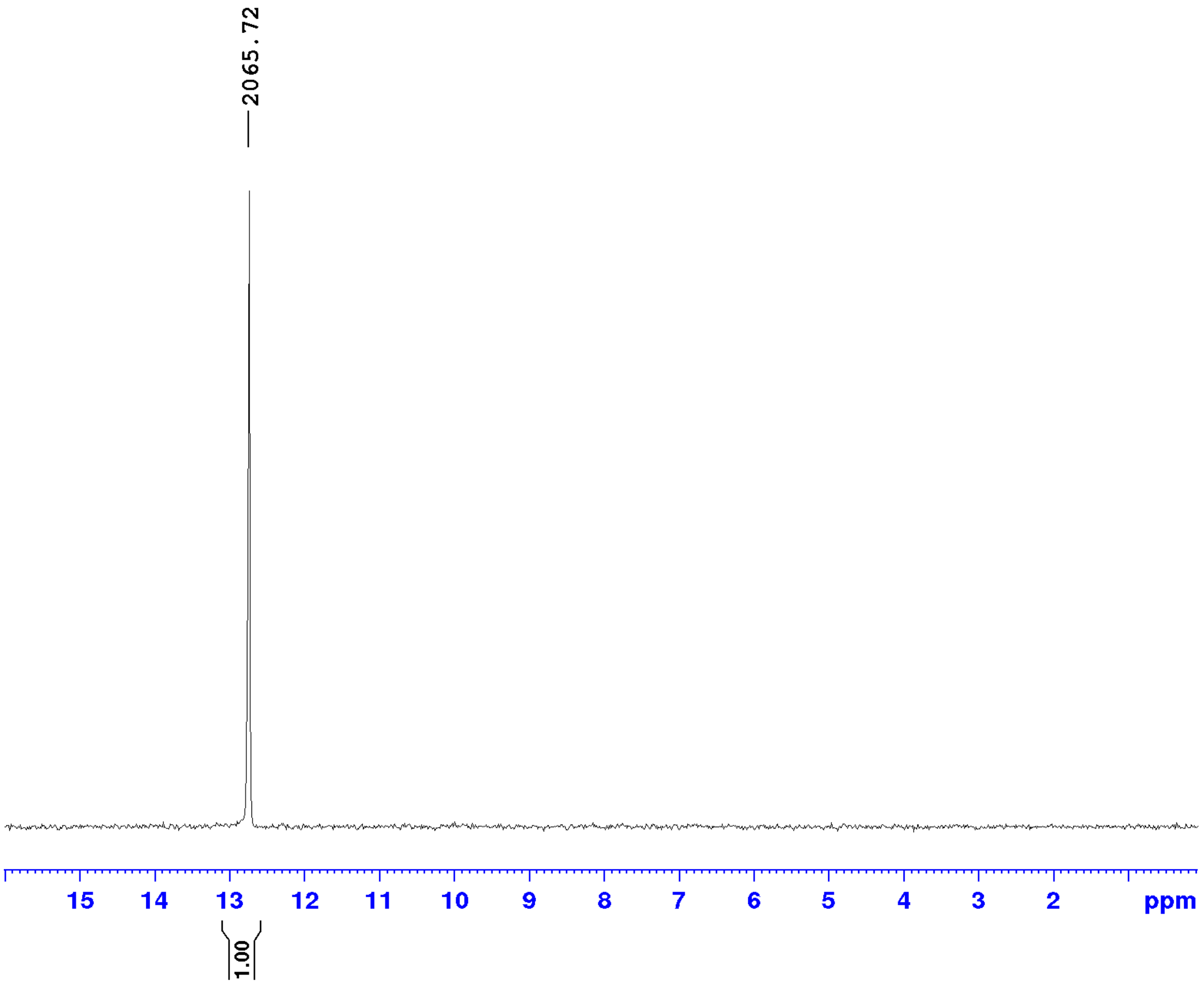

^{31}P NMR of diisopropyl 1-oxo-3-methylbutylphosphonate (162.04 MHz, $CDCl_3$) (10):

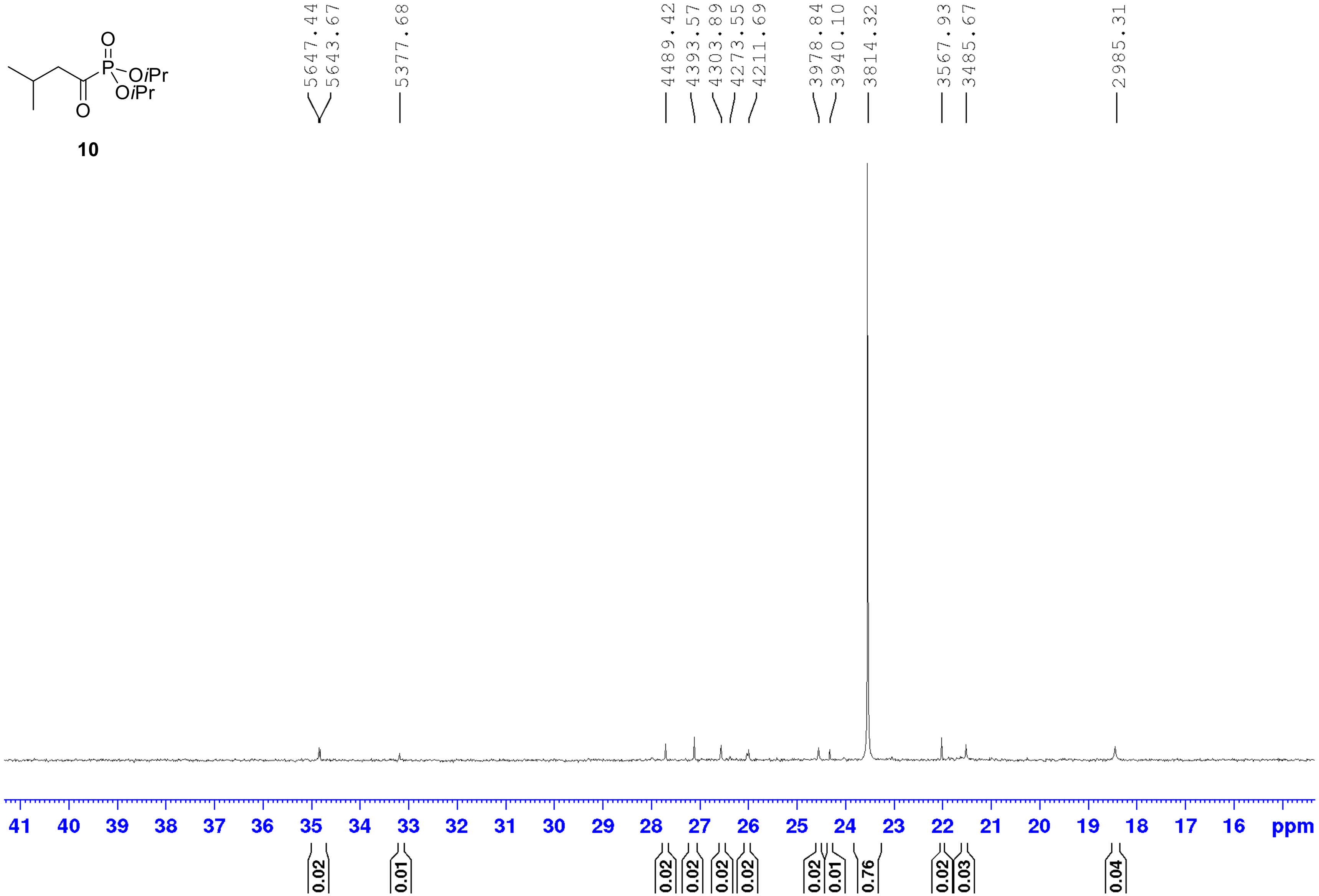

^{1}H NMR of (*S*)-diisopropyl 1-hydroxy-3-methylbutylphosphonate (600.25 MHz, $CDCl_3$) [(*S*)-27]:

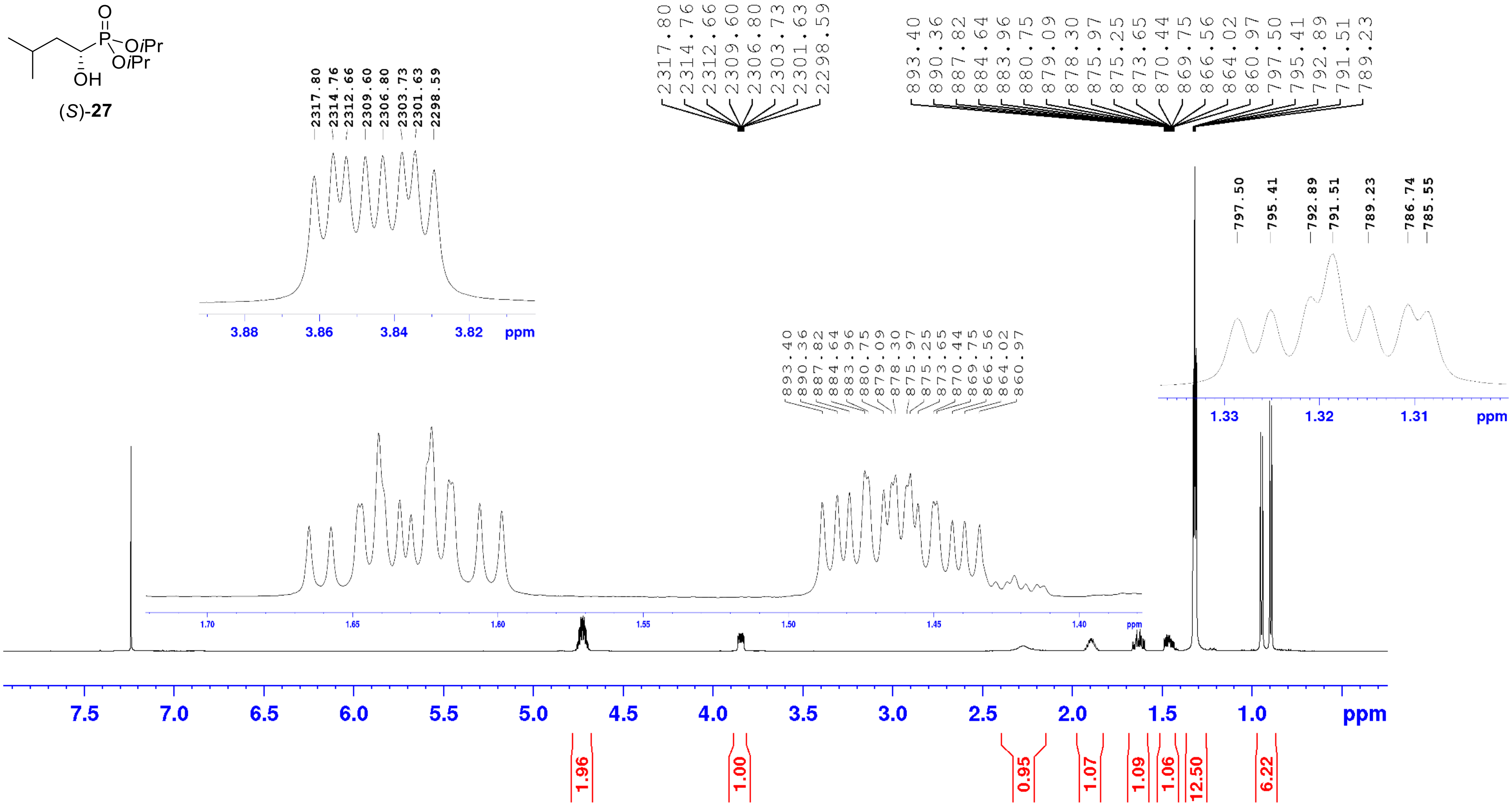

^{13}C NMR of (*S*)-Diisopropyl 1-hydroxy-3-methylbutylphosphonate (150.93 MHz, $CDCl_3$) [(*S*)-27]:

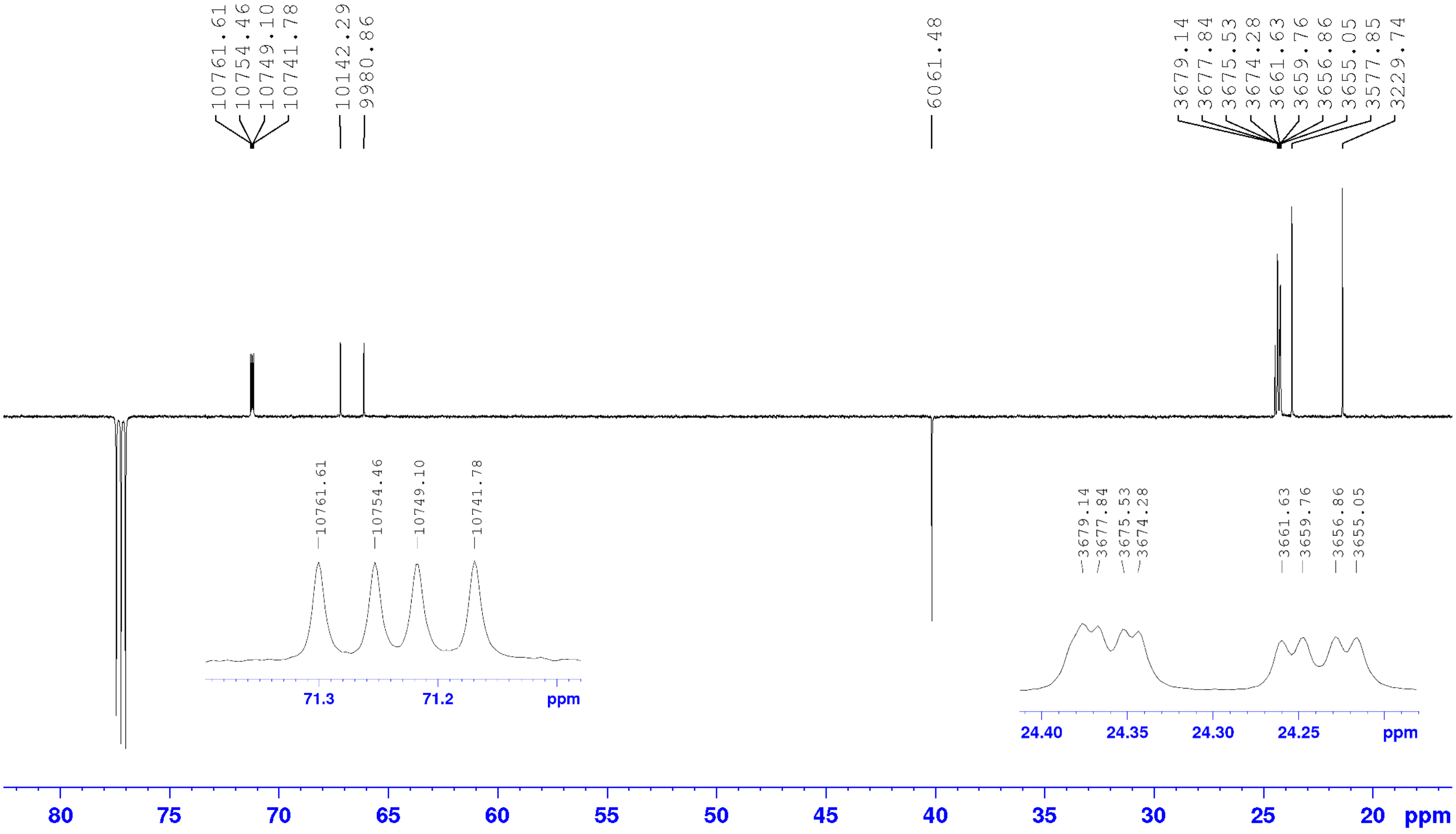

^{31}P NMR of (*S*)-Diisopropyl 1-hydroxy-3-methylbutylphosphonate (162.03 MHz, $CDCl_3$) [(*S*)-27]:

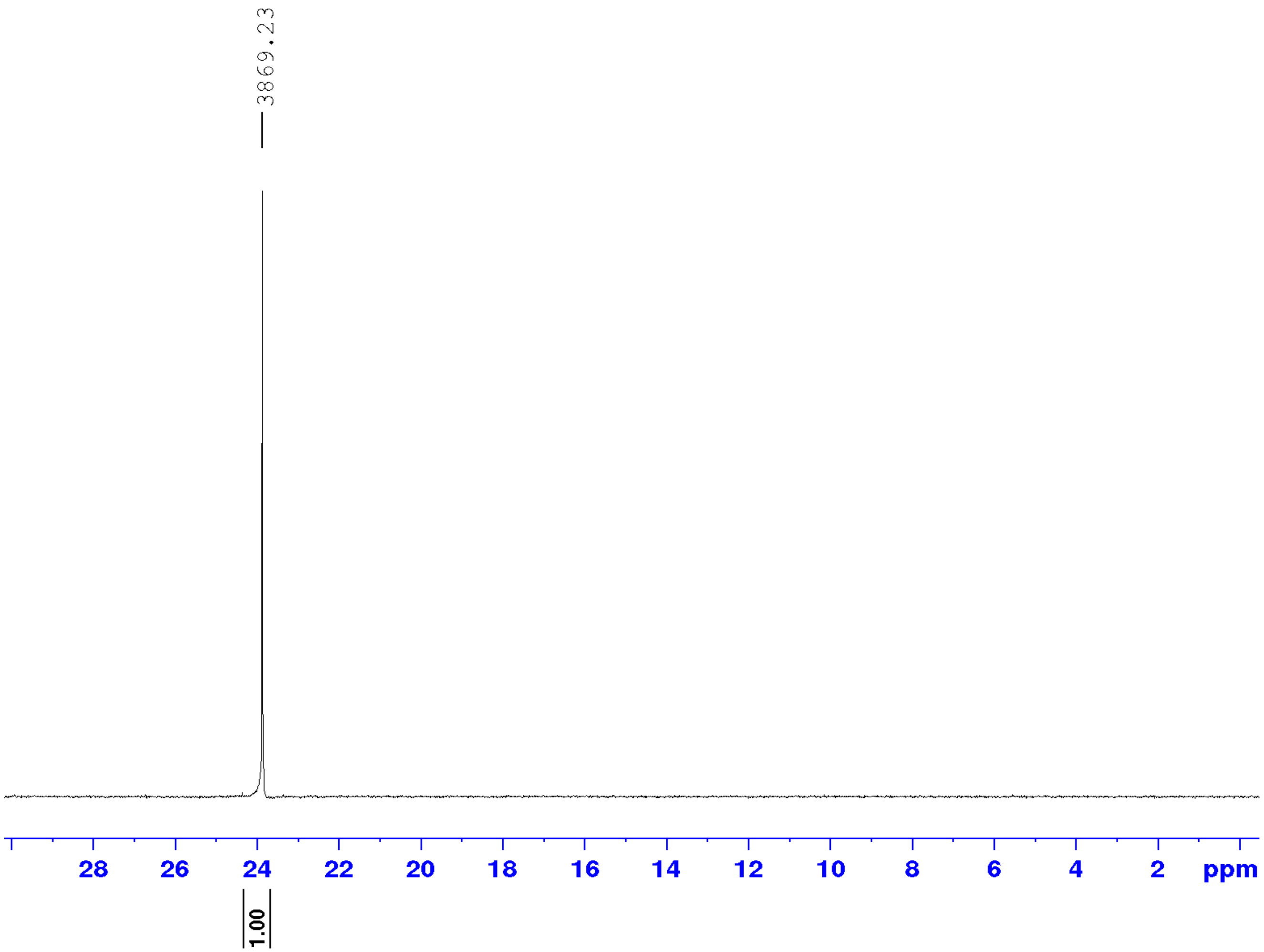

^{1}H NMR of (*R*)-diisopropyl 1-azido-3-methylbutylphosphonate (600.25 MHz, $CDCl_3$) [(*R*)-74]:

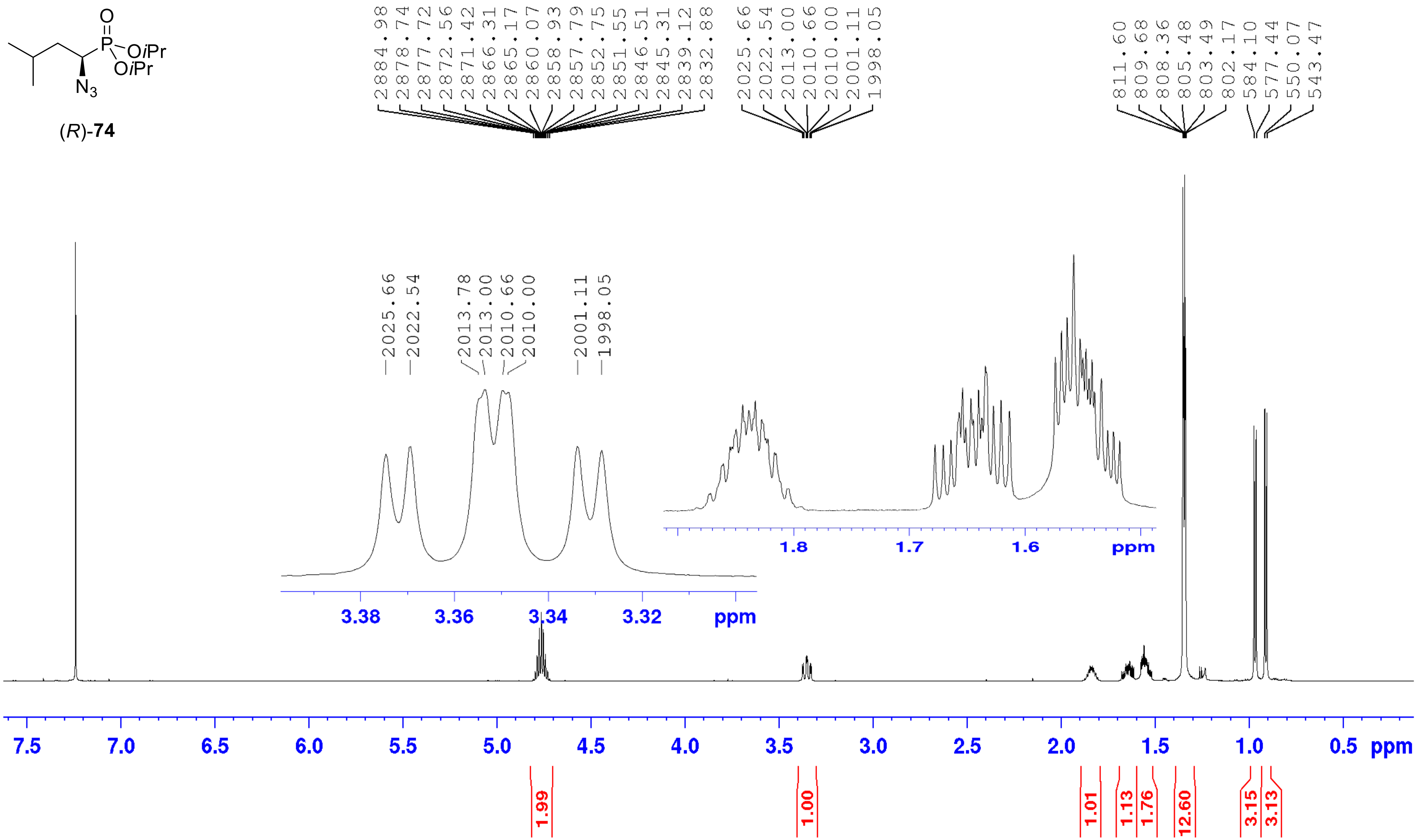

^{13}C NMR of (*R*)-diisopropyl 1-azido-3-methylbutylphosphonate (150.93 MHz, $CDCl_3$) [(*R*)-74]:

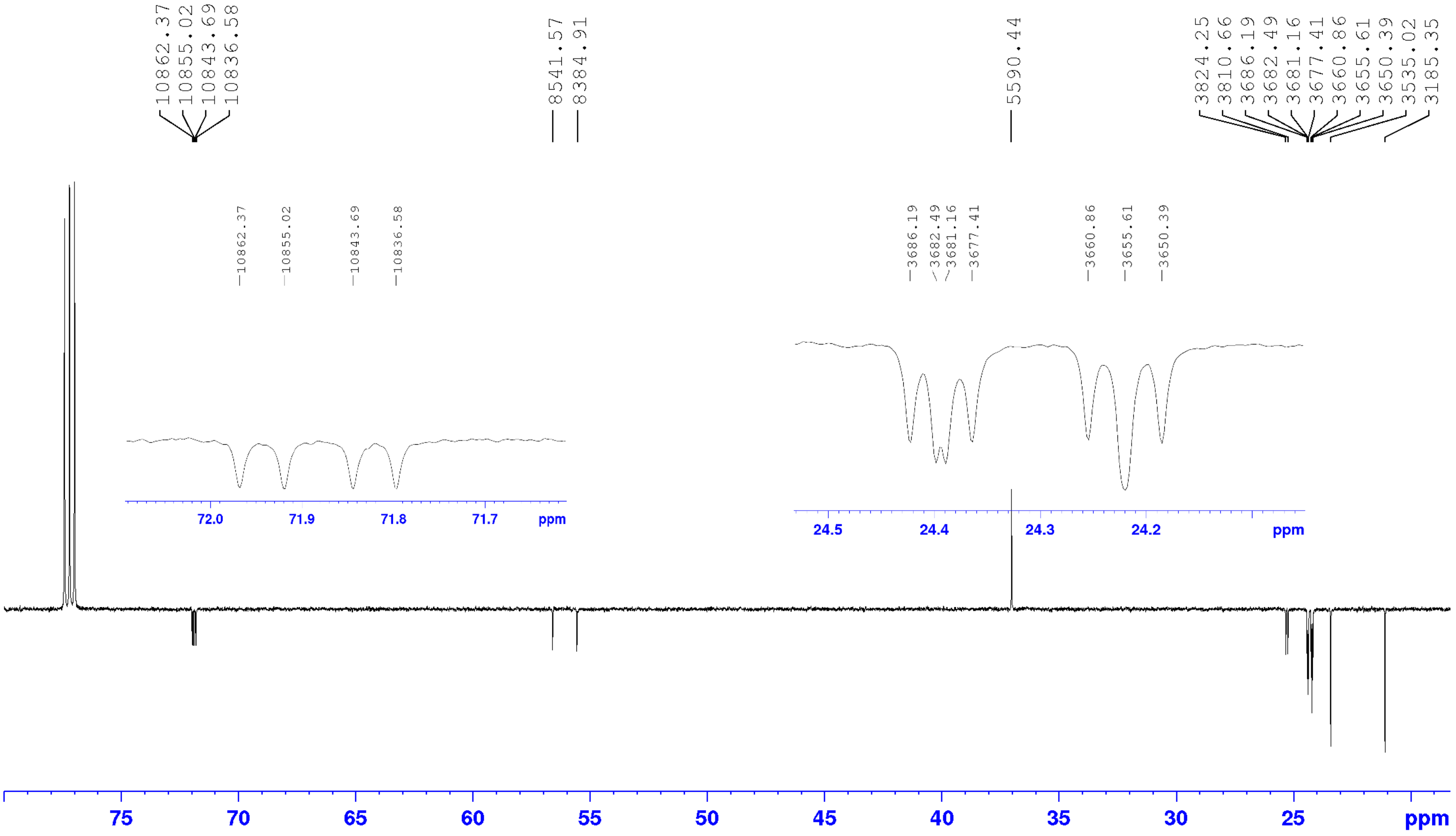

^{31}P NMR of (*R*)-diisopropyl 1-azido-3-methylbutylphosphonate (242.99 MHz, $CDCl_3$) [(*R*)-74]:

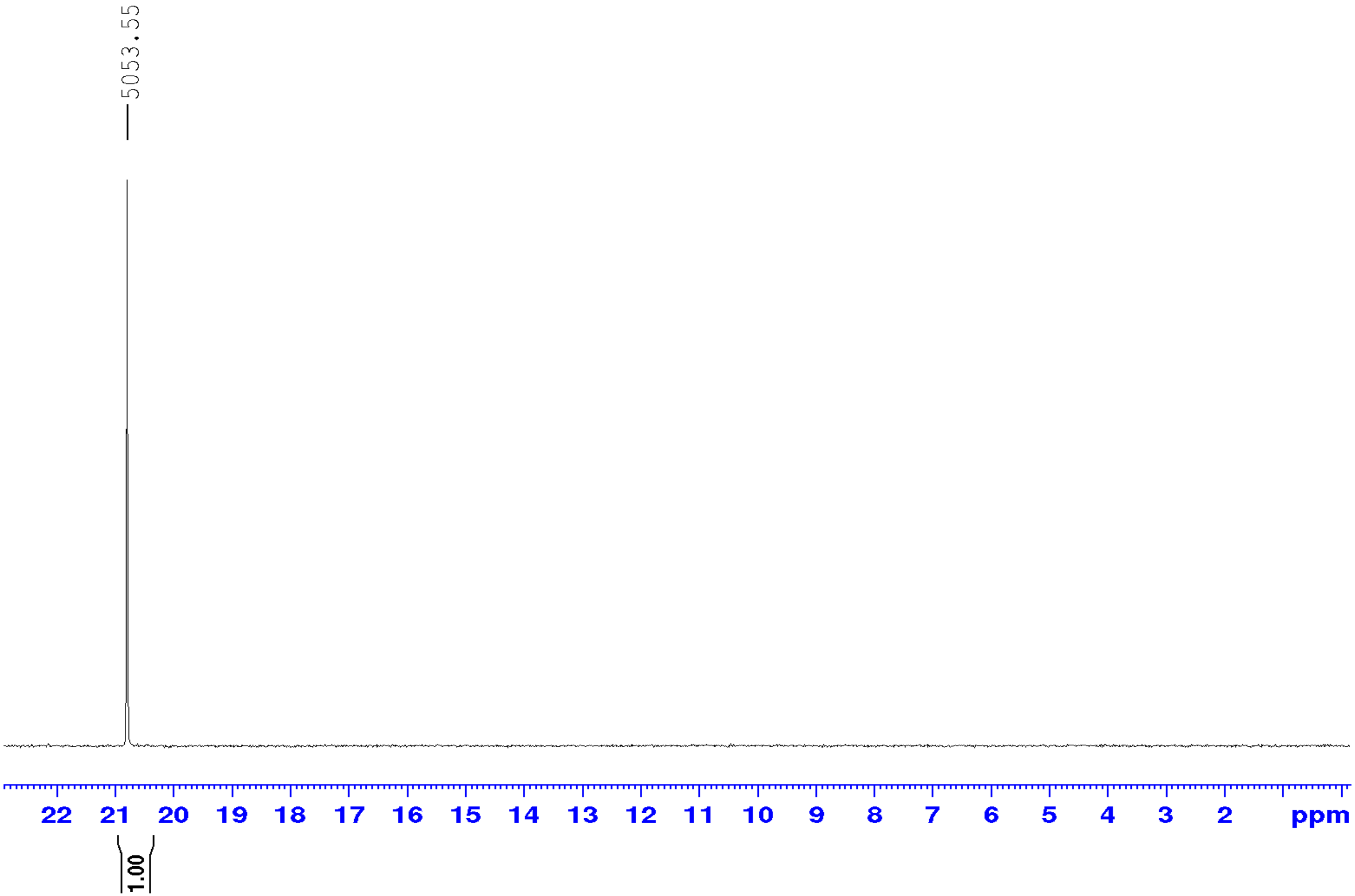

^{1}H NMR of (*R*)-1-amino-3-methylbutylphosphonic acid, (*R*)-phosphavaline (400.27 MHz, D_2O) [(*R*)-61]:

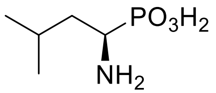

(*R*)-**61**

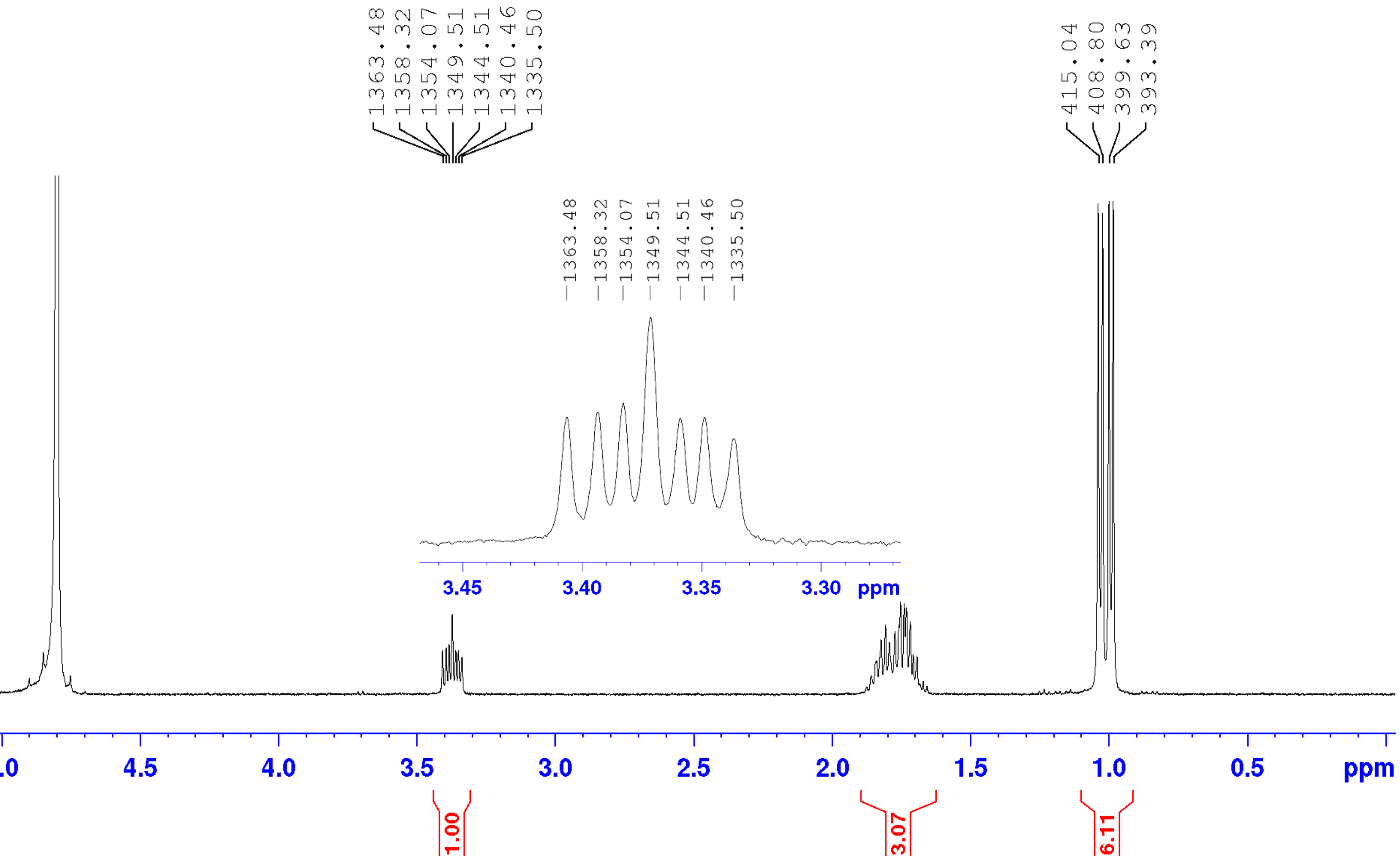

^{13}C NMR of (*R*)-1-amino-3-methylbutylphosphonic acid, (*R*)-phosphavaline (100.65 MHz, D_2O) [(*R*)-61]:

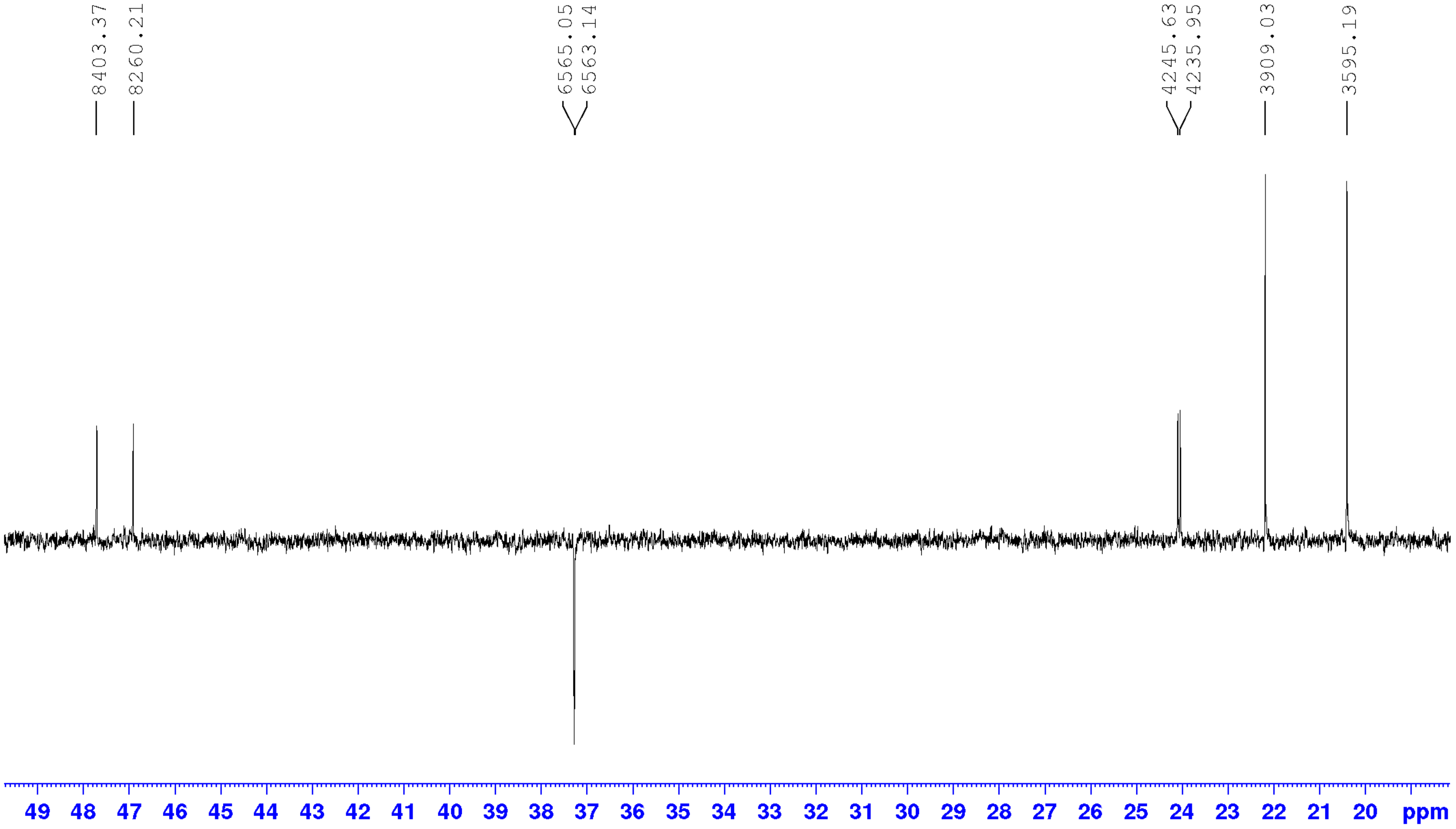

^{31}P NMR of (*R*)-1-amino-3-methylbutylphosphonic acid, (*R*)-phosphaleucine (162.03 MHz, D_2O) [(*R*)-61]:

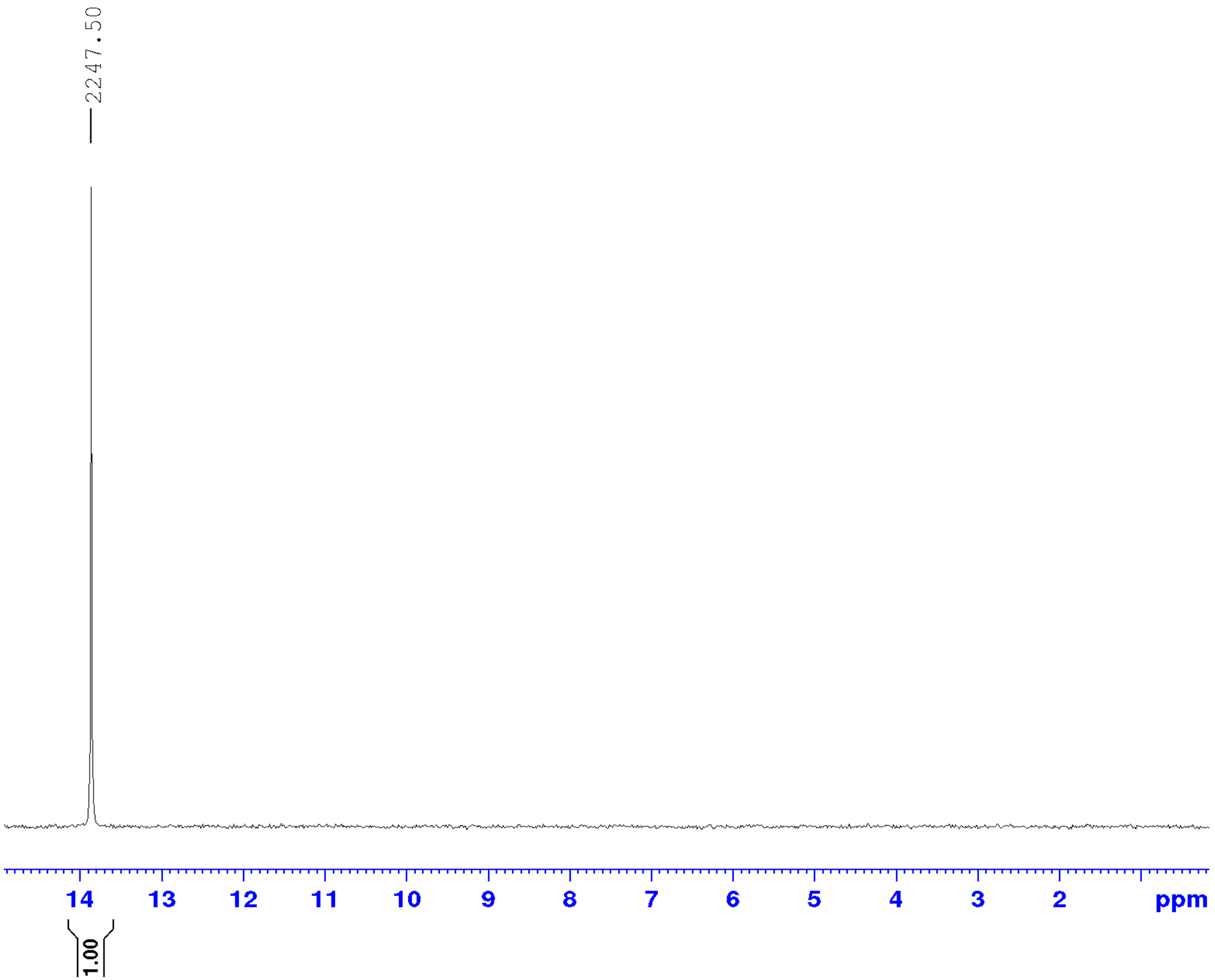

^{31}P NMR of (*S*)-Diisopropyl 1-oxo-2-methylbutylphosphonate (400.27 MHz, $CDCl_3$) [(*S*)-11]:

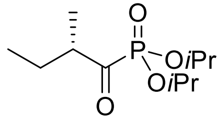

(*S*)-**11**

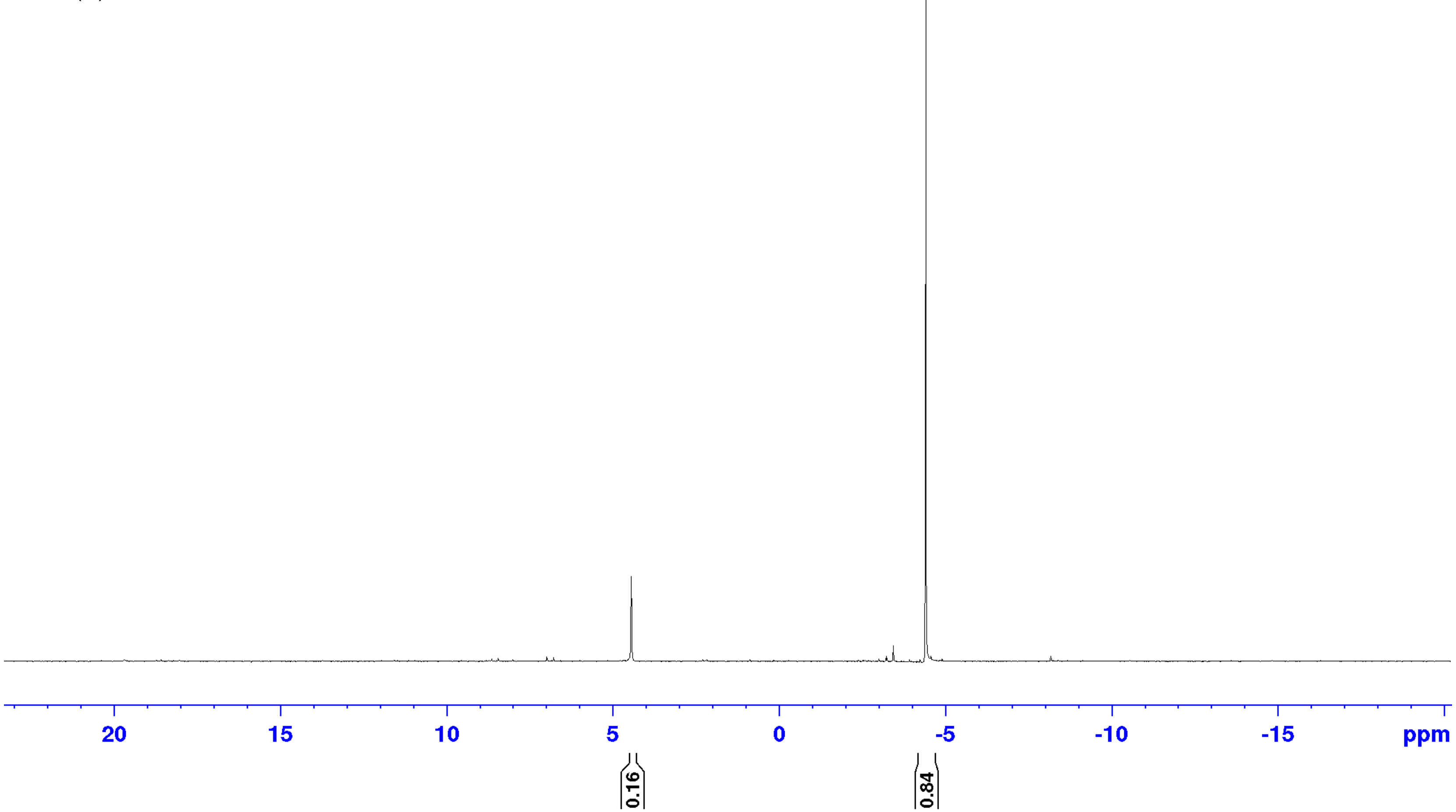

^{1}H NMR of (1*S*,2*S*)-Diisopropyl 1-hydroxy-2-methylbutylphosphonate (500.32 MHz, $CDCl_3$) [(1*S*,2*S*)-28]:

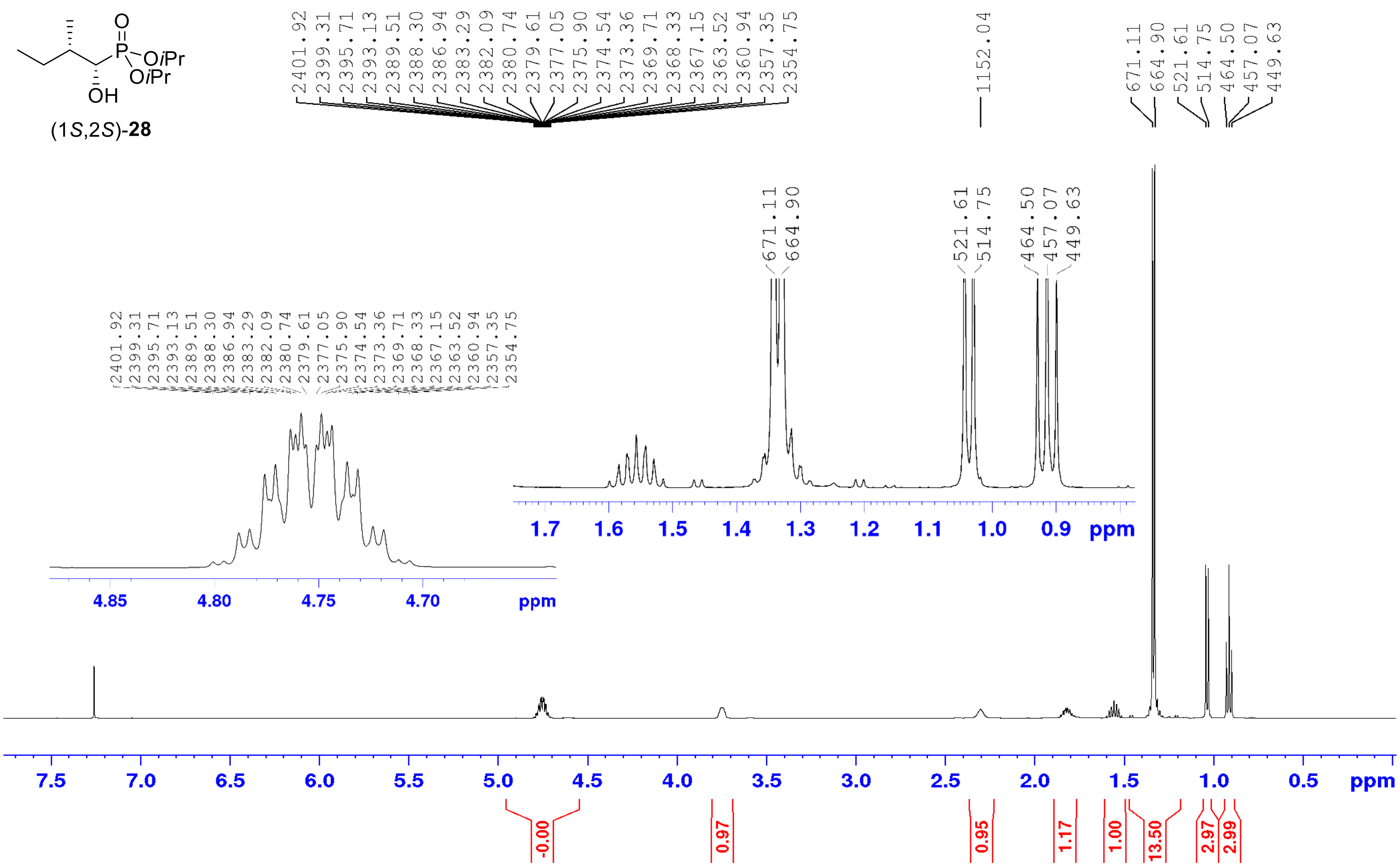

^{13}C NMR of (1*S*,2*S*)-Diisopropyl 1-hydroxy-2-methylbutylphosphonate (125.81 MHz, $CDCl_3$) [(1*S*,2*S*)-28]:

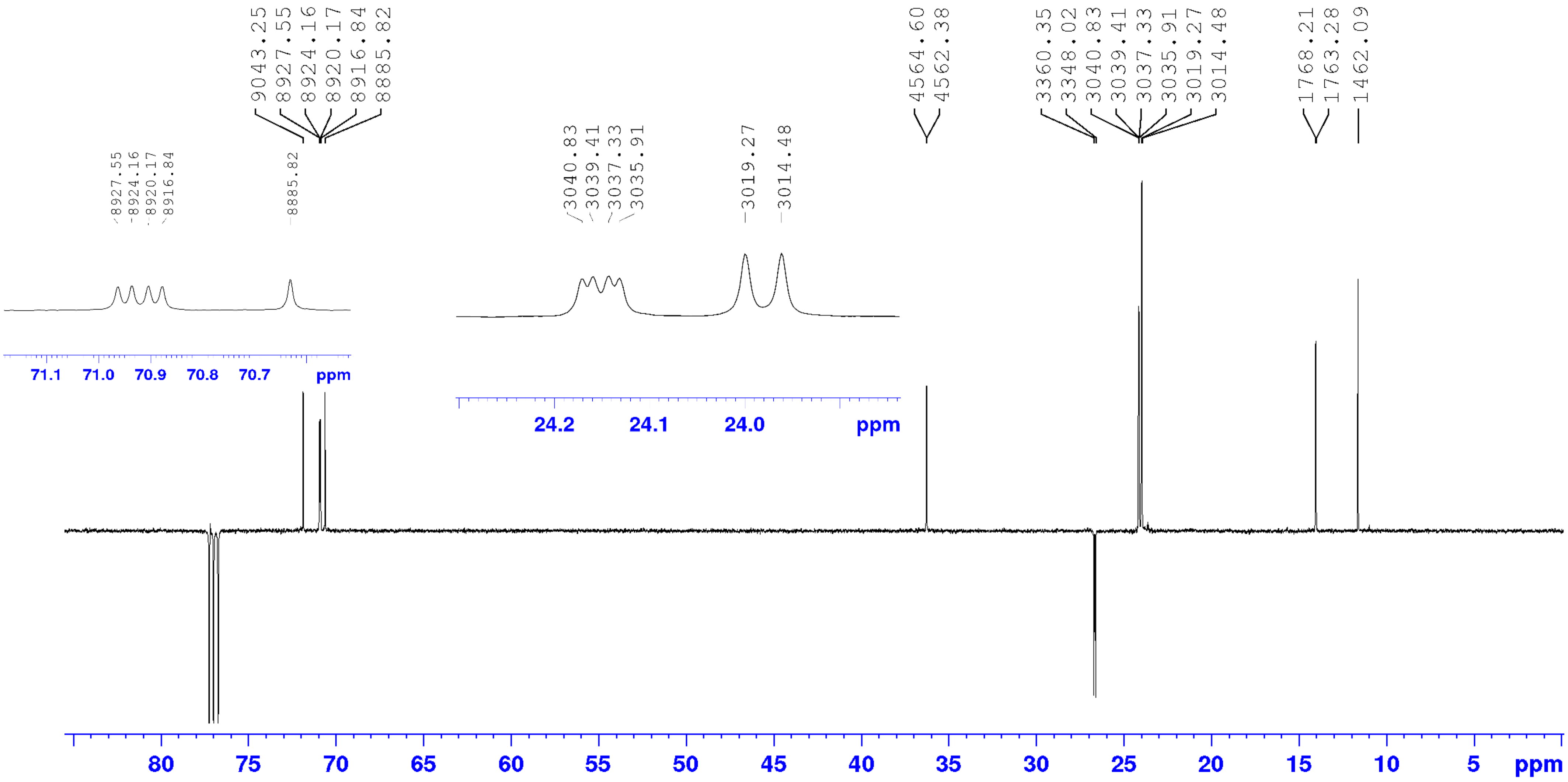

^{31}P NMR of (1*S*,2*S*)-Diisopropyl 1-hydroxy-2-methylbutylphosphonate (162.03 MHz, $CDCl_3$) [(1*S*,2*S*)-28]:

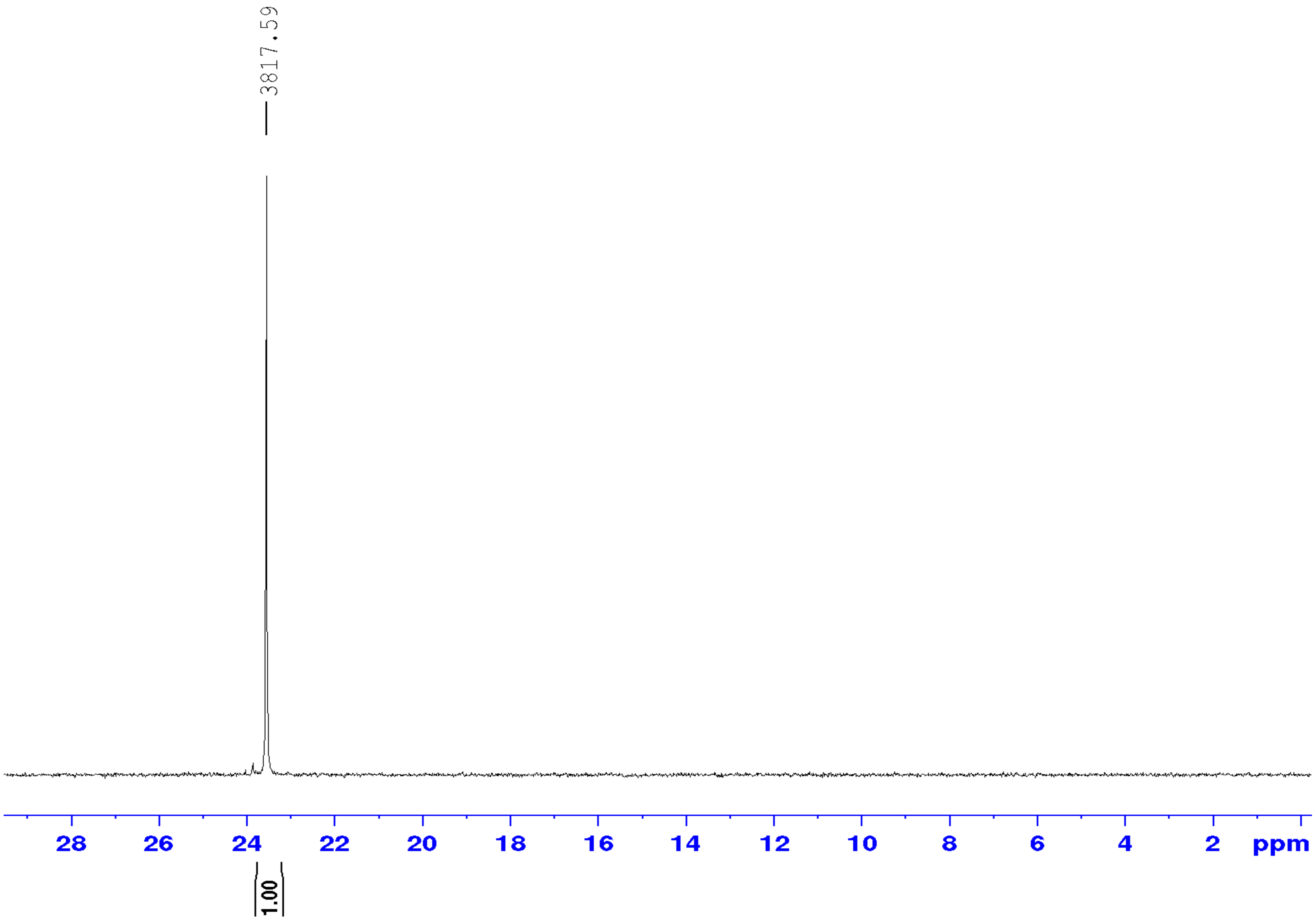

^{1}H NMR of (1*R*,2*S*)-Diisopropyl 1-azido-2-methylbutylphosphonate (700.40 MHz, $CDCl_3$) [(1*R*,2*S*)-75]:

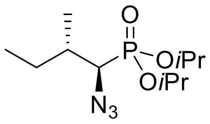

(1*R*,2*S*)-**75**

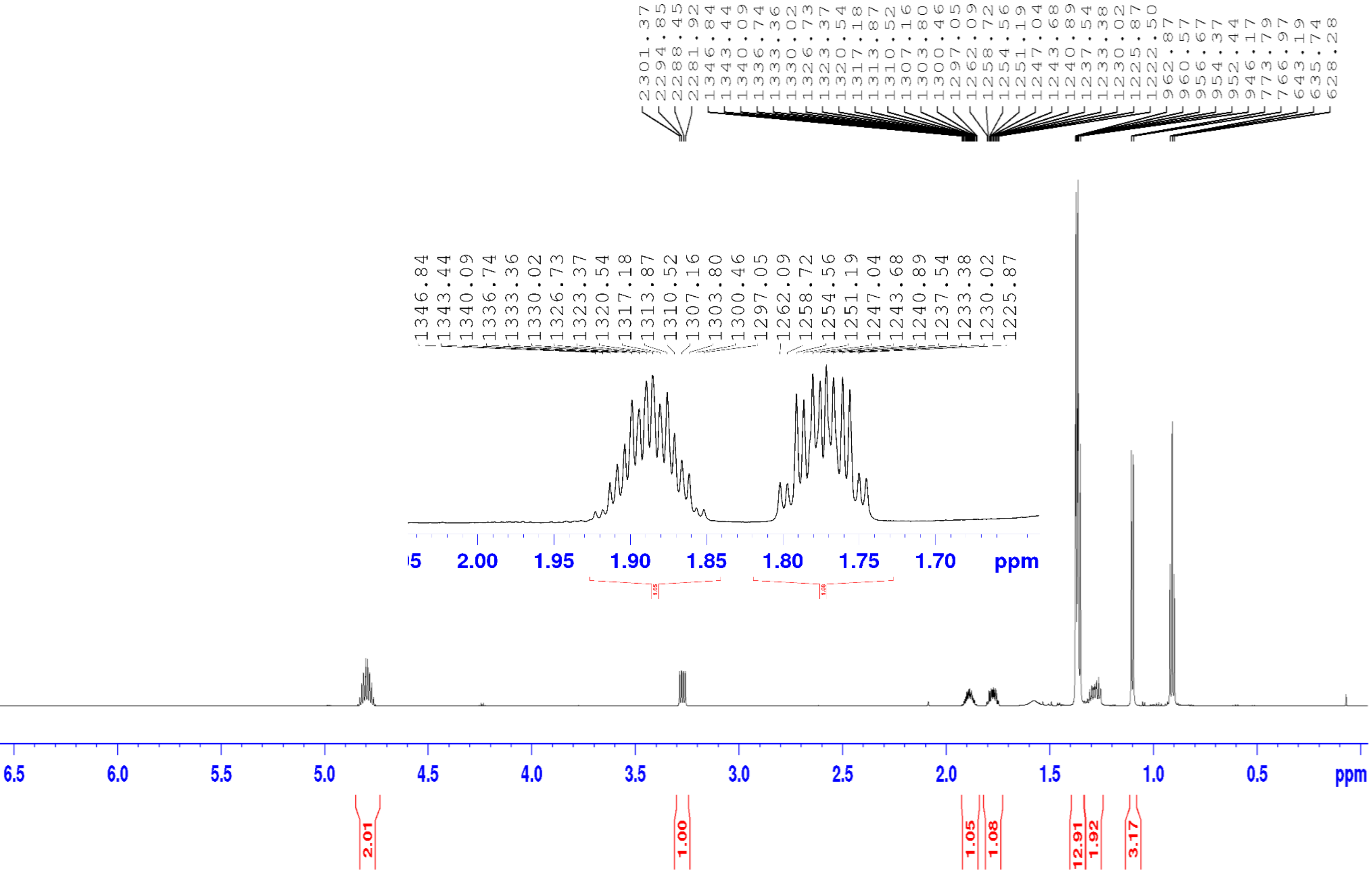

^{31}P NMR of (1*R*,2*S*)-Diisopropyl 1-azido-2-methylbutylphosphonate (161.98 MHz, $CDCl_3$) [(1*R*,2*S*)-75]:

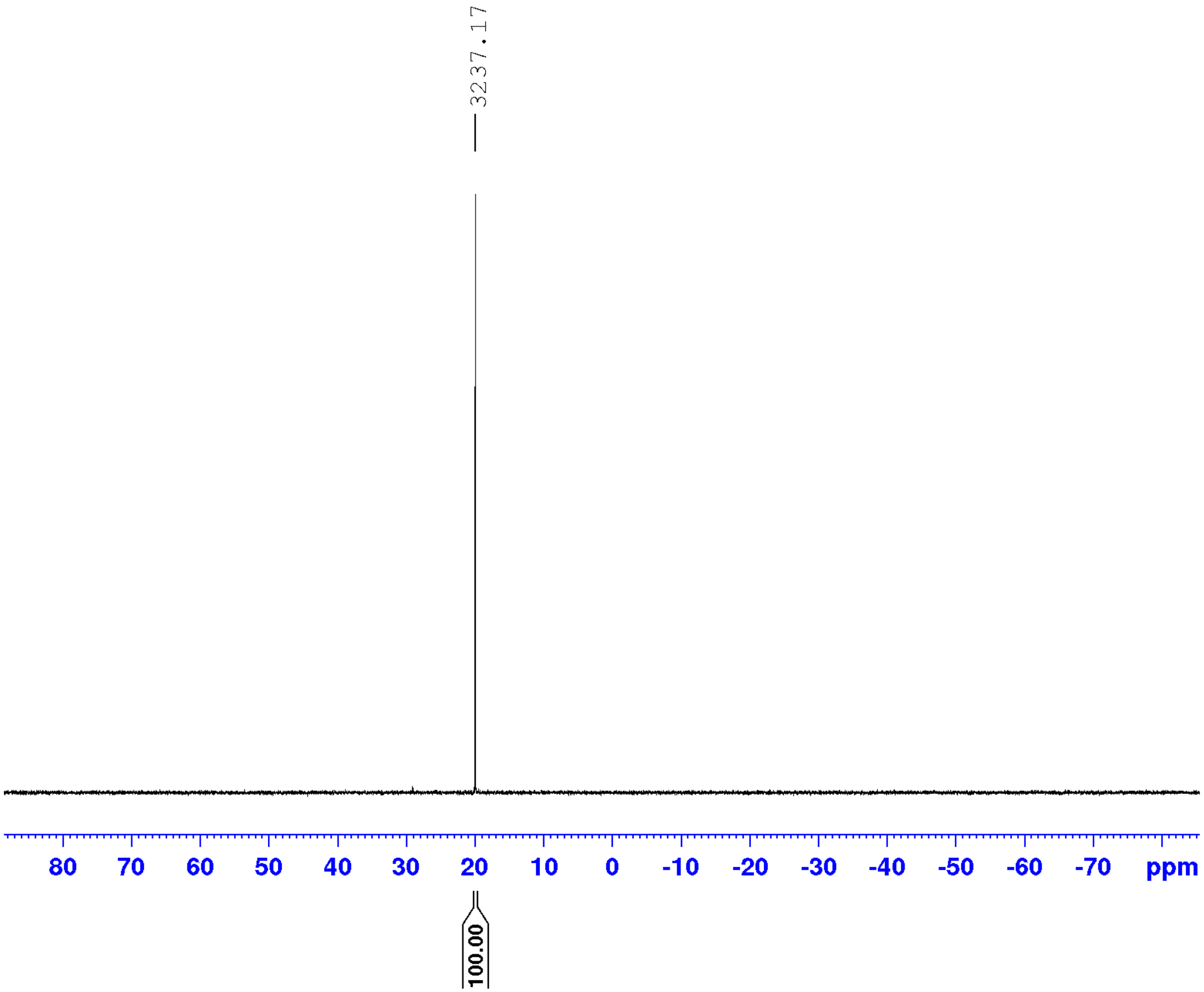

^{13}C NMR of (1*R*,2*S*)-Diisopropyl 1-azido-2-methylbutylphosphonate (176.12 MHz, CDCl$_3$) [(1*R*,2*S*)-75]:

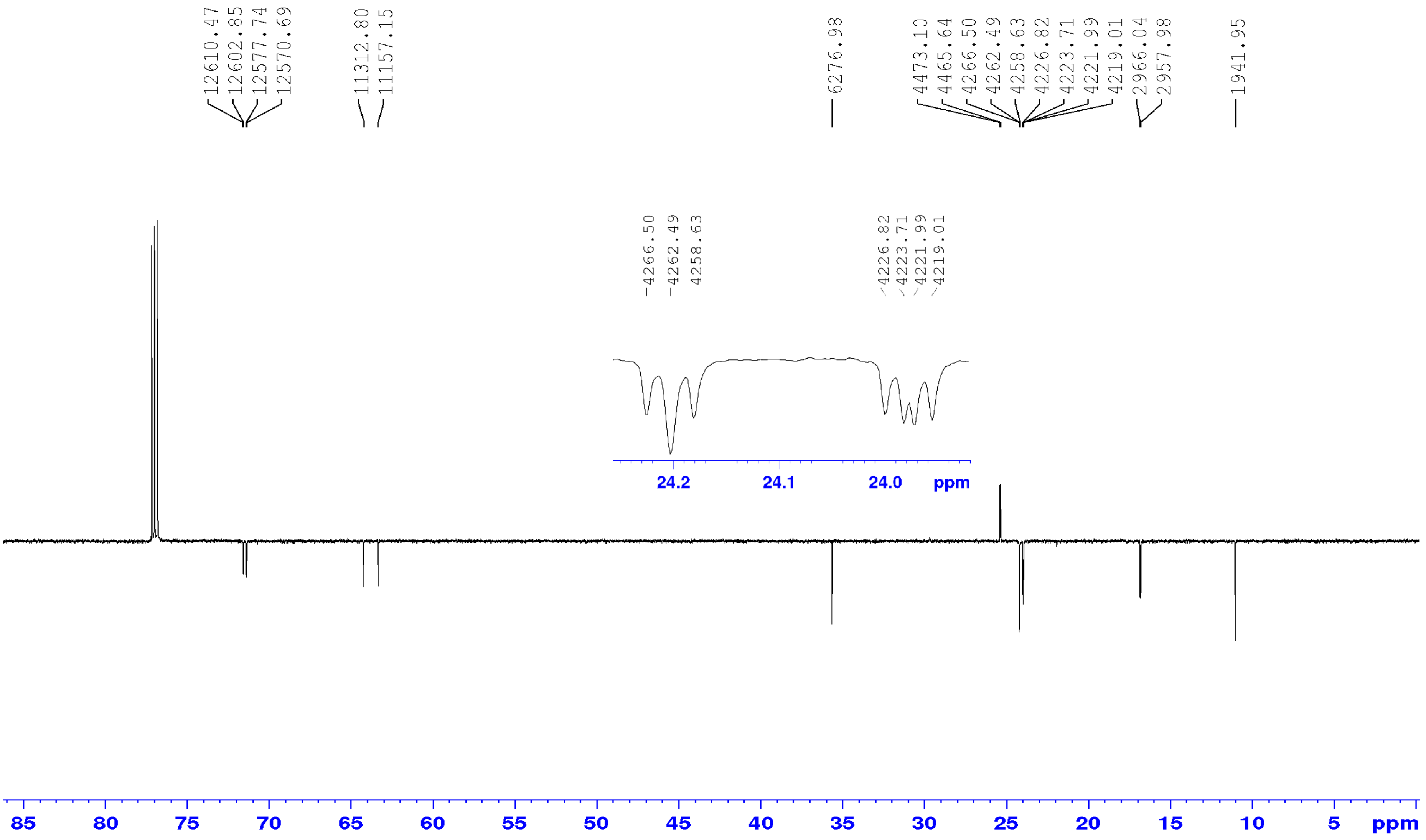

^{1}H NMR of (1*R*,2*S*)-1-amino-2-methylbutylphosphonic acid, (1*R*,2*S*)-phosphaisoleucine (700.40 MHz, D_2O) [(1*R*,2*S*)-62]:

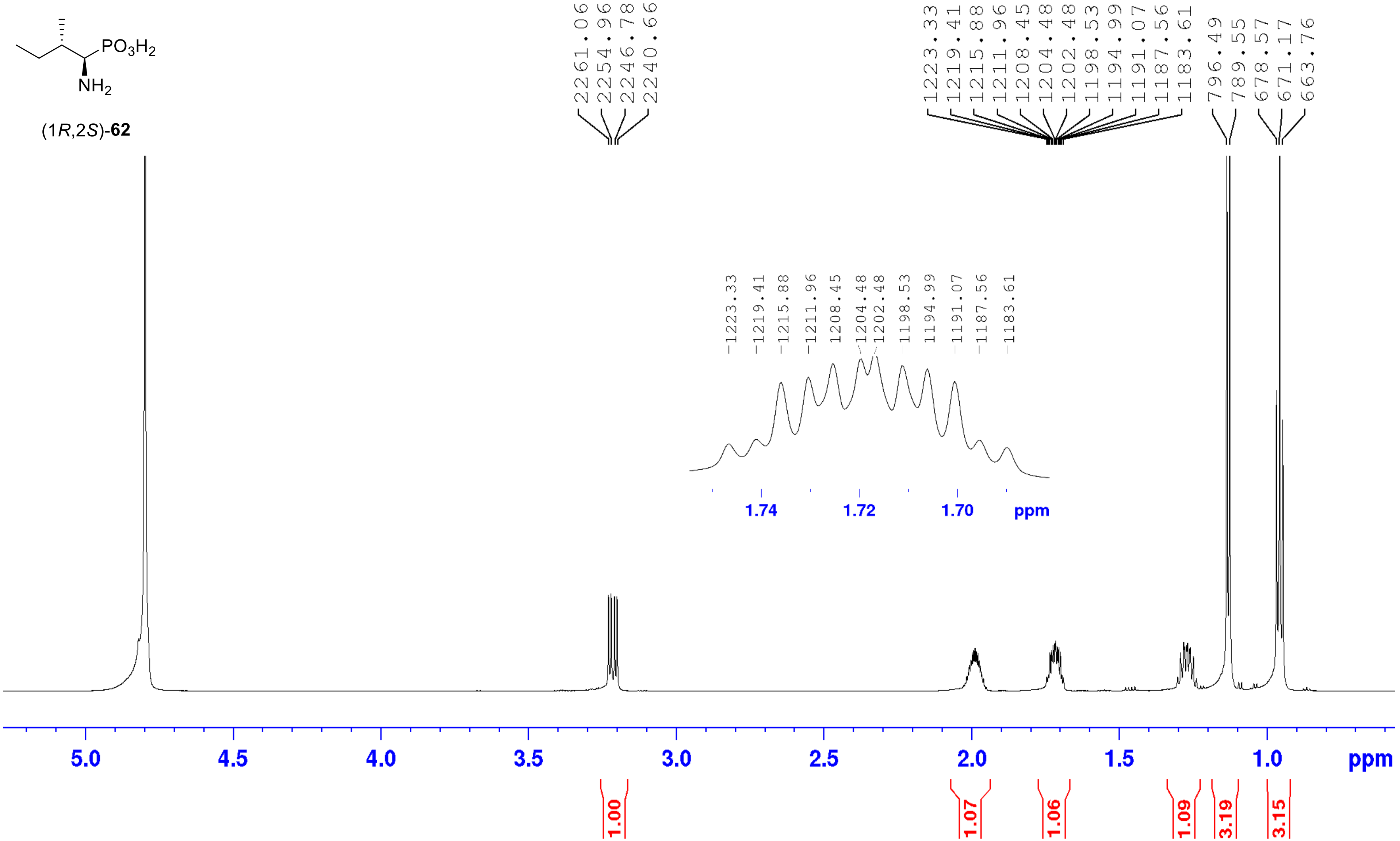

^{31}P NMR of (1*R*,2*S*)-1-amino-2-methylbutylphosphonic acid, (1*R*,2*S*)-phosphaisoleucine (161.98 MHz, D_2O) [(1*R*,2*S*)-62]

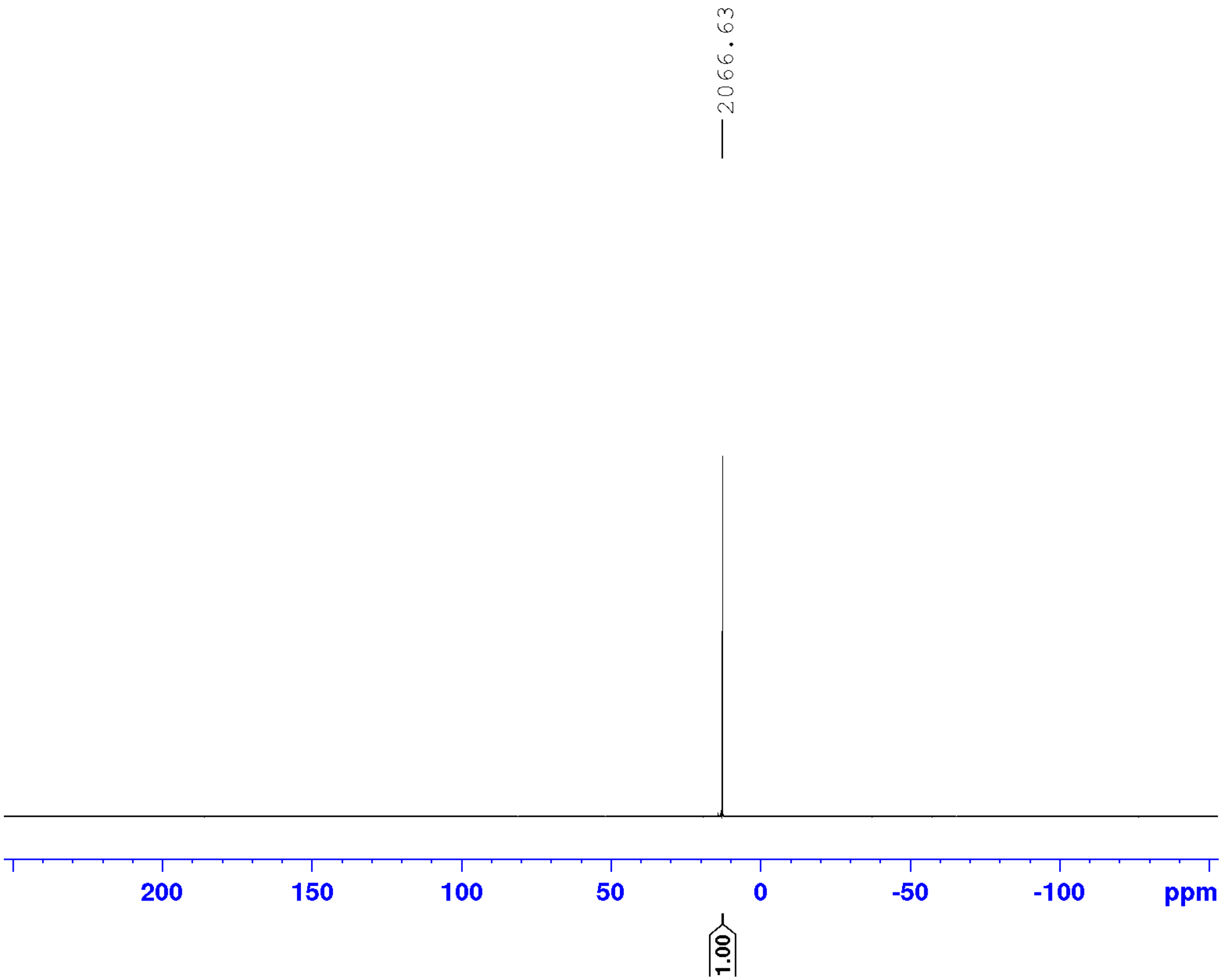

^{13}C NMR of (1*R*,2*S*)-1-amino-2-methylbutylphosphonic acid, (1*R*,2*S*)-phosphaisoleucine (176.12 MHz, D_2O) [(1*R*,2*S*)-X]

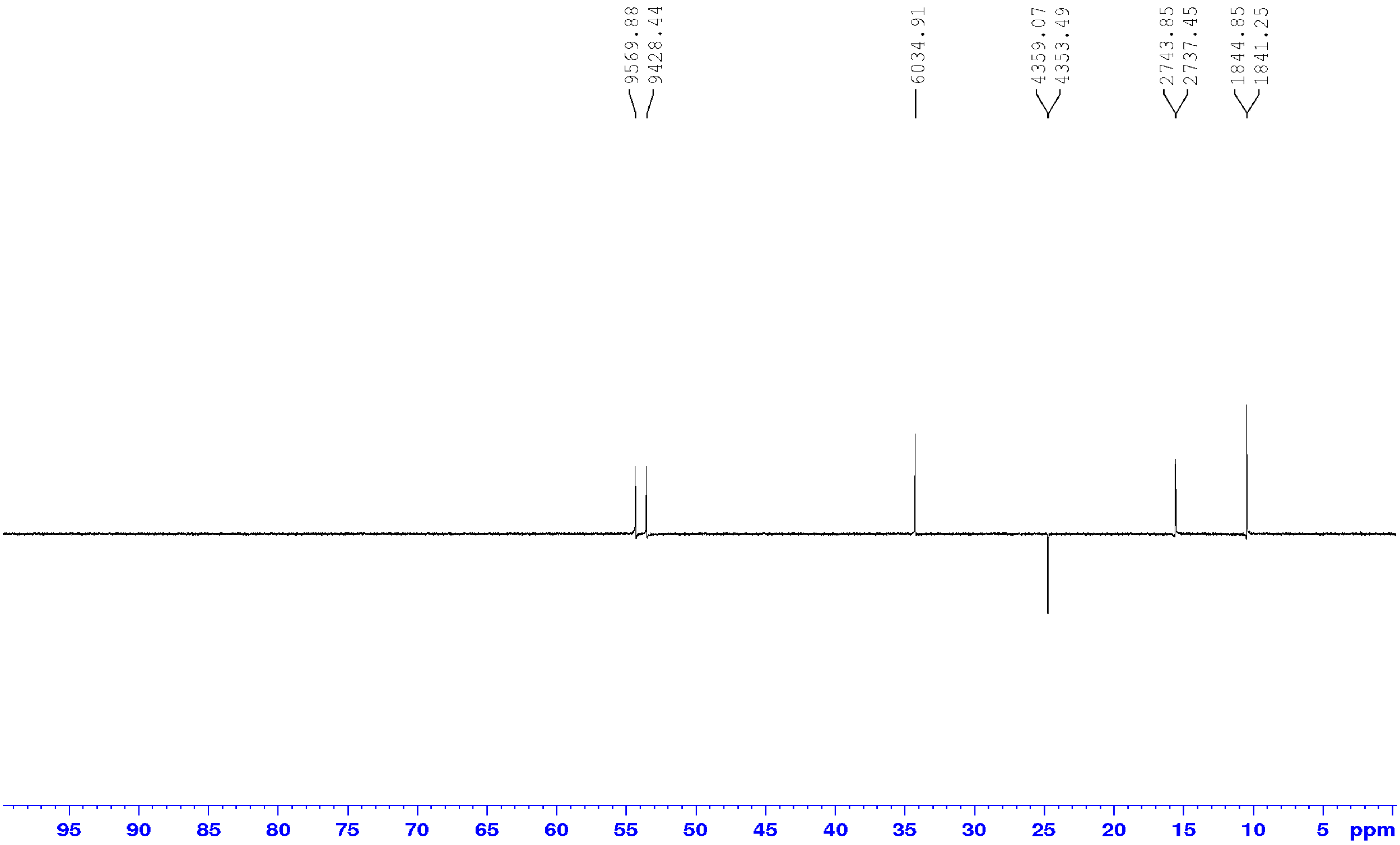

^{1}H NMR of diisopropyl pent-4-enoylphosphonate (400.27 MHz, $CDCl_3$) (12):

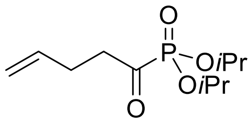

12

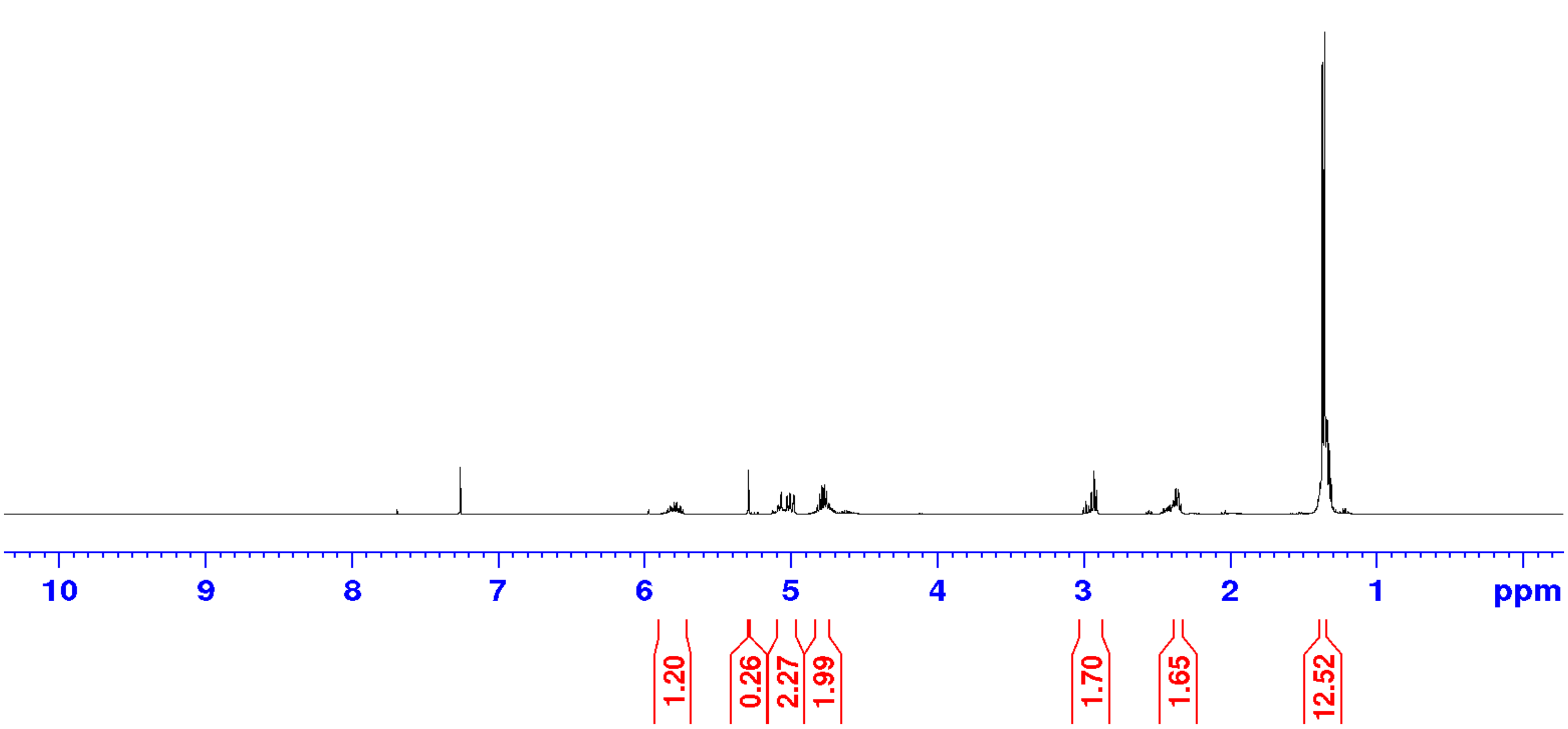

^{31}P NMR of diisopropyl pent-4-enoylphosphonate (162.03 MHz, $CDCl_3$) (12):

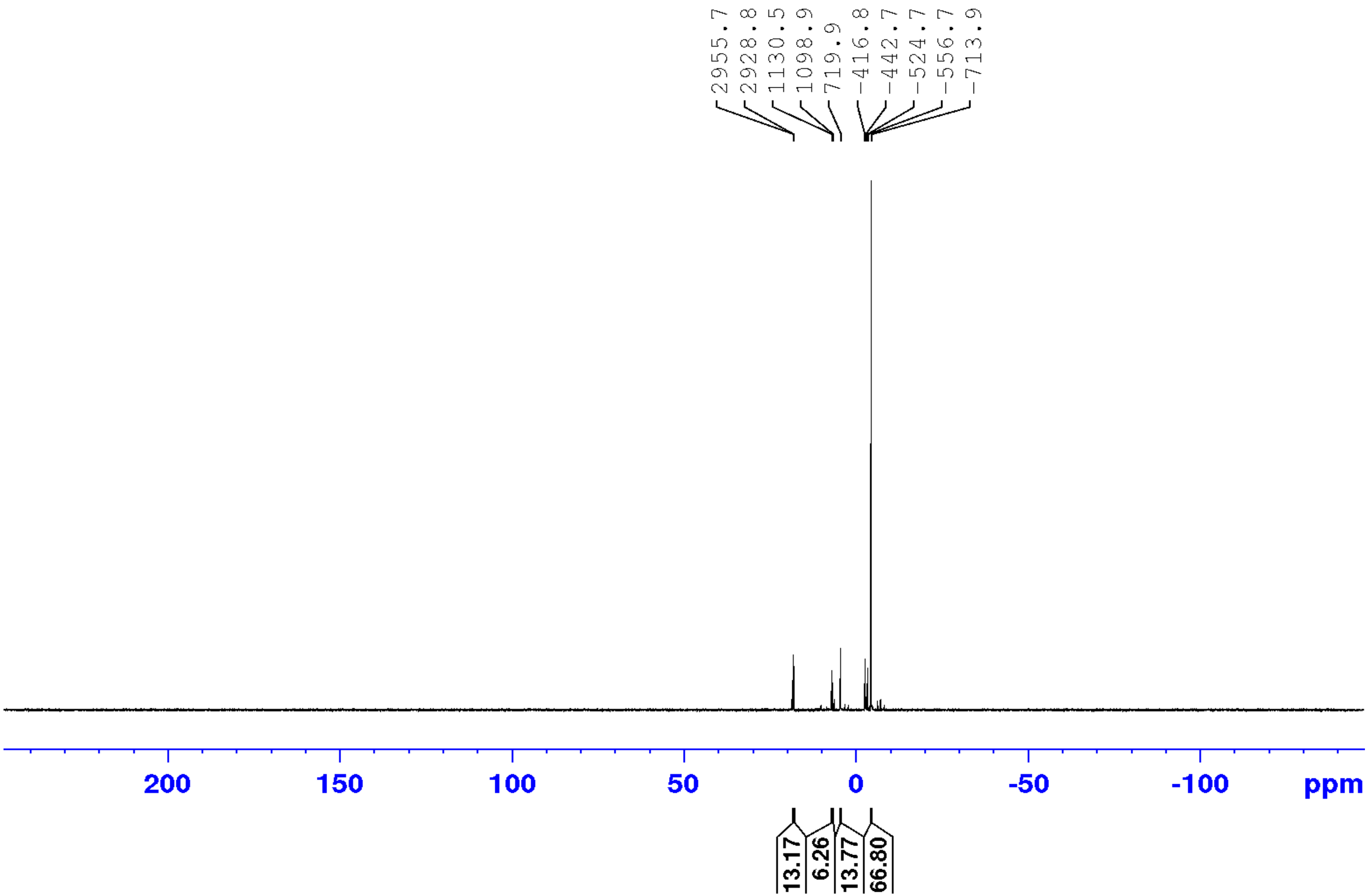

^{1}H NMR of (*S*)-diisopropyl 1-hydroxypent-4-en-1-yl-phosphonate (600.25 MHz, CDCl$_3$) [(*S*)-29]:

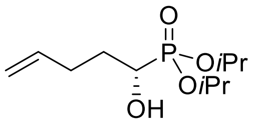

(S)-29

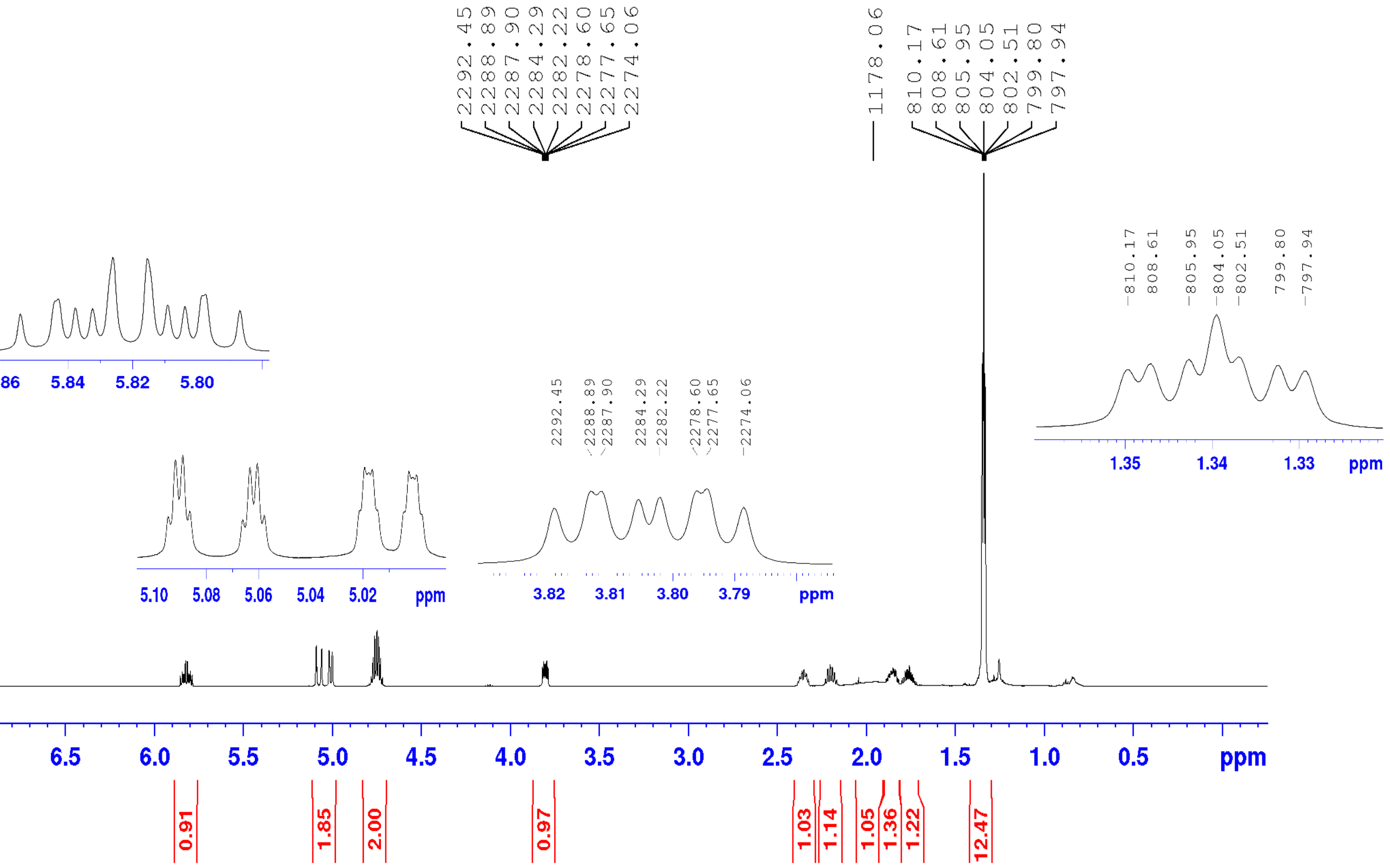

^{13}C NMR of (*S*)-diisopropyl 1-hydroxypent-4-en-1-yl-phosphonate (150.93 MHz, $CDCl_3$) [(*S*)-29]:

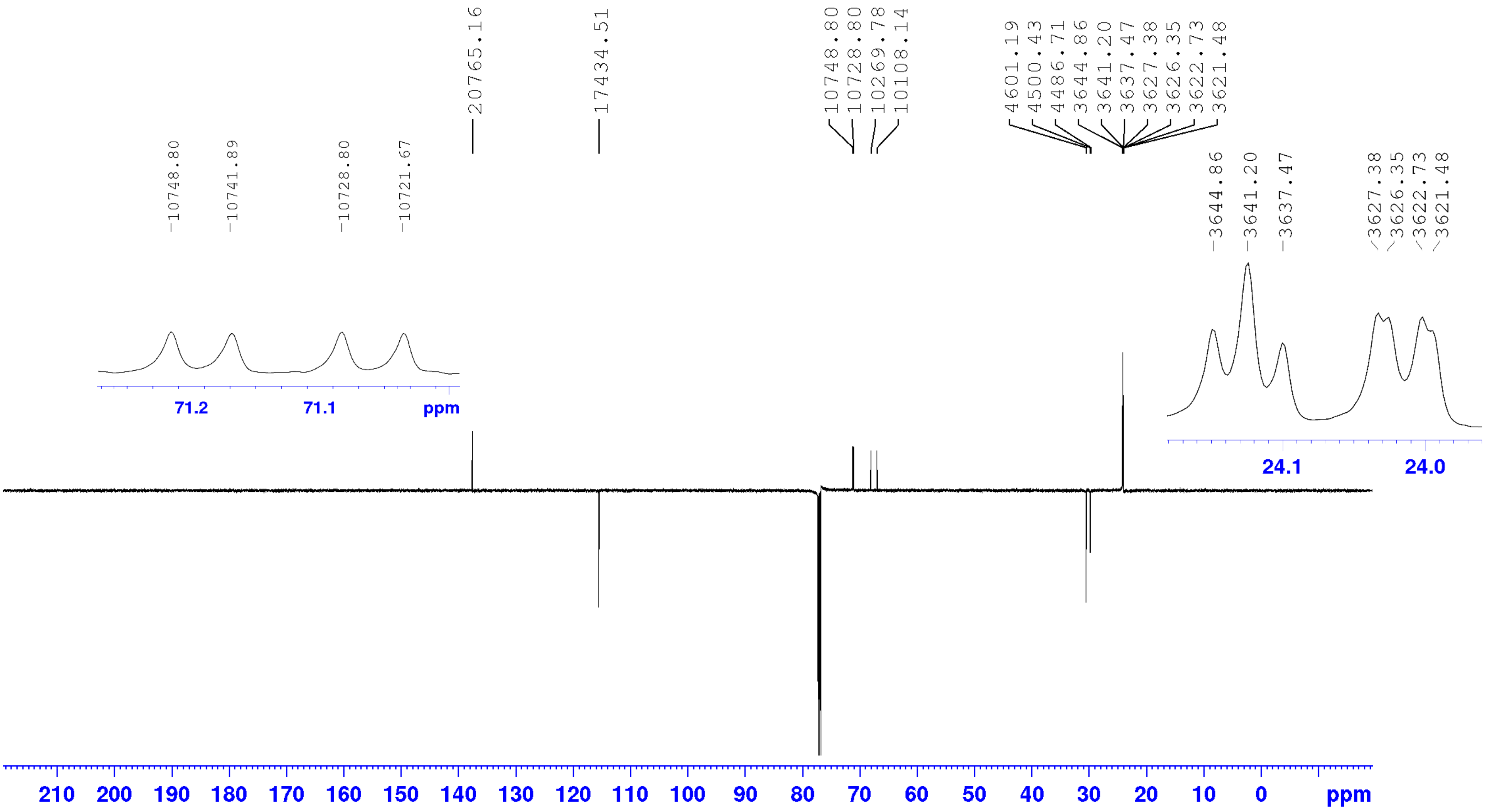

^{31}P NMR (*S*)-diisopropyl 1-hydroxypent-4-en-1-yl-phosphonate (242.99 MHz, $CDCl_3$) [(*S*)-29]:

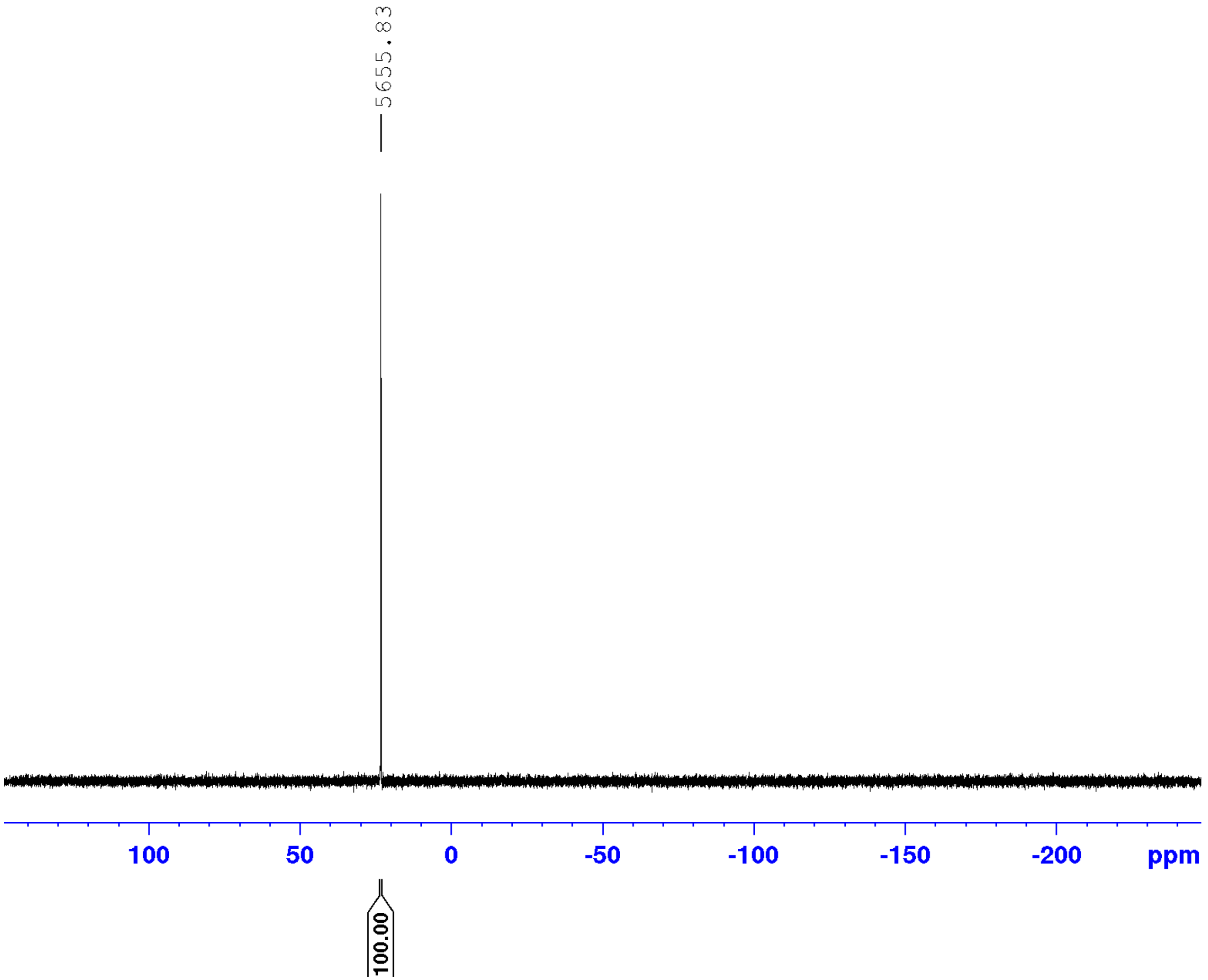

^{1}H NMR of (S)-diisopropyl-(1,5-dihydroxypentyl)phosphonate (600.25 MHz, $CDCl_3$) [(*S*)-76]:

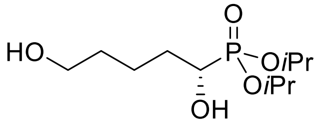

(*S*)-**76**

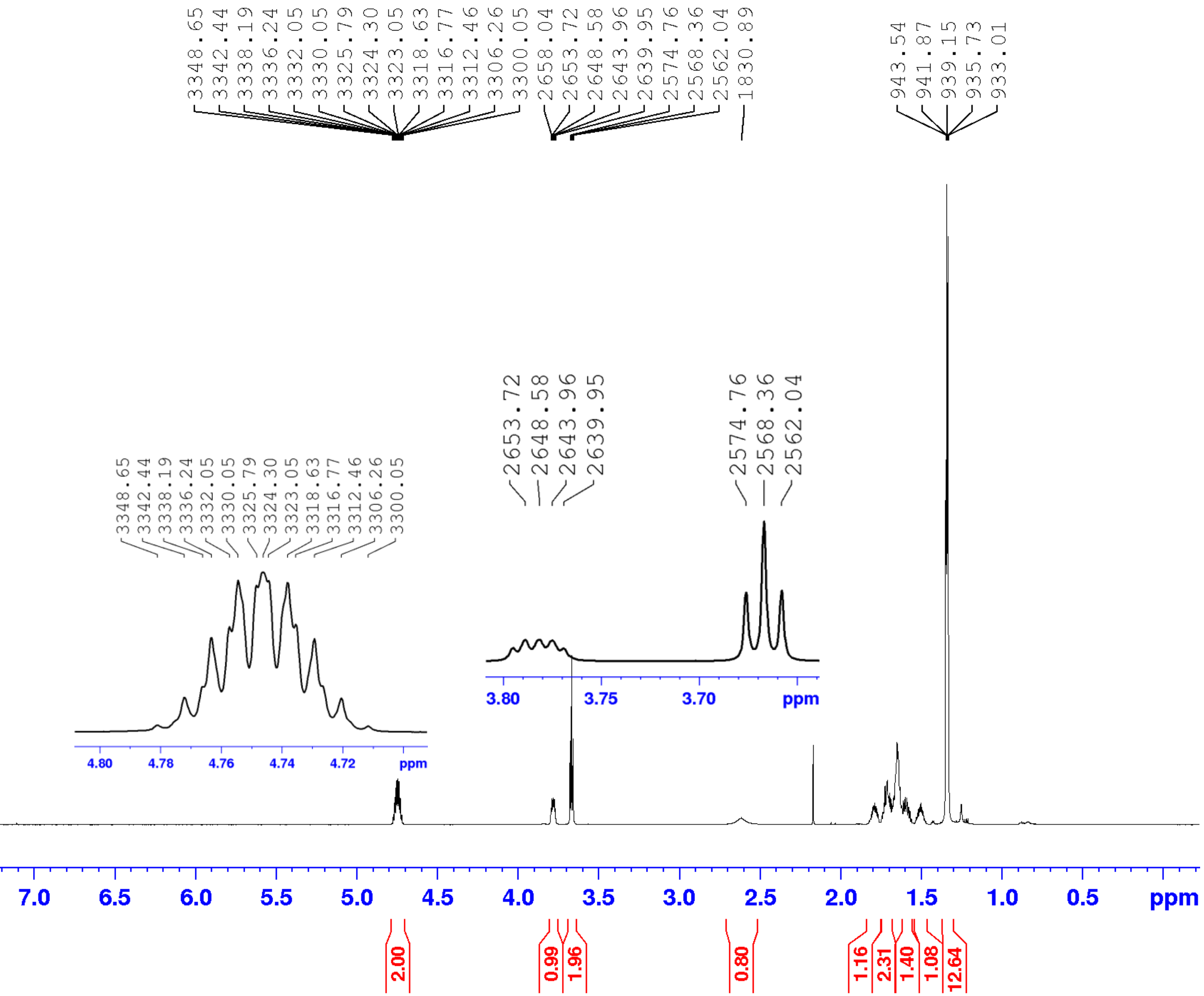

^{31}P NMR of (S)-diisopropyl-(1,5-dihydroxypentyl)phosphonate (162.03 MHz, $CDCl_3$) [(*S*)-76]:

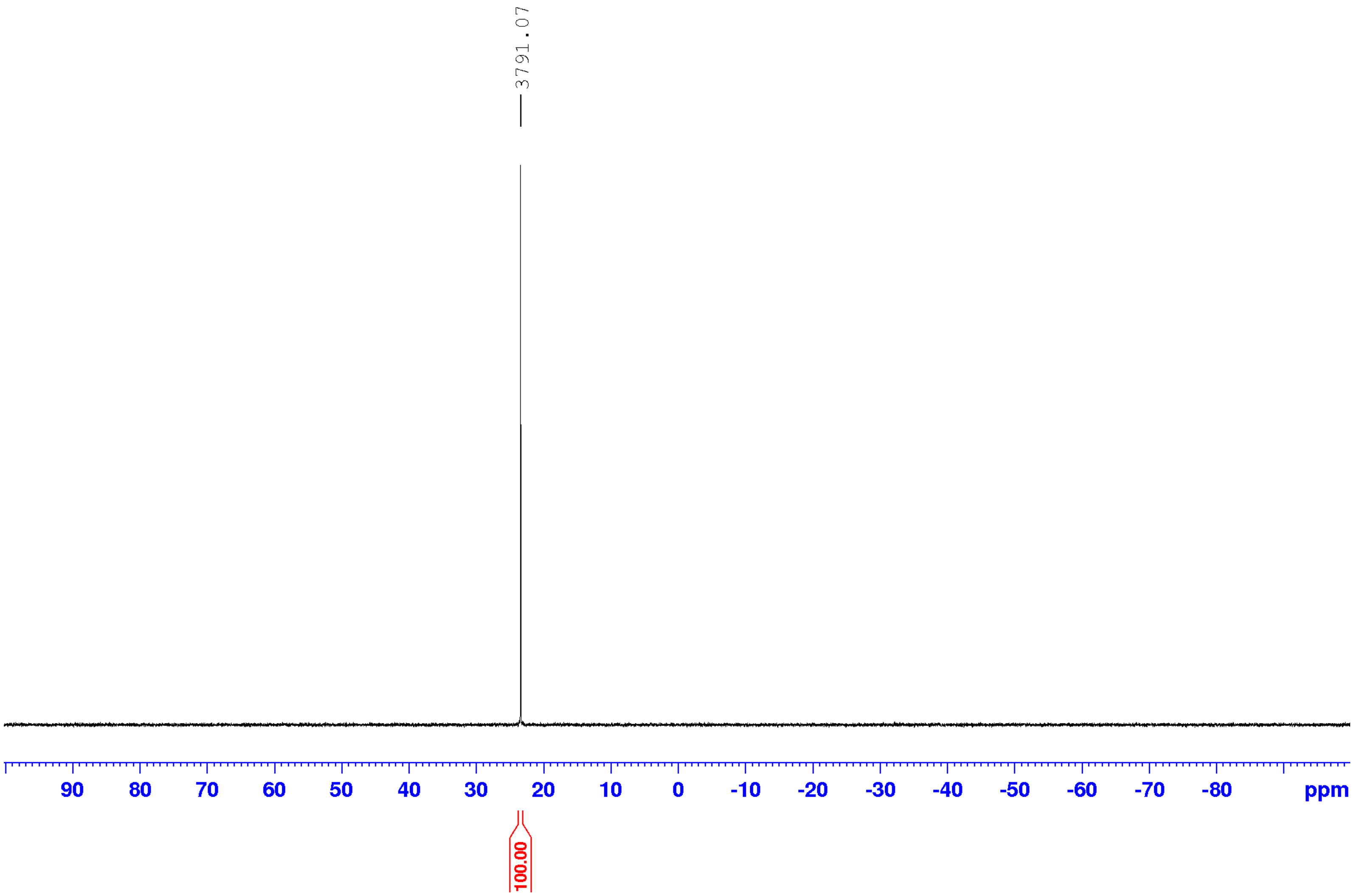

^{13}C NMR of (S)-diisopropyl-(1,5-dihydroxypentyl)phosphonate (150.93 MHz, $CDCl_3$) [(*S*)-76]:

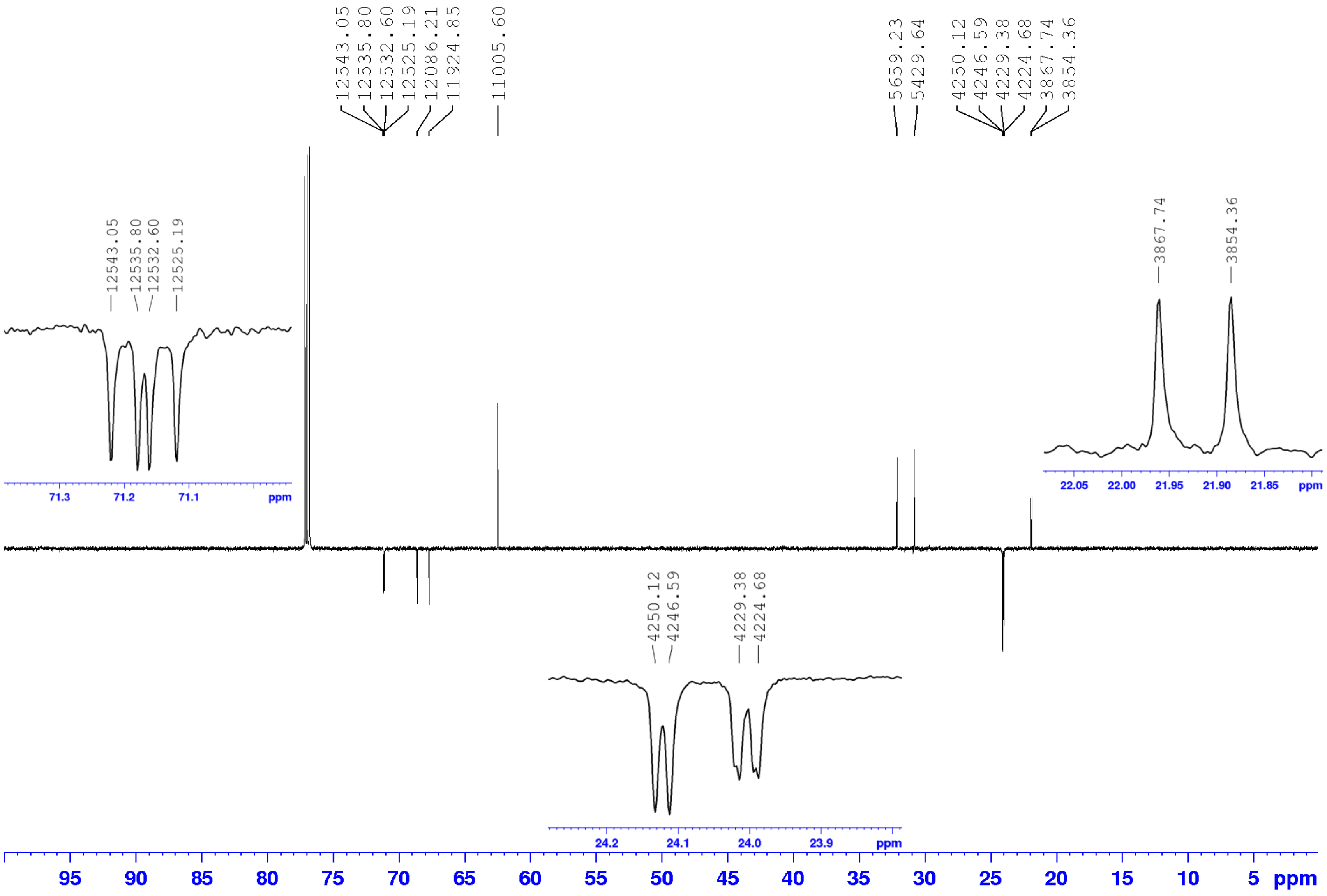

^{1}H NMR of (R)-diisopropyl-(1,5-diazidopentyl)phosphonate (600.25 MHz, $CDCl_3$) [(*R*)-77]:

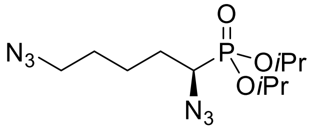

(*R*)-**77**

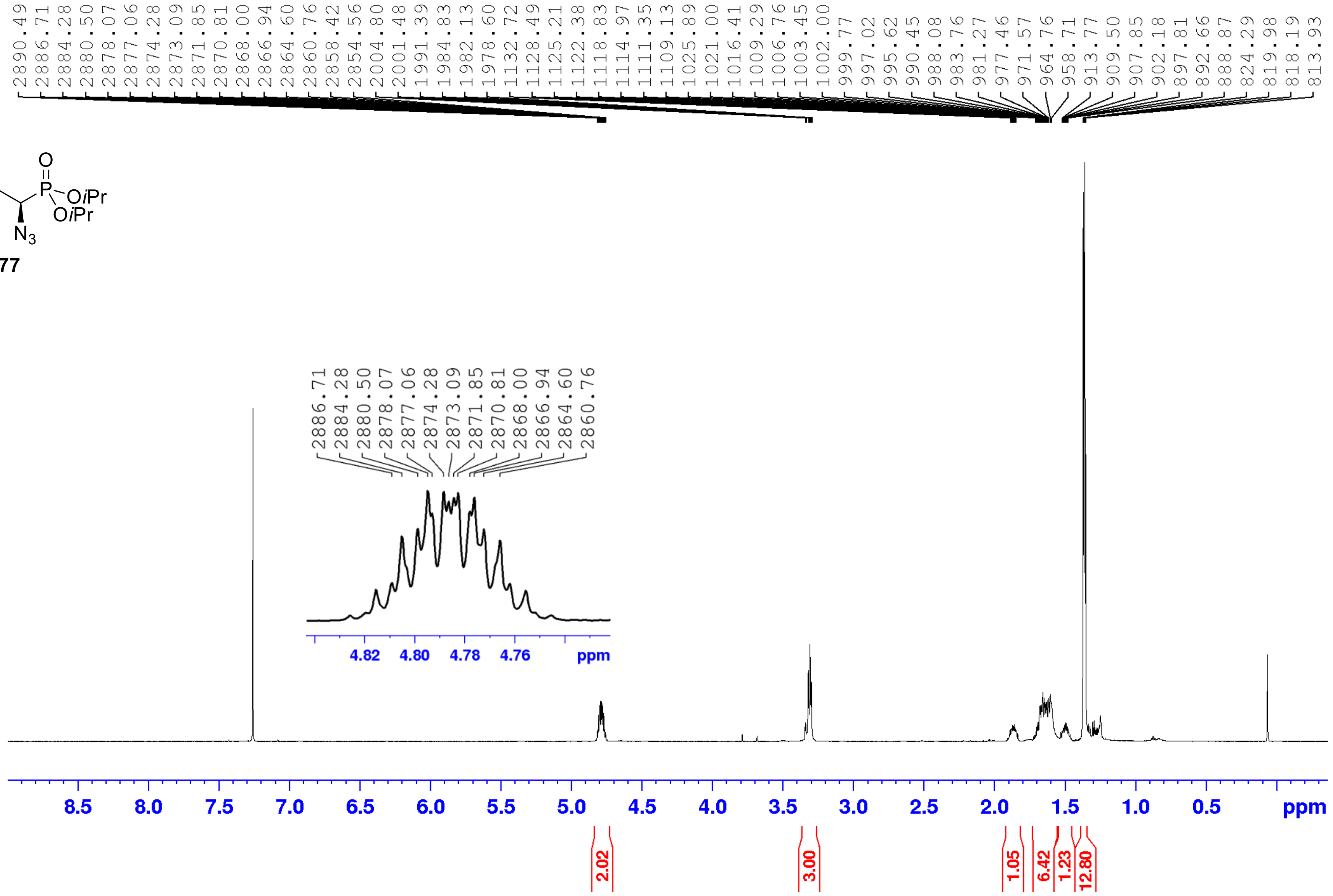

^{31}P NMR of (R)-diisopropyl-(1,5-diazidopentyl)phosphonate (162.03 MHz, $CDCl_3$) [(*R*)-77]:

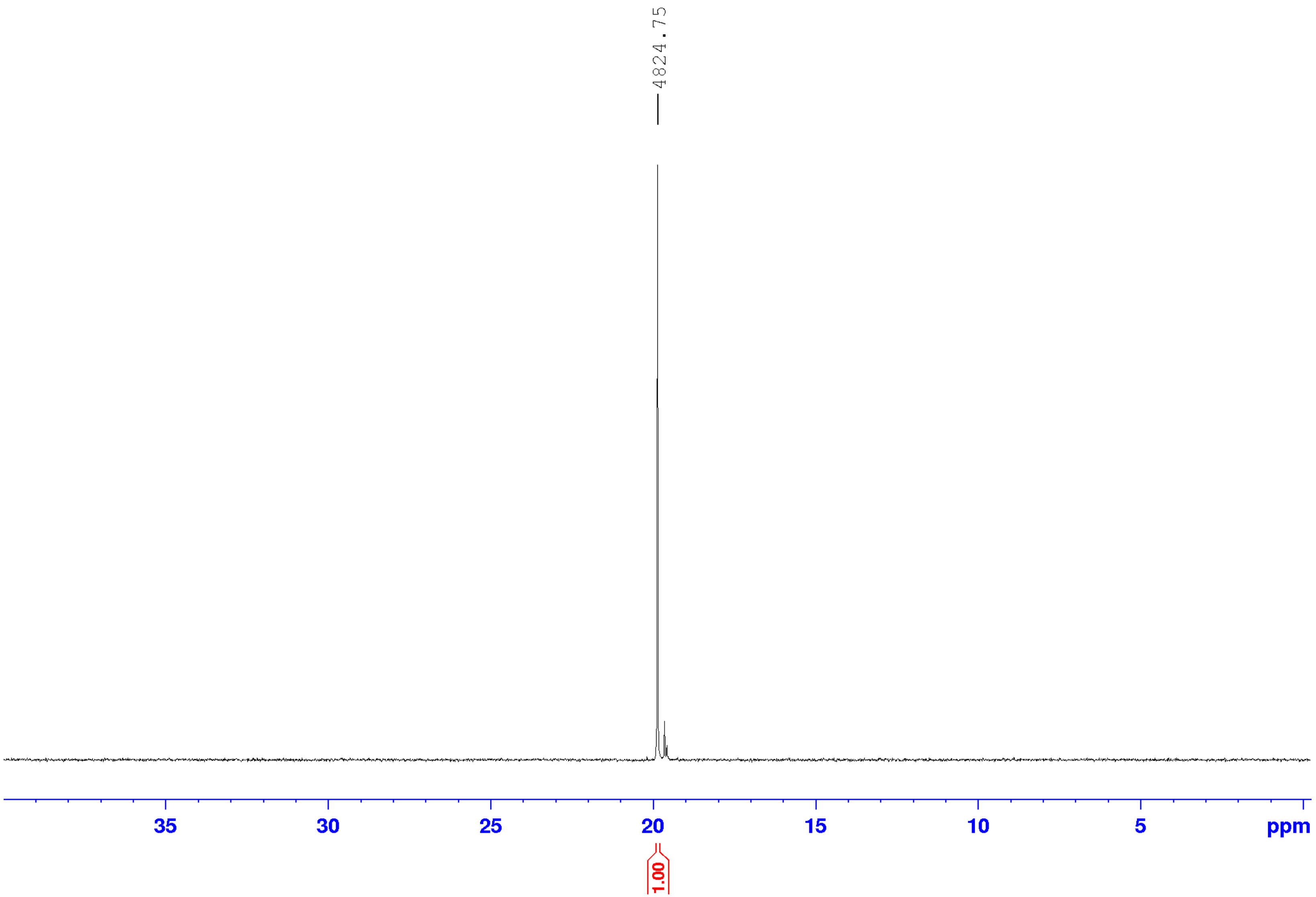

^{13}C NMR of (R)-diisopropyl-(1,5-diazidopentyl)phosphonate (150.93 MHz, $CDCl_3$) [(*R*)-77]:

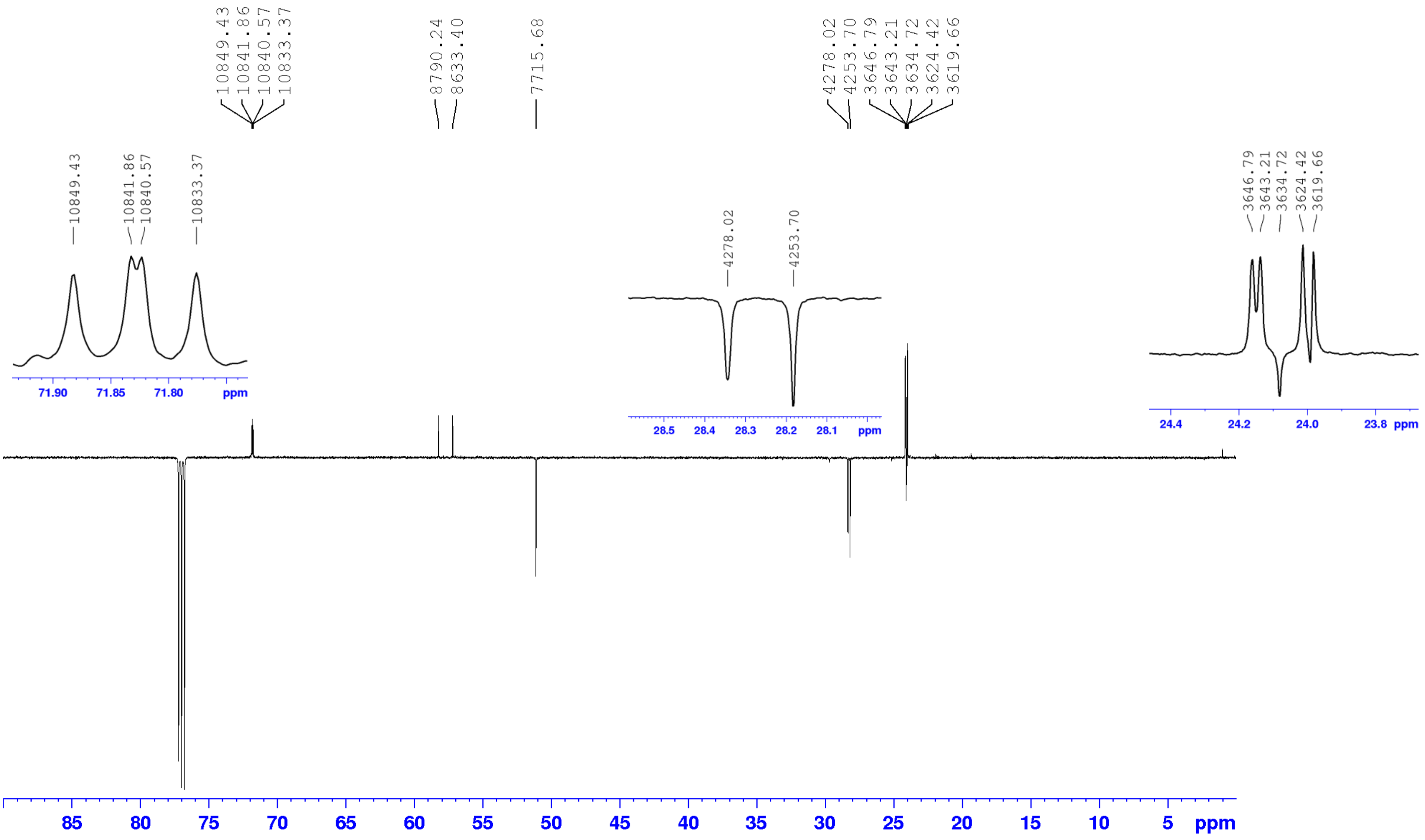

^{1}H NMR of (*R*)-(1,5-diaminopentyl)phosphonic acid, (*R*)-phosphalysine (600.25 MHz, D_2O) [(*R*)-63]:

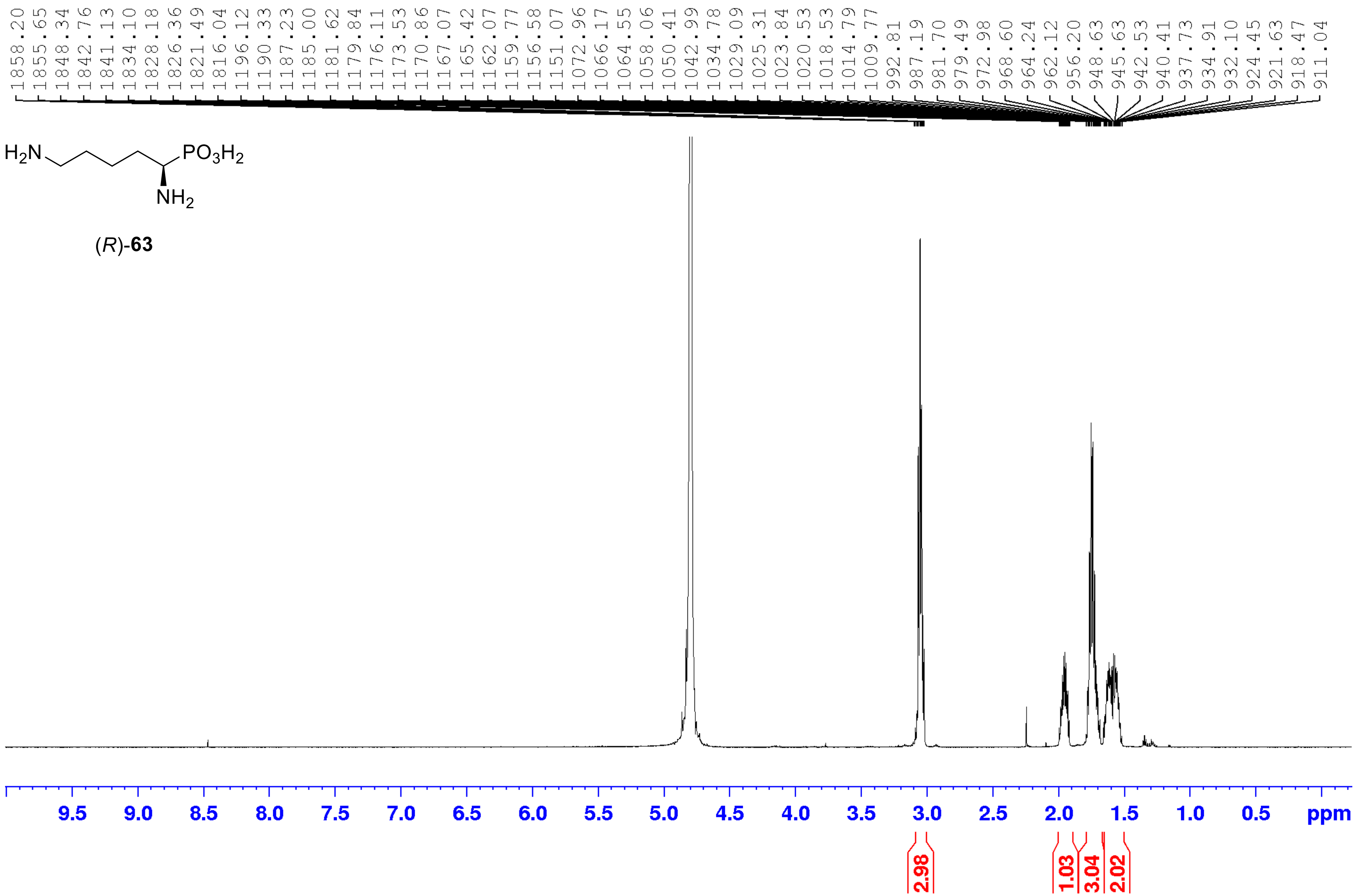

^{31}P NMR of (R)-(1,5-diaminopentyl)phosphonic acid, (*R*)-phosphalysine (162.03 MHz, D_2O) [(*R*)-63]:

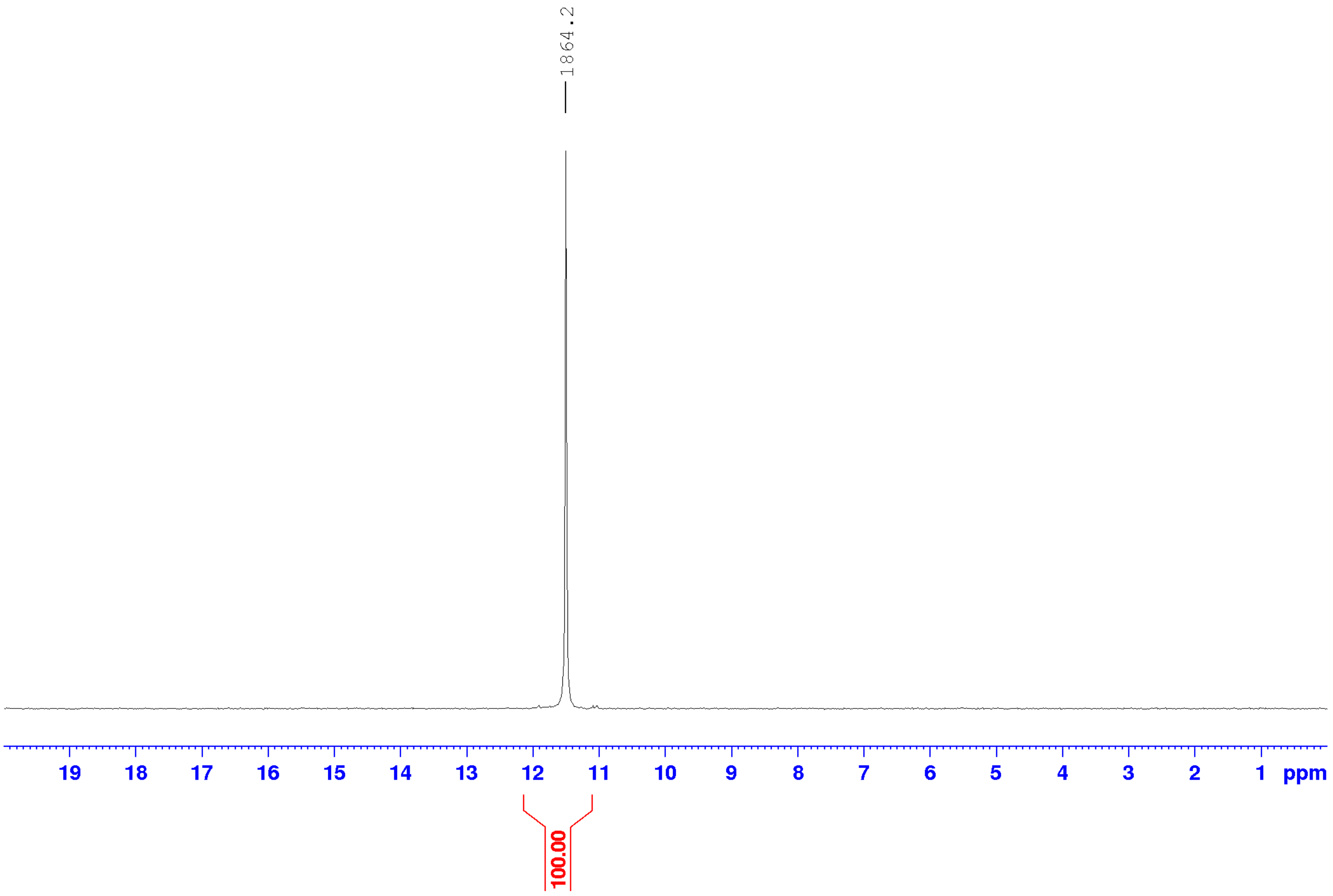

^{13}C NMR of (R)-(1,5-diaminopentyl)phosphonic acid, (*R*)-phosphalysine (150.93 MHz, D_2O) [(*R*)-63]:

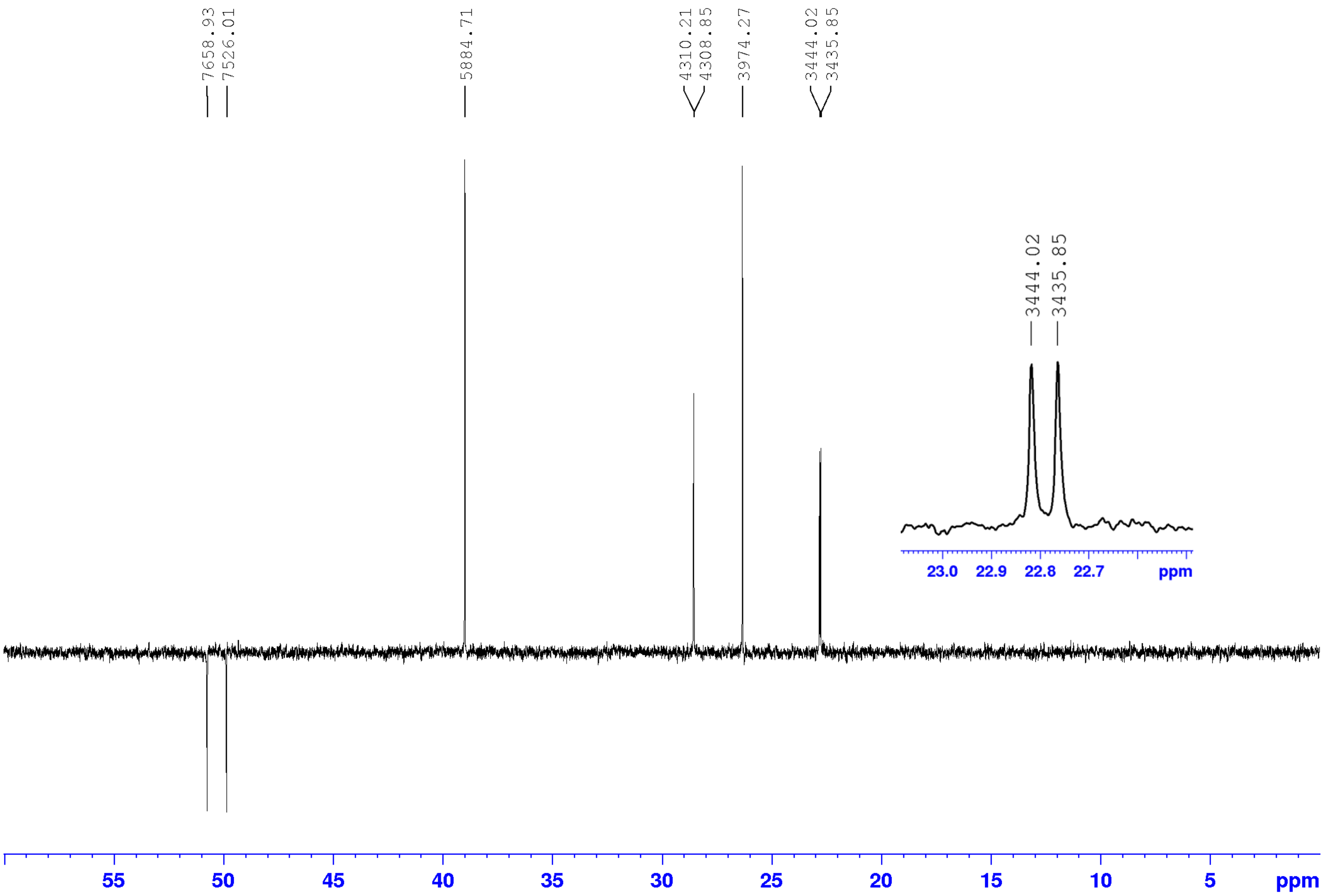

^{1}H NMR of (*R*)-diisopropyl 1-azidopent-4-en-1-yl-phosphonate (400.27 MHz, $CDCl_3$)[(*R*)-78]:

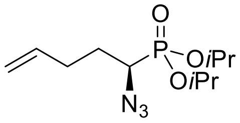

(*R*)-**78**

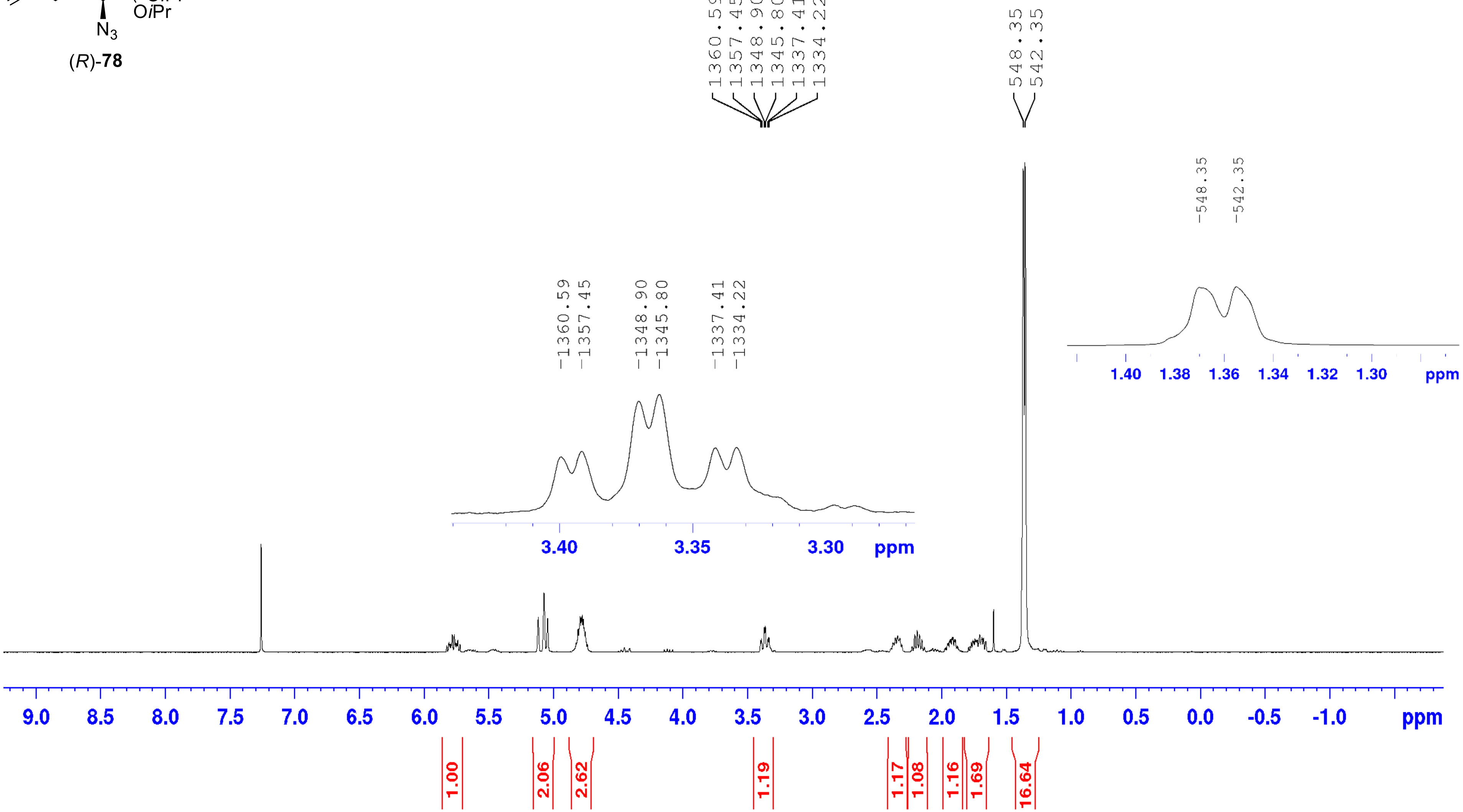

^{31}P NMR of (*R*)-diisopropyl 1-azidopent-4-en-1-yl-phosphonate (162.02 MHz, CDCl$_3$) [(*R*)-78]:

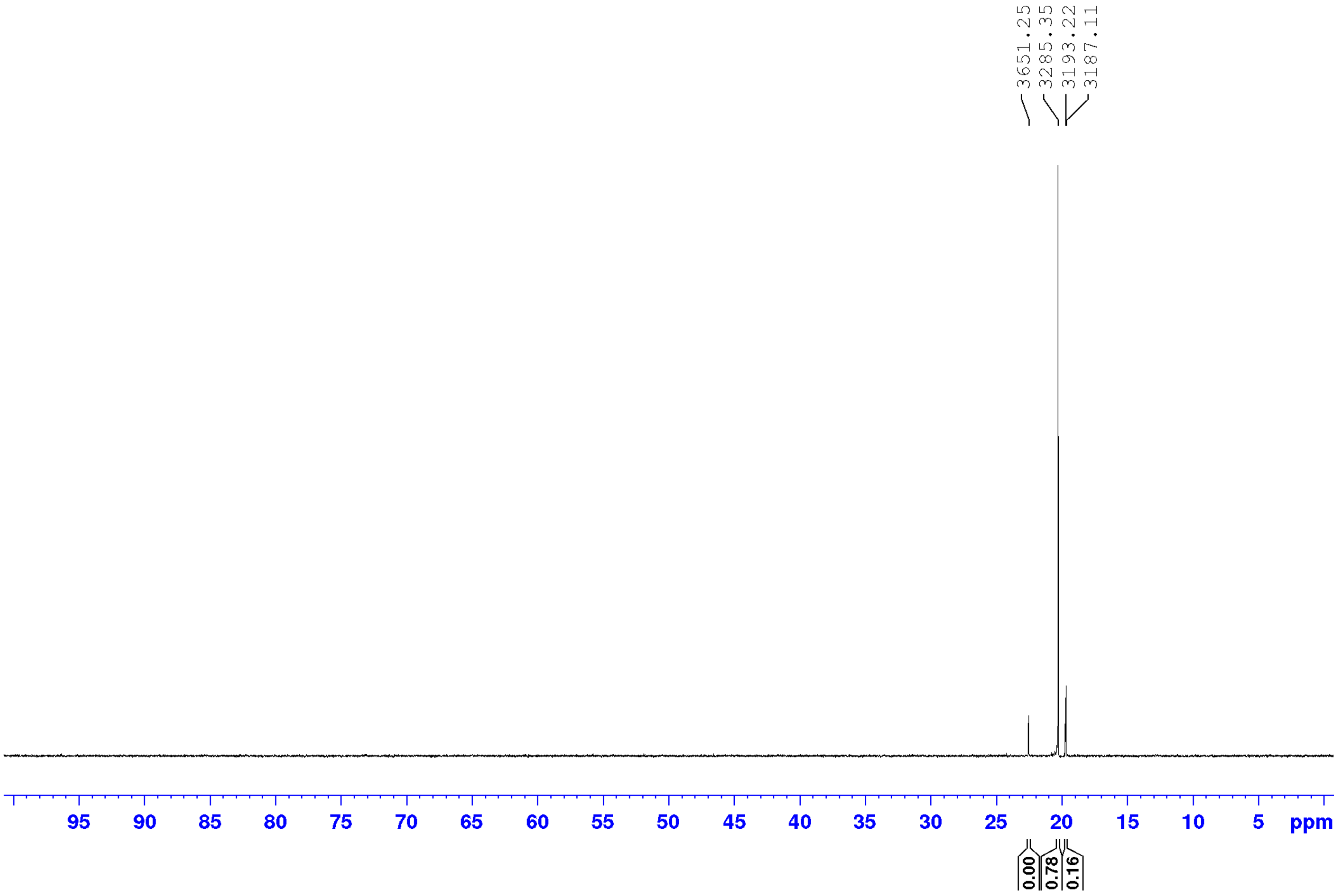

^{1}H NMR of (*R*)-diisopropyl 1-azido-4-hydroxybutylphosphonate (700.40 MHz, $CDCl_3$) [(*R*)-79]:

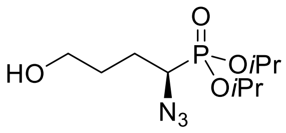

(*R*)-**79**

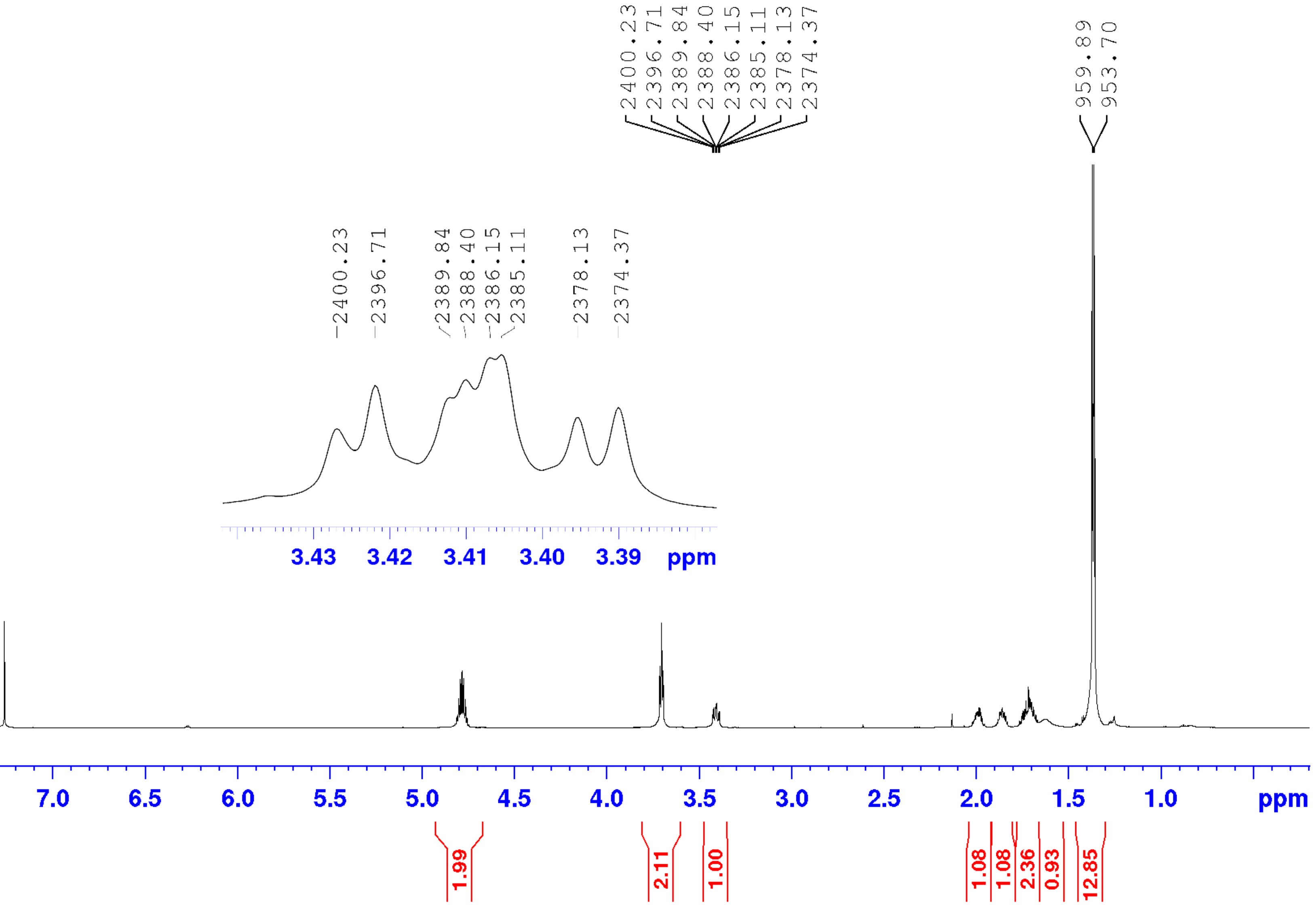

^{13}C NMR of (*R*)-diisopropyl 1-azido-4-hydroxybutylphosphonate (176.12 MHz, $CDCl_3$) [(*R*)-79]:

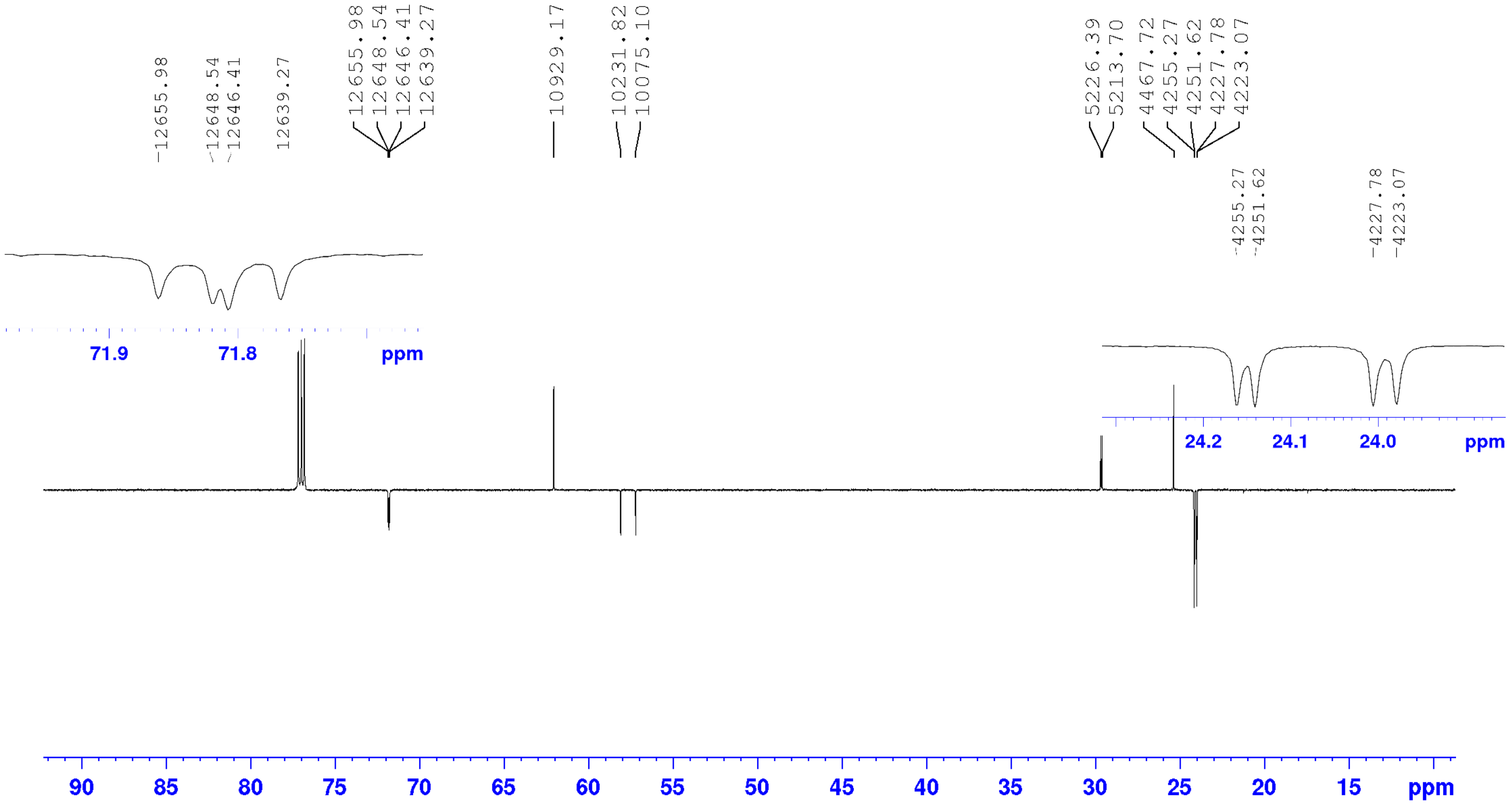

^{31}P NMR of (*R*)-diisopropyl 1-azido-4-hydroxybutylphosphonate (162.02 MHz, $CDCl_3$) [(*R*)-79]:

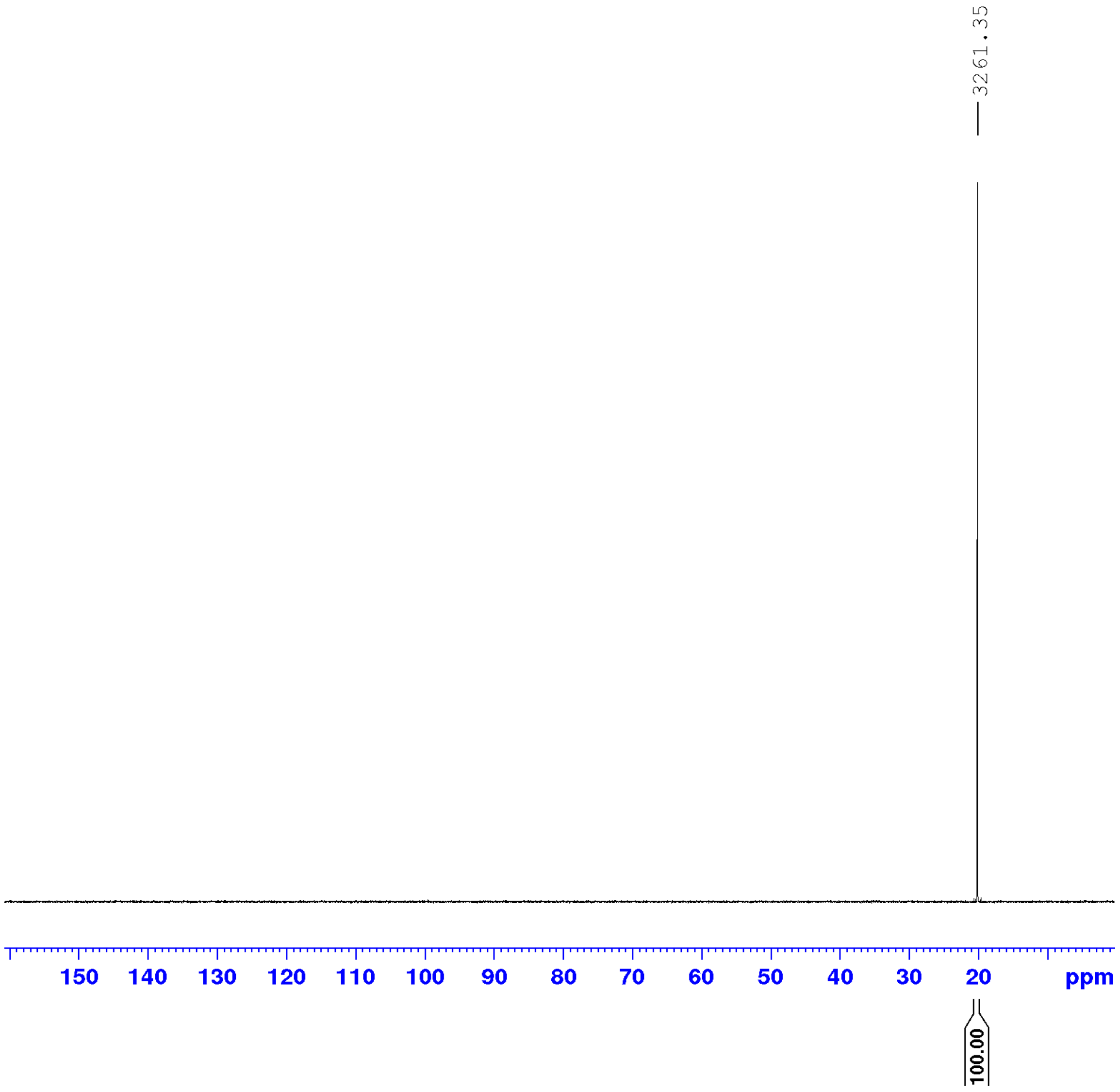

^{1}H NMR of (*R*)-diisopropyl [1-azido-4-bis(*tert*-butoxycarbonyl)guanidino]butylphosphonate (700.40 MHz, $CDCl_3$) [(*R*)-80]:

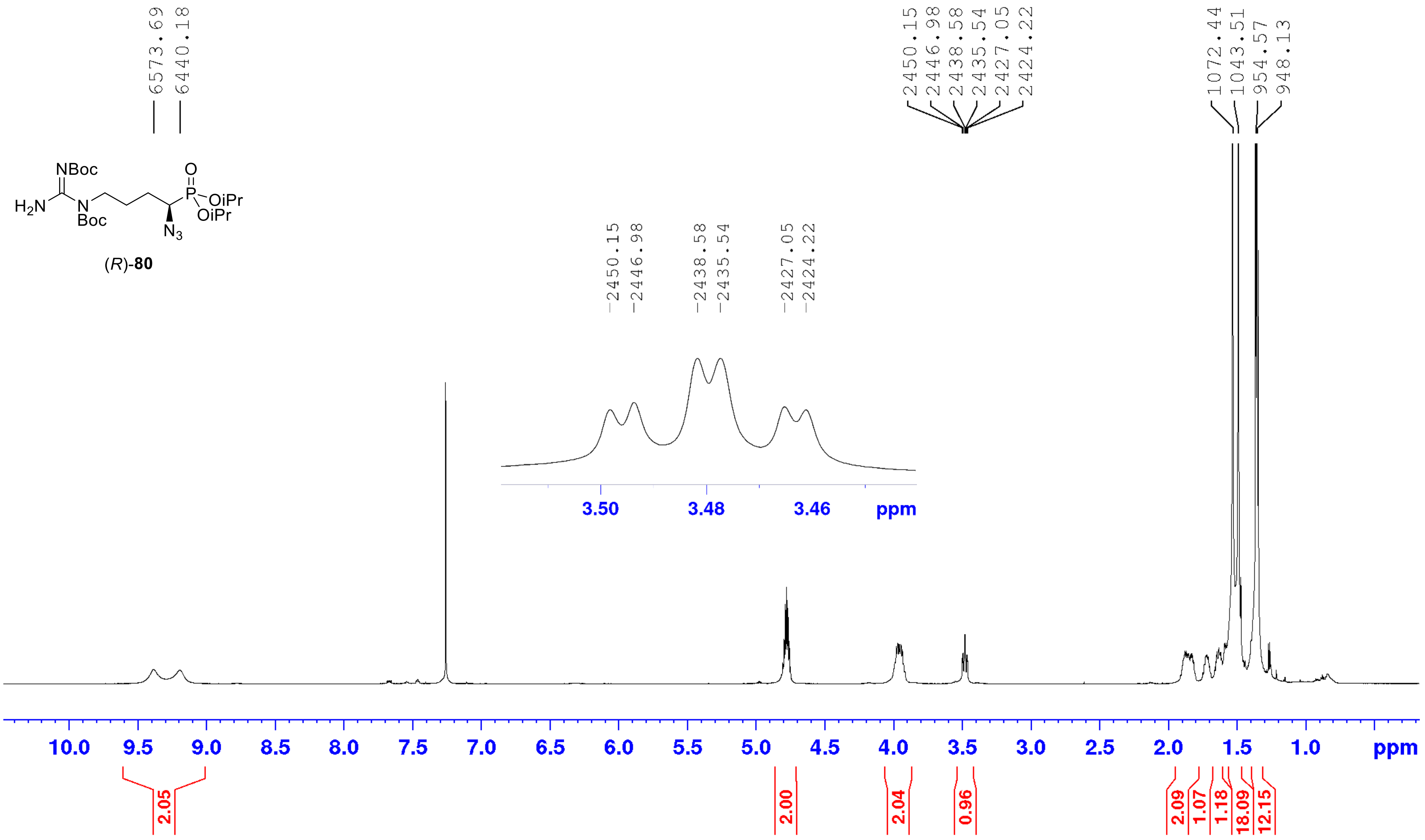

^{13}C NMR of (*R*)-diisopropyl [1-azido-4-bis(*tert*-butoxycarbonyl)guanidino]butylphosphonate (176.12 MHz, $CDCl_3$) [(*R*)-80]:

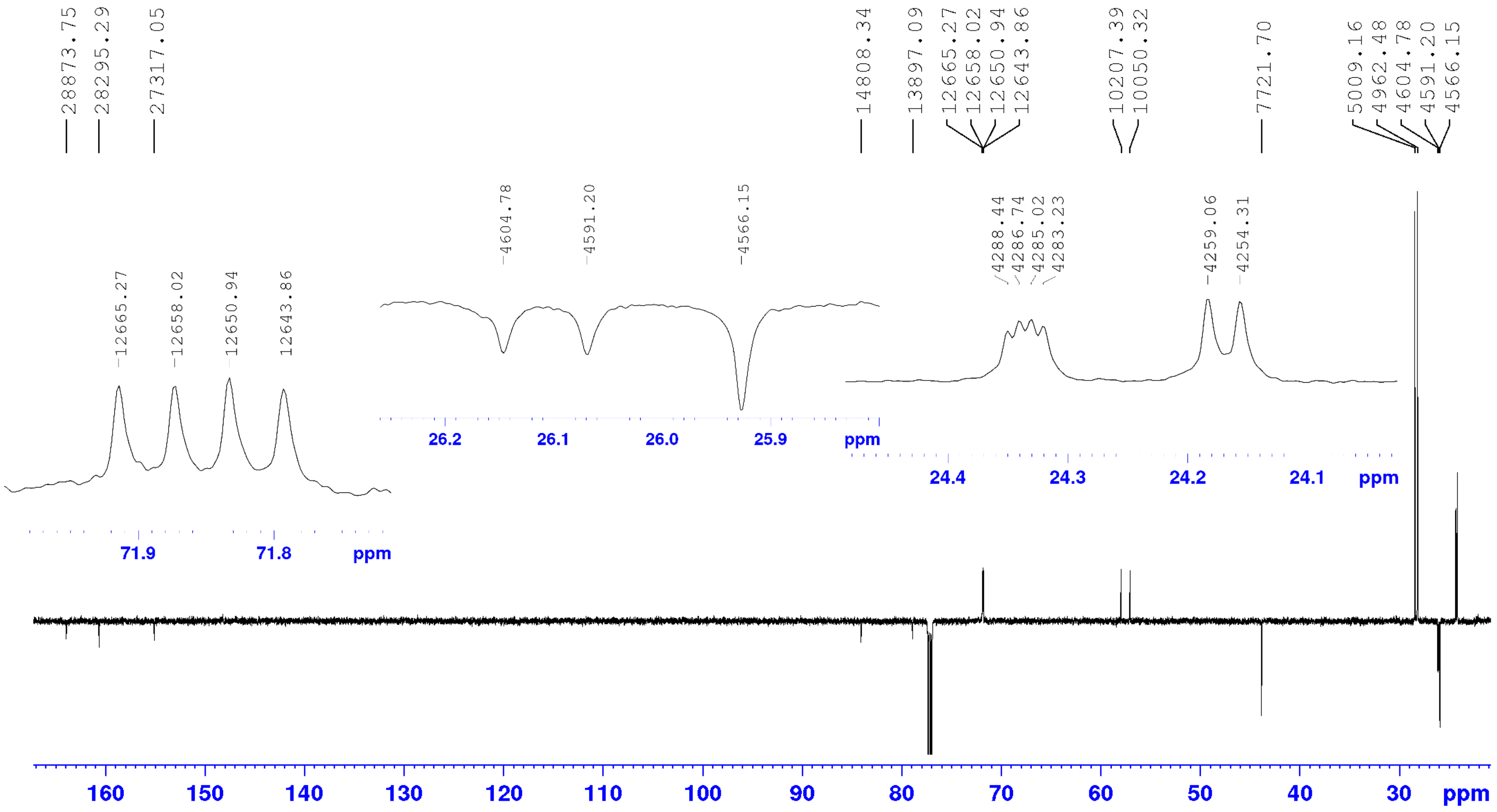

^{31}P NMR of (*R*)-diisopropyl [1-azido-4-bis(*tert*-butoxycarbonyl)guanidino]butylphosphonate (162.02 MHz, $CDCl_3$) [(*R*)-80]:

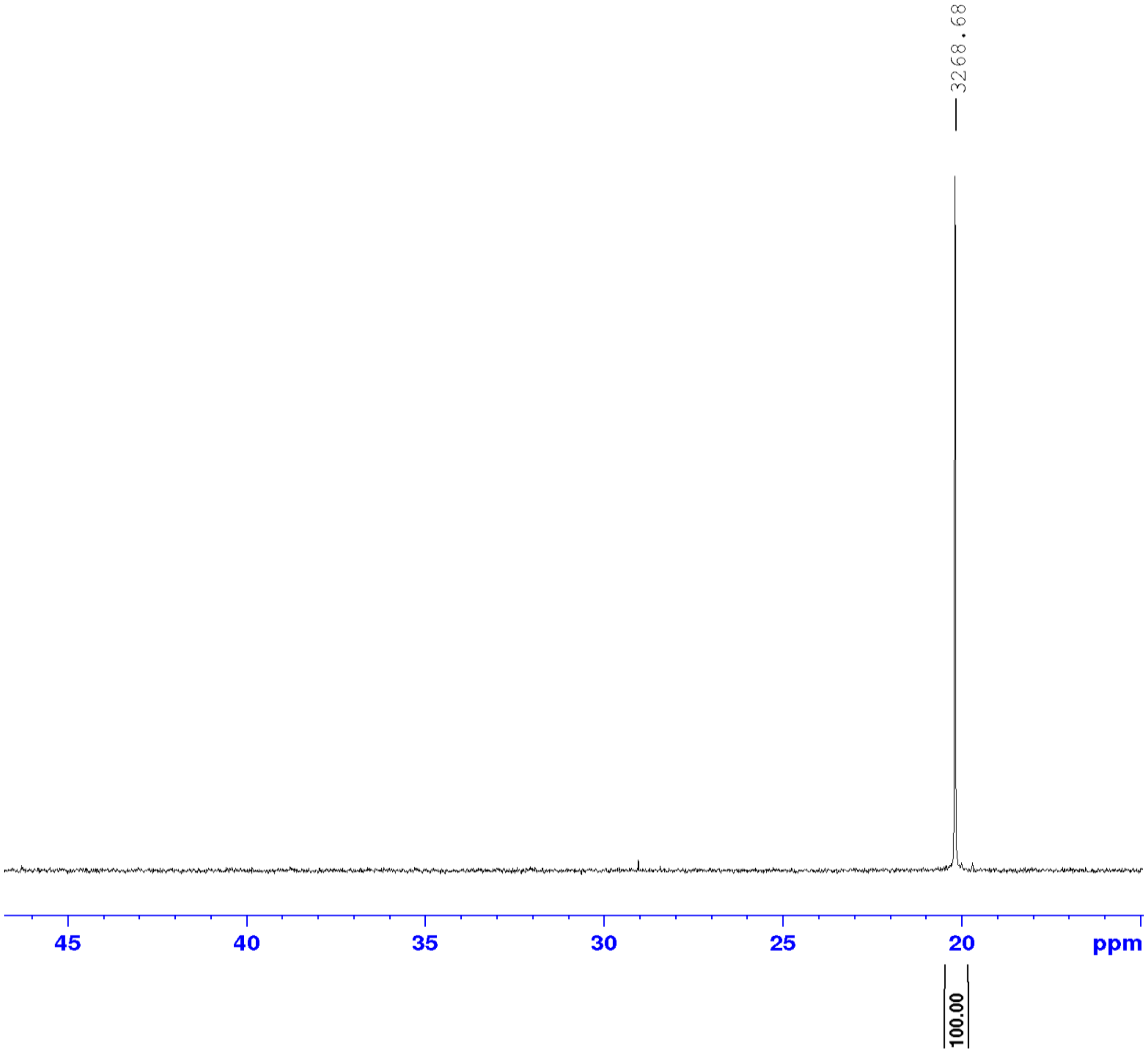

^{31}P NMR of diisopropyl (2-phenylacetyl)phosphonate (CDCl$_3$, 400.27 MHz) (13):

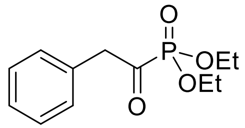

13

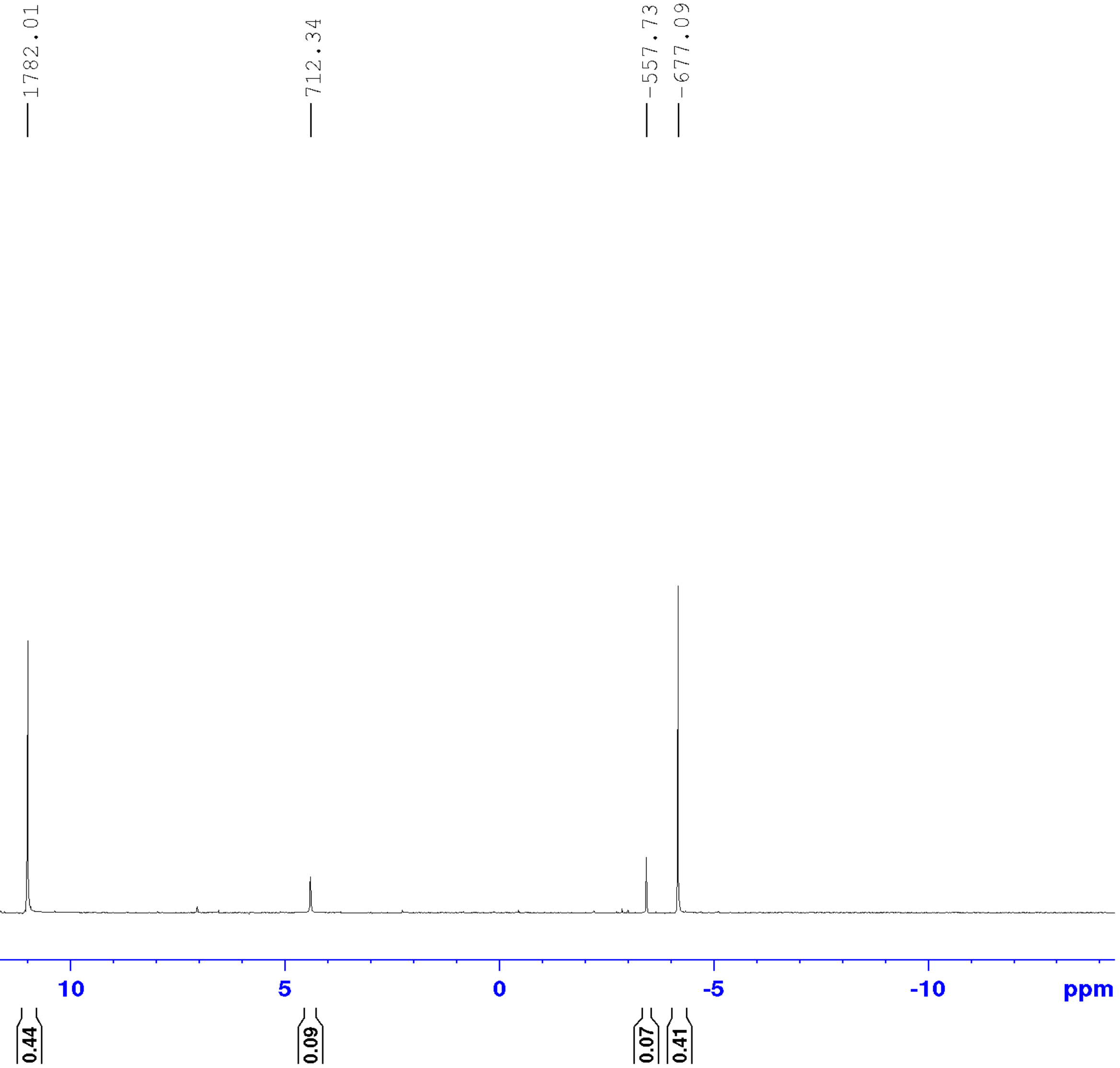

^{1}H NMR of (*S*)-diisopropyl (2-phenylacetyl)phosphonate ($CDCl_3$, 400.27 MHz) [(*S*)-30]:

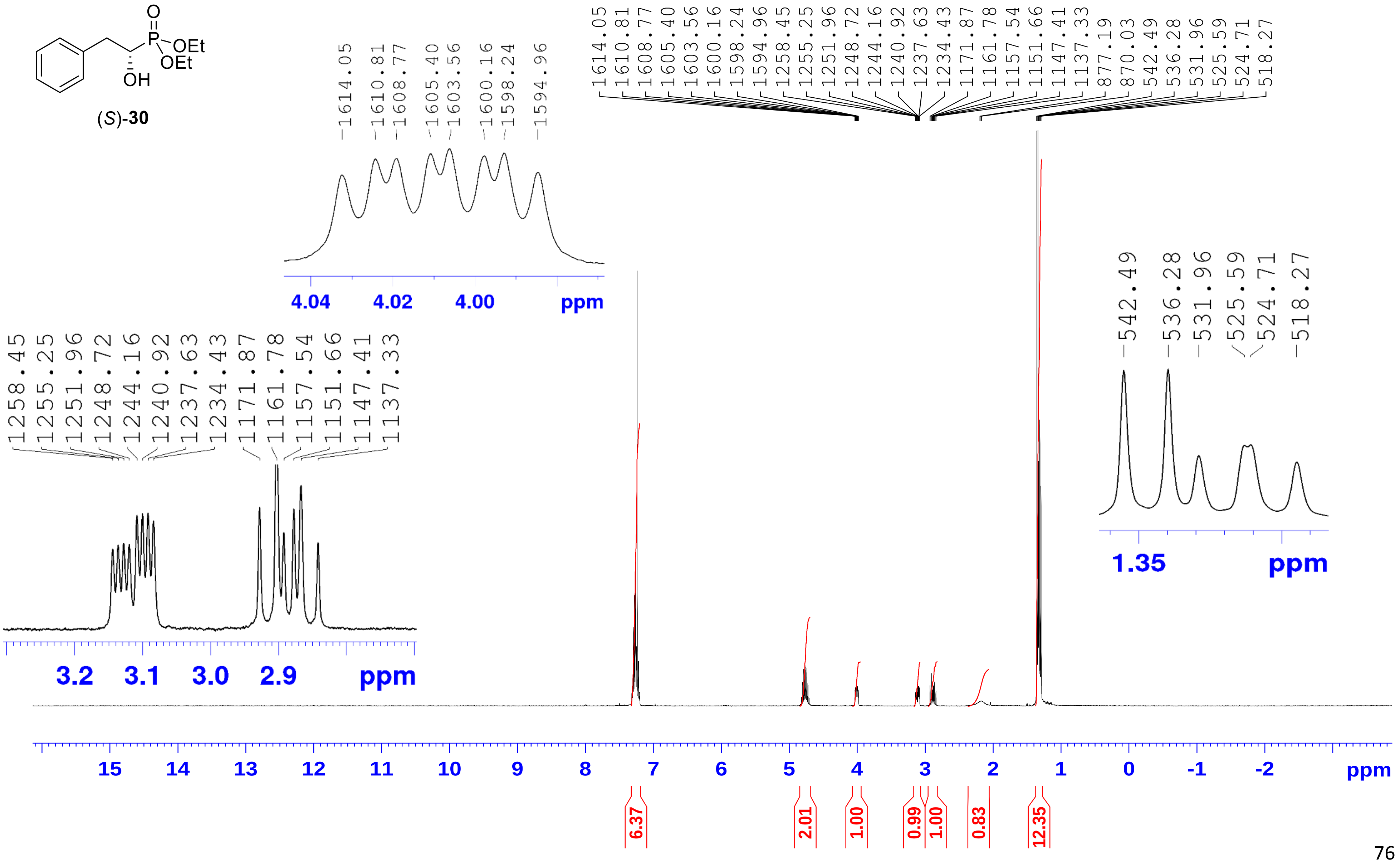

^{13}C NMR of (*S*)-diisopropyl (2-phenylacetyl)phosphonate ($CDCl_3$, 100.65 MHz) [(*S*)-30]:

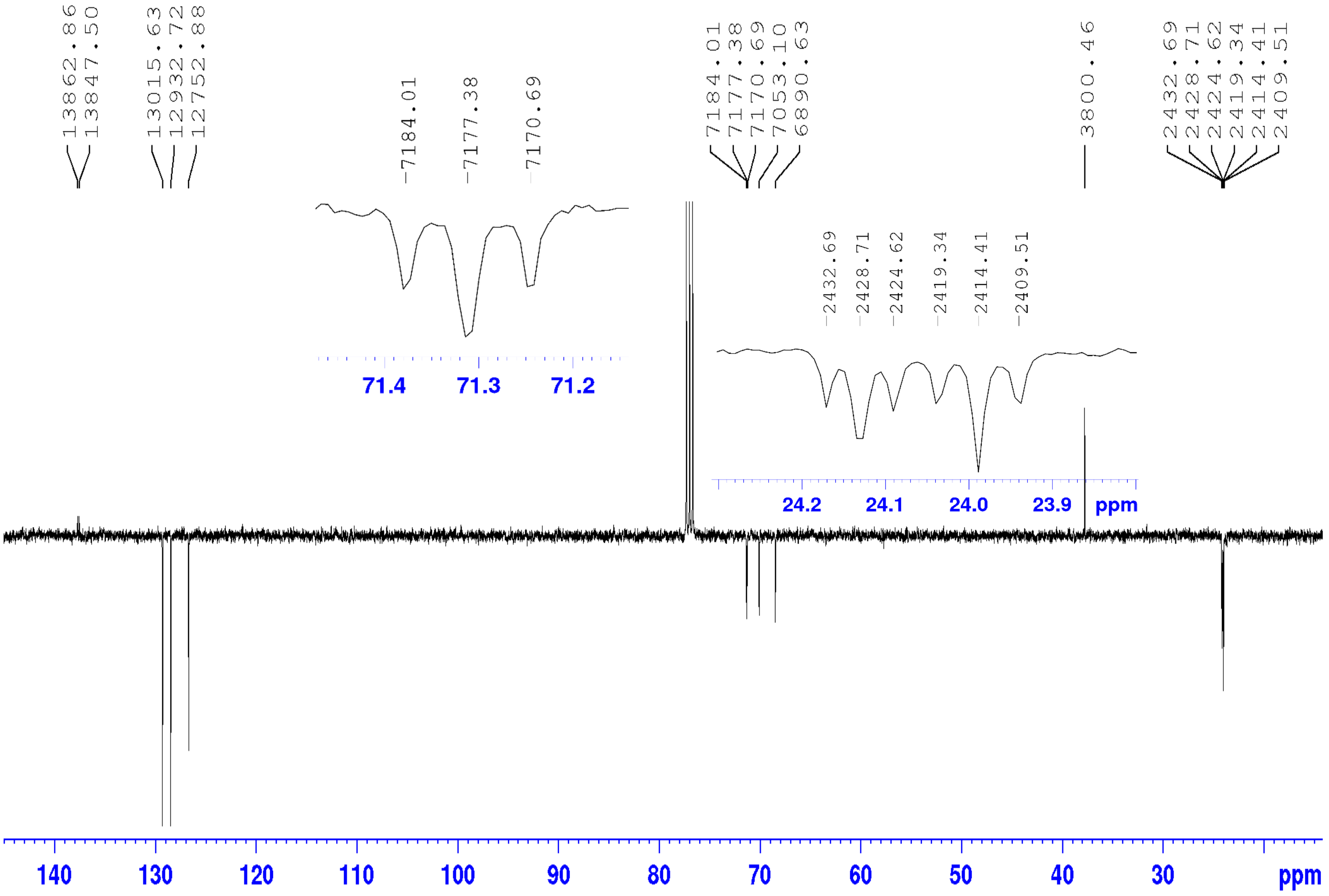

^{31}P NMR of (*S*)-diisopropyl (2-phenylacetyl)phosphonate ($CDCl_3$, 162.04 MHz) [(*S*)-30]:

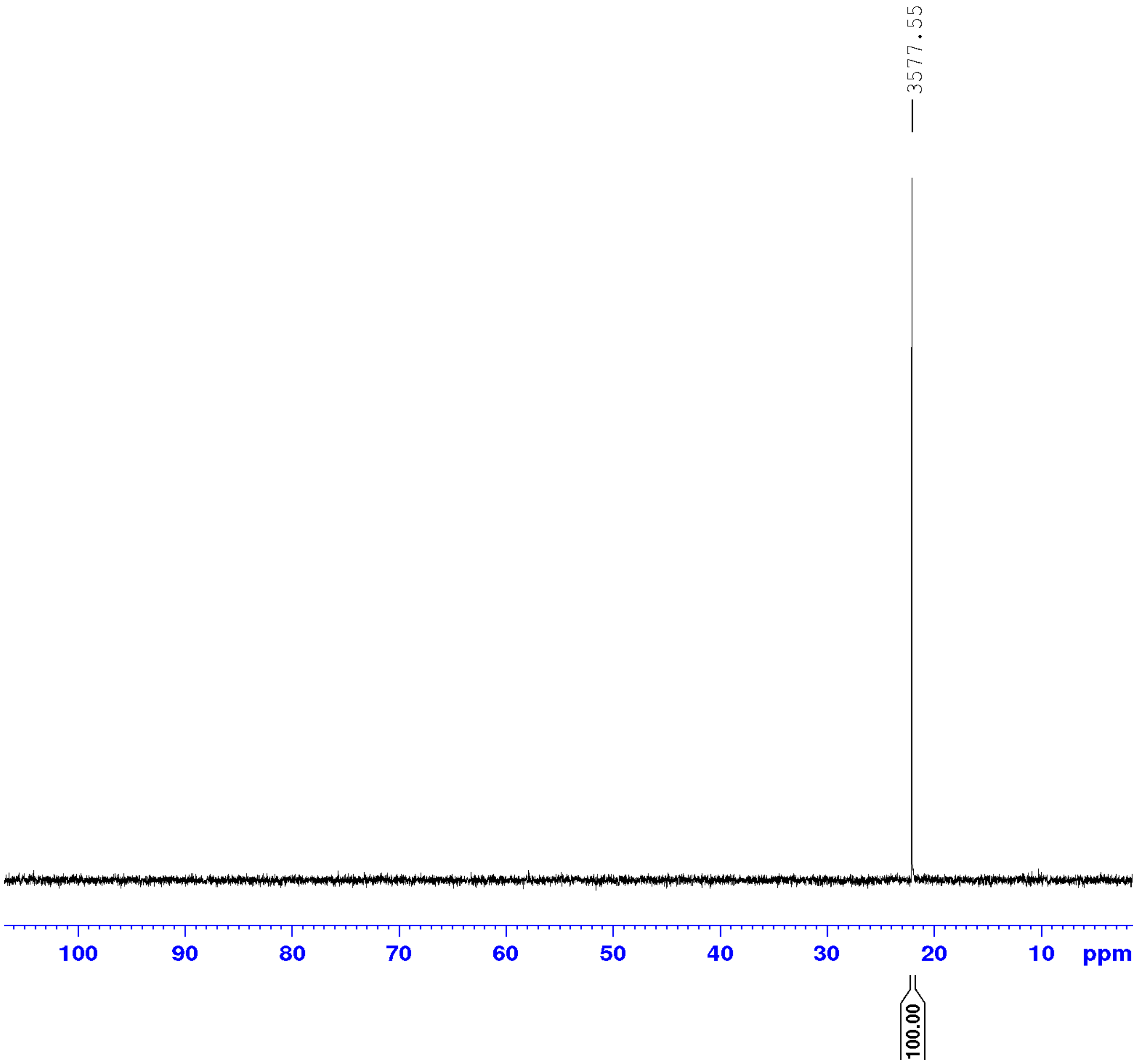

^{1}H NMR of (*R*)-diisopropyl (1-azido-2-phenylethyl)phosphonate ($CDCl_3$, 400.27 MHz) [(*R*)-81]:

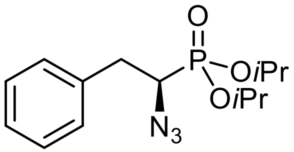

(*R*)-**81**

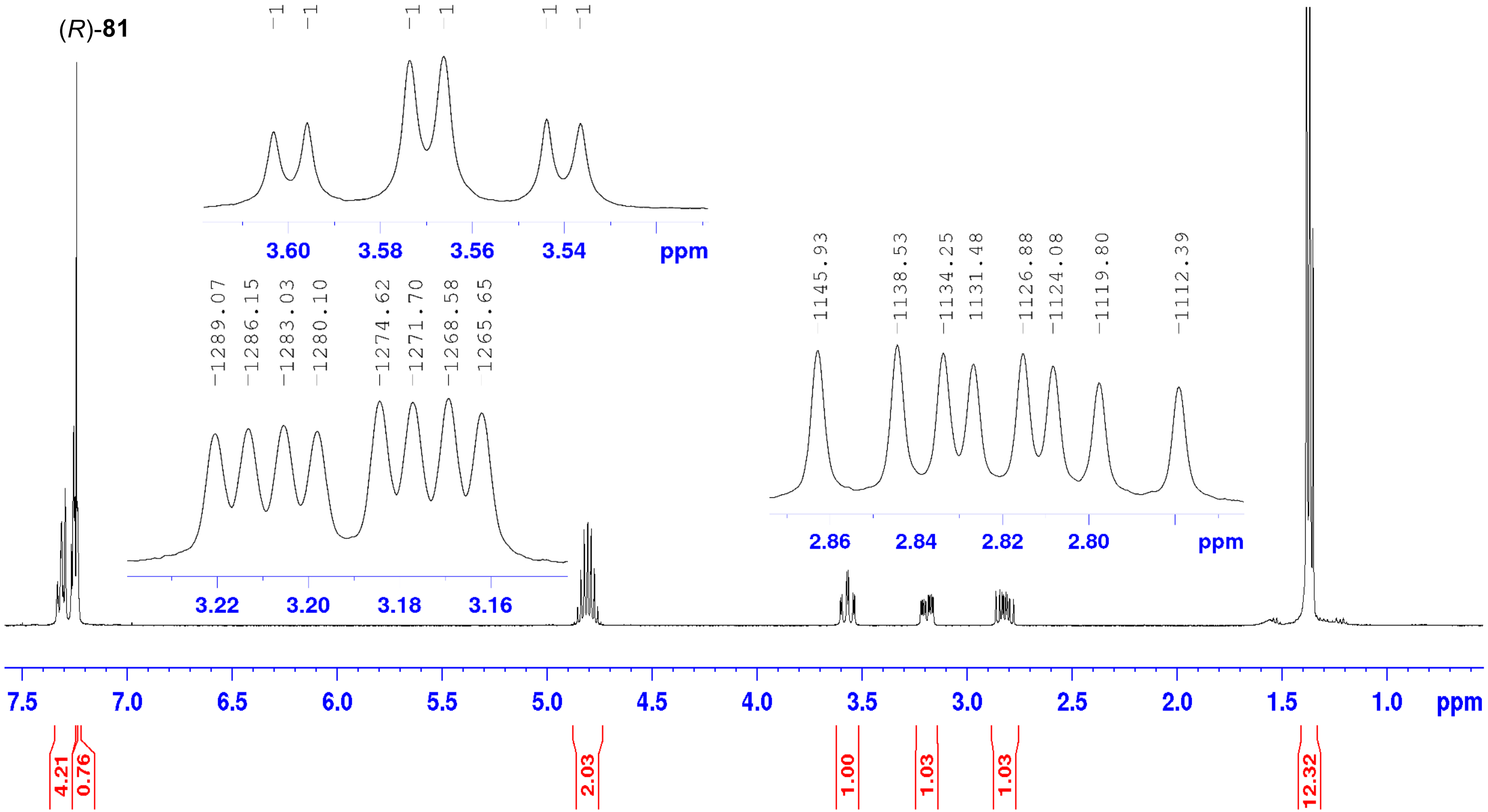

^{13}C NMR of (*R*)-diisopropyl (1-azido-2-phenylethyl)phosphonate ($CDCl_3$, 100.65 MHz) [(*R*)-81]:

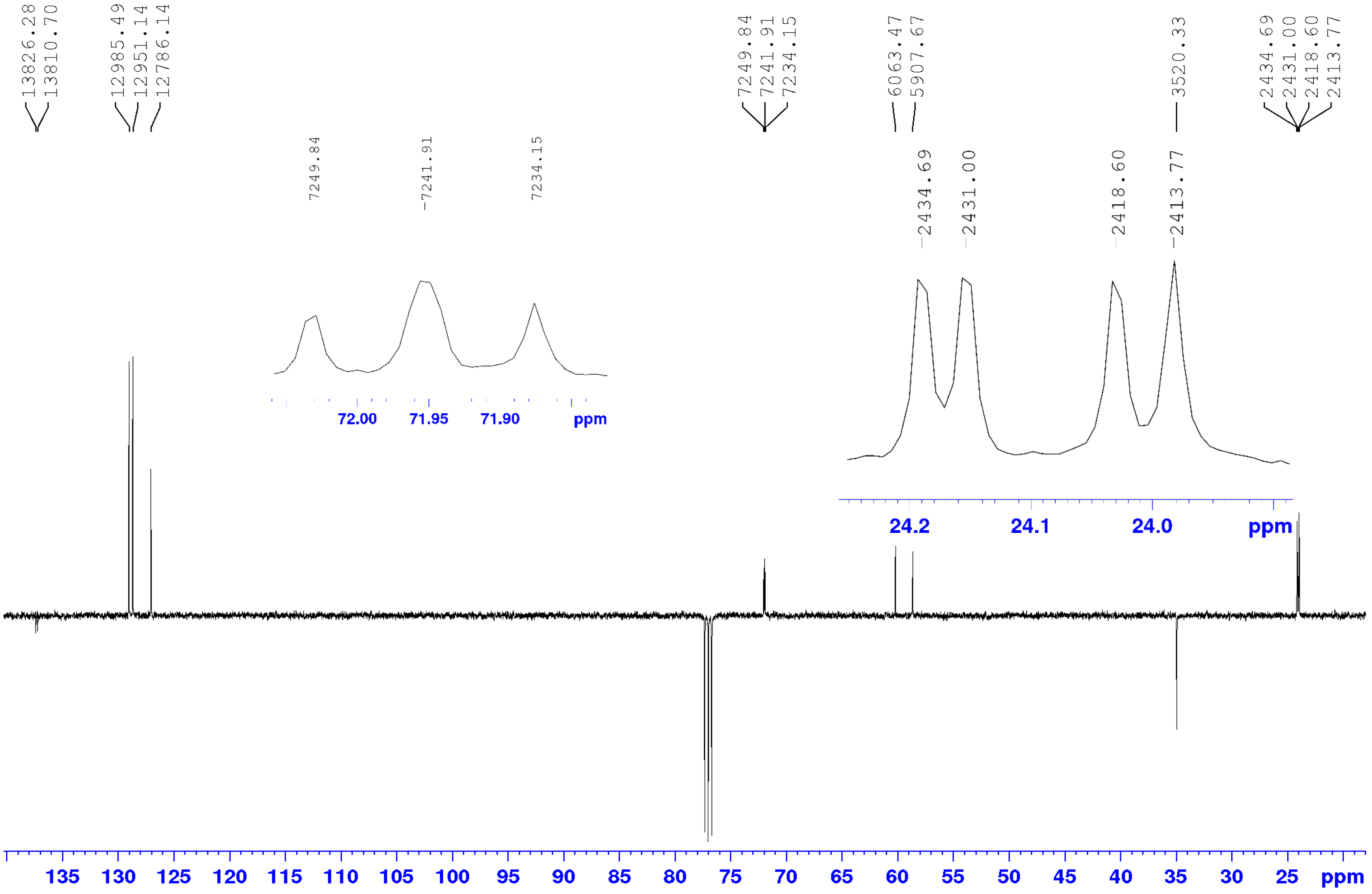

^{31}P NMR of (*R*)-diisopropyl (1-azido-2-phenylethyl)phosphonate ($CDCl_3$, 162.04 MHz) [(*R*)-81]:

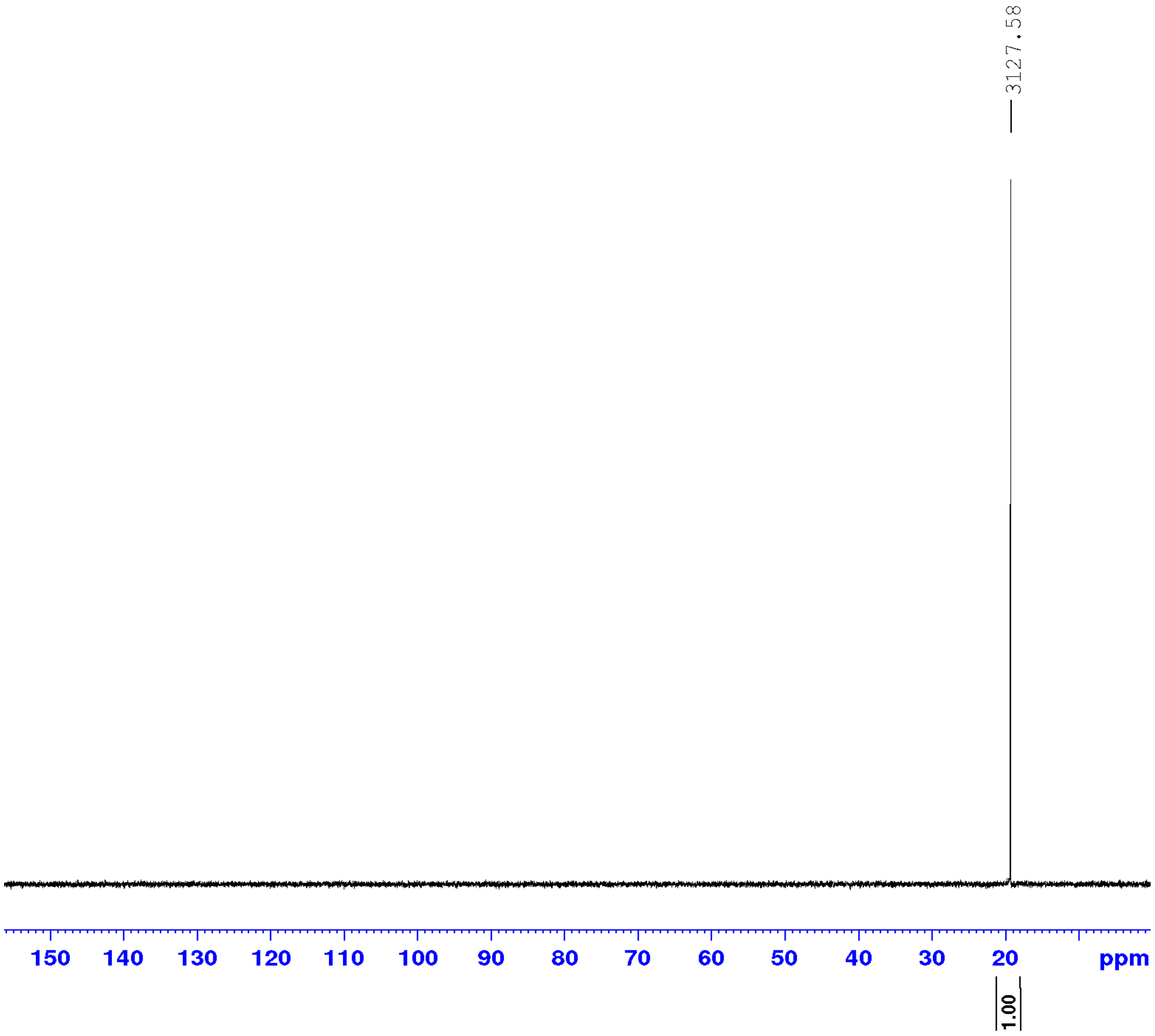

^{1}H NMR of (*R*)-(1-amino-2-phenylethyl)phosphonic acid, (*R*)-phosphaphenylalanine (D_2O, 600.25 MHz) [(*R*)-64]:

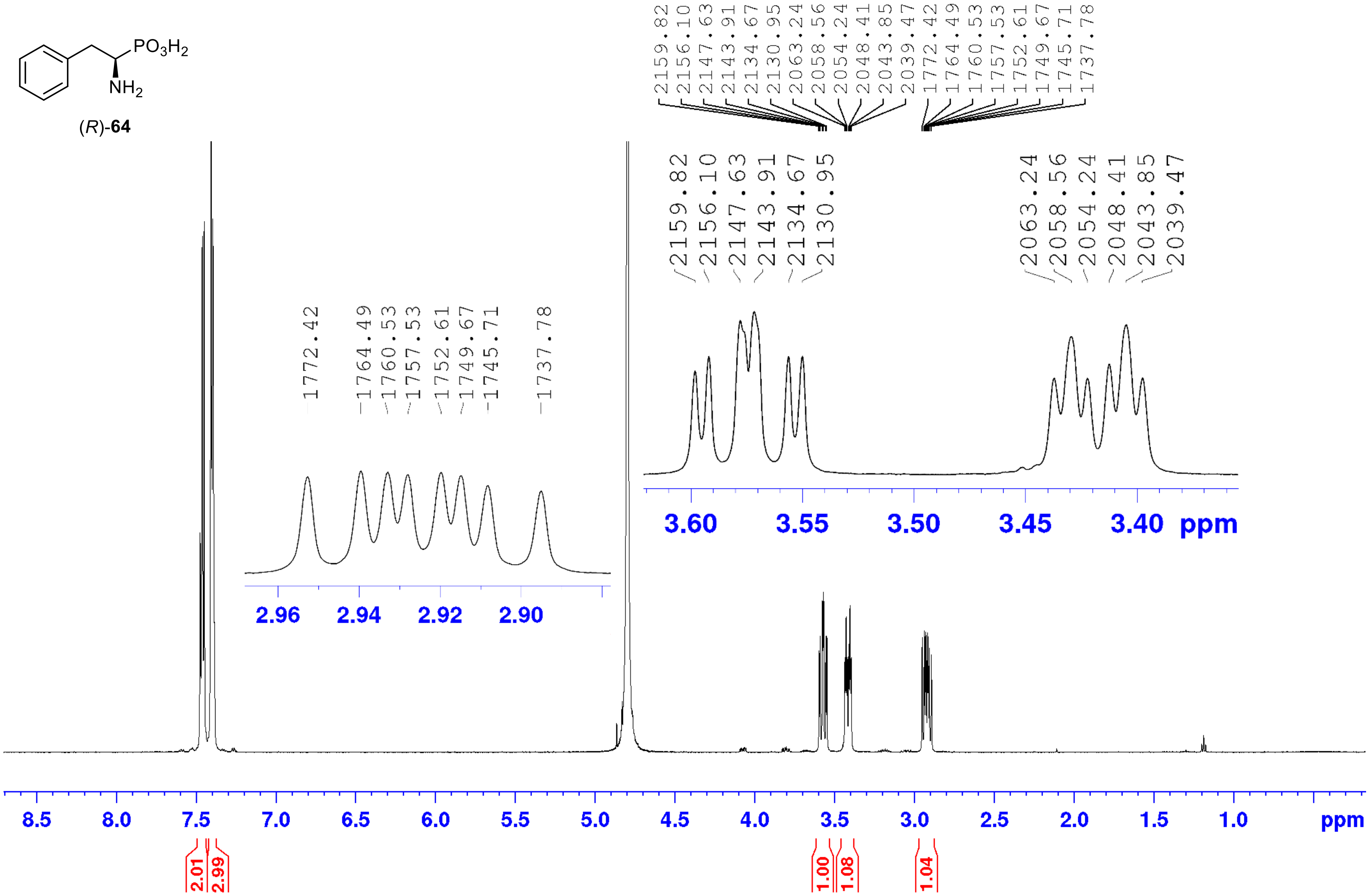

^{13}C NMR of (*R*)-(1-amino-2-phenylethyl)phosphonic acid, (*R*)-phosphaphenylalanine (D_2O, 150.94 MHz) [(*R*)-64]:

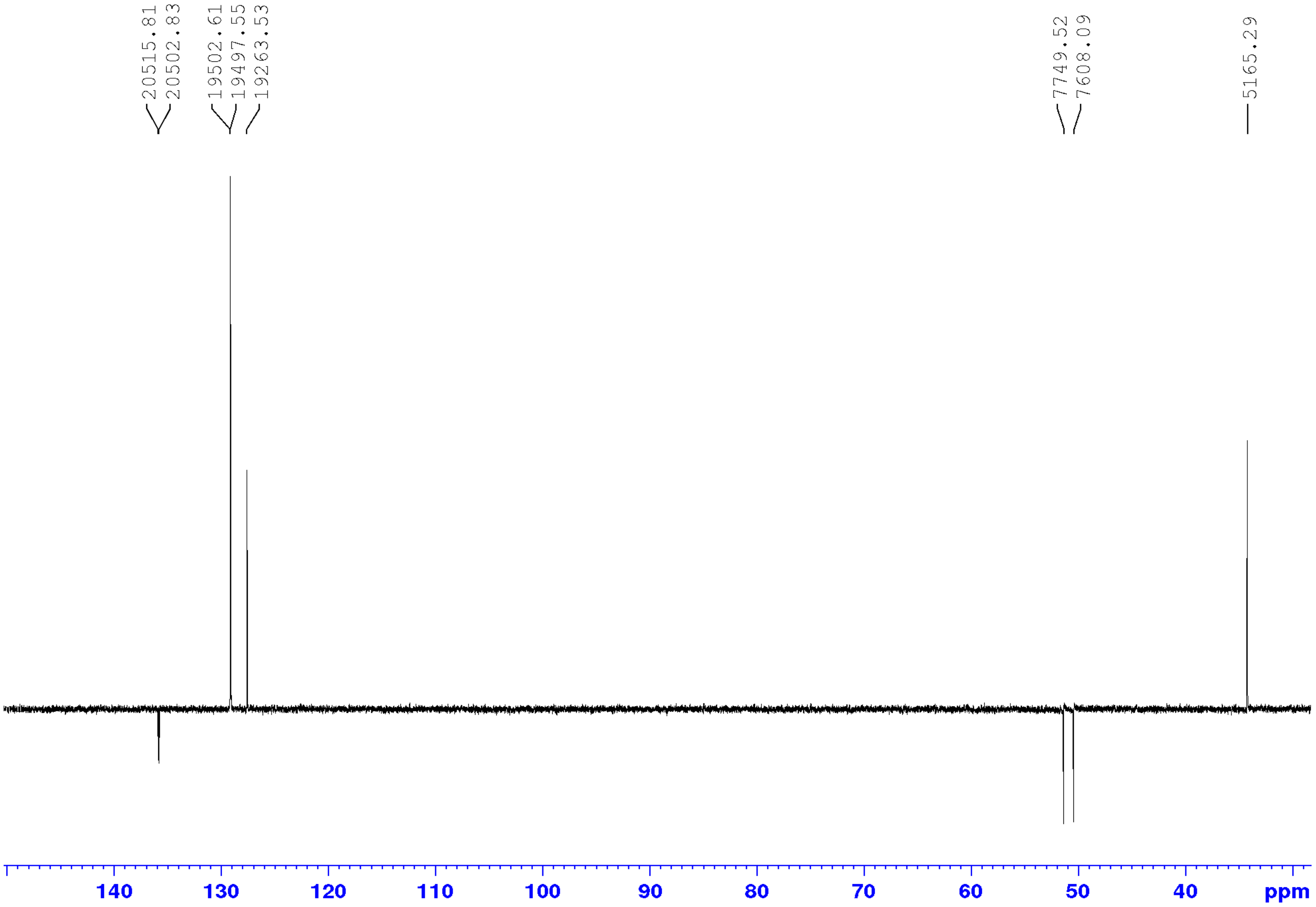

^{31}P NMR of (*R*)-(1-amino-2-phenylethyl)phosphonic acid, (*R*)-phosphaphenylalanine (D_2O, 242.97 MHz) [(*R*)-64]:

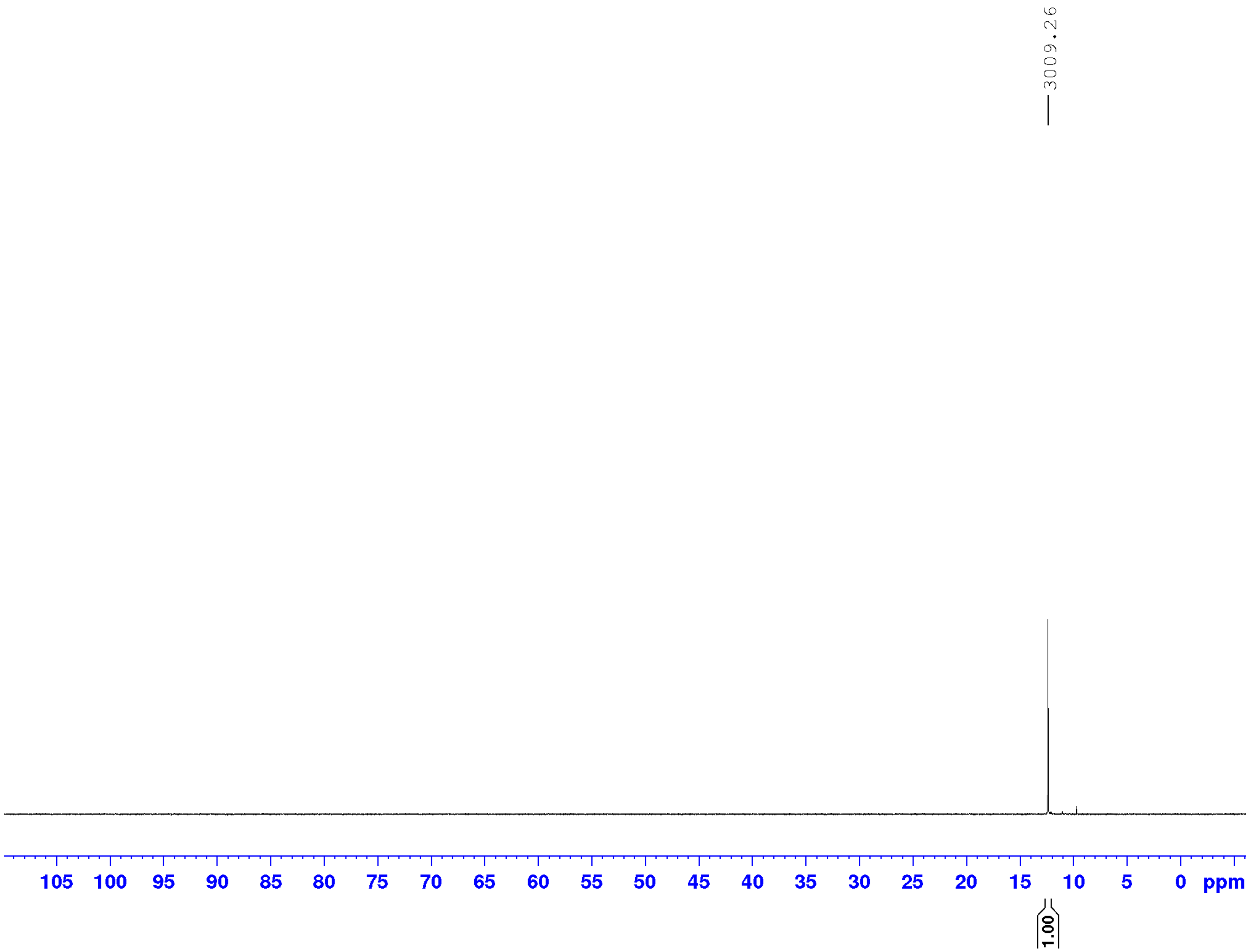

***ee* determination of (*R*)-64 by chiral stationary phase HPLC after derivatization as shown below**

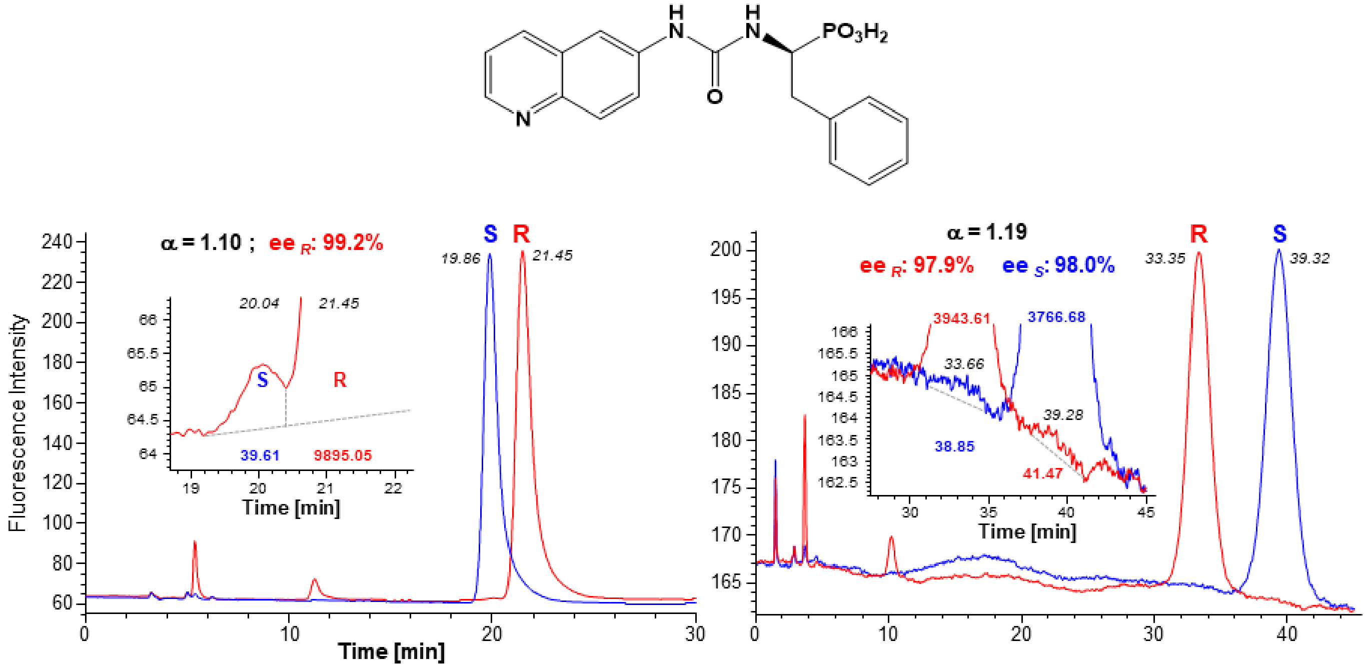

Chiral Separation of AQC-derivatized phosphaphenylalanine (*R*)-**64** and (*S*)-**64** on *a)* an unmodified OH-QN-AX and *b)* an OH-QD-AX column (150 mm x 4 mm, 5 µm) employing the mobile phase 2 M aqueous H_3PO_4:MeOH (1:9, (v/v)), pH 4.0 (adjusted with trimethylamine) at a flow rate of 0.5 mL/min for *a)* and 1 mL/min for *b),* a column temperature of 40°C and fluorescence detection (ex. 250 nm ; em .395 nm) was used. Peak areas are in bold and retention times are in italic.

^{31}P NMR of benzyl 2-(2-(diethoxyphosphoryl)-2-oxoethyl)-1H-indole-1-carboxylate ($CDCl_3$, 400.27 MHz) (14):

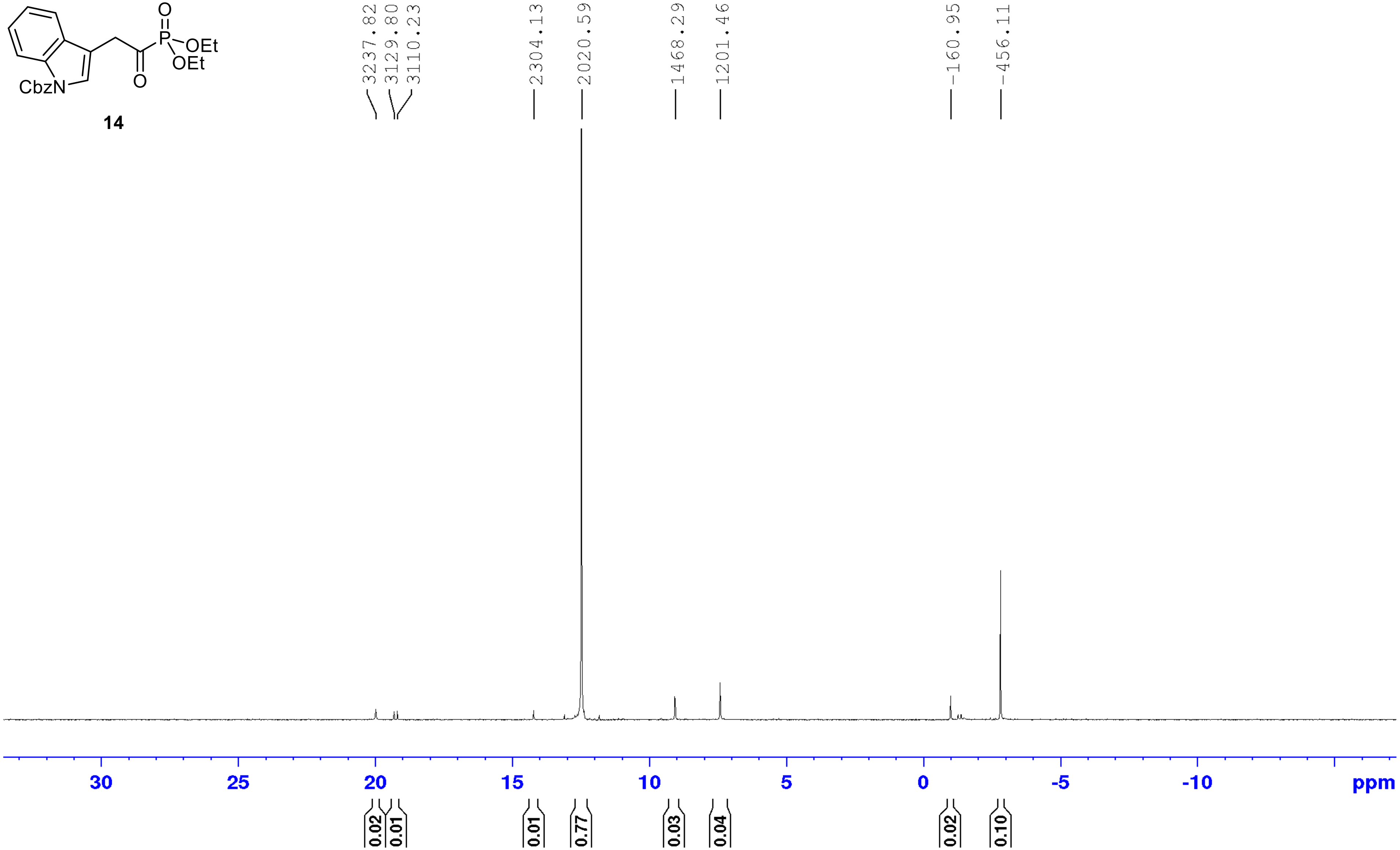

^{1}H NMR of (*S*)-diethyl (1-hydroxy-2-(1-(2-phenylacetyl)-1H-indol-2-yl)ethyl)phosphonate ($CDCl_3$, 700.40 MHz) [(*S*)-31]:

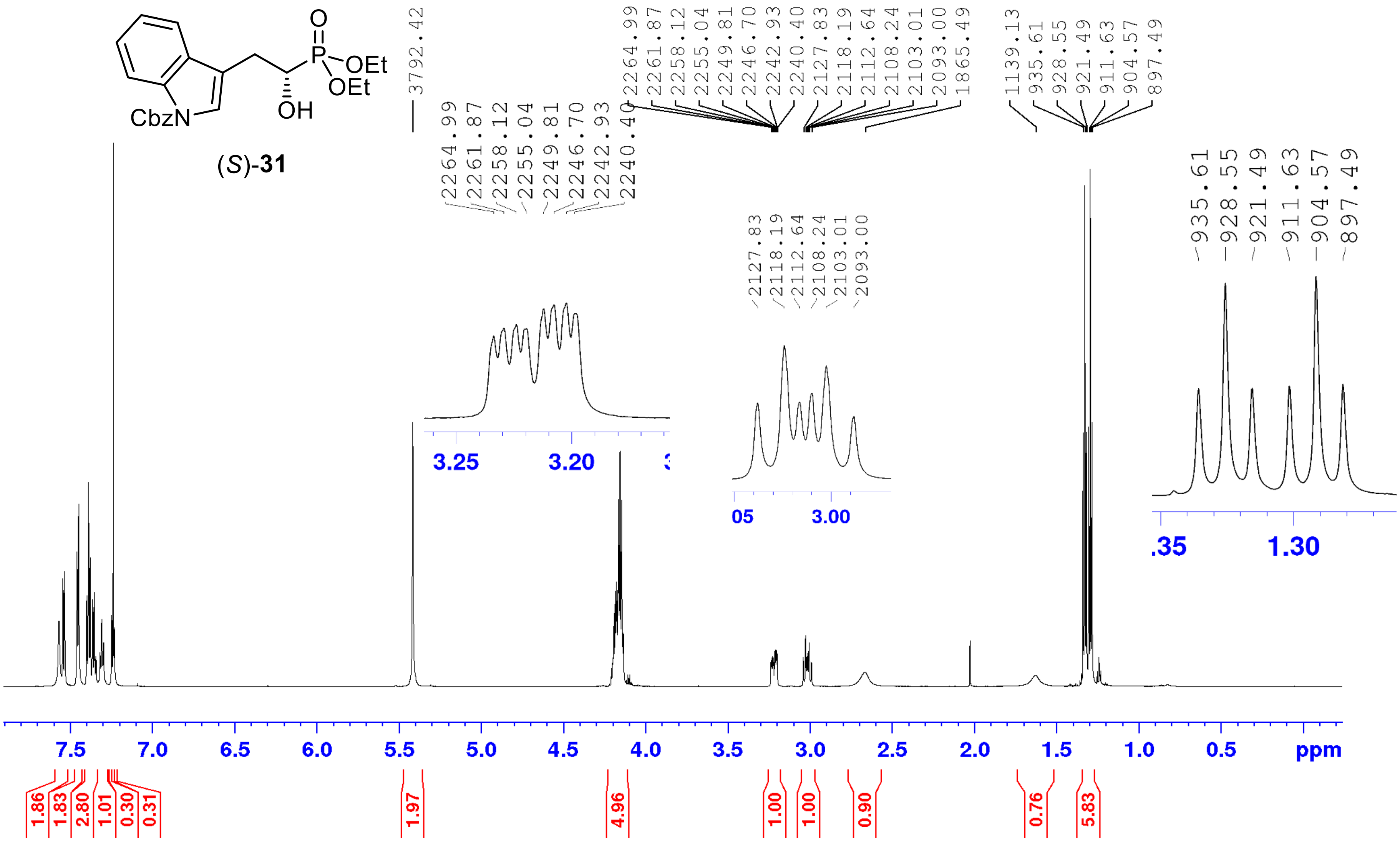

^{13}C NMR of (*S*)-diethyl (1-hydroxy-2-(1-(2-phenylacetyl)-1H-indol-2-yl)ethyl)phosphonate ($CDCl_3$, 150.93 MHz) [(*S*)-31]:

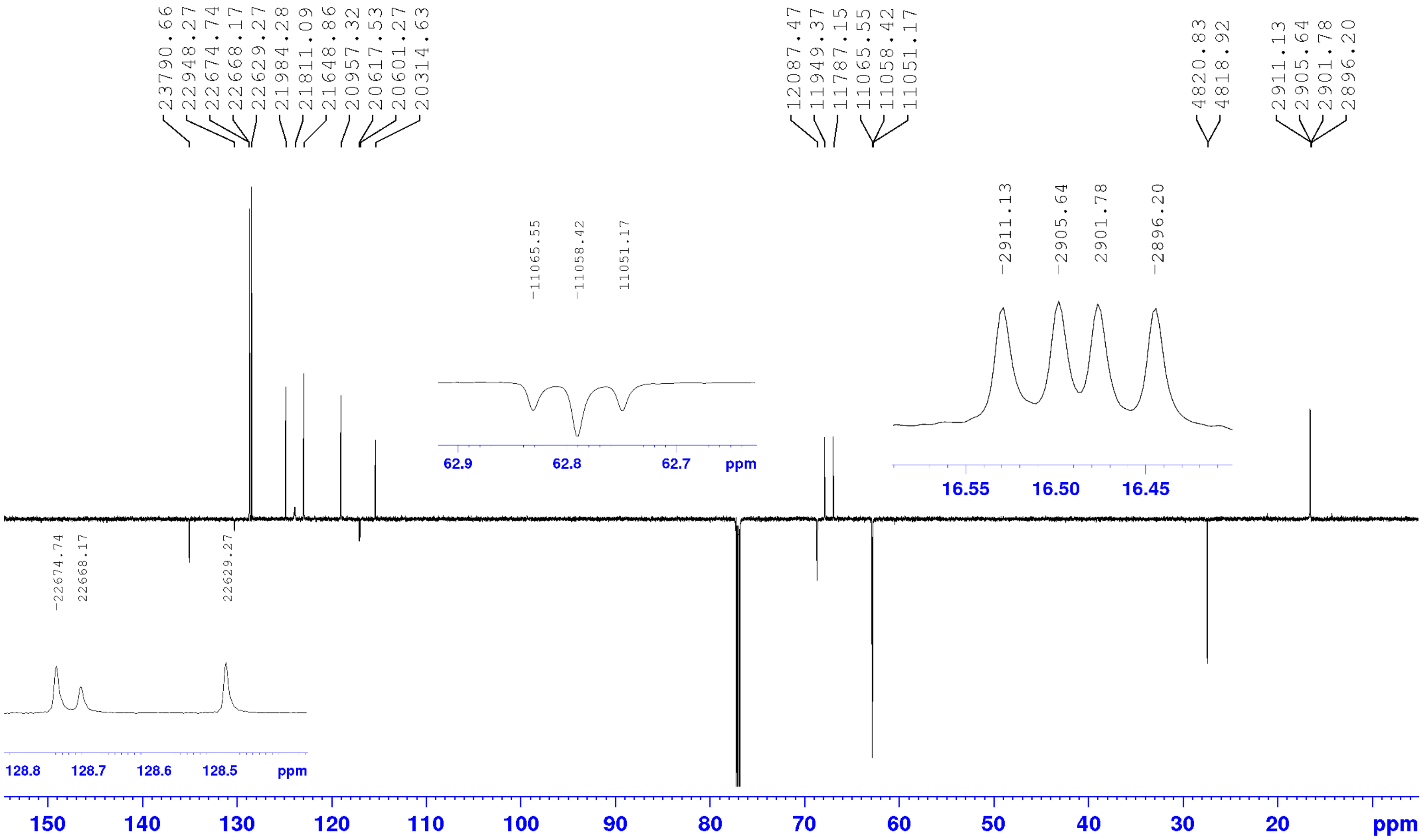

^{31}P NMR of (*S*)-diethyl (1-hydroxy-2-(1-(2-phenylacetyl)-1H-indol-2-yl)ethyl)phosphonate ($CDCl_3$, 242.97 MHz) [(*S*)-31]:

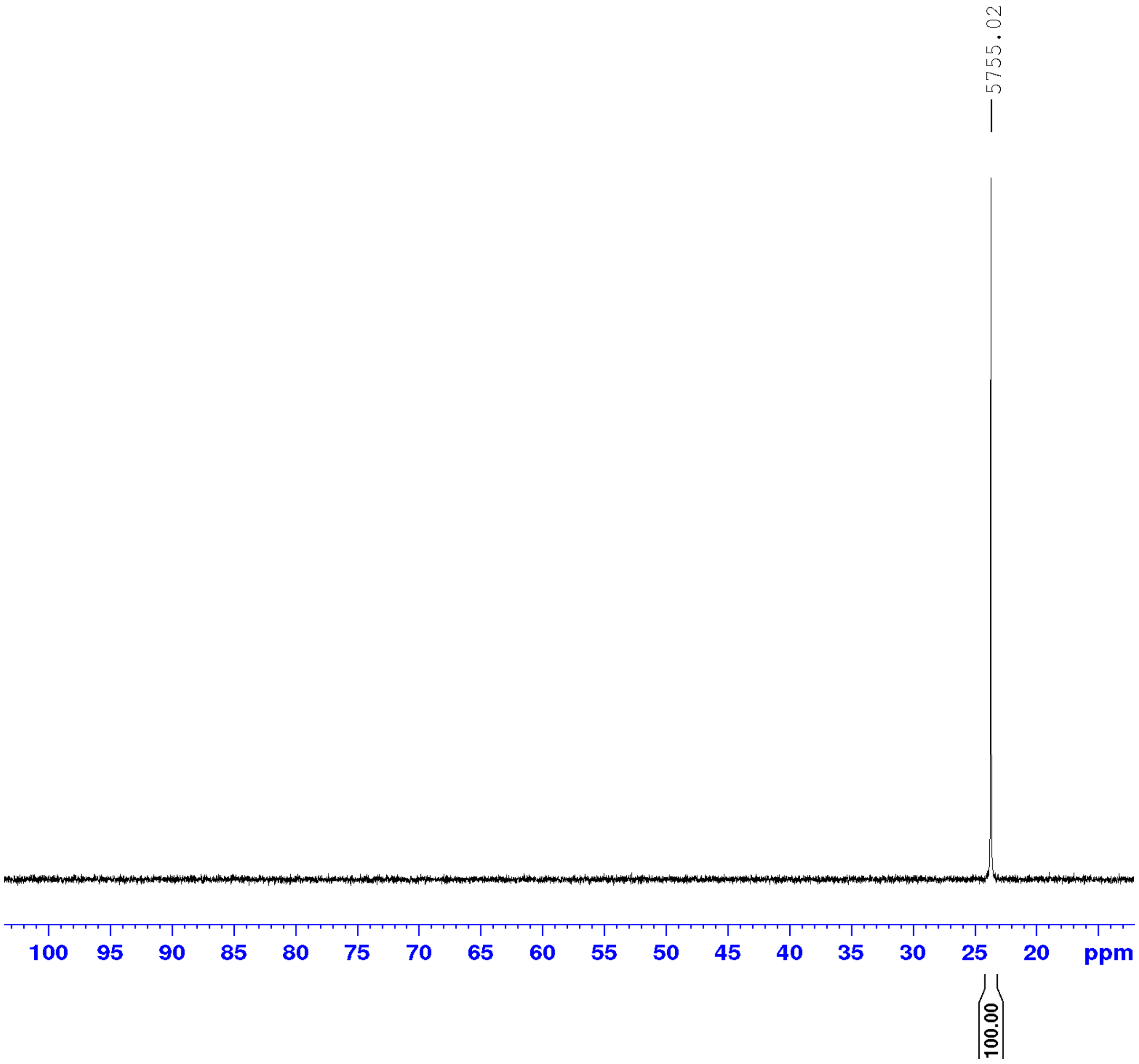

^{1}H NMR of (*R*)-diethyl (1-azido-2-(1-(2-phenylacetyl)-1H-indol-2-yl)ethyl)phosphonate ($CDCl_3$, 600.25 MHz) [(*R*)-82]:

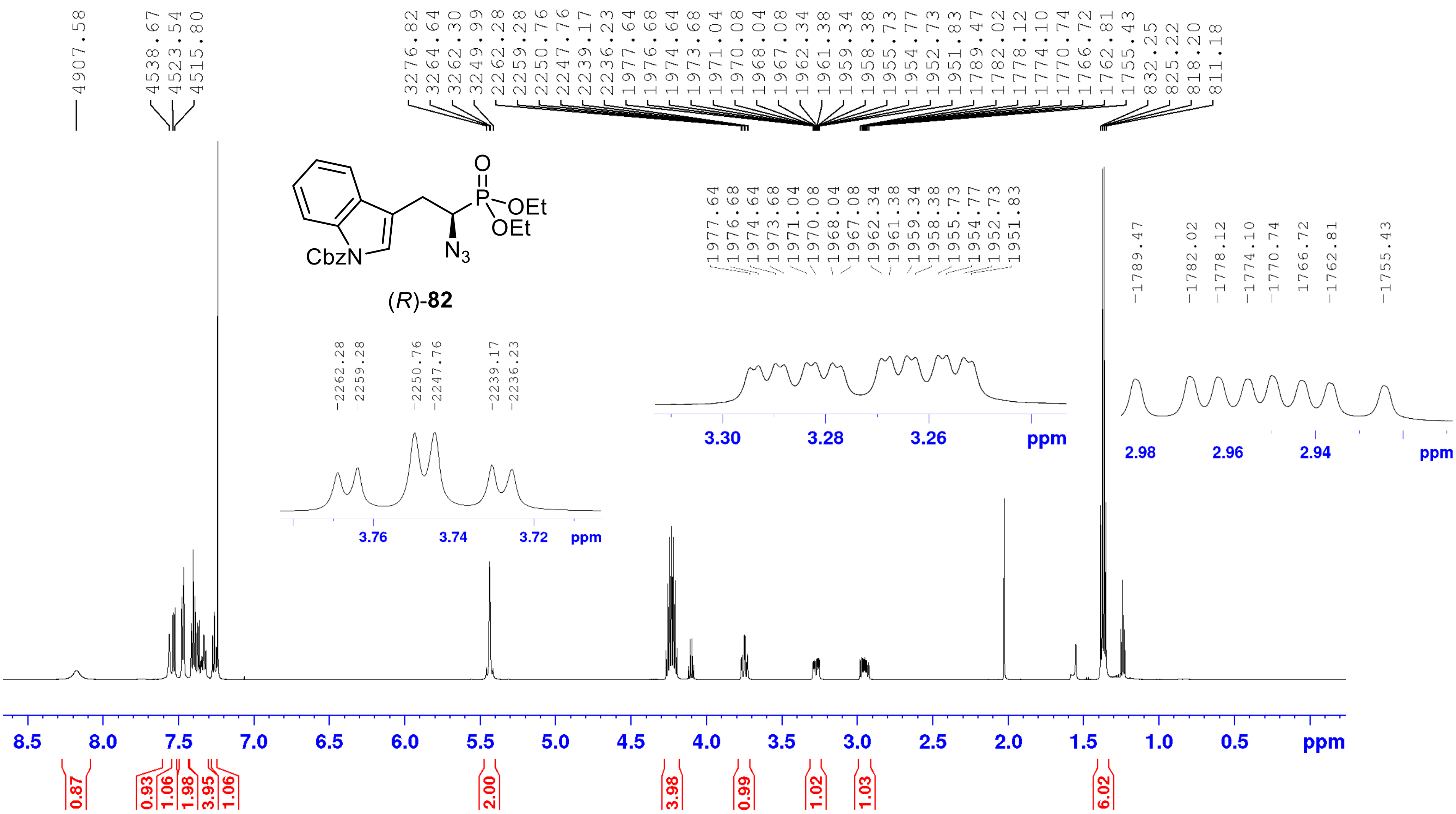

^{13}C NMR of (*R*)-diethyl (1-azido-2-(1-(2-phenylacetyl)-1H-indol-2-yl)ethyl)phosphonate ($CDCl_3$, 150.93 MHz) [(*R*)-82]:

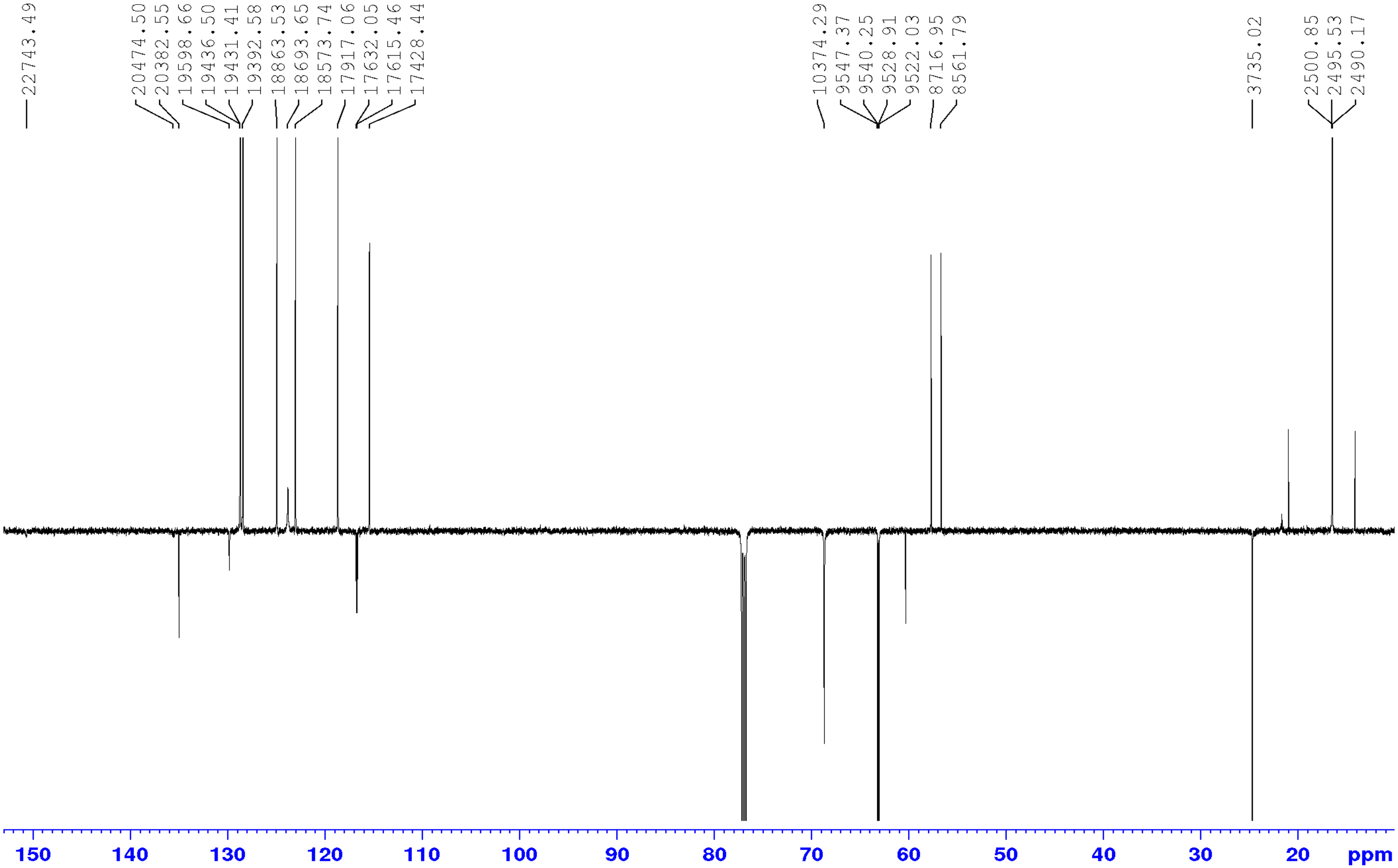

^{31}P NMR of (*R*)-diethyl (1-azido-2-(1-(2-phenylacetyl)-1H-indol-2-yl)ethyl)phosphonate ($CDCl_3$, 242.97 MHz) [(*R*)-82]:

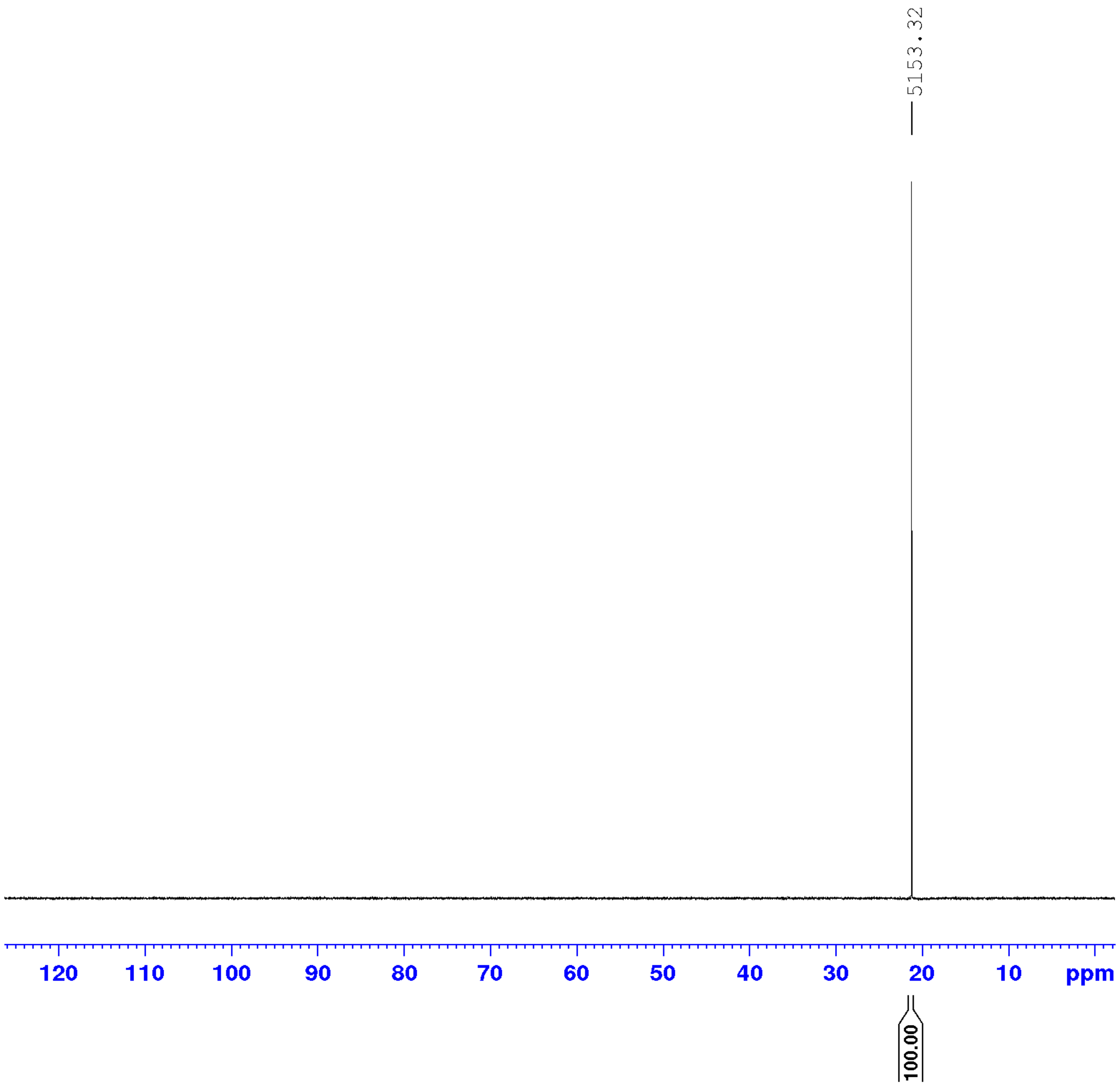

^{1}H NMR of (*R*)-(1-amino-2-(1H-indol-2-yl)ethyl)phosphonic acid, (*R*)-phosphatryptophane (D_2O, 600.25 MHz) [(*R*)-65]:

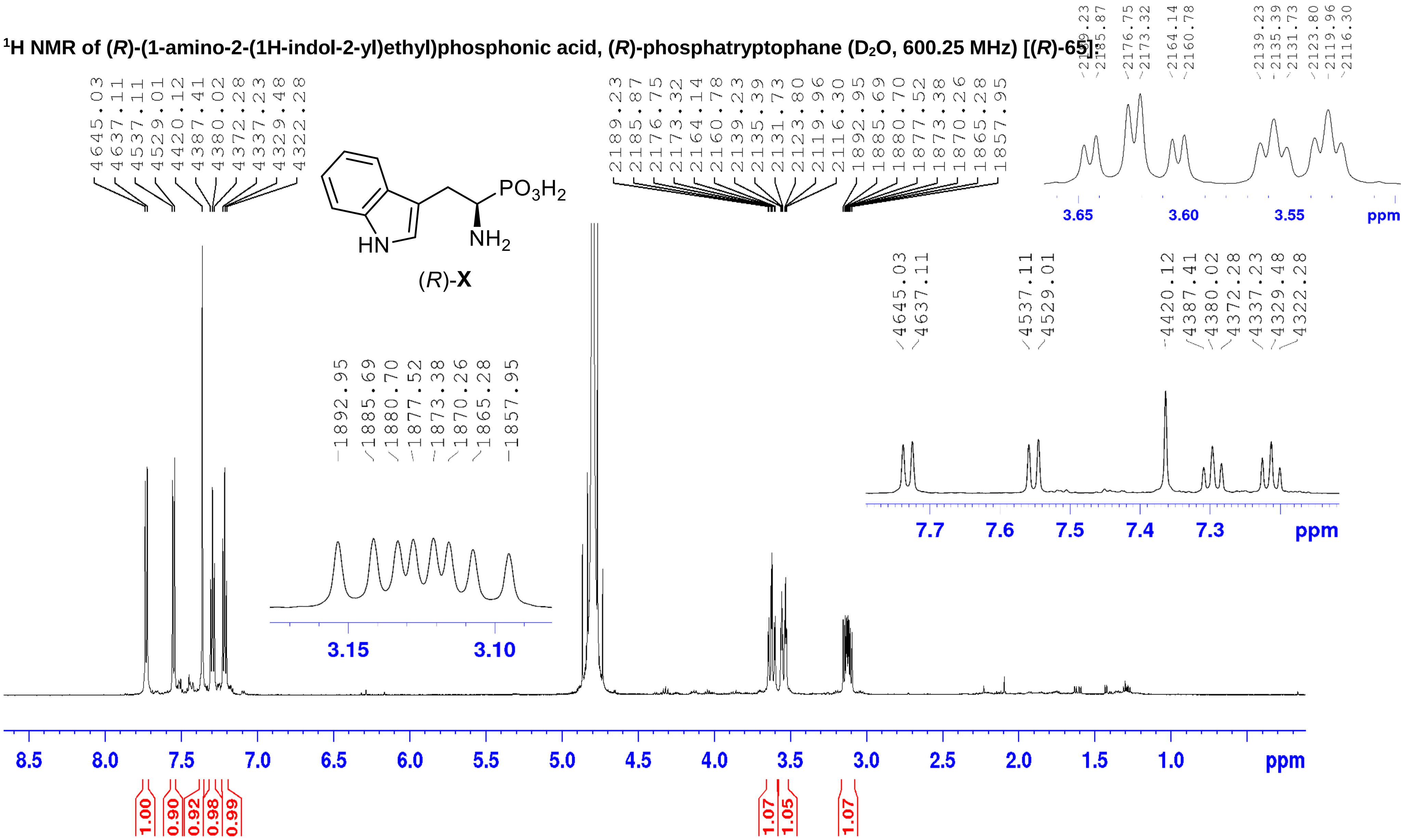

^{13}C NMR of (*R*)-(1-amino-2-(1H-indol-2-yl)ethyl)phosphonic acid, (*R*)-Phosphatryptophane [(*R*)-65] (D_2O, 150.94 MHz):

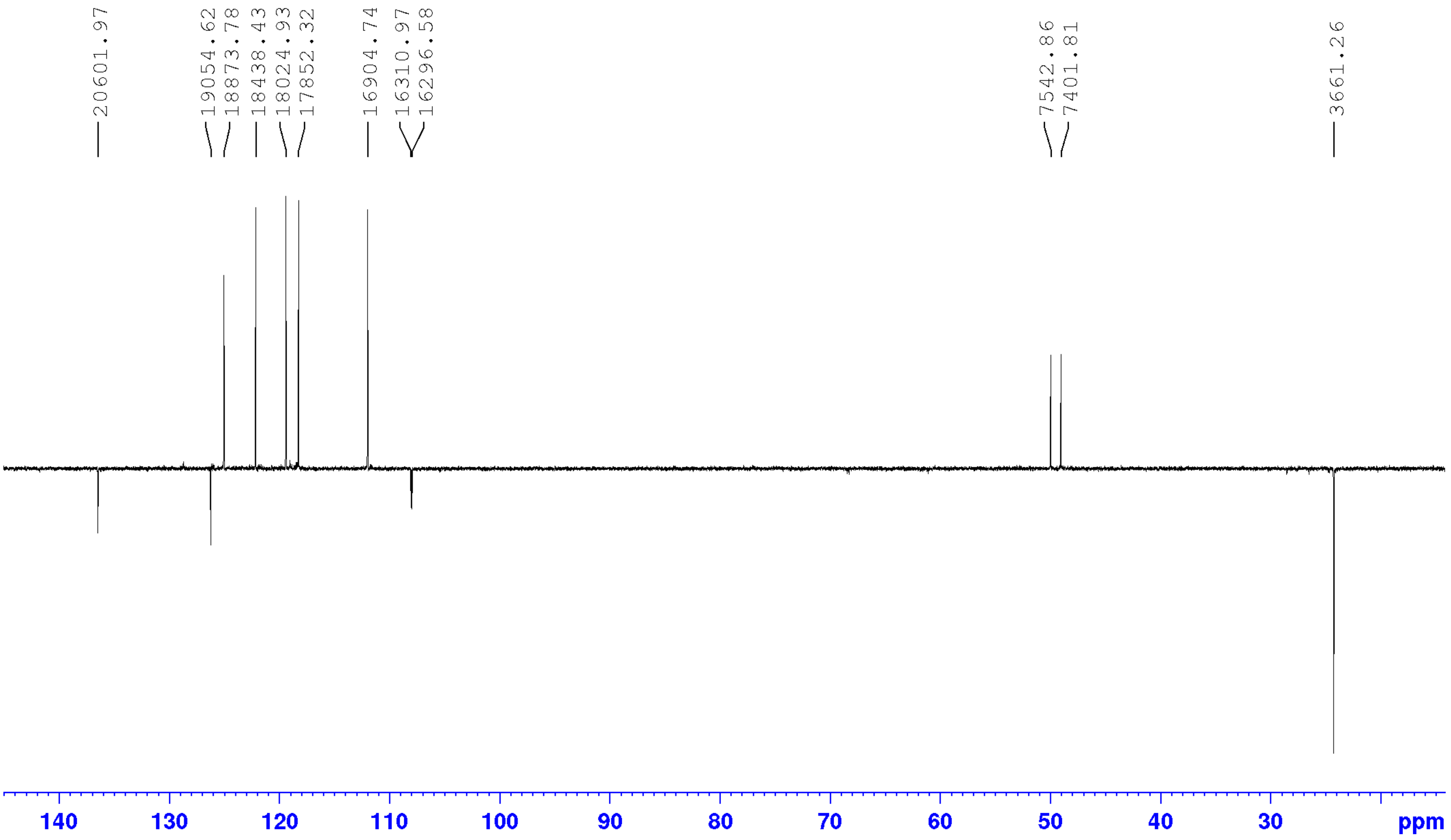

^{31}P NMR of (*R*)-(1-amino-2-(1H-indol-2-yl)ethyl)phosphonic acid, (*R*)-Phosphatryptophane [(*R*)-65] (D_2O, 242.97 MHz):

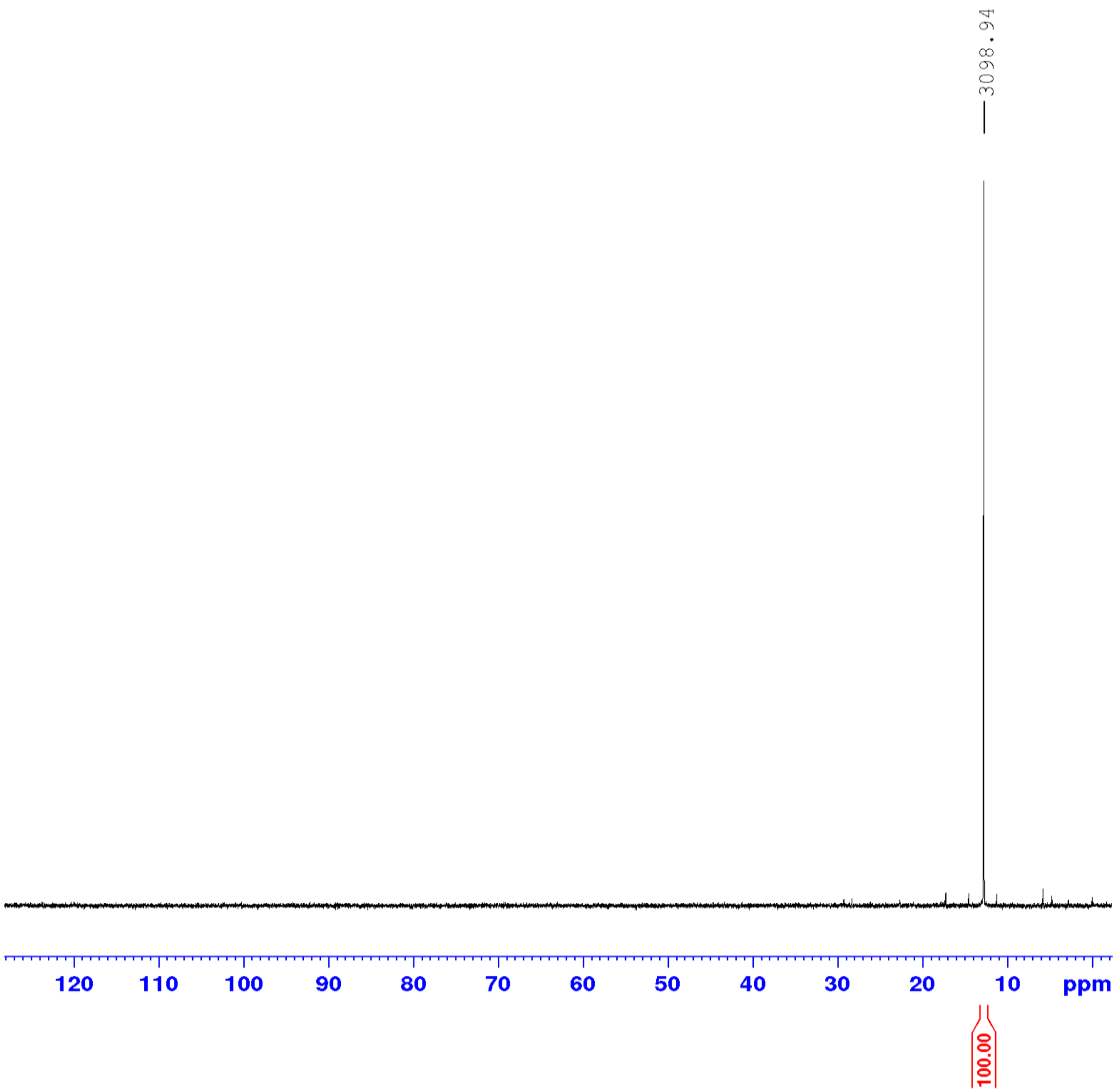

^{31}P NMR of diisopropyl 1-oxo-2-(4-methoxyphenyl)-ethylphosphonate (162.03 MHz, $CDCl_3$) (15):

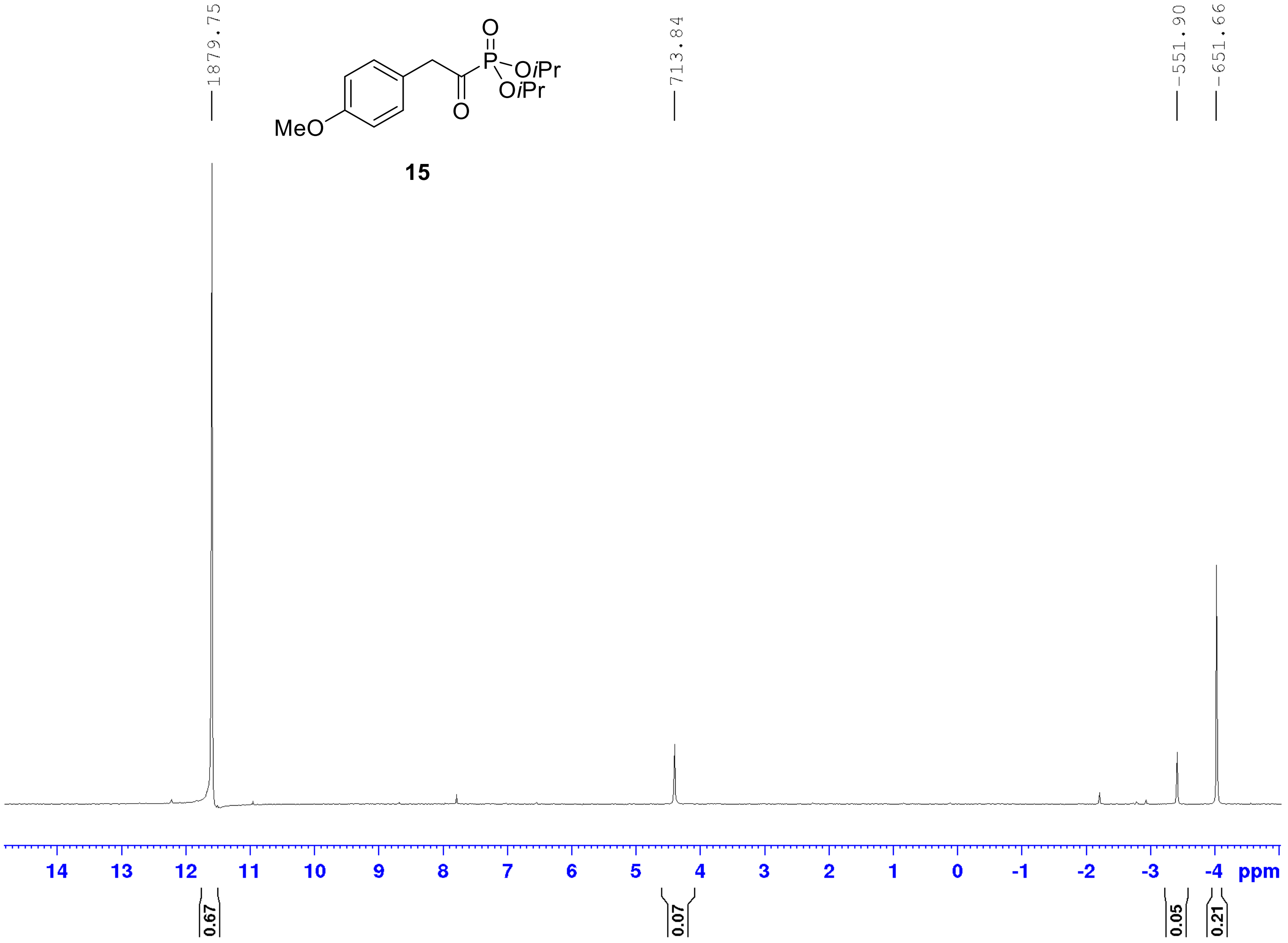

^{1}H NMR of (*S*)-diisopropyl 1-hydroxy-2-(4-methoxyphenyl)-ethylphosphonate (400.27 MHz, $CDCl_3$) [(*S*)-32]:

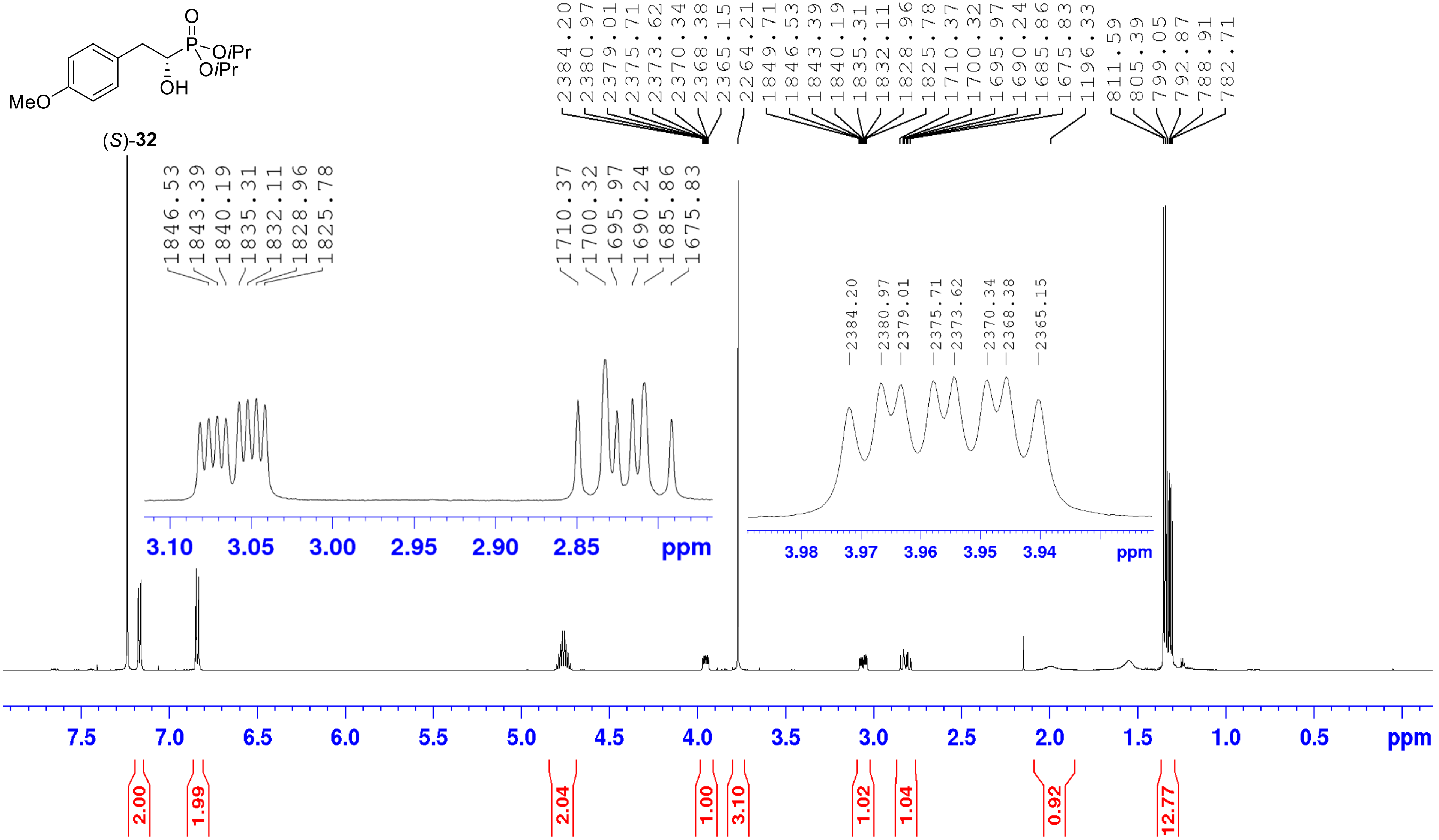

(*S*)-**32**

^{13}C NMR of (*S*)-diisopropyl 1-hydroxy-2-(4-methoxyphenyl)-ethylphosphonate (150.93 MHz, $CDCl_3$) [(*S*)-32]:

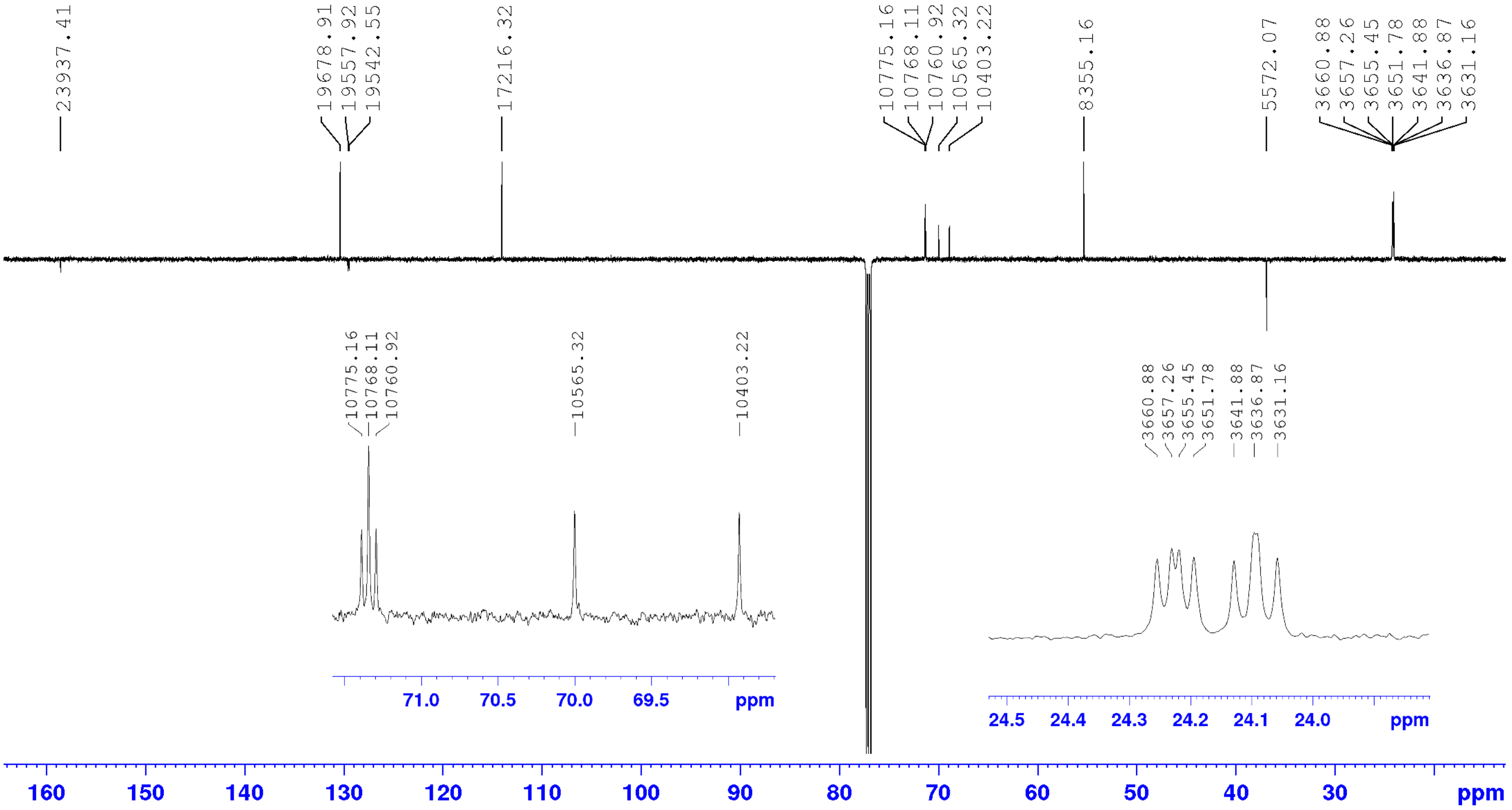

^{31}P NMR of (*S*)-diisopropyl 1-hydroxy-2-(4-methoxyphenyl)-ethylphosphonate (162.03 MHz, $CDCl_3$) [(*S*)-32]:

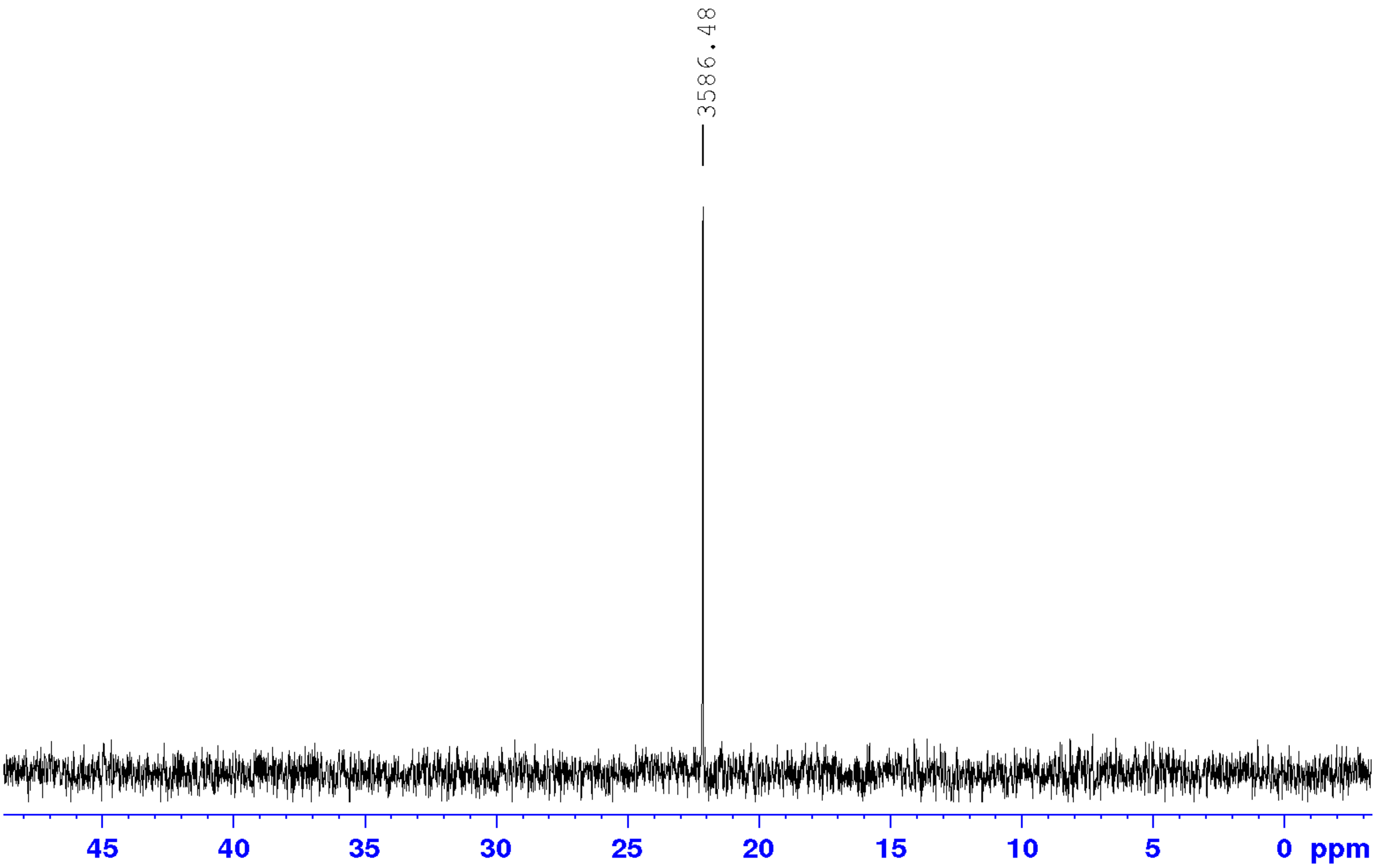

^{1}H NMR of (*R*)-diisopropyl 1-azido-2-(4-methoxyphenyl)-ethylphosphonate (400.27 MHz, $CDCl_3$) [(*R*)-83]:

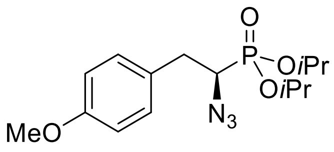

(*R*)-**83**

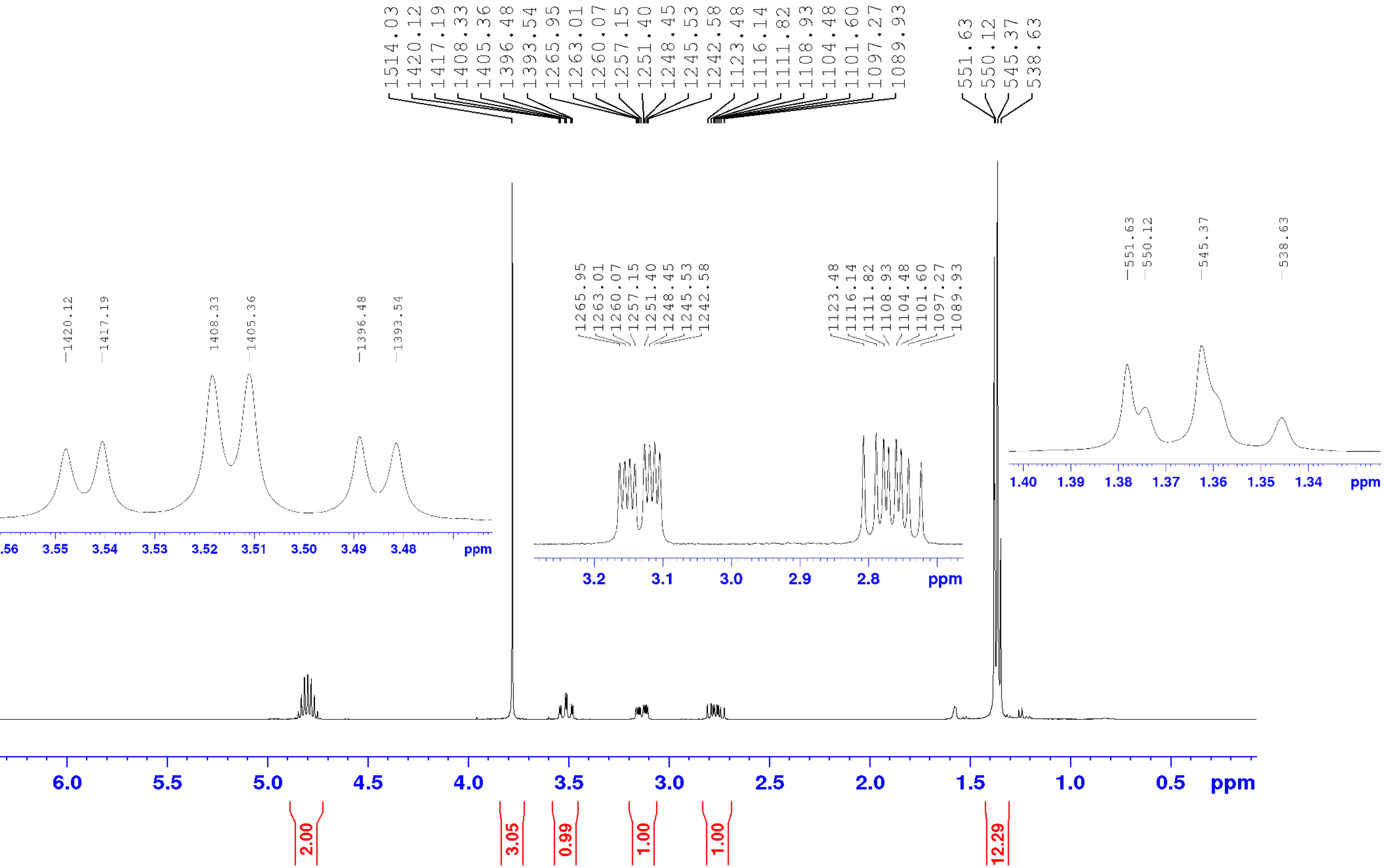

^{13}C NMR of (*R*)-diisopropyl 1-azido-2-(4-methoxyphenyl)-ethylphosphonate (150.93 MHz, $CDCl_3$) [(*R*)-83]:

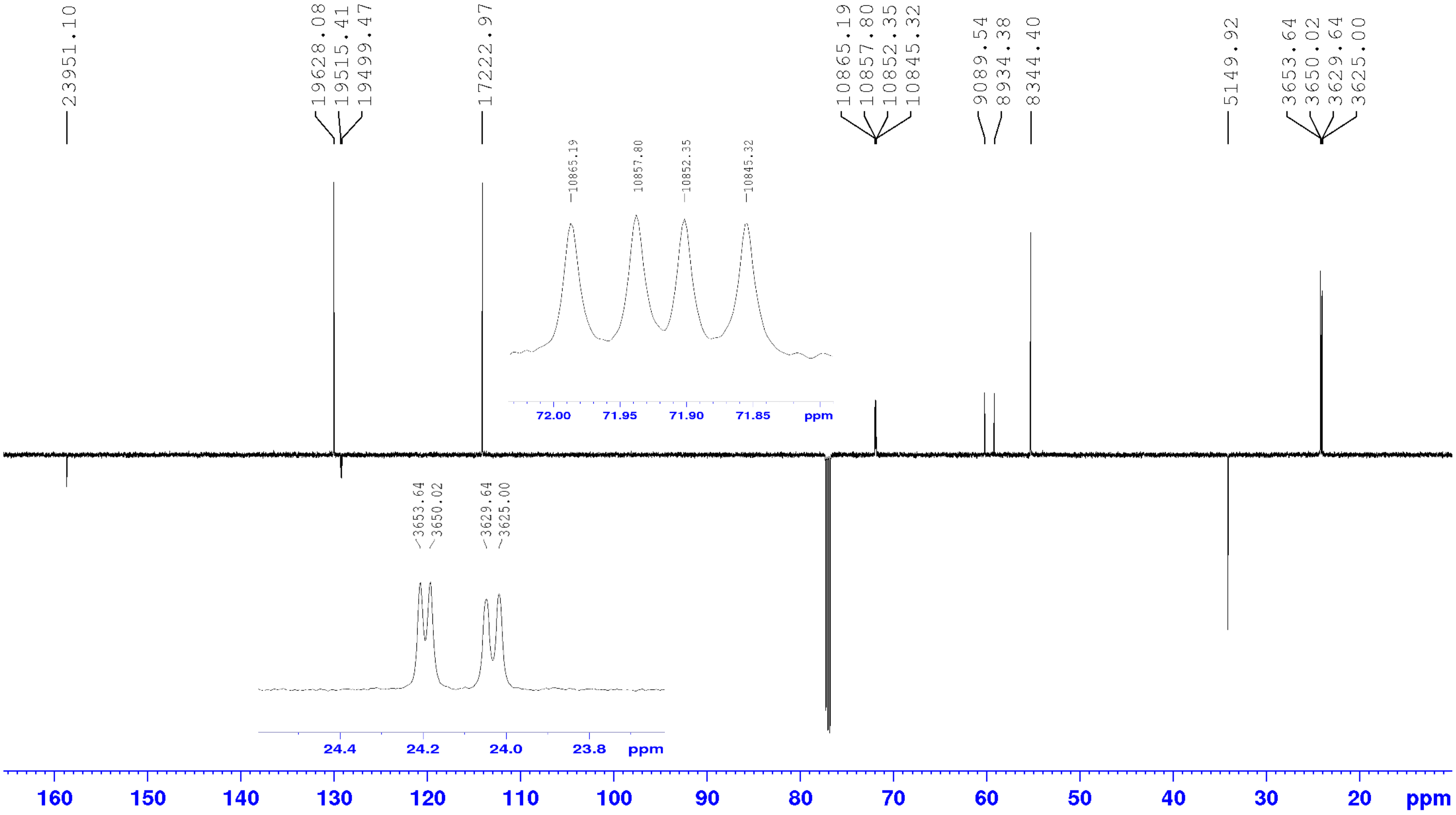

^{31}P NMR of *R*)-diisopropyl 1-azido-2-(4-methoxyphenyl)-ethylphosphonate (162.03 MHz, $CDCl_3$) [(*R*)-83]:

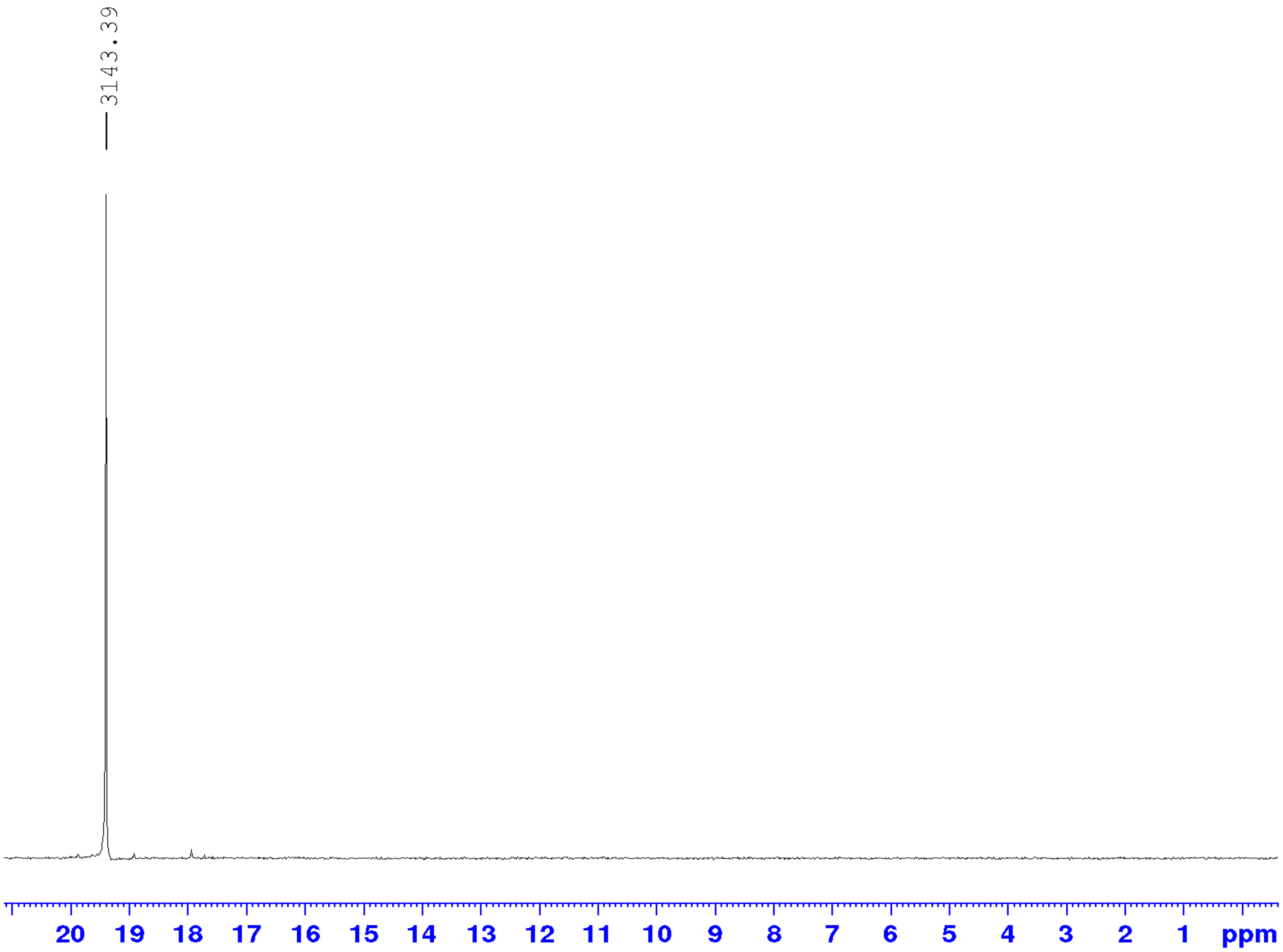

^{1}H NMR of (*R*)-1-amino-2-(hydroxyphenyl)-ethylphosphonic acid, (*R*)-phosphatyrosine (400.27 MHz, D_2O) [(*R*)-66]:

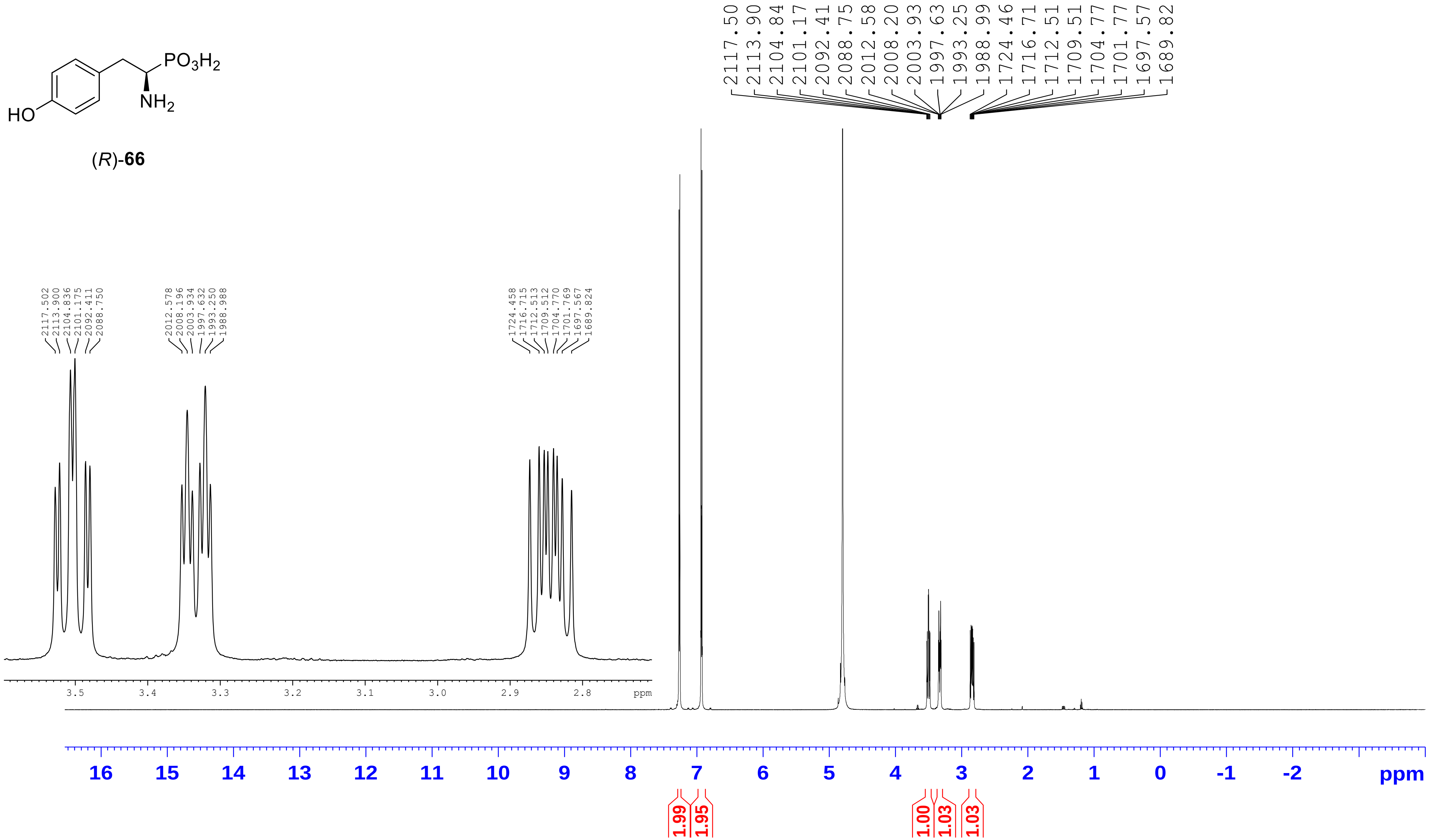

^{13}C NMR of (*R*)-1-amino-2-(hydroxyphenyl)-ethylphosphonic acid, (*R*)-phosphatyrosine (150.93 MHz, D_2O) [(*R*)-66]:

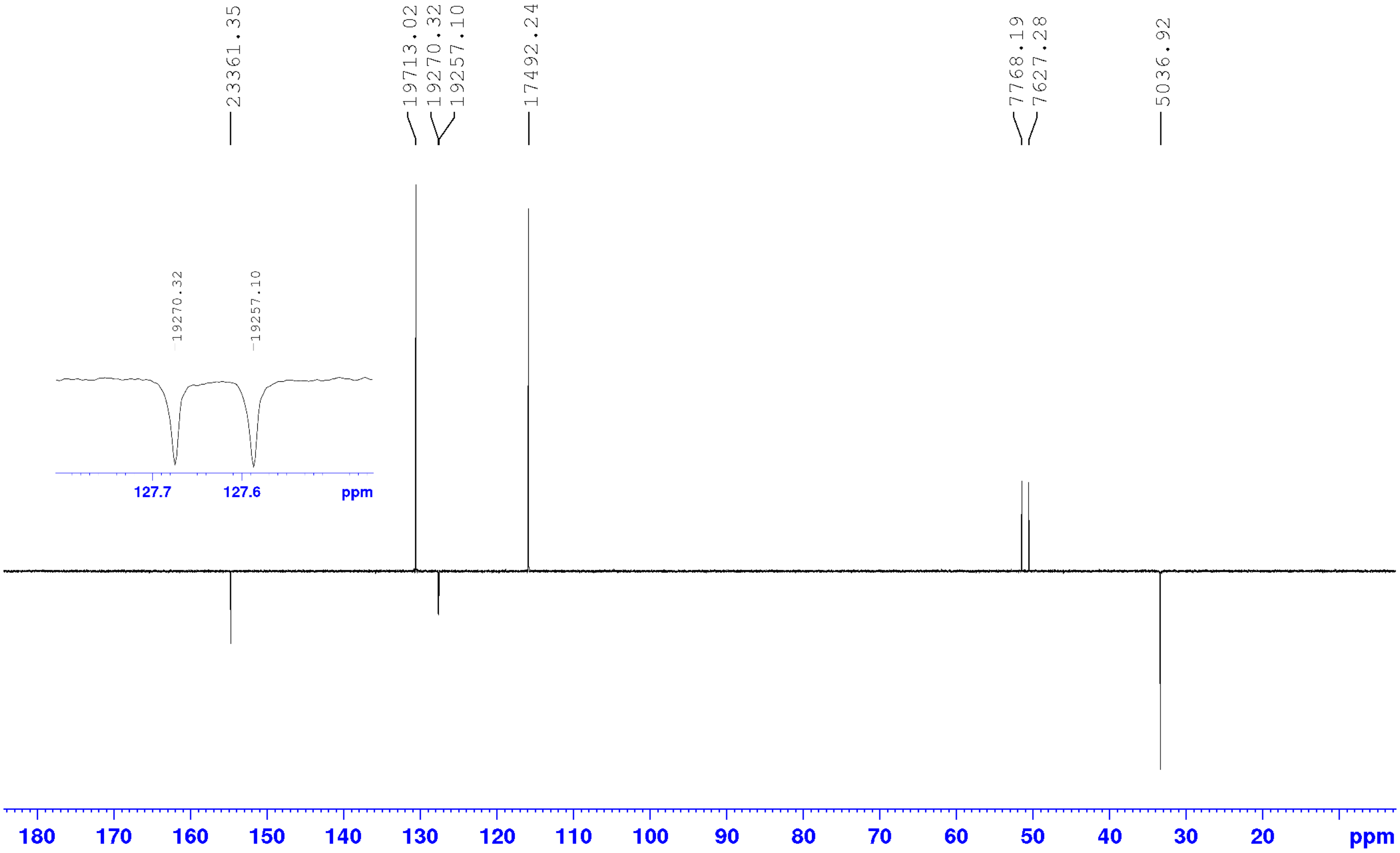

^{31}P NMR of (*R*)-1-amino-2-(hydroxyphenyl)-ethylphosphonic acid, (*R*)-phosphatyrosine (162.03 MHz, D_2O) [(*R*)-66]:

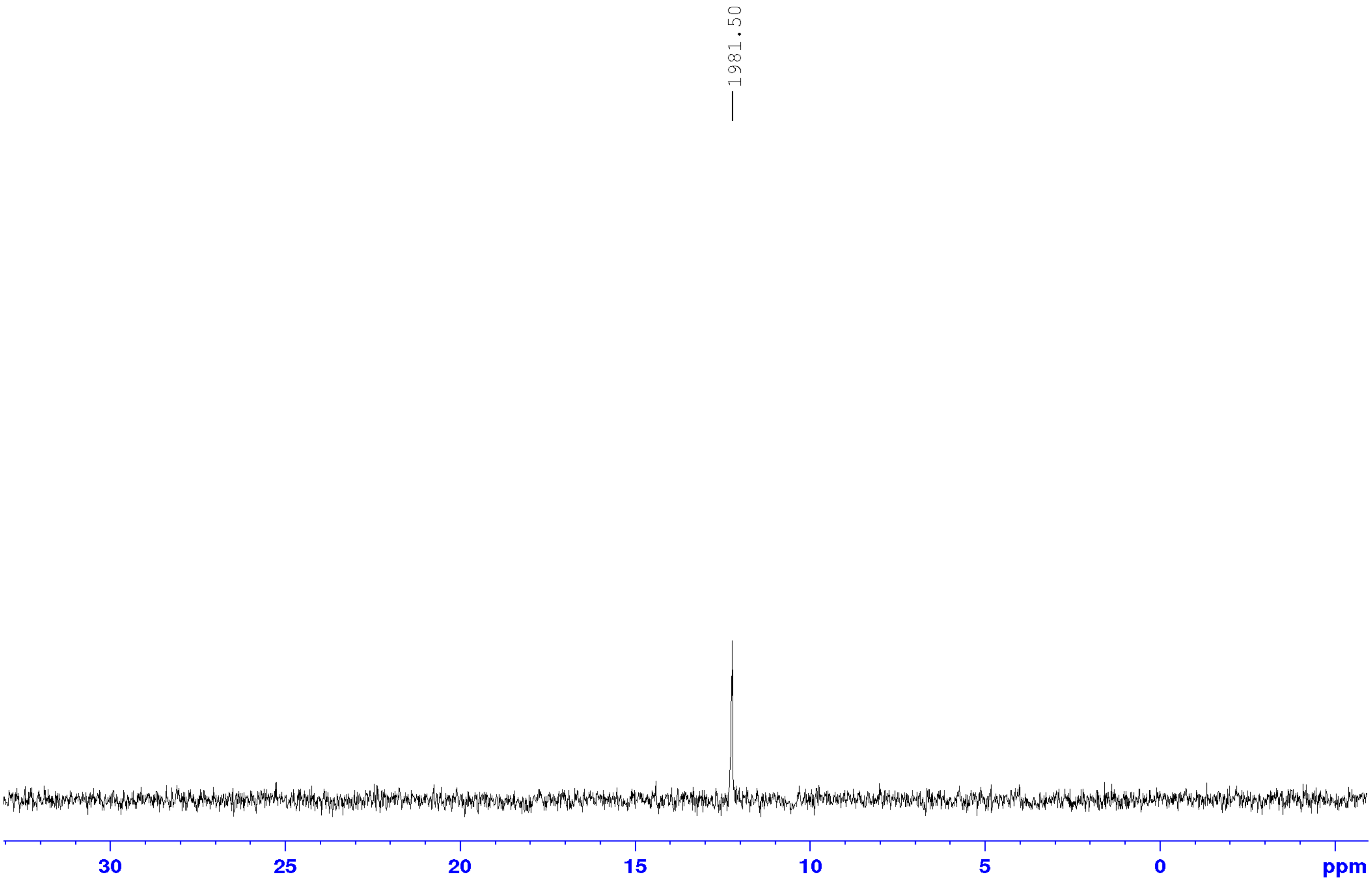

ee-determination of (R)-66 by chiral stationary phase HPLC after derivatization as shown below

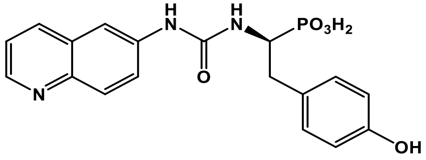

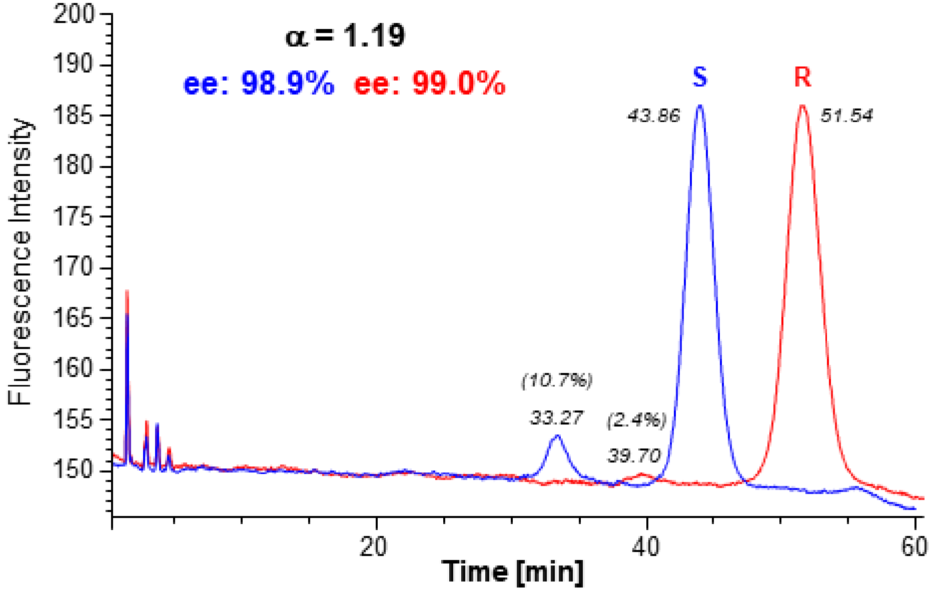

Chiral Separation of AQC derivatized phosphatyrosine (*R*)-**66** and (*S*)-**66** on an unmodified OH-QD-AX column (150 mm x 4 mm, 5 µm) using 2 M aqueous H_3PO_4:MeOH (1:9, (v/v)) with pH 4.0 (adjusted with trimethylamine) as mobile phase at a flow rate of 1 mL/min, a column temperature of 40°C and fluorescence detection (ex. 250 nm ; em .395 nm). Note that the impurity peaks at 33.27 min and 39.70 min are most probably methoxylated phosphatyrosine, since their retention times are similar to that of phospha-phenylalanine.

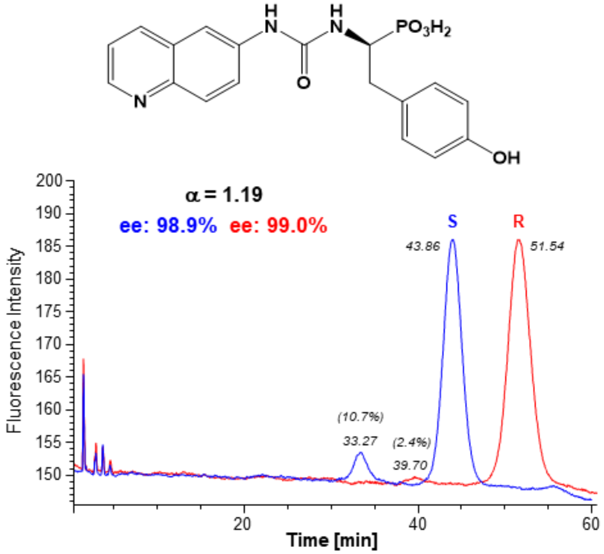

^{1}H NMR of diisopropyl 2-(benzyloxy)-1-oxoethylphosphonate (400.27 MHz, $CDCl_3$) (16):

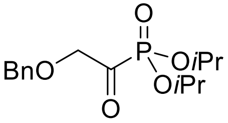

16

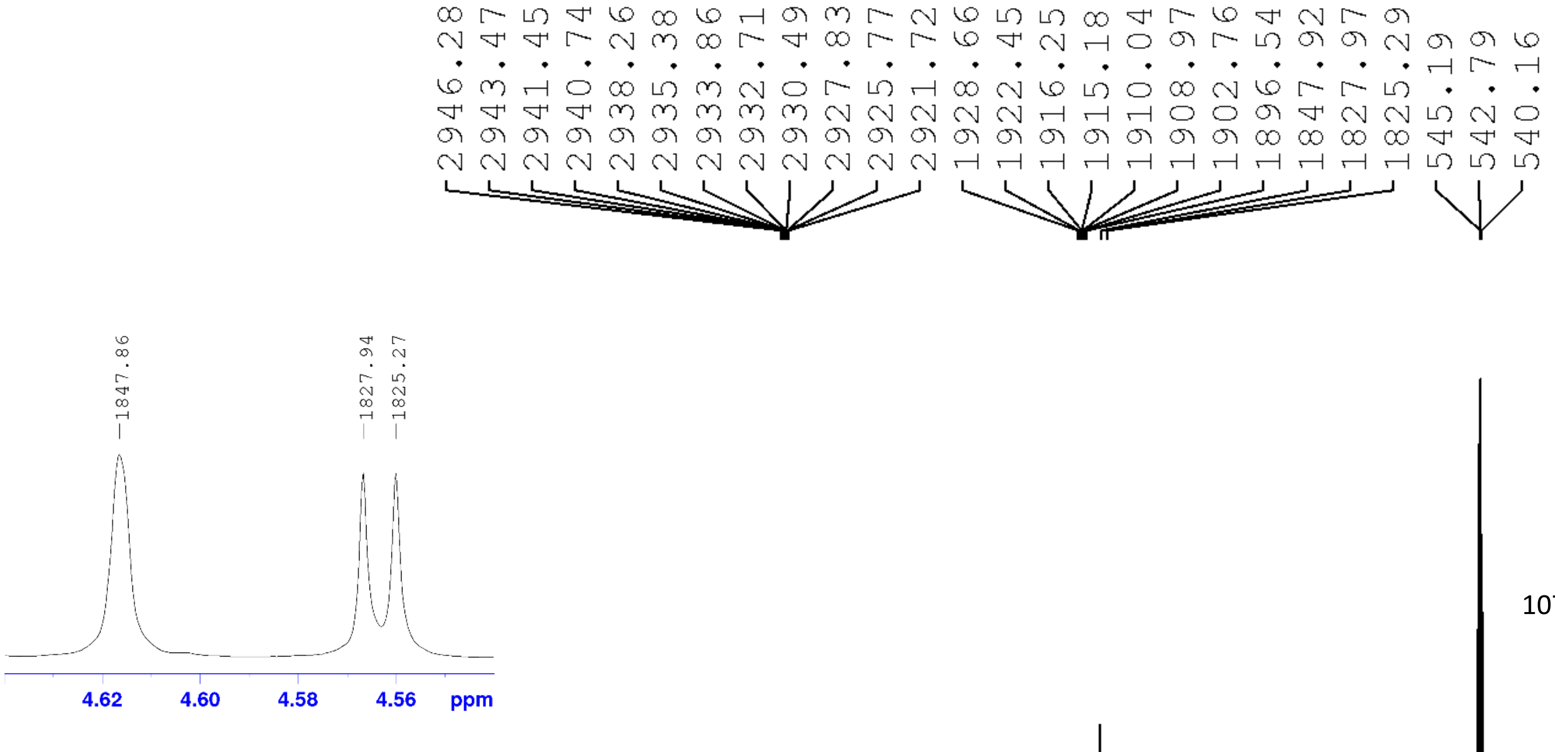

^{31}P NMR of diisopropyl 2-(benzyloxy)-1-oxoethylphosphonate (162.03 MHz, $CDCl_3$) (16):

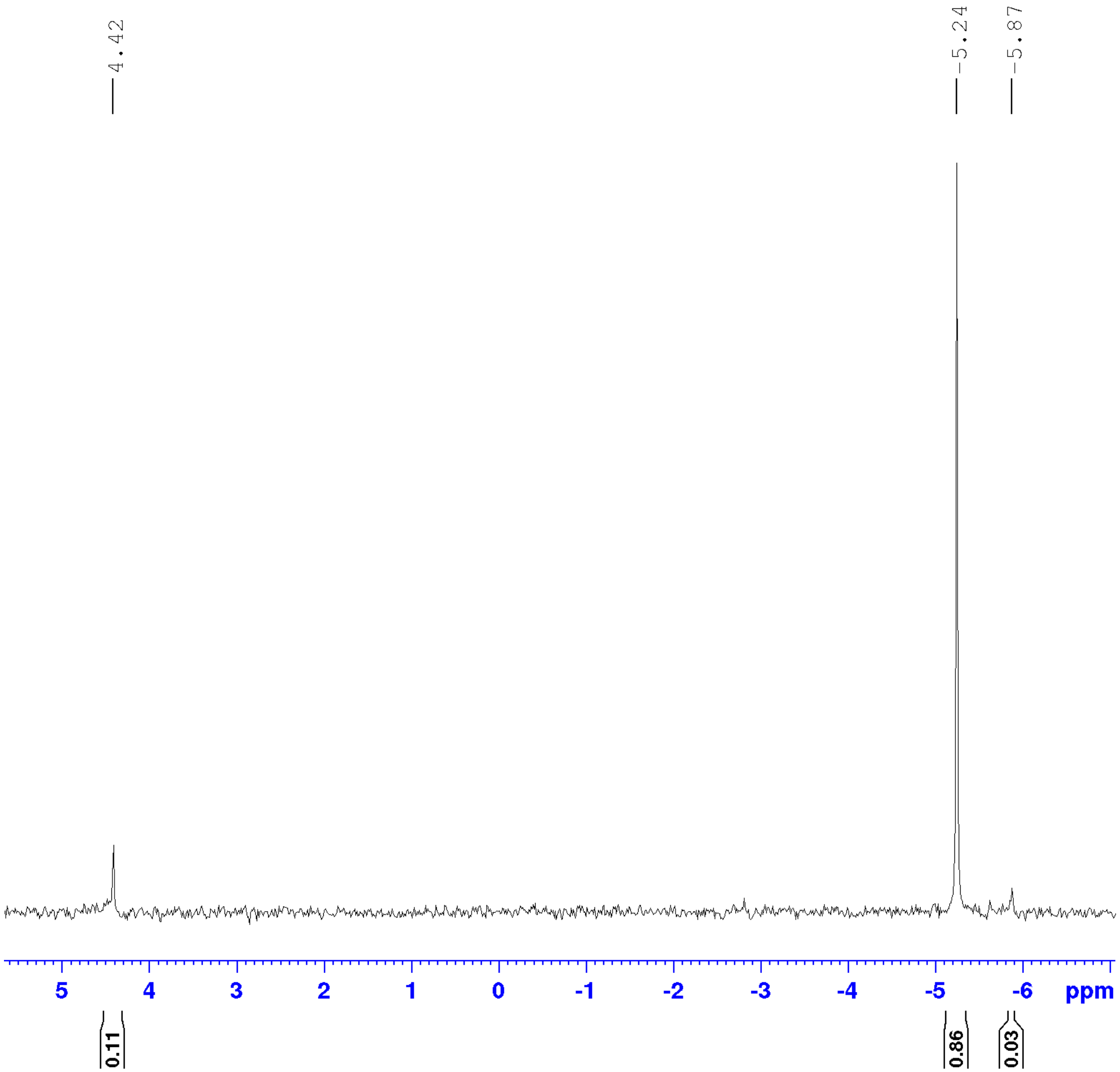

^{1}H NMR of (*S*)- diisopropyl (2-(benzyloxy)-1-hydroxyethyl)phosphonate (600.25 MHz, $CDCl_3$) [(*S*)-33]:

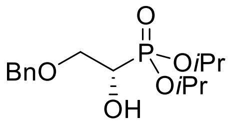

(*S*)-**33**

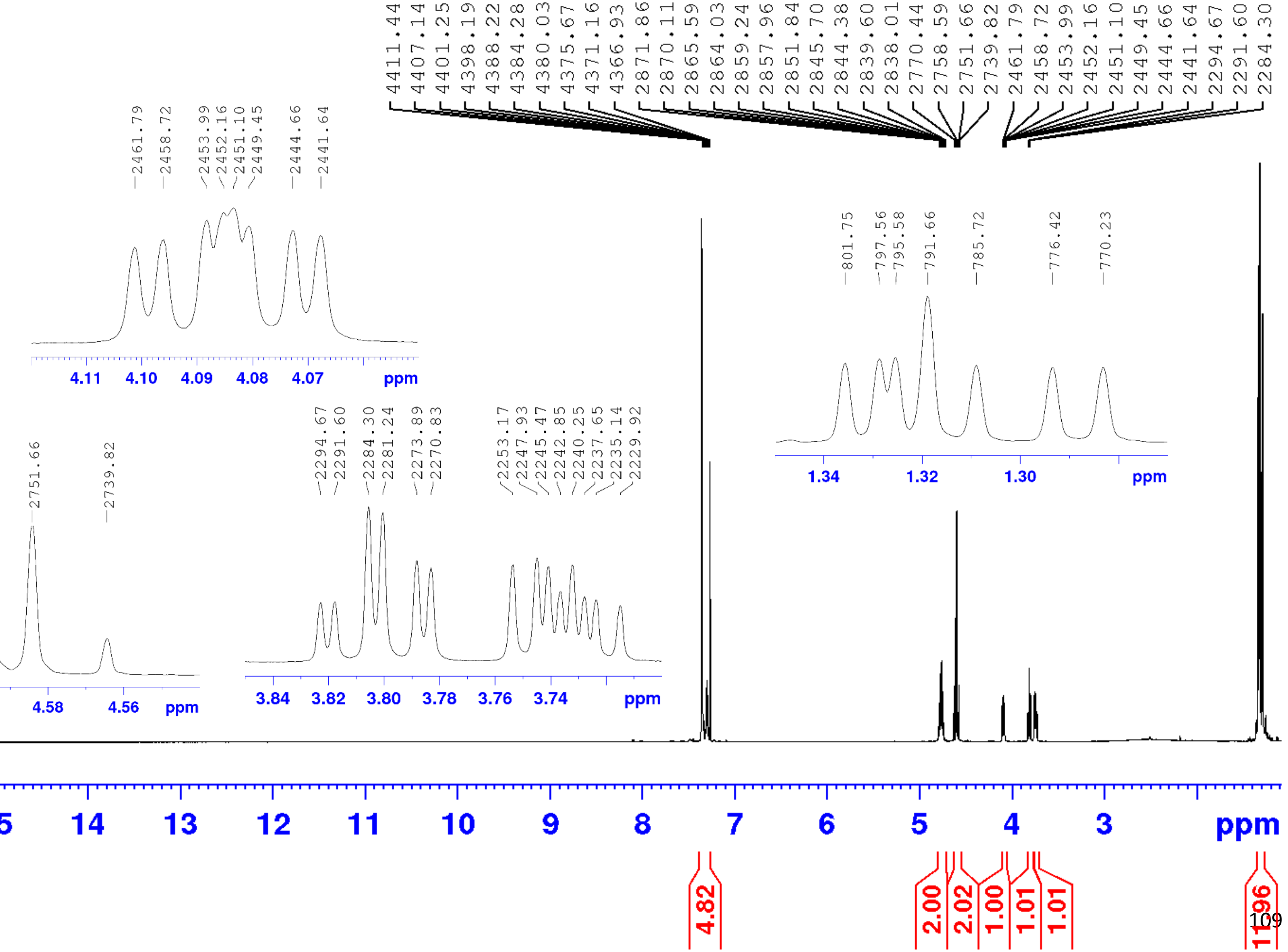

^{13}C NMR of (*S*)- diisopropyl (2-(benzyloxy)-1-hydroxyethyl)phosphonate (150.93 MHz, $CDCl_3$) [(*S*)-33]:

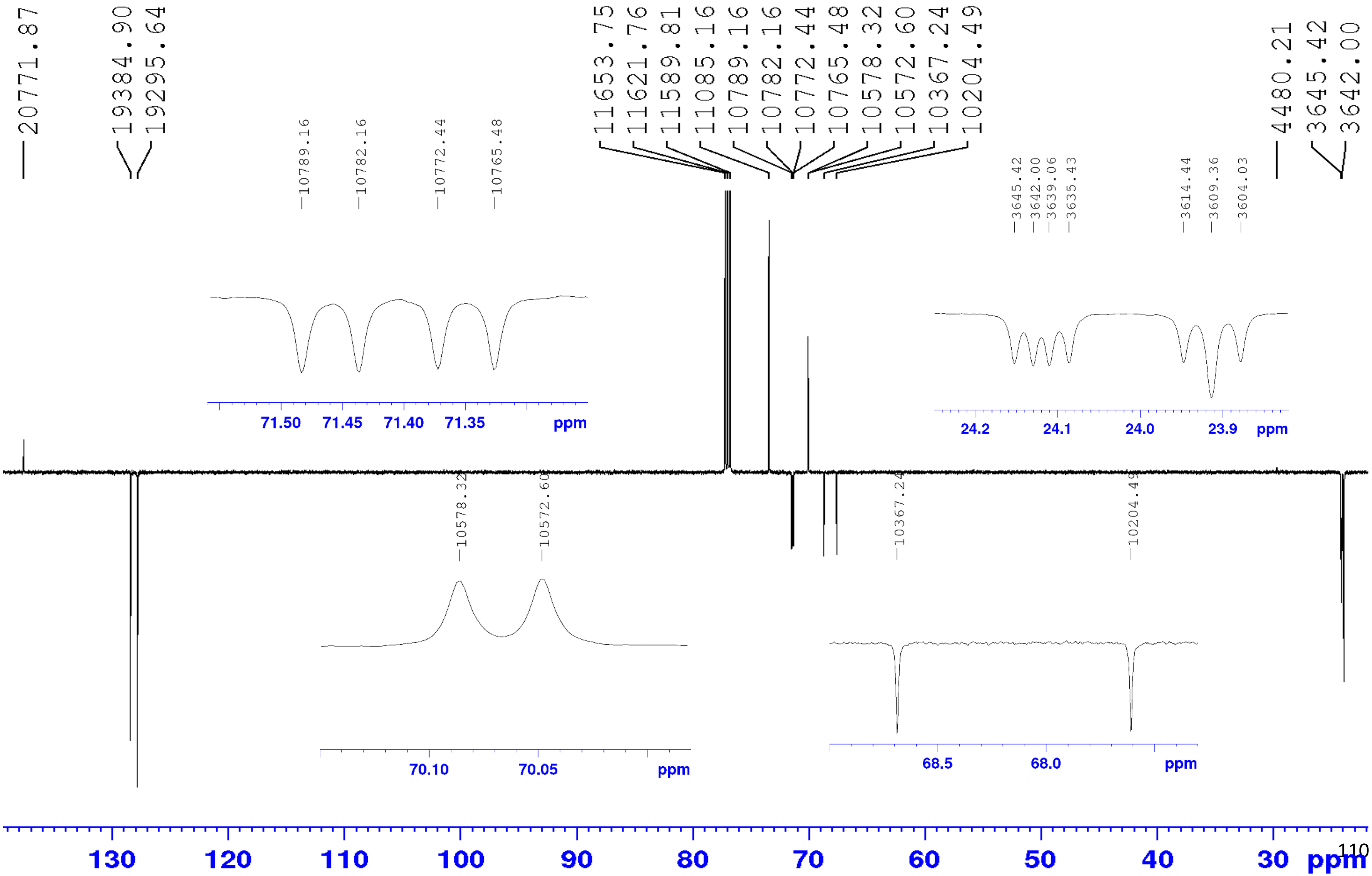

^{31}P NMR of (*S*)- diisopropyl (2-(benzyloxy)-1-hydroxyethyl)phosphonate (162.03 MHz, $CDCl_3$) [(*S*)-33]:

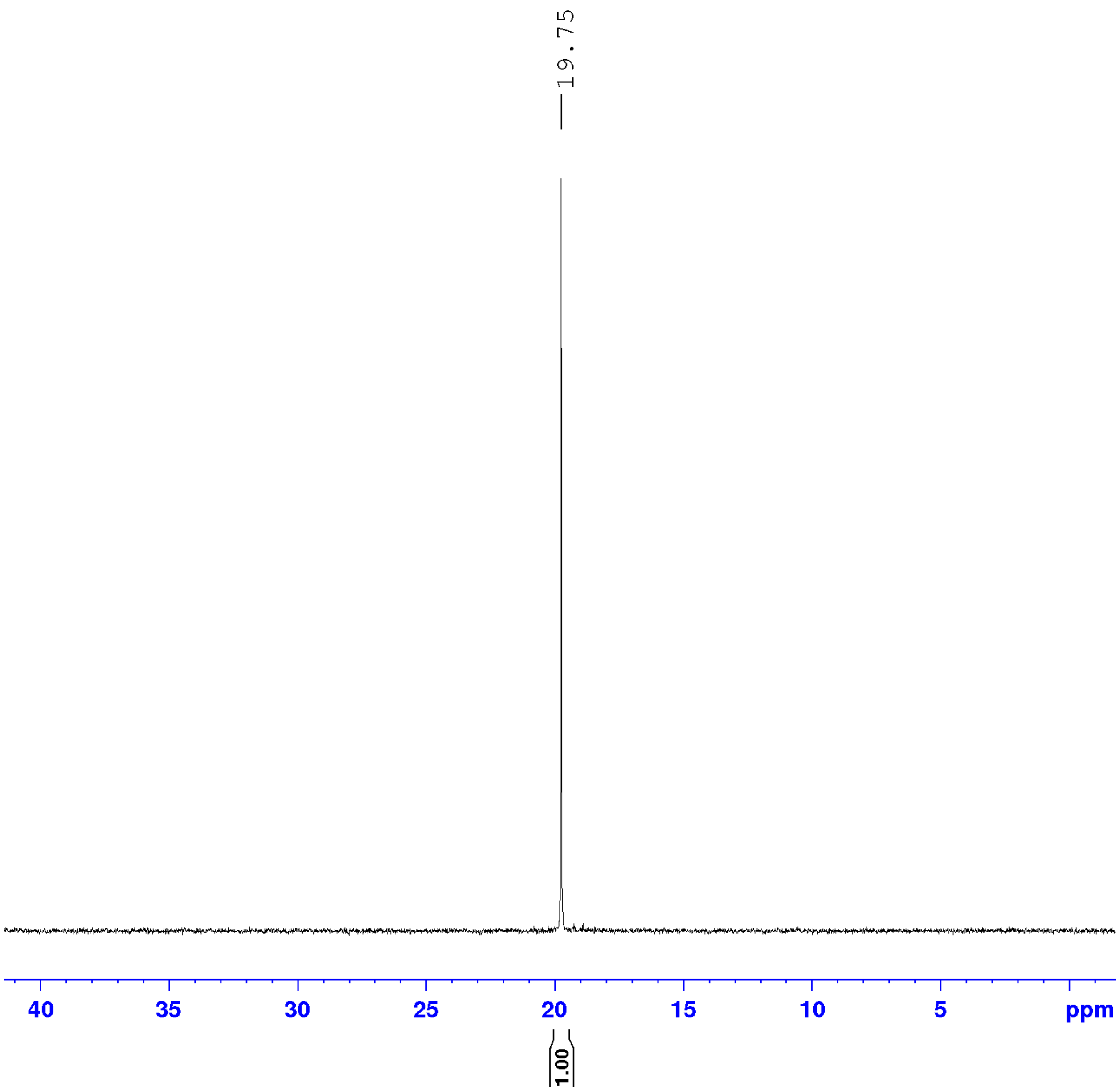

^{1}H NMR of (*R*)-1-[^{2}H]-diisopropyl (2-(benzyloxy)-1-hydroxyethyl)phosphonate (700.40 MHz, $CDCl_3$) {(*R*)-[^{2}H]-33}:

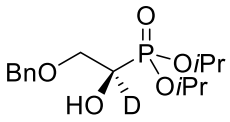

(*R*)-1-[^{2}H]-**33**

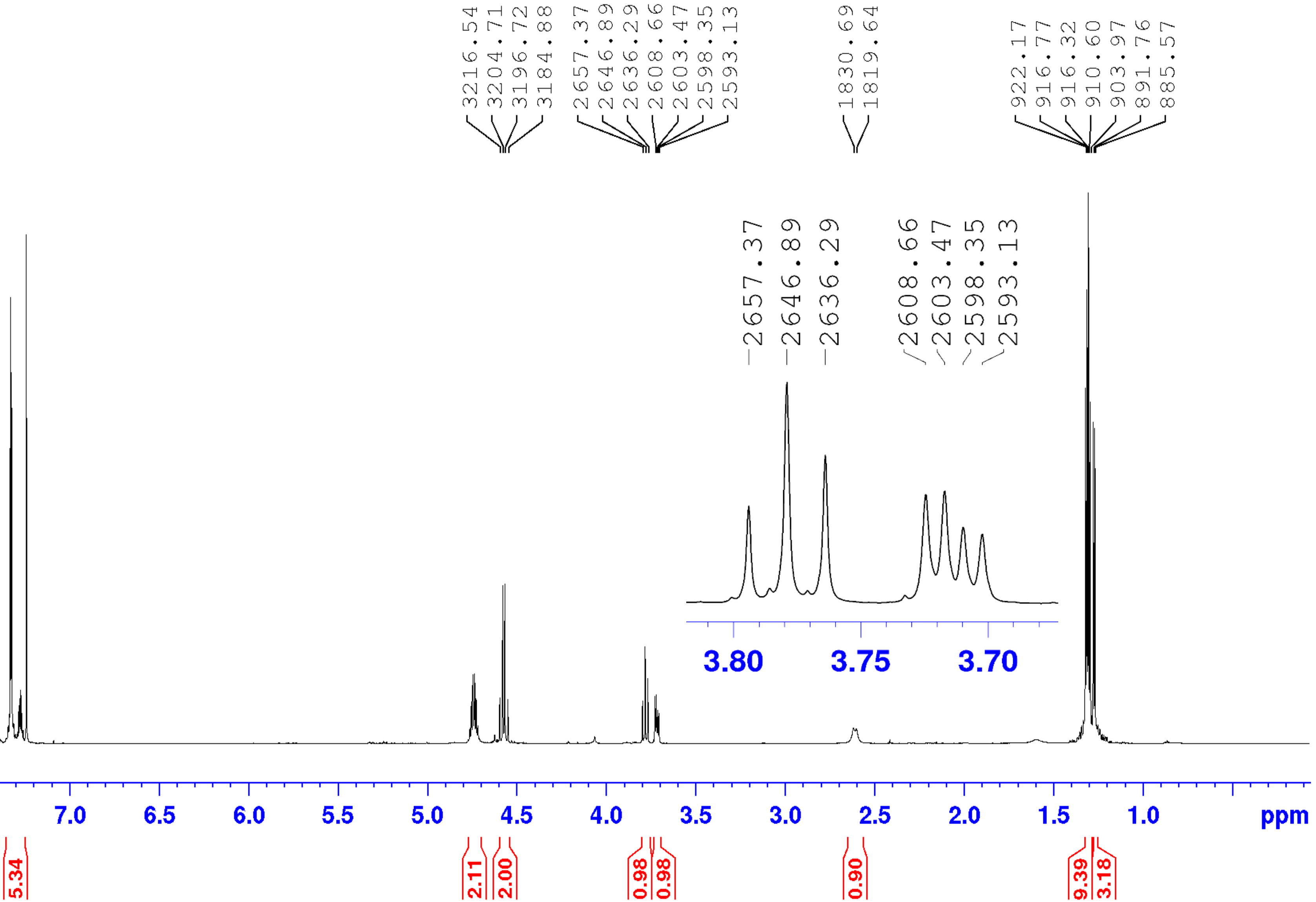

^{31}P NMR of (*R*)-1-[^{2}H]-diisopropyl (2-(benzyloxy)-1-hydroxyethyl)phosphonate (162.03 MHz, $CDCl_3$) {(*R*)-[^{2}H]-33}:

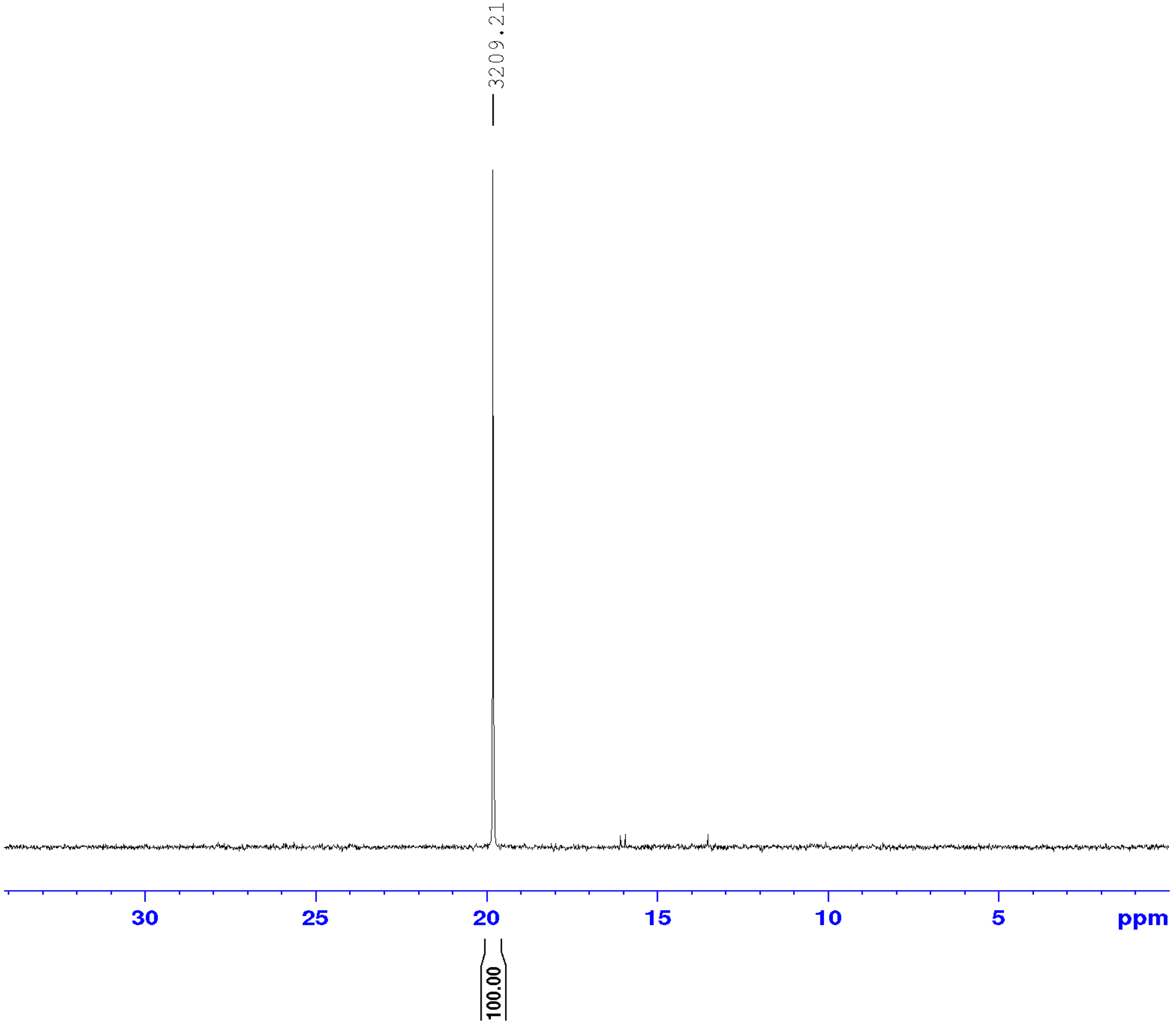

^{13}C NMR of (*R*)-1-[^{2}H]-diisopropyl (2-(benzyloxy)-1-hydroxyethyl)phosphonate (100.65 MHz, $CDCl_3$) {(*R*)-[^{2}H]-33}:

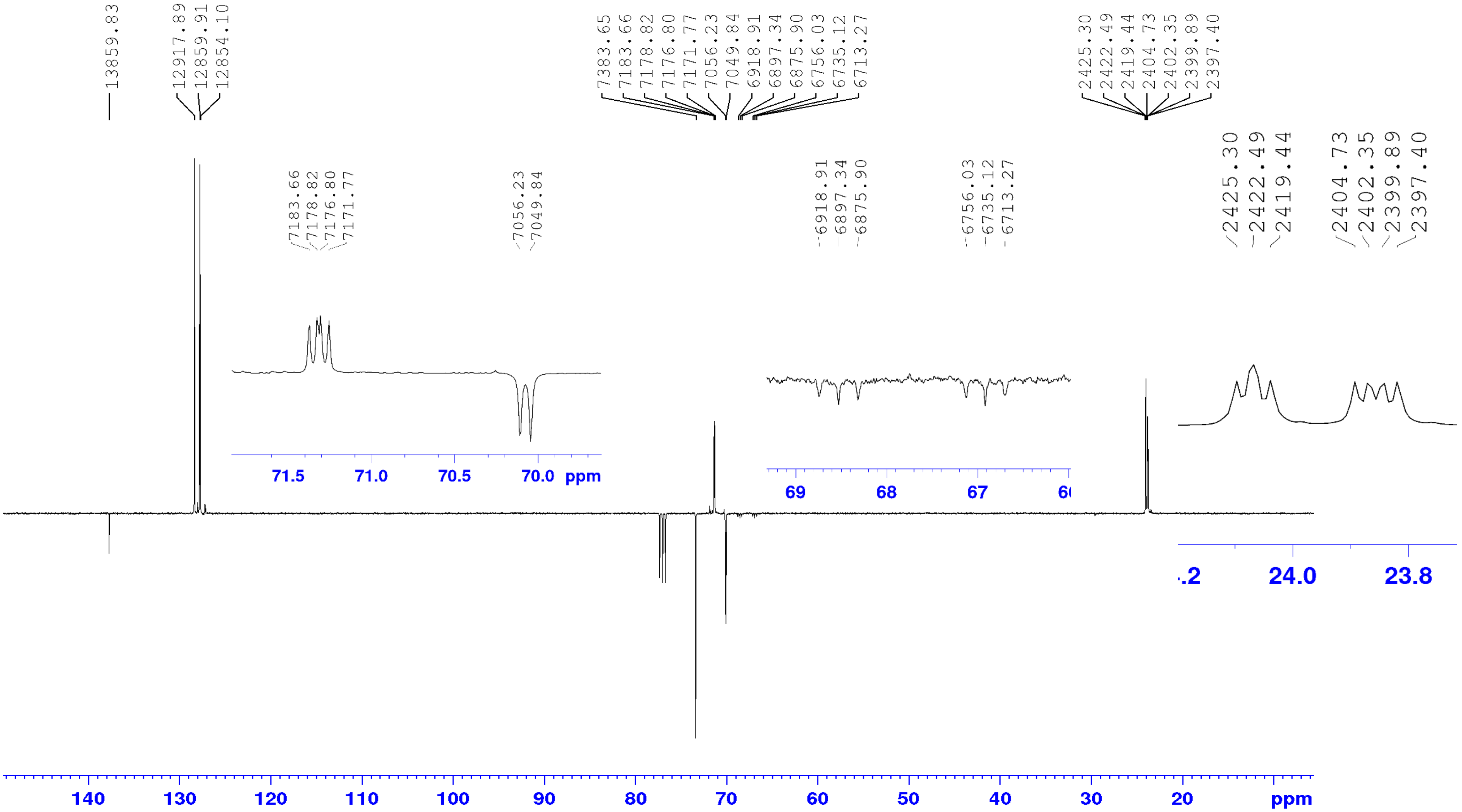

^{1}H NMR of (*R*)- diisopropyl(1-azido-2-(benzyloxy)ethyl)phosphonate (600.25 MHz, $CDCl_3$) [(*R*)-84]:

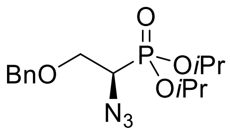

(*R*)-**84**

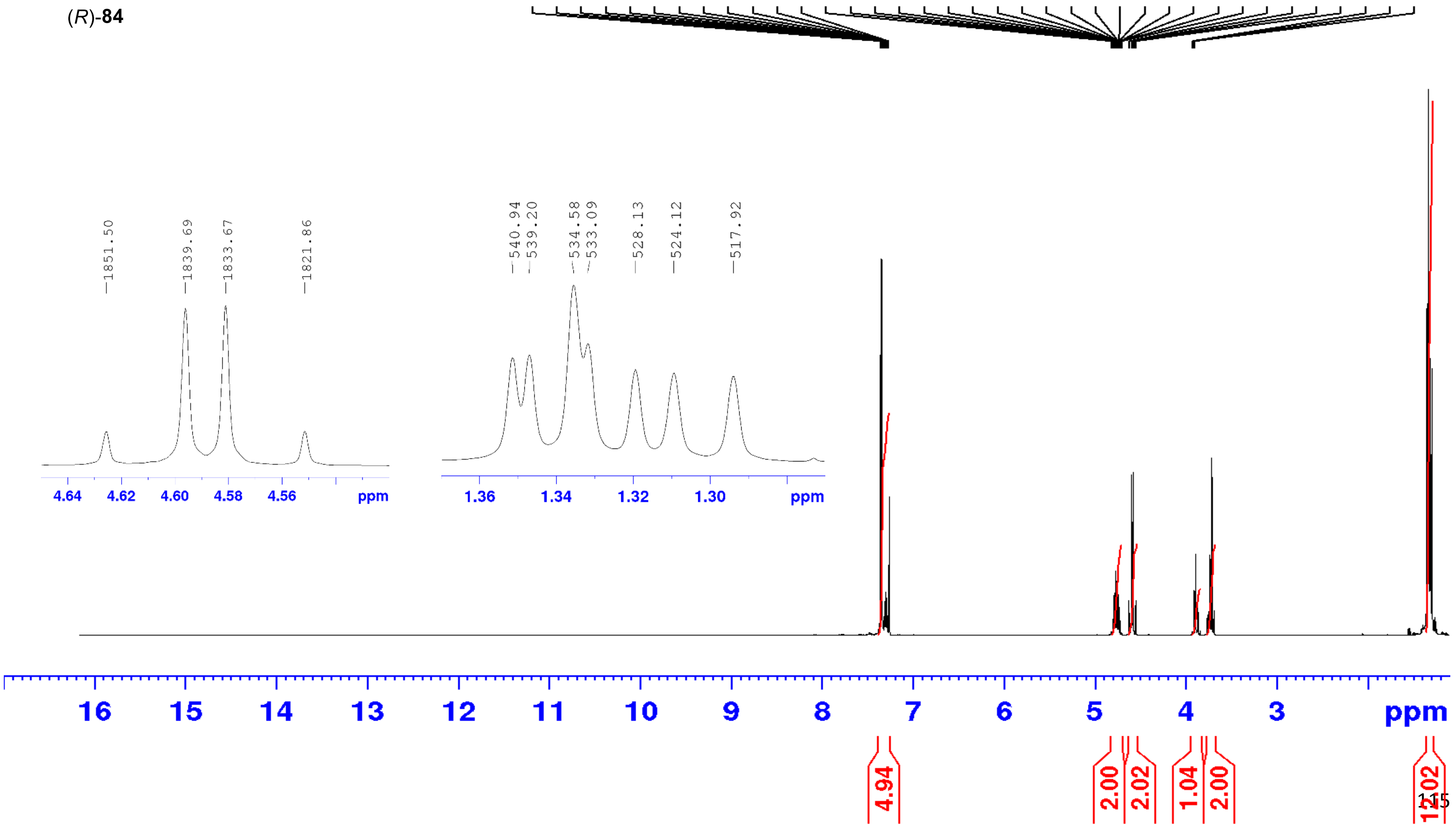

^{13}C NMR of (*R*)- diisopropyl(1-azido-2-(benzyloxy)ethyl)phosphonate (150.93 MHz, $CDCl_3$) [(*R*)-84]:

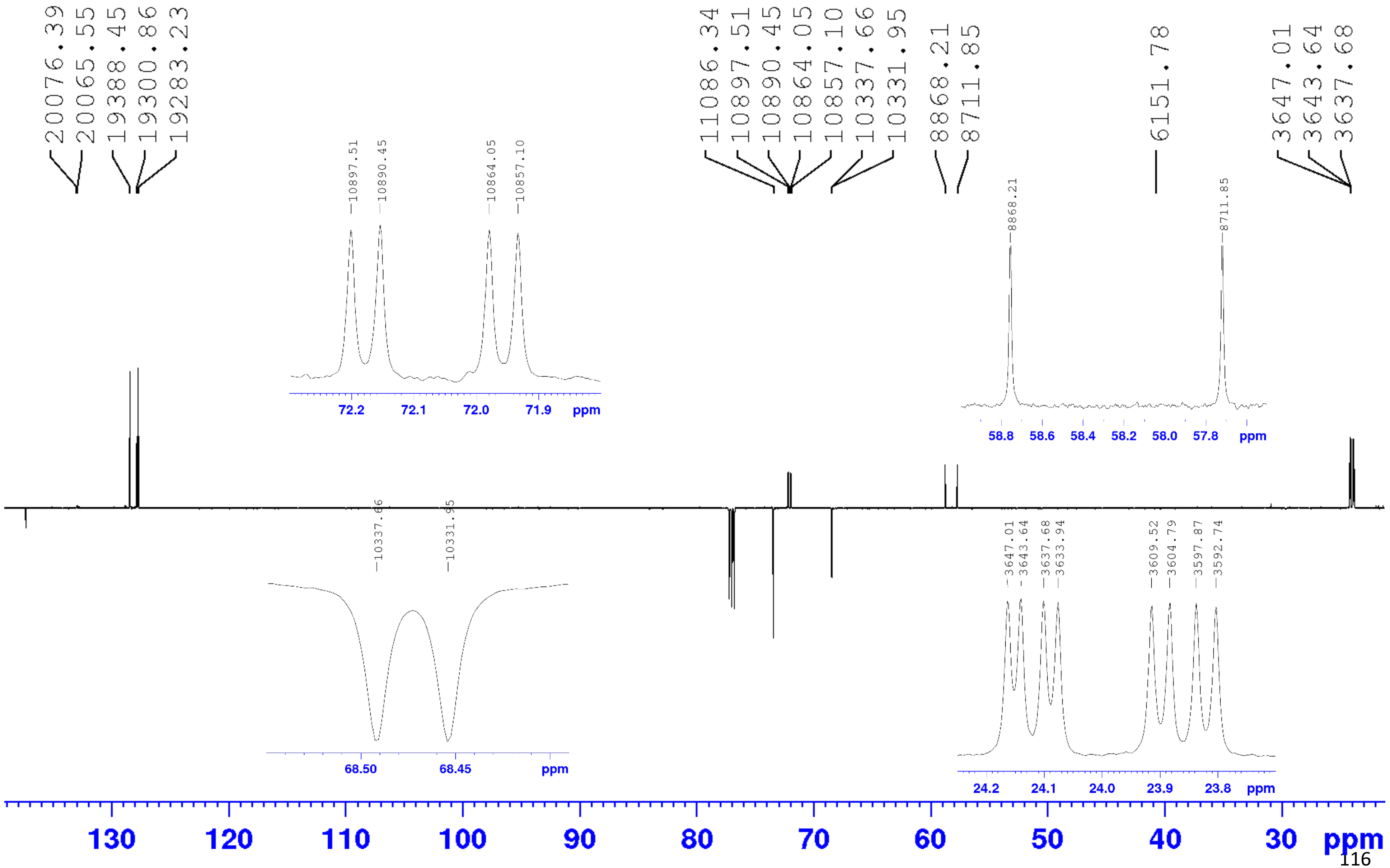

^{31}P NMR of (*R*)- diisopropyl(1-azido-2-(benzyloxy)ethyl)phosphonate (162.03 MHz, $CDCl_3$) [(*R*)-84]:

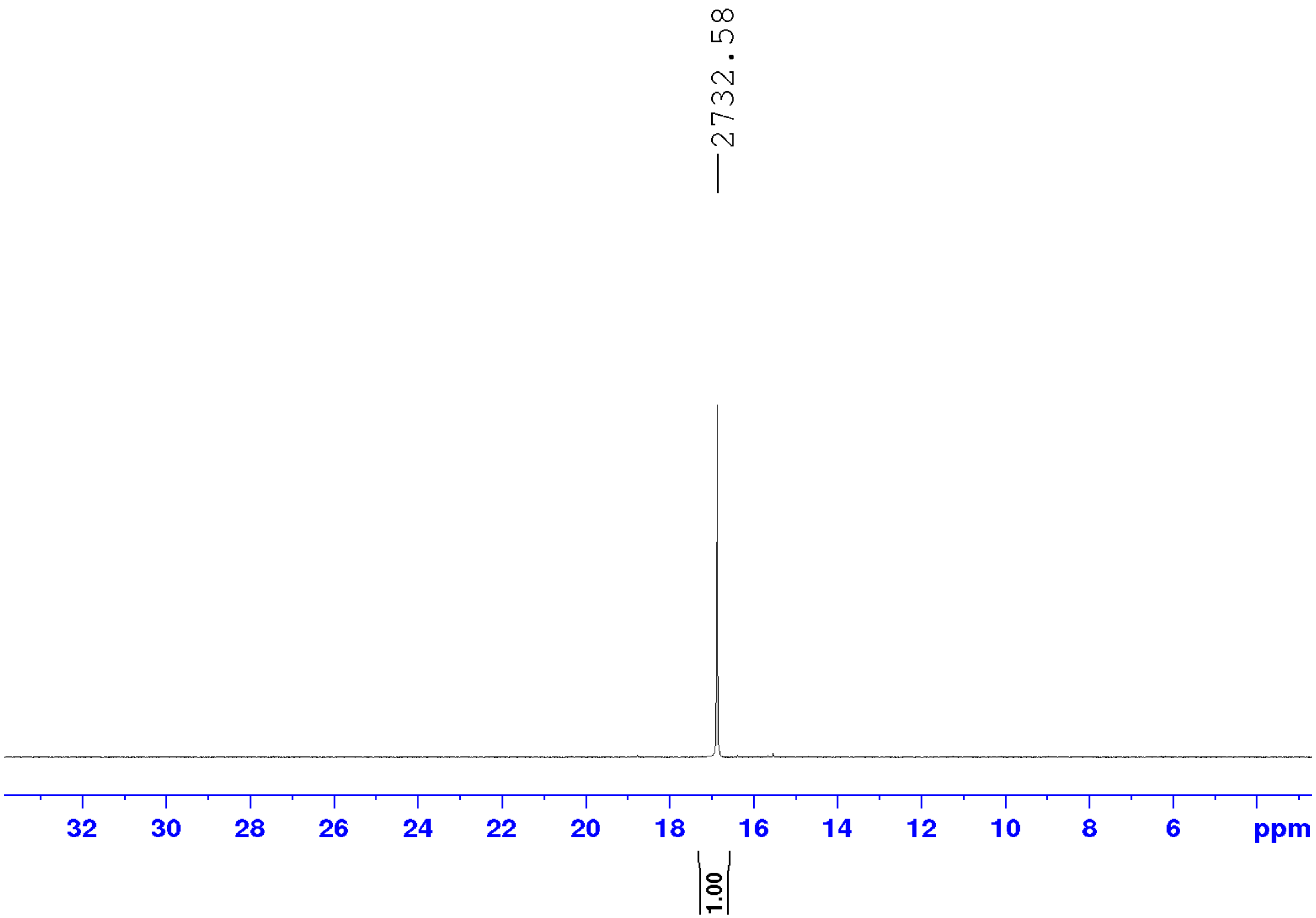

^{1}H NMR of (*R*)-(1-amino-2-hydroxyethyl)phosphonic acid (600.25 MHz, D_2O) [(*R*)-67]:

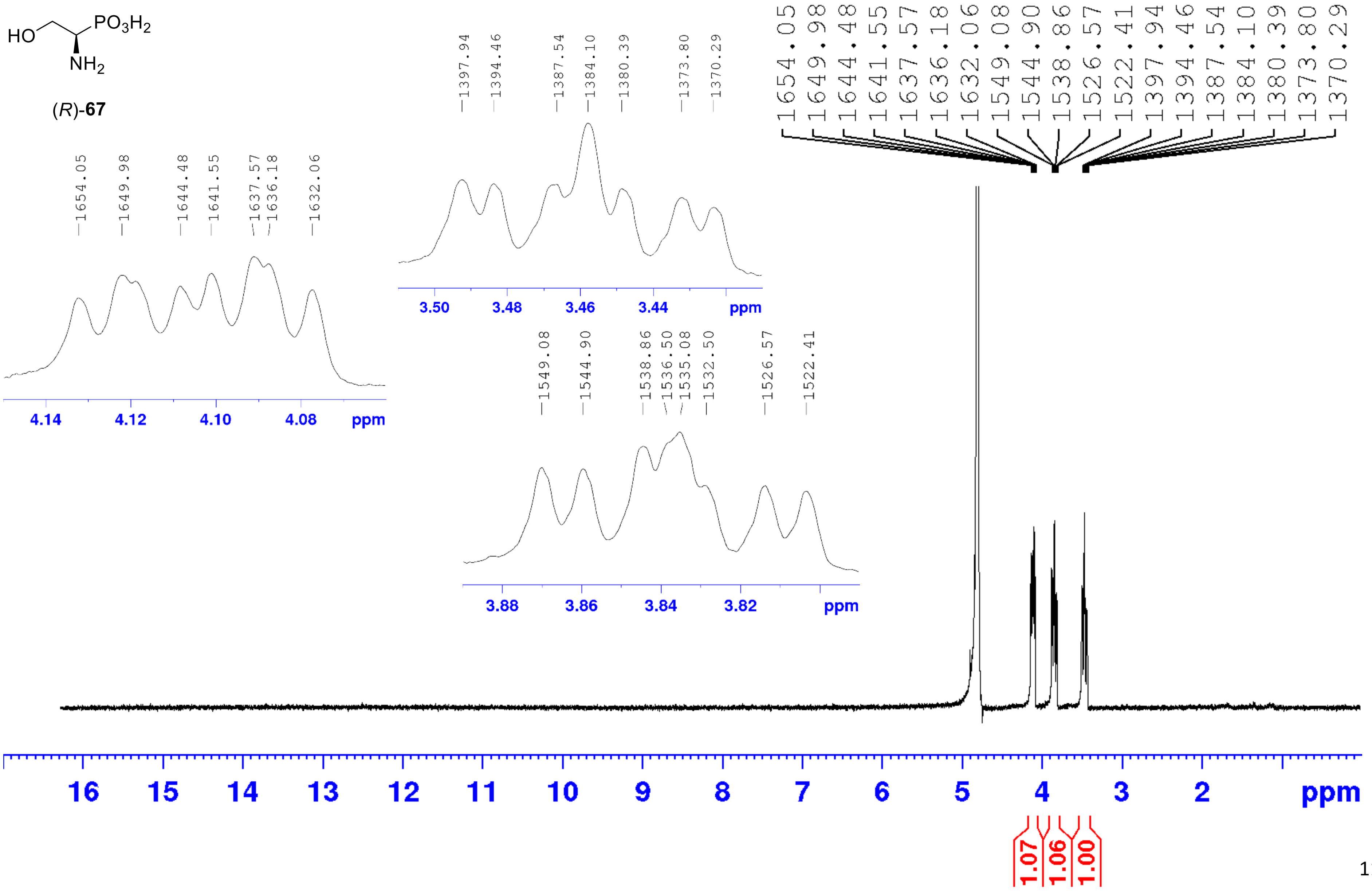

^{31}P NMR of (*R*)-(1-amino-2-hydroxyethyl)phosphonic acid (162.03 MHz, D_2O) [(*R*)-67]:

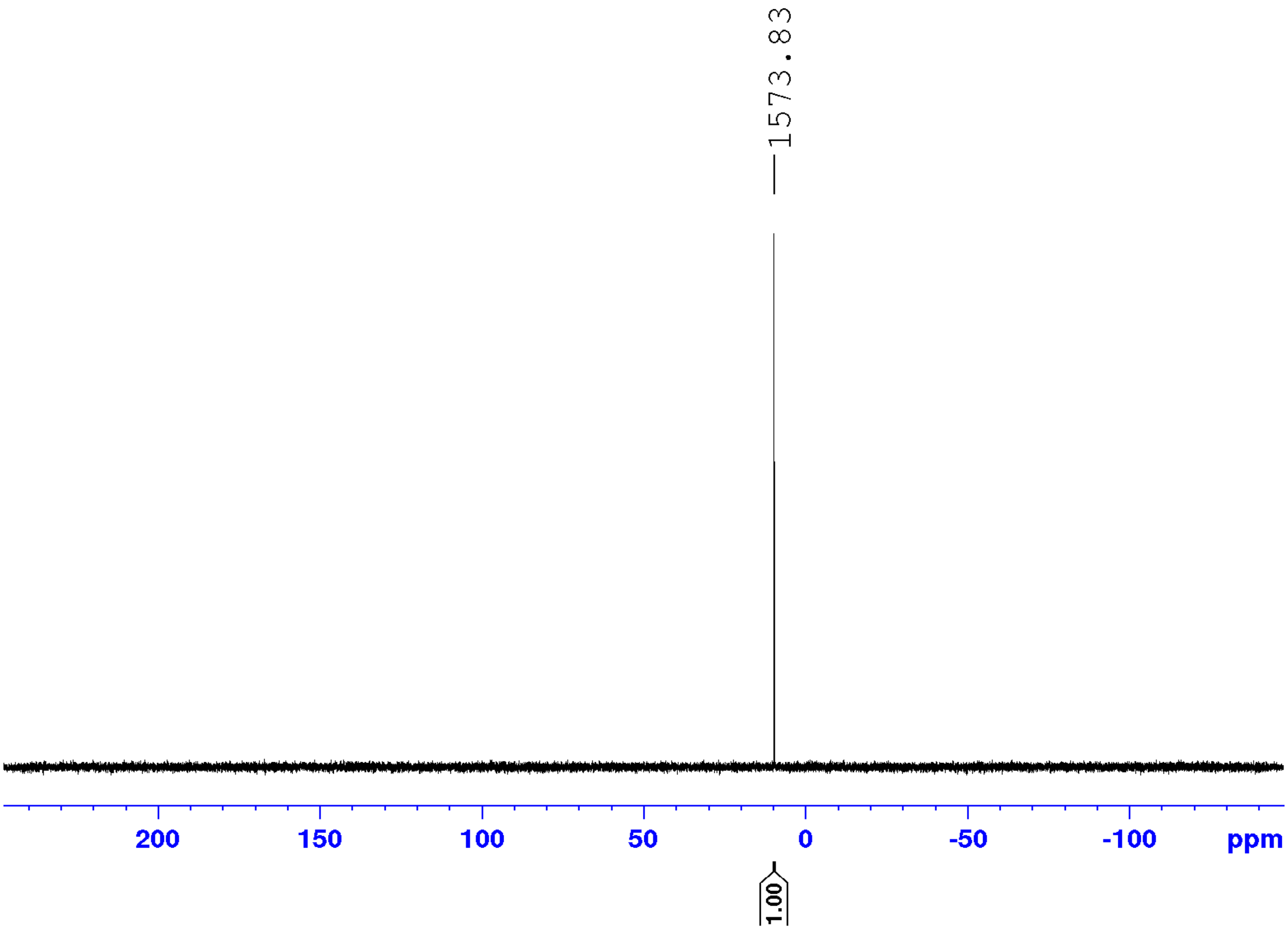

^{31}P NMR of ethyl 3-(diisopropoxyphosphoryl)-3-oxopropanoate (162.03 MHz, $CDCl_3$) (17):

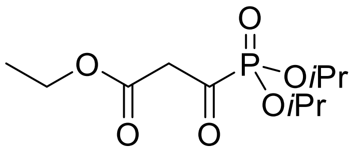

17

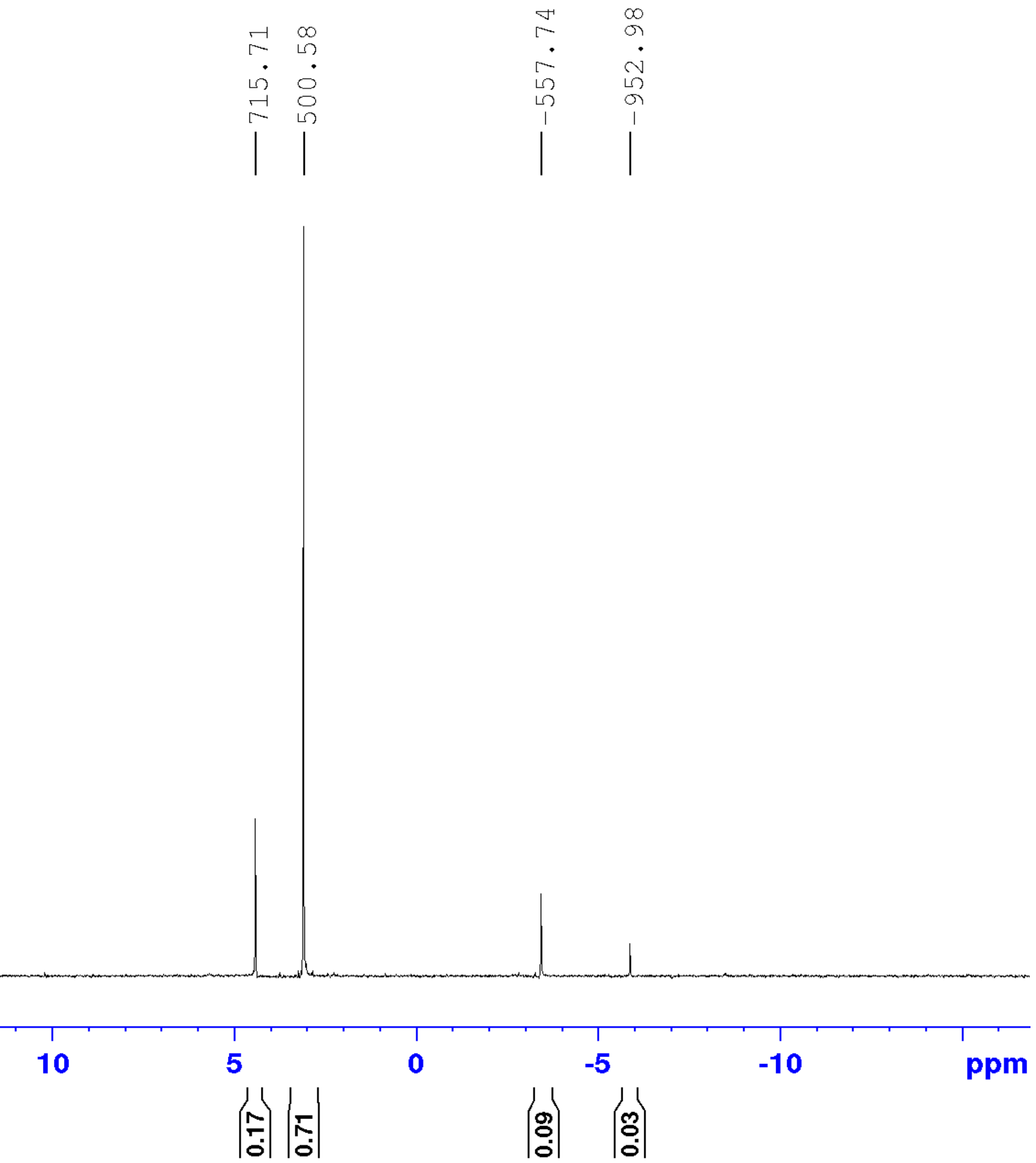

^{1}H NMR of (*S*)-ethyl 3-(diisopropoxyphosphoryl)-3-hydroxypropanoate (400.13 MHz, CDCl$_3$) [(S)-34a]:

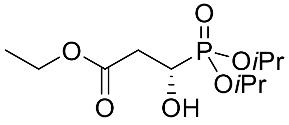

(*S*)-**34a**

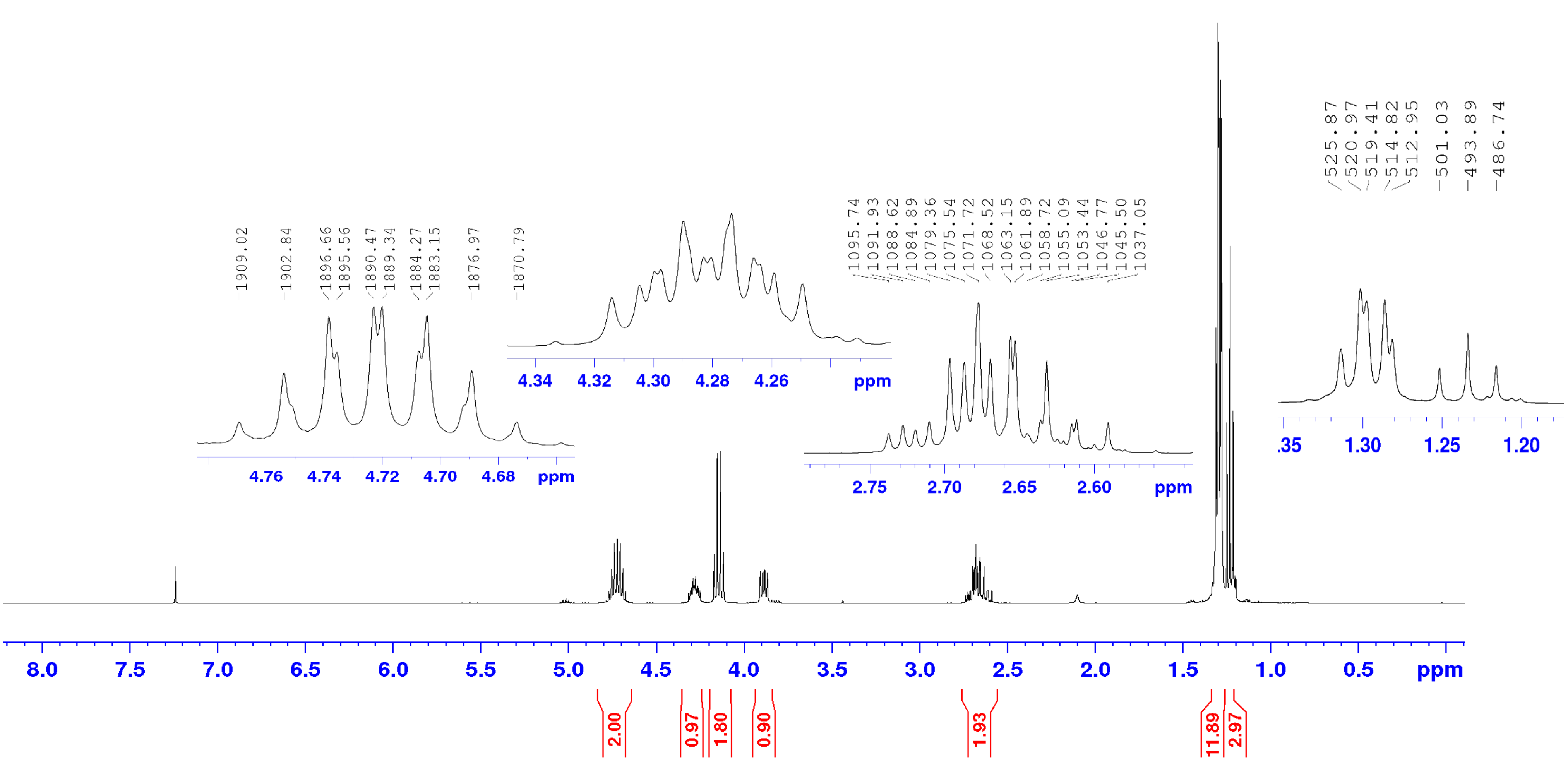

^{13}C NMR of (*S*)-ethyl 3-(diisopropoxyphosphoryl)-3-hydroxypropanoate (100.65 MHz, CDCl$_3$) [(S)- 34a]:

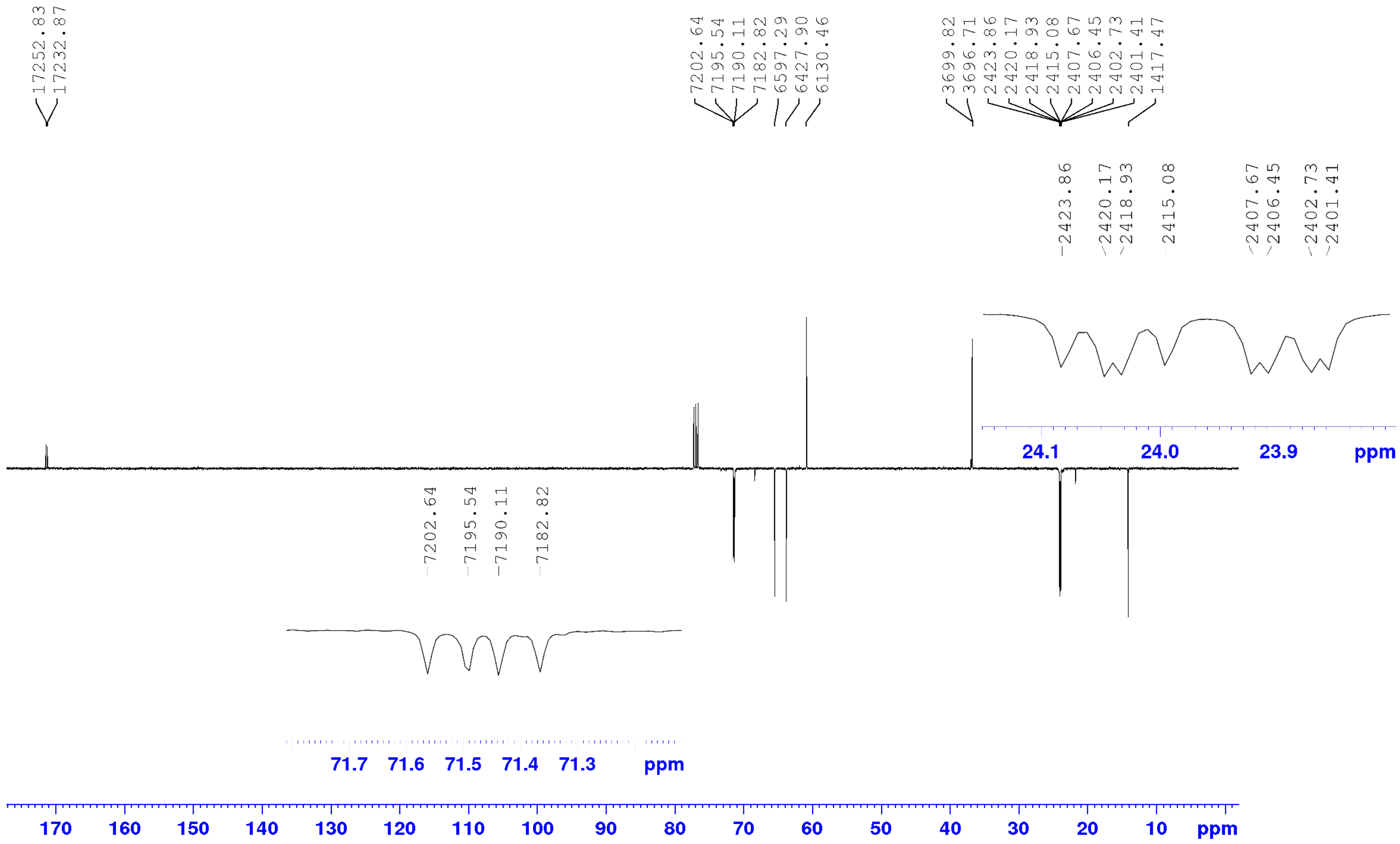

^{31}P NMR of (*S*)-ethyl 3-(diisopropoxyphosphoryl)-3-hydroxypropanoate (162.03 MHz, $CDCl_3$) [(*S*)-34a]:

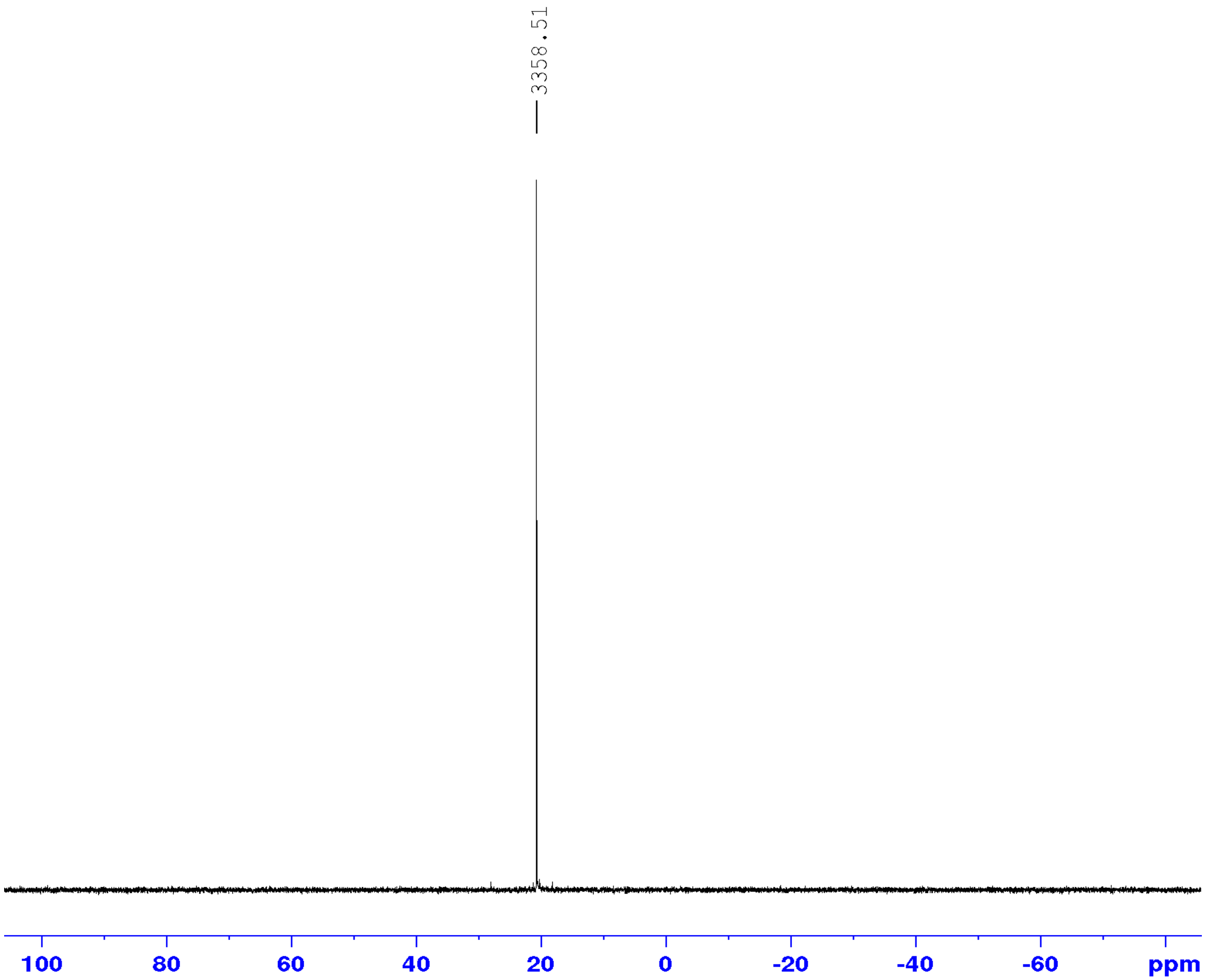

^{31}P NMR of methyl 4-(diisopropoxyphosphoryl)-4-oxobutanoate (162.03 MHz, $CDCl_3$) (18):

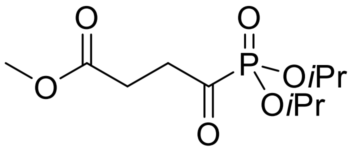

18

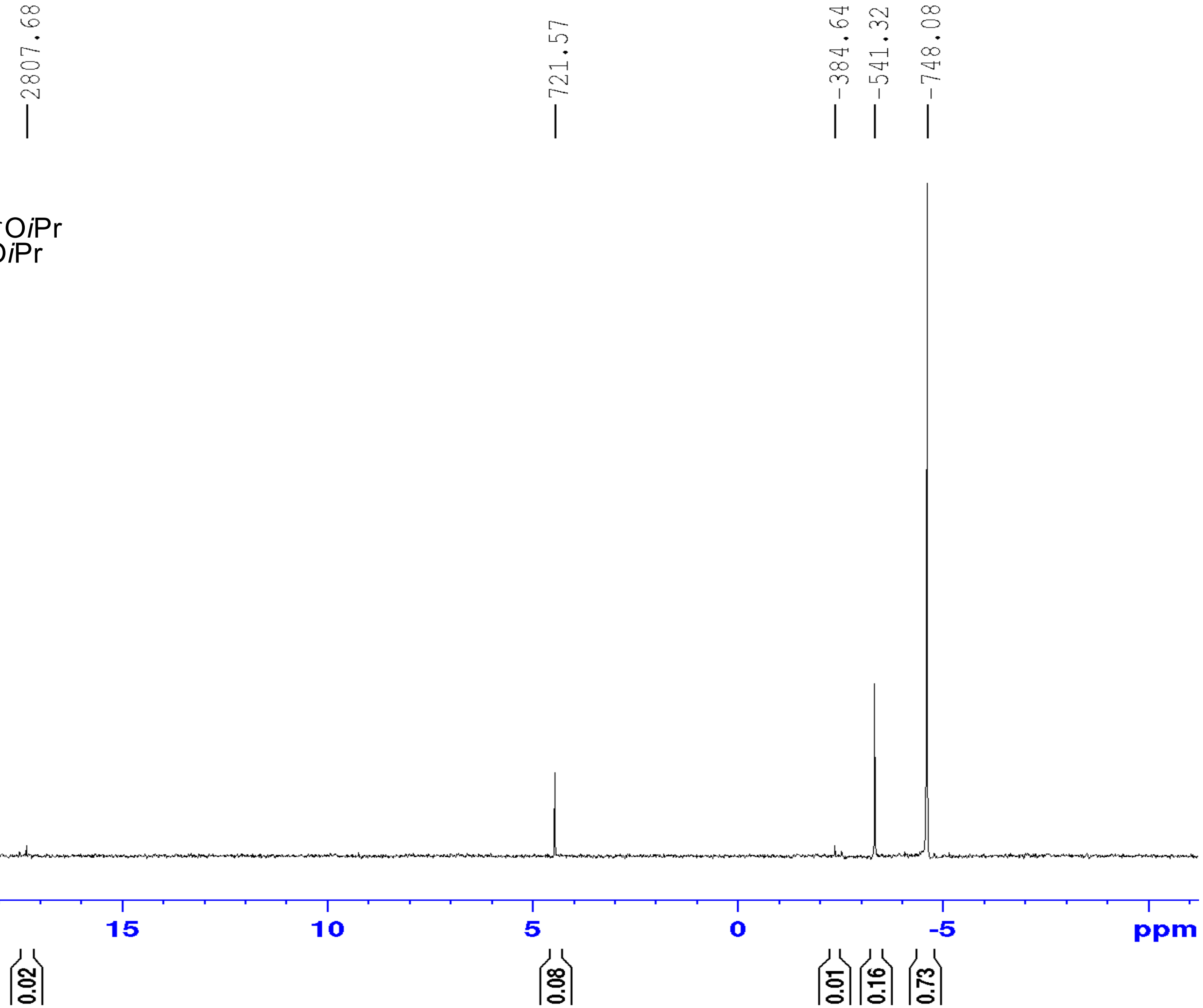

^{1}H NMR of methyl (*S*)-4-(diisopropoxyphosphoryl)-4-hydroxybutanoate (600.25 MHz, $CDCl_3$) [(*S*)-35]:

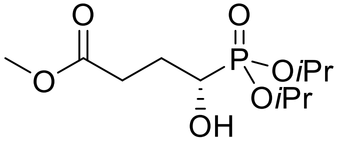

(*S*)-**35**

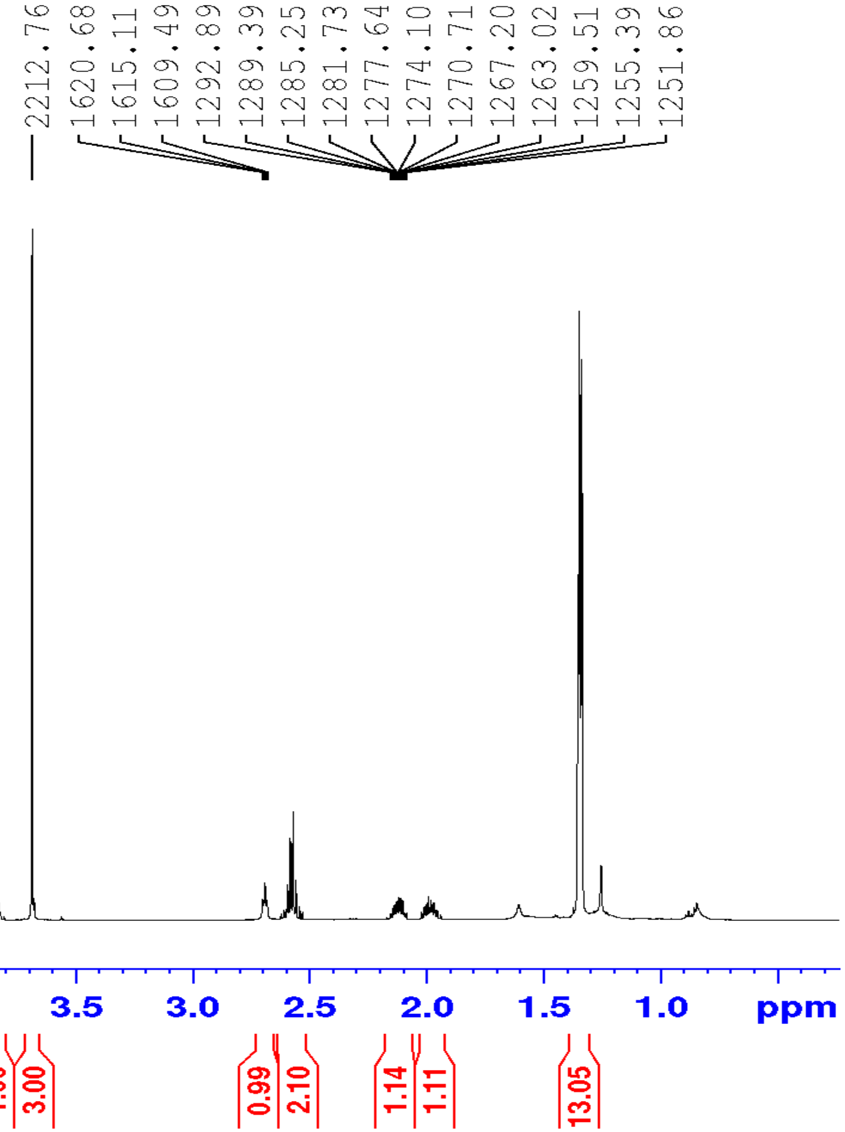

^{31}P NMR of methyl (*S*)-4-(diisopropoxyphosphoryl)-4-hydroxybutanoate (162.03 MHz, $CDCl_3$) [(*S*)-35]:

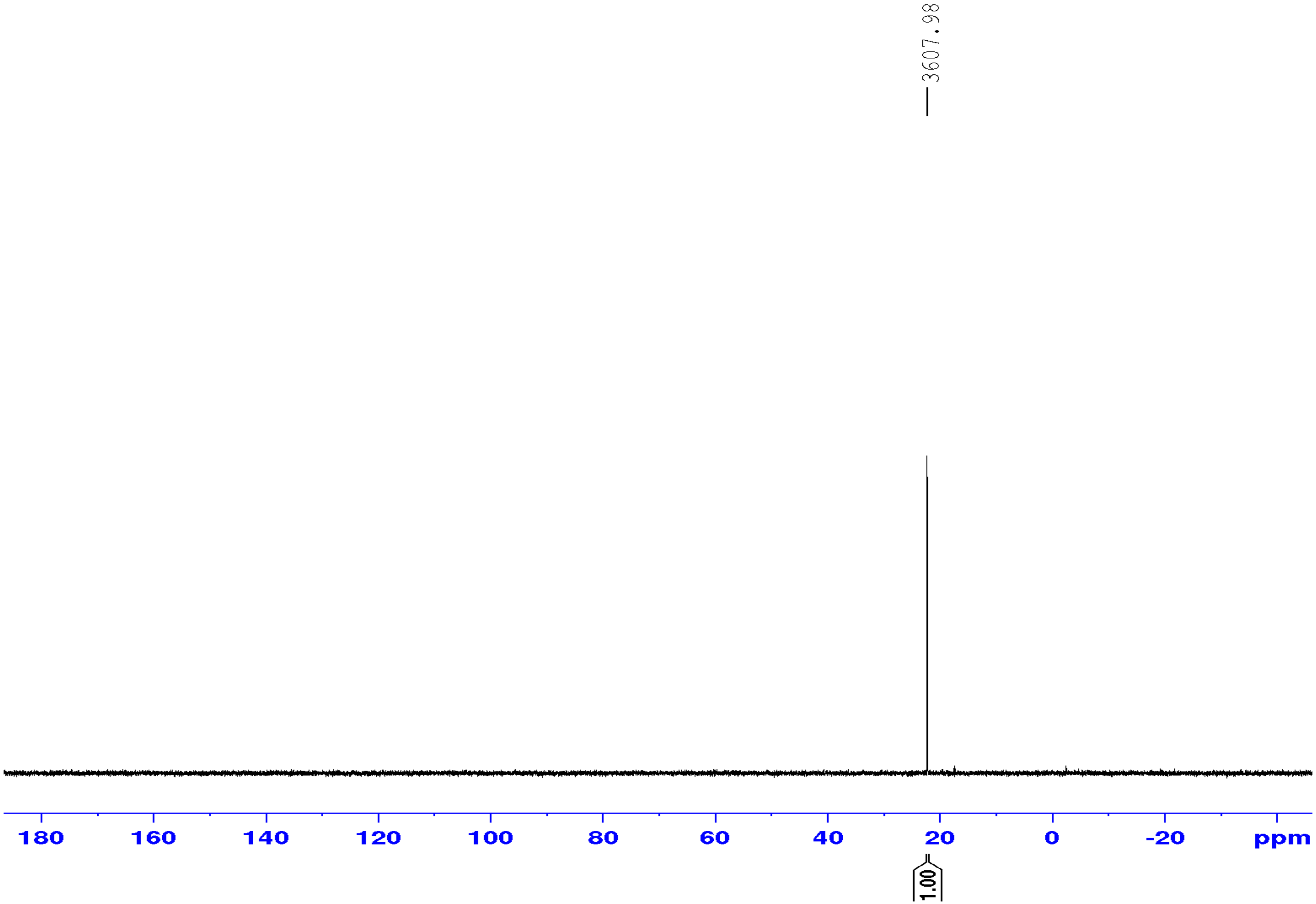

^{13}C NMR of methyl (*S*)-4-(diisopropoxyphosphoryl)-4-hydroxybutanoate (150.93 MHz, $CDCl_3$) [(*S*)-35]:

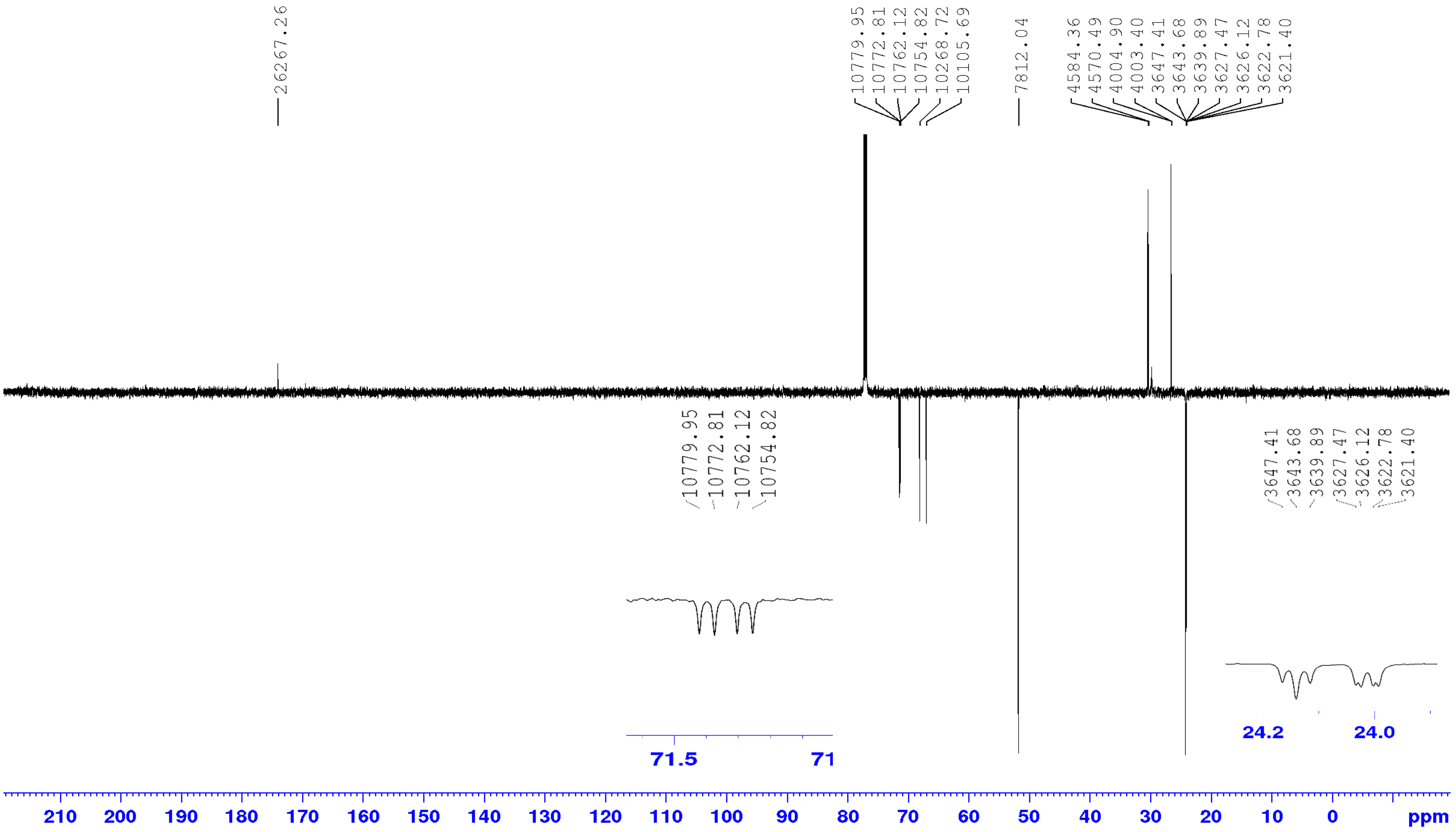

^{1}H NMR of methyl (*R*)-4-(diisopropoxyphosphoryl)-4-azidobutanoate (400.13 MHz, $CDCl_3$) [(*R*)-85]:

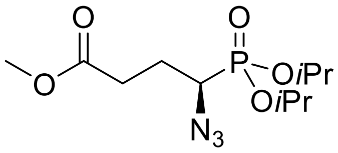

(*R*)-**85**

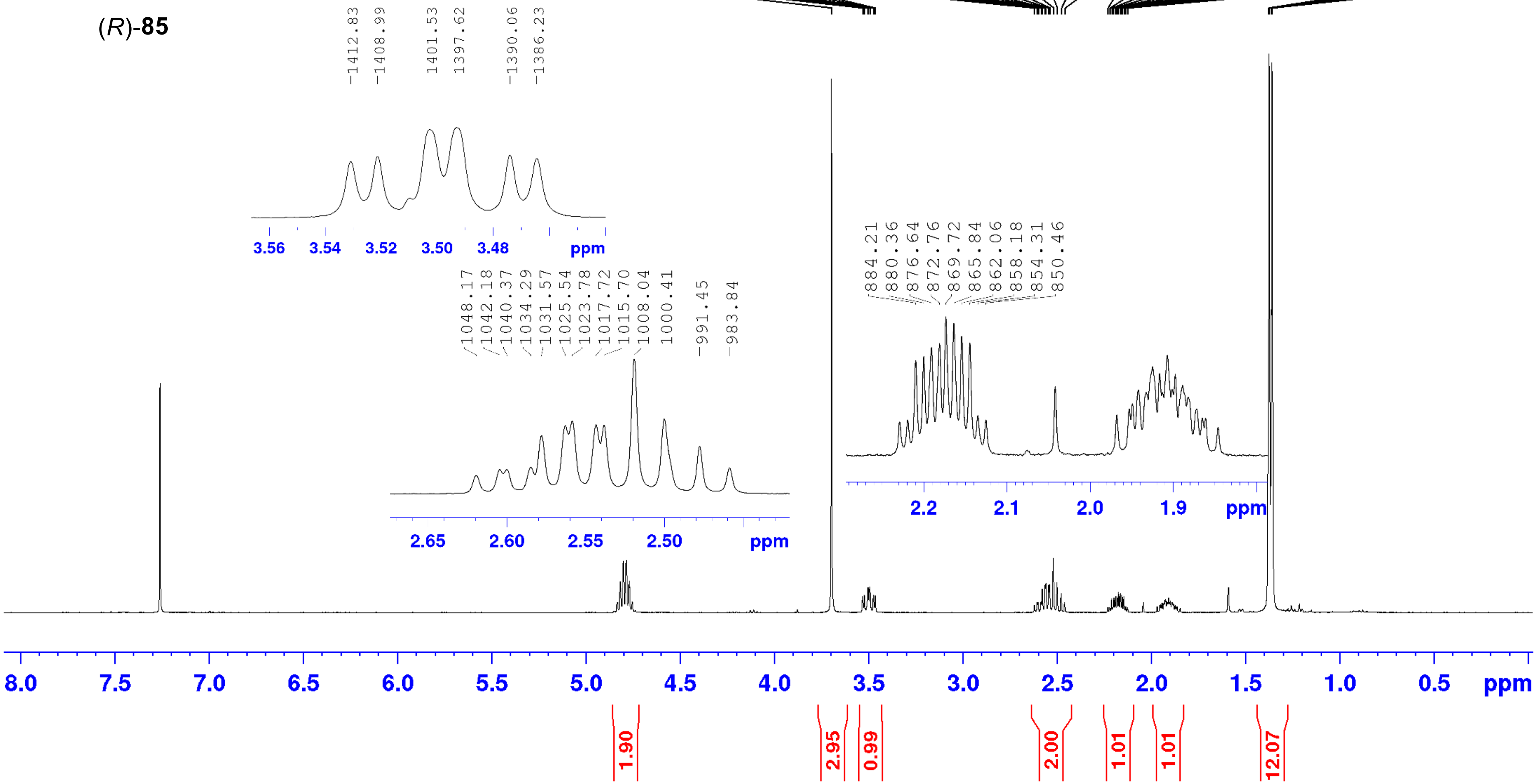

^{31}P NMR of methyl (*R*)-4-(diisopropoxyphosphoryl)-4-azidobutanoate (161.98 MHz, $CDCl_3$) [(*R*)-85]:

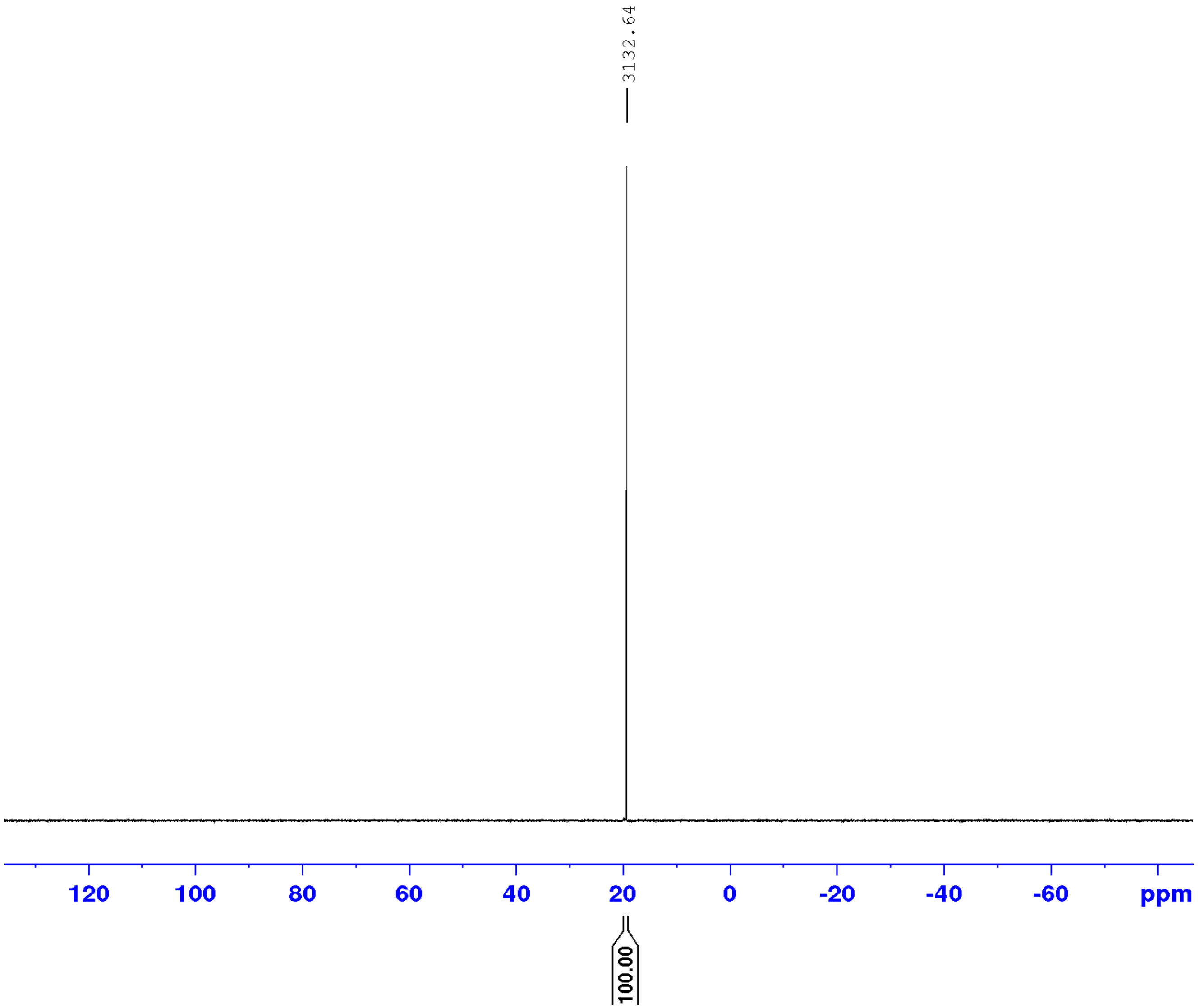

^{13}C NMR of methyl (*R*)-4-(diisopropoxyphosphoryl)-4-azidobutanoate (176.11 MHz, CDCl$_3$) [(*R*)-85]:

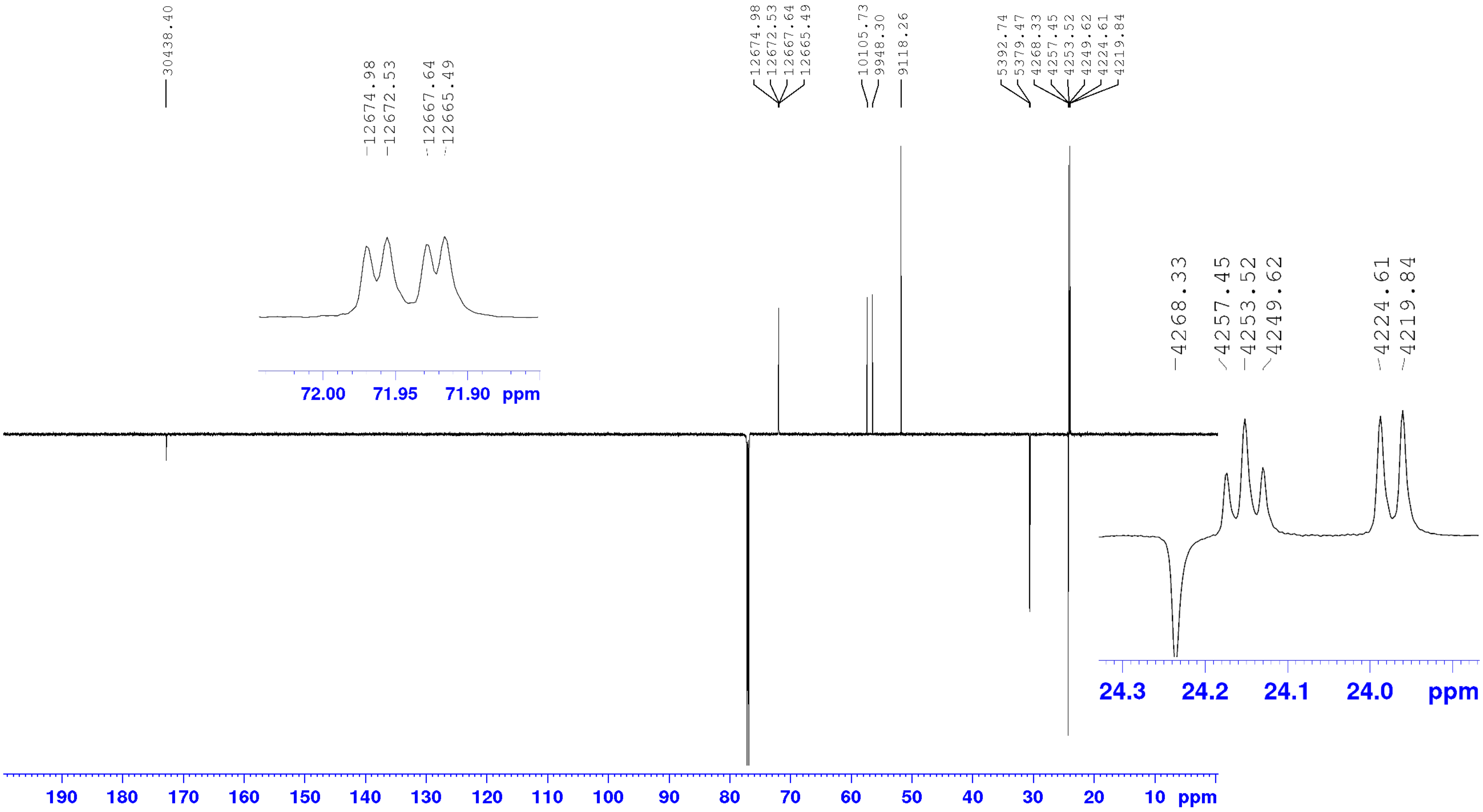

^{1}H NMR of (*R*)-4-amino-4-phosphoryl-butanoic acid, (*R*)-phosphaglutamic acid (600.25 MHz, $CDCl_3$) [(*R*)-68]:

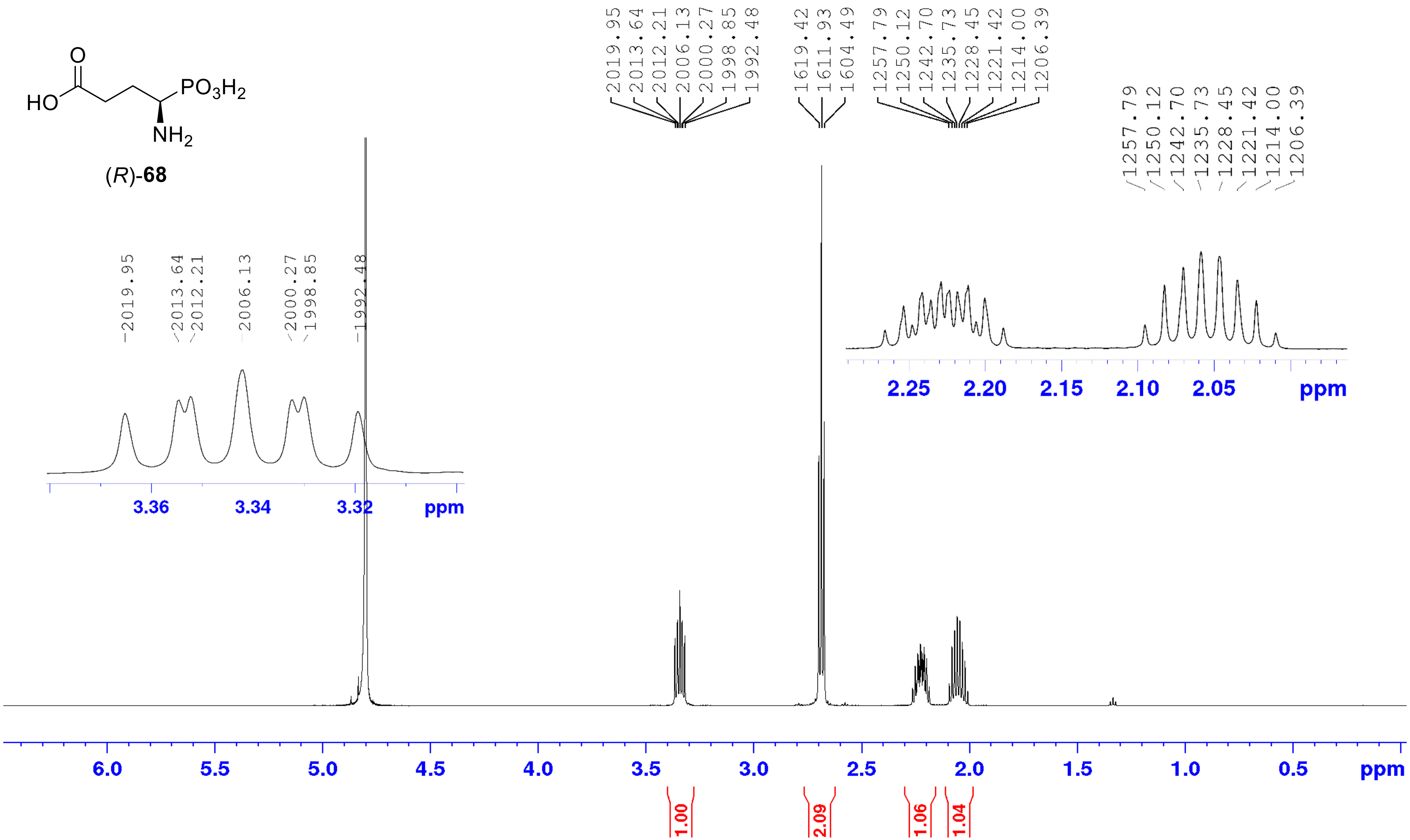

^{31}P NMR of (*R*)-4-amino-4-phosphoryl-butanoic acid, (*R*)-phosphaglutamic acid (162.03 MHz, $CDCl_3$) [(*R*)-68]:

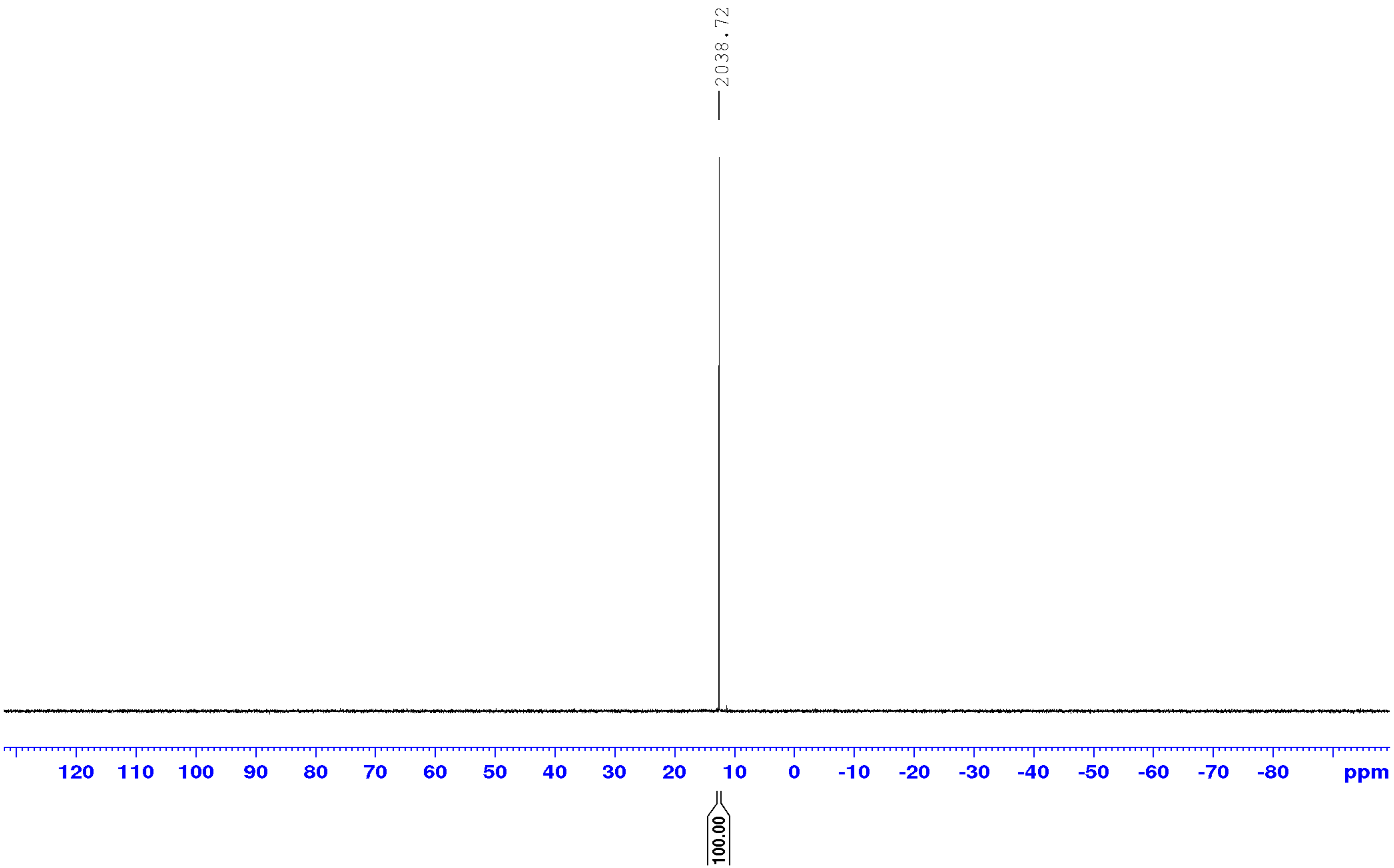

^{13}C NMR of (*R*)-4-amino-4-phosphoryl-butanoic acid, (*R*)-phosphaglutamic acid (150.93 MHz, $CDCl_3$) [(*R*)-68]:

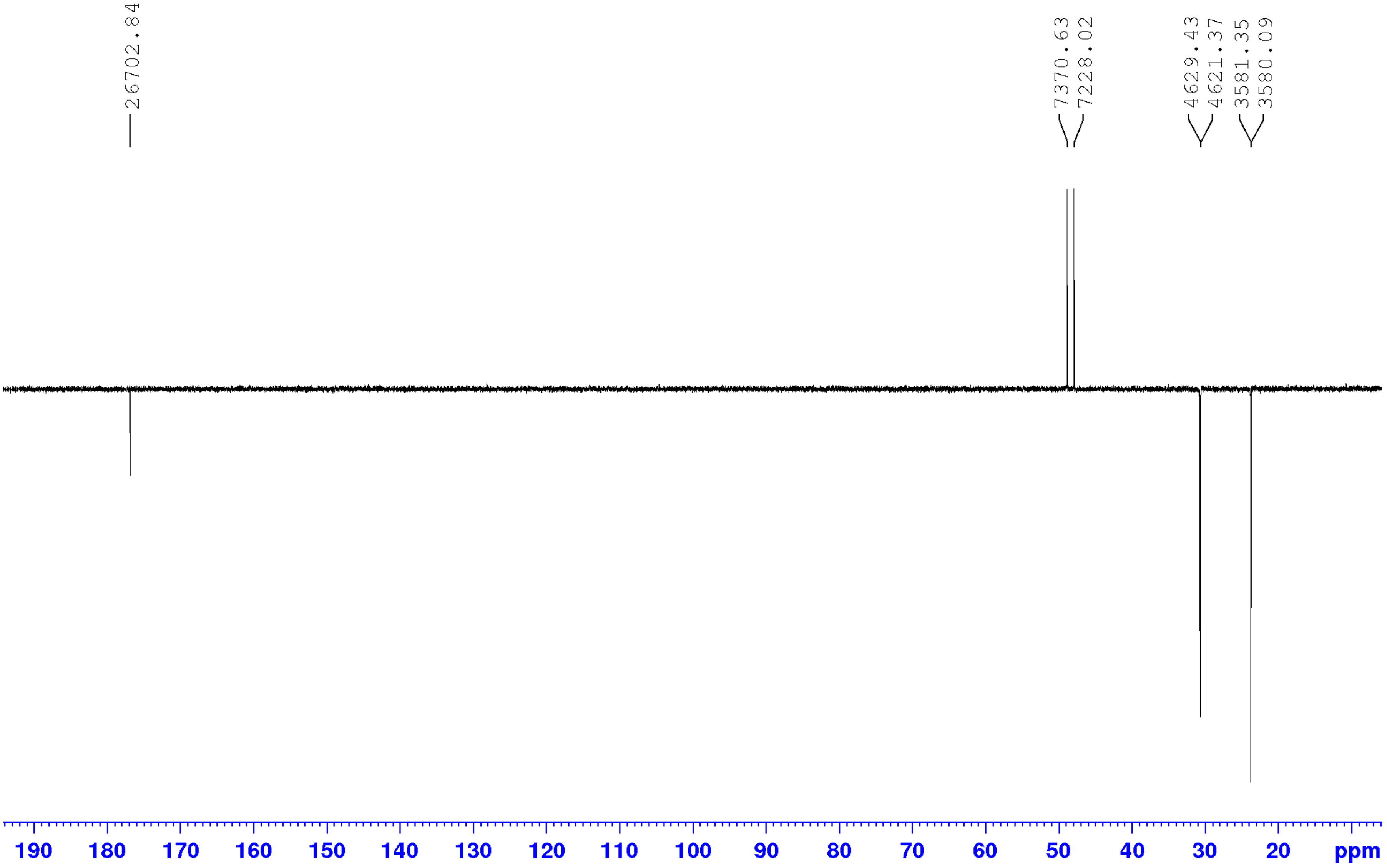

^{1}H NMR of diisopropyl (*R*)-(4-amino-1-azido-4-oxobutyl)-phosphonate (400.27 MHz, $CDCl_3$) [(*R*)-86]:

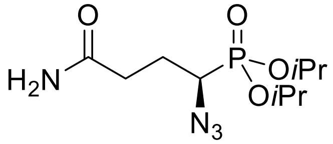

(*R*)-**86**

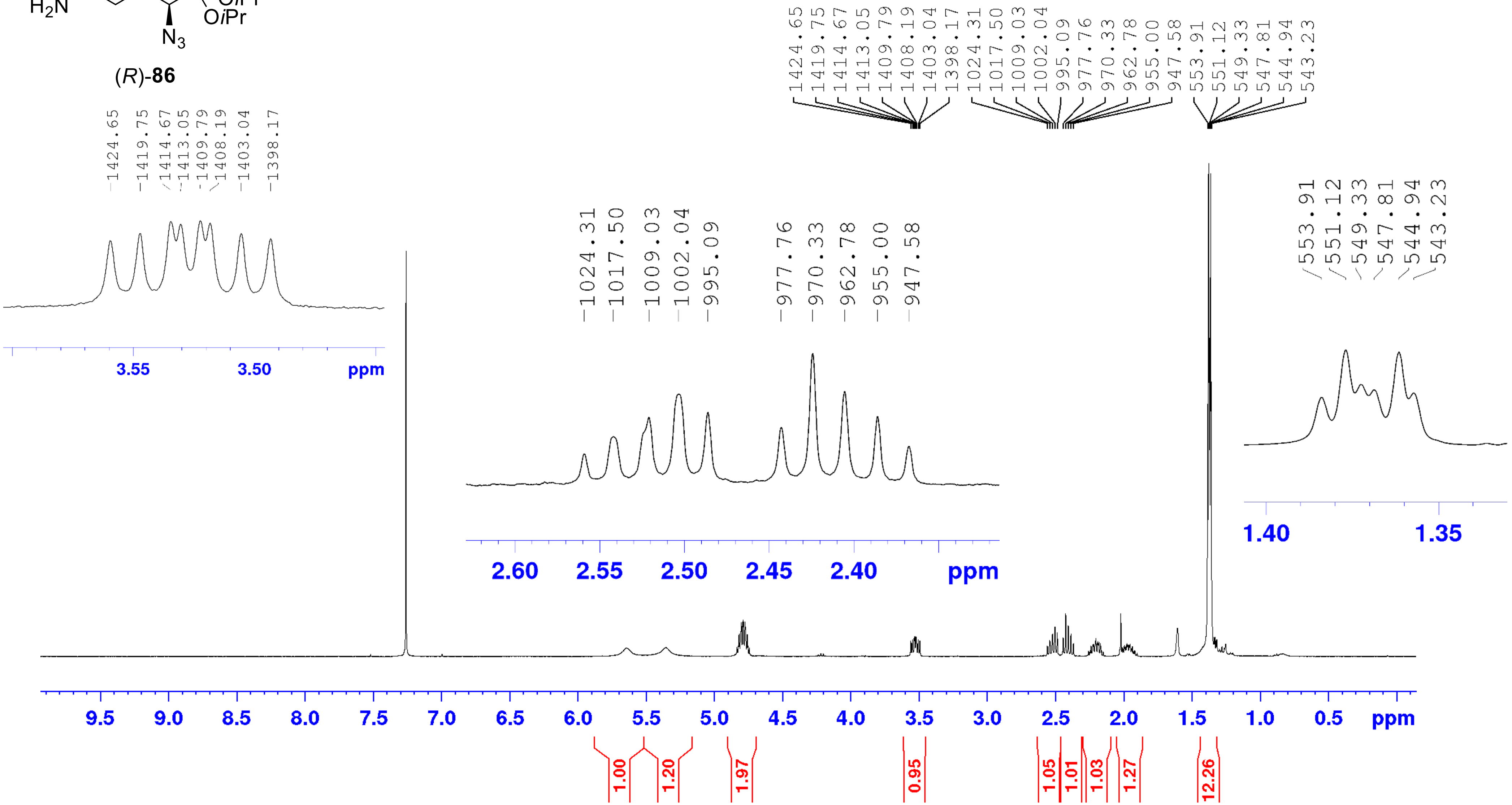

^{31}P NMR of diisopropyl (*R*)-(4-amino-1-azido-4-oxobutyl)-phosphonate (162.02 MHz, $CDCl_3$) [(*R*)-86]:

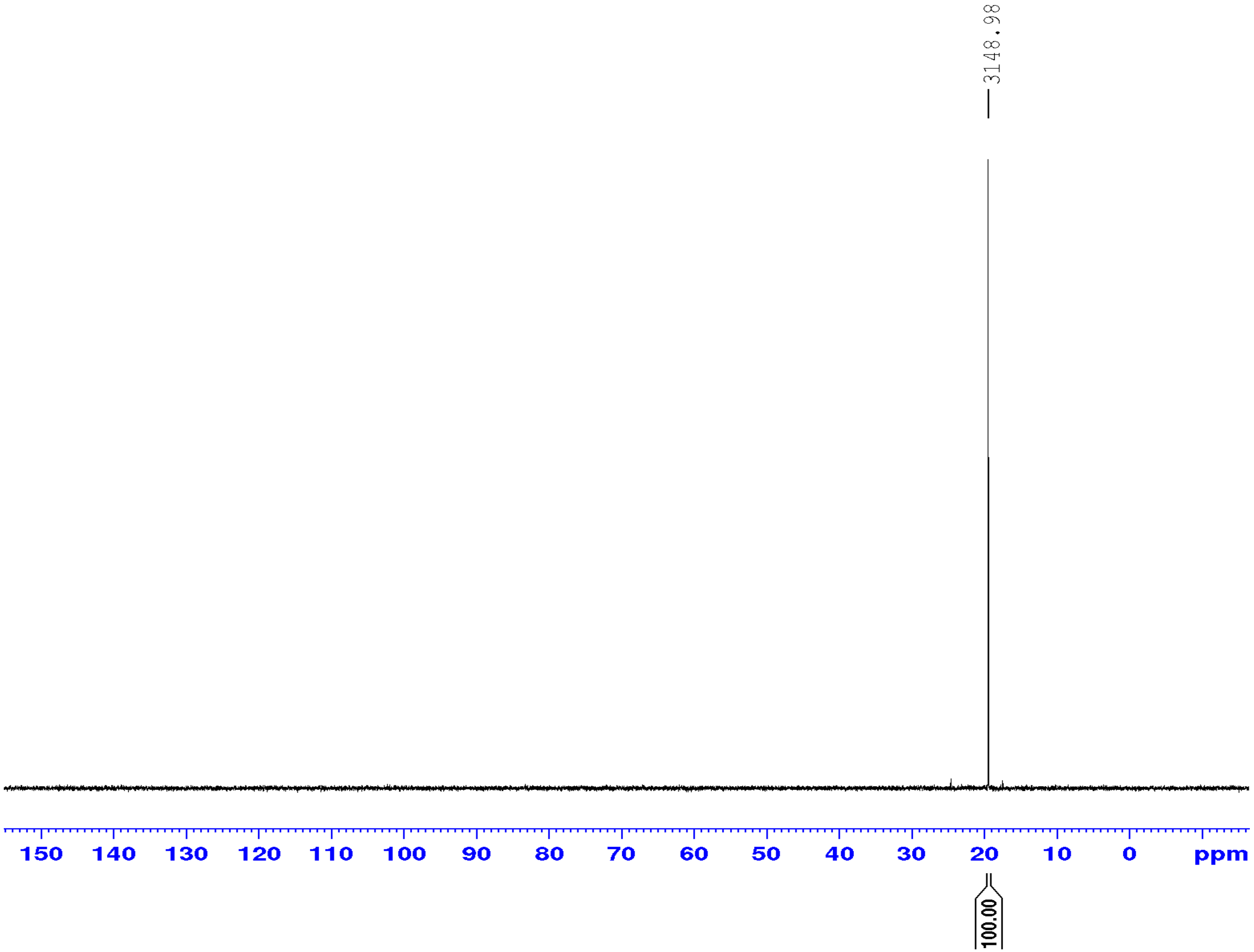

^{13}C NMR of diisopropyl (*R*)-(4-amino-1-azido-4-oxobutyl)-phosphonate (MHz, $CDCl_3$) [(*R*)-86]:

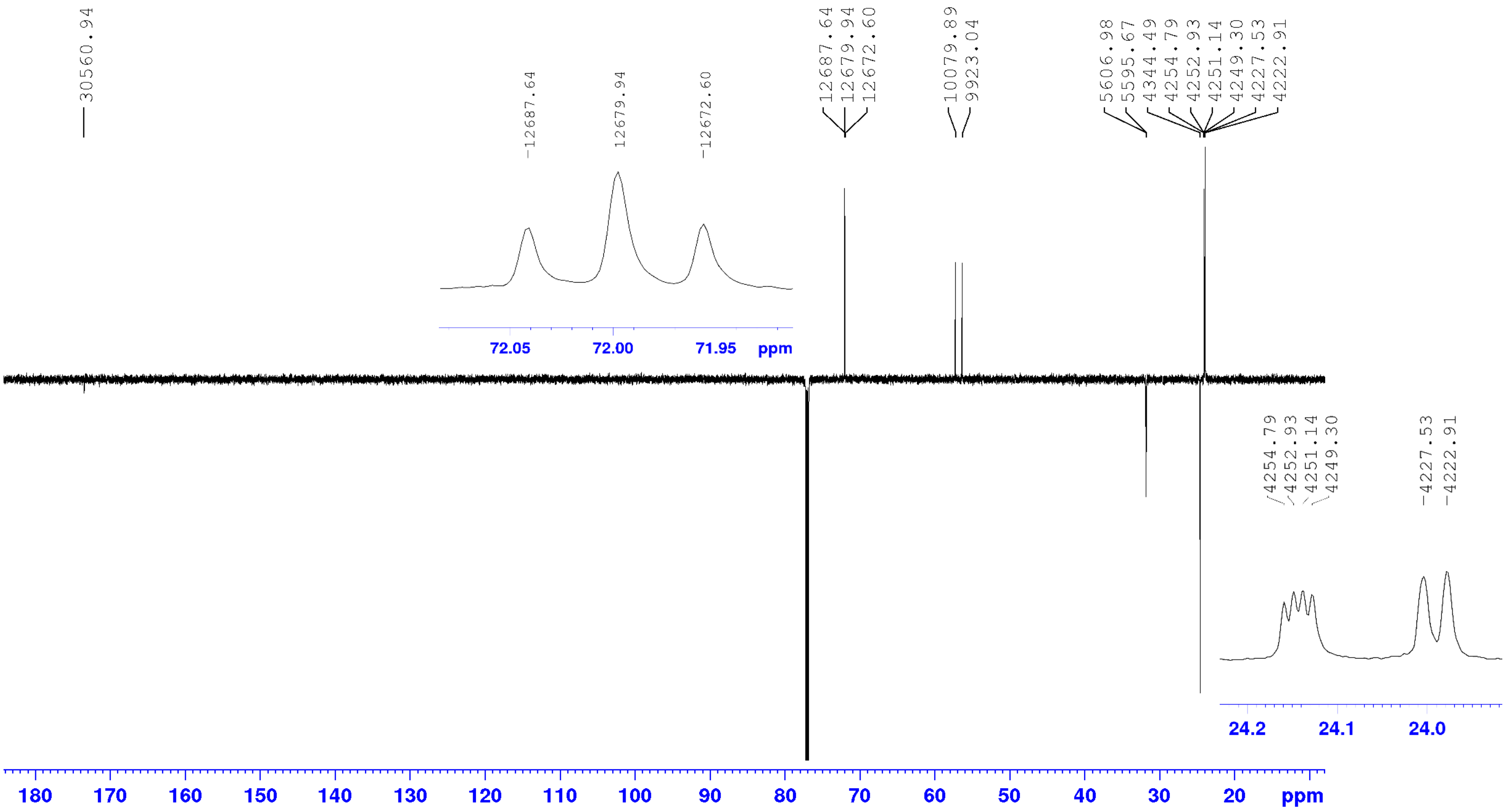

^{1}H NMR of (*R*)-1,4-diamino-4-oxobutylphosphonic acid, (*R*)-phosphaglutamine (400.13 MHz, D_2O) [(*R*)-69]:

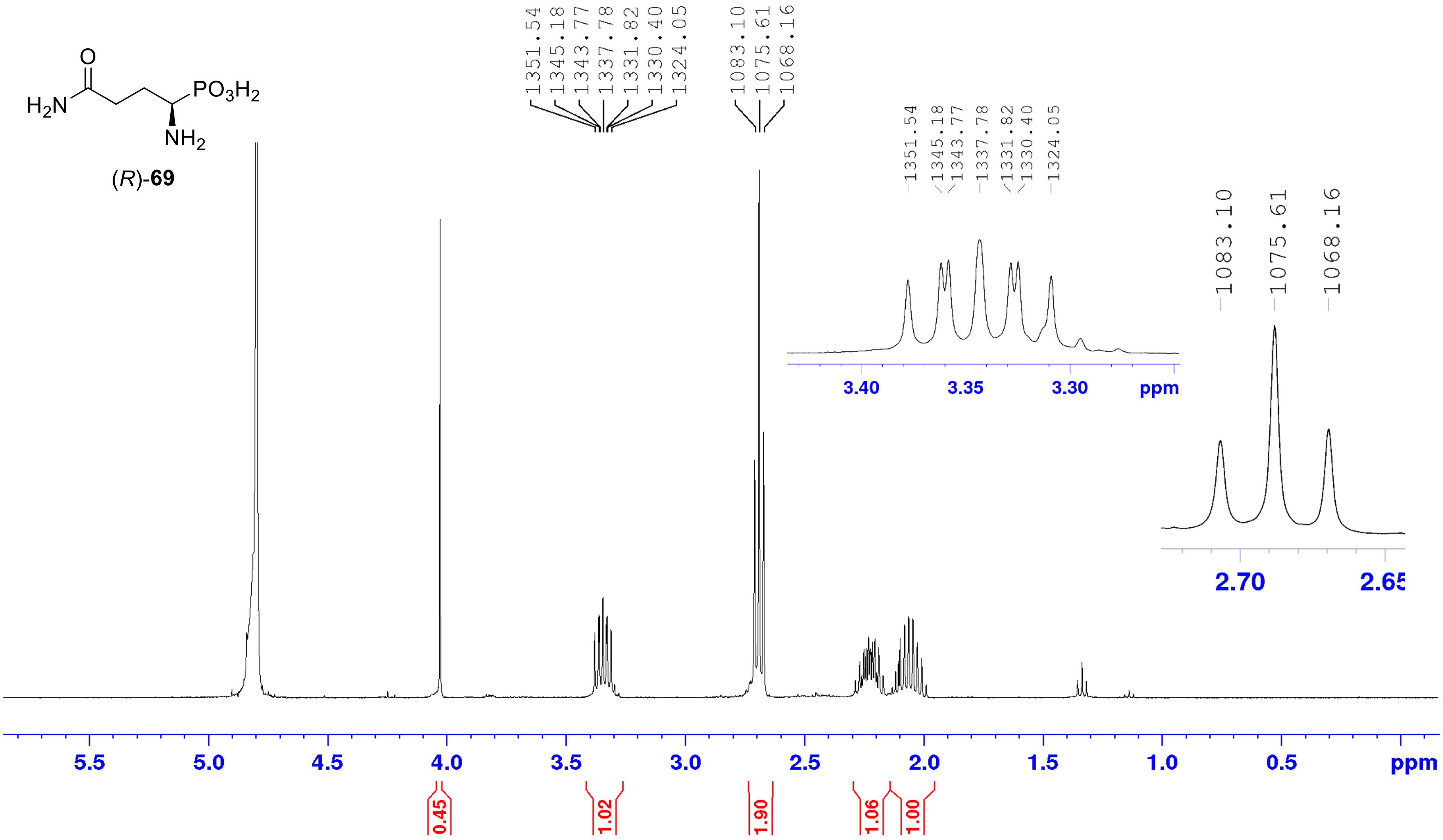

^{31}P NMR of (*R*)-1,4-diamino-4-oxobutylphosphonic acid, (*R*)-phosphaglutamine (161.98 MHz, D_2O) [(*R*)-69]:

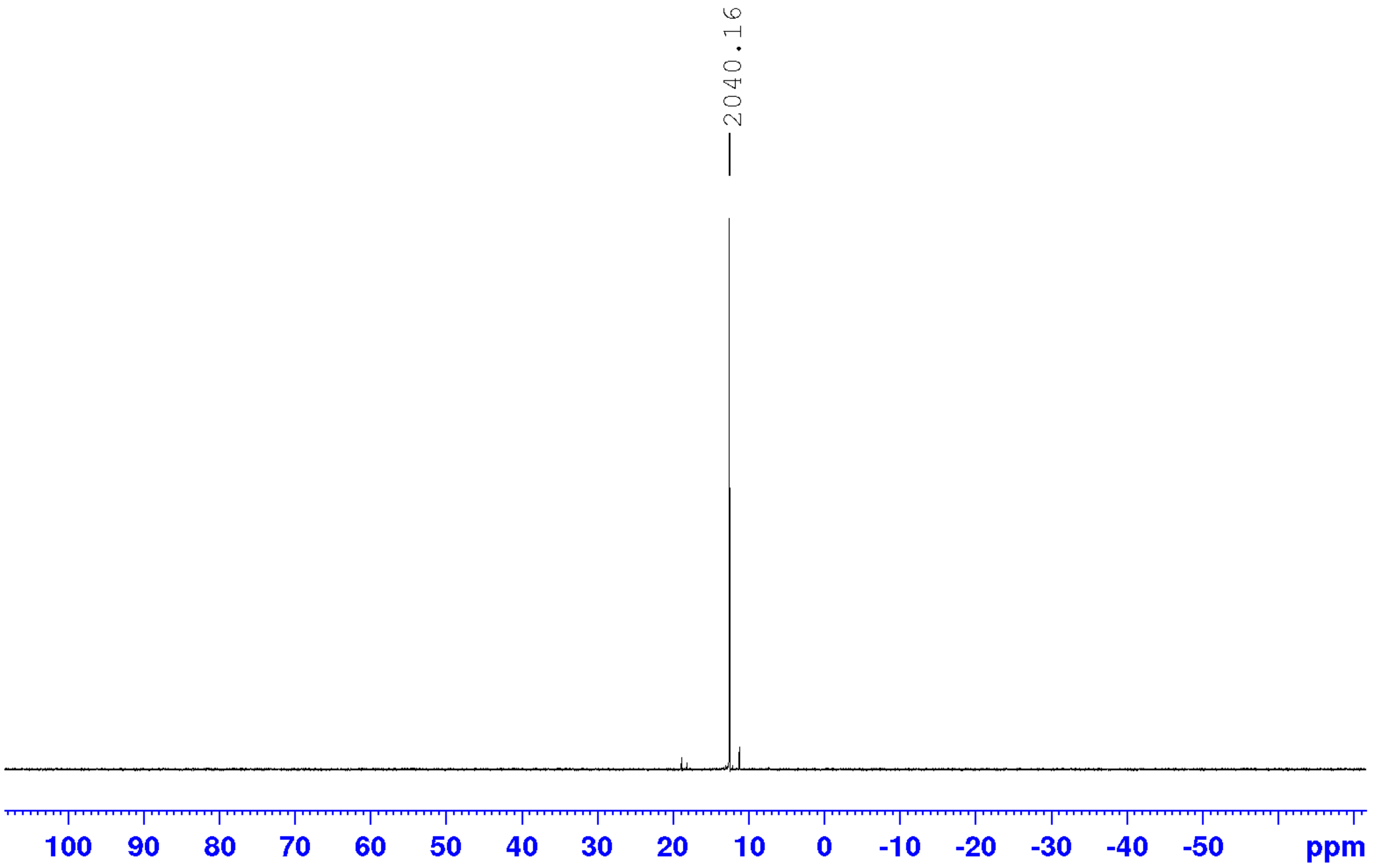

^{13}C NMR of (*R*)-1,4-diamino-4-oxobutylphosphonic acid, (*R*)-phosphaglutamine (100.61 MHz, D_2O) [(*R*)-69]:

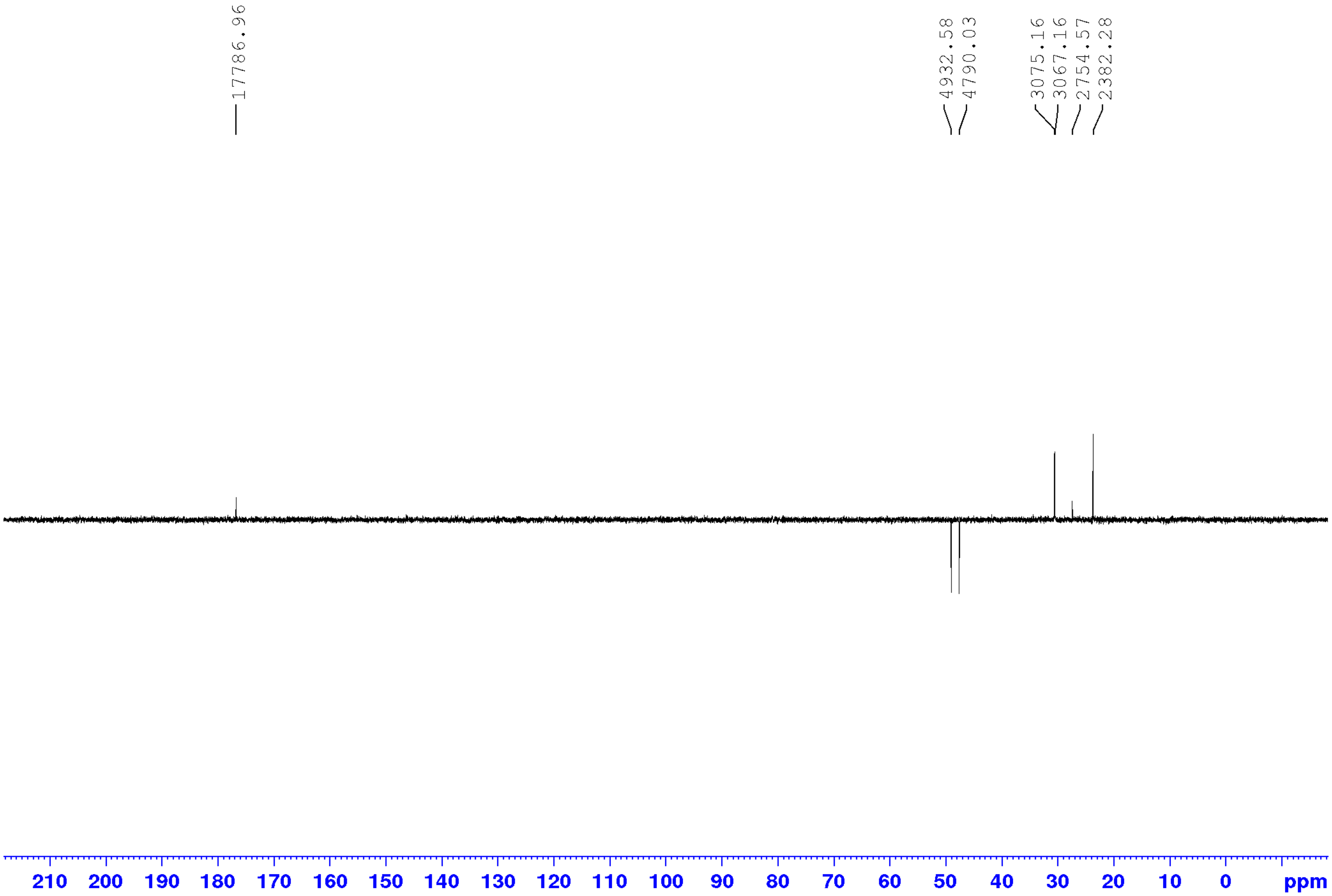

^{1}H NMR of diisopropyl 1-oxo-2-phthalimidoethyl phosphonate (400.27 MHz, $CDCl_3$) (19):

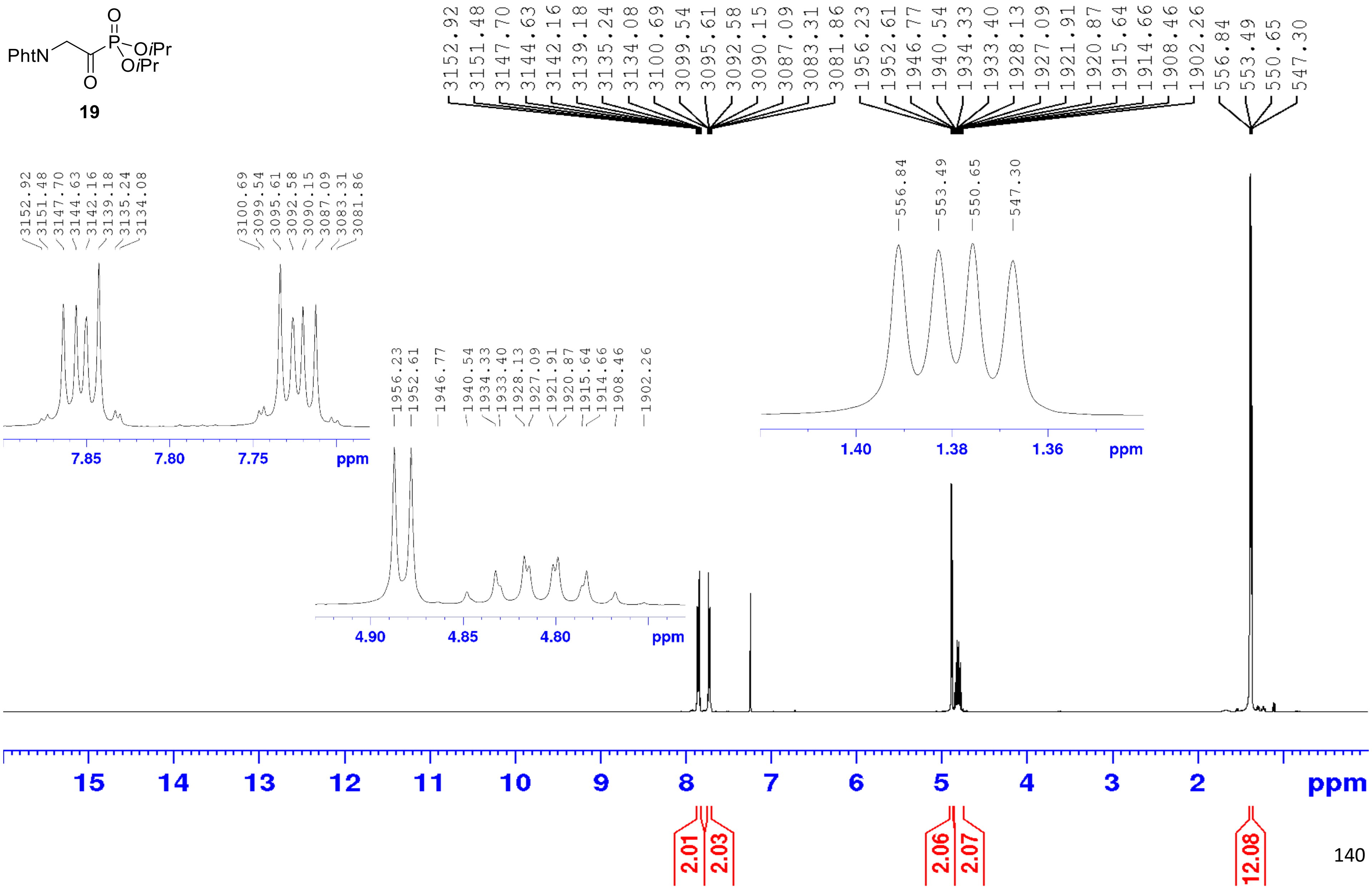

^{31}**P NMR of diisopropyl 1-oxo-2-phthalimidoethyl phosphonate (162.03 MHz, $CDCl_3$) (19):**

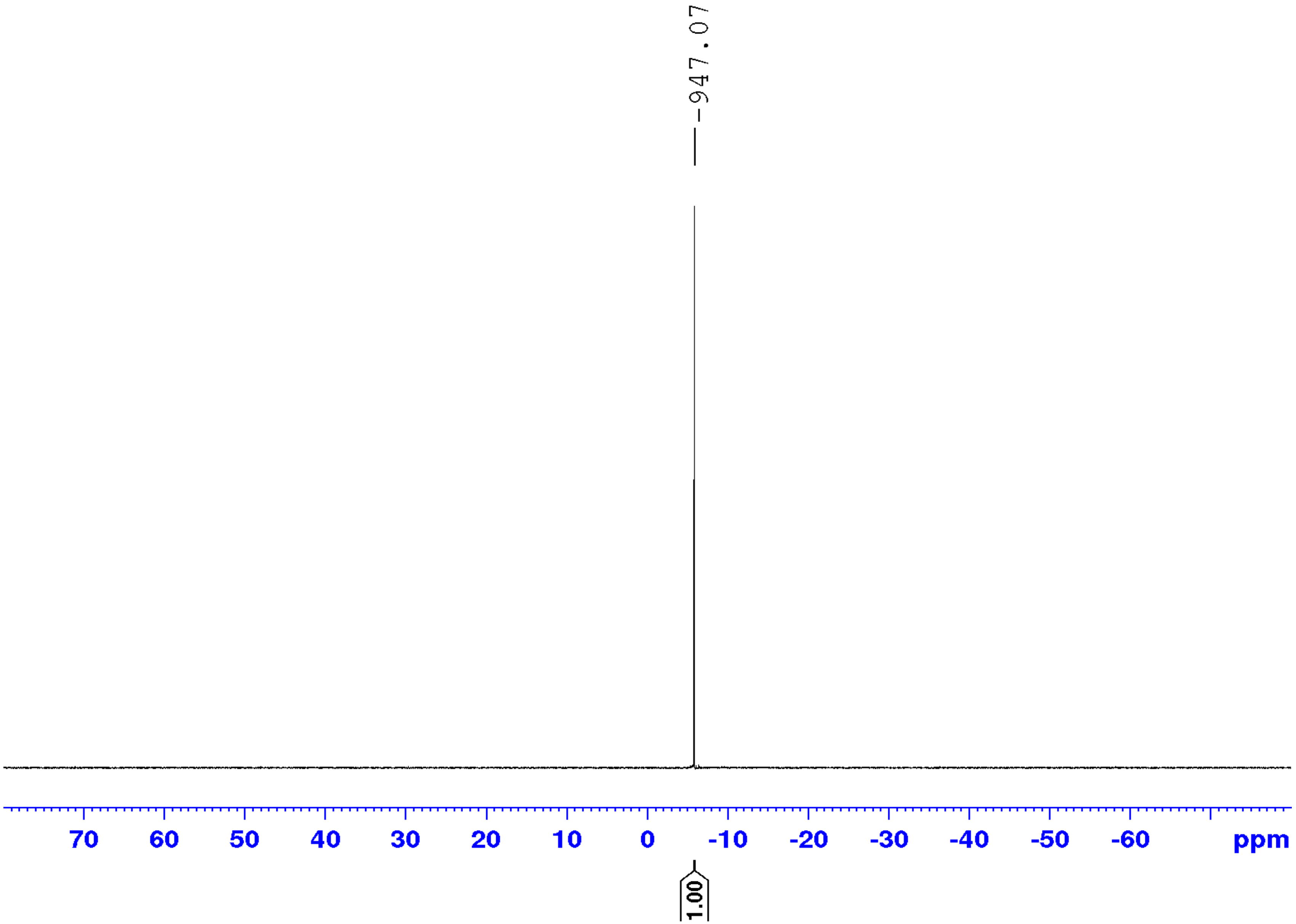

^{1}H NMR of diisopropyl (*R*)-1-[^{2}H]-1-hydroxy-2-phthalimido)-ethylphosphonate (400.27 MHz, CDCl$_3$) {(*R*)-1-[^{2}H]-36}:

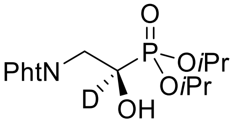

(*R*)-1-[^{2}H]-**36**

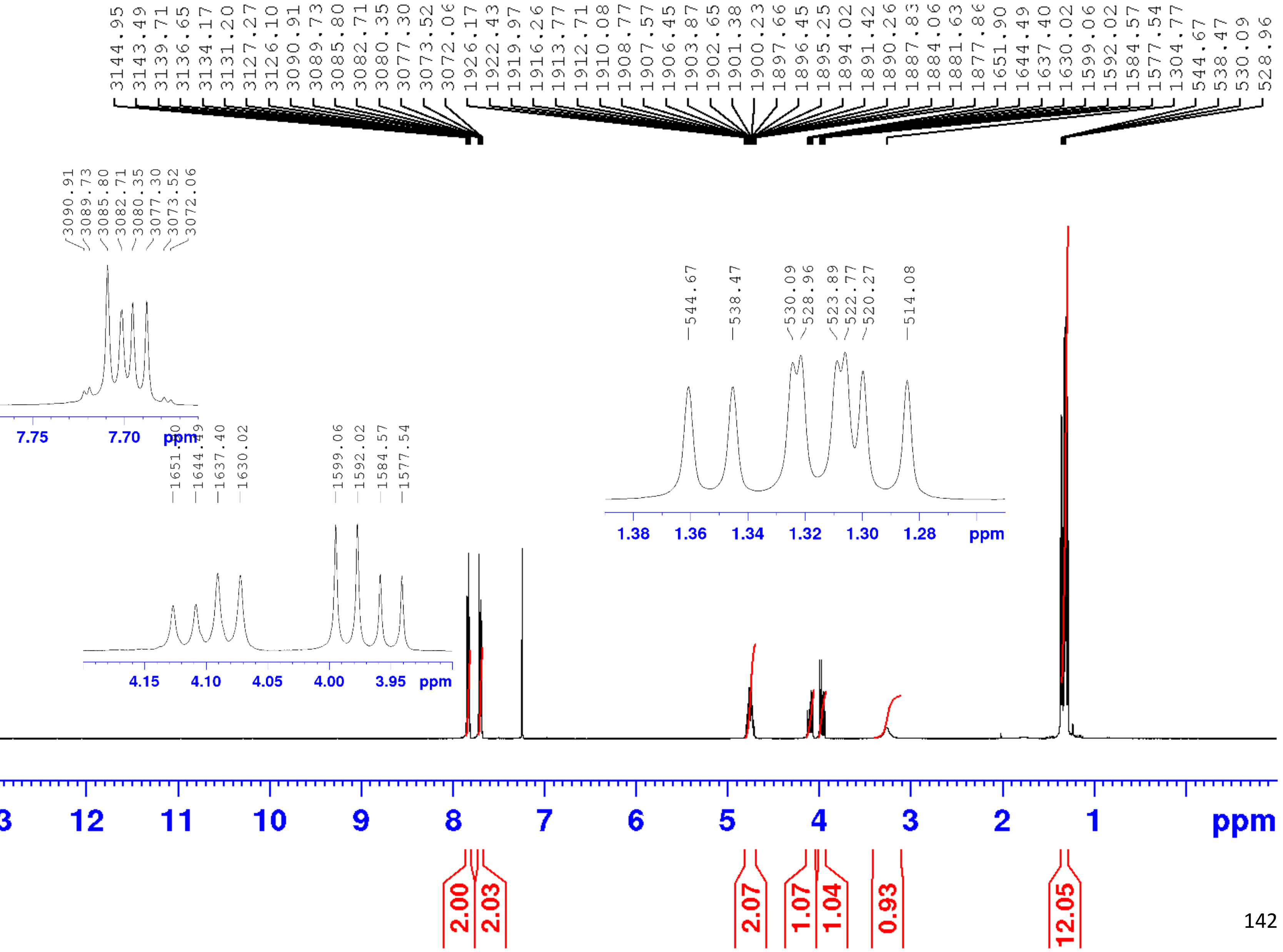

^{31}P NMR of diisopropyl (*R*)-1-[^{2}H]-1-hydroxy-2-phthalimido)-ethylphosphonate (161.98 MHz, $CDCl_3$) {(*R*)-1-[^{2}H]-36}:

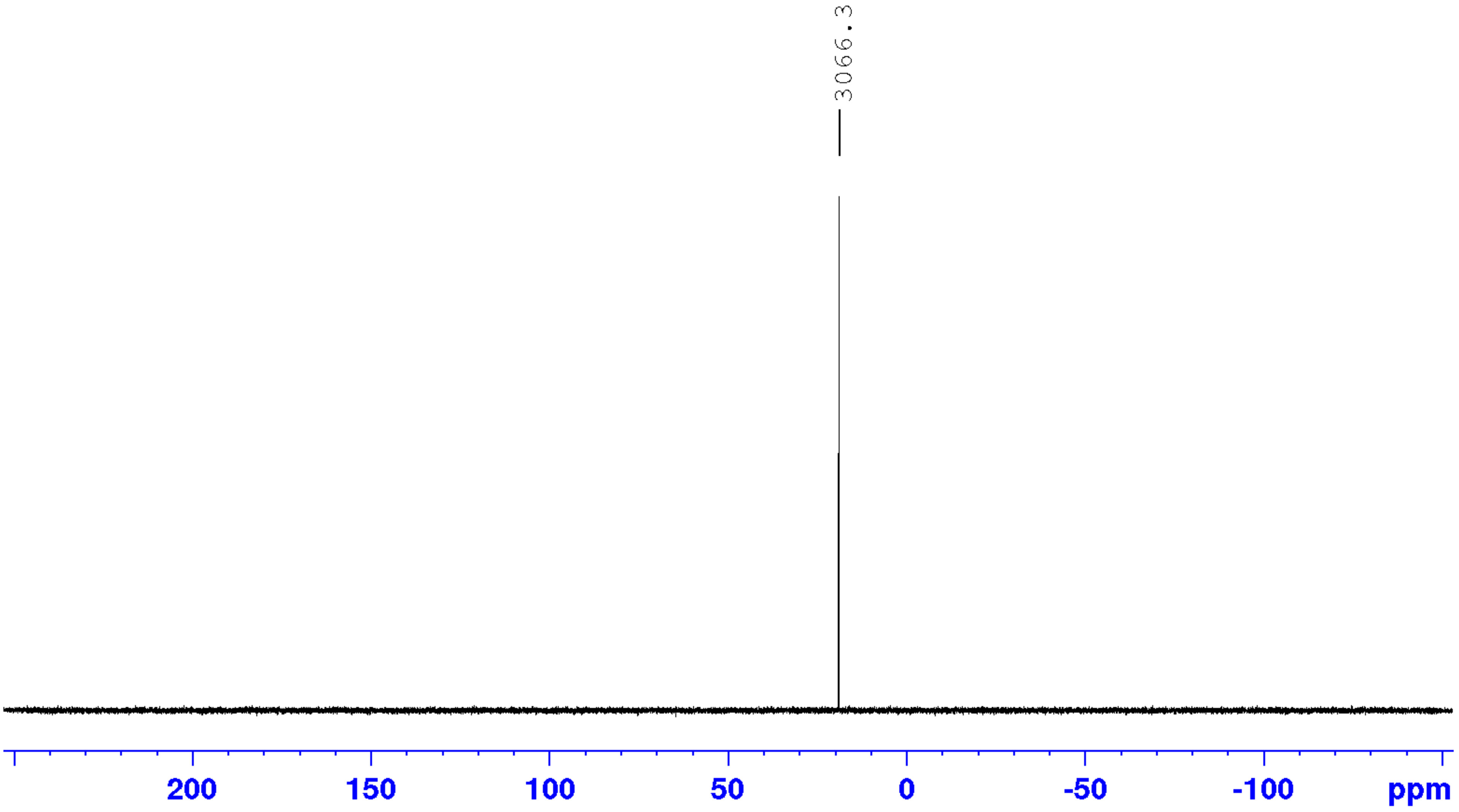

^{13}C NMR of diisopropyl (*R*)-1-[^{2}H]-1-hydroxy-2-phthalimido)-ethylphosphonate (150.93 MHz, $CDCl_3$) {(*R*)-1-[^{2}H]-36}:

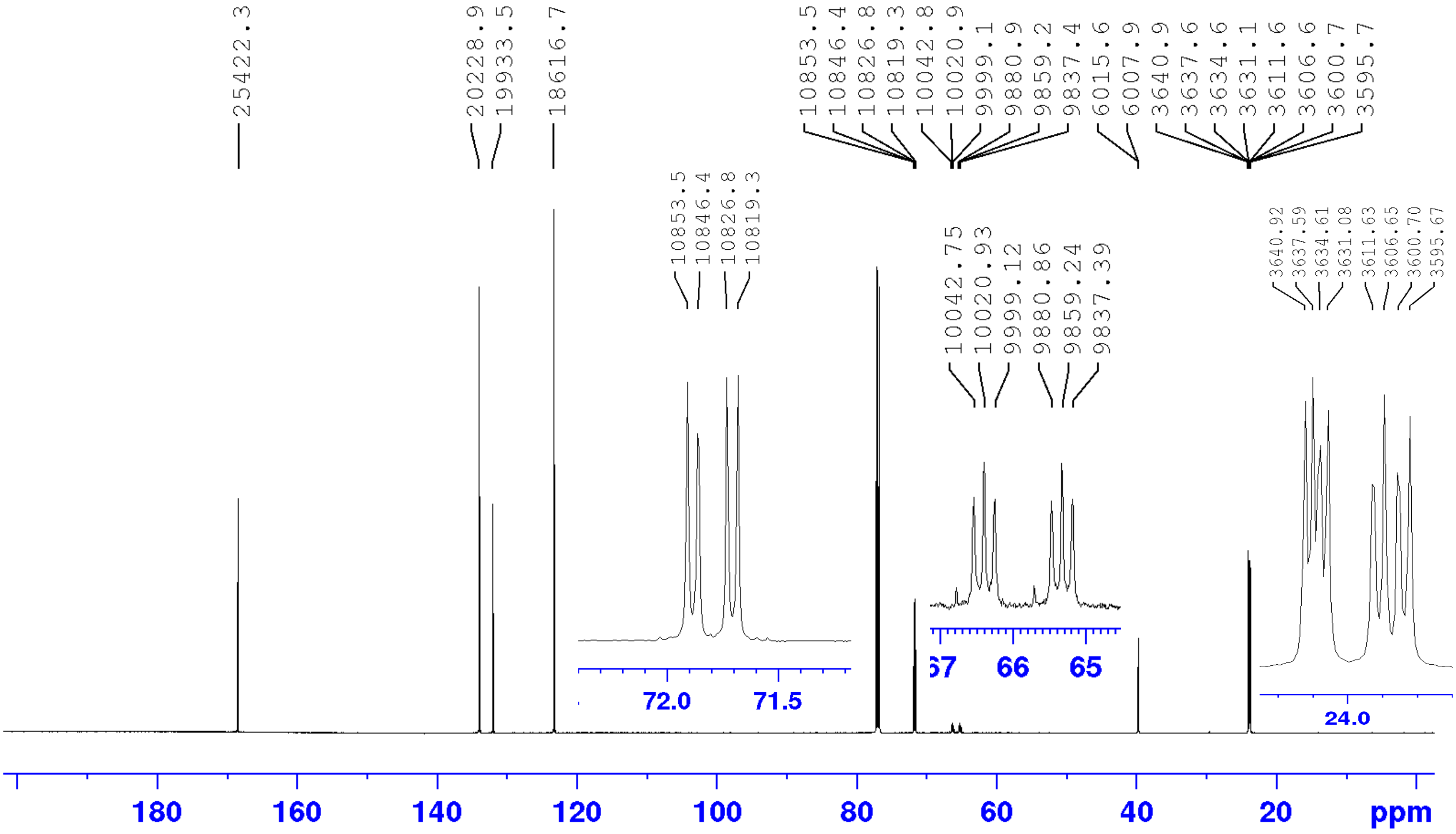

^{1}H NMR of (*R*)-1-[^{2}H]-2-amino-1-hydroxyethylphosphonic acid, (*R*)-1-[^{2}H]-phosphaisoserine (400.27 MHz, D_2O) {(*R*)-1-[^{2}H]-44}:

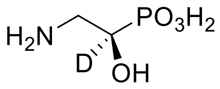

(*R*)-1-[^{2}H]-**44**

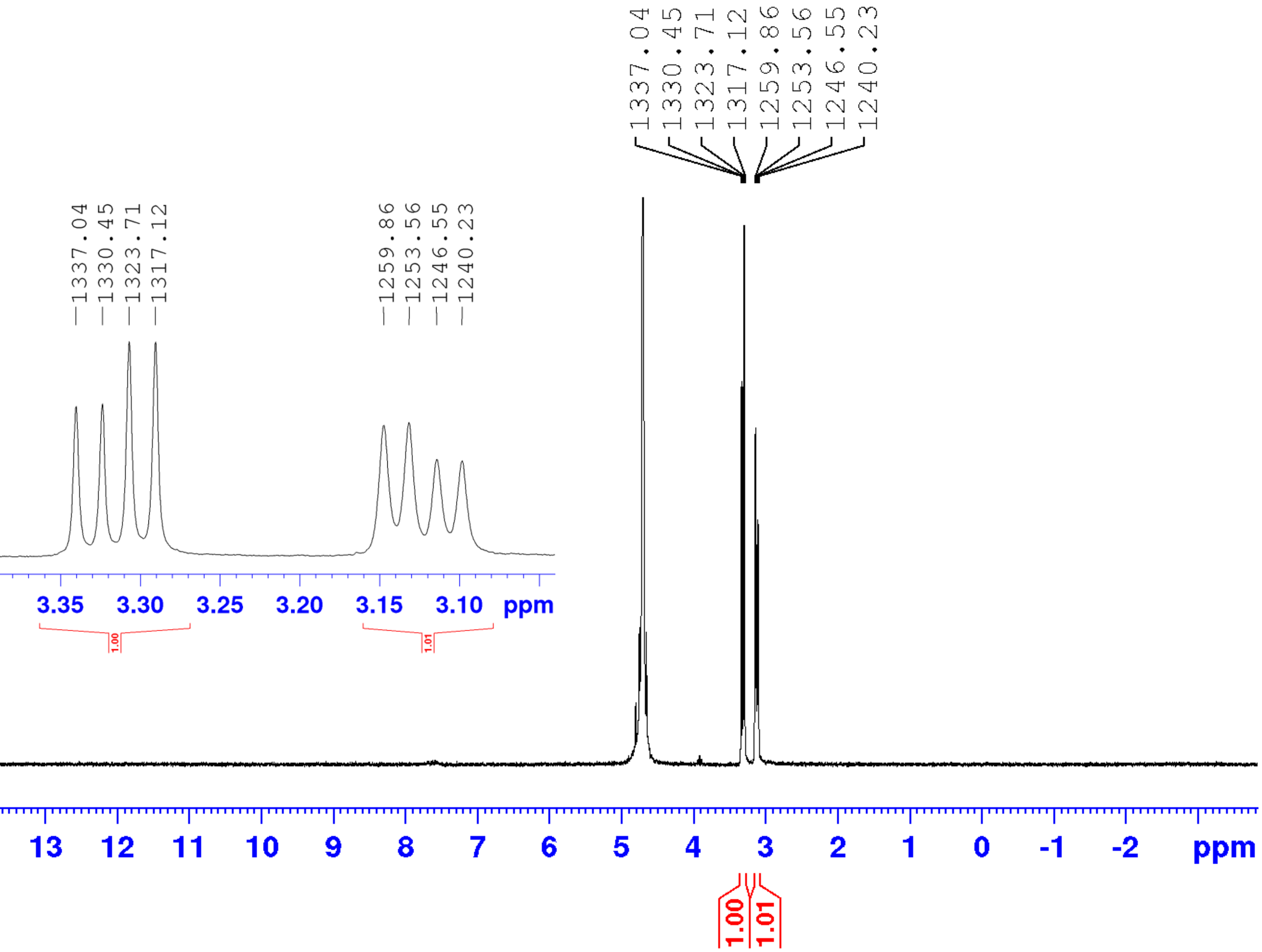

^{31}P NMR of (*R*)-1-[^{2}H]-2-amino-1-hydroxyethylphosphonic acid, (*R*)-1-[^{2}H]-phosphaisoserine {(*R*)-1-[^{2}H]-44} (162.03 MHz, D_2O):

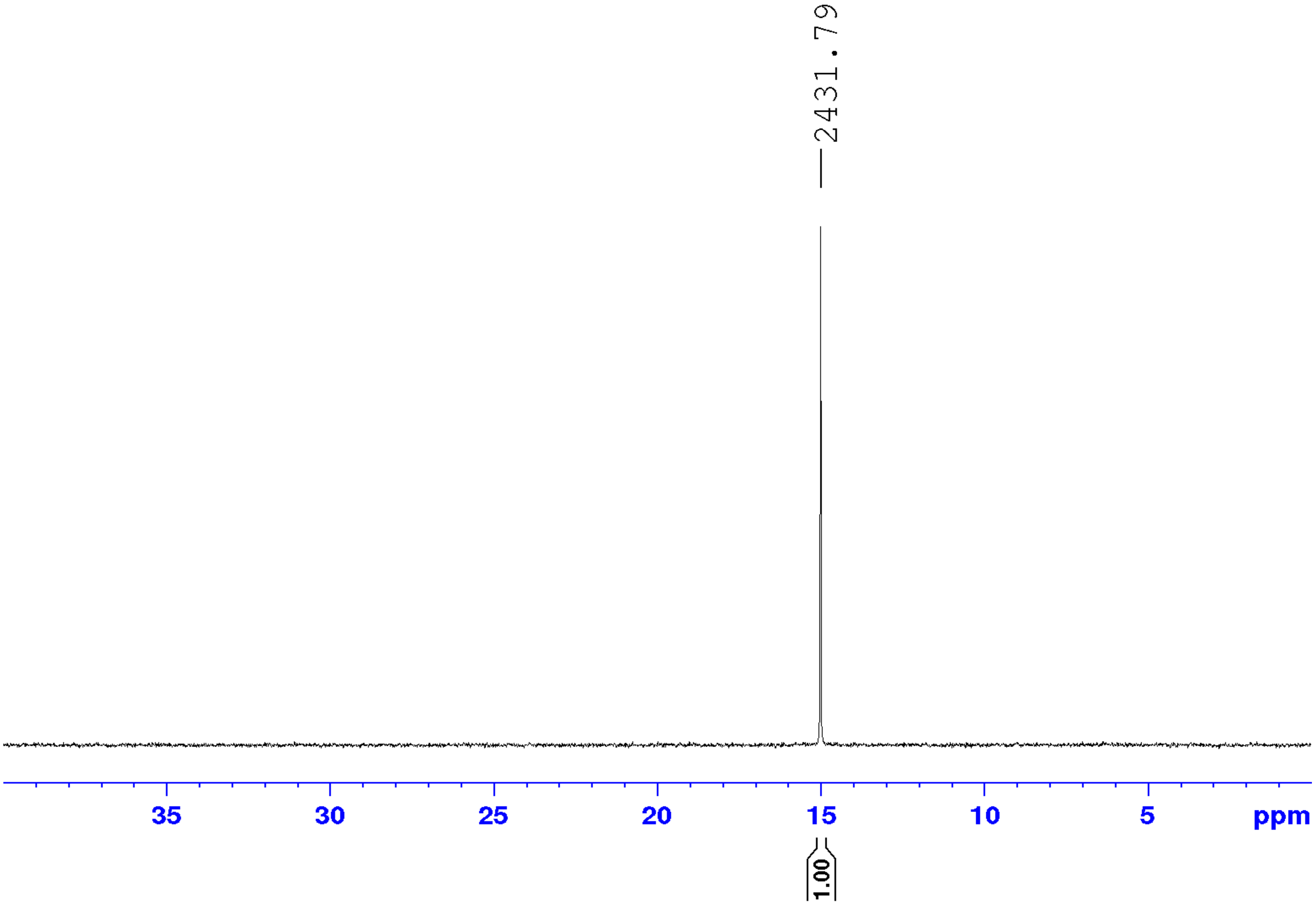

^{13}C NMR of (*R*)-1-[^{2}H]-2-amino-1-hydroxyethylphosphonic acid, (*R*)-1-[^{2}H]-phosphaisoserine {(*R*)-1-[^{2}H]-44} (100.65 MHz, D_2O):

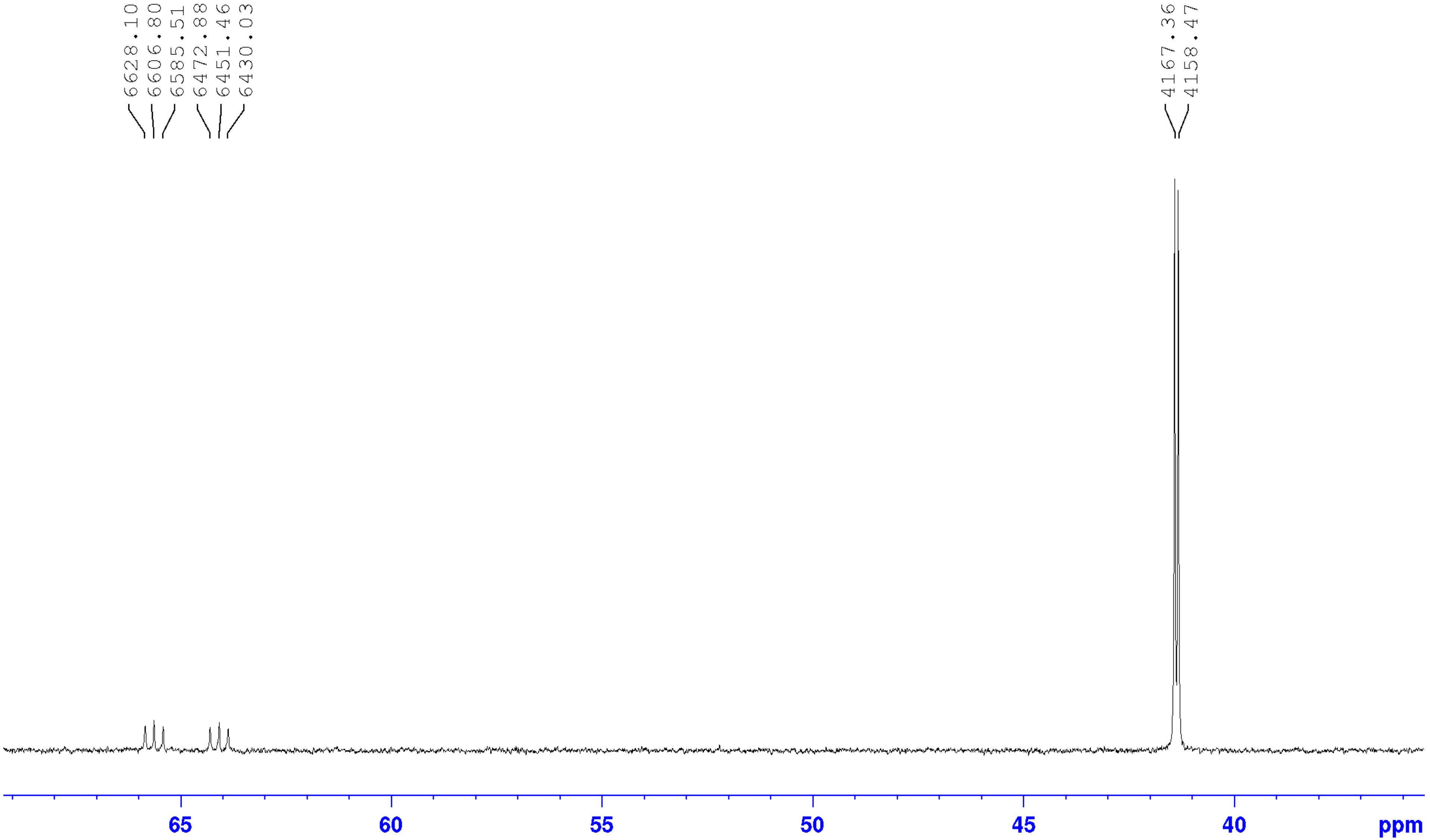

^{1}H NMR of diisopropyl 1-[^{13}C]-1-oxo-2-phthalimidoethyl phosphonate (400.27 MHz, $CDCl_3$) ([^{13}C]-19):

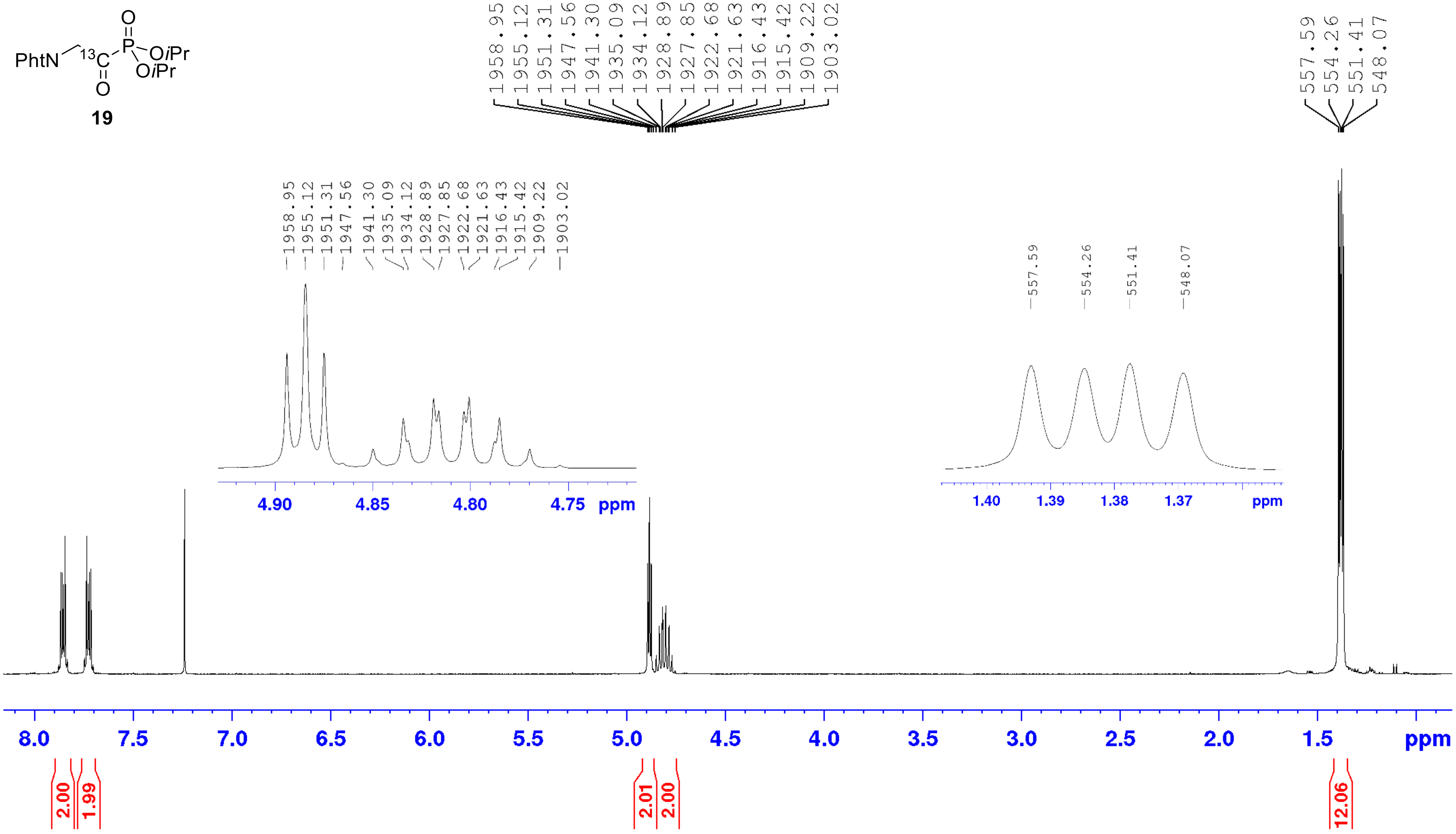

^{31}P NMR of diisopropyl 1-[^{13}C]-1-oxo-2-phthalimidoethyl phosphonate (162.03 MHz, $CDCl_3$) ([^{13}C]-19):

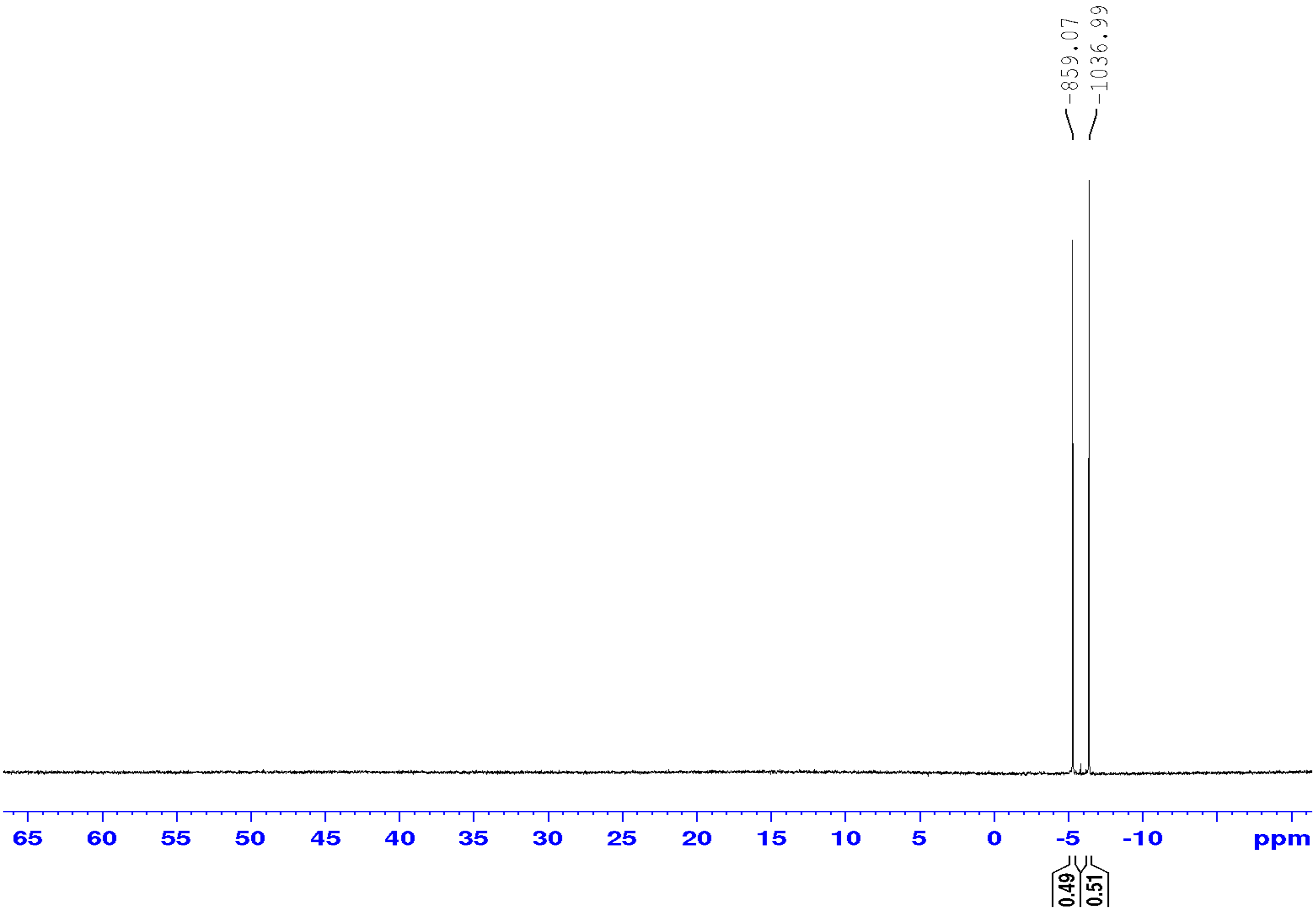

^{1}H NMR of diisopropyl (*R*)-1-[^{13}C]-1-hydroxy-2-phthalimido)-ethylphosphonate (400.27 MHz, $CDCl_3$) {(*R*)-1-[^{13}C]-36}:

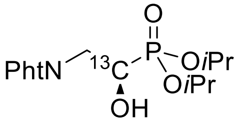

(*R*)-1-[^{13}C]-**36**

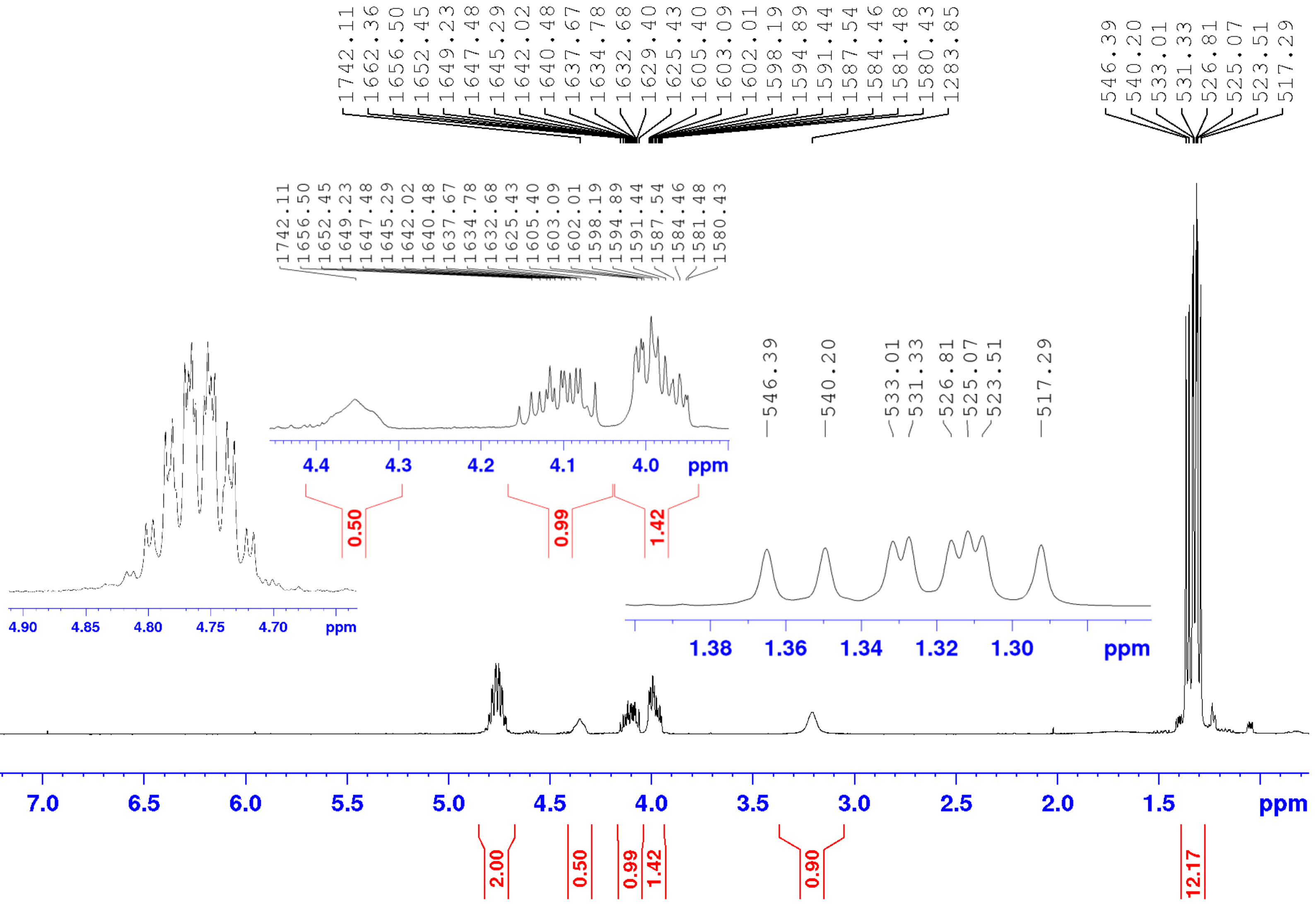

^{31}P NMR of diisopropyl (*R*)-1-[^{13}C]-1-hydroxy-2-phthalimido)-ethylphosphonate (162.03 MHz, $CDCl_3$) {(*R*)-1-[^{13}C]-36}:

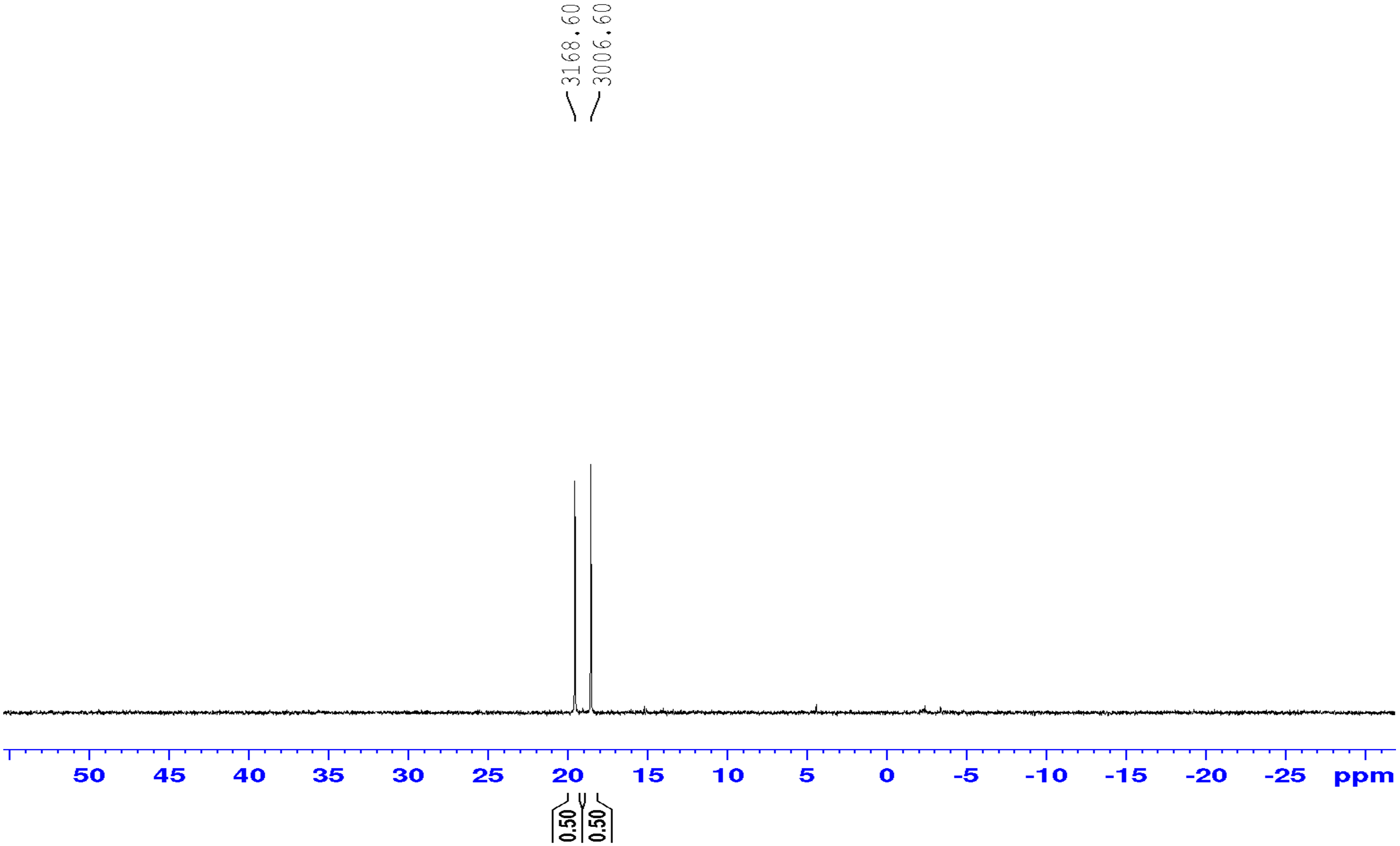

^{1}H NMR of (*R*)-1-[^{13}C]-2-amino-1-hydroxyethylphosphonic acid, (*R*)-1-[^{13}C]-phosphaisoserine (600.25 MHz, D_2O) {(*R*)-1-[^{13}C]-44}:

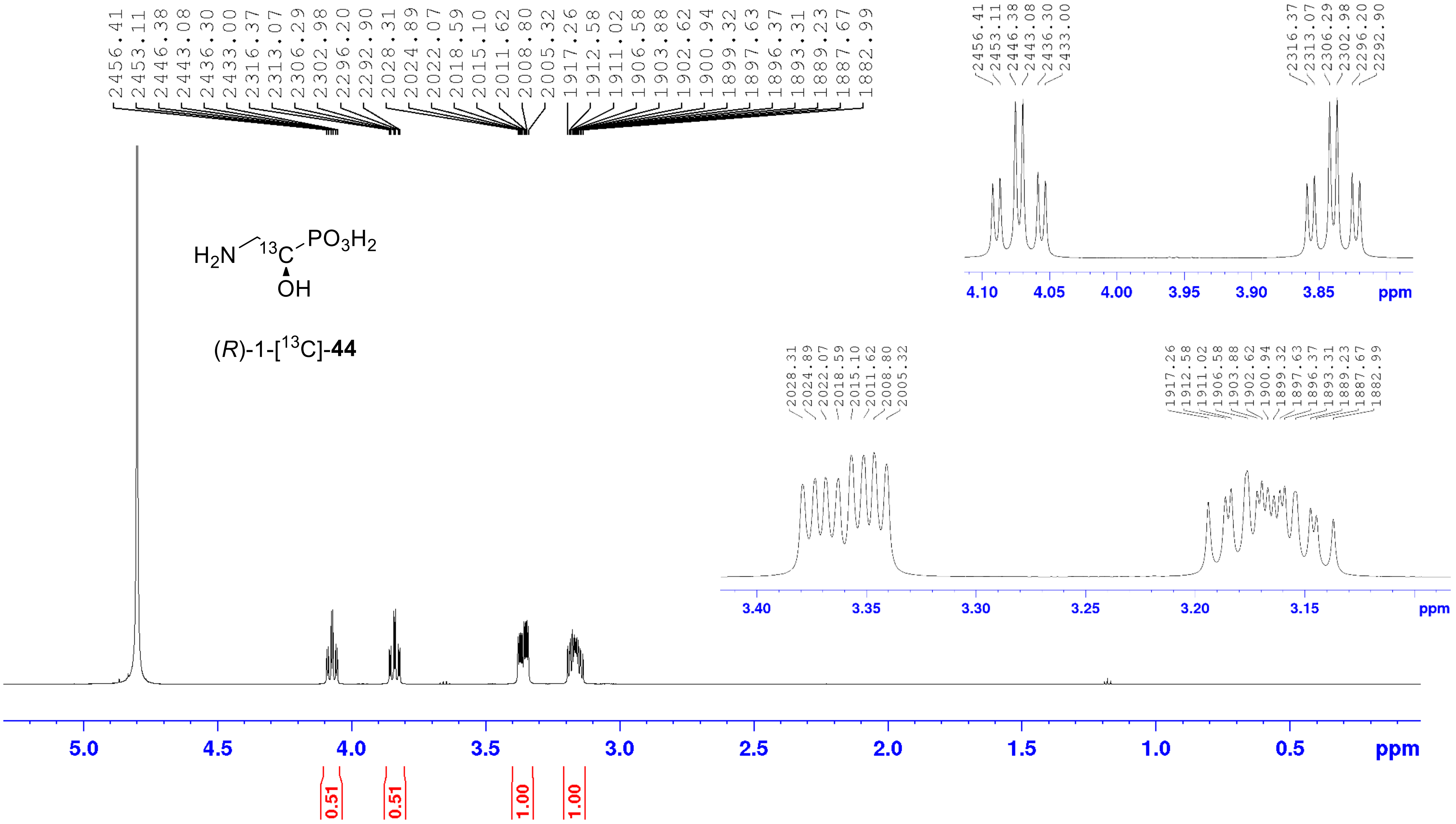

^{31}P NMR of (*R*)-1-[^{13}C]-2-amino-1-hydroxyethylphosphonic acid, (*R*)-1-[^{13}C]-phosphaisoserine (242.99 MHz, D_2O) {(*R*)-1-[^{13}C]-44}:

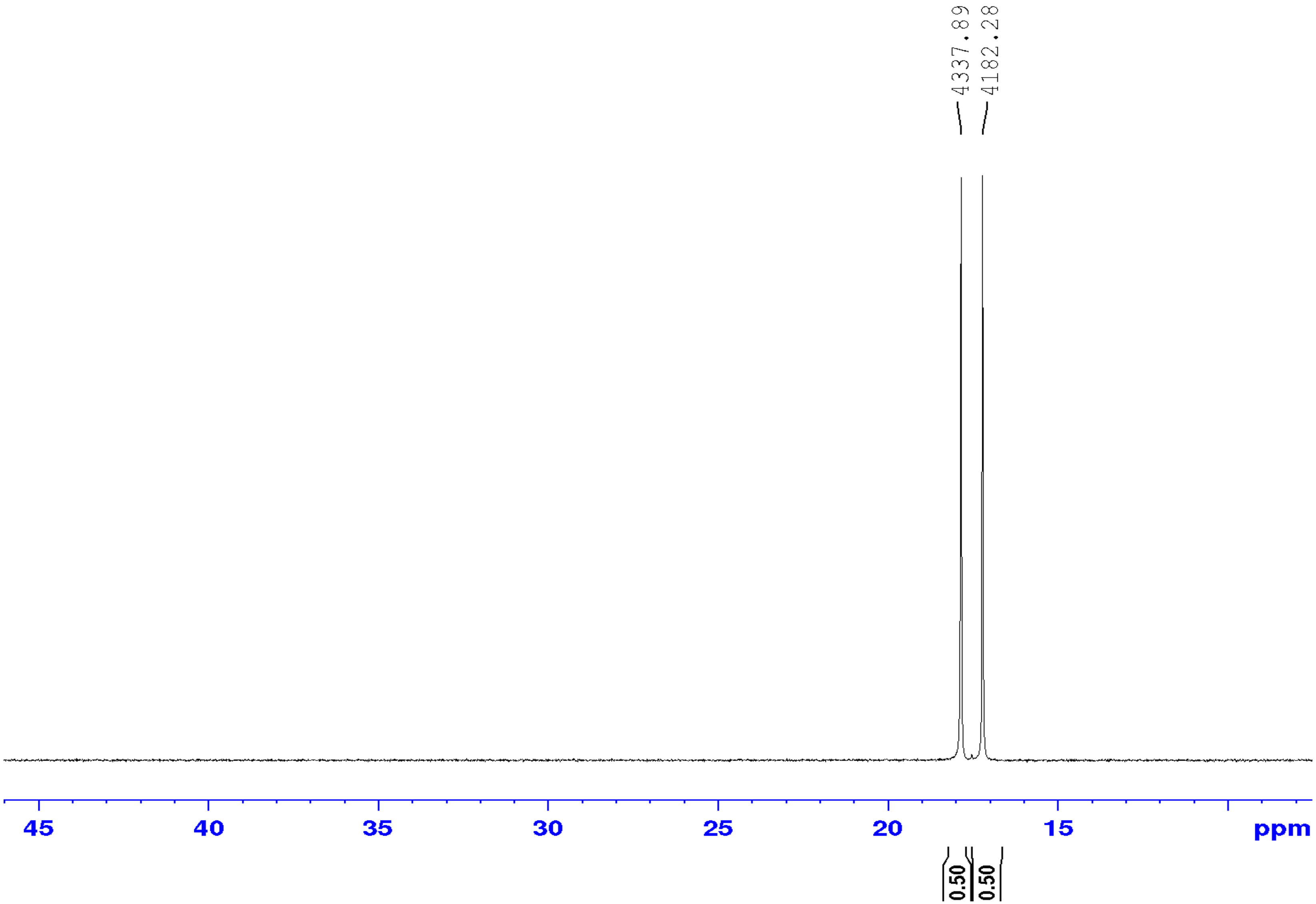

^{13}C NMR of (*R*)-1-[^{13}C]-2-amino-1-hydroxyethylphosphonic acid, (*R*)-1-[^{13}C]-phosphaisoserine (150.93 MHz, D_2O) {(*R*)-1-[^{13}C]-44}:

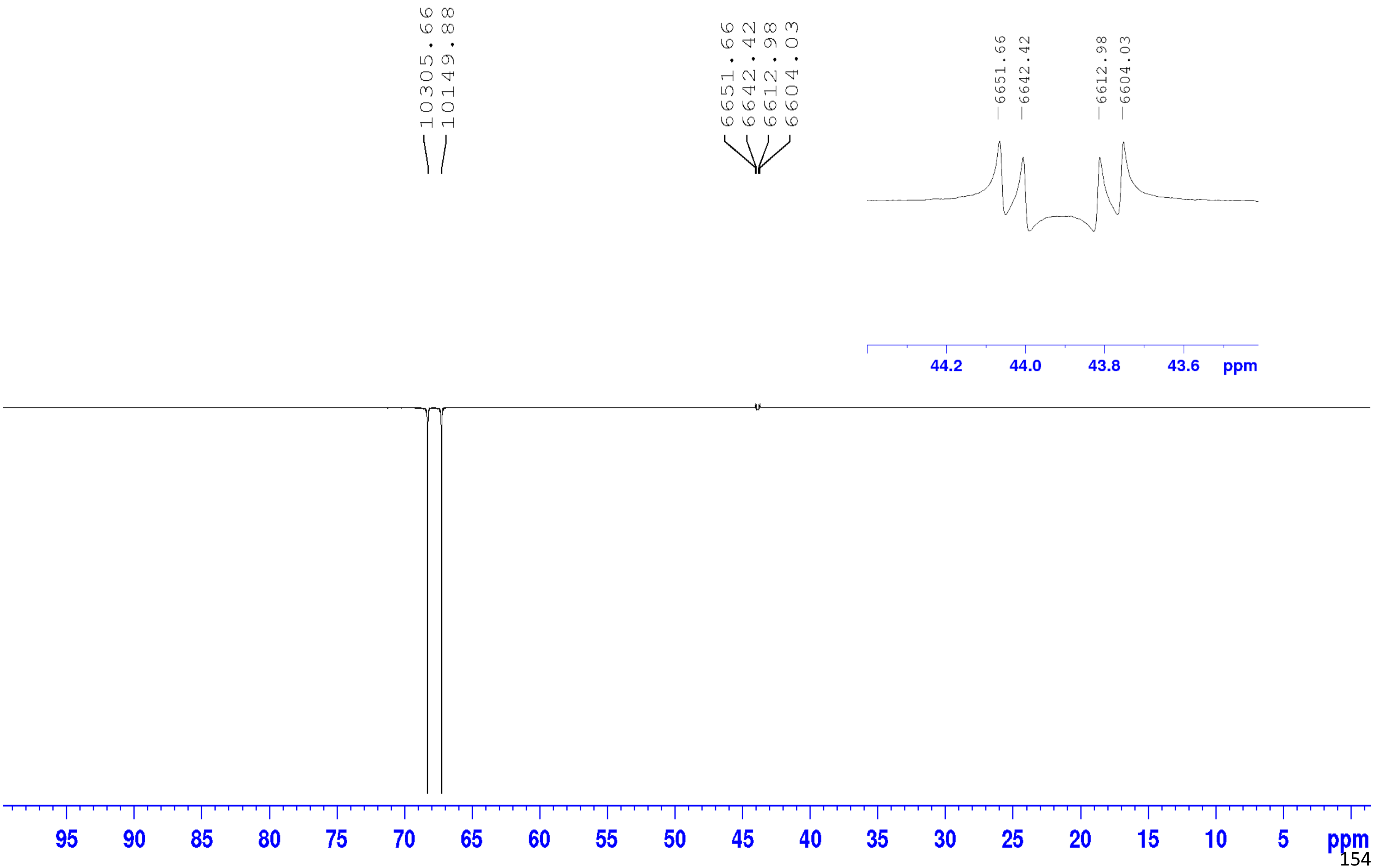

^{1}H NMR of diisopropyl (*R*)-1-[^{2}H]-1-[^{13}C]-1-hydroxy-2-phthalimido)-ethylphosphonate (400.27 MHz, $CDCl_3$) {(*R*)-1-[^{2}H]-1-[^{13}C]-36}:

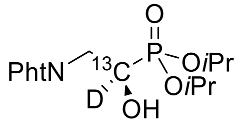

(*R*)-1-[^{2}H]-1-[^{13}C]-**36**

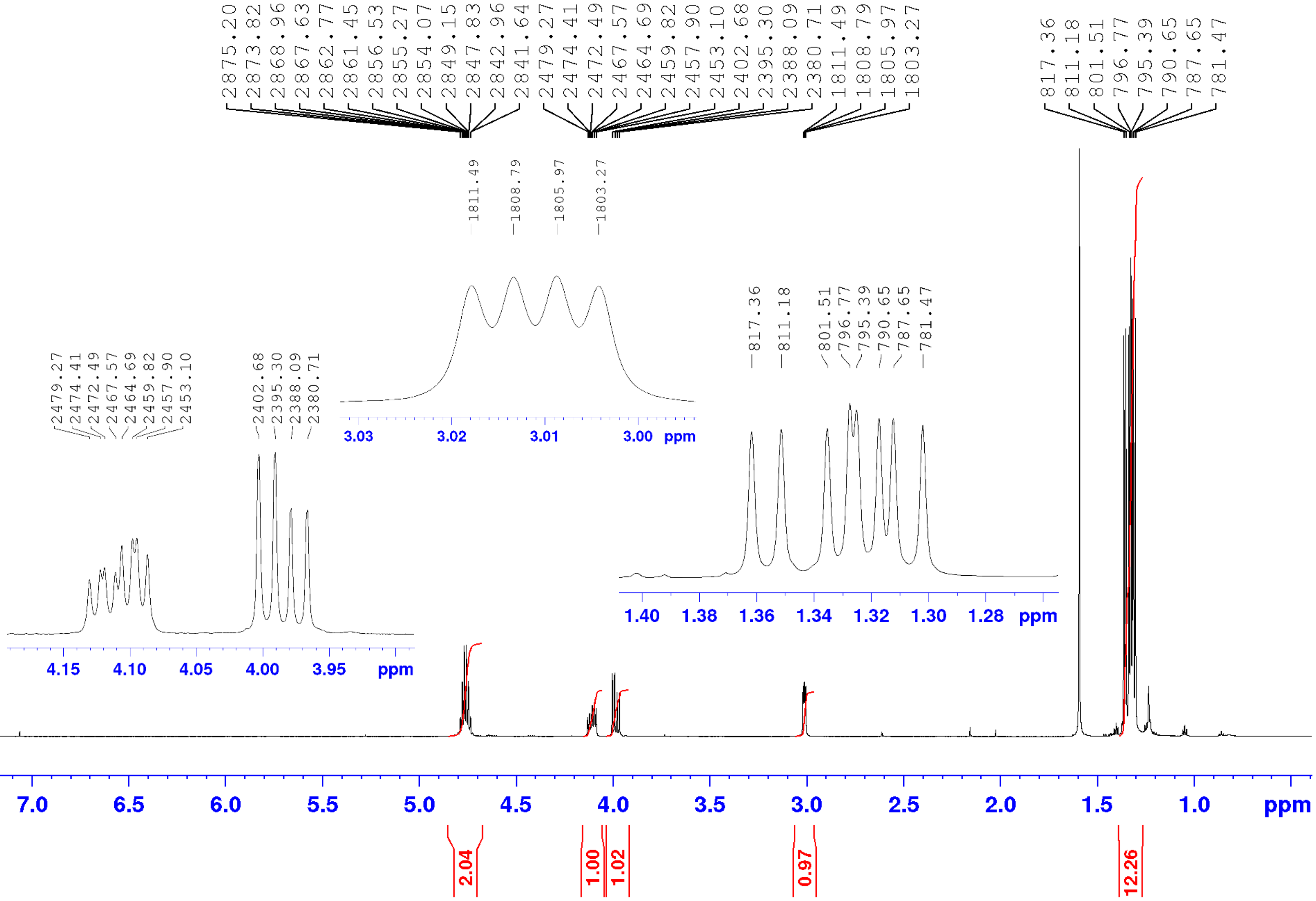

^{31}P NMR of diisopropyl (*R*)-1-[^{2}H]-1-[^{13}C]-1-hydroxy-2-phthalimido)-ethylphosphonate (162.03 MHz, $CDCl_3$) {(*R*)-1-[^{2}H]-1-[^{13}C]-36}:

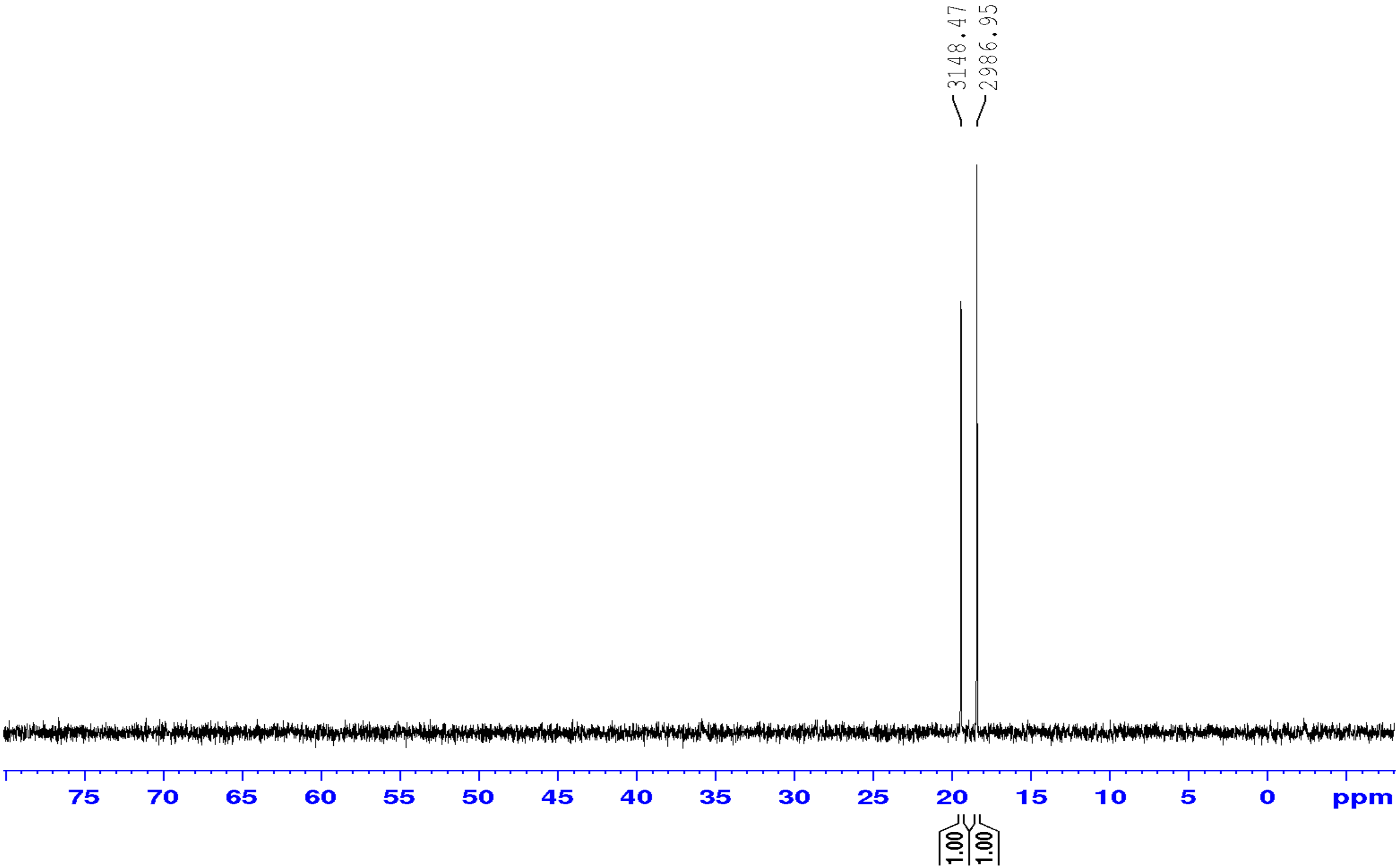

^{13}C NMR of diisopropyl (*R*)-1-[^{2}H]-1-[^{13}C]-1-hydroxy-2-phthalimido)-ethylphosphonate (150.93 MHz, $CDCl_3$) {(*R*)-1-[^{2}H]-1-[^{13}C]-36}:

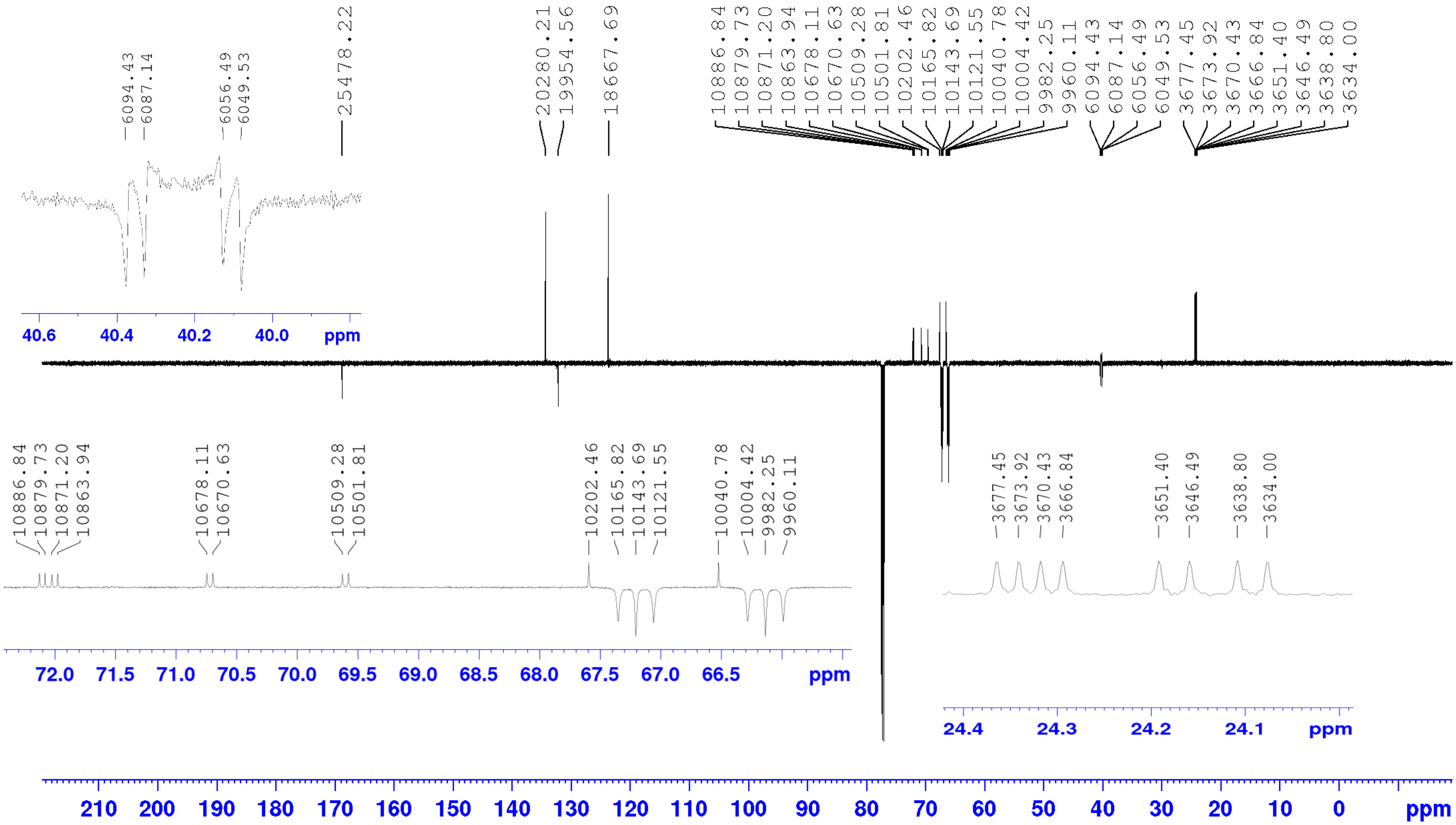

Degree of (*R*)-1-[^{2}H]-1-[^{13}C]-36, additional NMR experiments:

As the above shown ^{13}C NMR spectrum hints at a significant amount of un-deuterated byproduct (67.06 ppm, d, $^{2}J_{CP}$ = 161.68), additional NMR experiments were performed.

HSQC spectra: ^{13}C-decoupled (red) and **HSQC, ^{13}C-coupled** (violet)

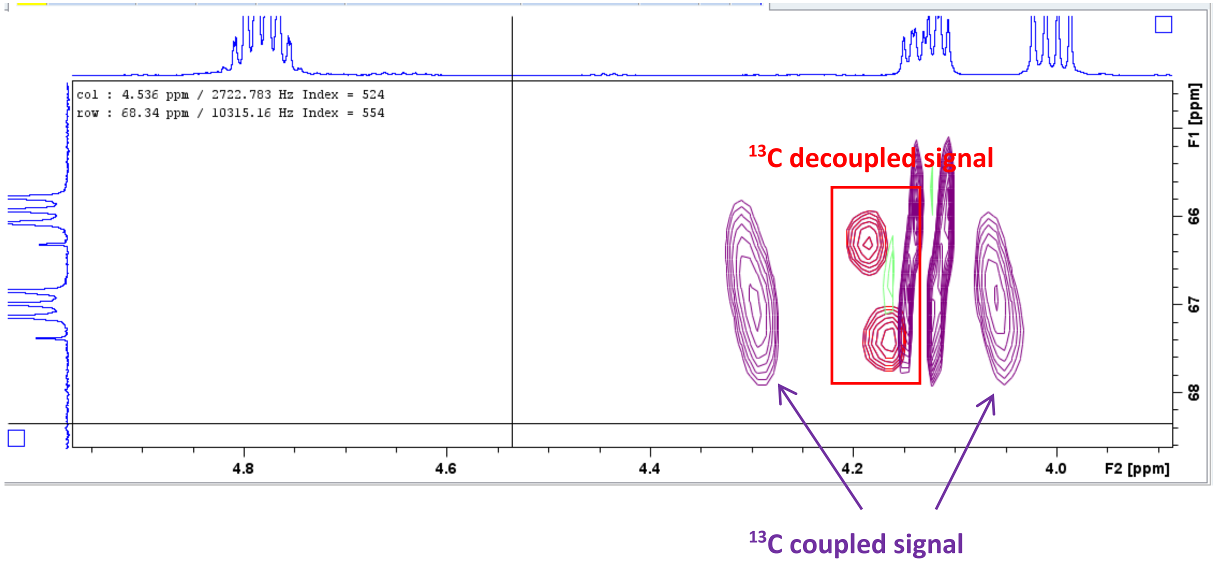

It can be clearly seen that the duplet at 67.06 ppm couples in a region of the ^{1}H NMR with very low signal intensity.

Increasing the spectrum intensity of this region reveals the protons attached to this ^{13}C-atom to be the following:

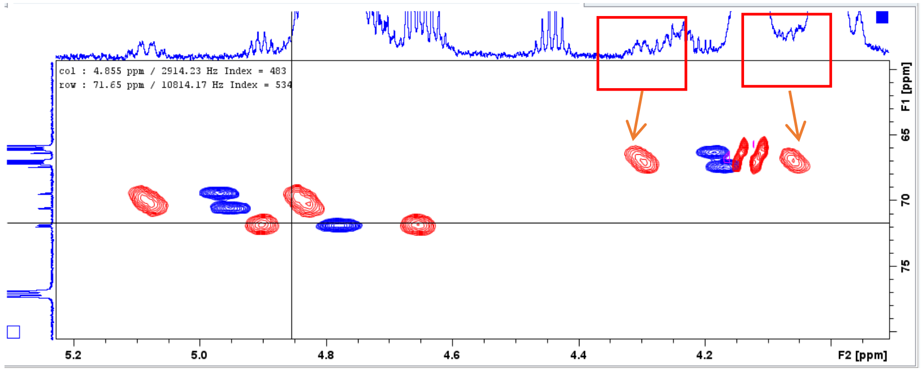

One half of this signal cannot be integrated due to an overlap with other signals. However, the second half is clearly visible (**red**). Comparison of the signal intensity to the ^{13}C-satelites of other proton signals (**green**) reveals it to be derived from a compound that is approximately 1mol% or less of the total mixture.

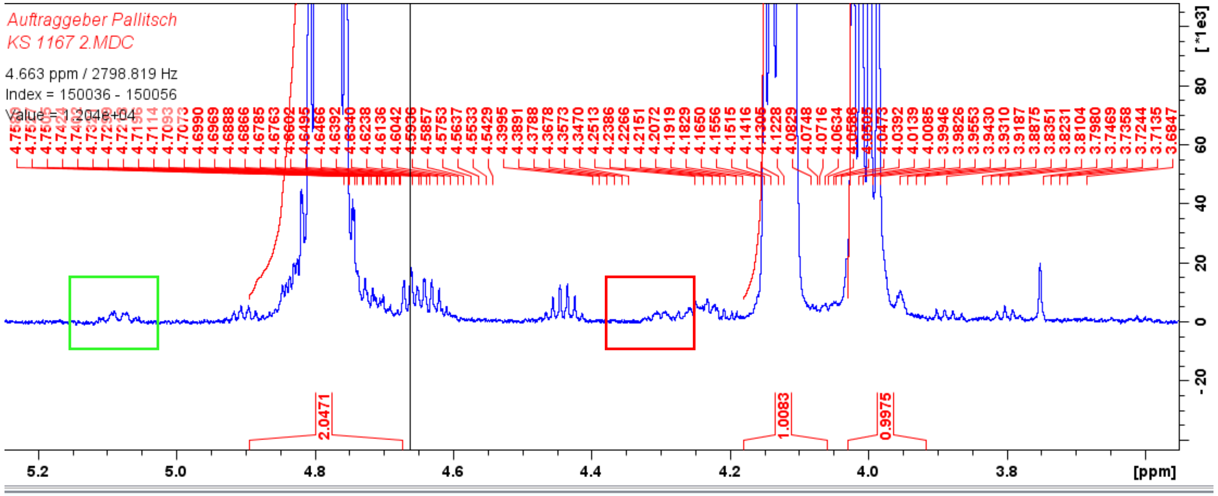

<u>→ degree of deuteration ≥ 99%!</u>

^{1}H NMR of (*R*)-1-[^{2}H]-1-[^{13}C]-2-amino-1-hydroxyethylphosphonic acid, *R*)-1-[^{2}H]-1-[^{13}C]-phosphaisoserine (600.27 MHz, D_2O) {(*R*)-1-[^{2}H]-1-[^{13}C]-44}:

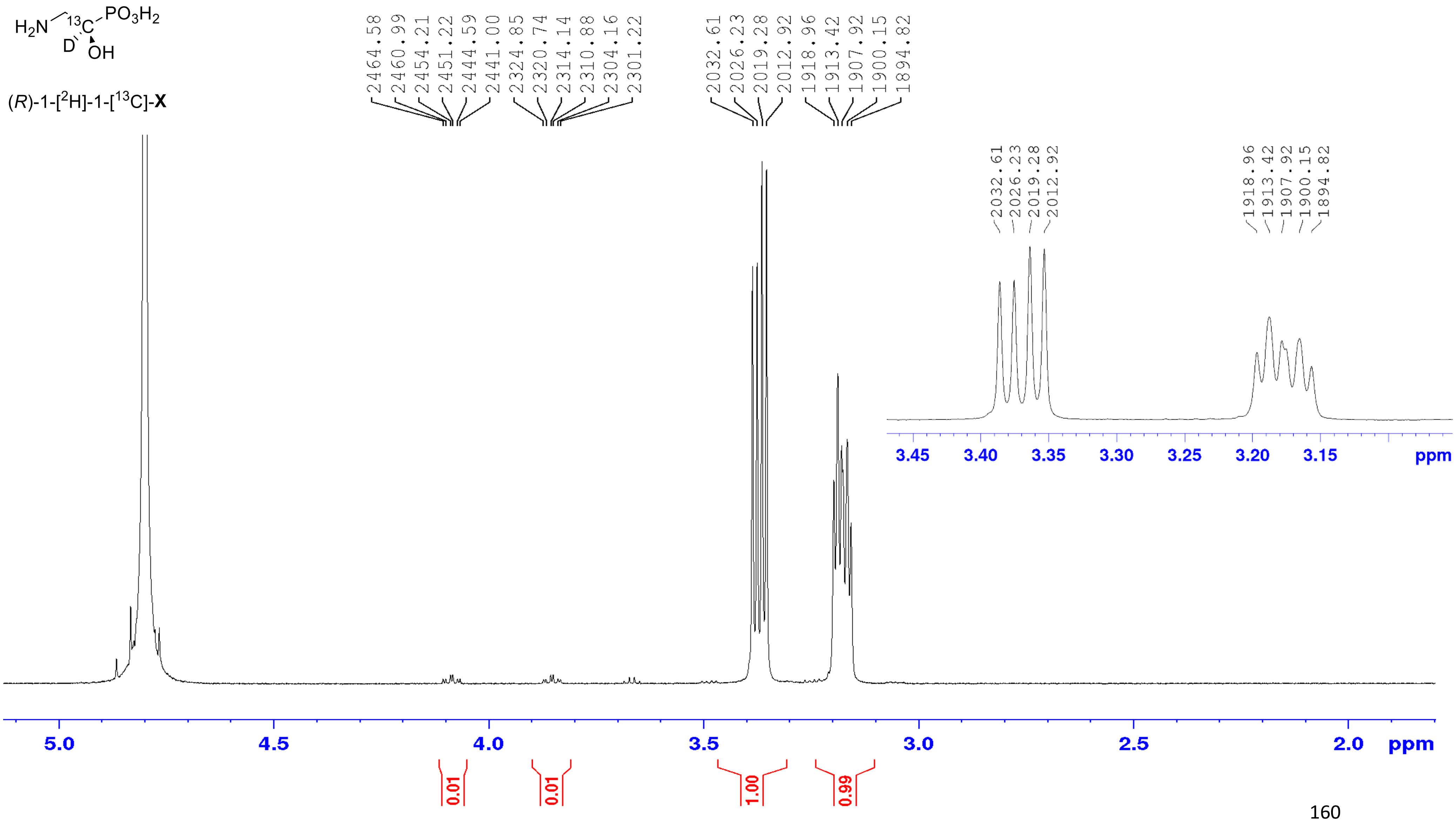

^{31}P NMR of *R*)-1-[^{2}H]-1-[^{13}C]-2-amino-1-hydroxyethylphosphonic acid, (*R*)-1-[^{2}H]-1-[^{13}C]-phosphaisoserine (162.03 MHz, D_2O) {(*R*)-1-[^{2}H]-1-[^{13}C]-44}:

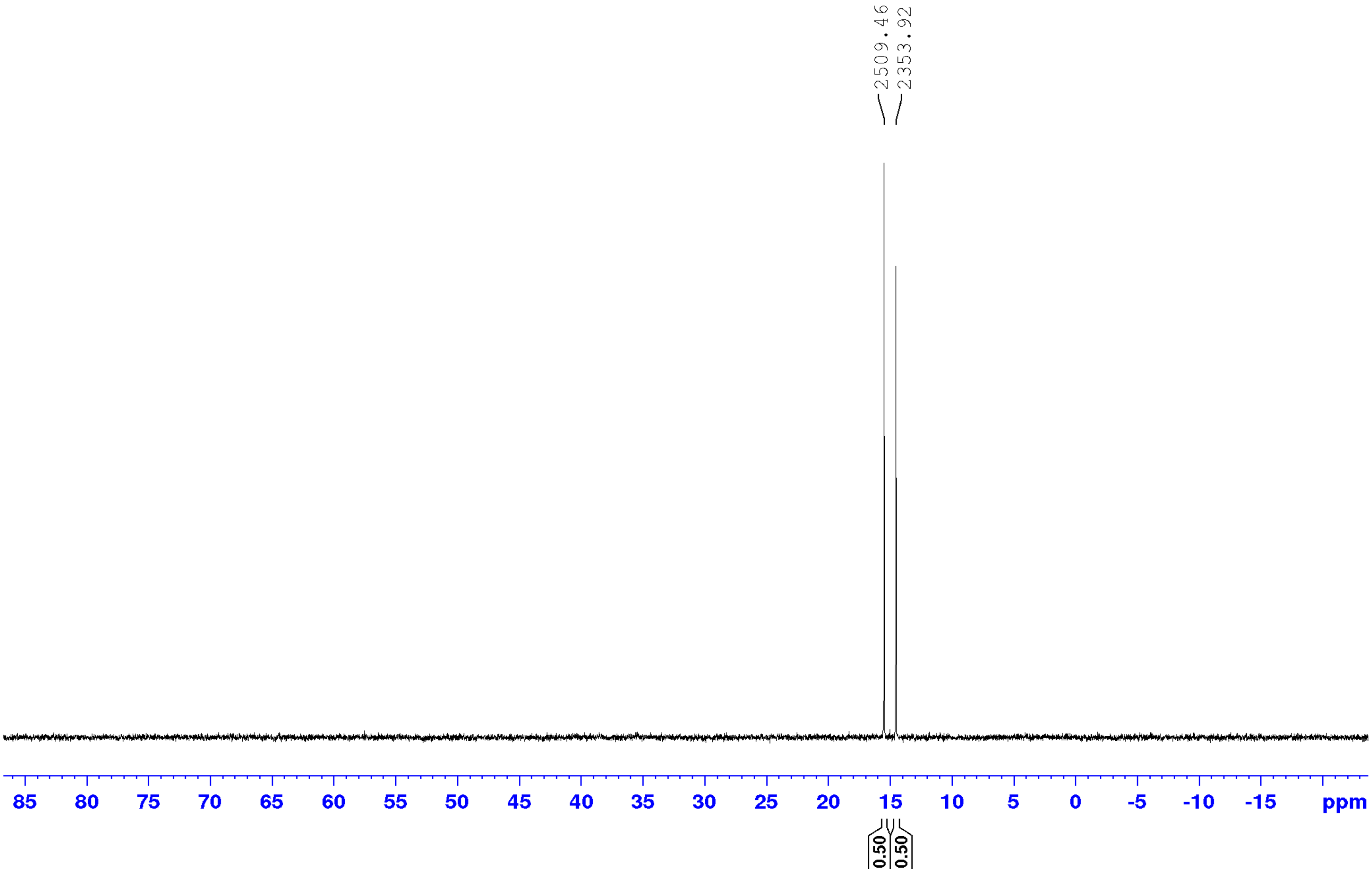

^{13}C NMR of (*R*)-1-[^{2}H]-2-amino-1-hydroxyethylphosphonic acid, (*R*)-1-[^{2}H]-1-[^{13}C]-phosphaisoserine (150.93 MHz, D_2O) {(*R*)-1-[^{2}H]-1-[^{13}C]-44}:

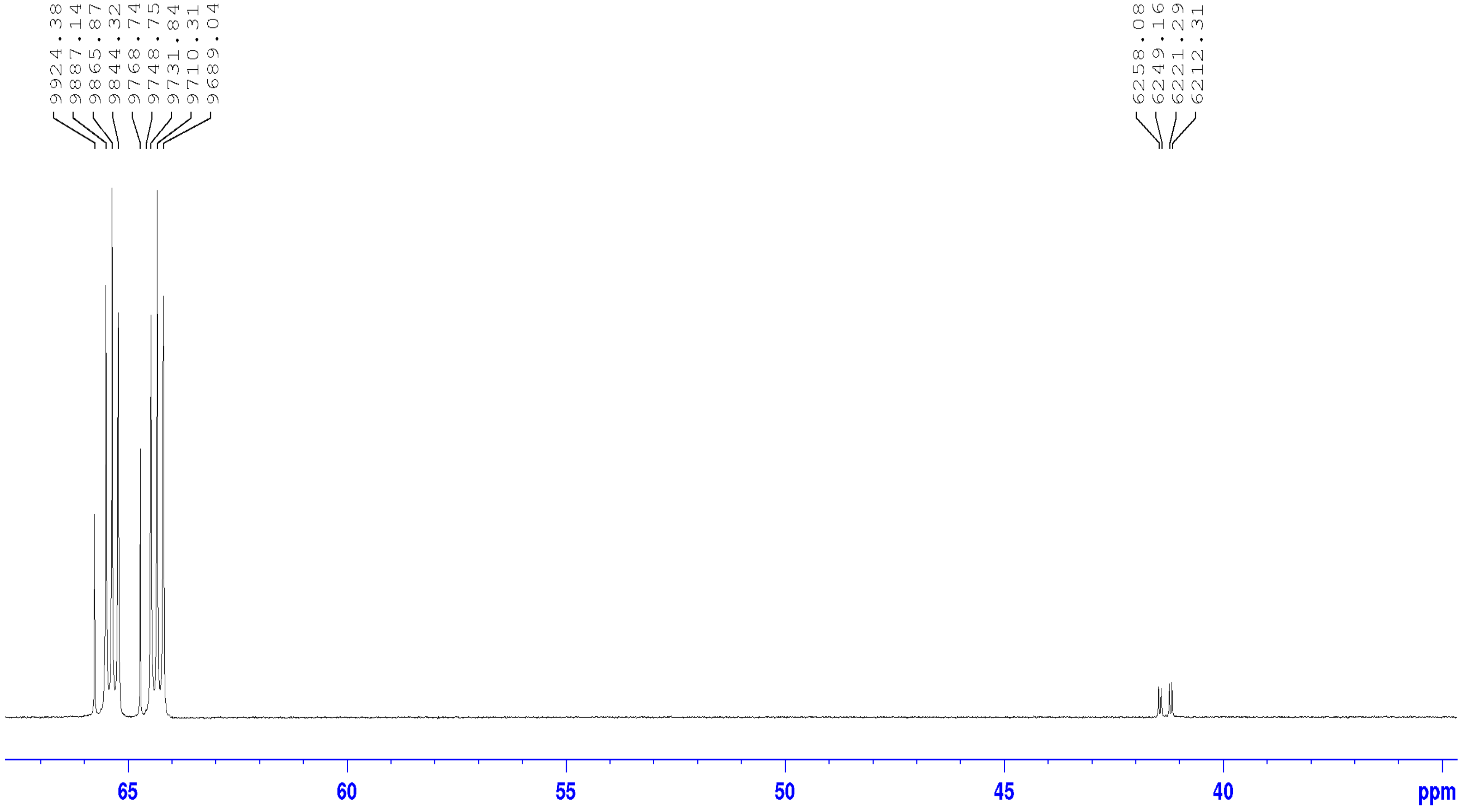

^{31}P NMR of diisopropyl (4-(1,3-dioxoisoindolin-2-yl)butanoyl)phosphonate (CDCl3, 162.04 MHz) (20):

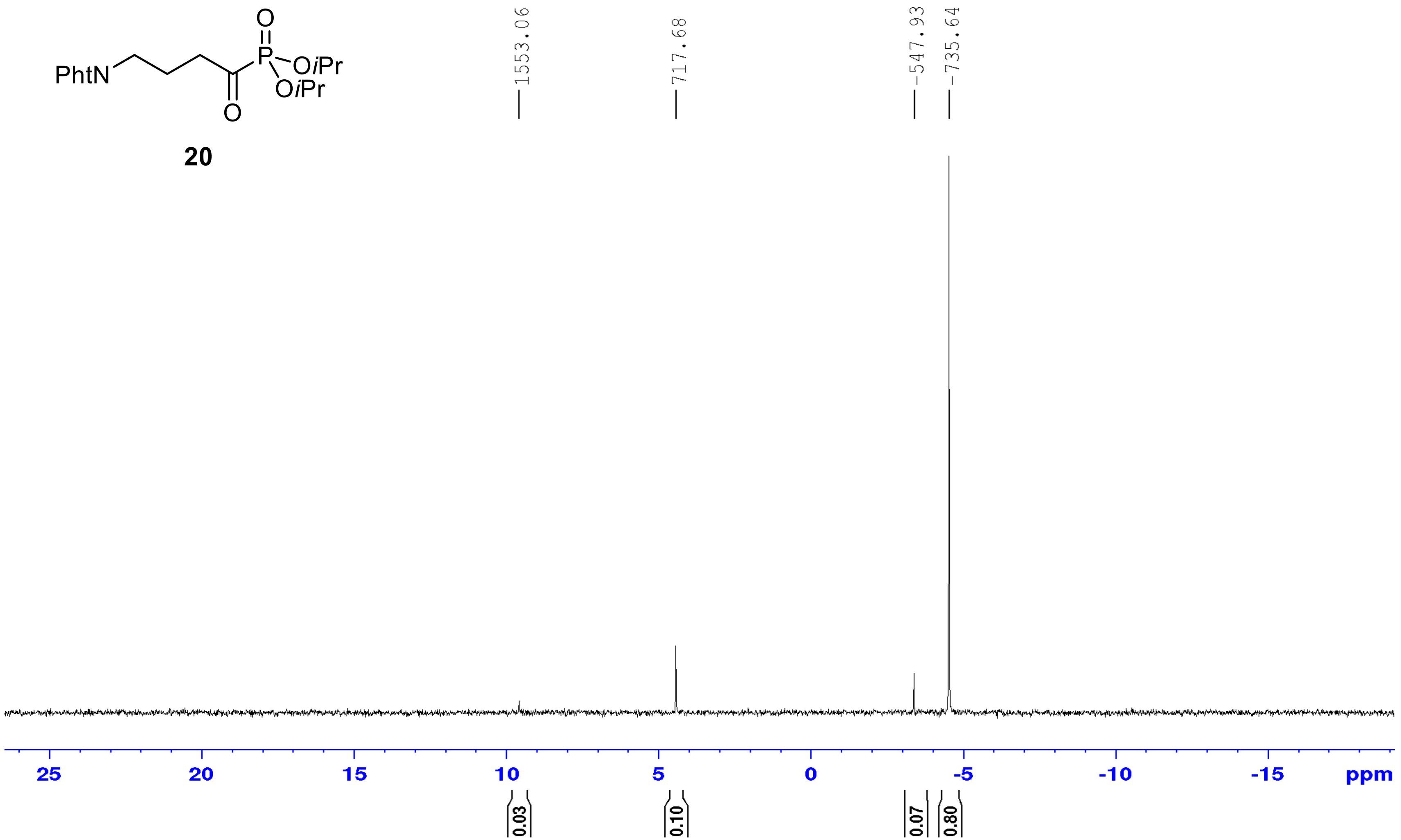

^{1}H NMR of (*S*)-diisopropyl (4-(1,3-dioxoisoindolin-2-yl)butanoyl)phosphonate ($CDCl_3$, 400.27 MHz) [(S)-37]:

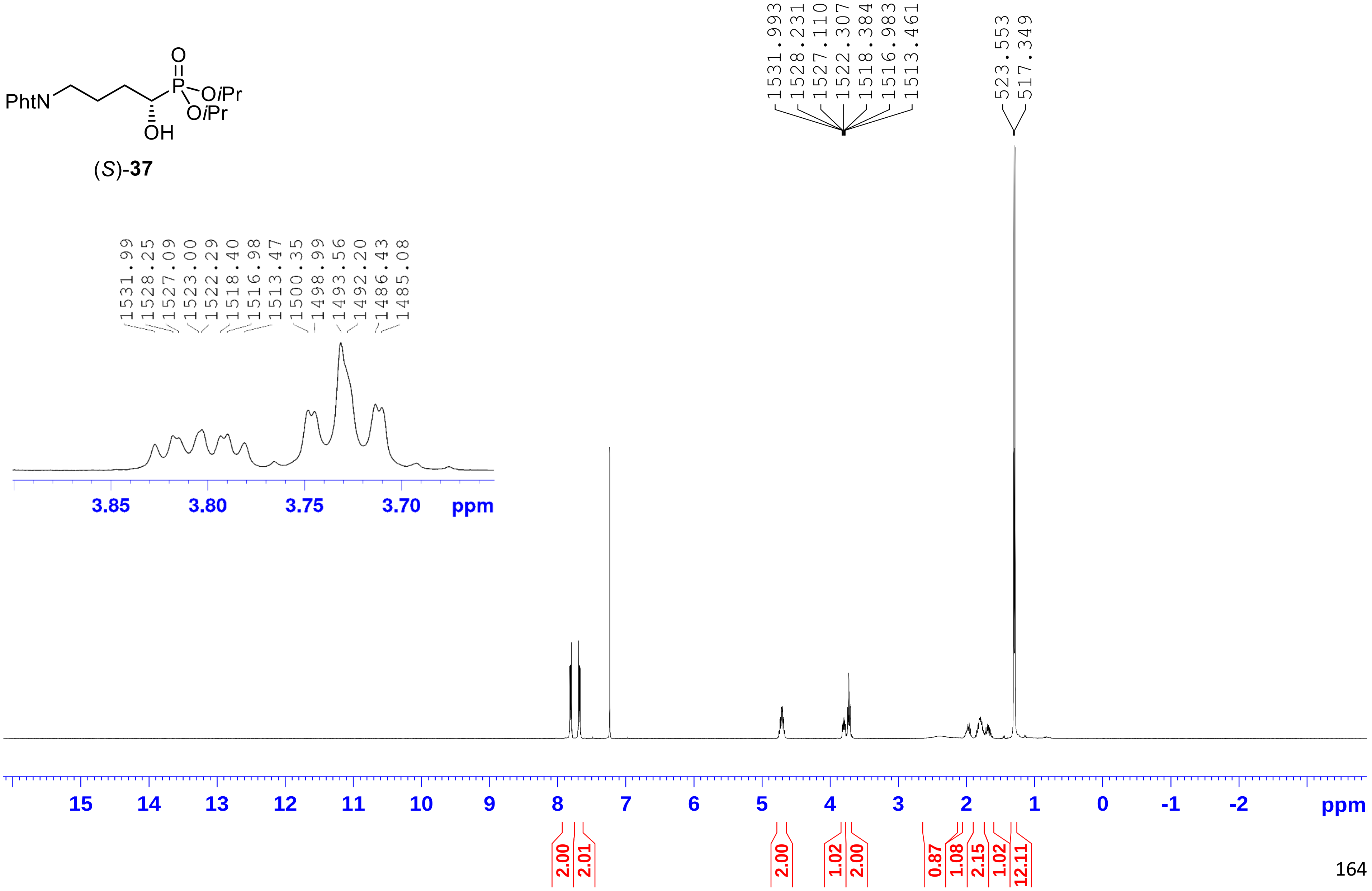

^{13}C NMR of (*S*)-diisopropyl (4-(1,3-dioxoisoindolin-2-yl)butanoyl)phosphonate ($CDCl_3$, 100.65 MHz) [(S)-37]:

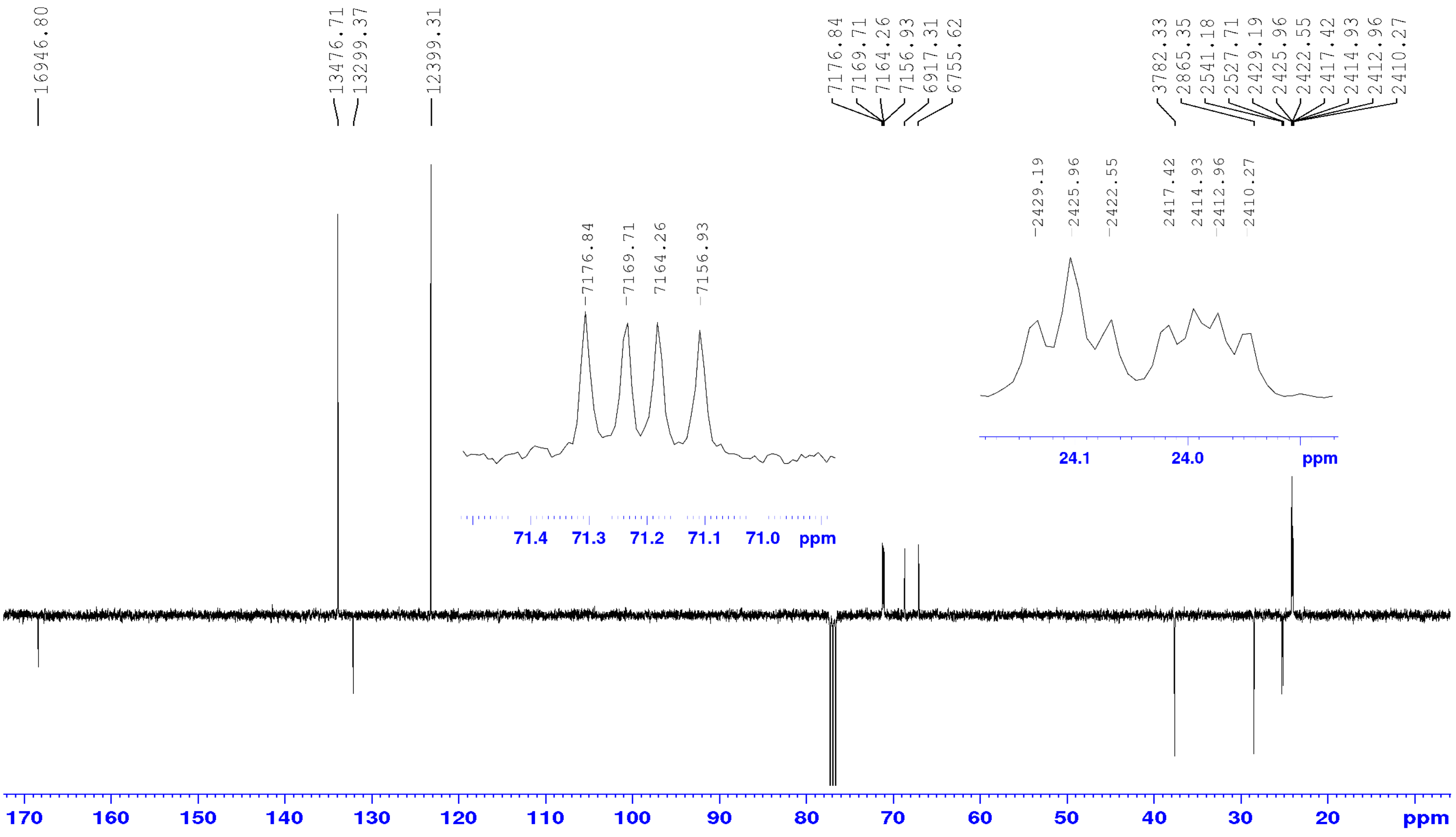

^{31}P NMR of (*S*)-diisopropyl (4-(1,3-dioxoisoindolin-2-yl)butanoyl)phosphonate ($CDCl_3$, 162.04 MHz) [(S)-37]:

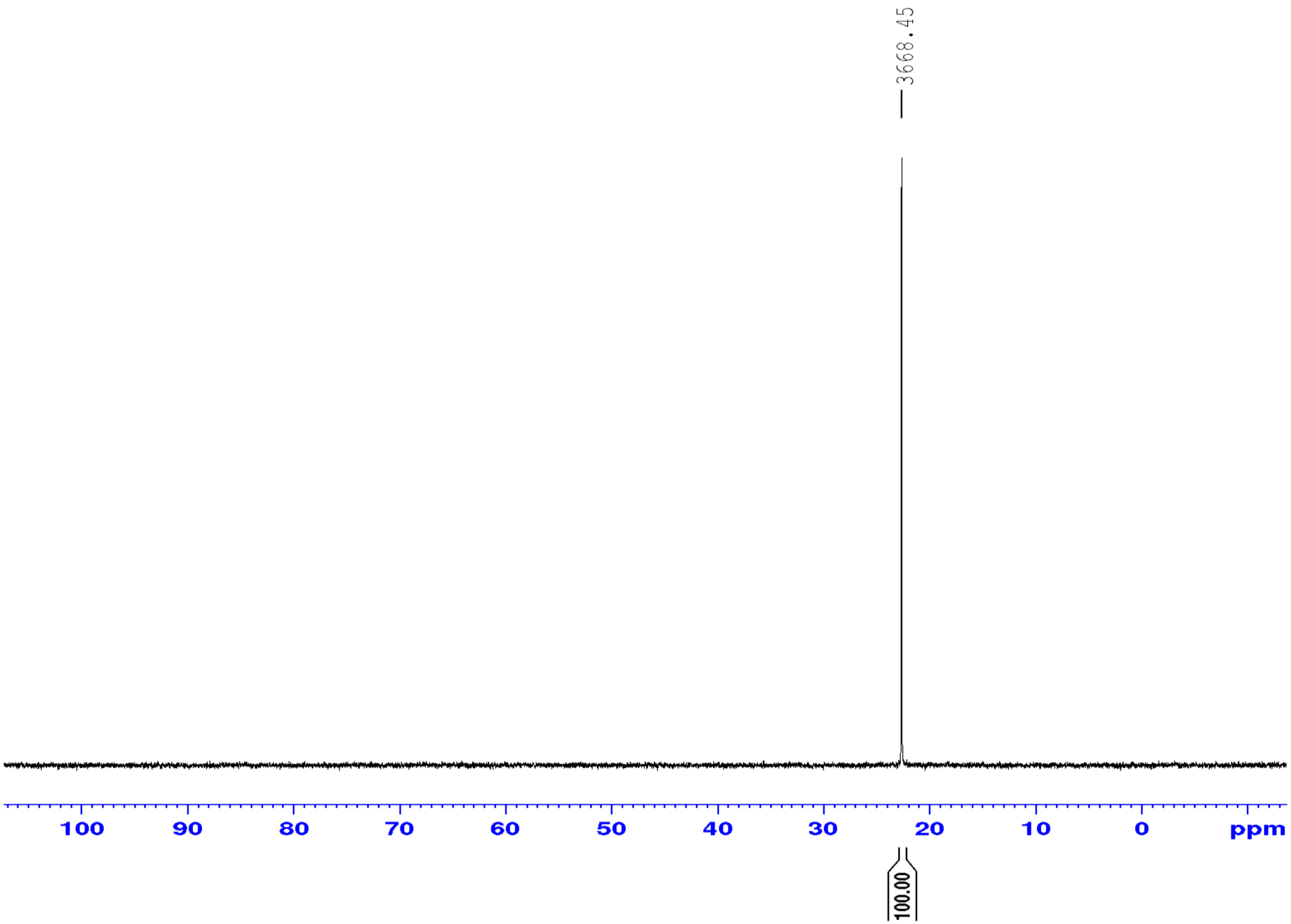

^{1}H NMR of (*S*)-(4-amino-1-hydroxybutyl)phosphonic acid (D_2O, 400.27 MHz) [(*S*)-87]:

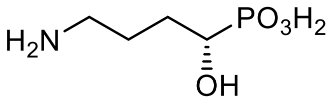

(*S*)-**87**

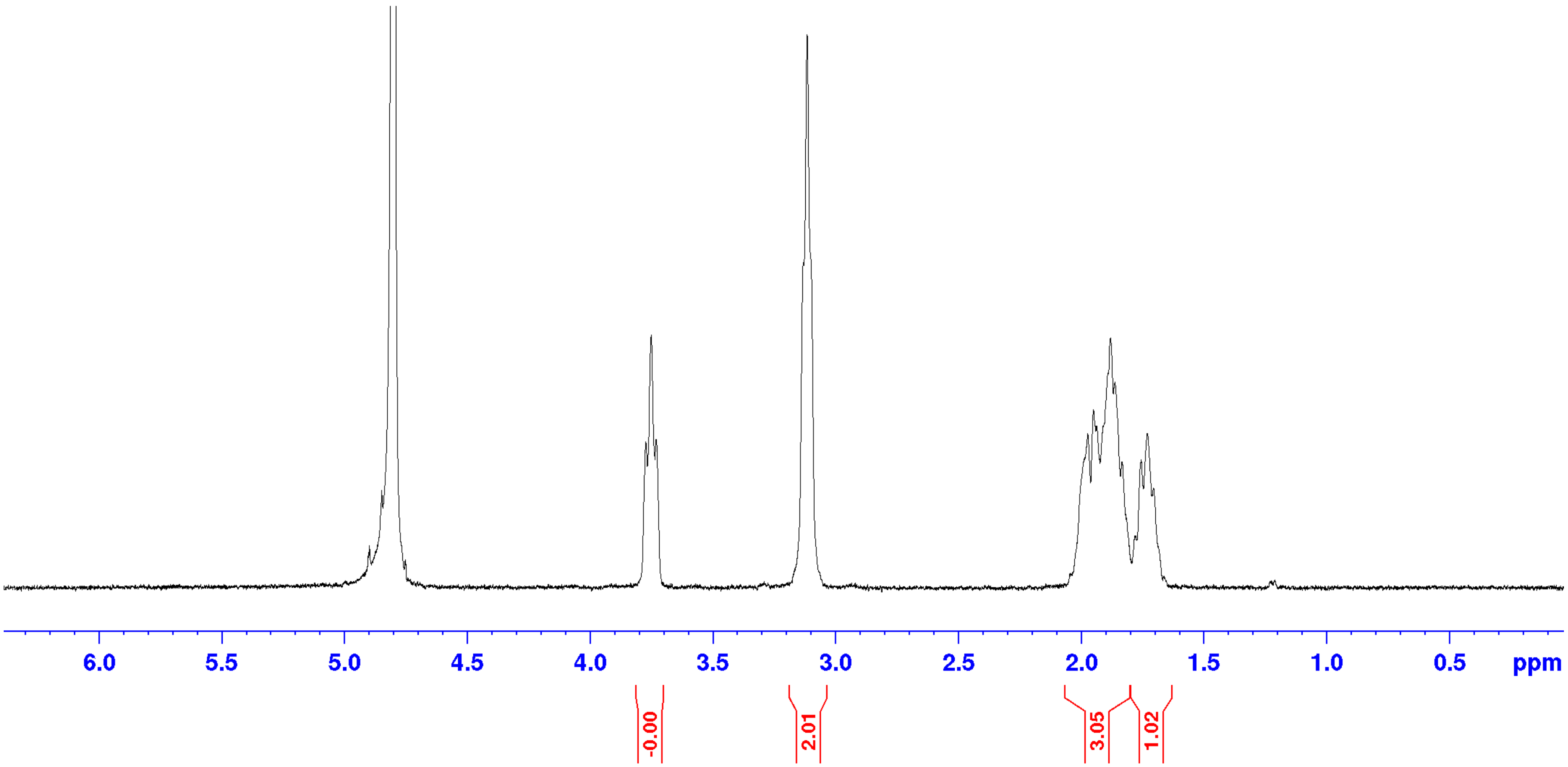

^{13}C NMR of (*S*)-(4-amino-1-hydroxybutyl)phosphonic acid (D_2O, 100.65 MHz) [(*S*)-87]:

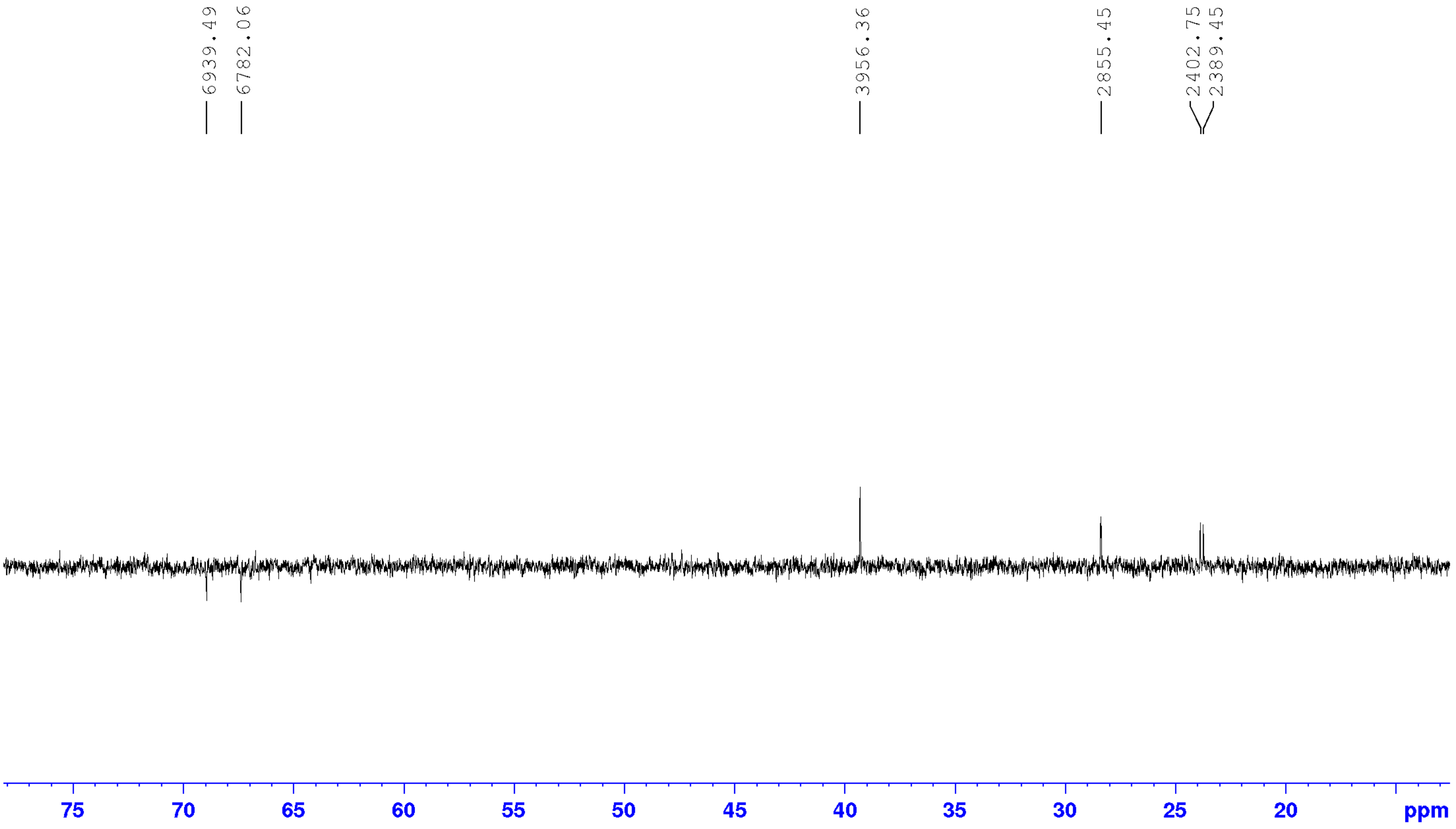

^{31}P NMR of (*S*)-(4-amino-1-hydroxybutyl)phosphonic acid (D_2O, 162.04 MHz) [(*S*)-87]:

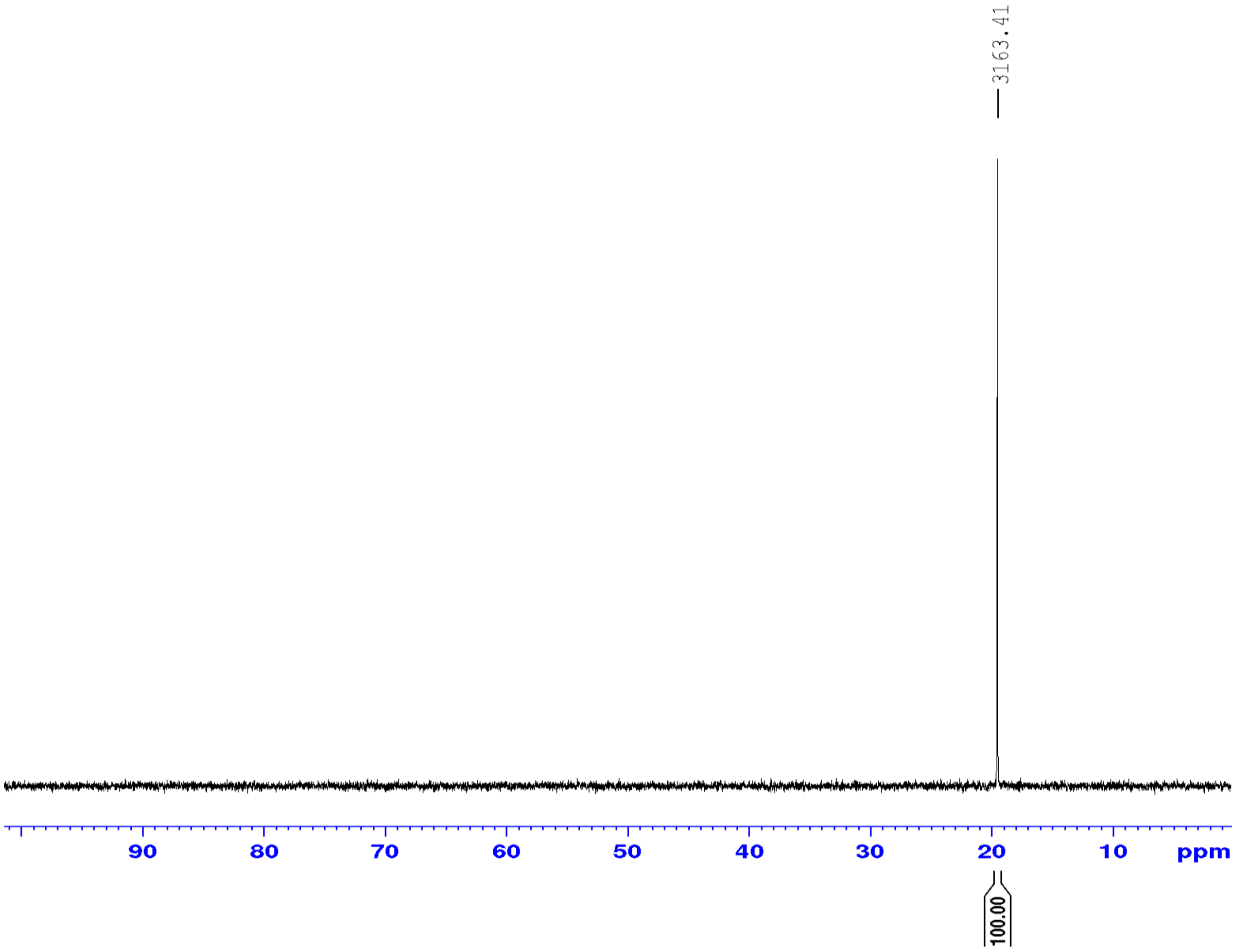

^{1}H NMR of diisopropyl (2-(benzylthio)acetyl)phosphonate (400.27 MHz, CDCl$_3$) (21):

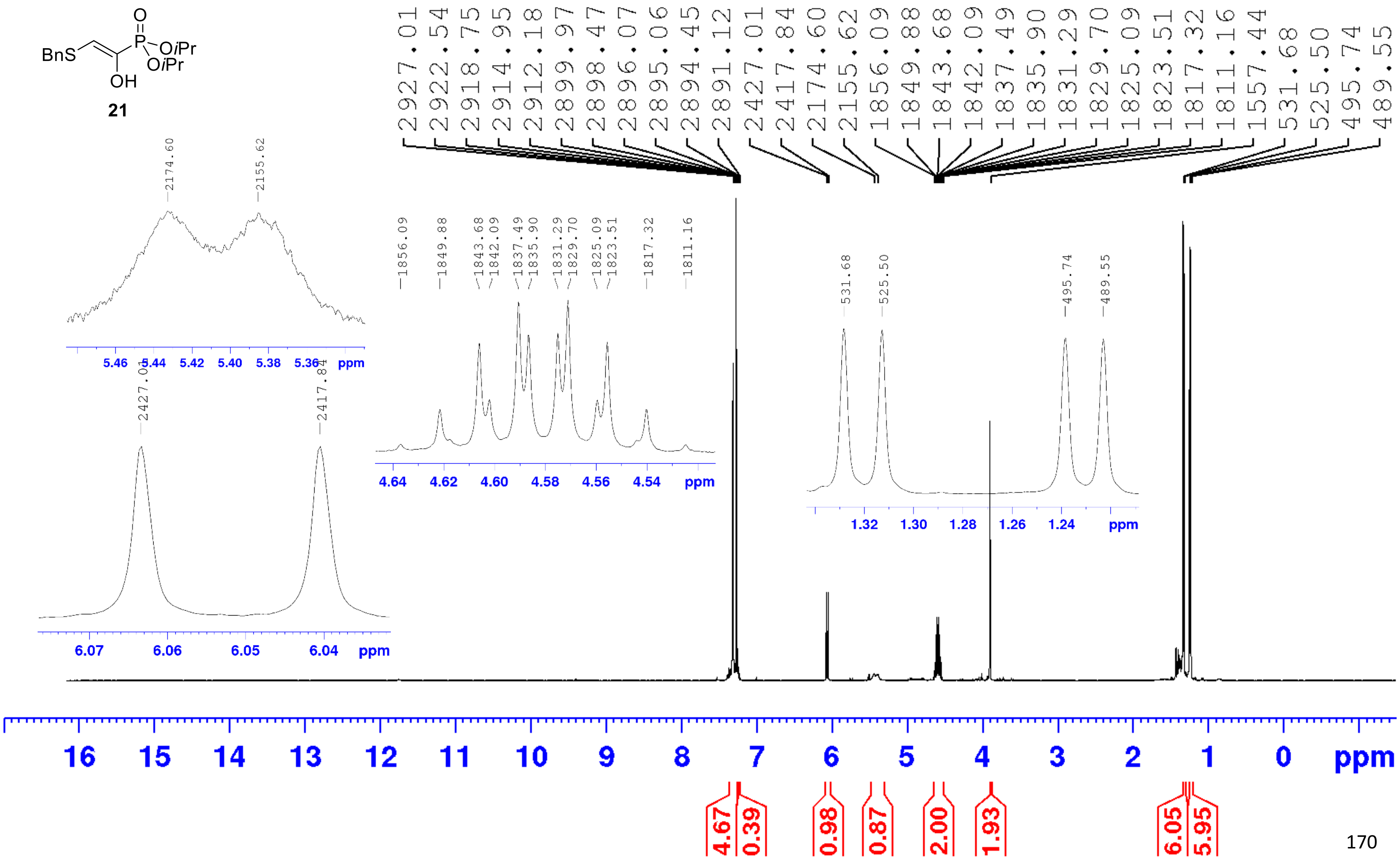

^{31}P NMR of diisopropyl (2-(benzylthio)acetyl)phosphonate (162.03 MHz, $CDCl_3$) (21):

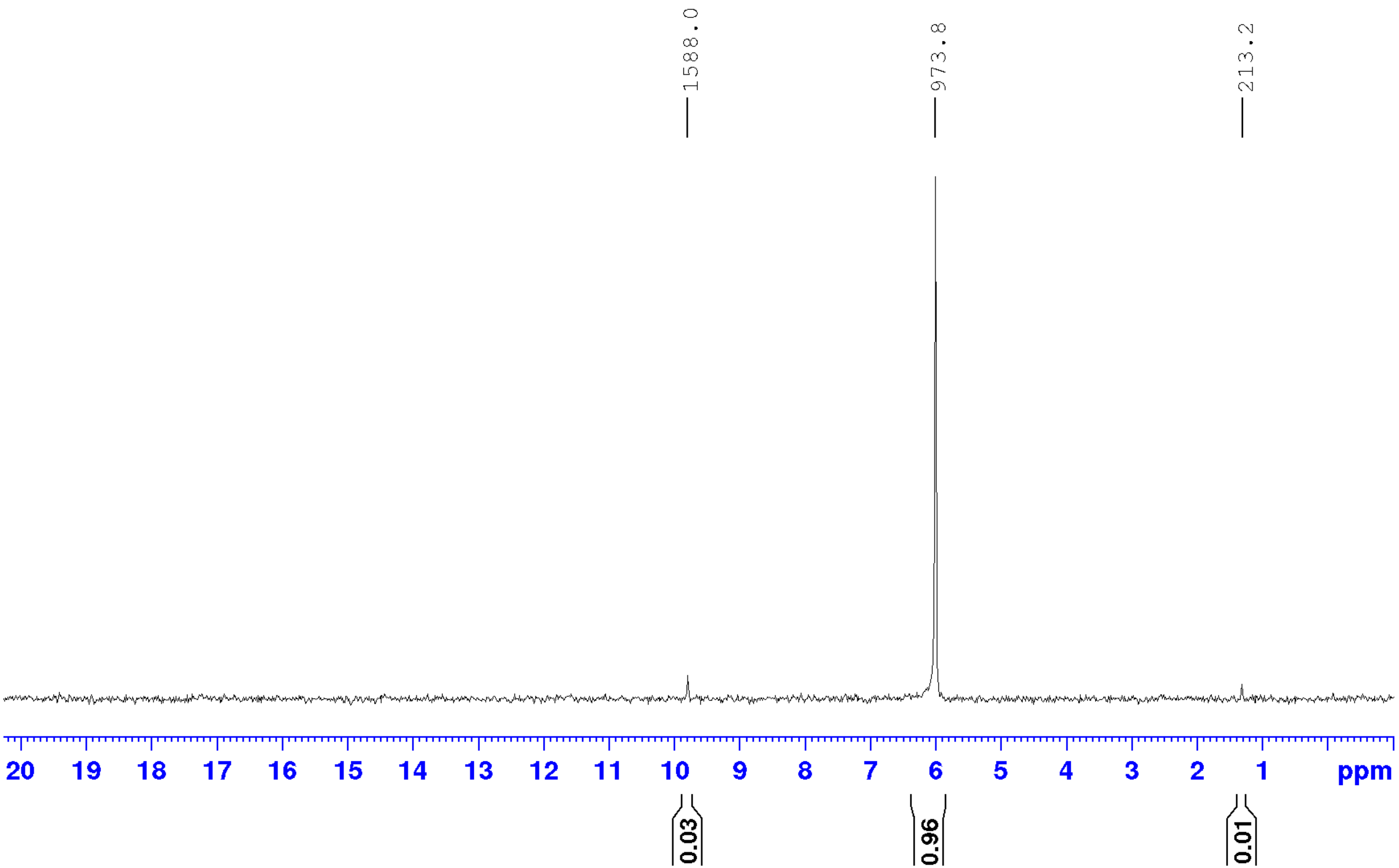

^{1}H NMR of diisopropyl (*S*)-(2-(benzylthio)-1-hydroxyethyl)phosphonate (400.27 MHz, $CDCl_3$) [(*S*)-38]:

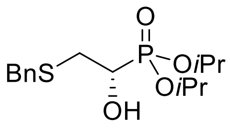

(*S*)-**38**

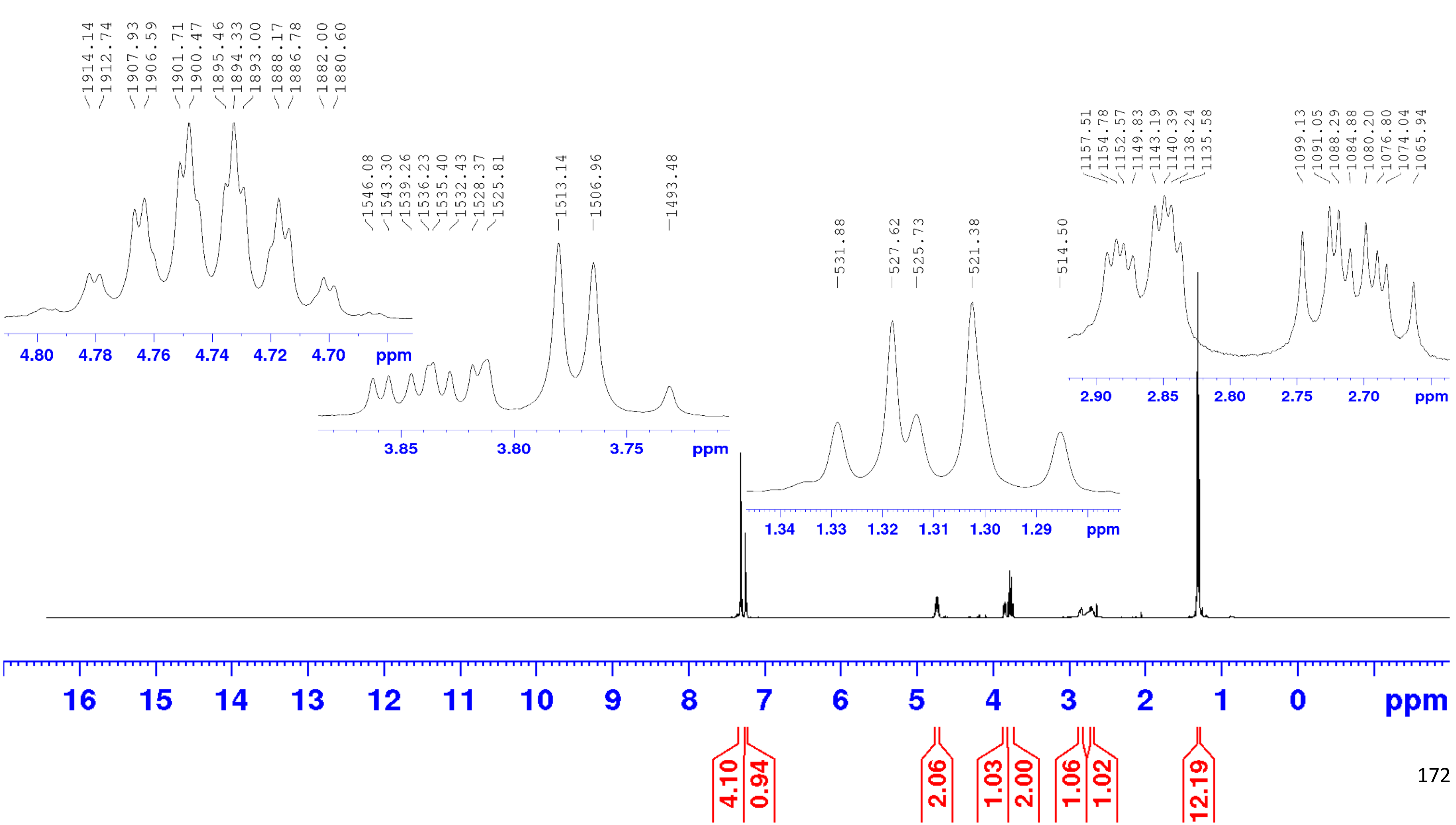

^{13}C NMR of diisopropyl (*S*)-(2-(benzylthio)-1-hydroxyethyl)phosphonate (150.93 MHz, $CDCl_3$) [(*S*)-38] :

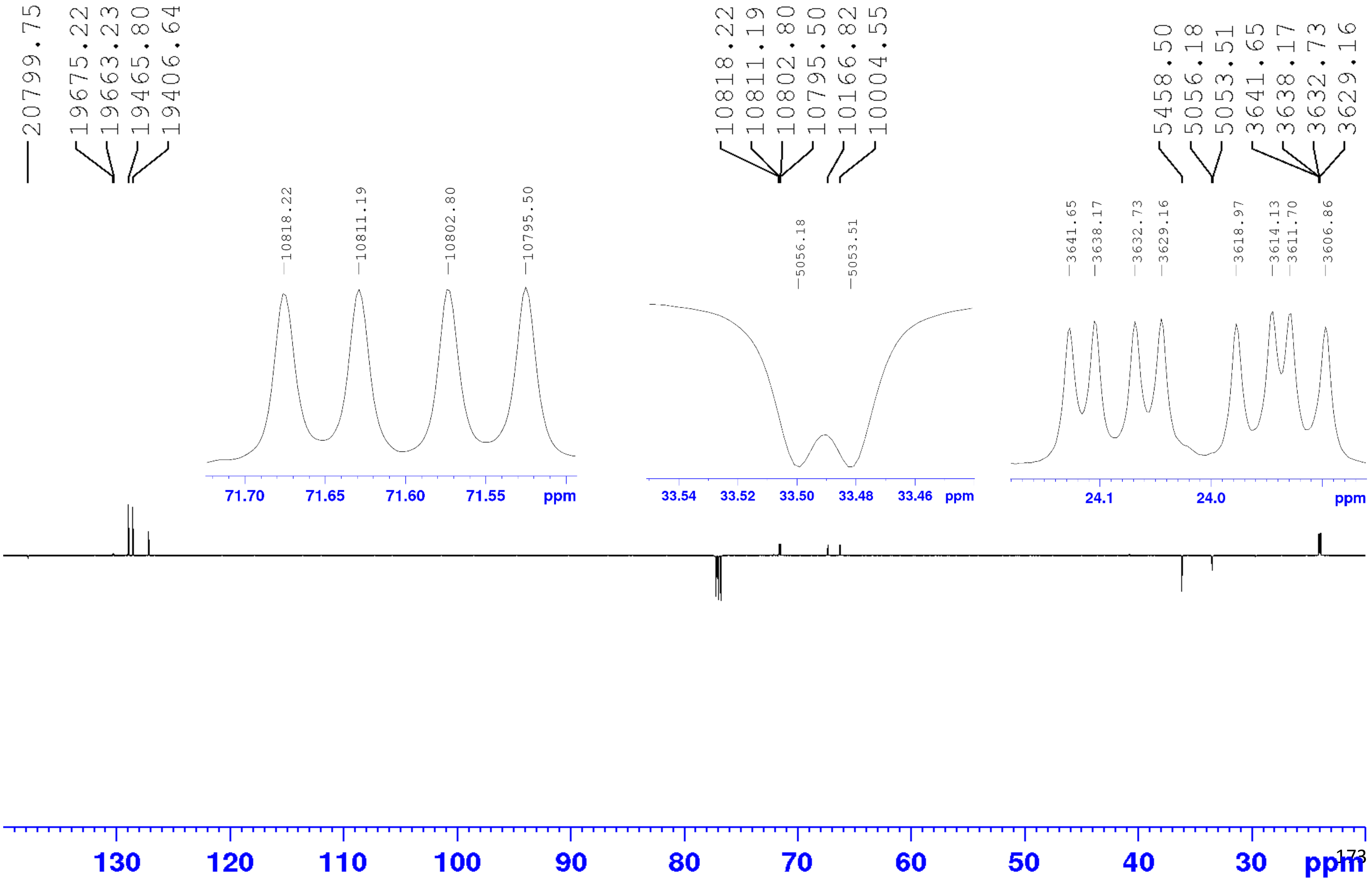

^{31}P NMR of diisopropyl (*S*)-(2-(benzylthio)-1-hydroxyethyl)phosphonate (162.03 MHz, $CDCl_3$) [(*S*)-38] :

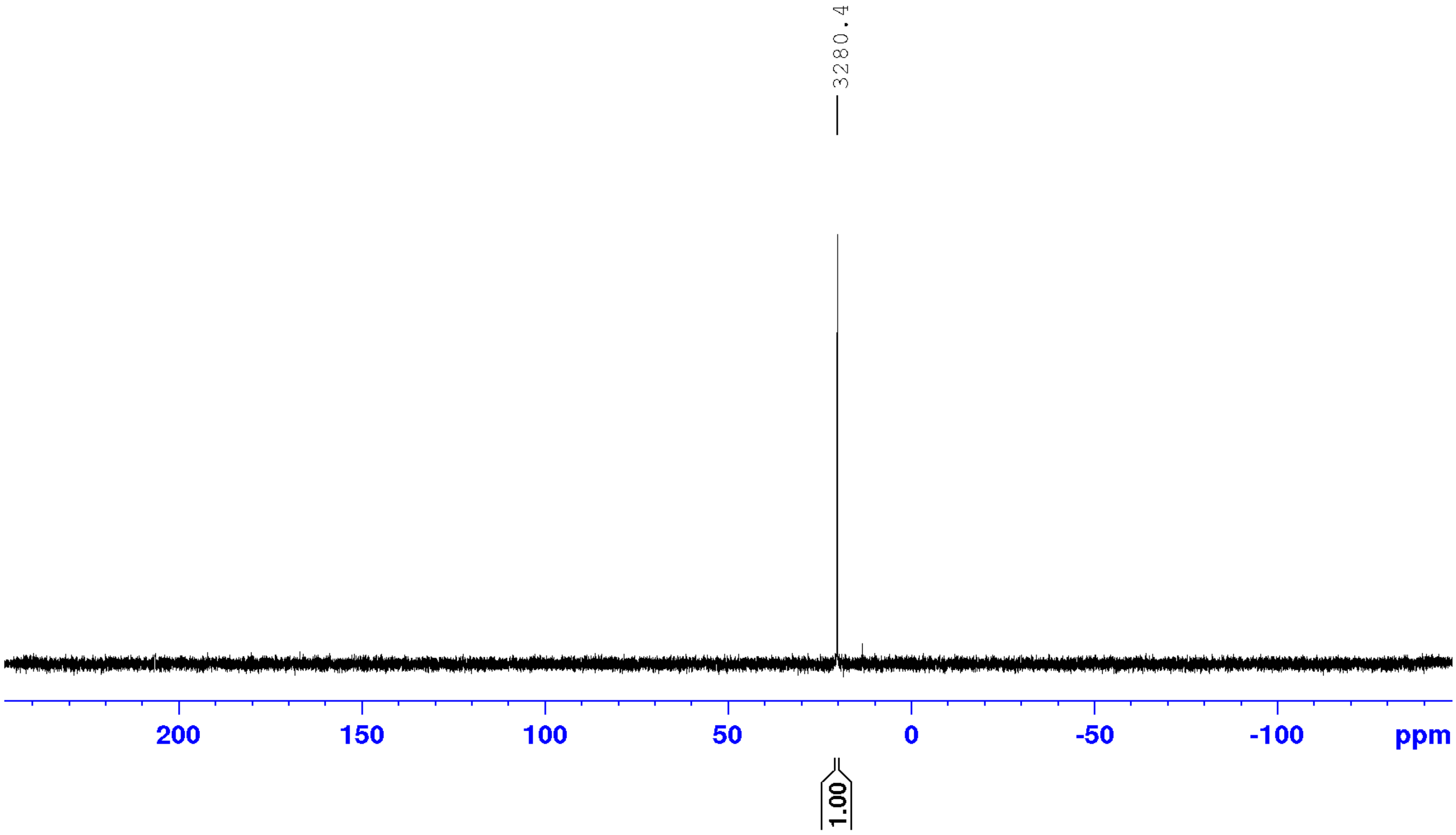

^{1}H NMR of diisopropyl (*R*)-(1-azido-2-(benzylthio)ethyl)phosphonate (400.27 MHz, $CDCl_3$) [(*R*)-47a] :

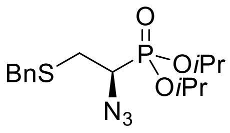

(*R*)-**47a**

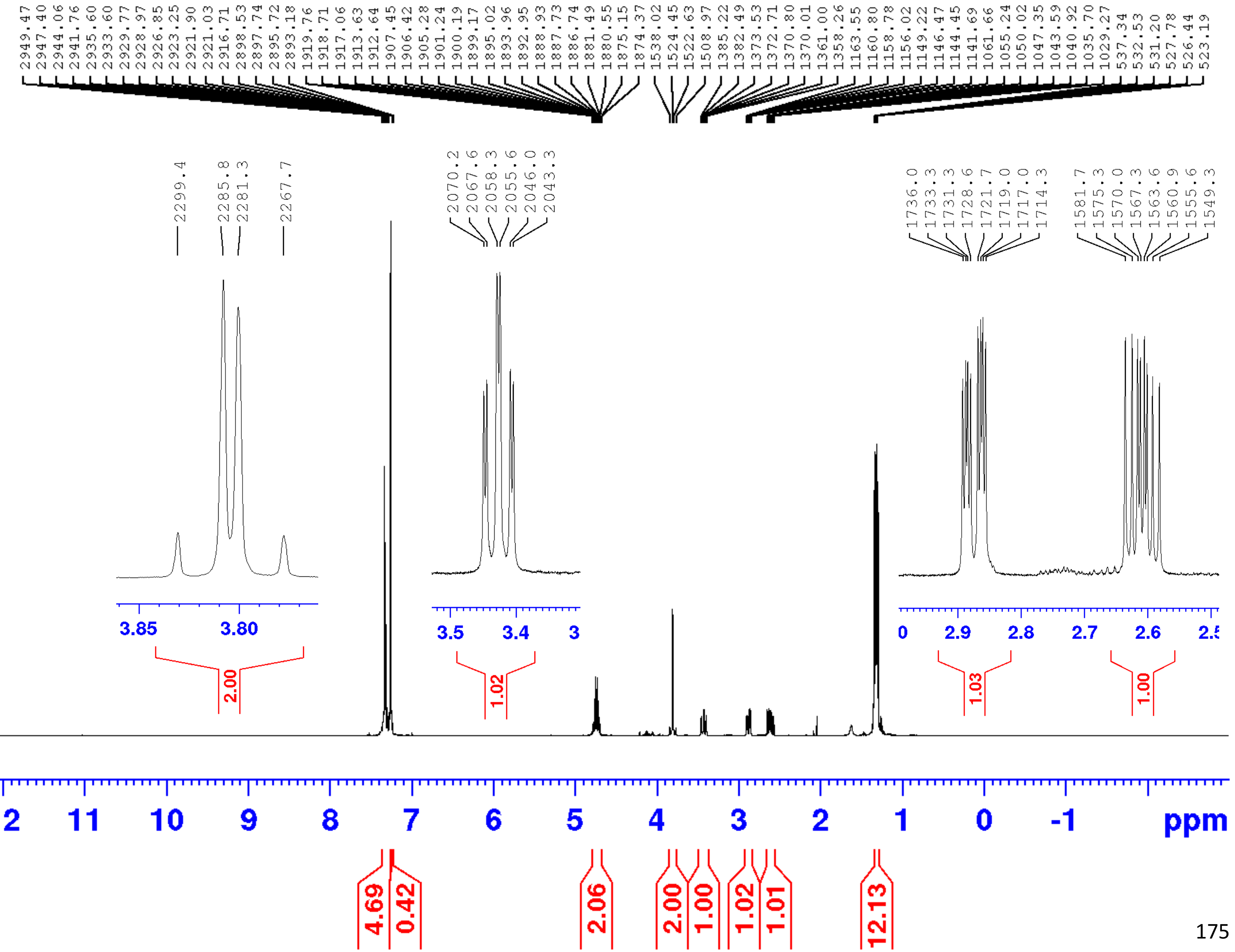

^{13}C NMR of diisopropyl (*R*)-(1-azido-2-(benzylthio)ethyl)phosphonate (150.93 MHz, $CDCl_3$) [(*R*)-47a]:

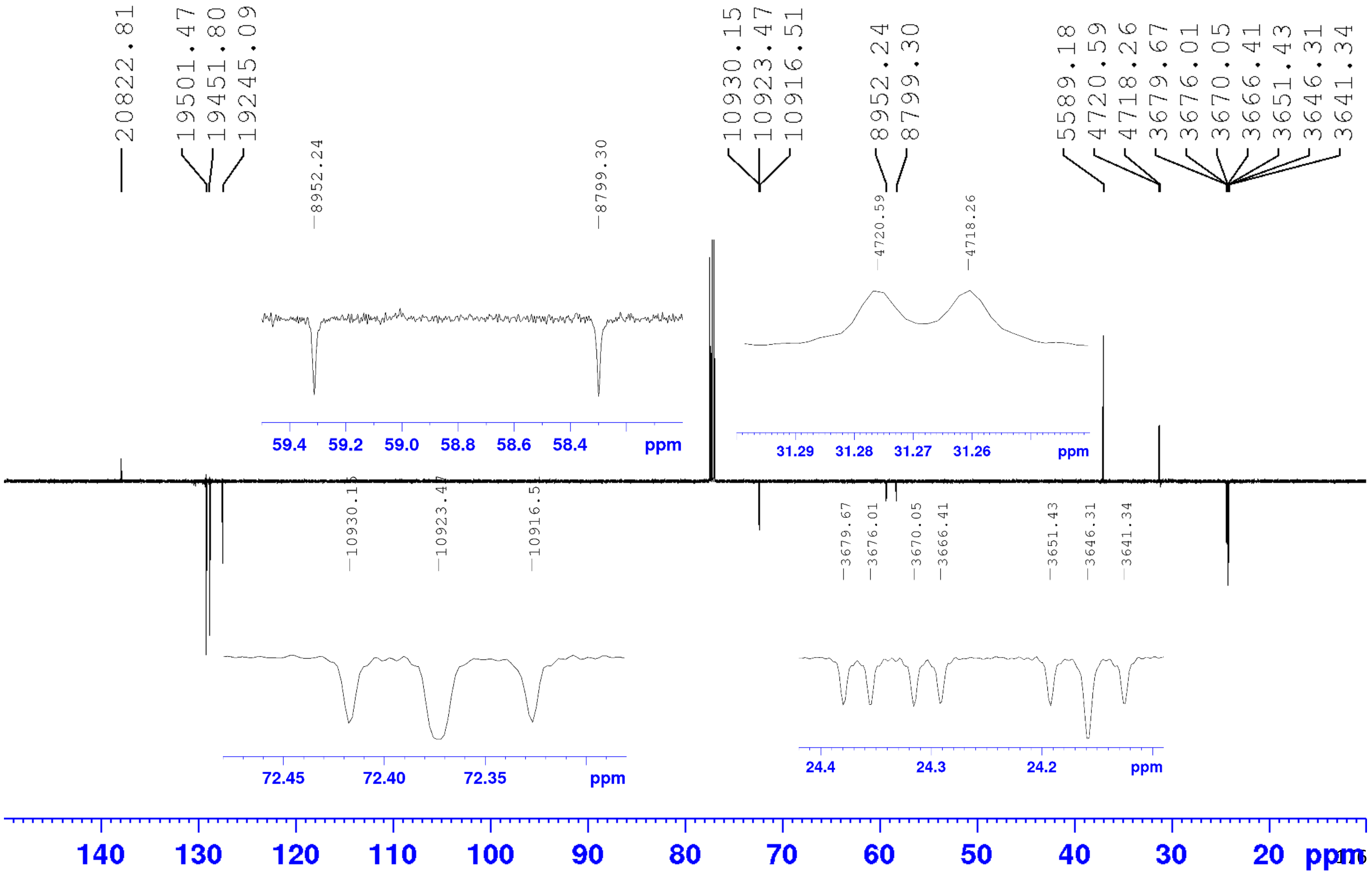

^{31}P NMR of diisopropyl (*R*)-(1-azido-2-(benzylthio)ethyl)phosphonate (162.03 MHz, CDCl$_3$) [(*R*)-47a]:

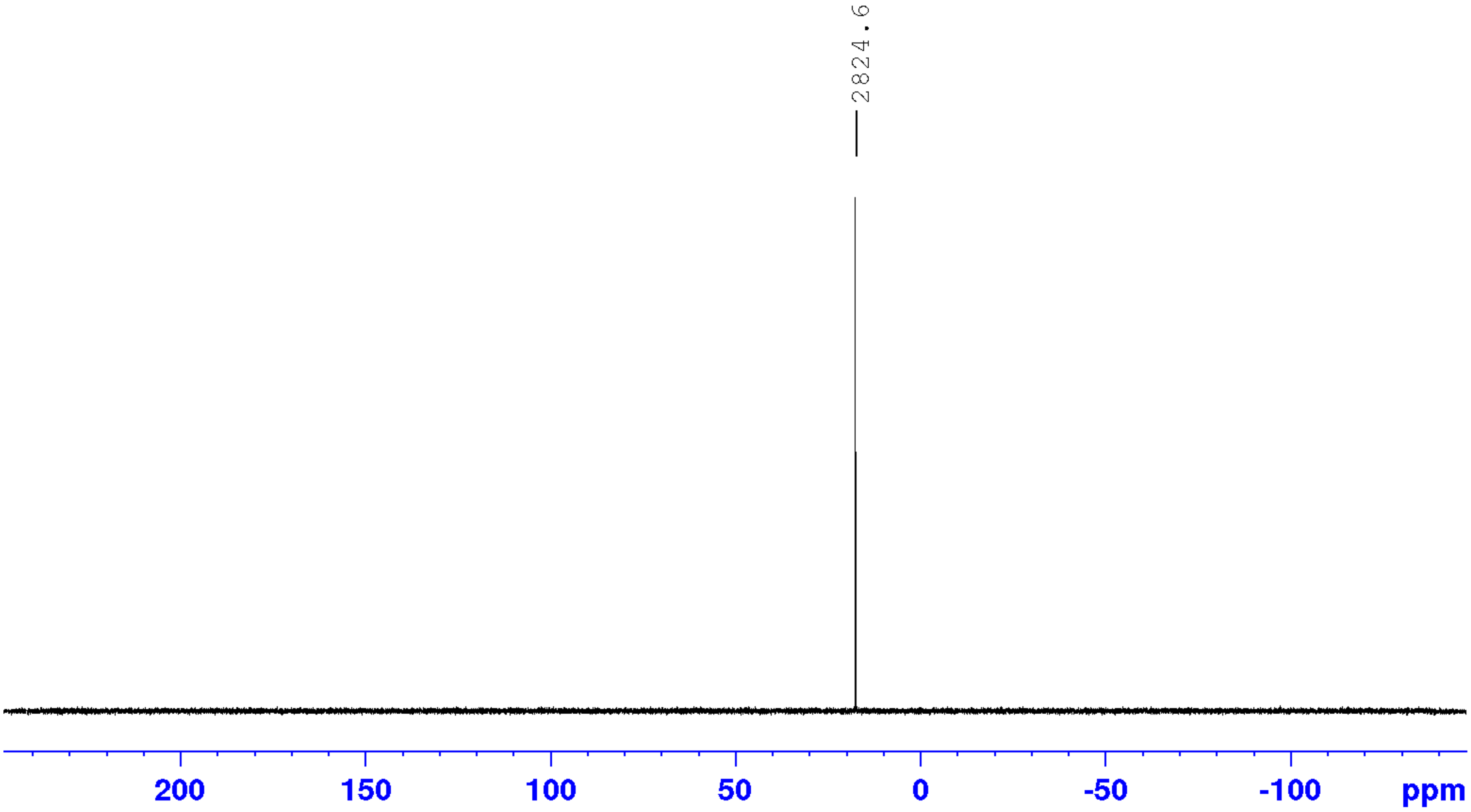

^{1}H NMR of diisopropyl (*S*)-(2-azido-1-(benzylthio)ethyl)phosphonate (700.40 MHz, $CDCl_3$) [(*S*)-47b]:

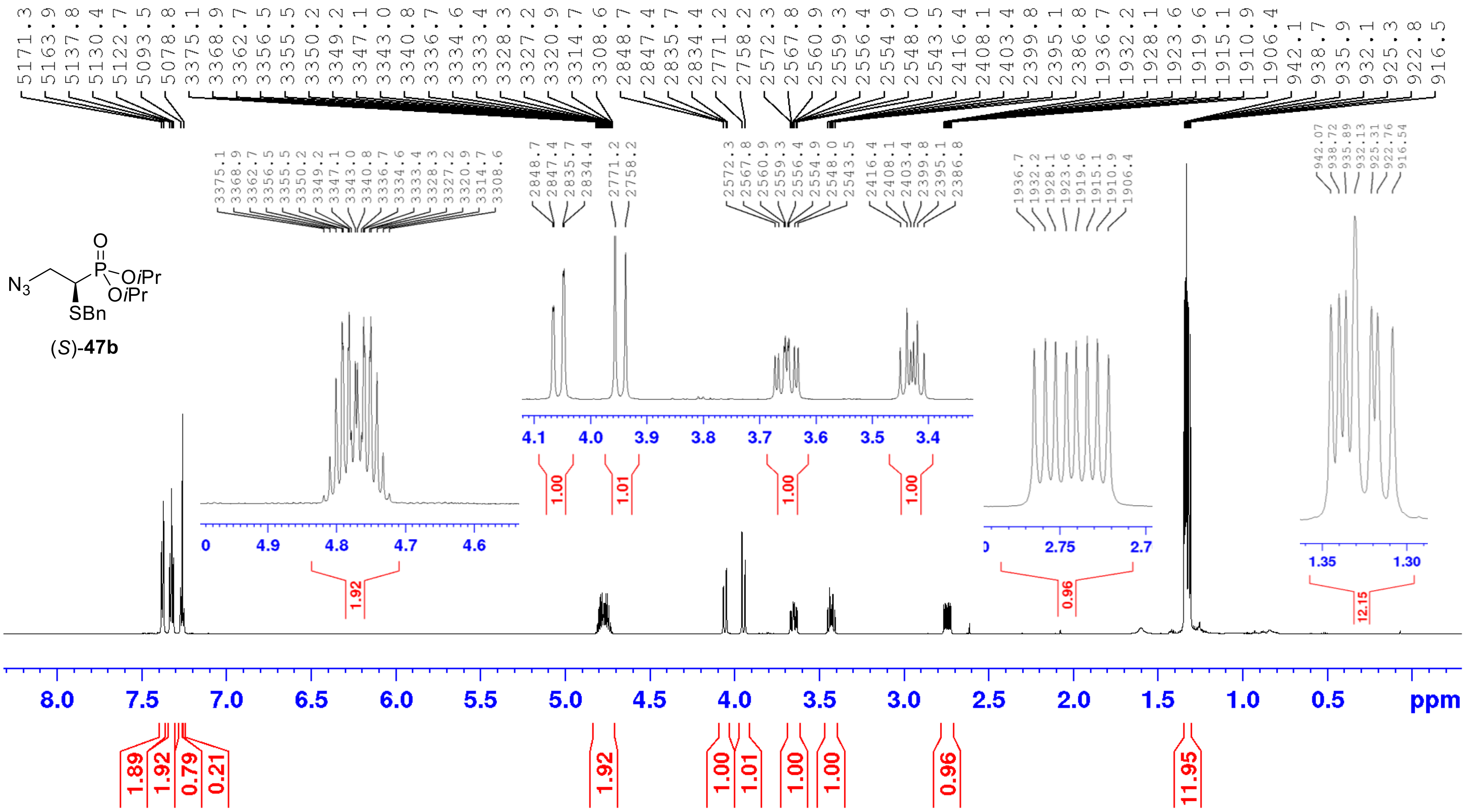

^{13}C NMR of diisopropyl (*S*)-(2-azido-1-(benzylthio)ethyl)phosphonate (176.12 MHz, $CDCl_3$) [(*S*)-47b]:

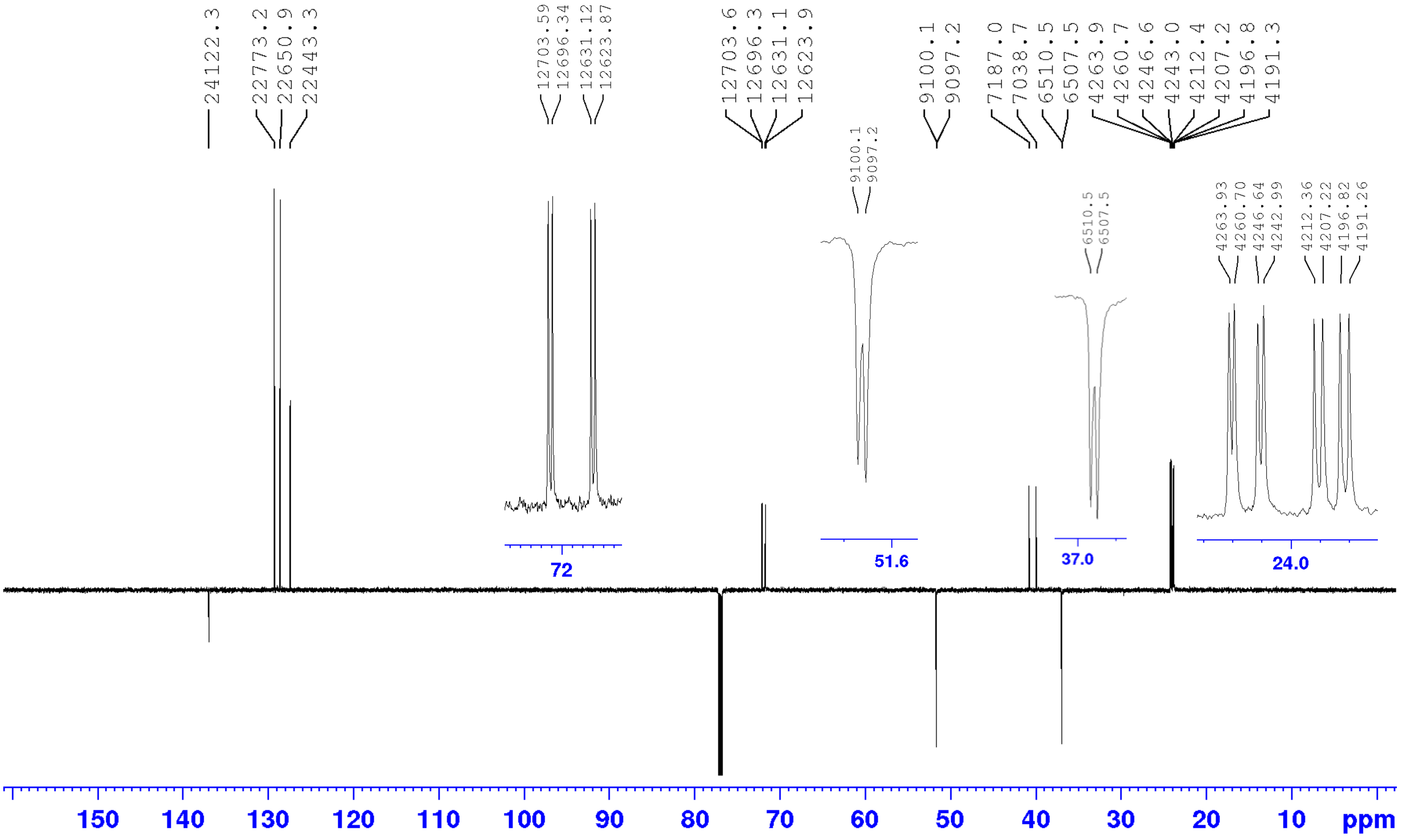

^{31}P NMR of diisopropyl (*S*)-(2-azido-1-(benzylthio)ethyl)phosphonate (161.98 MHz, $CDCl_3$) [(*S*)-47b]:

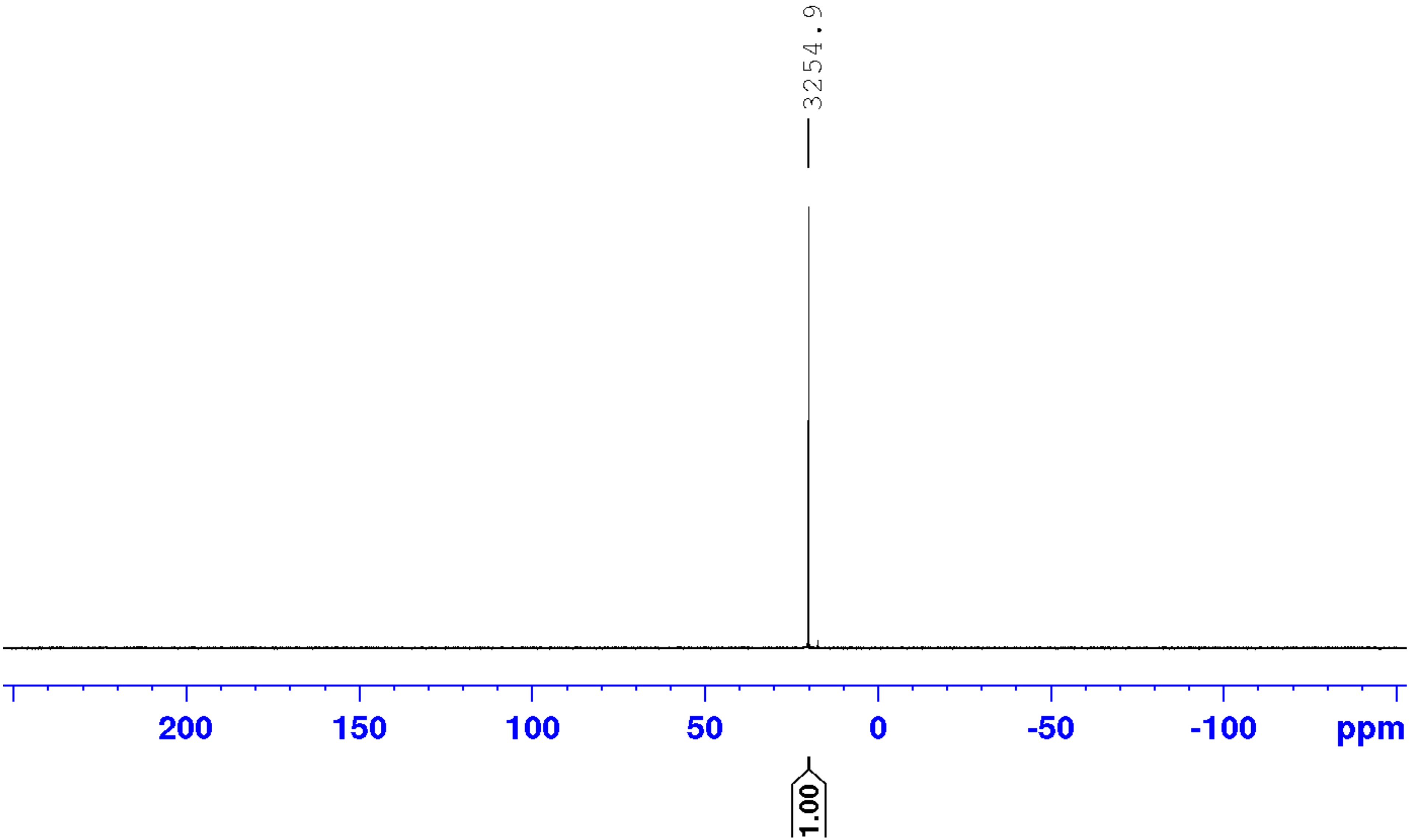

^{1}H NMR of *tert*-butyl (*R*)-(2-(benzylthio)-1-(diisopropoxyphosphoryl)ethyl)carbamate (400.27 MHz, $CDCl_3$) [(*R*)-88]:

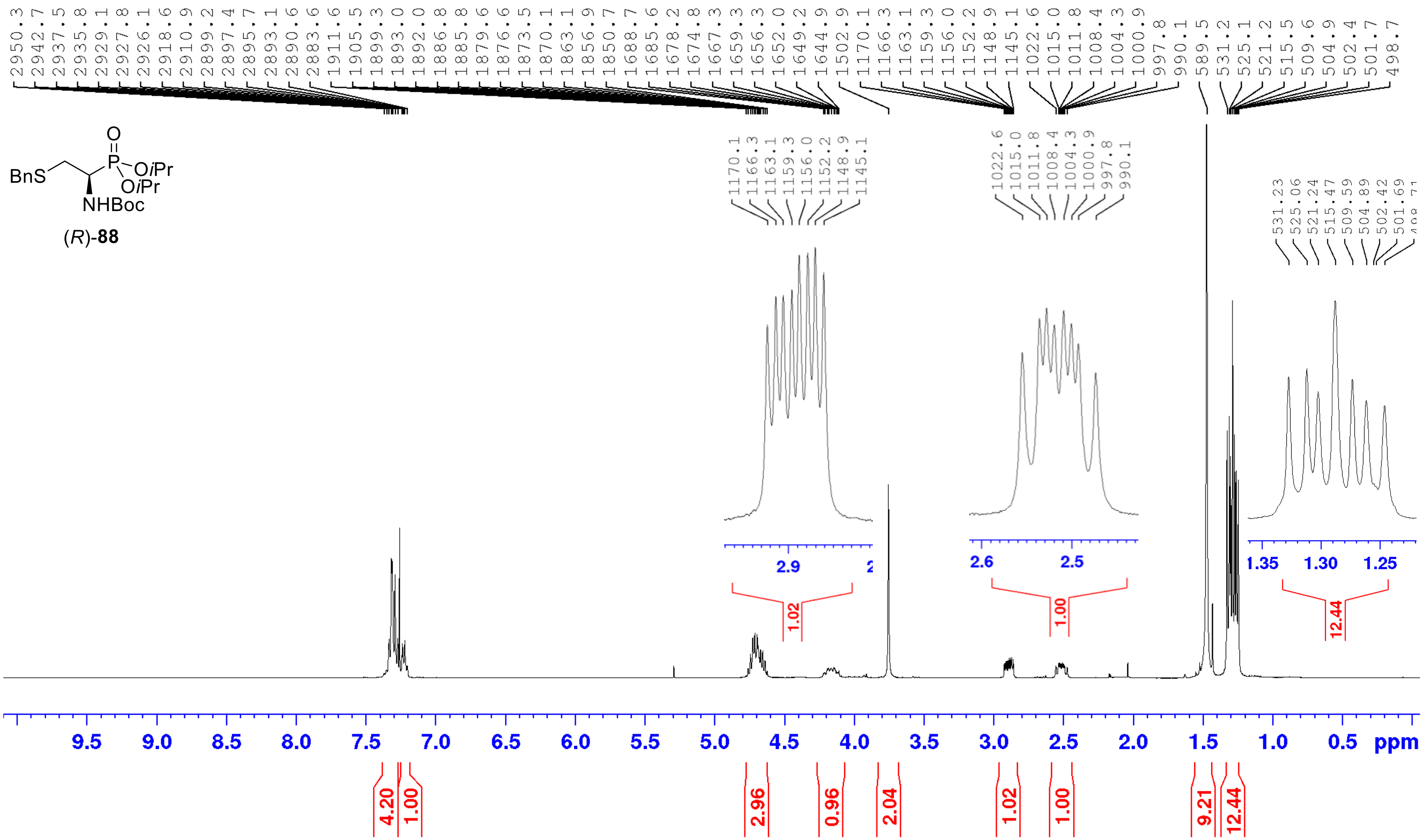

^{13}C NMR of *tert*-butyl (*R*)-(2-(benzylthio)-1-(diisopropoxyphosphoryl)ethyl)carbamate (150.93 MHz, $CDCl_3$) [(*R*)-88]:

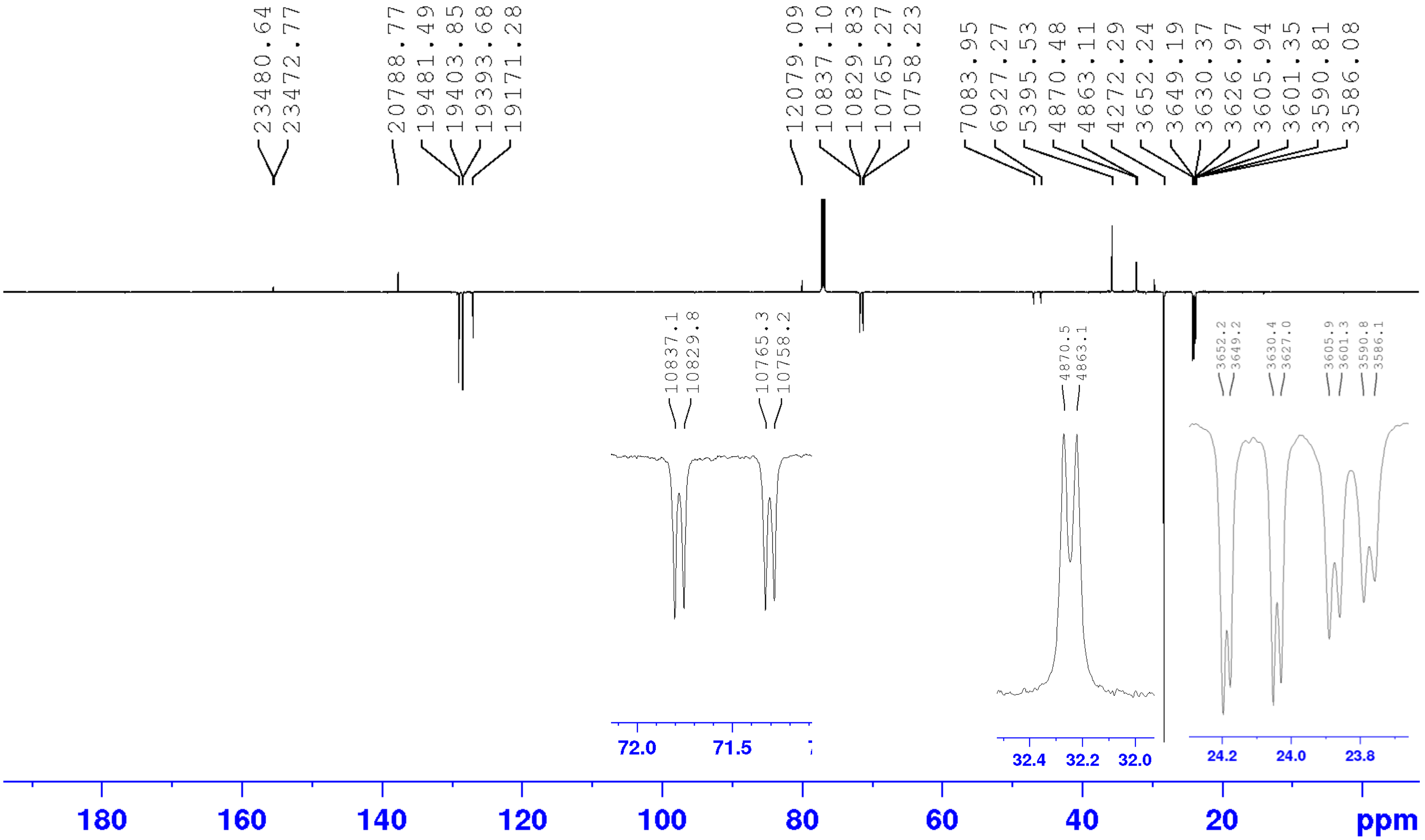

^{31}P NMR of *tert*-butyl (*R*)-(2-(benzylthio)-1-(diisopropoxyphosphoryl)ethyl)carbamate (162.03 MHz, $CDCl_3$) [(*R*)-88]:

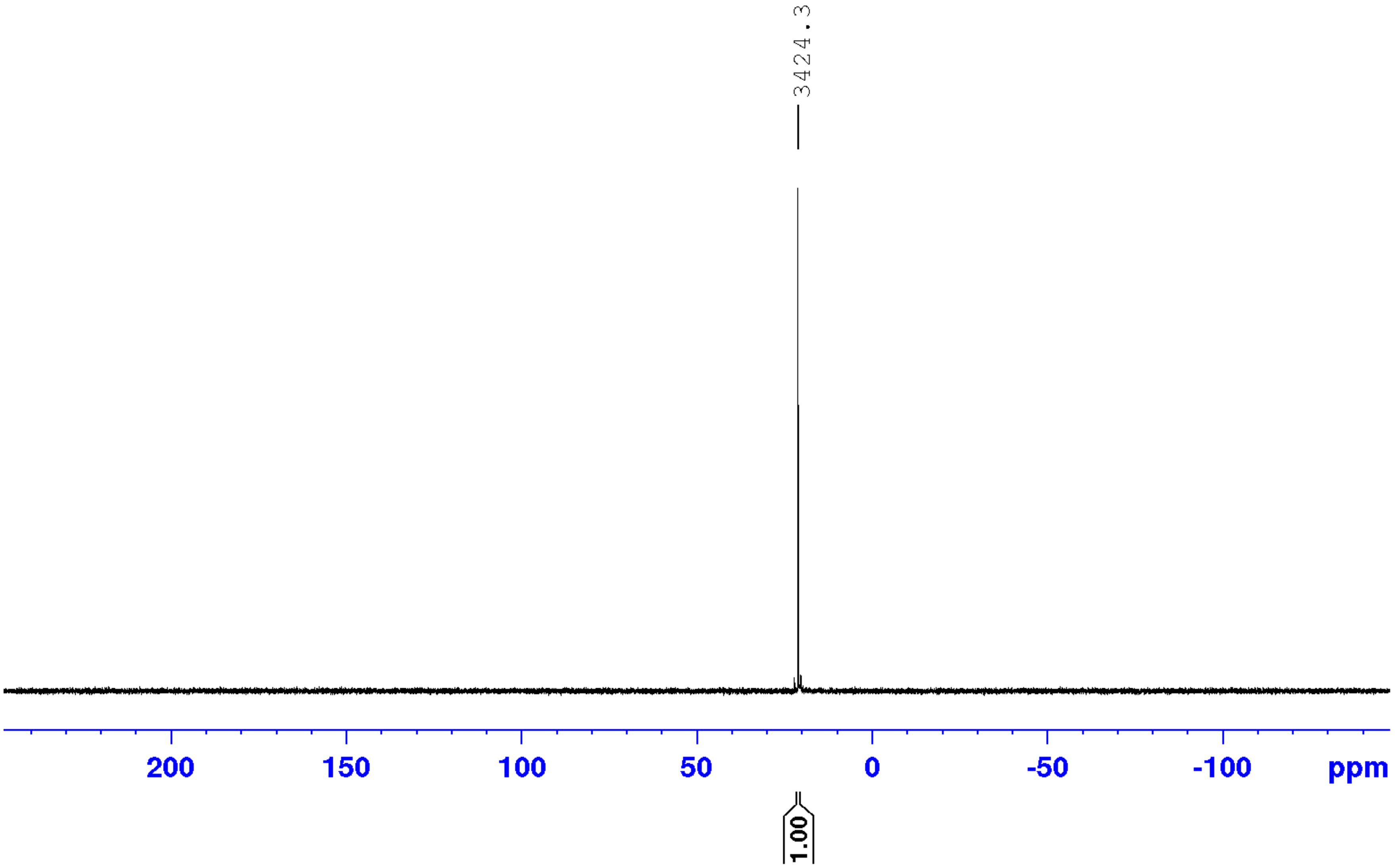

^{1}H NMR of (*R*)-(1-amino-2-mercaptoethyl)phosphonic acid, (*R*)-phosphacysteine (600.25 MHz, D_2O) [(*R*)-70]:

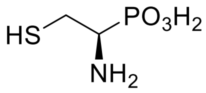

(*R*)-**70**

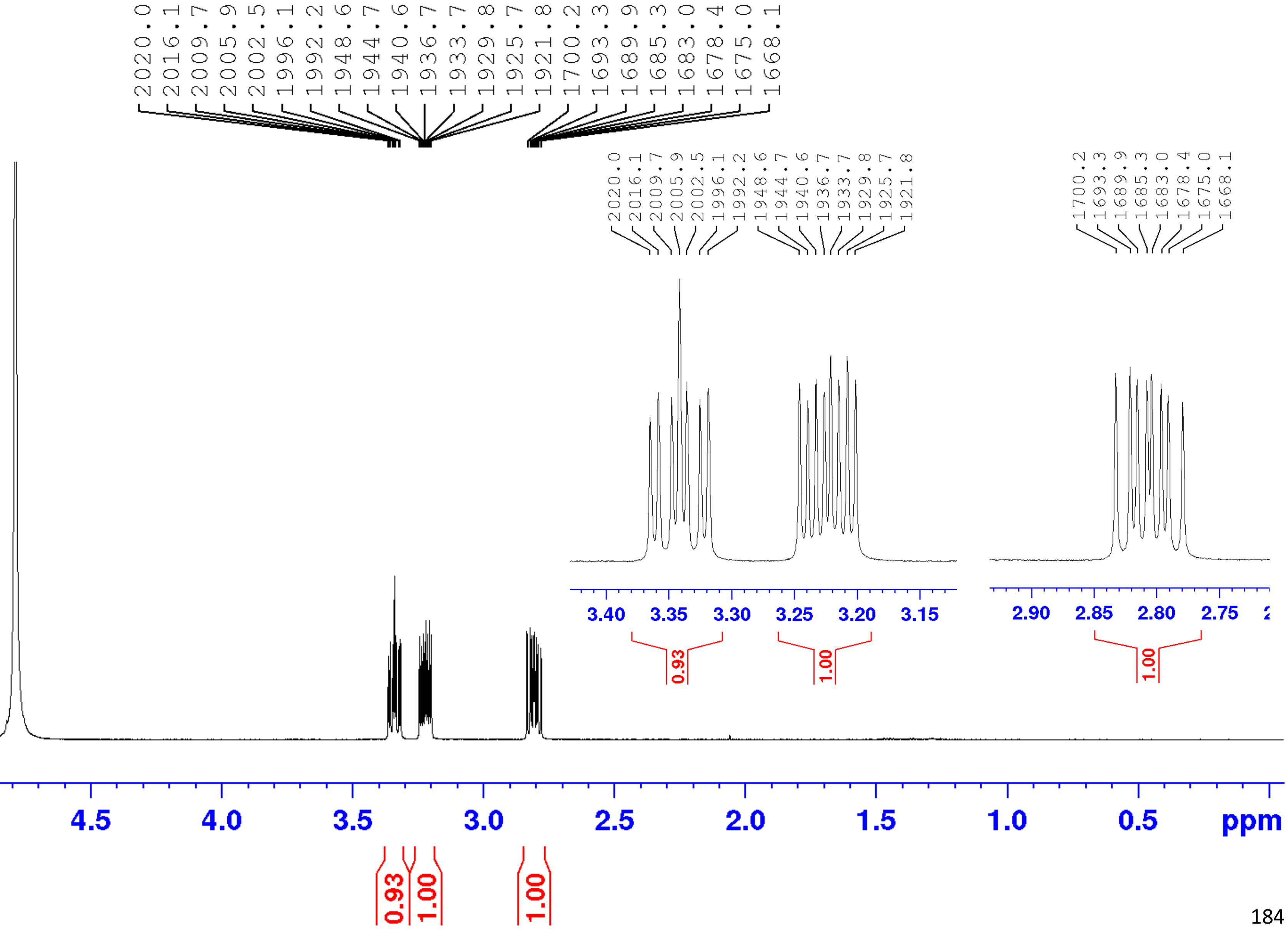

^{13}C NMR of (*R*)-(1-amino-2-mercaptoethyl)phosphonic acid, (*R*)-phosphacysteine (150.93 MHz, D_2O) [(*R*)-70]:

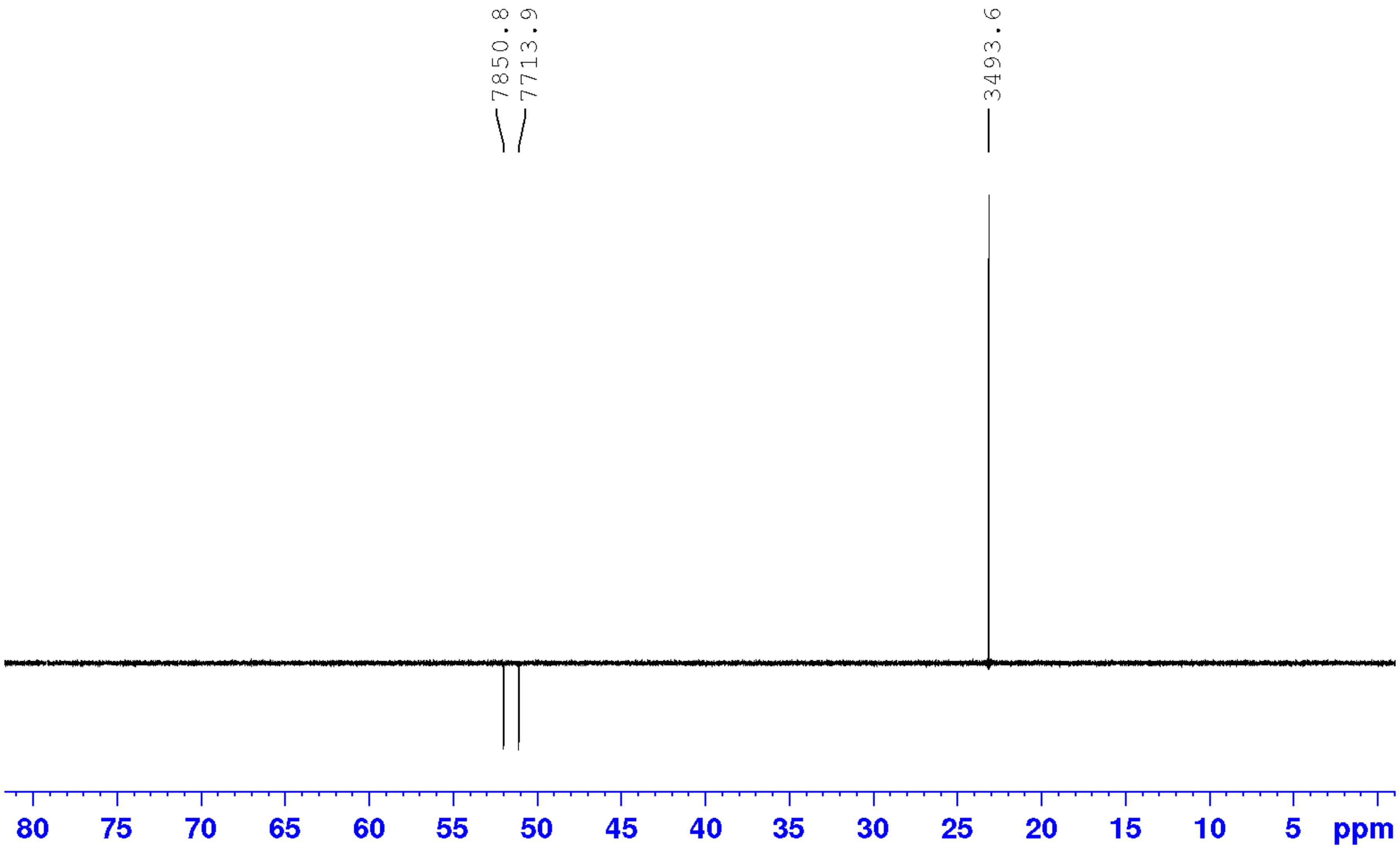

^{31}P NMR of (*R*)-(1-amino-2-mercaptoethyl)phosphonic acid, (*R*)-phosphacysteine (162.03 MHz, D_2O) [(*R*)-70]:

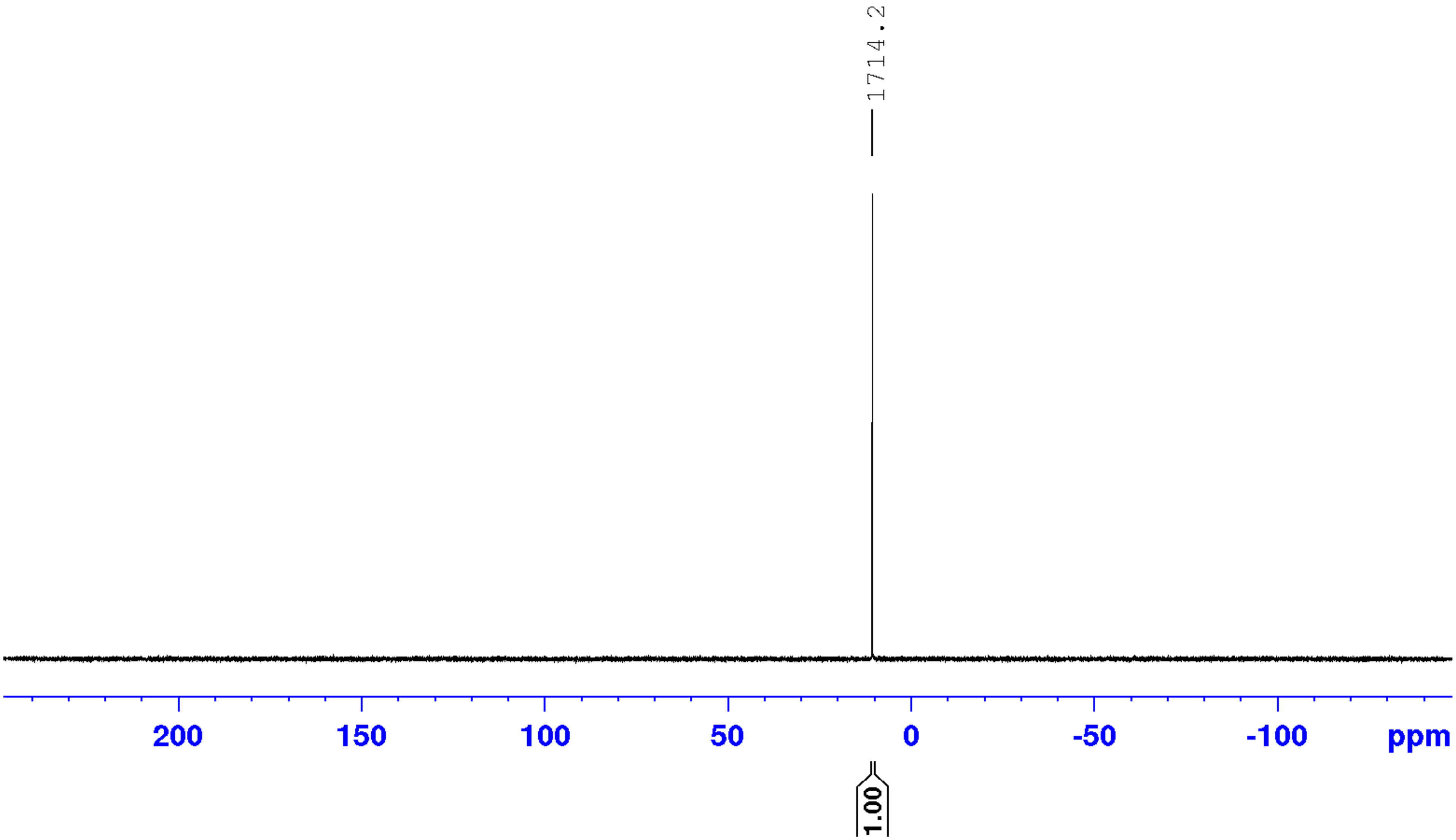

^{31}P NMR of Diisopropyl 1-oxo-3-(methylthio)propylphosphonate (162.03 MHz, $CDCl_3$) (21):

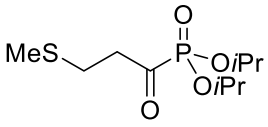

21

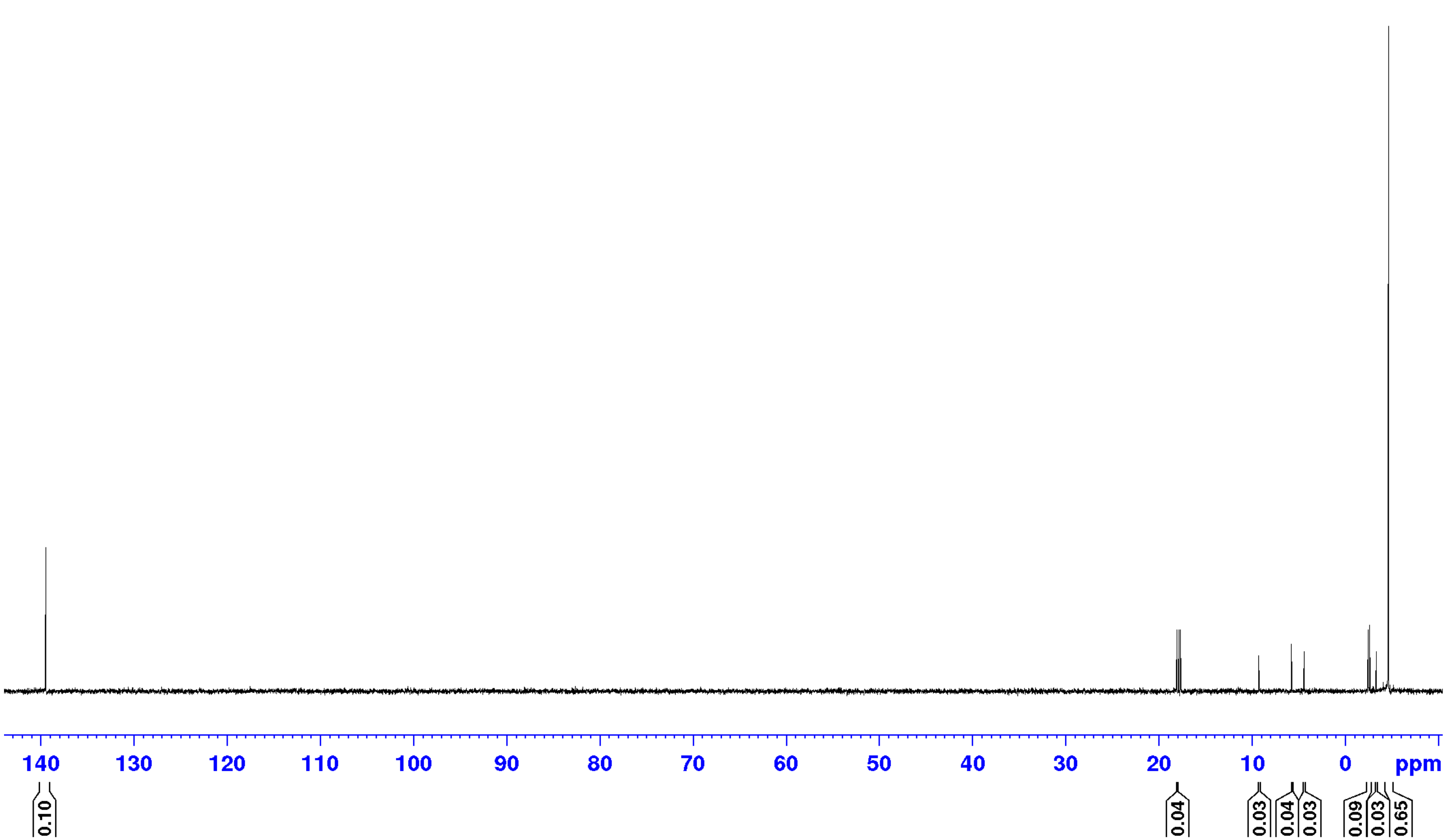

^{1}H NMR of (*S*)-diisopropyl (1-hydroxy-3-methylthiopropyl)phosphonate (400.27 MHz, $CDCl_3$) [(*S*)-39]:

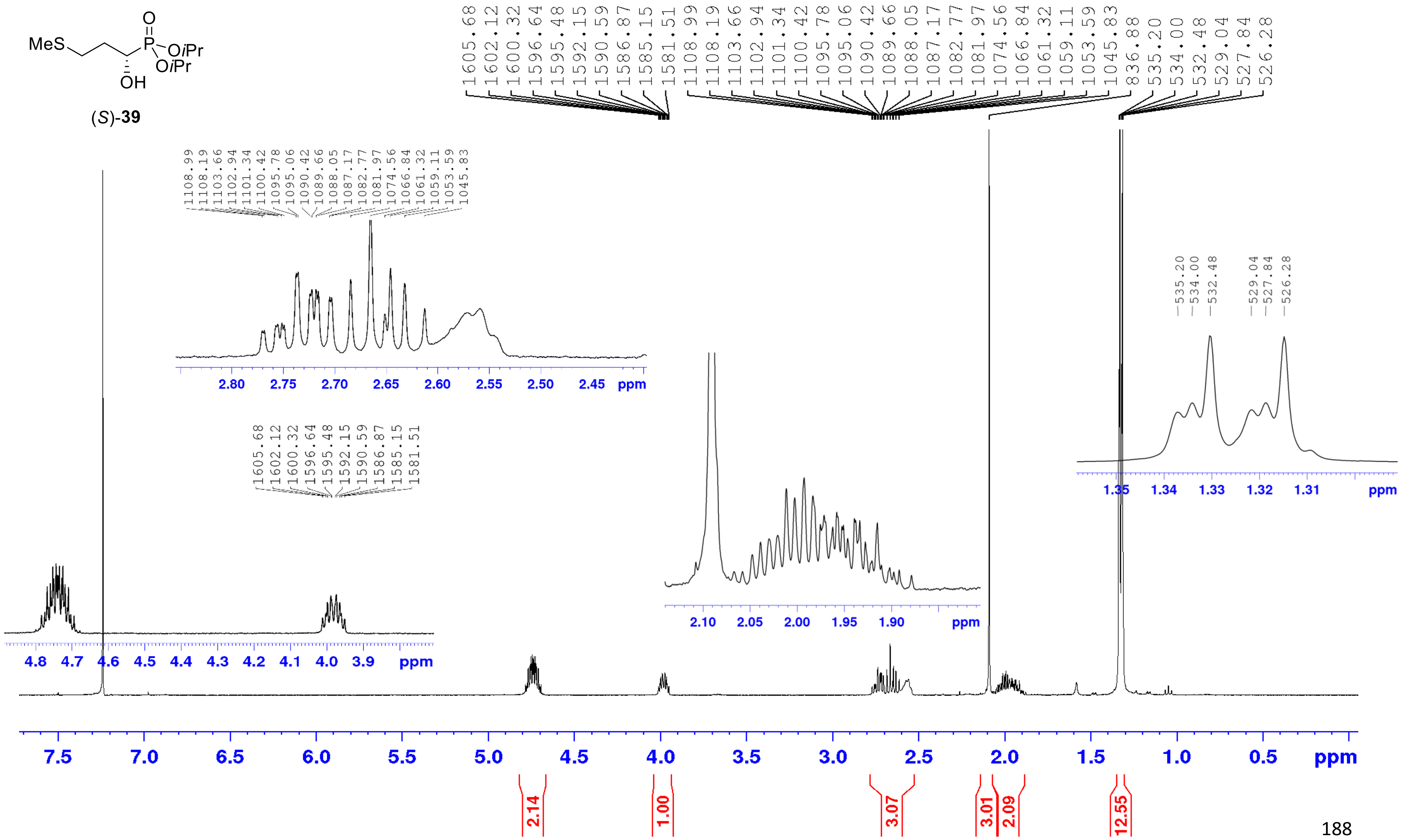

^{13}C NMR of (*S*)-diisopropyl (1-hydroxy-3-methylthiopropyl)phosphonate (150.93 MHz, CDCl$_3$) [(*S*)-39]:

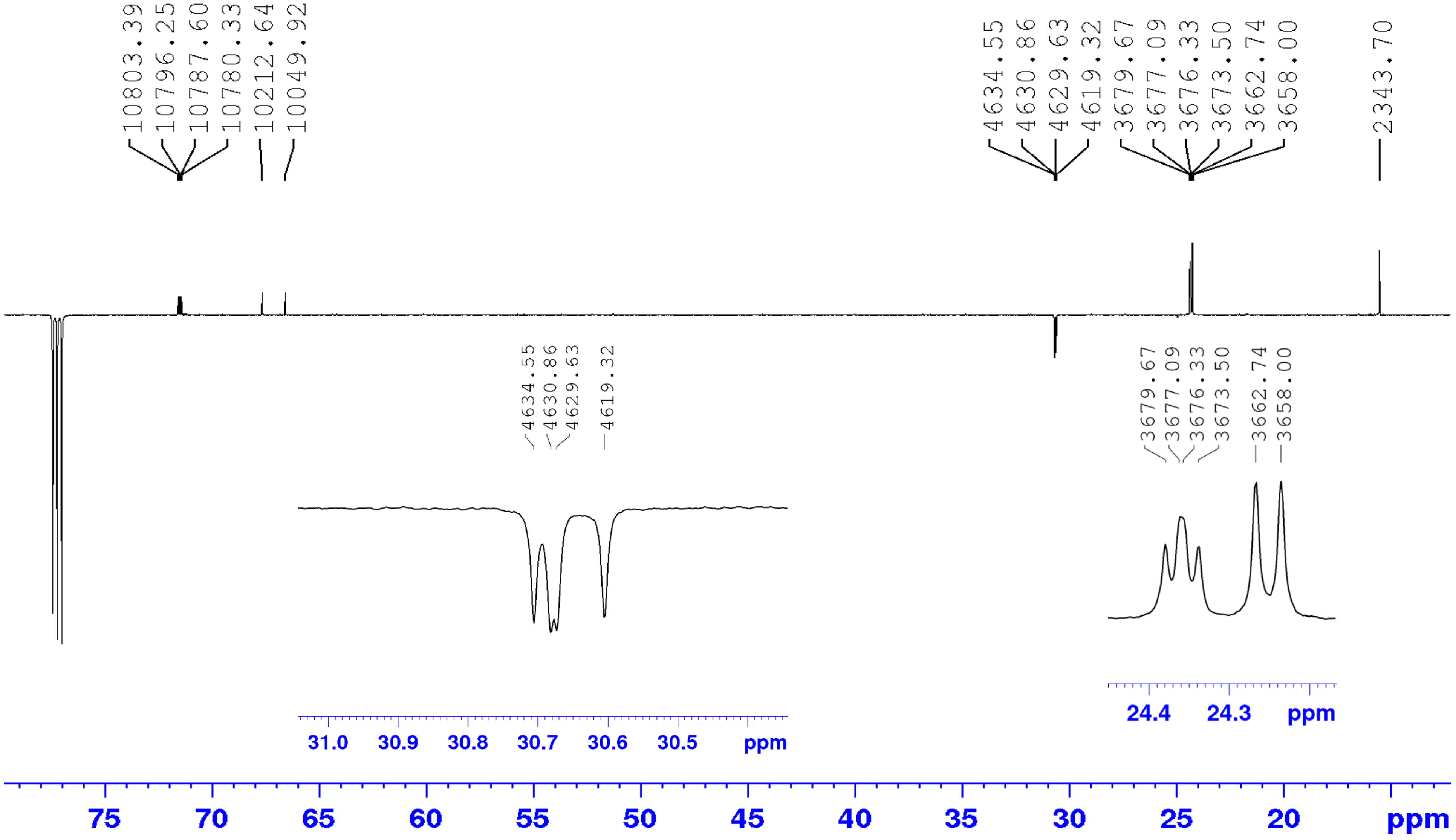

^{31}P NMR of diisopropyl (1-hydroxy-3-methylthiopropyl)phosphonate (162.03 MHz, $CDCl_3$) [(*S*)-39]:

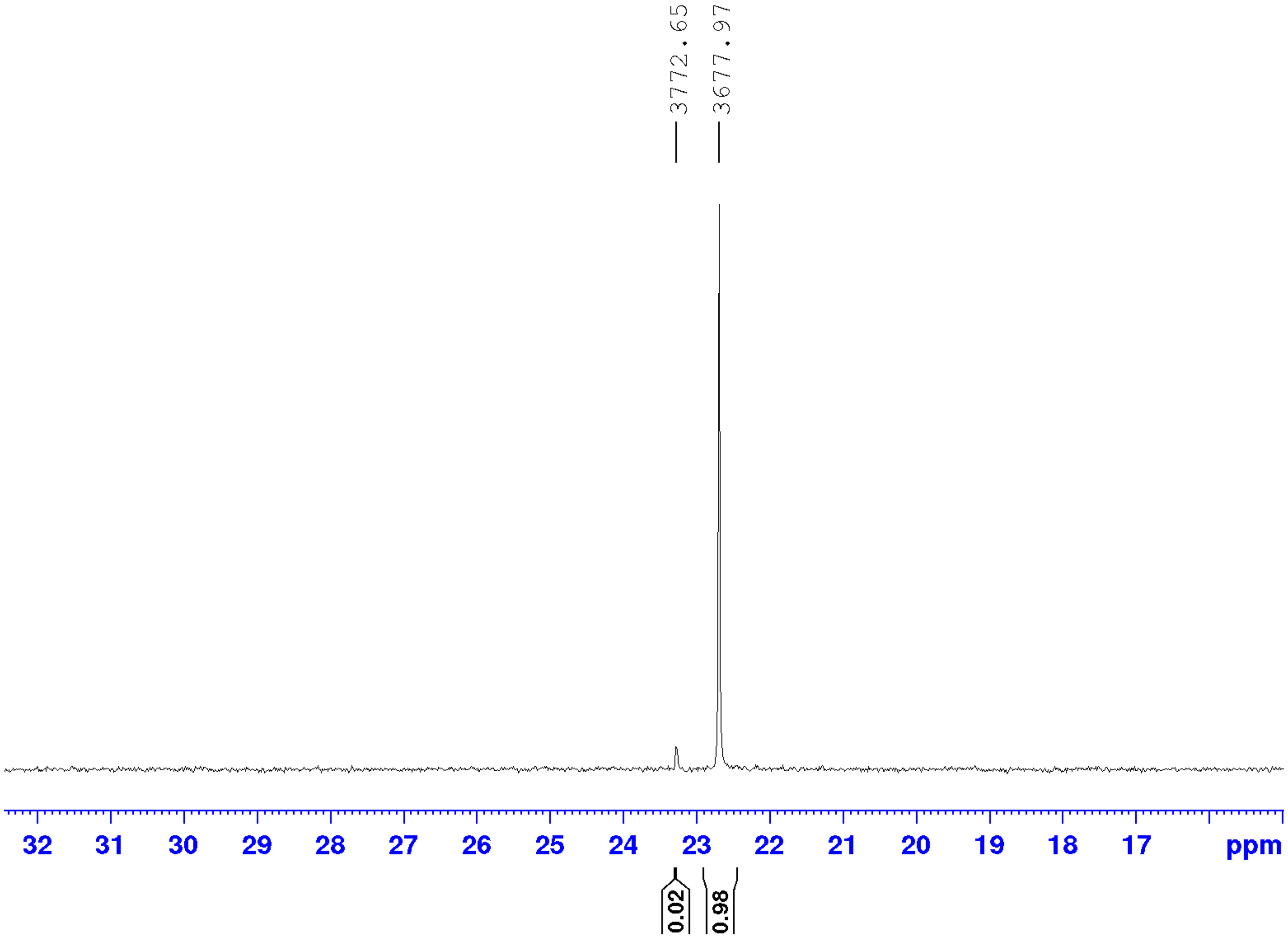

^{1}H NMR of (*R*)-diisopropyl (1-hydroxy-3-methylthiopropyl)phosphonate (400.27 MHz, CDCl₃) [(*R*)-89]:

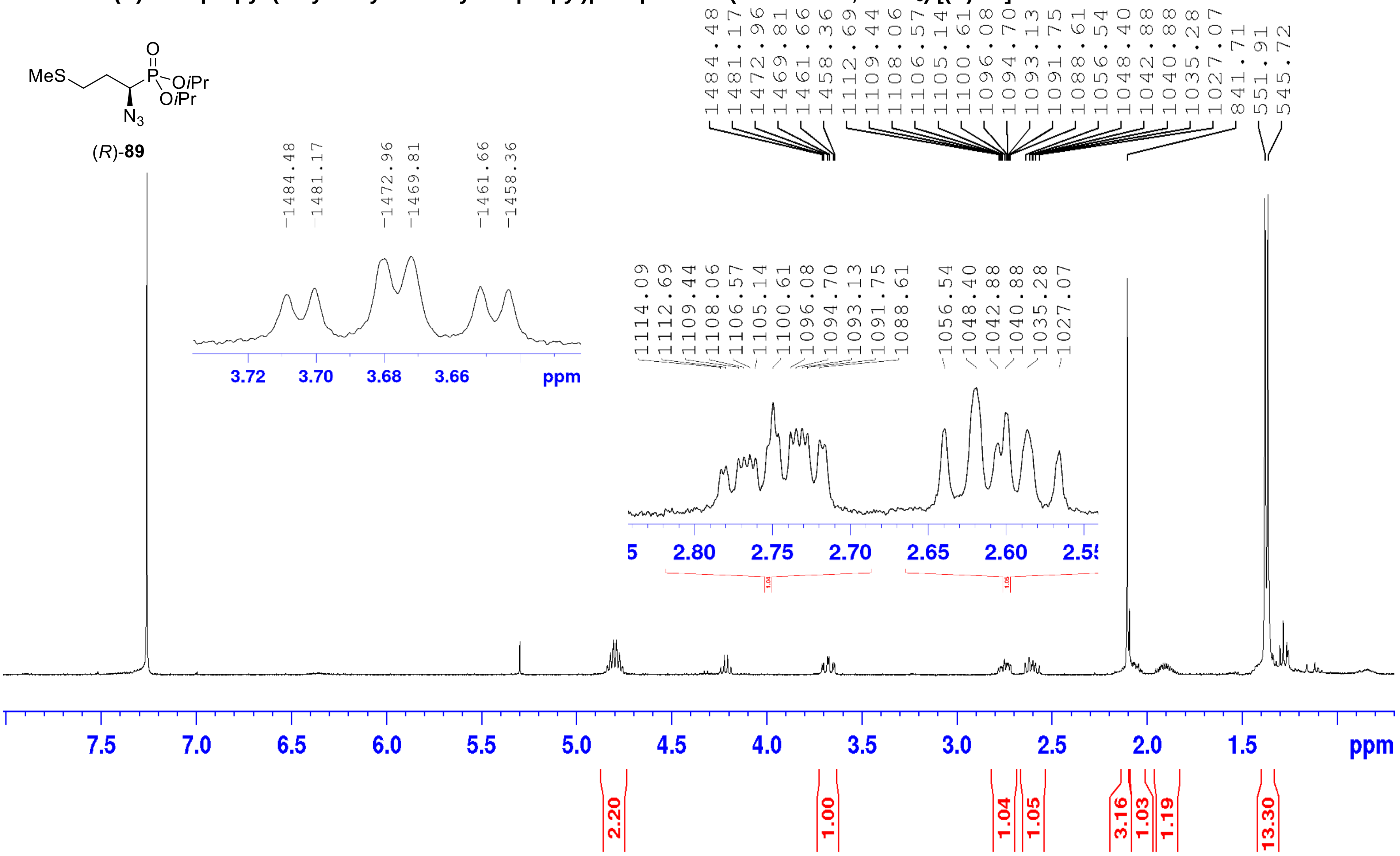

^{31}P NMR of (*R*)-diisopropyl (1-hydroxy-3-methylthiopropyl)phosphonate (162.03 MHz, $CDCl_3$) [(*R*)-89]:

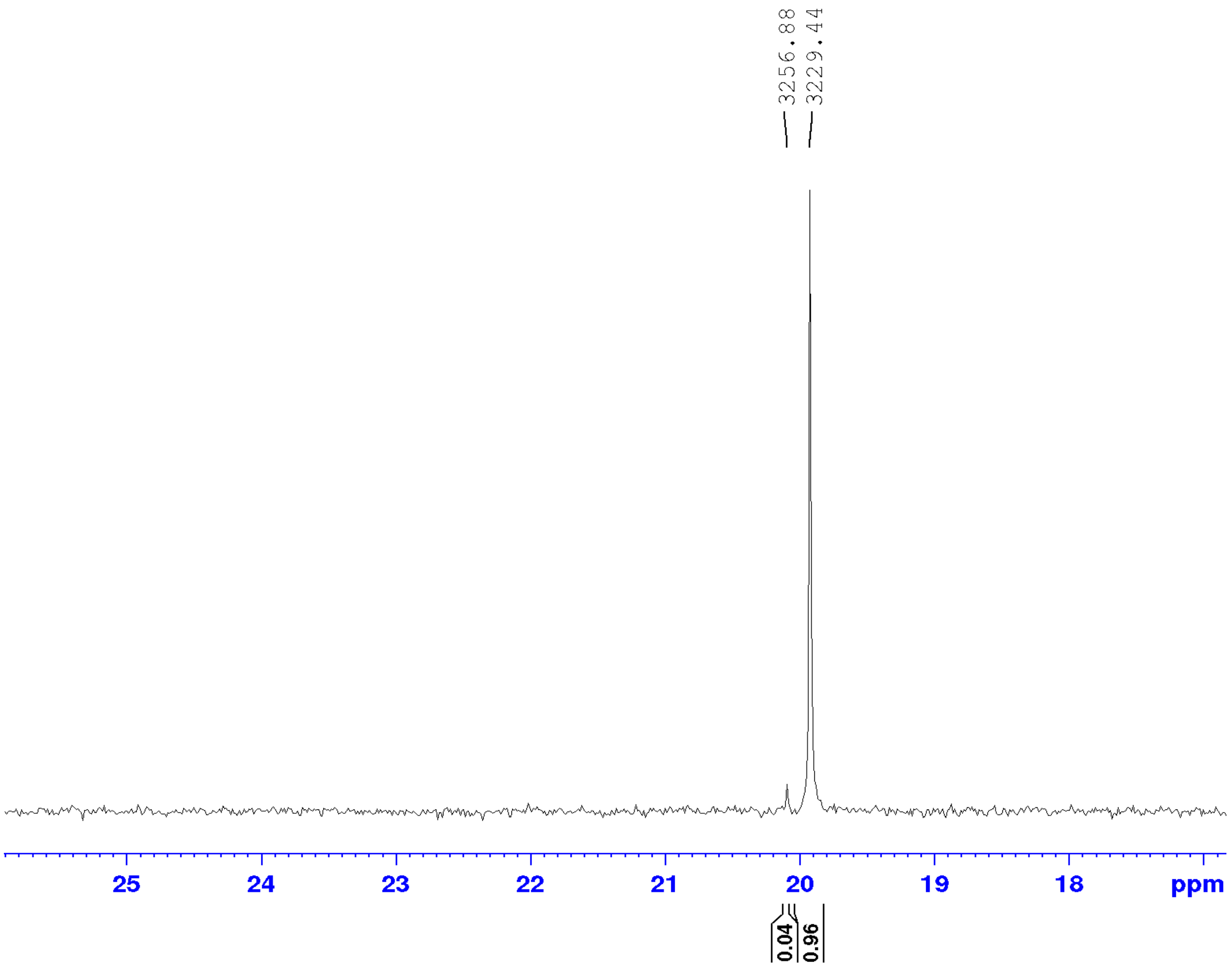

^{31}P NMR of diisopropyl 1-oxo-3-(benzylthio)-propylphosphonate (162. 03 MHz, $CDCl_3$) (23):

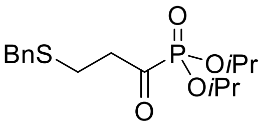

23

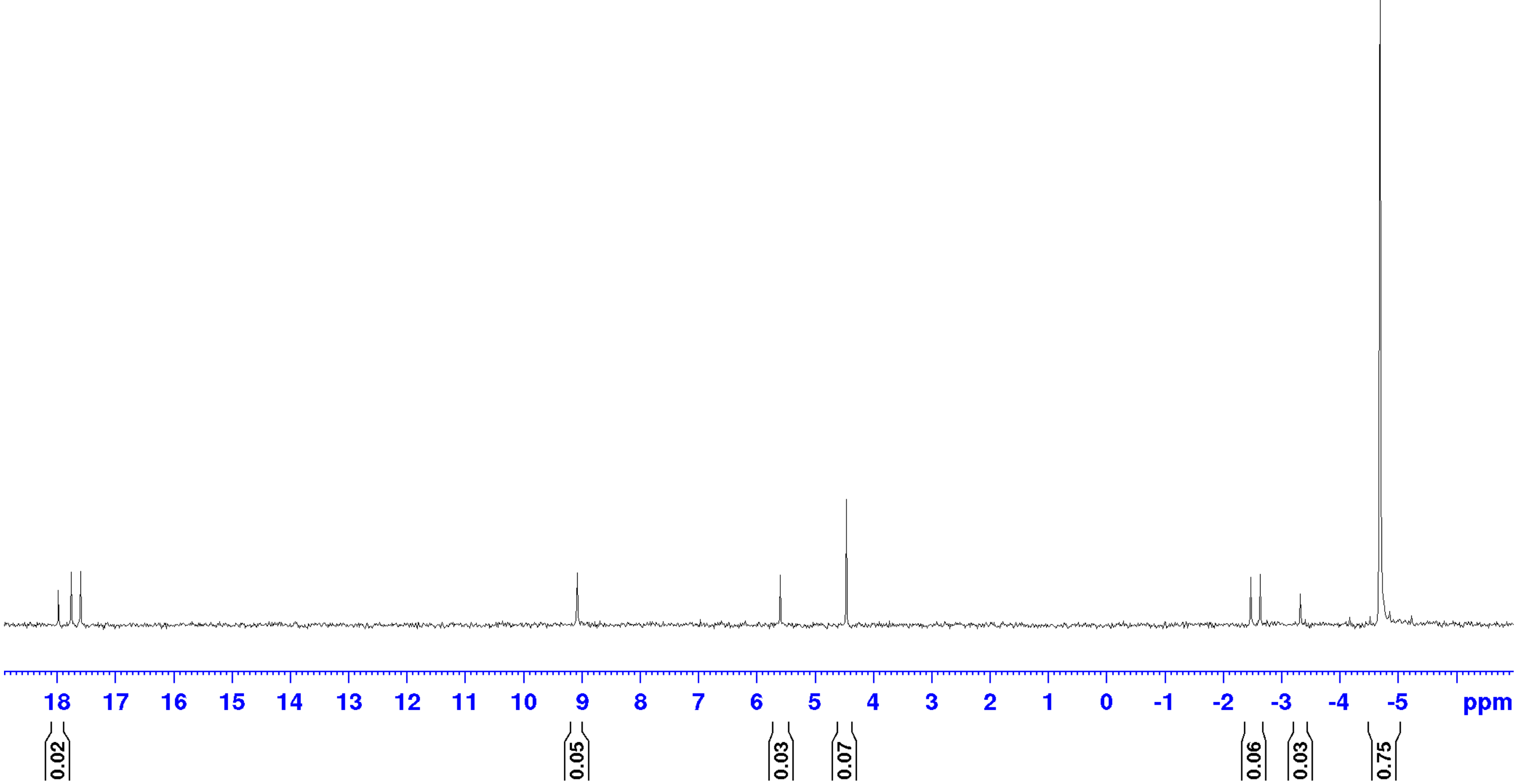

^{1}H NMR of (*S*)-diisopropyl [3-(benzylthio)-1-hydroxypropyl]phosphonate (600.25 MHz, $CDCl_3$) [(*S*)-40]:

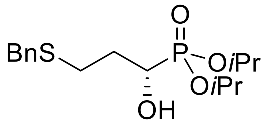

(*S*)-**40**

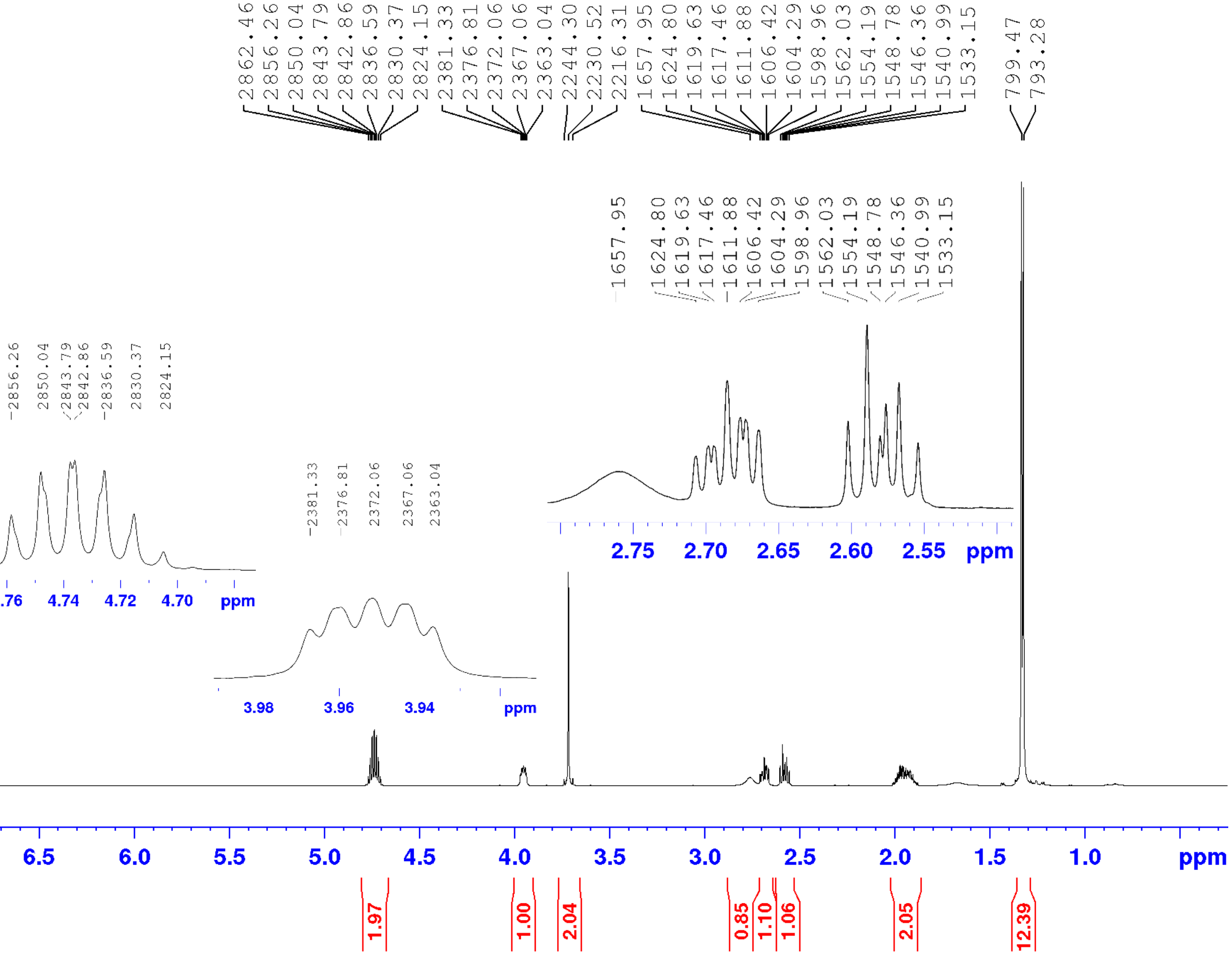

^{31}P NMR of (*S*)-diisopropyl [3-(benzylthio)-1-hydroxypropyl]phosphonate (161.98 MHz, $CDCl_3$) [(*S*)-40]:

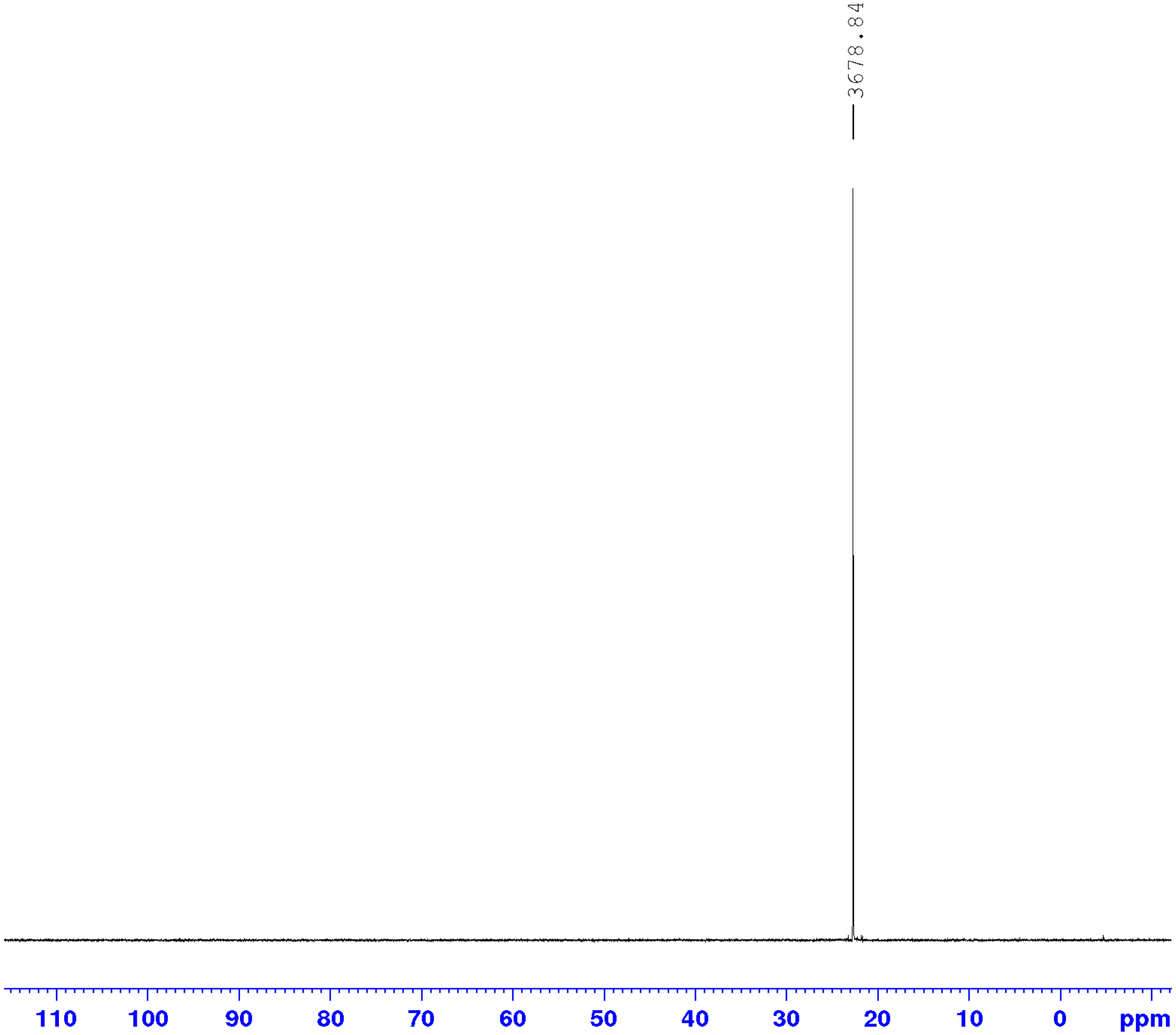

^{13}C NMR of (*S*)-diisopropyl [3-(benzylthio)-1-hydroxypropyl]phosphonate (150.93 MHz, $CDCl_3$) [(*S*)-40]:

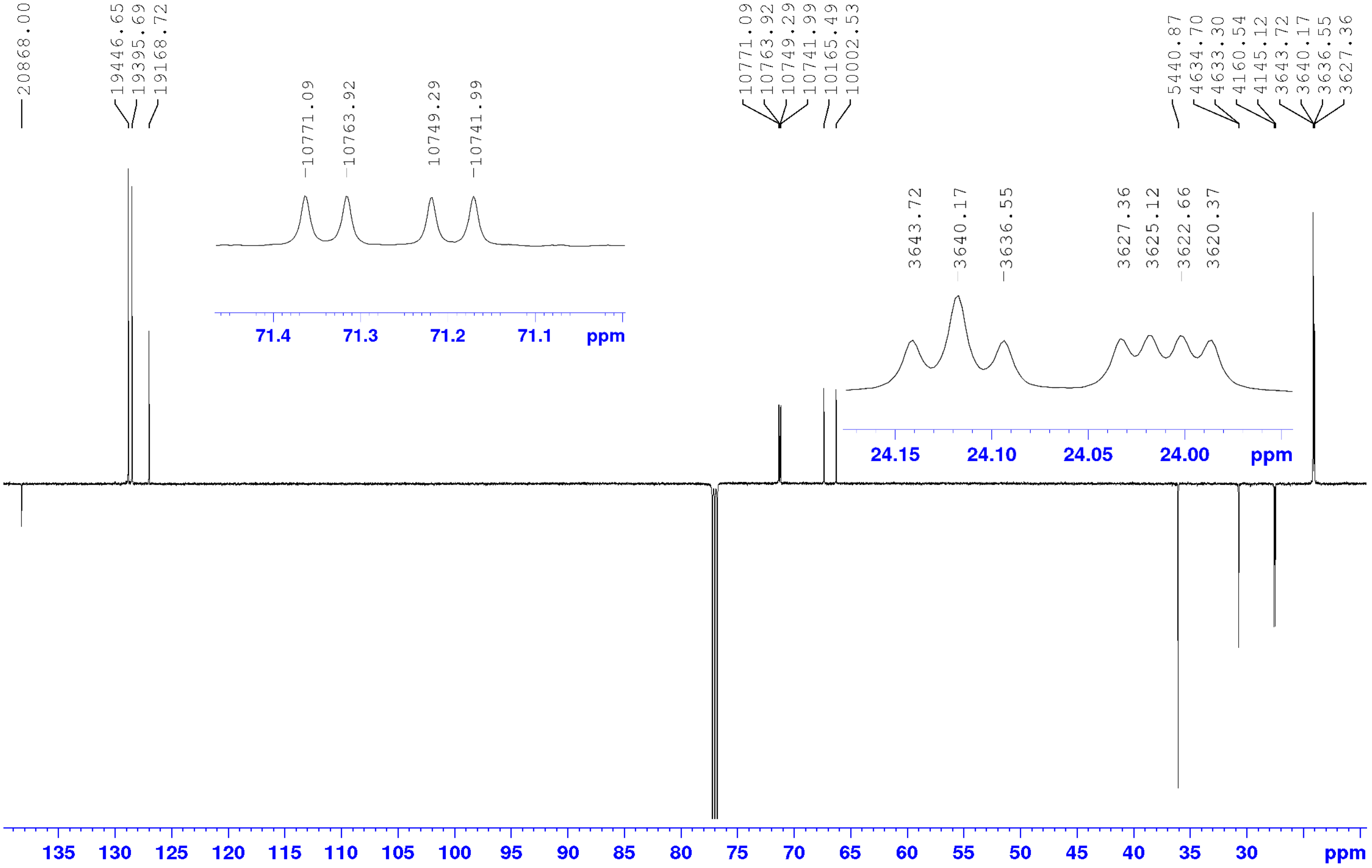

^{1}H NMR of (*R*)-diisopropyl [1-azido-3-(benzylthio)propyl]phosphonate(600.25 MHz, $CDCl_3$) [(*R*)-53]:

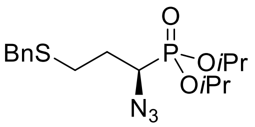

(*R*)-**53**

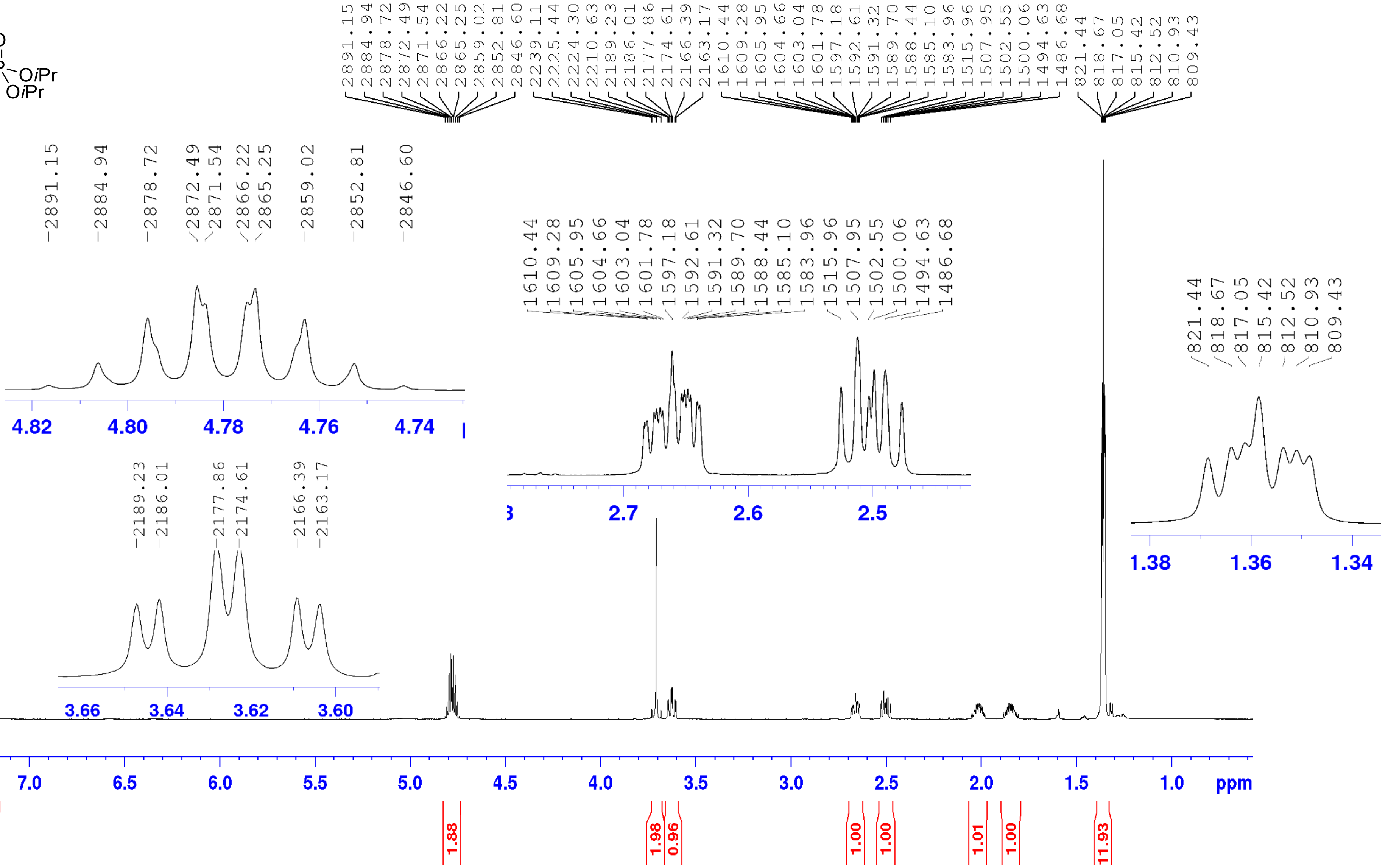

^{31}P NMR of (*R*)-diisopropyl [1-azido-3-(benzylthio)propyl]phosphonate(161.98 MHz, $CDCl_3$) [(*R*)-53]:

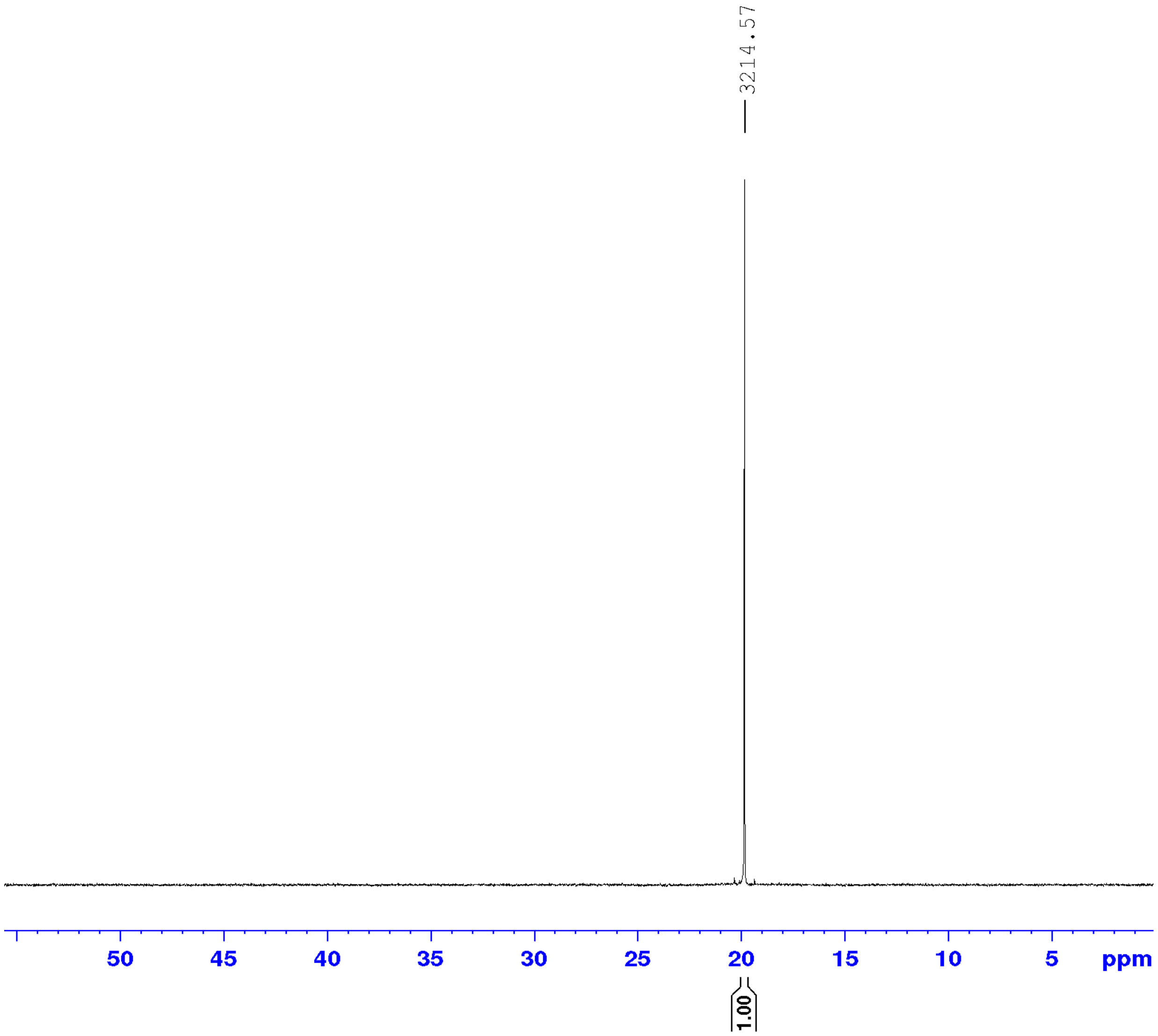

^{13}C NMR of (*R*)-diisopropyl [1-azido-3-(benzylthio)propyl]phosphonate(150.93 MHz, $CDCl_3$) [(*R*)-53]:

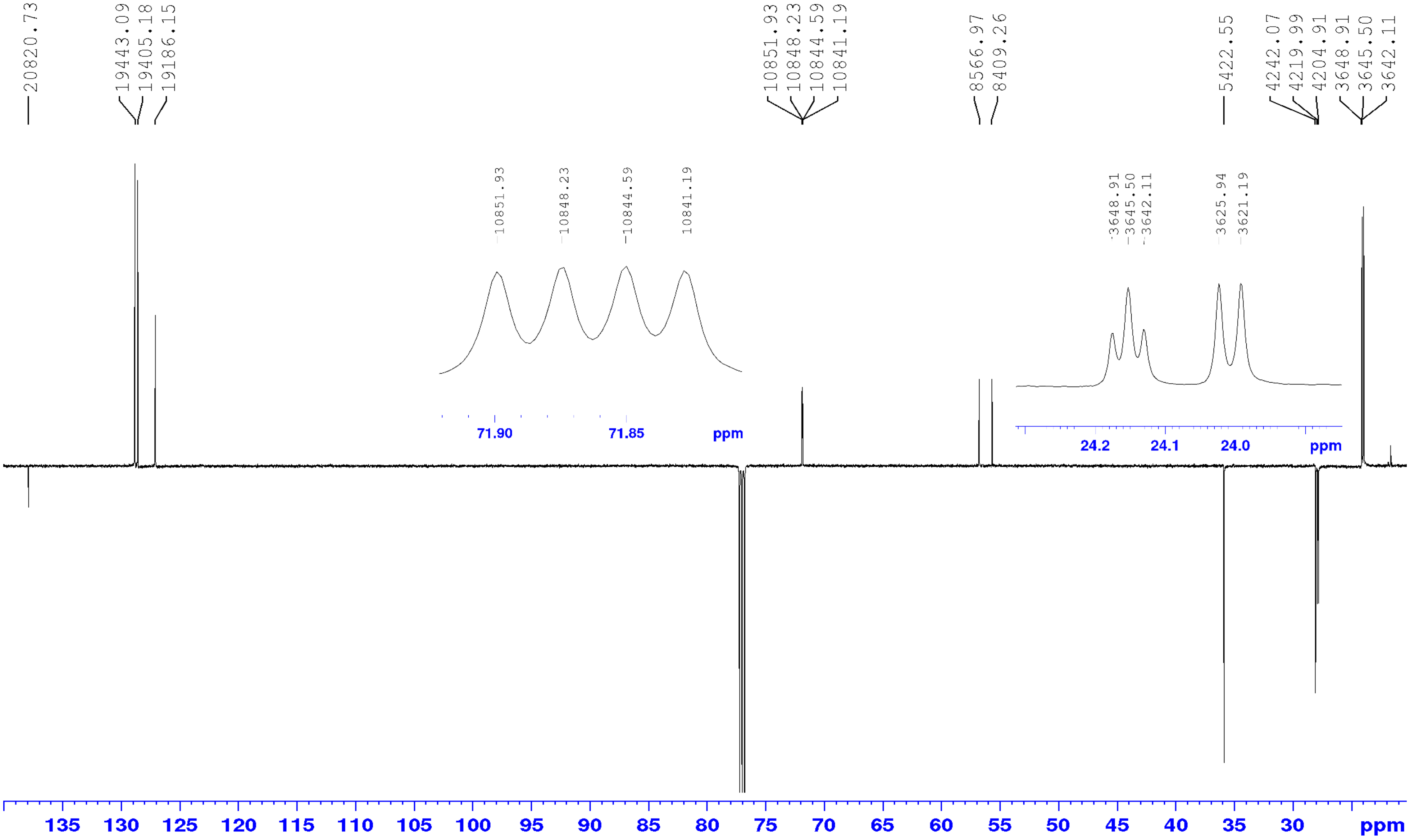

^{1}H NMR of diisopropyl 1-oxo-4-bromobutylphosphonate (400.27 MHz, $CDCl_3$) (24):

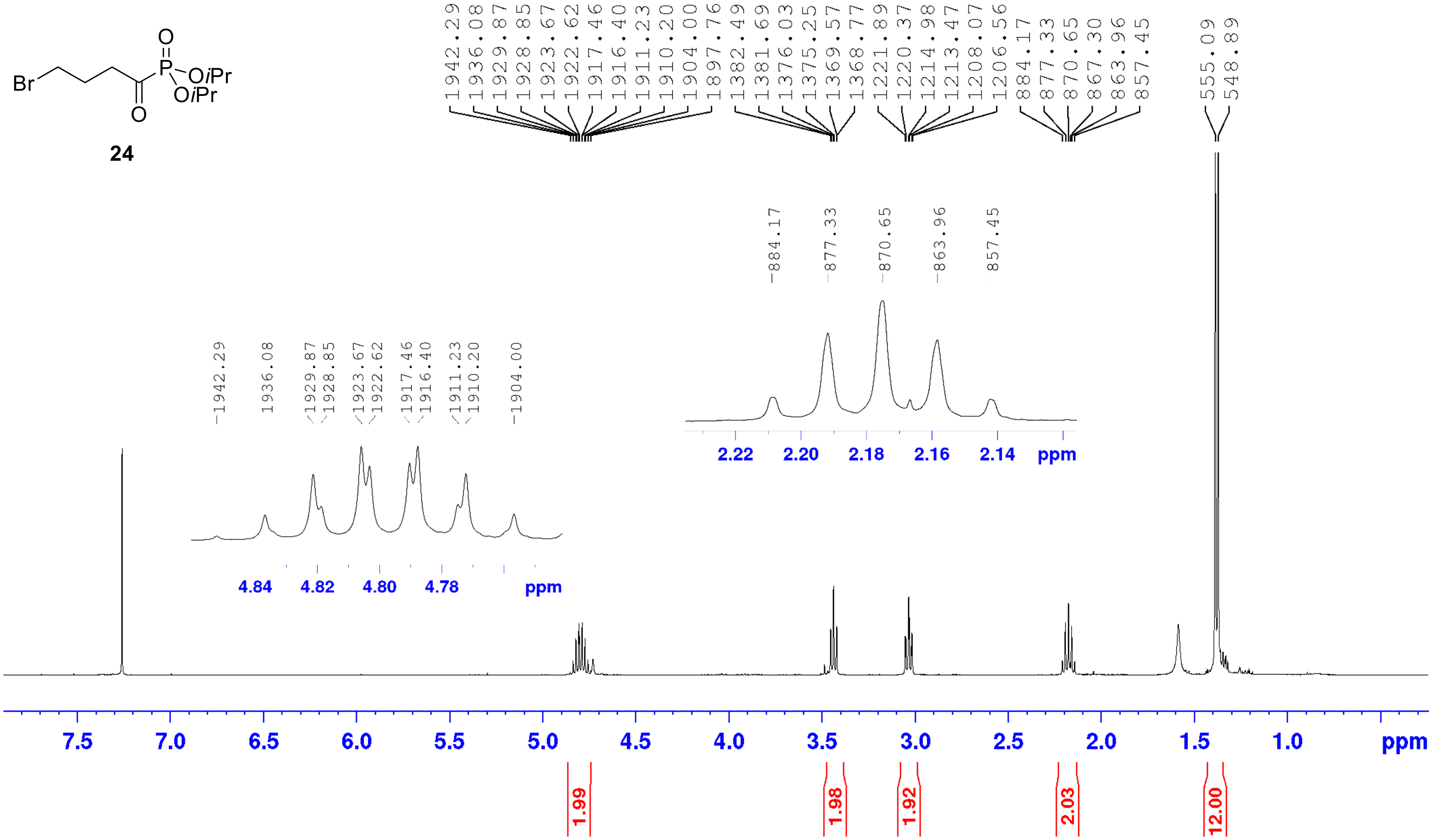

^{31}P NMR of diisopropyl 1-oxo-4-bromobutylphosphonate (162.03 MHz, $CDCl_3$) (24):

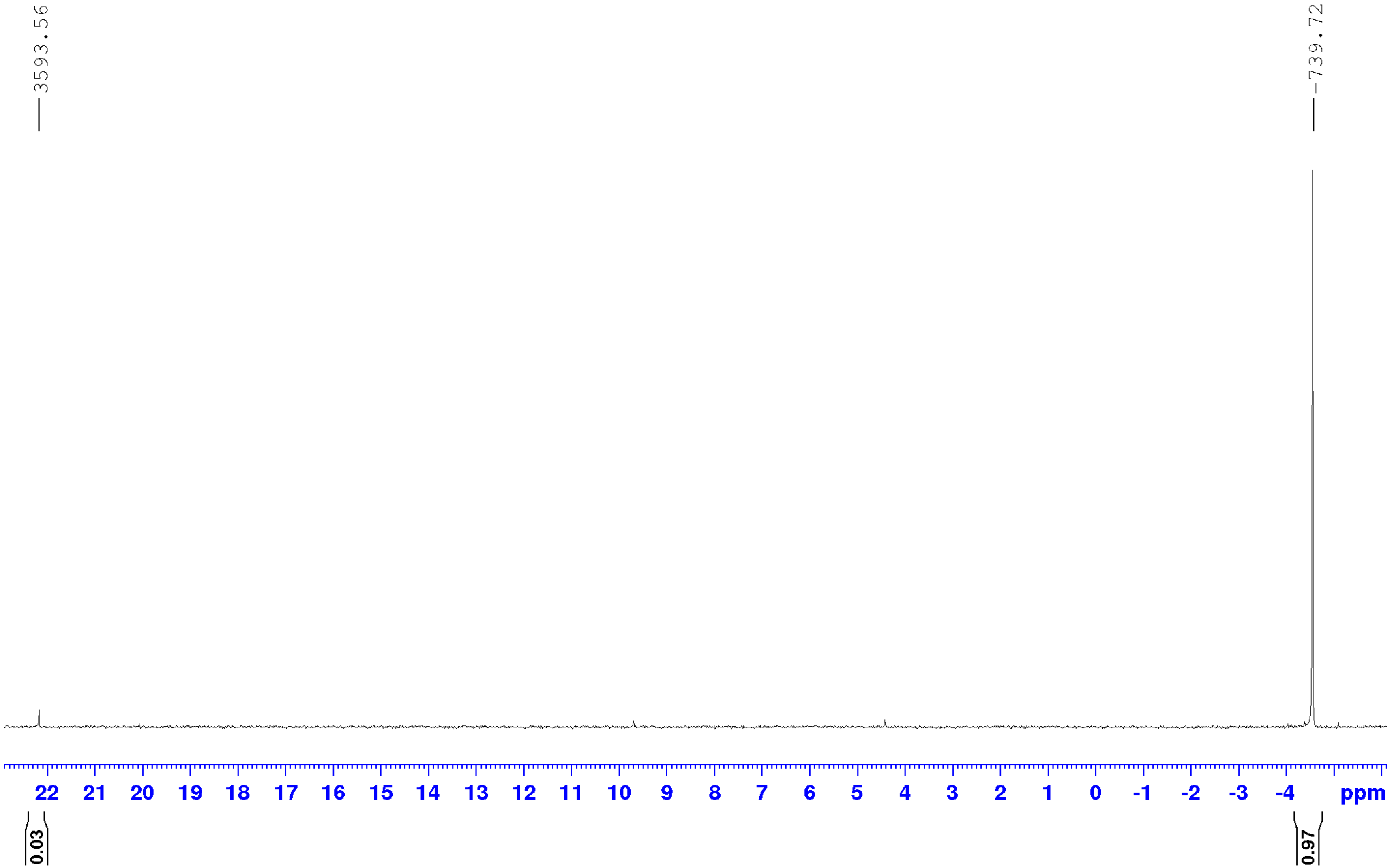

^{1}H NMR of (*S*)-diisopropyl 1-hydroxy-4-bromobutylphosphonate (400.27 MHz, $CDCl_3$) [(*S*)-41]:

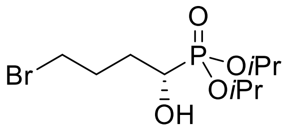

(*S*)-**41**

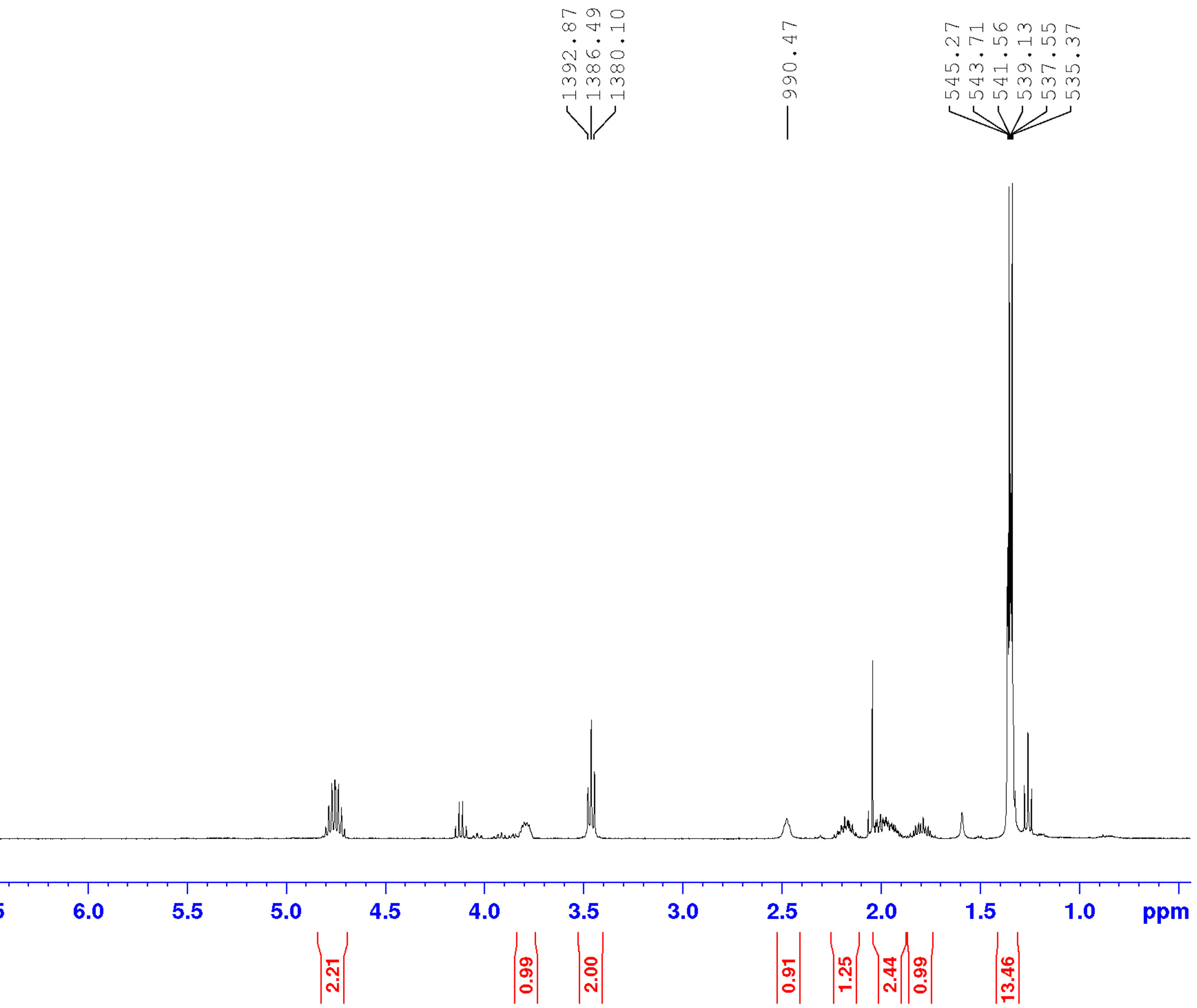

^{31}P NMR of (*S*)-diisopropyl 1-hydroxy-4-bromobutylphosphonate (162.03 MHz, CDCl$_3$) [(*S*)-41]:

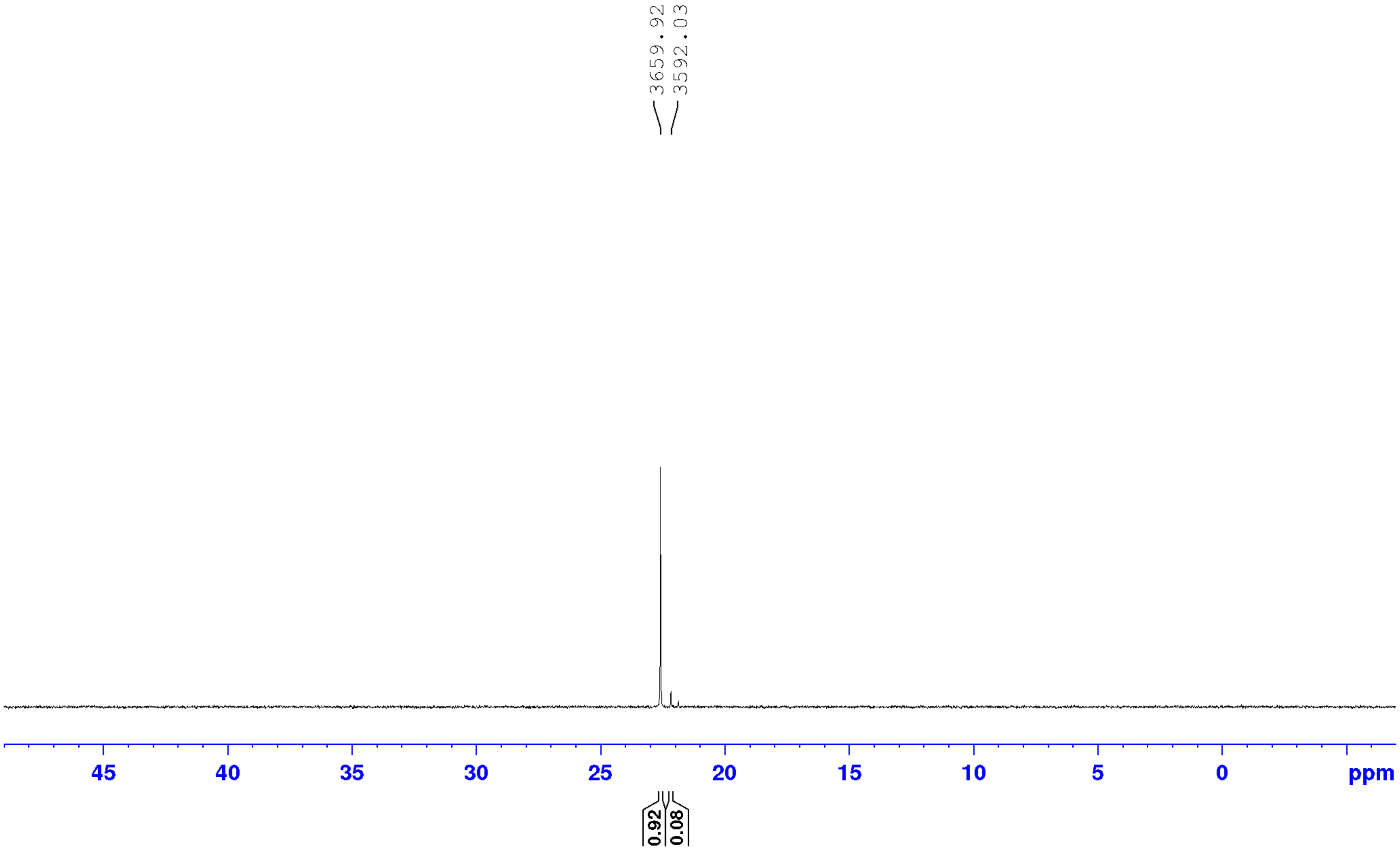

Supplement: Supplementary file 1 — Supporting Information [file CHEM-29-0-s002.pdf]
