# Supplementary material for: Asymmetric Transfer Hydrogenation as a Key Step in the Synthesis of the Phosphonic Acid Analogs of Aminocarboxylic Acids
Source: Chemistry. 2023 Sep 20;29(72):e202302171. doi: 10.1002/chem.202302171 (PMC10947287; doi:10.1002/chem.202302171)
Supplement: Supplementary file 2 — Supporting Information [file CHEM-29-0-s001.pdf]

Chemistry–A European Journal

Supporting Information

Asymmetric Transfer Hydrogenation as a Key Step in the Synthesis of the Phosphonic Acid Analogs of Aminocarboxylic Acids

Tamara Dinhof, Thomas Kalina, Toda Stanković, Kristóf Braunsteiner, Philipp Rohrbach, Ertan Turhan, Andreas Gradwohl, Artur Königshofer, Jeannie Horak, and Katharina Pallitsch*

Supporting Information

Asymmetric Transfer Hydrogenation as a Key Step in the Synthesis of the Phosphonic Acid Analogues to Aminocarboxylic Acids

Tamara Dinhof, Thomas Kalina, Toda Stanković, Kristóf Braunsteiner, Philipp Rohrbach, Ertan Turhan, Andreas Gradwohl, Artur Königshofer, Jeannie Horak and Katharina Pallitsch

Experimental details and compound characterisation

The mentioned general procedures **A**-**H** can be found in the experimental section of the main manuscript. The synthesized compounds are grouped in sequences leading to the respective aminophosphonic acids and can be found on the pages denoted below.

1. Phosphaalanine 2
2. Phosphavaline 4
3. Phosphaleucine 6
4. Phosphaisoleucine 8
5. Phosphalysine 10
6. Phosphaarginine (precursor) 13
7. Phosphaphenylalanine 15
8. Phosphatryptophane 17
9. Phosphatyrosine 19
10. Phosphaserine 21
11. Phosphaasparagine (first step) 23
12. Phosphaglutamic acid 24
13. Phosphaglutamine 26
14. Phosphaisoserine (labeled) 28
15. 4-Amino-1-hydroxybutylphosphonic acid 31
16. Phosphacysteine 33
17. Phosphamethionine (intermediate) 37
18. Phosphaproline and Phosphaornithine (precursor) 40

1. Phosphaalanine

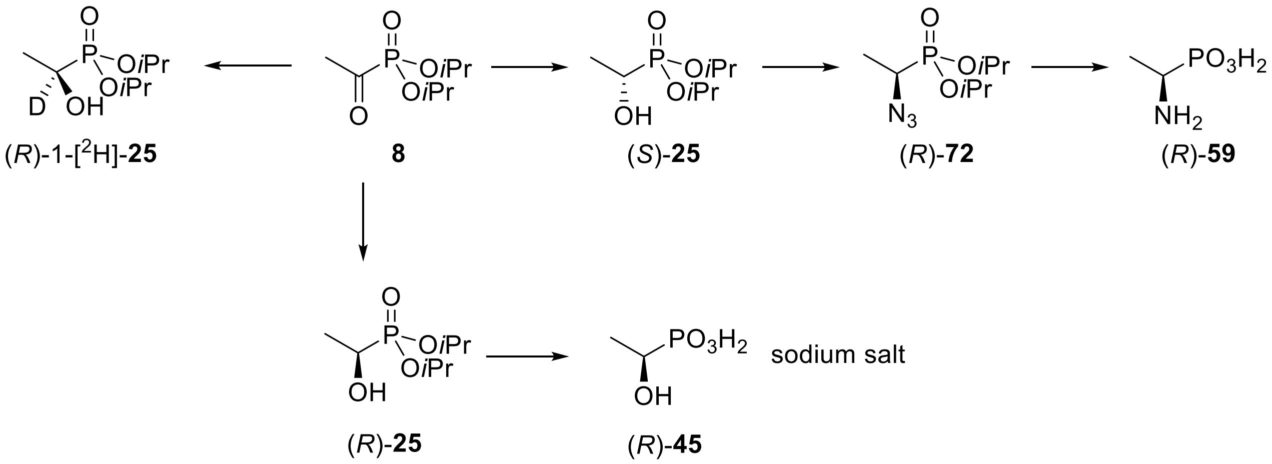

Diisopropyl acetylphosphonate (8): Ketophosphonate **8** was obtained starting from acetyl chloride (392 mg, 5.0 mmol, 0.36 mL) following **general procedure A** at 0°C within 3 h. A small analytical sample was purified by bulb-to-bulb distillation (120°C, 5 Torr); ^{1}H NMR (400.13 MHz, $CDCl_3$): δ=4.84 – 4.73 (m, 2H, 2 × CH *i*Pr), 2.47 (d, $^{2}J_{HP}$=4.9 Hz, 3H, $CH_3C(O)$-P), 1.37 ppm (d, $^{3}J_{HH}$=6.2 Hz, 12H, 4 × CH_3 *i*Pr); ^{31}P NMR (161.98 MHz, $CDCl_3$): δ=4.45 (s, ∫0.02, unknown impurity), −3.34 (s, ∫0.04, unknown impurity), −4.43 ppm (s, ∫0.94, **8**).

(*S*)-Diisopropyl 1-hydroxy-ethylphosphonate [(*S*)-25]: (*S*)-**25** was obtained from crude keto-phosphonate **8** (approx. 4.70 mmol[1]) following **general procedure D** within 16 h using (*R,R*)-**7** (0.01 equiv., see **general procedure C**) as catalyst. The crude residue was purified by MPLC using a solvent gradient (*n*-heptane/EtOAc, from 18 to 100% EtOAc) to give the desired product as colorless oil (978 mg, 4.65 mmol, 99%); R_f=0.18 (EtOAc); α_D^{20}=+9.7 (*c*=1.10 in acetone); ^{1}H NMR (500.32 MHz, $CDCl_3$): δ=4.79 – 4.65 (m, 2H, 2 × CH *i*Pr), 3.93 (qd, $^{3}J_{HH}$=7.1 Hz, $^{2}J_{HP}$=3.2 Hz, 1H, CH-P), 2.87 (s, 1H, OH), 1.40 (dd, $^{3}J_{HH}$=7.1 Hz, $^{2}J_{HP}$=17.2 Hz 3H, C$\underline{H_3}$CH-P), 1.32 (d, $^{3}J_{HH}$=6.4 Hz, 3H, CH_3 *i*Pr), 1.31 (d, $^{3}J_{HH}$=6.2 Hz, 3H, CH_3 *i*Pr), 1.31 ppm (d, $^{3}J_{HH}$=6.3 Hz, 6H, 2 × CH_3 *i*Pr); ^{31}P NMR (162.03 MHz, $CDCl_3$): δ=23.73 ppm (s); ^{13}C NMR (176.12 MHz, $CDCl_3$): δ=71.4 (d, $^{2}J_{CP}$=7.1 Hz, CH *i*Pr), 71.1 (d, $^{2}J_{CP}$=7.3 Hz, CH *i*Pr), 64.4 (d, $^{1}J_{CP}$=162.9 Hz, CH-P), 24.3 (d, $^{3}J_{CP}$=3.5 Hz, CH_3 *i*Pr), 24.3 (d, $^{3}J_{CP}$=3.5 Hz, CH_3 *i*Pr), 24.2 (d, $^{3}J_{CP}$=4.9 Hz, CH_3 *i*Pr), 24.1 (d, $^{3}J_{CP}$=4.9 Hz, CH_3 *i*Pr), 17.4 ppm (s, $\underline{C}H_3$-CH(OH)-P), IR (ATR): $\tilde{\nu}$=2981, 1376, 1218, 1106, 992, 911, 734, 631 cm^{-1}; HRMS (ESI): calc. for $[C_8H_{19}O_4P+Na]^+$: *m/z* 233.0913 $[M+Na]^+$; found: *m/z* 233.0912; chiral stationary phase HPLC after derivatization to the respective nitrobenzoate (Chiralpak® OH-QD-AX, 250 × 4.6 mm, UV-detection (254 nm), *n*-heptane + 0.1% *i*PrOH/*i*PrOH, 80:20; R_T [(*R*)-enantiomer]=21.74 min (∫0.6), R_T [(*S*)- -enantiomer]=24.36 min (∫99.4) ; *ee* ≥ 98%.

---

[1] The used amount of α-oxo-phosphonate is estimated by ^{31}P NMR spectroscopy.

(*R*)-Diisopropyl 1-hydroxy-ethylphosphonate [(*R*)-25]: (*R*)-**25** can be obtained by the same procedure as the (*S*)-enantiomer by using (*S,S*)-**7** as catalyst. The obtained analytical data are in agreement for both enantiomers; α_D^{20}=−10.1 (*c*=0.97 in acetone); ^{31}P NMR (161.98 MHz, d_8-toluene) with chiral solvating agent: δ=94.42 [∫0.54, chiral solvating agent], 24.34 [∫0.46, complex of chiral solvating agent with (*R*)-**25**], 24.14 ppm [∫0.003, complex of chiral solvating agent with (*S*)-**25**]; *ee* ≥ 98%.[1]

(*R*)-1-Hydroxyethylphosphonic acid sodium salt [(*R*)-45]: (*R*)-Hydroxyphosphonate **25** (300 mg, 1.43 mmol) was deprotected using **general procedure H**, with 1,2-dichloroethane as solvent within 5 h at 60°C to give (*R*)-**45** (243 mg, 1.41 mmol, 99%)[2] as colorless solid after lyophilisation; α_D^{20}=−8.1 (*c*=0.91 in water), lit. for the cyclohexylamine salt of (*R*)-**45**: α_D^{20}=−1.38 (*c*=0.735 in H_2O);[9] ^{1}H NMR (700.40 MHz, D_2O): δ=3.82-3.75 (m, 1H, CH), 1.33 ppm (dd, $^3J_{PH}$=15.0 Hz, $^3J_{HH}$=7.1 Hz, 3H, CH_3); ^{31}P NMR (162.03 MHz, D_2O): δ=19.49 ppm (s); ^{13}C NMR (176.12 MHz, D_2O): δ=65.8 (d, $^1J_{CP}$=153.4 Hz, CH), 17.47 ppm (s, CH_3); IR (ATR): $\tilde{\nu}$=3209, 2303, 1659, 1372, 1052, 979, 879, 705 cm^{-1}; HRMS (ESI): calc. for the dimer $C_2H_7O_4P$-H^-: 251.0091 [2*M*-H]$^-$; found: 251.0088.

(*R*)-Diisopropyl 1-[^{2}H]-1-hydroxy-ethylphosphonate {(*R*)-1-[^{2}H]-25}: (*R*)-1-[^{2}H]-**25** was obtained from crude keto-phosphonate **8** (approx. 0.50 mmol[1]) following **general procedure D** within 16 h using (*S,S*)-**7** (0.01 equiv., see **general procedure C**) as catalyst. Adapted amounts of reagents are used in this case as follows: deuterated formic acid (61 mg, 1.30 mmol, 0.05 mL, 2.6 equiv.) and Et_3N (132 mg, 1.30 mmol, 0.18 mL, 2.6 equiv.). The crude residue was purified by MPLC using a solvent gradient (*n*-heptane/EtOAc, from 18 to 100% EtOAc) to give the desired product as colorless oil (104 mg, 0.49 mmol, 99%);^{1}H NMR (400.27 MHz, $CDCl_3$): δ=4.82 – 4.68 (m, 2H, 2 × CH *i*Pr), 2.22 (br d, $^3J_{HP}$=5.1 Hz, 1H, OH), 1.41 (d, $^2J_{HP}$=17.3 Hz, 3H, C$\underline{H_3}$CH-P), 1.35 (br d, $^3J_{HH}$=6.1 Hz, 6H, 2 × CH_3 *i*Pr), 1.34 ppm (d, $^3J_{HH}$=6.2 Hz, 6H, 2 ×CH_3 *i*Pr), ^{31}P NMR (162.03 MHz, $CDCl_3$): δ=23.73 ppm (s); ^{13}C NMR (176.12 MHz, $CDCl_3$): δ=71.2 (d, $^2J_{CP}$=7.5 Hz, CH *i*Pr), 70.9 (d, $^2J_{CP}$=7.6 Hz, CH *i*Pr), 63.70 (dt, $^1J_{CP}$=162.7 Hz, $^1J_{CD}$=21.9 Hz, CD-P), 24.1 (d, $^3J_{CP}$=3.3 Hz, CH_3 *i*Pr), 24.0 (d, $^3J_{CP}$=4.0 Hz, CH_3 *i*Pr), 23.98 (d, $^3J_{CP}$=4.7 Hz, CH_3 *i*Pr), 23.95 (d, $^3J_{CP}$=4.5 Hz, CH_3 *i*Pr), 17.1 ppm (s, $\underline{C}H_3$-CD(OH)-P); HRMS (ESI): calc. for $[C_8H_{18}DO_4P+Na]^+$: *m/z* 234.0976 [M+Na]$^+$; found: *m/z* 234.0977; all other analytical data were in agreement to the non-deuterated compound.

(*R*)-Diisopropyl 1-azido-ethylphosphonate [(*R*)-72]: Substitution of the hydroxyl group by an azide was accomplished following **general procedure E**, starting from (*S*)-**25** (104 mg, 0.49 mmol), dissolved in a 1:2 mixture of toluene and CH_2Cl_2, and using DIAD (di-isopropyl-

[2] The yield was determined by ^{31}P-NMR, by addition of an internal standard (2-AEP). The concentration was 5.8 µmol (*R*)-**45**/mg lyophilizate.

azodicraboxylate, 144 mg, 0.71 mmol, 0.14 mL) as the azoester component. Completion of the reaction was observed after 16 h at room temperature. The crude residue was purified by column chromatography (CH_2Cl_2/Et_2O, 95:5) to give the desired product as colorless oil (90 mg, 0.38 mmol, 77%); R_f=0.69 (CH_2Cl_2/Et_2O, 9:1); α_D^{20}=−9.93 (*c*=1.01 in acetone); ^{1}H NMR (400.27 MHz, $CDCl_3$): δ=4.81-4.70 (m, 2H, 2 × CH *i*Pr), 3.45 (qd, $^3J_{HH}$=7.3 Hz, $^2J_{HP}$=11.9 Hz, 1H, CH-P), 1.44 (dd, $^3J_{PH}$=16.9 Hz, $^3J_{HH}$=7.3 Hz 3H, C<u>H3</u>CH(N_3)-P), 1.34 (d, $^3J_{HH}$=6.2 Hz, 9H, 3 × CH_3 *i*Pr), 1.33 ppm (d, $^3J_{HH}$=6.2 Hz, 3H, CH_3 *i*Pr); ^{31}P NMR (162.03 MHz, $CDCl_3$): δ=20.63 ppm (s); ^{13}C NMR (150.93 MHz, $CDCl_3$): δ=71.8 (d, $^2J_{CP}$=7.3 Hz, CH *i*Pr), 71.7 (d, $^2J_{CP}$=7.0 Hz, CH *i*Pr), 52.7 (d, $^1J_{CP}$=159.4 Hz, CH-P), 24.2 (d, $^3J_{CP}$=3.6 Hz, CH_3 *i*Pr), 24.2 (d, $^3J_{CP}$= 3.7 Hz, CH_3 *i*Pr), 24.0 (d, $^3J_{CP}$=4.6 Hz, CH_3 *i*Pr), 24.00 (d, $^3J_{CP}$=4.9 Hz, CH_3 *i*Pr), 14.1 ppm (d, $^2J_{CP}$=1.4 Hz, C<u>H3</u>-CH(N_3)-P); IR (ATR): $\tilde{\nu}$=2981, 2086, 1376, 1270, 1232, 1105, 978 cm^{-1}; HRMS (ESI): calc. for $[C_8H_{18}N_3O_3P+Na]^+$: *m/z* 258.0978 $[M+Na]^+$; found: *m/z* 258.0984; elemental analysis calc. (%) for $C_8H_{18}N_3PO_3$: C 40.85, H 7.71; found: C 40.99, H 7.64.

(*R*)-1-Amino-ethylphosphonic acid, (*R*)-phosphaalanine [(*R*)-59]: (*R*)-Azidophosphonate **69** (56 mg, 0.24 mmol), could be transformed to (*R*)-phosphaalanine (24 mg, 0.19 mmol, 79%) by **general procedure F**, followed by **general procedure G** (reaction time 5 h) as colorless needles; R_f=0.23 [*i*PrOH/H_2O/NH_3 (aq., 25%), 6:3:1]; m.p. 274-278°C; α_D^{20}=−7.33 (*c*=0.90 in water); ^{1}H NMR (400.27 MHz, D_2O): δ=3.43 (qd, $^3J_{HH}$=7.2 Hz, $^3J_{PH}$=12.9, 1H, CH), 1.47 ppm (dd, $^3J_{HH}$=7.2 Hz, $^3J_{PH}$=15.1 Hz, 3H, CH_3); ^{31}P NMR (162.03 MHz, D_2O): δ=14.71 ppm (s); ^{13}C NMR (100.65 MHz, D_2O): δ=44.6 (d, $^1J_{CP}$=146.2 Hz, CH), 13.5 ppm (d, $^2J_{CP}$=2.5 Hz, CH_3); IR (ATR): $\tilde{\nu}$=3419, 2485, 1206, 891 cm^{-1}; HRMS (ESI): calc. for the dimer $[C_2H_8NO_3P+H]^+$: *m/z* 251.0556 $[2M+H]^+$; found: *m/z* 251.0555; elemental analysis calc. (%) for $C_2H_8NPO_3$: C 19.21, H 6.45; found: C 19.21, H 6.54.

2. Phosphavaline

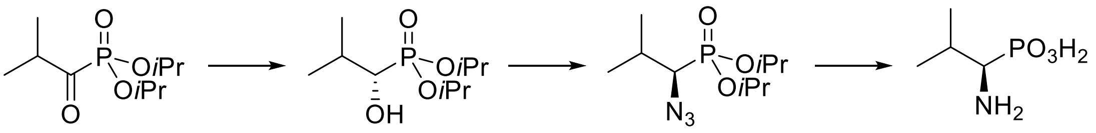

9 (*S*)-**26** (*R*)-**73** (*R*)-**60**

Diisopropyl isobutyrylphosphonate (9): Ketophosphonate **9** was obtained starting from isobutyryl chloride (533 mg, 5.0 mmol, 0.52 mL) by following **general procedure A** at 0°C within 2.5 h; ^{1}H NMR (400.27 MHz, $CDCl_3$): δ=4.84-4.72 (m, 2H, 2 × CH *i*Pr), 3.18 (septd, $^3J_{HH}$=6.8 Hz, $^2J_{PH}$=0.7 Hz, 1H, C<u>H</u>(CH_3)$_2$), 1.35 (d, $^3J_{HH}$=6.3 Hz, 12H, 4 × CH_3 *i*Pr), 1.17 ppm (dd, $^3J_{HH}$=6.8 Hz, $^4J_{HP}$=0.7 Hz 6H, 2 × CH_3); ^{31}P NMR (162.03 MHz, $CDCl_3$): δ=139.48 (s, ∫0.02, (O*i*Pr)$_3$P), 4.40 (s, ∫0.09, HP(O)(O*i*Pr)$_2$), −3.37 (s, ∫0.02, unknown impurity), −4.20 ppm (s, ∫0.88, **9**).

(*S*)-Diisopropyl 1-hydroxy-2-methylpropylphosphonate [(*S*)-26]: (*S*)-**26** was obtained from crude keto-phosphonate **9** (approx. 5.0 mmol[1]) following **general procedure D** within 18 h using (*R,R*)-**7** (0.02 equiv., see **general procedure C**) as catalyst. The crude residue was purified by MPLC using a solvent gradient (*n*-heptane/EtOAc, from 16 to 100% EtOAc) to give the desired product as colorless oil (1.13 g, 4.8 mmol, 95%); R_f=0.25 (*n*-heptane/EtOAc, 1:2); α_D^{20}=+14.42 (*c*=0.99 in acetone), lit. value for (*S*)-**26** of *ee* 60 %: α_D^{20}=+3.3 (*c*=1.1 in acetone);[2] ^{1}H NMR (400.27 MHz, $CDCl_3$): δ=4.74 (septd, $^3J_{HH}$=6.2 Hz, $^3J_{PH}$=7.3 Hz, 2H, 2 × CH *i*Pr), 3.52 (ddd, $^3J_1$=$^3J_2$=5.7 Hz, $^2J_{PH}$=11.6 Hz, 1H, CH-P), 2.29 (broad s, 1H, OH), 2.12-1.97 (m, 1H, C<u>H</u>-CH(OH)-P), 1.32 (d, $^3J_{HH}$=6.2 Hz, 6H, 2 × CH_3 *i*Pr), 1.32 (d, $^3J_{HH}$=6.2 Hz, 6H, 2 × CH_3 *i*Pr), 1.04 (d, $^3J_{HH}$=6.8 Hz, 3H, CH_3), 1.03 ppm (d, $^3J_{HH}$=6.8 Hz, 3 H, CH_3); ^{31}P NMR (162.03 MHz, $CDCl_3$): δ=23.40 ppm (s); ^{13}C NMR (176.12 MHz, $CDCl_3$): δ=73.7 (d, $^1J_{CP}$=156.7 Hz, CH-P), 71.2 (d, $^2J_{CP}$=7.3 Hz, CH *i*Pr), 71.2 (d, $^2J_{CP}$=7.3 Hz, CH *i*Pr), 30.4 (d, $^2J_{CP}$=1.6 Hz, <u>C</u>H-CH(OH)-P), 24.4 (d, $^3J_{CP}$=3.8 Hz, CH_3 *i*Pr), 24.4 (d, $^3J_{CP}$=3.9 Hz, CH_3 *i*Pr), 24.2 (d, $^3J_{CP}$=4.8 Hz, CH_3 *i*Pr), 24.2 (d, $^3J_{CP}$=4.8 Hz, CH_3 *i*Pr), 20.1 (d, $^3J_{CP}$=9.1 Hz, <u>C</u>H_3-CH-CH), 18.1 ppm (d, $^3J_{CP}$=7.9 Hz, <u>C</u>H_3-CH-CH); IR (ATR): $\tilde{v}$=3328, 3293, 2979, 2934, 1466, 1457, 1386, 1375, 1213, 1179 cm^{-1}; HRMS (ESI): calc. for $[C_{10}H_{23}PO_4+H]^+$: *m/z* 239.1407 [M+H$^+$]; found: *m/z* 239.1409; ^{31}P NMR of (*R*)-**26** with chiral solvating agent (162.03 MHz, $CDCl_3$): δ=95.54 (∫2.68, chiral solvating agent), 23.83 ppm [∫1.00, complex of chiral solvating agent with (*S*)-**26**]; complex of chiral solvating agent with (*R*)-**26** not detected; ee ≥ 99%.

(*R*)-Diisopropyl 1-azido-2-methylpropylphosphonate [(*R*)-73]: Substitution of the hydroxyl group by an azide was accomplished following **general procedure E**, starting from (*S*)-**26** (140 mg, 0.59 mmol), dissolved in dry CH_2Cl_2, and using D*t*BAD (di-*tert*-butyl-azodicarboxylate, 203 mg, 0.88 mmol) as azoester component. Completion of the reaction was observed after 16 h at room temperature. The crude residue was purified by MPLC using a solvent gradient (*n*-heptane/EtOAc, from 12 to 100% EtOAc) to give the desired product (*R*)-**73** as colorless oil (95 mg, 0.36 mmol, 61%); R_f=0.34 (*n*-heptane/EtOAc, 1:1); α_D^{20}=−56.10 (*c*=1.05 in acetone), lit. value for (*R*)-**73**: α_D^{20}=−56 (c=1.1 in actone);[2] ^{1}H NMR (400.27 MHz, $CDCl_3$): δ=4.85-4.71 (m, 2H, 2 × CH *i*Pr), 3.22 (dd, $^3J_{HH}$=5.4 Hz, $^2J_{PH}$=12.8 Hz, 1H, CH-P), 2.14 (m, 1H, C<u>H</u>-CH(N_3)-P), 1.352 (d, $^3J_{HH}$=6.1 Hz, 3H, CH_3 *i*Pr), 1.347 (d, $^3J_{HH}$=6.2 Hz, 6H, 2 × CH_3 *i*Pr), 1.34 (d, $^3J_{HH}$=6.1 Hz, 3H, CH_3 *i*Pr), 1.10 (d, $^3J_{HH}$=6.8 Hz, 3H, C<u>H_3</u>-CH-CH), 1.05 ppm (d, $^3J_{HH}$=6.8 Hz, 3H, <u>CH_3</u>-CH-CH); ^{31}P NMR (162.03 MHz, $CDCl_3$): δ=19.68 ppm (s); ^{13}C NMR (150.93 MHz, $CDCl_3$): δ=71.8 (d, $^2J_{CP}$=7.6 Hz, CH *i*Pr), 71.7 (d, $^2J_{CP}$=7.0 Hz, CH *i*Pr), 64.8 (d, $^1J_{CP}$=156.1 Hz, CH-P), 29.6 (s, <u>C</u>H-CH(N_3)-P), 24.5 (d, $^3J_{CP}$=3.6 Hz, CH_3 *i*Pr), 24.4 (d, $^3J_{CP}$=3.4 Hz, CH_3 *i*Pr), 24.2 (d, $^3J_{CP}$=5.0 Hz, CH_3 *i*Pr), 24.19 (d, $^3J_{CP}$=4.8 Hz, CH_3 *i*Pr), 21.4 (d, $^3J_{CP}$=10.3 Hz, <u>C</u>H_3-CH-CH), 18.9 ppm (d, $^3J_{CP}$=6.7 Hz, <u>C</u>H_3-CH-CH); IR (ATR): $\tilde{v}$=2979, 2936, 2876, 2101, 1468,

1386, 1375, 1255, 1228, 1176 cm^{-1}; elemental analysis calc. (%) for $C_{10}H_{22}N_3PO_3$: C 45.62, H 8.42, N 15.96; found: C 45.43, H 8.71, N 15.16.[3]

(*R*)-1-Amino-2-methylpropylphosphonic acid, (*R*)-phosphavaline [(*R*)-60]: (*R*)-Azidophosphonate **73** (605 mg, 2.3 mmol), can be transformed to (*R*)-phosphavaline (282 mg, 1.8 mmol, 80%) by **general procedure F**, followed by **general procedure G** (reaction time 15 h). The obtained solid could be recrystallised from hot H_2O (60°C) by addition of EtOH to give colorless crystals; R_f=0.20 [*i*PrOH/H_2O/NH_3 (aq., 25%), 6:3:1]; m.p. 281-283°C; α_D^{20}=−1.61 (*c*=0.56 in water); α_D^{20}=+1.42 (*c*=0.67 in NaOH, 1M), lit. for (*R*)-**57**: α_D^{20}=+0.4 (*c*=0.9 in NaOH, 1 M);[2] ^{1}H NMR (400.27 MHz, D_2O): δ=3.15 (dd, $^3J_{HH}$=6.4 Hz, $^2J_{PH}$=14.0 Hz, 1H, CH-P), 2.38 (m, 1H, $(CH_3)_2$-C<u>H</u>), 1.18 (d, $^3J_{HH}$=6.9 Hz, 3H, CH_3), 1.14 ppm (d, $^3J_{HH}$=6.9 Hz, 3H, CH_3); ^{31}P NMR (162.03 MHz, D_2O): δ=12.75 ppm (s); ^{13}C NMR (100.65 MHz, D_2O): δ=54.9 (d, $^1J_{CP}$=141.6 Hz, CH-P), 27.6 (s, $(CH_3)_2$-<u>C</u>H), 19.8 (d, $^3J_{CP}$=7.2 Hz, CH_3), 17.9 ppm (d, $^3J_{CP}$=6.5 Hz, CH_3); IR (ATR): $\tilde{\nu}$=2961, 2668, 2578, 2159, 1608, 1526, 1478, 1229, 1163, 1053 cm^{-1}.

3. Phosphaleucine

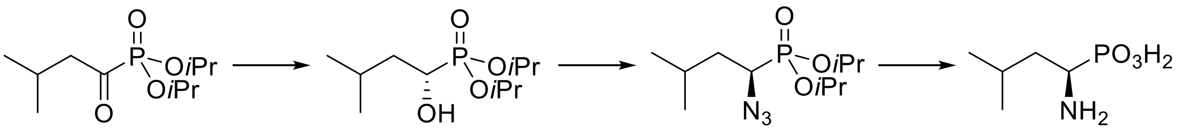

10 (*S*)-**27** (*R*)-**74** (*R*)-**61**

Diisopropyl 3-methylbutanoylphosphonate (10): Ketophosphonate **10** was obtained starting from isovaleryl chloride (603 mg, 5.0 mmol, 0.61 mL) by following **general procedure A** at 0°C within 2.5 h; ^{31}P NMR (162.03 MHz, $CDCl_3$): δ=139.44 (s, $(iPrO)_3P$, ∫0.02), 18.92 and −2.55 (2 × d, J_{PP}=23.3 Hz, unknown impurity, ∫0.04), 4.45 (s, HP(O)(OiPr)2, ∫0.04), 4.38 ppm (s, ∫0.90, **10**).

(*S*)-Diisopropyl 1-hydroxy-3-methylbutylphosphonate [(*S*)-27]: (*S*)-**27** was obtained from crude keto-phosphonate **10** (approx. 4.4 mmol[1]) following **general procedure D** within 18 h using (*R,R*)-**7** (0.02 equiv., see **general procedure C**) as catalyst. The crude residue was purified by MPLC using a solvent gradient (*n*-heptane/EtOAc, from 24 to 100% EtOAc) to give the desired product as colorless oil (1.068 g, 4.22 mmol, 96%); R_f=0.25 (*n*-heptane/EtOAc 1:2); α_D^{20}=−26.95 (*c*=0.95 in acetone); ^{1}H NMR (400.27 MHz, $CDCl_3$): δ=4.73 (m symm, 2H, 2 × CH *i*Pr), 3.85 (ddd, $^3J_{HH}$=3.2 Hz, $^3J_{HH}$=5.2 Hz, $^2J_{HP}$=10.9 Hz, 1H, CH-P), 2.27 (broad s, 1H, OH), 1.95-1.84 (m, 1H, $(CH_3)_2$C<u>H</u>), 1.68-1.58 (m, 1H, $(CH_3)_2$CHC<u>H2</u>), 1.46 (ddd, $^2J_{AB}$= 14.3 Hz, $^3J_{HH}$=3.2 Hz, $^3J_{HH}$=9.5 Hz, $^3J_{HP}$=5.8 Hz; 1H, $(CH_3)_2$CHC<u>H2</u>), 1.33 (d, $^3J_{HH}$=4.6 Hz, 3H, CH_3 *i*Pr),

[3] The rather high difference in the calculated and found values for nitrogen stem from a common loss of N_2 from azide samples under the combustion conditions.

1.32 (d, $^3J_{HH}$=3.8 Hz, 3H, CH_3 *i*Pr), 1.32 (d, $^3J_{HH}$=4.8 Hz, 3H, CH_3 *i*Pr), 1.31 (d, $^3J_{HH}$=3.7 Hz, 3H, CH_3 *i*Pr), 0.95 (d, $^3J_{HH}$=6.7 Hz, 3H, $C\underline{H_3}$-CH-CH_2), 0.90 ppm (d, $^3J_{HH}$=6.8 Hz, 3H, $C\underline{H_3}$-CH-CH_2); ^{31}P NMR (162.03 MHz, $CDCl_3$): δ=23.88 ppm (s); ^{13}C NMR (150.93 MHz, $CDCl_3$): δ=71.3 (d, $^2J_{CP}$=7.2 Hz, CH *i*Pr), 71.2 (d, $^2J_{CP}$=7.3 Hz, CH *i*Pr), 66.7 (d, $^1J_{CP}$=161.1 Hz, CH-P), 40.2 (s, 2H, CH_2), 24.4 (d, $^3J_{CP}$=3.6 Hz, CH_3 *i*Pr), 24.4 (d, $^3J_{CP}$=3.6 Hz, CH_3 *i*Pr), 24.2 (d, $^3J_{CP}$=4.8 Hz, CH_3 *i*Pr), 24.23 (d, $^3J_{CP}$=4.7 Hz, CH_3 *i*Pr), 23.7 (s, $\underline{C}H_3$-CH-CH_2), 21.4 ppm (s, $\underline{C}H_3$-CH-CH_2); IR (ATR): $\tilde{v}$=3302, 2979, 2957, 2870, 1979, 1469, 1385, 1226, 1206, 1107 cm^{-1}; HRMS (ESI): calc. for $[C_{11}H_{25}PO_4+Na]^+$: *m/z* 275.1383 [M+Na^+]; found: *m/z* 275.1385; elemental analysis calc. (%) for $C_{10}H_{23}PO_4$: C 52.37, H 9.99, found: C 52.45, H 9.91; ^{31}P NMR (162.03 MHz, d_8-toluene) of (*S*)-**27** with chiral solvating agent: δ=95.30 (∫2.08, chiral solvating agent), 24.36 ppm [∫1.00, complex of chiral solvating agent with (*S*)-**27**]; complex of chiral solvating agent with (*R*)-**27** not detected]→ *ee* ≥ 99%.

(*R*)-Diisopropyl 1-azido-3-methylbutylphosphonate [(*R*)-74]: Substitution of the hydroxyl group by an azide was accomplished following **general procedure E**, starting from (*S*)-**27** (1.031 g, 4.1 mmol), dissolved in dry CH_2Cl_2, and using DIAD (1.240 g, 6.1 mmol, 1.20 mL) as azoester component. Completion of the reaction was observed after 18 h at room temperature. The crude residue was purified by MPLC using a solvent gradient (CH_2Cl_2/Et_2O, from 1 to 2% Et_2O) to give the desired product as colorless oil (730 mg, 2,6 mmol, 63%); R_f=0.36 (CH_2Cl_2/Et_2O 9:1); α_D^{20}=−53.52 (*c*=0.44 in acetone), lit. for (*R*)-**74**: α_D^{20}=−56.6 (*c*=1.1 in acetone)[2]; ^{1}H NMR (600.25 MHz, $CDCl_3$): δ=4.81-4.72 (m, 2H, 2 × CH *i*Pr), 3.35 (ddd, $^3J_{HH}$=3.1 Hz, $^3J_{HH}$=12.6 Hz $^2J_{HP}$=12.0 Hz, 1H, CH-P), 1.89-1.79 (m, 1H, $(CH_3)_2$-C$\underline{H}$), 1.69-1.51 (m, 2H, CH_2), 1.35 (d, $^3J_{HH}$=6.1 Hz, 6H, 2 × CH_3 *i*Pr), 1.34 (d, $^3J_{HH}$=6.2 Hz, 3H, CH_3 *i*Pr), 1.34 (d, $^3J_{HH}$=6.6 Hz, 3H, CH_3 *i*Pr), 0.97 (d, $^3J_{HH}$=6.7 Hz, 3H, $C\underline{H_3}$-CH-CH_2), 0.91 ppm (d, $^3J_{HH}$=6.6 Hz, 3 H, $C\underline{H_3}$-CH-CH_2); ^{31}P NMR (242.99 MHz, $CDCl_3$): δ=20.80 ppm (s); ^{13}C NMR (150.93 MHz, $CDCl_3$): δ=71.9 (d, $^2J_{CP}$=7.0 Hz, CH *i*Pr), 71.8 (d, $^2J_{CP}$ = 7.0 Hz, CH *i*Pr), 56.1 (d, $^1J_{CP}$ = 157.1 Hz, CH-P), 37.0 (s, CH_2), 25.3 (d, $^3J_{CP}$ = 13.6 Hz, $(CH_3)_2\underline{C}H$), 24.4 (d, $^3J_{CP}$=5.0 Hz, CH_3 *i*Pr), 24.4 (d, $^3J_{CP}$=5.1 Hz, CH_3 *i*Pr), 24.2 (d, $^3J_{CP}$=5.3 Hz, CH_3 *i*Pr), 24.2 (d, $^3J_{CP}$=5.2 Hz, CH_3 *i*Pr), 23.4 (s, $\underline{C}H_3$-CH-CH_2), 21.1 ppm (s, $\underline{C}H_3$-CH-CH_2); IR (ATR): $\tilde{v}$=2979, 2249, 2105, 1978, 1470, 1387, 1261, 1105, 987 cm^{-1}; HRMS (ESI): calc. for $[C_{11}H_{24}PO_3+Na]^+$: *m/z* 300.1447 [M+Na^+]; found: *m/z* 300.1448; elemental analysis calc. (%) for $C_{11}H_{24}N_3PO_3$: C 47.64, H 8.72, N 15.15; found: C 47.91, H 9.16, N 14.07.[3]

(*R*)-1-Amino-3-methylbutylphosphonic acid, (*R*)-phosphaleucine [(*R*)-61]: (*R*)-Azidophosphonate **74** (924 mg, 3.3 mmol), can be transformed to (*R*)-phosphaleucine (501 mg, 2.99 mmol, 90%) by **general procedure F**, followed by **general procedure G** (reaction time 15 h). The obtained white solid could be recrystallized from hot H_2O (60°C) by addition of EtOH to give colorless crystals; R_f 0.33 [*i*PrOH/H_2O/NH_3 (aq., 25%) 6:3:1]; m.p. 282-286°C;

α_D^{20}=−20.65 (*c*=0.77 in water); lit. for (*R*)-**61**: α_D^{20}=−25.0 (*c*=0.7, in 1 M NaOH)[2]; ^{1}H NMR (400.27 MHz, D_2O): δ=3.37 (ddd, $^3J_{HH}$=5.2 Hz, $^3J_{HH}$=9.4 Hz, $^2J_{PH}$ =14.0 Hz, 1H, CH-P), 1.90-1.63 (m, 3H, CH-CH_2-CH_3), 1.03 (d, $^3J_{HH}$=6.3 Hz, 3H, CH_3), 0.99 ppm (d, $^3J_{HH}$=6.1 Hz, 3H, CH_3); ^{31}P NMR (162.03 MHz, D_2O): δ=13.87 ppm (s); ^{13}C NMR (100.65 MHz, D_2O): δ=47.3 (d, $^1J_{CP}$=142.7 Hz, CH-P), 37.3 (d, $^2J_{CP}$=1.9 Hz,CH_2), 24.08 (d, $^2J_{CP}$=9.8 Hz, CH-CH_3), 22.2 (s, CH_3), 20.4 ppm (s, CH_3); IR (ATR): $\tilde{v}$=2963, 2846, 1630, 1612, 1536, 1466, 1386, 1365, 1186, 1165 cm^{-1}; elemental analysis calc. (%) for $C_5H_{14}NPO_3$: C 35.93, H 8.44, N 8.38; O 28.72 found: C 35.83, H 8.49, N 8.37, O 28.68.

4. Phosphaisoleucine

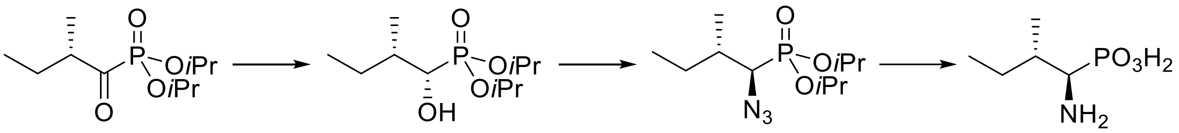

(*S*)-**11** (1*S*,2*S*)-**28** (1*R*,2*S*)-**75** (1*R*,2*S*)-**62**

(*S*)-Diisopropyl 2-methylbutanoylphosphonate (11): (*S*)-**11** can be obtained by **general procedure A** starting from (*S*)-methylbutyric acid (389 mg, 3.2 mmol, 0.40 mL). Acyl chloride formation is complete within 2 h at room temperature. In this case, excess oxalyl chloride cannot be removed by evaporation due to the relatively low boiling point of the intermediate acyl chloride. Thus, $(iPrO)_3P$ was directly added to the reaction mixture. The reaction requires 1.5 h at 0°C to be finished; ^{31}P NMR (162.03 MHz, $CDCl_3$): δ=4.44 (s, ∫0.16, HP(O)(O*i*Pr)$_2$), −4.42 ppm (s, ∫0.84 mol, **11**); ^{1}H NMR (400.27 MHz, $CDCl_3$) of (*S*)-**11** with chiral solvating agent: δ=0.80 ppm [t, $^3J_{HH}$=7.4 Hz, 3H, CH_3 of complex of (*S*)-**11** with chiral solvating agent], [CH_3 of complex of (*R*)-**11** with chiral solvating agent not detected]→ *ee* ≥ 99%.

(1*S*,2*S*)-Diisopropyl 1-hydroxy-2-methylbutylphosphonate [(1*S*,2*S*)-28]: Reduction of the crude keto-phosphonate (*S*)-**11** (approx. 2.6 mmol[1]) following **general procedure D** with (*R*,*R*)-**7** (0.02 equiv., see **general procedure C**) was complete at room temperature after 18 h. The crude residue was purified by MPLC using a solvent gradient (*n*-heptane/EtOAc, 24 to 100% EtOAc) to give the desired product as colorless oil (623 mg, 2.5 mmol, 96%); R_f=0.51 (*n*-heptane/EtOAc, 1:3); α_D^{20}=+7.5 (*c*=0.97 in CH_2Cl_2); ^{1}H NMR (500.32 MHz, $CDCl_3$): δ=4.73 (2 × septd, $^3J_{PH}$=2.5 Hz, $^3J_{HH}$=6.2 Hz, 2H, 2 × CH *i*Pr), 3.73 (symm m, 1H, CH-P), 2.28 (broad s, 1H, OH), 1.88-1.75 (m, 1H, CH_3-CH_2), 1.61-1.50 (m, 1H, CH_3-CH_2), 1.39-1.26 (m, 1H, CH-CH_3), 1.33 (d, $^3J_{HH}$=6.2 Hz, 12H, 4 × CH_3 *i*Pr), 1.02 (d, $^3J_{HH}$ =6.9 Hz, 3H, CH_3-CH), 0.89 ppm (t, $^3J_{HH}$=7.4 Hz, 3H, CH_3-CH_2); ^{31}P NMR (162.03 MHz, $CDCl_3$): δ=23.56 ppm (s); de ≥ 99%; ^{13}C NMR (125.81 MHz, $CDCl_3$): δ=71.7 (d, $^1J_{CP}$=157.4 Hz, CH-P), 71.2 (d, $^2J_{CP}$=7.3 Hz, CH *i*Pr), 71.1 (d, $^2J_{CP}$=7.5 Hz, CH *i*Pr), 36.5 (d, $^2J_{CP}$=2.2 Hz, 1H, CH-CH_3), 26.9 (d, $^3J_{CP}$=12.2 Hz, CH_2), 24.4 (d, $^3J_{CP}$=3.4 Hz, CH_3 *i*Pr), 24.4 (d, $^3J_{CP}$=3.5 Hz, CH_3 *i*Pr), 24.2 (d, $^3J_{CP}$=4.8 Hz, 2 × CH_3

*i*Pr), 14.3 (d, $^3J_{CP}$=4.9 Hz, $\underline{C}H_3$-CH), 11.9 ppm (s, $\underline{C}H_3$-CH_2); IR (ATR): $\tilde{\nu}$=3315, 2978, 1358, 1209, 1105, 979, 886, 626 cm^{-1}; HRMS (ESI): calc. for $[C_{11}H_{25}PO_4+Na]^+$: *m/z* 275.1383 $[M+Na^+]$; found: *m/z* 275.1392; ^1H (400.27 MHz, *d_8*-toluene) NMR of (1*S,2S*)-**28** with chiral solvating agent: δ=4.17 [∫1.00, complex of chiral solvating agent with (1*S,2S*)-**28**], 3.87 ppm [∫0.017, complex of chiral solvating agent with presumably (1*S,2R*)-**28**]→ *de* ≥ 97%, ee ≥ 99% for each diastereomer.

(1*R*,2*S*)-Diisopropyl 1-azido-2-methylbutylphosphonate [(1*R*,2*S*)-75]: Substitution of the hydroxyl group by an azide was accomplished following **general procedure E**, starting from (1*S*,2*S*)-**28** (453 mg, 1.80 mmol) dissolved in dry CH_2Cl_2 and using DEAD as azoester component. Completion of the reaction was observed after 18 h at room temperature. The crude residue was purified by MPLC using a solvent gradient (CH_2Cl_2/Et_2O, 1 to 5% Et_2O) to give the desired product (1*R*,2*S*)-**75** as colorless oil (454 mg, 1.64 mmol, 91%). R_f=0.50 (CH_2Cl_2/Et_2O, 9:1); α_D^{20}=−45.1 (*c*=0.45 in acetone), ^1H NMR (600.25 MHz, $CDCl_3$): δ=4.84-4.75 (m, 2H, 2 × CH *i*Pr), 3.27 (dd, $^3J_{HH}$=6.6 Hz, $^2J_{HP}$=12.9 Hz, 1H, CH-P), 1.88 (m, 1H, C$\underline{H}$-CH-P), 1.77 (dqd, $^2J_{AB}$=15.1 Hz, $^3J_{AH}$=7.5 Hz $^3J_{AH}$=3.4 Hz, 1H, CH_2), 1.37 (d, $^3J_{HH}$=6.2 Hz, 3H, CH_3 *i*Pr), 1.37 (d, $^3J_{HH}$=6.2 Hz, 6H, 2 × CH_3 *i*Pr), 1.36 (d, $^3J_{HH}$=6.3 Hz, 3H, CH_3 *i*Pr), 1.32-1.25 (m, 1H, CH_2), 1.10 (d, $^3J_{HH}$=6.7 Hz, 3H, C$\underline{H}_3$-CH-CH-P), 0.91 ppm (t, $^3J_{AH}$=7.5 Hz, 3H, C$\underline{H}_3$-CH_2); ^{31}P NMR (161.98 MHz, $CDCl_3$): δ=19.99 ppm (s); de ≥ 99%; ^{13}C NMR (176.12MHz, $CDCl_3$): δ=71.7 (d, $^2J_{CP}$=7.6 Hz, CH *i*Pr), 71.6 (d, $^2J_{CP}$=7.0 Hz, CH *i*Pr), 64.0 (d, $^1J_{CP}$=155.7 Hz, CH-P), 35.8 (s, $\underline{C}$H-CH-P), 25.5 (d, $^3J_{CP}$=7.6 Hz, $(CH_3)_2\underline{C}H$), 24.4 (d, $^3J_{CP}$=4.0 Hz, CH_3 *i*Pr), 24.4 (d, $^3J_{CP}$=3.8 Hz, CH_3 *i*Pr), 24.2 (d, $^3J_{CP}$=4.9Hz, CH_3 *i*Pr), 24.1 (d, $^3J_{CP}$=4.7 Hz, CH_3 *i*Pr), 17.00 (d, $^3J_{CP}$=8.1 Hz $\underline{C}H_3$-CH), 11.2 ppm (s, $\underline{C}H_3$-CH_2); IR (ATR): $\tilde{\nu}$=2975, 2099, 1460, 1381, 1249, 1105, 981, 623 cm^{-1}; HRMS (ESI): calc. for $[C_{11}H_{24}N_3PO_3+Na]^+$: *m/z* 300.1447 $[M+Na^+]$; found: *m/z* 300.1446; ^{31}P NMR (161.98 MHz, *d_8*-toluene) of (1*R*,2*S*)-**75** with chiral solvating agent: δ=96.05 [∫0.83, chiral solvating agent], 19.93 [∫0.17, complex of chiral solvating agent with (1*R*,2*S*)-**75**], 19.83 ppm [∫0.002, complex of chiral solvating agent with presumably (1*S*,2*R*)-**75**] →*ee* ≥ 97%.

(1*R*,2*S*)-1-amino-2-methylbutylphosphonic acid, (1*R*,2*S*)-phosphaisoleucine [(1*R*,2*S*)-62]: (1*R*,2*S*)-Azidophosphonate **75** (330 mg, 1.19 mmol), can be transformed to (1*R*,2*S*)-phosphaisoleucine (192 mg, 1.15 mmol, 97%) by **general procedure F**, followed by **general procedure G** (reaction time 15 h, reaction temperature should not exceed 100°C in this case). The obtained solid was purified by ion exchange chromatography using aqueous HCO_2H (1% v/v) as eluent. R_f=0.33 [*i*PrOH/H_2O/NH_3 (aq., 25%), 6:3:1]; α_D^{20}=−8.7 (*c*=1.08 in water); ^1H NMR (700.40 MHz, D_2O): δ=3.20 (dd, $^3J_{HH}$=6.0 Hz, $^2J_{HP}$=14.3 Hz, 1H, CH-P), 1.98 (symm m, 1H, C$\underline{H}$-CH_3), 1.71 (dqd, $^2J_{AB}$=14.9 Hz, $^3J_{AH}$=7.5 Hz, $^3J_{AH}$ =3.9 Hz, 1H CH_2) 1.31-1.22 (m, 1H, CH_2), 1.12 (d, $^3J_{HH}$=6.9 Hz, 3H, C$\underline{H}_3$-CH), 0.94 ppm (t, $^3J_{AH}$ =7.5 Hz, 3H, C$\underline{H}_3$-CH_2); ^{31}P NMR

(161.98 MHz, D_2O): δ=12.76 ppm (s); de ≥ 99%; ^{13}C NMR (176.12 MHz, D_2O): δ=53.9 (d, $^{1}J_{CP}$=141.4 Hz, CH-P), 34.3 (s, <u>C</u>H-CH-P), 24.7 (d, $^{3}J_{CP}$=5.6 Hz, <u>C</u>H_2), 15.6 (d, $^{3}J_{CP}$=6.4 Hz, <u>C</u>H_3-CH), 10.5 ppm (s, <u>C</u>H_3-CH_2); IR (ATR): $\tilde{\nu}$=2881, 1610, 1525, 1176, 1058, 926, 815, 730 cm^{-1}; HRMS (ESI): calc. for the dimer $[(C_5H_{14}NPO_3)_2+H]^+$: *m/z* 335.1495 $[2M+H^+]$; found: *m/z* 335.1499; elemental analysis calc. (%) for $C_5H_{14}NPO_3$: C 35.93, H 8.44, N 8.38, O 28.72, P 18.53; found: C 35.60, H 8.56, N 8.09, O 28.96, P 18.35.

5. Phosphalysine

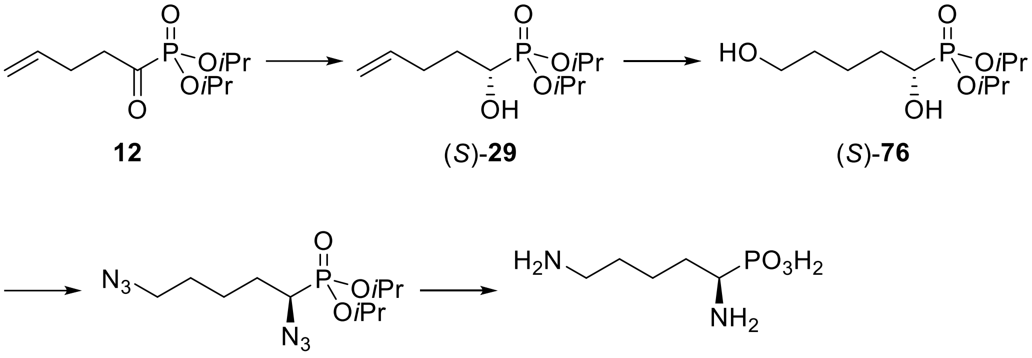

12 (*S*)-**29** (*S*)-**76**

(*R*)-**77** (*R*)-**63**

Diisopropyl pent-4-enoylphosphonate (12): Ketophosphonate **12** was obtained starting from 4-pentenoic acid (1.0 g, 10 mmol, 1.0 mL) by following **general procedure A** at 25°C within 3 h; ^{1}H NMR (400.27 MHz, $CDCl_3$): δ=5.88-5.71 (m, 1.2H, CH= from keto- and enol form + CH-C-P enol-form), 5.29 (s, 0.2H, enol-form, OH), 5.15-4.96 (m, 2H, =CH_2), 4.87-4.68 (m, 2H, CH *i*Pr), 3.01-2.89 (m, 2H, CH_2 enol-form + CH_2-C(O) keto-form), 2.40-2.32 (m, 1.6H, =CH-C<u>H</u>$_2$ keto-form); ^{31}P NMR (162.03 MHz, $CDCl_3$): δ=18.16 and −2.65 (2 × d, J_{PP}=26.9 Hz, unknown impurity, ∫0.13), 6.89 and −3.33 (2 × d, J_{PP}=31.6, unknown impurity, ∫0.06), 4.44 (s, enol-form of **12**, ∫0.14), −4.41 ppm (s, keto-form of **12**, ∫0.67).

(*S*)-Diisopropyl 1-hydroxypent-4-en-1-yl-phosphonate [(*S*)-29]: (*S*)-**29** was obtained from crude keto-phosphonate **12** (approx. 4.34 mmol[1]) following **general procedure D** within 16 h using (*R,R*)-**7** (0.01 equiv., see **general procedure C**) as catalyst. The crude residue was purified by flash chromatography to give the desired product as colorless oil (982 mg, 3.92 mmol, 90%). R_f=0.17 (*n*-heptane/EtOAc 1:1); α_D^{20}=+5.9 (*c*=0.53 in CH_2Cl_2); ^{1}H NMR (600.25 MHz, $CDCl_3$): δ=5.87-5.77 (m, 1H, -CH=), 5.10-4.99 (m, 2H, =CH_2), 4.79-4.72 (m, 2H, 2 × CH *i*Pr), 3.80 (ddd, $^{1}J_{HP}$=10.2 Hz, $^{3}J_{HH}$=4.6 Hz, $^{3}J_{HH}$=3.6 Hz, 1H, CH-P), 2.39-2.31 (m, 1H, C<u>H</u>$_2$-CH=CH_2), 2.24-2.15 (m, 1H, C<u>H</u>$_2$-CH=CH_2), 1.97 (broad s, 1H, OH), 1.89-1.81 (m, 1H, C<u>H</u>$_2$-CH-P), 1.80-1.71 (m, 1H, C<u>H</u>$_2$-CH-P), 1.35 (d, $^{3}J_{HH}$=4.2 Hz, 3H, CH_3), 1.34 (d, $^{3}J_{HH}$=4.6 Hz, 3H, CH_3), 1.34 (d, $^{3}J_{HH}$=4.2 Hz, 3H, CH_3), 1.33 ppm (d, $^{3}J_{HH}$=4.6 Hz, 3H, CH_3); ^{31}P NMR (242.99 MHz, $CDCl_3$): δ=23.28 ppm (s); ^{13}C NMR (150.93 MHz, $CDCl_3$): δ=137.7 (s, CH=), 115.7 (s,

CH_2=), 71.4 (d, $^2J_{CP}$=7.1 Hz, CH *i*Pr), 71.2 (d, $^2J_{CP}$=7.3 Hz, CH *i*Pr), 67.7 (d, $^1J_{CP}$=161.6 Hz, CH-P), 30.6 (s, $\underline{C}H_2$-CH=), 29.9 (d, $^2J_{CP}$=13.7 Hz, $\underline{C}H_2$-CH-P), 24.3 (d, $^3J_{CP}$=3.7 Hz, CH_3), 24.3 (d, $^3J_{CP}$=3.7 Hz, CH_3), 24.2 (d, $^3J_{CP}$=4.7 Hz, CH_3), 24.2 ppm (d, $^3J_{CP}$=4.5 Hz, CH_3); IR (ATR): $\tilde{v}$=3307, 2979, 2936, 1641, 1385, 1219, 1105, 979 cm^{-1}; HRMS (ESI): calc. for $[C_{11}H_{23}O_4P+Na]^+$: *m/z* 273.1227 $[M+Na]^+$; found: *m/z* 273.1225. ^{31}P NMR (242.99 MHz, $CDCl_3$) of (*R*)-**29** with chiral solvating agent: δ=95.28 [∫0.37, chiral solvating agent], 23.38 [∫0.004, complex of chiral solvating agent with (*R*)-**29**], 23.24 ppm [∫0.62, complex of chiral solvating agent with (*S*)-**29**]; *ee* ≥ 98.7%.

(*S*)-Diisopropyl-(1,5-dihydroxypentyl)phosphonate [(*S*)-76]: Hydroxyphosphonate (*S*)-**29** (0.400 g, 1.6 mmol, 1equiv.) was dissolved in dry THF (5 mL) under an argon atmosphere, BH_3*THF (3.2 ml, 3.2 mmol, 2 equiv.) was added dropwise at 0°C and the mixture was stirred for 3h. The reaction was quenched with MeOH (5 mL, added slowly to prevent foam-formation) and stirring was continued for 30 minutes, followed by concentration to 1/3 of its volume to remove MeOH. Then THF (5 mL), H_2O_2 [3 mL, 35 % (v/v)] and $NaHCO_3$ (3 mL) were added and stirred for another 2h. H_2O (7 mL) was added, after 30 minutes of stirring the organic solvent was removed under reduced pressure and the crude product was extracted from the aqueous solution with EtOAc (3 × 20 mL). The organic phases were combined, concentrated *in vacuo* and purified by flash chromatography with a solvent gradient (EtOAc/MeOH, 0 to 5% MeOH) to give (S)-diisopropyl-(1,5-dihydroxypentyl)phosphonate (*S*)-**76** (290 mg, 1.08 mmol, 73%) as a colorless oil; R_f=0.29 (EtOAc/MeOH 19:1); α_D^{20}=+14.9 (*c*=1.04 in CH_2Cl_2); ^{1}H NMR (600.25 MHz, $CDCl_3$) δ=4.79-4.71 (m, 2H, 2 × CH *i*Pr), 3.81-3.76 (m, 1H, CH-P), 3.67 (t, $^3J_{HH}$=6.4 Hz, 2H, HO-C$\underline{H_2}$-CH_2), 2.61 (broad s, OH), 1.83-1.76 (m, 1H, CH_2), 1.75-1.68 (m, 2H, 1 × CH_2 and 1 × CH_2), 1.67-1.55 (m, 2H, CH_2), 1.54-1.47 (m, 1H, CH_2), 1.36-1.32 ppm (m, 12H, 4 × CH_3 iPr); ^{31}P NMR (162.03 MHz, $CDCl_3$) δ=23.4 ppm (s); ^{13}C NMR (150.93 MHz, $CDCl_3$) δ=71.2 (d, $^2J_{CP}$=7.3 Hz, CH *i*Pr), 71.1 (d, $^2J_{CP}$=7.48 Hz, CH *i*Pr), 68.2 (d, $^1J_{CP}$=161.3 Hz, CH-P), 62.5 (s, HO-CH_2), 32.1 (s, CH_2), 30.8 (s, CH_2), 24.1 (s, CH_3 *i*Pr), 24.1 (s, CH_3 *i*Pr), 24.0 (s, CH_3 *i*Pr), 24.0 (s, CH_3 *i*Pr), 21.9 ppm (d, $^2J_{CP}$=13.5 Hz, $\underline{C}H_2$-CH-P); IR (ATR): $\tilde{v}$=3462, 2980, 2937, 1454, 1385, 1375, 1217, 1104, 983 cm^{-1}; HRMS (ESI): calc. for $[C_{11}H_{25}O_5P+Na]^+$: *m/z* 291.1332 $[M+Na]^+$; found: *m/z* 291.1333; ^{31}P NMR (242.99 MHz, $CDCl_3$) with chiral solvating agent: δ=96.74 [∫2.49, chiral solvating agent], 23.10 [∫1.00, complex of (*S*)-**76** with chiral solvating agent], complex of chiral solvating agent with (*R*)-**76** not detected; *ee* ≥ 99.9 %.

(*R*)-Diisopropyl (1,5-diazidopentyl)phosphonate [(*R*)-77]: Dihydroxyphosphonate (*S*)-**76** (0.270 g, 1 mmol) was converted to (*R*)-diisopropyl-(1,5-diazidopentyl)phosphonate (*R*)-**77** (0.110 g, 0.34 mmol, 34%) following **general procedure E** but with adapted amounts of PPh_3 (0.68 g, 2.6 mmol, 2.6 equiv.), HN_3 (3.6 mmol, 2 mL, 3.6 equiv.) and DIAD (2.6 mmol, 0.51 mL,

2.6 equiv.) in THF (3 mL) within 3 h. The crude product was purified by flash chromatography using a solvent gradient (n-heptane/EtOAc, 16 to 100% EtOAc) to give a faint yellow oil; R_f=0.34 (n-heptane/EtOAc 1:1); α_D^{20}=−43.9 (*c*=1.10 in CH_2Cl_2); ^{1}H NMR (600.25 MHz, $CDCl_3$) δ=4.83-4.75 (m, 2H, 2 × CH *i*Pr), 3.35-3.28 (m, 3H, CH-P and N_3-CH_2), 1.92-1.82 (m, 1H, CH_2), 1.73-1.56 (m, 4H, from three different CH_2), 1.55-1.45 (m, 1H, CH_2), 1.39-1.34 ppm (m, 12H, 3 × CH_3 *i*Pr); ^{31}P NMR (162.03 MHz, $CDCl_3$) δ=19.86 ppm (s); ^{13}C NMR (150.93 MHz, $CDCl_3$) δ=71.9 (d, $^2J_{CP}$=7.5 Hz, C̲H iPr), 71.8 (d, $^2J_{CP}$=7.2 Hz, C̲H iPr), 57.7 (d, $^1J_{CP}$=156.8 Hz, N_3-CH-P), 51.1 (s, N_3-CH_2), 28.3 (s, CH_2), 28.2 (s, CH_2), 24.16 (s, CH_3 *i*Pr), 24.1 (s, CH_3 *i*Pr), 24.1 (s, CH_2), 24.0 (s, CH_3 *i*Pr), 24.0 (s, CH_3 *i*Pr); IR (ATR): $\tilde{v}$=2984, 2934, 2098, 1738, 1375, 1252, 1105, 988 cm^{-1}; HRMS (ESI): calc. for $[C_{11}H_{23}N_6O_3P+Na]^+$: *m/z* 341.1462 $[M+Na]^+$; found: *m/z* 341.1466; ^{31}P NMR (242.99 MHz, $CDCl_3$) with chiral solvating agent: δ=97.49 [∫1.65, chiral solvating agent], 19.91 (∫1.00, complex of (*R*)-**77** with chiral solvating agent), complex of chiral solvating agent with (*S*)-**77** not detected; *ee* ≥ 99.9 %.

(*R*)-(1,5-Diaminopentyl)phosphonic acid, (*R*)-phosphalysine [(*R*)-63]: The azide (*R*)-**77** (90 mg, 0.28 mmol) can be transformed to (*R*)-phosphalysine [(*R*)-**63**, 25 mg, 0.14 mmol, 50%] in two steps: **general procedure F** yields (*R*)-diisopropyl-,5-diaminopentylphosphonate; ^{1}H NMR (600.25 MHz, $CDCl_3$) δ=4.79-4.60 (m, 2 × CH *i*Pr), 2.87-2.77 (m, 1H, N-CH-P), 2.73-2.64 (m, 2H, N-CH_2), 1.85-1.55 (m, 2H, C$\underline{H_2}$-CH), 1.52-1.37 (m, 4H, -CH_2-CH_2-), 1.35-1.26 ppm (m, 12H, 4 × CH_3 *i*Pr); ^{31}P NMR (162.03 MHz, $CDCl_3$) δ=27.29 ppm (s); HRMS (ESI): calc. for $[C_{11}H_{27}N_2O_3P+Na]^+$: *m/z 2*89.1652 $[M+Na]^+$; found: *m/z* 289.1653. Then, **general procedure G** (reaction time 12 h) produces (*R*)-**63** as a glassy solid. An aqueous solution of NH_3 [2% (v/v)] was used to elute the product in this case; α_D^{20}=−13.25 (*c*=0.40 in D_2O), lit. value for (*R*)-**63** × 0.17 HBr: α_D^{20} –9.7 (*c*=0.66, H_2O);[3] ^{1}H NMR (600.25 MHz, $CDCl_3$) δ=3.08-3.01 (m, 3H, H_2N-CH_2 and H_2N-CH-P), 2.00-1.90 (m, 1H, H_2N-CH_2-C$\underline{H_2}$), 1.79-1.67 (m, 3H, 1H from H_2N-CH_2-C$\underline{H_2}$, 2H from H_2N-CH_2-CH_2-C$\underline{H_2}$), 1.65-1.50 ppm (m, 2H, P-CH-C$\underline{H_2}$); ^{31}P NMR (162.03 MHz, $CDCl_3$) δ=11.51 ppm (s); ^{13}C NMR (150.93 MHz, $CDCl_3$) δ=50.7-49.9 (d, $^1J_{CP}$=133.0 Hz, H_2N-C̲H-P), 39.0 (s, H_2N-CH_2), 28.6 (d, $^3J_{CP}$=1.4 Hz, H_2N-CH_2-CH_2-C̲H_2), 26.3 (s, H_2N-CH_2-C̲H_2), 22.8 ppm (d, $^2J_{CP}$=8.1 Hz, P-CH-C̲H_2); IR (ATR): $\tilde{v}$=2931, 2861, 2554, 2349, 2138, 1611, 1562, 1469, 1042, 972, 738 cm^{-1}; HRMS (ESI): calc. for $[C_5H_{15}N_2O_3P+Na]^+$: *m/z* 205.0713 $[M+Na]^+$; found: *m/z* 205.0705.

6. Phosphaarginine (precursor)

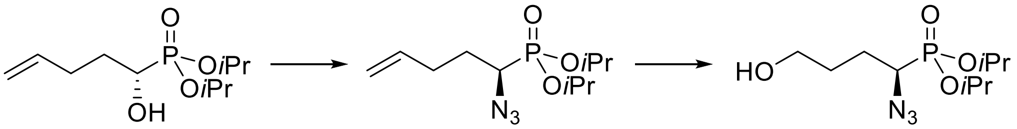

(*S*)-**29** (*R*)-**78** (*R*)-**79**

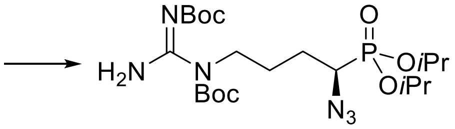

(*R*)-**80**

(*R*)-Diisopropyl 1-azidopent-4-en-1-yl-phosphonate [(*R*)-78]: (*S*)-**29** (931 mg, 3.72 mmol) was converted to (*R*)-**78** (658 mg, 2.39 mmol, 64%) following **general procedure E**, using DIAD as azoester. The product is prone to cyclize during chromatographic separation. Most impurities were removed by fast MPLC (CH_2Cl_2: Et_2O, 95:5). This gave the product as colorless oil in 78 mol% purity, which was sufficient for the subsequent step; R_f=0.52 (CH_2Cl_2/Et_2O, 9:1); ^{1}H NMR (*tentatively assigned signals,* 400.24 MHz, $CDCl_3$): δ=5.85-5.70 (m, 1H, CH=), 5.14-5.00 (m, 2H, CH_2=), 4.88-4.70 (m, 2H, 2 × CH *i*Pr), 3.40-3.33 (td, $^2J_{HP}$=11.7 Hz, $^3J_{HH}$=3.7 Hz, 1H, CH-P), 2.40-2.30 (m, 1H, C$\underline{H}_2$-CH=CH_2), 2.25-2.12 (m, 1H, C$\underline{H}_2$-CH=CH_2), 1.99-1.86 (m, 1H, C$\underline{H}_2$-CH-P), 1.81-1.63 (m, 1H, C$\underline{H}_2$-CH-P), 1.36 ppm (broad d, $^3J_{HH}$=6.0 Hz, 12H, 4 × CH_3); ^{31}P NMR: (162.02 MHz, $CDCl_3$): δ=22.53 (s, ∫0.07, unknown impurity), 20.28 (s, ∫0.78, **78**), 19.71 (s, ∫0.075, unknown impurity), 19.67 ppm (s, ∫0.075, unknown impurity). HRMS (ESI): calc. for $[C_{11}H_{22}N_3O_3P+Na]^+$: *m/z* 298.1291 $[M+Na]^+$; found: *m/z* 298.1289.

(*R*)-Diisopropyl 1-azido-4-hydroxybutylphosphonate [(*R*)-79]: (*R*)-**78** (482 mg, 1.75 mmol) was dissolved in a mixture of MeOH and CH_2Cl_2 [16 mL, 1:1 (v/v)] and the resulting solution was cooled to −78°C. O_3 (flow: 100 N/h, 70% O_3) was bubbled through the reaction mixture until the solution turned pale blue (5 min). Then, O_2 was bubbled through the mixture until the blue colour disappeared again. Dimethylsulfide (120 mg, 0.14 mL, 1.93 mmol, 1.1 equiv.) was added and the cooling bath was removed. The reaction mixture was allowed to come to 20°C for 20 minutes. Subsequently, the solution was again cooled to −35°C and $NaBH_4$ (132 mg, 3.50 mmol, 2 equiv.), dissolved in EtOH (2 mL), was added. The reaction mixture was allowed to warm up to 20°C in the cooling bath over a period of 4 h, before the solvent was evaporated *in vacuo* at ambient temperature. The residue was re-dissolved in EtOAc (15 mL), washed with a saturated aqueous $NaHCO_3$ solution (3 × 15 mL) and the combined aqueous phases were re-extracted with EtOAc (2 × 10 mL). The combined organic layers were dried (Na_2SO_4), filtered and evaporated. The crude residue was purified by MPLC (*n*-heptane/EtOAc, 18 to 100% EtOAc) to give (*R*)-**79** (383 mg, 1.37 mmol, 78%) as colorless oil; R_f=0.09 (*n*-

heptane:EtOAc, 1:1); α_D^{20}=−46.9 (*c*=0.55 in acetone); ^{1}H NMR (700.40 MHz, $CDCl_3$): δ=4.84-4.74 (m, 2H, 2 × CH *i*Pr), 3.70 (dd, $^3J_{HH}$=6.2 Hz, $^3J_{HH}$=5.5 Hz, 2H, -$C\underline{H}_2$-OH), 3.43-3.39 (ddd, $^3J_{HH}$=3.5 Hz, $^3J_{HH}$ and $^2J_{HP}$=10.4 Hz, 11.8 Hz, 1H, CH-P), 2.05-1.93 (m, 1H, $C\underline{H}_2$-CH-P), 1.93-1.80 (m, 1H, $C\underline{H}_2$-CH-P), 1.80-1.66 (m, 1H, $C\underline{H}_2$-CH_2-OH), 1.63 (broad s, 1H, OH), 1.37 (d, $^3J_{HH}$=6.2 Hz, 9H, 3 × CH_3), 1.36 ppm (d, $^3J_{HH}$=6.0 Hz, 3H, CH_3); ^{31}P NMR: (162.02 MHz, $CDCl_3$): δ=0.13 ppm (s); ^{13}C NMR (176.12 MHz, $CDCl_3$): δ=72.0 (d, $^2J_{CP}$=7.4 Hz, CH *i*Pr), 72.0 (d, $^2J_{CP}$=7.2 Hz, CH *i*Pr), 62.2 (s, CH_2-OH), 57.8 (d, $^1J_{CP}$=156.7 Hz, CH-P), 29.8 (d, $^2J_{CP}$=12.7 Hz, $\underline{C}H_2$-CH-P), 25.5 (s, $\underline{C}H_2$-CH_2-OH), 24.3 (d, $^3J_{CP}$=3.7 Hz, 2 × CH_3), 24.2 ppm (d, $^3J_{CP}$=4.7 Hz, 2 × CH_3); IR (ATR): $\tilde{\nu}$=3406, 2941, 2101, 1451, 1379, 1234, 985 cm^{-1}; HRMS (ESI): calc. for $[C_{10}H_{22}N_3O_4P+Na]^+$: *m/z* 302.1241 $[M+Na]^+$; found: *m/z* 302.1241.

(*R*)-Diisopropyl [1-azido-4-bis(*tert*-butoxycarbonyl)guanidino]butylphosphonate [(*R*)-80]:(*R*)-**79** (300 mg, 1.08 mmol,1 equiv.) was dried by co-evaporation of residual water with toluene (1 mL) and then dissolved in toluene (4 mL) under argon atmosphere. The resulting solution was added to a mixture of DIAD (365 mg, 0.36 mL, 1.80 mmol, 1.66 equiv.) and Ph_3P (473 mg, 1.80 mmol, 1.66 equiv.) in dry toluene (5 mL) under argon atmosphere at 0°C. After stirring for 5 minutes, *N*,*N´*-bis(*tert*-butoxycarbonyl)guanidine[4] (0.341 mg, 1.32 mmol, 1.22 equiv.) in dry toluene (4 mL) was added dropwise. The reaction mixture was allowed to come to 20°C in the cooling bath and stirring was continued for 4 h. The solvent was removed *in vacuo* at ambient temperature and the residue was purified by flash column chromatography (*n*-heptane:EtOAc, 2:1) to yield (*R*)-**80** (499 mg, 0.96 mmol, 89%) as colorless oil; R_f=0.54 (*n*-heptane:EtOAc, 1:1); α_D^{20}=−44.0 (*c*=0.5 in acetone); ^{1}H NMR (700.40 MHz, $CDCl_3$): δ=9.39 (broad s, 1H, NH), 9.20 (broad s, 1H, NH), 4.85-4.71 (m, 2H, 2 × CH *i*Pr), 3.95 (symm m, 2H, $C\underline{H}_2$-NBoc), 3.48 (ddd, $^2J_{HP}$=$^3J_{HH}$=11.5 Hz, $^3J_{HH}$=3.0 Hz, 1H, CH-P), 1.97-1.78 (m, 2H, $C\underline{H}_2$-CH_2-NBoc), 1.77-1.60 (m, 2H, $C\underline{H}_2$-CH-P), 1.53 (s, 9H, 3 × CH_3 Boc), 1.49 (s, 9H, 3 × CH_3 Boc), 1.36 (d, $^3J_{HH}$=6.4 Hz, 9H, 3 × CH_3 *i*Pr), 1.35 ppm (d, $^3J_{HH}$=7.2 Hz, 3H, CH_3 *i*Pr); ^{31}P NMR: (162.02 MHz, $CDCl_3$): δ=20.17 ppm (s); ^{13}C NMR (176.12 MHz, $CDCl_3$): δ=164.0 (s, C-NH_2), 160.7 (s, C=O), 155.1 (s, C=O), 84.1 (s, $\underline{C}(CH_3)_3$), 78.9 (s, $\underline{C}(CH_3)_3$), 71.9 (d, $^2J_{CP}$=7.3 Hz, CH *i*Pr), 71.8 (d, $^2J_{CP}$=7.1 Hz, CH *i*Pr), 57.5 (d, $^1J_{CP}$=157.1Hz, CH-P), 43.8 (s, CH_2-NBoc), 28.4 (s, 3C, 3 × CH_3 Boc), 28.2 (s, 3C, 3 × CH_3 Boc), 26.1 (d, $^2J_{CP}$=13.6 Hz, $\underline{C}H_2$-CH-P), 25.93 (s, $\underline{C}H_2$-CH_2-NBoc), 24.34 (d, $^3J_{CP}$=3.4 Hz, CH_3 *i*Pr), 24.33 (d, $^3J_{CP}$=3.5 Hz, CH_3 *i*Pr), 24.17 ppm (d, *J*=4.8 Hz, 2 × CH_3 *i*Pr); IR (ATR): $\tilde{\nu}$=3398, 2978, 2102, 1711, 1610, 1509, 1373, 1246, 1141, 983 cm^{-1}; HRMS (ESI): calc. for $[C_{21}H_{41}N_6O_7P+H]^+$: *m*/*z* 521.2848 $[M+H]^+$; found: *m*/*z* 521.2849; ^{31}P NMR (161.98 MHz, *d$_8$*-toluene) with chiral solvating agent: δ=95.82 [∫0.75, chiral solvating agent], 20.23 [∫0.25, complex of chiral solvating agent with (*R*)-**80**], 20.27 ppm [∫0.002 complex of chiral solvating agent with (*S*)-**80**]; *ee* ≥ 98.4%.

7. Phosphaphenylalanine

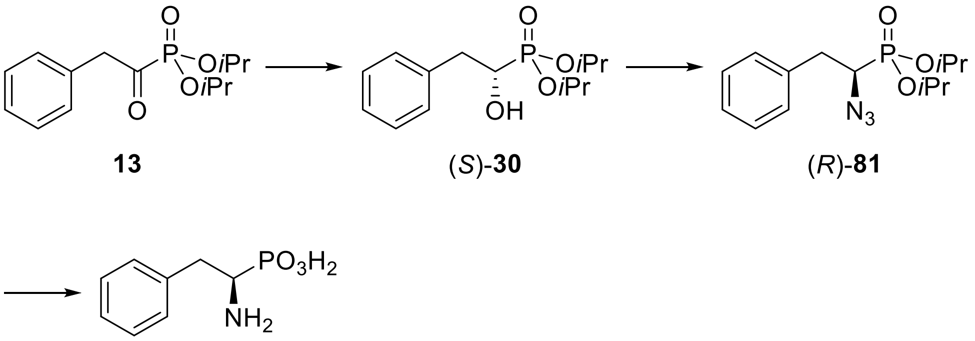

13 (*S*)-**30** (*R*)-**81** (*R*)-**64**

Diisopropyl (2-phenylacetyl)phosphonate (13): Ketophosphonate **13** (1.198 g, 2.79 mmol, 83%) was obtained as yellowish oil starting from 2-phenylacetic acid (1.035 g, 3.34 mmol) by following **general procedure A** within 16 h at 35°C. The crude product was used without further purification; ^{31}P NMR (162.03 MHz, $CDCl_3$): δ=11.00 (s, enol-form of **13**, ∫0.43), 4.40 (s, HP(O)(O*i*Pr)$_2$, ∫0.09), −3.44 (s, unknown impurity, ∫0.07), −4.18 (s, ∫0.48, keto-form of **13**).

(*S*)-Diisopropyl (1-hydroxy-2-phenylethyl)phosphonate [(*S*)-30]: (*S*)-**30** was obtained from crude keto-phosphonate **13** (approx. 1.9 mmol[1]) following **general procedure D** within 18 h using (*R*,*R*)-**7** (0.02 equiv. see **general procedure C**) as catalyst. Purification was performed by column chromatography (*n*-heptane/EtOAc, 1:1) to furnish the desired product as a yellowish liquid (0.441 g, 1.54 mmol, 78%); R_f=0.56 (*n*-heptane/EtOAc, 1:1); α_D^{20}=+31.53 (*c*=1.5 in acetone); ^{1}H NMR ($CDCl_3$, 400.27 MHz): δ=7.33-7.19 (m, 5H, CH arom), 4.84-4.69 (m, 2H, 2 × CH *i*Pr), 4.01 (ddd, $^3J_{AH}$=3.2 Hz, $^2J_{PH}$=5.3 Hz, $^3J_{BH}$=10.3 Hz, 1H, CH-P), 3.11 (ddd, $^2J_{AB}$=14.3 Hz, $^3J_{AP}$=6.5 Hz, $^3J_{AH}$=3.2 Hz, 1H, CH_2), 2.88 (ddd, $^2J_{AB}$=14.3 Hz, $^3J_{BH}$=$^3J_{BP}$=10.3 Hz, 2H, CH_2), 2.17 (broad s, 1H, OH), 1.35 (d, $^3J_{HH}$=6.2 Hz, 6H, 2 × CH_3), 1.33 (d, $^3J_{HH}$=6.4 Hz, 3H, CH_3), 1.30 ppm (d, $^3J_{HH}$=6.4 Hz, 3H, CH_3);); ^{31}P NMR ($CDCl_3$, 242.92 MHz): δ=22.08 ppm (s); ^{13}C NMR (150.92 MHz, $CDCl_3$): δ=137.7 (d, $^3J_{CP}$=15.3 Hz, C arom), 129.3 (s, 2 × CH arom), 128.5 (s, 2 × CH arom), 126.7 (s, CH arom), 71.4 (d, $^2J_{CP}$=6.6 Hz, CH *i*Pr), 71.3 (d, $^2J_{CP}$=6.7 Hz, CH *i*Pr), 69.3 (d, $^1J_{CP}$=162.5 Hz, CH-P), 37.8 (s, CH_2), 24.2 (d, $^3J_{CP}$=4.0 Hz, CH_3), 24.1 (d, $^3J_{CP}$=4.1 Hz, CH_3), 24.0 (d, $^3J_{CP}$=4.9 Hz, CH_3), 24.0 ppm (d, $^3J_{CP}$=4.9 Hz, CH_3); IR (ATR): $\tilde{\nu}$=3307, 1739, 1374, 1217, 986 cm^{-1}; HRMS (ESI): calc. for $[C_{14}H_{23}O_4P+Na]^+$: *m*/*z* 309.1227 [*M*+Na^+], found: *m*/*z* 309.1226; elemental analysis calc. (%) for $C_{14}H_{23}O_4P$: C 58.73, H 8.10; found: C 58.42, H 8.18; *ee* determination by chiral stationary phase HPLC [Chiralpak® OH-QD-AX, 150 × 4 mm, *n*-heptane + 0.1% *i*PrOH/*i*PrOH, 9:1: R_T [(*S*)-**30**] = 11.44 min (∫99.3), R_T [(*R*)-**30**] = 10.35 min (∫0.7); ee ≥ 98.5%.

(*R*)-Diisopropyl (1-azido-2-phenylethyl)phosphonate [(*R*)-81]: Substitution of the hydroxyl group by an azide was accomplished following **general procedure E**, starting from (*S*)-**30** (220 mg, 0.76 mmol), dissolved in dry toluene, and using DIAD (223 mg, 1.1 mmol) as azoester component. Completion of the reaction was observed after 16 h at room temperature. Purification was performed by column chromatography (*n*-heptane/EtOAc, 1:3) to furnish the desired product (*R*)-**81** as a yellowish oil (0.191 g, 0.61 mmol, 81%); R_f=0.62 (*n*-heptane/EtOAc, 1:3); α_D^{20}=−65.7 (*c*=1.05 in acetone); ^{1}H NMR (400.27 MHz, $CDCl_3$): δ=7.37-7.20 (m, 5H, CH arom), 4.88-4.74 (m, 2H, 2 × CH *i*Pr), 3.57 (ddd, $^3J_{BH}$=$^2J_{PH}$=11.8 Hz, $^3J_{AH}$=2.9 Hz, 1H, CH-P), 3.19 (ddd, $^2J_{AB}$=14.5 Hz, $^3J_{AP}$=6.0 Hz, $^3J_{AH}$=2.9 Hz, 1H, CH_2), 2.83, ddd, $^2J_{AB}$=14.5 Hz, $^3J_{BH}$=11.8 Hz, $^3J_{PB}$=7.4 Hz, 1H, CH_2), 1.37 (d, $^3J_{HH}$=6.2 Hz, 6H, 2 × CH_3), 1.37 (d, $^3J_{HH}$=6.2 Hz, 3H, CH_3), 1.36 ppm (d, $^3J_{HH}$=6.6 Hz, 3H, CH_3); ^{31}P NMR ($CDCl_3$, 242.92 MHz): δ=19.30 ppm (s); ^{13}C NMR (100.65 MHz, $CDCl_3$): δ=137.3 (d, $^3J_{CP}$=15.6 Hz, C arom), 129.0 (s, 2 × CH arom), 128.7 (s, 2 × CH arom), 127.0 (s, CH arom), 72.0 (d, $^2J_{CP}$=7.9 Hz, CH *i*Pr), 71.9 (d, $^2J_{CP}$=7.8 Hz, CH *i*Pr), 59.5 (d, $^1J_{CP}$=155.8 Hz, CH-P), 35.0 (s, CH_2), 24.2 (d, $^3J_{CP}$=3.7 Hz, 2 × CH_3), 24.0 ppm (d, $^3J_{CP}$=4.8 Hz, 2 × CH_3); IR (ATR): $\tilde{\nu}$=3455, 1739, 1375, 1217, 632 cm^{-1}; HRMS (ESI): calc. for $[C_{14}H_{22}N_3O_3P+Na]^+$: *m/z* 334.1291 [$M+Na^+$], found: *m/z* 334.1295; elemental analysis calc. (%) for $C_{14}H_{22}N_3O_3P$: C 54.01, H 7.12; found: C 53.80, H 7.50.

(*R*)-1-Amino-2-phenylethyl)phosphonic acid, (*R*)-phosphaphenylalanine [(*R*)-64]: (*R*)-Azidophosphonate **81** (186 mg, 0.60 mmol), can be transformed to (*R*)-phosphaphenylalanine (**64**, 94 mg, 0.47 mmol, 78%) by **general procedure F**, followed by **general procedure G** (reaction time 16 h). The obtained white solid was lyophilized to remove remaining traces of water; R_f=0.15 [*i*PrOH/H_2O/NH_3 (aq., 25%), 6:3:1]; m.p. 274-276°C; α_D^{20}=−59.81 (*c*=0.53 in water); ^{1}H NMR (600.25 MHz, D_2O): δ=7.49-7.44 (m, 2H, CH arom), 7.42-7.37 (m, 3H, CH arom), 3.57 (ddd, $^3J_{HB}$=$^2J_{HP}$=12.1 Hz, $^3J_{HA}$=4.2 Hz, 1H, CH-P), 3.42 ppm (ddd, $^2J_{AB}$=14.8 Hz, $^3J_{AH}$=$^3J_{AP}$=4.2 Hz, 1H, CH_2), 2.93 (ddd, $^2J_{AB}$=14.9 Hz, $^3J_{BH}$=12.1 Hz, $^3J_{BP}$=7.9 Hz, 1H, CH_2); ^{31}P NMR (242.97 MHz, D_2O): δ=12.38 ppm (s); ^{13}C NMR (150.93 MHz, D_2O): δ=135.9 (d, $^3J_{CP}$=13.0, C arom), 129.2 (s, 2 × CH arom), 129.2 (s, 2 × CH arom), 127.6 (s, CH arom), 50.9 (d, $^1J_{CP}$=141.4 Hz, CH-P), 34.2 ppm (s, CH_2); IR (ATR): $\tilde{\nu}$=3389, 2203, 1653, 632 cm^{-1}; HRMS (ESI): calc. for $[C_8H_{12}NO_3P+H]^+$: *m/z* 202.0628 [$M+H^+$], found: *m/z* 202.0627; elemental analysis calc. (%) for $C_8H_{12}NO_3P$: C 47.77, H 6.01; found: C 47.98, H 5.78; ee determination by chiral stationary phase HPLC [Chiralpak® OH-QN-AX, 150 × 4 mm, H_3PO_4 (2 M, aqueous)/MeOH, 1:9, (pH 4.0, adjusted with Et_3N)]: R_T [(*S*)-**64**]=19.86 min (∫0.4), R_T [(*R*)-**64**]=21.45 min (∫99.6); ee ≥ 99%.

8. Phosphatryptophane

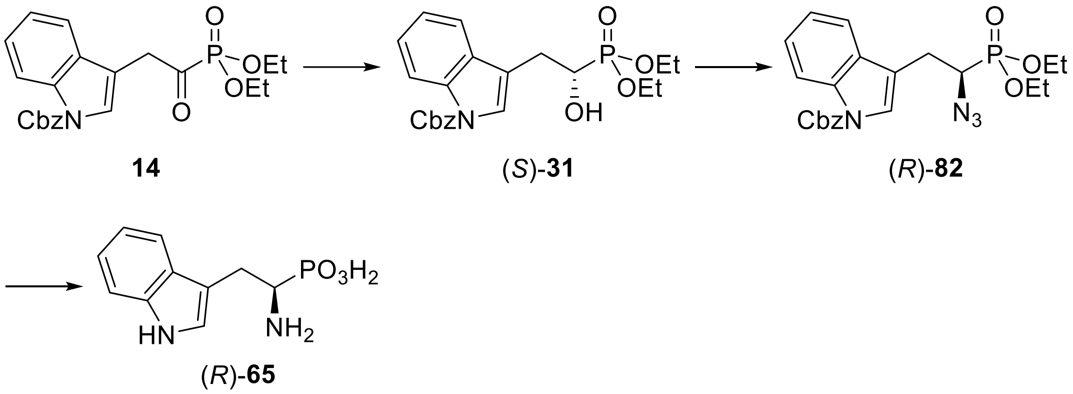

Benzyl 2-(2-(diethoxyphosphoryl)-2-oxoethyl)-1H-indole-1-carboxylate (14): Ketophosphonate **14** was obtained as yellowish crystalline solid starting from 2-(1-((Benzyloxy)carbonyl)-1H-indol-2-yl)acetic acid[5] (1.035 g, 3.34 mmol) by following **general procedure A** within 16 h at 35°C. The crude product was used without further purification for the next step; ^{31}P NMR (162.03 MHz, $CDCl_3$): δ=19.98 (s, unknown impurity, ∫0.02), 19.26 and −1.35 (2 × d, J_{PP}=19.6 Hz, unknown impurity, ∫0.01), 14.22 (s, unknown impurity, ∫0.01), 12.47 (s, enol-form of **14**, ∫0.77), 9.06 (s, unknown impurity, ∫0.03), 7.42 (s, unknown impurity, ∫0.04), −0.99 (s, unknown impurity, ∫0.02), −2.82 (s, keto-form of **14**, ∫0.10).

(*S*)-Benzyl 2-(2-(diethoxyphosphoryl)-2-hydroxyethyl)-1H-indole-1-carboxylate [(*S*)-31]: (*S*)-**32** was obtained from crude keto-phosphonate **14** (approx.1.7 mmol[1]) following **general procedure D** within 18 h using (*R*,*R*)-**7** (0.02 equiv., see **general procedure C**) as catalyst. Purification was performed by column chromatography (*n*-heptane/EtOAc, 1:1) to furnish the desired product as colorless solid (0.497 g, 1.1 mmol, 65%); R_f=0.32 (*n*-heptane/EtOAc, 1:1); α_D^{20}=+21.36 (*c*=1.175 in acetone); m.p. 101-102°C; ^{1}H NMR (600.25 MHz, $CDCl_3$): δ=8.16 (broad s, 1H, CH arom), 7.57 (s, 1H, CH arom), 7.54 (d, $^3J_{HH}$=7.7 Hz, 1H, CH arom), 7.48-7.43 (m, 2H, 2 × CH arom), 7.42-7.33 (m, 3H, 3 × CH arom), 7.31 (t, $^3J_{HH}$=7.6 Hz, 1H, CH arom), 7.26-7.22 (m, 1H, CH arom), 5.41 ($^2J_{AB}$=12.2 Hz, 2H, CH_2 Bn), 4.23-4.12 (m, 5H, 2 × CH_2 Et + CH-P), 3.22, dddd, $^2J_{AB}$=14.8 Hz, $^3J_{HH}$=6.9 Hz, $^3J_{HH}$=2.3 Hz, $^4J_{HH}$=0.8 Hz, 1H, C$\underline{H}_2$-CH-P), 3.06-2.97 (m, 1H, C$\underline{H}_2$-CH-P), 2.70 (broad s, 1H, OH), 1.33 (t, $^3J_{HH}$=7.1 Hz, 3H, CH_3 Et), 1.29 ppm (t, $^3J_{HH}$=7.0 Hz, 3H, CH_3 Et); ^{31}P NMR ($CDCl_3$, 242.92 MHz): δ=23.68 ppm (s); ^{13}C NMR (176.15 MHz, $CDCl_3$): δ=150.7 (s, C=O), 135.6 (s, C arom), 135.1 (s, C arom), 130.3 (s, C arom), 128.7 (s, 2 × CH arom), 128.7 (s, CH arom), 128.5 (s, 2 × CH arom), 124.8 (s, CH arom), 123.9 (s, CH arom), 122.9 (s, CH arom), 119.0 (s, CH arom), 117.0 (d, $^3J_{CP}$=16.3 Hz, C arom), 115.3 (s, CH arom), 68.6 (s, CH_2 Bn), 67.34 (d, $^1J_{CP}$=162.2 Hz, CH-P), 62.8 (d, $^2J_{CP}$=6.7 Hz, CH_2 Et), 62.8 (d, $^2J_{CP}$=6.9 Hz, CH_2 Et), 27.4 (d, $^2J_{CP}$=1.9 Hz, $\underline{C}H_2$-CH-P), 16.5 (d, $^3J_{CP}$=5.1 Hz, CH_3 Et), 16.5 ppm (d, $^3J_{CP}$=5.6 Hz, CH_3 Et); IR (ATR): $\tilde{v}$=3223, 1726, 1453, 1403, 743 cm^{-1}; elemental

analysis calc. (%) for $C_{22}H_{26}NO_6P$: C 61.25, H 6.07, N 3.25; found: C 61.05, H 6.16, N 3.24; *ee* determination by chiral stationary phase HPLC [Chiralpak® AS-3, 150 × 4.6 mm, *n*-heptane/*i*-propanol, 8:2: R_T [(*S*)-**31**] = 22.82 min (∫99.5), R_T [(*R*)-**31**] = 7.38 min (∫0.5); ee ≥ 99.0%.

(*R*)-Benzyl 2-(2-azido-2-(diethoxyphosphoryl)ethyl)-1H-indole-1-carboxylate [(*R*)-82]: Substitution of the hydroxyl group by an azide was accomplished following **general procedure E**, starting from (*S*)-**31** (452 mg, 1.04 mmol), dissolved in dry toluene, and using DIAD (305 mg, 1.51 mmol) as azoester component. Completion of the reaction was observed after 4 h at room temperature. Purification was performed by column chromatography (*n*-heptane/EtOAc, 1:1) to furnish the desired product as a yellowish oil (0.452 g, 1.00 mmol, 95%); R_f=0.5 (*n*-heptane/EtOAc, 1:1); α_D^{20}=−39.91 (*c*=1.2 in acetone); ^{1}H NMR (600.25 MHz, $CDCl_3$): *δ*=8.18 (broad s, 1H, CH arom), 7.56 (s, 1H, CH arom), 7.53 (d, $^3J_{HH}$=7.7 Hz, 1H, CH arom), 7.49-7.44 (m, 2H, 2 × CH arom), 7.43-7.30 (m, 4H, 4 × CH arom), 7.28-7.24 (m, 1H, CH arom) 5.44 ($^2J_{AB}$=12.2 Hz, 2H, CH_2 Bn), 4.28-4.19 (m, 4H, 2 × CH_2 Et), 3.75 (ddd, $^3J_{AH}$=3.0 Hz, $^2J_{PH}$=$^3J_{BH}$=11.5 Hz, 1H, CH-P), 3.27 (ddd, $^2J_{AB}$=15.3 Hz, $^3J_{AP}$=6.6 Hz, $^3J_{AH}$=3.0 Hz, 1H, C$\underline{H}_2$-CH-P), 2.95 (ddd, $^2J_{AB}$=15.3 Hz, $^3J_{BH}$=11.5 Hz, $^3J_{BP}$=7.4 Hz, 2H, C$\underline{H}_2$-CH-P), 1.37 (t, $^3J_{HH}$=7.0 Hz, 3H, CH_3 Et), 1.36 ppm (t, $^3J_{HH}$=7.0 Hz, 3H, CH_3 Et); ^{31}P NMR (242.92 MHz, $CDCl_3$): *δ*=21.21 ppm (s); ^{13}C NMR (150.92 MHz, $CDCl_3$): *δ*=150.7 (s, C=O), 135.7 (s, C arom), 135.0 (s, C arom), 129.9 (s, C arom), 128.8 (s, 2 × CH arom), 128.7 (s, CH arom), 128.5 (s, 2 × CH arom), 125.0 (s, CH arom), 123.9 (s, CH arom), 123.1 (s, CH arom), 118.7 (s, CH arom), 116.7 (d, $^3J_{CP}$=16.6 Hz, C arom), 115.5 (s, CH arom), 68.7 (s, CH_2 Bn), 63.2 (d, $^2J_{CP}$=7.1 Hz, CH_2 Et), 63.1 (d, $^2J_{CP}$=6.9 Hz, CH Et), 57.2 (d, $^1J_{CP}$=155.2 Hz, CH-P), 24.8 (s, $\underline{C}H_2$-CH-P), 16.6 (d, $^3J_{CP}$=5.3 Hz, CH_3 Et), 16.5 ppm (d, $^3J_{CP}$=5.4 Hz, CH_3 Et); IR (ATR): $\tilde{v}$=2104, 1730, 1453, 1245, 1015 cm^{-1}; elemental analysis calc. (%) for $C_{22}H_{25}N_4O_5P$: C 57.89, H 5.52, N 12.27; found: C 57.66, H 5.53, N 12.01.

(*R*)-(1-Amino-2-(1H-indol-2-yl)ethyl)phosphonic acid, (*R*)-phosphatryptophan [(*R*)-65]: Azidophsophonate **82** (0.100 g, 0.22 mmol) was deprotected following by **general procedure F**, followed by **general procedure G** but in this case the reaction mixture was stirred at room temperature for 16 h in this case. The obtained solid was purified by ion exchange chromatography using water as eluent to give aminophosphonic acid **65** (14 mg, 0.06 mmol, 27%) as colorless crystals. R_f=0.41 [*i*PrOH/H_2O/NH_3 (aq., 25%), 6:3:1]; α_D^{20}=−29.3 [*c*=0.45 in NaOH (1M)]; m.p. 280°C (decomp.); ^{1}H NMR (700.25 MHz, D_2O): *δ*=7.73 (d, $^3J_{HH}$=7.9 Hz, 1H, CH arom), 7.55 (d, $^3J_{HH}$=8.1 Hz, 1H, CH arom), 7.36 (s, 1H, CH arom), 7.30 (dd, $^3J_{HH}$=$^3J_{HH}$=7.3 Hz, 1H, CH arom), 7.21 (t, $^3J_{HH}$=$^3J_{HH}$=7.7 Hz, 1H, CH arom), 3.55 (ddd, $^2J_{AB}$=$^3J_{AH}$=15.4 Hz, $^3J_{AP}$=3.8 Hz, 1H, C$\underline{H}_2$-CH-P), 3.12 (ddd, $^2J_{AB}$=15.4 Hz, $^3J_{BP}$=3.6 Hz, $^3J_{BH}$=7.6 Hz, 2H, C$\underline{H}_2$-CH-P), 3.12 ppm (ddd, $^3J_{BH}$=7.6 Hz, $^2J_{PH}$=12.2 Hz, $^3J_{AH}$=15.4 Hz, 1H, CH-P); ^{31}P NMR (242.97

MHz, D_2O): *δ*=12.75 ppm (s); ^{13}C NMR (150.94 MHz, D_2O): *δ*=136.5 (s, C arom), 126.3 (s, C arom), 125.1 (s, CH arom), 122.2 (s, CH arom), 119.4 (s, CH arom), 118.3 (s, CH arom), 112.0 (s, CH arom), 108.0 (s, C arom), 49.5 (d, $^{1}J_{CP}$=141.1 Hz, CH-P), 24.26 ppm (s, CH_2); IR (ATR): $\tilde{\nu}$=3256, 1728, 1423, 1199, 1026 cm^{-1}; elemental analysis calc. (%) for $C_{10}H_{13}N_2O_3P$: C 50.00, H 5.46, N 11.66; found: C 50.17, H 5.39, N 11.78.

9. Phosphatyrosine

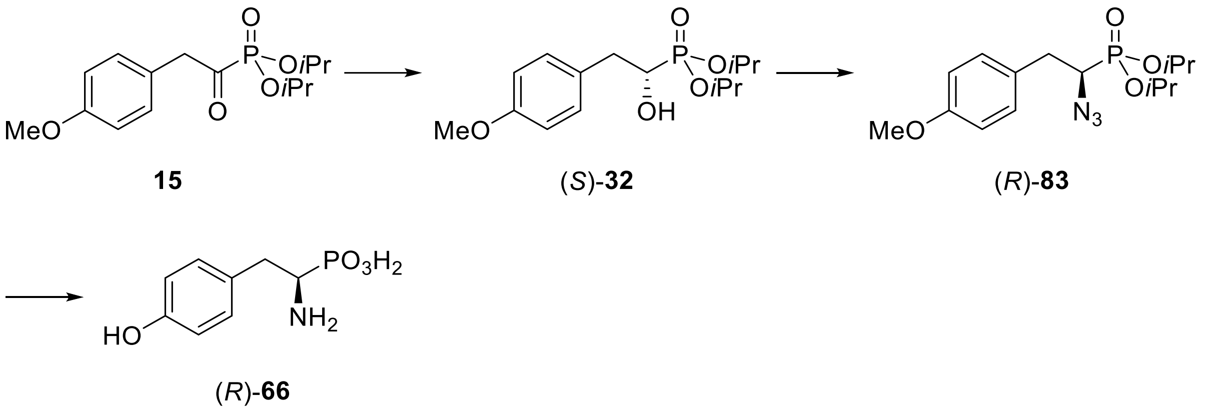

Diisopropyl (2-(4-methoxyphenyl)acetyl)phosphonate (15): Ketophosphonate **15** was obtained as yellowish crystalline solid starting from 2-(4-methoxyphenyl)acetic acid (830 mg, 5.0 mmol) by following **general procedure A**. Acyl chloride formation needed 4 h at 35°C; subsequent ketophosphonate formation was complete within 4 h at 0°C; ^{31}P NMR (162.03 MHz, $CDCl_3$): δ=11.79 (s, ∫0.67, enol-form), 4.41 (s, ∫0.07, HP(O)(O*i*Pr)$_2$), −3.41 (s, ∫0.05, unknown impurity), −3.99 ppm (s, ∫0.21, keto-form).

(*S*)-Diisopropyl 1-hydroxy-2-(4-methoxyphenyl)-ethylphosphonate [(*S*)-32]: (*S*)-**32** was obtained from crude keto-phosphonate **15** (approx. 1.76 mmol mmol[1]) following **general procedure D** within 16 h using (*R,R*)-**7** (0.02 equiv., see **general procedure C**) as catalyst. The crude residue was purified by flash chromatography (*n*-heptane/EtOAc 1:1) to yield the desired product as brownish crystals (424 mg, 1.34 mmol, 76%); R_f=0.25 (*n*-heptane/EtOAc 1:1); m.p. 68-73°C; α_D^{20}=+28.9 (*c*=0.79 in acetone); ^{1}H NMR (400.27 MHz, $CDCl_3$): δ=7.17 (m, 2H, 2 × CH arom), 6.84 (m, 2H, 2 × CH arom), 4.81-4.71 (m, 2H, 2 × CH *i*Pr), 3.96 (ddd, $^{2}J_{HP}$=5.2 Hz, $^{3}J_{HA}$=3.2 Hz, $^{3}J_{HB}$=10.6 Hz, 1H, CH-P), 3.77 (s, 3H, OCH_3), 3.06 (ddd, $^{2}J_{AB}$=14.4 Hz, $^{3}J_{HA}$=3.2 Hz, $^{3}J_{AP}$=6.3 Hz, 1H, CH_2), 2.82 (ddd, $^{2}J_{AB}$=14.4 Hz, $^{3}J_{HB}$=10.6 Hz, $^{3}J_{BP}$=9.7 Hz, 1H, CH_2), 1.99 (broad s, 1H, OH), 1.33 (d, $^{3}J_{HH}$=6.2 Hz, 6H, 2 × CH_3 *i*Pr), 1.33 (d, $^{3}J_{HH}$=6.2 Hz, 3H, CH_3 *i*Pr), 1.31 ppm (d, $^{3}J_{HH}$=6.2 Hz, 3 H, CH_3); ^{31}P NMR (162.03 MHz, $CDCl_3$): δ=22.13 ppm (s); ^{13}C NMR (150.93 MHz, $CDCl_3$): δ=158.6 (s, C arom), 130.4 (s, 2 × CH arom), 129.5 (s, C arom), 114.0 (s, 2 × CH arom), 71.3 (d, $^{2}J_{CP}$=7.0 Hz, CH *i*Pr), 71.3 (d, $^{2}J_{CP}$=7.1 Hz, CH *i*Pr), 69.4 (d, $^{1}J_{CP}$=162.3 Hz, CH-P), 55.3 (s, CH_3-O), 36.9 (s, CH_2), 24.2 (d, $^{3}J_{CP}$=3.6 Hz, CH_3

*i*Pr), 24.2 (d, $^3J_{CP}$=3.7 Hz, CH_3 *i*Pr), 24.1 (d, $^3J_{CP}$=4.7 Hz, CH_3 *i*Pr), 24.1 ppm (d, $^3J_{CP}$=4.9 Hz, CH_3 *i*Pr); IR (ATR): $\tilde{\nu}$=3279, 2978, 1612, 1513, 1467, 1385, 1246, 984, 890 cm^{-1}; HRMS (ESI): calc. for $[C_{15}H_{25}O_5P+Na]^+$: *m/z* 339.1331 [M+Na^+], found: *m/z* 339.1332; *ee* determination by chiral stationary phase HPLC (Chiralpak® OH-QD-AX, 250 × 4.6 mm, *n*-heptane + 0.1% *i*PrOH/*i*PrOH, 9:1; R_T [(*R*)-**32**] = 15.96 min (∫0.3), R_T [(*S*)-**32**] = 18.44 min (∫99.7); *ee* ≥ 99.4%.

(*R*)-Diisopropyl 1-azido-2-(4-methoxyphenyl)-ethylphosphonate [(*R*)-83]: Substitution of the hydroxyl group by an azide was accomplished following **general procedure E**, starting from (*S*)-**32** (165 mg, 0.52 mmol), dissolved in a 1:2 mixture of toluene and CH_2Cl_2, and using DIAD (151 mg, 0.75 mmol, 0.15 mL) as azoester component. Completion of the reaction was observed after 16 h at 35°C. The crude residue was purified by flash chromatography (*n*-heptane/EtOAc 1:1) to give the desired product as brownish oil (76 mg, 0.22 mmol, 43%); R_f=0.22 (*n*-heptane/EtOAc 1:1); α_D^{20}=−67.8 (*c*=0.85 in acetone); ^{1}H NMR (400.27 MHz, $CDCl_3$): δ=7.16 (m, 2H, 2 × CH arom), 6.85 (m, 2H, 2 × CH arom), 4.87-4.73 (m, 2H, 2 × CH *i*Pr), 3.78 (s, 3H, OMe), 3.51 (ddd, $^2J_{HP}$=14.8 Hz, $^3J_{HA}$=2.9 Hz, $^3J_{HB}$=11.8 Hz, 1H, CH-P), 3.13 (ddd, $^2J_{AB}$=14.5 Hz, $^3J_{HA}$=2.9 Hz, $^3J_{AP}$=5.9 Hz, 1H, CH_2), 2.77 (ddd, $^2J_{AB}$=14.5 Hz, $^3J_{HB}$=11.8 Hz, $^3J_{BP}$=7.3 Hz, 1H, CH_2), 1.37 (d, $^3J_{HH}$=8.9 Hz, 3H, CH_3 *i*Pr), 1.37 (d, $^3J_{HH}$=6.3 Hz, 6H, 2 × CH_3 *i*Pr), 1.37 ppm (d, $^3J_{HH}$=8.9 Hz, 3 H, CH_3); ^{31}P NMR (162.03 MHz, $CDCl_3$): δ= 9.40 ppm (s); ^{13}C NMR (150.93 MHz, $CDCl_3$): δ=158.7 (s, C arom), 130.1 (s, 2 × CH arom), 129.3 (d, $^3J_{CP}$=15.9 Hz, C arom), 114.1 (s, 2 × CH arom), 72.0 (d, $^2J_{CP}$=7.4 Hz, CH *i*Pr), 71.9 (d, $^2J_{CP}$=7.0 Hz, CH *i*Pr), 59.7 (d, $^1J_{CP}$=154.7 Hz, CH-P), 55.3 (s, CH_3-O), 34.2 (s, CH_2), 24.2 (d, $^3J_{CP}$=3.6 Hz, 2 × CH_3 *i*Pr), 24.1 ppm (d, $^3J_{CP}$=3.7 Hz, 2 × CH_3 *i*Pr); IR (ATR): $\tilde{\nu}$=2983, 2165, 2117, 1515, 1458, 1254, 1111, 1014, 891 cm^{-1}; elemental analysis calc. (%) for $C_{15}H_{24}N_3O_4P$: C 52.78, H 7.09; found: C 53.15, H 7.29[3]; ^{31}P NMR (162.03 MHz, $CDCl_3$) of (*R*)-**83** with chiral solvating agent: δ=97.49 (∫8.43, chiral solvating agent), 19.28 ppm [∫0.99, complex of chiral solvating agent with (*R*)-**83**]; 17.82 ppm [∫0.01, complex of chiral solvating agent with (*S*)-**83**]→ *ee* ≥ 98%.

(*R*)-1-Amino-2-(4-hydroxyphenyl)ethylphosphonic acid, (*R*)-phosphatyrosine [(*R*)-66]: (*R*)-Azidophosphonate **83** (54 mg, 0.15 mmol), can be transformed to (*R*)-phosphatyrosine (28 mg, 0.12 mmol, 74%) by **general procedure F**, followed by refluxing the hydrogenation residue in HBr in glacial acetic acid (30 %, 10 mL) for 4 h. The product was isolated as colorless powder after ion exchange chromatography as described in **general procedure G**; R_f=0.41 [*i*PrOH/H_2O/NH_3 (aq., 25%), 6:3:1]; α_D^{20}=−61.3 (*c*=0.85 in water); ^{1}H NMR (400.27 MHz, D_2O): δ=7.27 (symm m, 2H, 2 × CH arom), 6.93 (symm m, 2H, 2 × CH arom), 3.51 (td, $^2J_{HP}$=$^3J_{HB}$=12.7 Hz, $^3J_{HA}$=3.9 Hz ,1H, CH-P), 3.33 (td, $^2J_{AB}$=14.9 Hz, $^3J_{AH}$=3.9 Hz, 1H, CH_2), 2.87 ppm (ddd, $^2J_{AB}$=14.9 Hz, $^3J_{PB}$=7.8 Hz, $^3J_{BH}$=12.7 Hz, 1H, CH_2); ^{31}P NMR (162.03 MHz, D_2O): δ=12.23 ppm (s); ^{13}C NMR (150.93 MHz, D_2O): δ=154.8 (s, C arom), 130.6 (s, 2 × CH arom), 127.6 (d,

$^{3}J_{CP}$=13.3 Hz, C arom), 115.9 (s, 2 × CH arom), 51.0 (d, $^{1}J_{CP}$=141.0 Hz, CH-P), 33.37 ppm (s, CH_2); IR (ATR): $\tilde{\nu}$=3368, 2324, 2189, 2166, 2123, 2033, 1983, 1641, 890 cm^{-1}; elemental analysis calc. (%) for $C_8H_{12}NO_4P$: C 44.25, H 5.57; found: C 43.99, H 5.29, *ee* determination by chiral stationary phase HPLC [Chiralpak® OH-QD-AX, 150 × 4 mm, H_3PO_4 (2 M, aqueous) : MeOH, 1:9, (pH 4.0, adjusted with Me_3N)]: R_T [(*S*)-**66**] = 43.86 min (not detected), R_T [(*R*)-**66**] = 51.54 min (∫100.0); *ee* ≥ 99.9%.

10. Phosphaserine

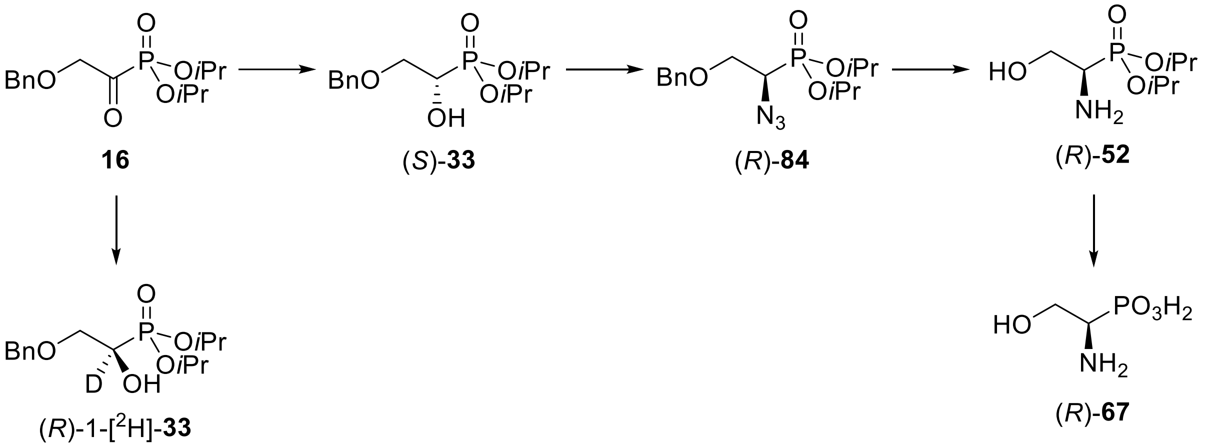

Diisopropyl 2-(benzyloxy)-1-oxoethylphosphonate (16): Ketophosphonate **16** (1.35 g, 4.3 mmol, 86%) was obtained by **general procedure B** starting from diisopropyltrimethylsilyl phosphite (3.58 g, 15 mmol).**Error! Bookmark not defined.** The resulting intermediate hydroxyphosphonate (±)-**33** was purified by MPLC using a solvent gradient (*n*-heptane/EtOAc; 12% to 100% ethyl acetate); R_f=0.22 (*n*-heptane/EtOAc, 1:1); ^{1}H NMR (600.25 MHz, $CDCl_3$): δ=7.36-7.27 (m, 5H, 5 × CH arom), 4.77-4.70 (m, 2H, 2 × CH *i*Pr), 4.61 (d, $^{2}J_{AB}$=11.9 Hz, 1H, CH_2 Bn), 4.57 (d, $^{2}J_{AB}$=11.9 Hz, 1H, CH_2 Bn), 4.08 (ddd, $^{3}J_{HP}$=9.6 Hz, $^{3}J_{HA}$=3.1 Hz, $^{3}J_{HB}$=7.8 Hz, 1H, CH-P), 3.80 (ddd, $^{2}J_{AB}$=10.4 Hz, $^{3}J_{AH}$=3.1 Hz, $^{3}J_{AP}$=10.4 Hz, 1H C$\underline{H}_2$CH-P), 3.73 (ddd, $^{2}J_{AB}$=10.4 Hz, $^{3}J_{BH}$=7.8 Hz, $^{3}J_{BP}$=5.2, 1H, C$\underline{H}_2$CH-P), 1.33 (d, $^{3}J_{HH}$=6.2 Hz, 3H, CH_3), 1.32 (d, $^{3}J_{HH}$=5.9 Hz, 3H, CH_3), 1.31 (d, $^{3}J_{HH}$=5.9 Hz, 3H, CH_3), 1.29 ppm (d, $^{3}J_{HH}$=6.2 Hz, 3H, CH_3); ^{31}P NMR (162.03 MHz, $CDCl_3$): δ=19.98 ppm (s); ^{13}C NMR (150.93 MHz, $CDCl_3$): δ=137.6 (s, C arom), 128.4 (s, 2 × CH arom), 127.8 (s, 2 × CH arom), 127.8 (s, CH arom), 73.4 (s, CH_2 Bn), 71.5 (d, $^{2}J_{CP}$=7.1 Hz, CH *i*Pr), 71.4 (d, $^{2}J_{CP}$=6.9 Hz, CH *i*Pr), 70.1 (d, $^{2}J_{CP}$=5.8 Hz, CH_2-O), 68.1 (d, $^{1}J_{CP}$=162.8 Hz, CH-P), 24.1 (d, $^{3}J_{CP}$=6.4 Hz, CH_3), 24.1 (d, $^{3}J_{CP}$=6.5 Hz, CH_3), 23.9 (d, $^{3}J_{CP}$=5.0 Hz, CH_3), 23.9 ppm (d, $^{3}J_{CP}$=5.4 Hz, CH_3); IR (ATR): $\tilde{\nu}$=3291, 2979, 1497, 1385, 1229, 1141, 985 cm^{-1}; HRMS (ESI): calc. for $[C_{15}H_{25}O_5P+H]^+$: *m/z* 317.1513 $[M+H]^+$; found: *m/z* 317.1506. The oxidation in the presence of DMP needed 4 h at room temperature until completion; ^{1}H NMR (400.27 MHz, $CDCl_3$): δ=7.37-7.25 (m, 5H, 5 × CH arom), 4.83-4.71 (m, 2H, 2 × CH *i*Pr), 4.62 (s, 2H, CH_2 Bn), 4.56 (d, $^{3}J_{HP}$=2.7 Hz, 2H, C$\underline{H}_2$C(O)-P), 1.35-1.31 ppm

(m, 12H, 4 × CH_3); ^{31}P NMR (162.04 MHz, $CDCl_3$): δ=4.42 (s, HP(O)(O*i*Pr)$_2$, ∫0.11), −5.24 (s, **16**, ∫0.86), −5.87 ppm (s, unknown impurity, ∫0.03).

(*S*)-Diisopropyl (2-(benzyloxy)-1-hydroxyethyl)phosphonate [(*S*)-33]: (*S*)-**33** was obtained from crude keto-phosphonate **16** (approx. 4.1 mmol[1]) following **general procedure D** within 15 h using (*R,R*)-**7** (0.02 equiv., see **general procedure C**) as catalyst. The residue was purified via MPLC using a solvent gradient (*n*-heptane/EtOAc, 12% to 100% EtOAc), to yield hydroxyphosphonate (*S*)-**33** (1.1 g, 3.48 mmol, 85%) as a colorless oil; α_D^{20}=+2.0 (*c*=1.0 in CH_2Cl_2); All spectroscopic data were identical to those of (±)-**33**. ^{31}P NMR (162.03 MHz, $CDCl_3$) of (*S*)-**33** with chiral solvating agent: δ=95.85 (∫0.52, chiral solvating agent), 20.08 [∫0.48 complex of chiral solvating agent with (*S*)-**33**], 19.9 ppm [∫0.001, complex of chiral solvating agent with (*R*)-**33**]; *ee* ≥ 99.5%.

(*R*)-Diisopropyl (2-(benzyloxy)-1-[^{2}H]-hydroxyethyl)phosphonate [(*R*)-1-[^{2}H]-33]: (*R*)-1-[^{2}H]-**33** was obtained from crude keto-phosphonate **16** (approx. 0.4 mmol[1]) following **general procedure D** within 16 h using (*S,S*)-**7** (0.04 equiv., see **general procedure C**) as catalyst. Adapted amounts of reagents are used in this case as follows: deuterated formic acid (49 mg, 1.04 mmol, 0.04 mL, 2.6 equiv.) and Et_3N (105 mg, 1.04 mmol, 0.14 mL, 2.6 equiv.). The residue was purified via MPLC using a solvent gradient (*n*-heptane/EtOAc, 12% to 100% EtOAc), to give the desired product as colorless oil (100 mg, 0.32 mmol, 79%); R_f=0.29 (EtOAc); ^{1}H NMR (700.40 MHz, $CDCl_3$): δ=7.35-7.25 (m, 5H, 5 × CH arom), 4.77-4.70 (m, 2H, 2 × CH *i*Pr), 4.58 (d, $^2J_{AB}$=11.8 Hz, 1H, CH_2 Bn), 4.55 (d, $^2J_{AB}$=11.8 Hz, 1H, CH_2 Bn), 3.78 (dd, $^2J_{AB}$=$^3J_{AP}$= 10.4 Hz, 1H, C$\underline{H}_2$CH-P), 3.71 (dd, $^2J_{AB}$=10.4 Hz, $^3J_{BP}$=5.3, 1H, C$\underline{H}_2$CH-P), 2.60 (br d, $^3J_{HP}$= 11.0 Hz, 1H, OH), 1.31 (d, $^3J_{HH}$=5.8 Hz, 3H, CH_3 *i*Pr), 1.30 (d, $^3J_{HH}$=6.2 Hz, 3H, CH_3 *i*Pr), 1.295 (d, $^3J_{HH}$=6.6 Hz, 3H, CH_3 *i*Pr), 1.27 ppm (d, $^3J_{HH}$=6.2 Hz, 3H, CH_3 *i*Pr); ^{31}P NMR (162.03 MHz, $CDCl_3$): δ=19.81 ppm (s); ^{13}C NMR (100.64 MHz, $CDCl_3$): δ=137.7 (s, C arom), 128.3 (s, 2 × CH arom), 127.8 (s, 2 × CH arom), 127.7 (s, CH arom), 73.4 (s, CH_2 Bn), 71.33 (d, $^2J_{CP}$=6.8 Hz, CH *i*Pr), 71.29 (d, $^2J_{CP}$=7.2 Hz, CH *i*Pr), 70.1 (d, $^2J_{CP}$=6.3 Hz, CH_2-O), 67.7 (dt, $^1J_{CP}$=167.2 Hz, $^1J_{CD}$=21.4 Hz CD-P), 24.09 (d, $^3J_{CP}$=2.9 Hz, CH_3 *i*Pr), 24.05 (d, $^3J_{CP}$=3.0 Hz, CH_3 *i*Pr), 23.87 (d, $^3J_{CP}$=4.8 Hz, CH_3 *i*Pr), 23.84 ppm (d, $^3J_{CP}$=4.9 Hz, CH_3 *i*Pr); HRMS (ESI): calc. for $[C_{15}H_{24}DO_5P+Na]^+$: *m/z* 340.1395 $[M+Na]^+$; found: *m/z* 340.1395; all other analytical data were in agreement to the non-deuterated compound.

(*R*)-Diisopropyl(1-azido-2-(benzyloxy)ethyl)phosphonate [(*R*)-84]: Substitution of the hydroxyl group by an azide was accomplished following **general procedure E**, starting from (*S*)-**33** (400 mg, 1.27 mmol), dissolved in dry CH_2Cl_2, and using DIAD (490 mg, 2.41 mmol, 0.47 mL) as azoester component. Completion of the reaction was observed after 18 h at 35°C. The residue was purified by MPLC with a solvent gradient (*n*-heptane/EtOAc, 12% to 100%

EtOAc) to yield azide (*R*)-**84** (310 mg, 0.91 mmol, 72%) as a colorless oil; R_f=0.53 (*n*-heptane/EtOAc, 1:1); α_D^{20}=−13.3 (*c*=1.15 in CH_2Cl_2); ^{1}H NMR (600.25 MHz, $CDCl_3$): δ=7.37-7.27 (m, 5H, 5 × CH arom), 4.83-4.70 (m, 2H, CH *i*Pr), 4.59 ($^2J_{AB}$=11.8 Hz, 2H, CH_2 Bn), 3.94-3.84 (m, 1H,CH-P), 3.79-3.68 (m, 2H, C<u>H</u>$_2$CH-P), 1.34 (d, $^3J_{HH}$=6.2 Hz, 3H, CH_3), 1.34 (d, $^3J_{HH}$=6.2 Hz, 3H, CH_3), 1.31 (d, $^3J_{HH}$=6.2 Hz, 3H, CH_3), 1.30 ppm (d, $^3J_{HH}$=6.2 Hz, 3H, CH_3); ^{31}P NMR (162.03 MHz, $CDCl_3$): δ=16.86 ppm (s); ^{13}C NMR (150.93 MHz, $CDCl_3$): δ=137.4 (s, C arom), 128.5 (s, 2 × CH arom), 127.9 (s, CH arom), 127.8 (s, 2 × CH arom), 73.5 (s, CH_2 Bn), 72.2 (d, $^2J_{CP}$=7.1 Hz, CH *i*Pr), 72.0 (d, $^2J_{CP}$=6.9Hz, CH *i*Pr), 68.5 (d, $^2J_{CP}$=5.7 Hz, CH_2-O), 58.2 (d, $^1J_{CP}$=156.4 Hz, CH-P), 24.2 (d, $^3J_{CP}$=3.3 Hz, CH_3), 24.1 (d, $^3J_{CP}$ = 3.7 Hz, CH_3), 23.9 (d, $^3J_{CP}$=4.7 Hz, CH_3), 23.8 ppm (d, $^3J_{CP}$=5.1 Hz, CH_3); IR (ATR): $\tilde{\nu}$=2981, 2361, 2095, 1454, 1386, 1257, 1103, 988, 741, 632 cm^{-1}; HRMS (ESI): calc. for $[C_{15}H_{24}O_4P+H]^+$: *m/z* 342.1578 $[M+H]^+$; found: *m/z* 342.1563; ^{31}P NMR (162.03 MHz, $CDCl_3$) of (*S*)-**84** with chiral solvating agent: δ=97.15 (∫0.58, chiral solvating agent), 16.86 [∫0.42 complex of chiral solvating agent with (*S*)-**84**], 16.51 ppm [∫0.001, complex of chiral solvating agent with (*R*)-**84**]; *ee* ≥ 99.5%.

(*R*)-(1-Amino-2-hydroxyethyl)phosphonic acid, (*R*)-phosphaserine [(*R*)-67]: The azide (*R*)-**84** (100 mg, 0.3 mmol) can be transformed to (*R*)-phosphaserine (501 mg, 2.99 mmol, 90%) by **general procedure F** to yield diisopropyl 1-amino-2-hydroxyethylphosphonate (**52**, 0.065 g, 0.29 mmol, 96%); ^{1}H NMR (400.27 MHz, *d*$_4$-methanol): δ=4.94-4.74 (m, 2H, CH *i*Pr), 4.10 (ddd, $^2J_{AB}$=11.6 Hz, $^3J_{AH}$=3.9 Hz, $^3J_{AP}$=4.0 Hz, 1H, CH_2), 3.46 (ddd, $^2J_{AB}$=1.6 Hz, $^3J_{BH}$=8.2 Hz, $^3J_{BP}$=5.6 Hz, 1H, CH_2), 3.58 (ddd, $^2J_{HP}$=12.2 Hz, $^3J_{XA}$=3.9 Hz, $^3J_{HB}$=8.2 Hz, 1H, CH-P), 1.40 (d, $^3J_{HH}$=6.2 Hz, 3H, CH_3), 1.40 (d, $^3J_{HH}$=6.2 Hz, 3H, CH_3), 1.40 (d, $^3J_{HH}$=6.2 Hz, 3H, CH_3), 1.40 ppm (d, ^3J=6.2 Hz, 3H, CH_3); ^{31}P NMR (162.03 MHz, *d*$_4$-methanol): δ=15.88 ppm (s); followed by **general procedure G** (reaction time 8 h). Lyophilisation finally yielded (*R*)-**67** as a colorless solid; m.p. 95-110°C (decomp.); α_D^{20}=−30.2 (*c*=0.75 in water), lit. for (*R*)-**67** α_D^{20}=−30.5 (0.525, H_2O)[6]; ^{1}H NMR (400.27 MHz, D_2O): δ= 4.10 (ddd, $^2J_{AB}$=12.6 Hz, $^3J_{AH}$=3.7 Hz, $^3J_{AP}$=5.5 Hz, 1H, CH_2), 3.84 (ddd, $^2J_{AB}$=12.6 Hz, $^3J_{BH}$=10.3 Hz, $^3J_{BP}$=4.2, 1H, CH_2CH-P), 3.47 ppm (ddd, $^2J_{HP}$=13.8 Hz, $^3J_{HA}$=3.7 Hz, $^3J_{HB}$=10.3 Hz, 1H, CH-P). ^{31}P NMR (162.04 MHz, D_2O): δ=9.71 ppm (s); all other analytical data were in agreement to those reported in the literature.[7]

11. Phosphaasparagine (first step)

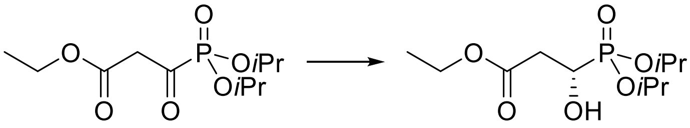

17 (*S*)-**34**

Ethyl 3-(diisopropoxyphosphoryl)-3-oxopropanoate (17): Ketophosphonate **17** was obtained starting from ethyl malonate (0.661 g, 5 mmol) by following **general procedure A** at

35°C within 16 h; ^{1}H NMR (400.27 MHz, $CDCl_3$): δ=5.88-5.71 (m, 1.2H, CH= from keto- and enol form + CH-C-P enol-form), 5.29 (s, 0.2H, enol-form, OH), 5.15-4.96 (m, 2H, =CH_2), 4.87-4.68 (m, 2H, CH *i*Pr), 3.01-2.89 (m, 2H, CH_2 enol-form + CH_2-C(O) keto-form), 2.40-2.32 (m, 1.6H, =CH-C$\underline{H}_2$ keto-form); ^{31}P NMR (162.03 MHz, $CDCl_3$): δ=4.42 (s, HP(O)(O*i*Pr)$_2$, ∫0.17), 3.09 (s, enol-form of **17**, ∫0.71), -3.44 (s, presumably keto-form of **17**, ∫0.09), −5.88 ppm (s, unknown impurity, ∫0.03).

(*S*)-Ethyl 3-(diisopropoxyphosphoryl)-3-hydroxypropanoate [(*S*)-34]: (*S*)-**34** was obtained from crude keto-phosphonate **17** (approx. 5.0 mmol[1]) following **general procedure D** within 16 h using (*R,R*)-**7** (0.01 equiv., see **general procedure C**) as catalyst. The crude residue was purified via MPLC using a solvent gradient (*n*-heptane/EtOAc, from 12 to 100 % EtOAc) to give the desired product as colorless oil (1.159 g, 4.10 mmol, 82%). R_f=0.19 (n-heptane:EtOAc = 1:3); α_D^{20}=+7.8 (*c*=0.825 in acetone); ^{1}H NMR ($CDCl_3$; 400.13 MHz): δ= 4.723 (sept, $^{3}J_{HH}$=6.2 Hz, 1H, CH *i*Pr), 4.720 (sept, $^{3}J_{HH}$=6.2 Hz, 1H, CH *i*Pr), 4.32-4.23 (m, 1H, CH-P), 4.14 (q, $^{3}J_{HH}$=7.2 Hz, 2H, C$\underline{H}_2$-CH_3), 3.89 (dd, $^{3}J_{HH}$=10.1 Hz, $^{3}J_{HP}$=5.7 Hz, 1H, OH), 2.70 (ddd, $^{3}J_{AB}$=16.4 Hz, $^{3}J_{HH}$ or $^{3}J_{HP}$=7.2 Hz, $^{3}J_{HH}$ or $^{3}J_{HP}$=3.8 Hz, 1H, CH_2), 2.64 (ddd, $^{3}J_{AB}$=16.4 Hz, $^{3}J_{HH}$ or $^{3}J_{HP}$=9.7 Hz, $^{3}J_{HH}$ or $^{3}J_{HP}$=8.4 Hz, 1H, CH_2), 1.30 (d, $^{3}J_{HH}$=6.5 Hz, 3H, CH_3 *i*Pr), 1.29 (d, $^{3}J_{HH}$=6.2 Hz, 6H, 2 × CH_3 *i*Pr), 1.29 (d, $^{3}J_{HH}$=6.5 Hz, 3H, CH_3 *i*Pr), 1.23 (t, $^{3}J_{HH}$=7.2 Hz, 3H, C$\underline{H}_3$-CH_2); ^{13}C NMR ($CDCl_3$, 100.65 MHz): δ=171.3 (d, $^{3}J_{CP}$=20.0 Hz, C=O), 71.5 (d, $^{2}J_{CP}$=7.1 Hz, CH *i*Pr), 71.4 (d, $^{2}J_{CP}$=7.3 Hz, CH *i*Pr), 64.7 (d, $^{1}J_{CP}$=169.4 Hz, CH-P), 60.9 (s, $\underline{C}H_2$-CH_3), 36.7 (d, $^{2}J_{CP}$=3.1 Hz, $\underline{C}H_2$-CH-P), 24.1 (d, $^{3}J_{CP}$=4.9 Hz, CH_3 *i*Pr), 24.0 (d, $^{3}J_{CP}$=5.1 Hz, CH_3 *i*Pr), 23.1 (d, $^{3}J_{CP}$=4.9 Hz, CH_3 *i*Pr), 23.0 (d, $^{3}J_{CP}$=5.0 Hz, CH_3 *i*Pr), 14.1 (s, $\underline{C}H_3$-CH_2); ^{31}P NMR ($CDCl_3$, 162.03 MHz): *δ*=20.73 ppm (s); ^{31}P NMR (242.99 MHz, $CDCl_3$) of (*S*)-**34** with chiral solvating agent: δ=96.24 [∫0.59, chiral solvating agent], 21.02 ppm [∫0.51, complex of chiral solvating agent with (*S*)-**34**]; complex of chiral solvating agent with (*R*)-**34** not detected; *ee* ≥ 99.9 %.IR (ATR): ν = 2980, 1738, 1219, 987, 631 cm^{-1}; HRMS (ESI): calc. for $[C_{11}H_{23}O_6P+Na]^+$: *m/z* 305.1125 $[M+Na]^+$; found: *m/z* 305.1128.

12. Phosphaglutamic acid

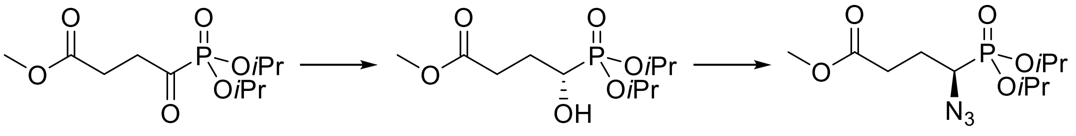

18 (*S*)-**35** (*R*)-**85**

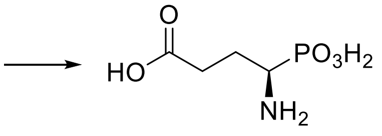

(*R*)-**68**

Methyl 4-(diisopropoxyphosphoryl)-4-oxobutanoate (18): Ketophosphonate **18** was obtained starting from mono-ethyl succinate (1.10 g, 8.23 mmol) by following **general procedure A** at 25°C within 3 h; ^{31}P NMR (162.03 MHz, $CDCl_3$): δ= 17.34 (s, unknown impurity, ∫0.02), 4.45 (s, HP(O)(O*i*Pr)$_2$, ∫0.08), −2.37 (s, unknown impurity, ∫0.01), −3.34 (s, unknown impurity, ∫0.16), −4.62 (s, **18**, ∫0.73).

(*S*)-Methyl 4-(diisopropoxyphosphoryl)-4-hydroxybutanoate [(*S*)-35]: (*S*)-**35** was obtained from crude keto-phosphonate **18** (approx. 1.00 mmol[1]) following **general procedure D** within 16 h using (*R,R*)-**7** (0.02 equiv., see **general procedure C**) as catalyst. The crude residue was purified via MPLC using a solvent gradient (CH_2Cl_2/acetone, from 2 to 20% acetone) to give the desired product as colorless oil (232 mg, 0.82 mmol, 82%); R_f=0.11 (CH_2Cl_2/acetone 9:1); α_D^{20}=+10.1 (*c*=0.7 in CH_2Cl_2); ^{1}H NMR (600.25 MHz, $CDCl_3$): δ= 4.81-4.69 (m, 2H, 2 × CH *i*Pr), 3.88-3.81 (m, 1H, CH-P), 3.68 (s, 3H, CH_3), 2.69 (broad t, $^3J_{HH}$=5.6 Hz 1H, OH), 2.63-2.52 (m, 2H, C$\underline{H}_2$-CO_2Me), 2.12 (dtd, $^2J_{AB}$=15.0 Hz, $^3J_{HH}$=7.6 Hz, $^3J_{HP}$=3.4 Hz, 1H, C$\underline{H}_2$-CH-P), 2.03-1.93 (m, 1H, C$\underline{H}_2$-CH-P), 1.35 (d, $^3J_{HH}$=6.1 Hz, 3H, CH_3 *i*Pr), 1.344 (d, $^3J_{HH}$=6.2 Hz, 3H, CH_3 *i*Pr), 1.340 ppm (d, $^3J_{HH}$=6.1 Hz, 6H, 2 × CH_3 *i*Pr); ^{31}P NMR (162.03 MHz, $CDCl_3$): δ=22.27 ppm (s); ^{13}C NMR (150.93 MHz, $CDCl_3$): δ=174.0 (s, C=O), 71.4 (d, $^2J_{CP}$=7.4 Hz, CH *i*Pr), 71.3 (d, $^2J_{CP}$=7.1 Hz, CH *i*Pr), 67.5 (d, $^1J_{CP}$=163.0 Hz, CH-P), 51.8 (s, CH_3), 30.3 (d, $^3J_{CP}$=13.9 Hz, $\underline{C}H_2$-CO_2Me), 26.5 (d, $^2J_{CP}$=1.5 Hz, $\underline{C}H_2$-CH-P), 24.2 (d, $^3J_{CP}$=3.8 Hz, CH_3 *i*Pr), 24.1 (d, $^3J_{CP}$=3.8 Hz, CH_3 *i*Pr), 24.0 (d, $^3J_{CP}$=4.7 Hz, CH_3 *i*Pr), 24.0 ppm (d, $^3J_{CP}$=4.7 Hz, CH_3 *i*Pr); IR (ATR): $\tilde{\nu}$=3281, 2980, 2936, 2875, 1737, 1438, 1375, 1216, 1176, 980 cm^{-1}; HRMS (ESI): calc. for $[C_{11}H_{23}O_6P+Na]^+$: *m/z* 305.1125 $[M+Na]^+$; found: *m/z* 305.1123; ^{31}P NMR (242.99 MHz, $CDCl_3$) of (*S*)-**35** with chiral solvating agent: δ=97.14 [∫0.70, chiral solvating agent], 22.41 ppm [∫0.70, complex of chiral solvating agent with (*S*)-**35**]; complex of chiral solvating agent with (*R*)-**35** not detected; *ee* ≥ 99.9 %.

Methyl (*R*)-4-(diisopropoxyphosphoryl)-4-azidobutanoate [(*R*)-85]: Hydroxyphosphonate (*S*)-**35** (0.892 g, 3.16 mmol) was converted to (*R*)-**85** following **general procedure E** with CH_2Cl_2 as solvent and DIAD as azoester component. Completion of the reaction was observed after 16 h at room temperature. The crude residue was purified by MPLC using a solvent gradient (*n*-heptane/EtOAc, 8% to 100% EtOAc) to give the desired product (*R*)-**85** as colorless oil (0.851 g, 2.77 mmol, 88%); α_D^{20}=−58.8 (*c*=0.77 in CH_2Cl_2); ^{1}H NMR (400.13 MHz, $CDCl_3$) δ=4.86-4.73 (m, 2H, 2 × CH *i*Pr), 3.70 (s, 3H, CH_3), 3.50 (td, $^3J_{HX}$=$^2J_{PH}$=11.3 Hz, $^3J_{A'H}$=3.9 Hz, 1H, CH-P), 2.63-2.54 (ddd, $^2J_{AB}$=16.5 Hz, $^3J_{AB'}$=6.8 Hz, $^3J_{AA'}$=7.8 Hz, 1H, C$\underline{H}_2$-CO_2Me), 2.53-2.47 (dd, $^2J_{AB}$=16.5 Hz, $^3J_{BP}$=7.6 Hz, 1H, C$\underline{H}_2$-CO_2Me), 2.18 (m, 1H, C$\underline{H}_2$-CH-P), 2.12 (dtd, $^2J_{A'B'}$=15.3 Hz, $^3J_{AA'}$=7.8 Hz, $^3J_{A'H}$=3.9 Hz, 1H, C$\underline{H}_2$-CH-P), 1.98-1.83 (m, 1H, C$\underline{H}_2$-CH-P), 1.40-1.34 ppm (m, 12H, 4 × CH_3 *i*Pr); ^{31}P NMR (161.98 MHz, $CDCl_3$): δ=19.34 ppm (s); ^{13}C NMR (176.12 MHz, $CDCl_3$): δ=172.8 (s, C=O), 72.0(d, $^2J_{CP}$=7.3 Hz, CH *i*Pr), 71.9 (d, $^2J_{CP}$=7.0 Hz,

CH *i*Pr), 56,9 (d, $^1J_{CP}$=157.4 Hz, CH-P), 51.8 (s, CH_3), 30.6 (d, $^3J_{CP}$=13.3 Hz, <u>C</u>H_2-CO_2Me), 24.2 (s, <u>C</u>H_2-CH-P), 24.2 (d, $^3J_{CP}$=3.9 Hz, CH_3 *i*Pr), 24.1 (d, $^3J_{CP}$=3.9 Hz, CH_3 *i*Pr), 24.0ppm (d, $^3J_{CP}$=4.8 Hz, 2 × CH_3 *i*Pr); IR (ATR): $\tilde{v}$=2981, 2937, 2101, 1737, 1438, 1376, 1251, 977 cm^{-1}; HRMS (ESI): calc. for $[C_{11}H_{22}N_3O_5P+Na]^+$: *m/z* 330.1190 $[M+Na]^+$; found: *m/z* 330.1189; ^{31}P NMR (161.98 MHz, $CDCl_3$) of (*R*)-**85** with chiral solvating agent: δ=97.23 [∫0.60, chiral solvating agent], 19.23 ppm [∫0.40, complex of chiral solvating agent with (*R*)-**85**]; complex of chiral solvating agent with (*S*)-**85** not detected; *ee* ≥ 99.9 %.

(*R*)-4-Amino-4-phosphoryl-butanoic acid, (*R*)-phosphaglutamic acid [(*R*)-68]: (*R*)-Azidophosphonate **85** (154 mg, 0.50 mmol), can be transformed to (*R*)-phosphaglutamic acid **in two steps** (70 mg, 0.38 mmol, 77%) by **general procedure F**, but in this case, 1 drop of a diluted ethanolic HCl solution (prepared from 1 drop conc. HCl + 2 mL EtOH) is added instead of one drop of conc. HCl. Hydrogenation is performed according to **general procedure G** (water for elution) to give (*R*)-**68** as colourless crystals; R_f=0.29 [*i*PrOH/H_2O/NH_3 (aq., 25%), 6:3:1]; α_D^{20}=−1.0 (*c*=0.60 in D_2O), α_D^{20}=−17.2 (*c*=0.74 in 1 M NaOH), lit. for (*R*)-**68**: α_{578}^{20}=−20 (*c*=1.0 in 1 M NaOH) [8]; ^1H NMR (600.25 MHz, D_2O): δ=3.34 (ddd, $^3J_{HH}$=6.3, $^3J_{HH}$=7.5 Hz, $^3J_{PH}$=13.8 Hz, 1H, CH-P), 2.69 (t, *J*=7.5 Hz, 2H, C<u>H_2</u>-CO_2H), 2.27-2.18 (m, 1H C<u>H_2</u>-CH-P), 2.05 ppm (dtd, $^3J_{AB}$=15.5 Hz, 2 × $^3J_{HH}$ =7.5 Hz, 1H, C<u>H_2</u>-CH-P); ^{31}P NMR (162.03 MHz, D_2O): δ=12.58 ppm (s); ^{13}C NMR (100.65 MHz, D_2O): δ=176.92 (s, C=O), 48.37 (d, $^1J_{CP}$=142.33 Hz, CH-P), 30.65 (d, $^2J_{CP}$=8.1 Hz, <u>C</u>H_2-CO_2H), 23.72 ppm (d, $^2J_{CP}$=2.5 Hz, <u>C</u>H_2-CH-P); IR (ATR): $\tilde{v}$=2875, 1687, 1626, 1537, 1123, 1086, 931, 701 cm^{-1}; HRMS (ESI): calc. for $[C_4H_{10}NO_5P+Na]^+$: *m/z* 206.0194 $[M+Na]^+$; found: *m/z* 206.0187.

13. Phosphaglutamine

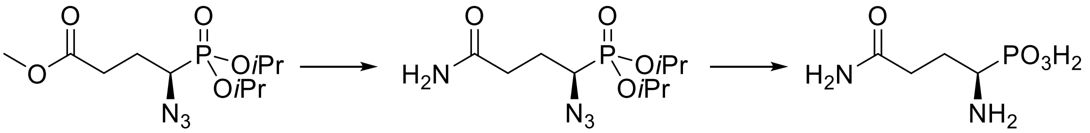

(*R*)-**85** (*R*)-**86** (*R*)-**69**

Diisopropyl (*R*)-(4-amino-1-azido-4-oxobutyl)-phosphonate [(*R*)-86]: (*R*)-**85** (285 mg, 0.93 mmol, 1 equiv.) was dissolved in THF (0.2 mL), then ammonia (25%, aqueous, 2 mL) and ammonium chloride (50 mg, 0.93 mmol, 1 equiv.) were added. After stirring for 16 h at room temperature the mixture was extracted with EtOAc (4 × 2 mL). The combined organic layers were dried ($MgSO_4$), filtered and the solvent was removed under reduced pressure to give (*R*)-**86** (233 mg, 0.80 mmol, 86%) as colorless oil; R_f=0.38 (EtOAc/EtOH 9:1); α_D^{20}=−50.95 (*c*=0.42 in CH_2Cl_2); ^1H NMR (400.27 MHz, $CDCl_3$): δ=5.65 (broad s, 1H, NH_2), 5.35 (broad s, 1H, NH_2), 4.88-4.71 (m, 2H, 2 × CH *i*Pr), 3.53 (ddd, $^2J_{HP}$=11.6, $^3J_{HH}$=10.0, $^3J_{HH}$=4.9 Hz, 1H, CH-P), 2.52 (dt, $^2J_{HH}$= 15.3 Hz, $^3J_{HH}$=6.8 Hz, 1H, CH_2), 2.41 (dt, $^2J_{HH}$= 15.3 Hz, $^3J_{HH}$=7.4 Hz, 1H, CH_2-

C(O)NH2), 2.28-2.13 (m, 1H, CH_2-C(O)NH_2), 2.05-1.88 (m, 1H, C<u>H2</u>-CH-P), 1.38 (m, 1H, C<u>H2</u>-CH-P), 1.37 (d, $^3J_{HH}$=6.2 Hz, 6H, 2 × CH_3 *i*Pr), 1.36 ppm (d, $^3J_{HH}$=6.1 Hz, 3H, CH_3 *i*Pr);^{31}P NMR (162.03 MHz, $CDCl_3$): δ=19.43 ppm (s); ^{13}C NMR (176.12 MHz, $CDCl_3$): δ=173.5 (s, C=O), 72.02 (d, $^2J_{CP}$=7.7 Hz, CH *i*Pr), 71.97 (d, $^2J_{CP}$=7.4 Hz, CH *i*Pr), 56.8 (d, $^1J_{CP}$=156.6Hz, CH-P), 31.8 (d, $^3J_{CP}$=11.3 Hz, <u>C</u>H2-C(O)NH_2), 24.7 (s, <u>C</u>H2-CH-P), 24.2 (d, $^3J_{CP}$=3.7 Hz, CH_3 *i*Pr), 24.1 (d, $^3J_{CP}$=3.7 Hz, CH_3 *i*Pr), 24.0 ppm (d, $^3J_{CP}$=4.6 Hz, 2 × CH_3 *i*Pr); IR (ATR): $\tilde{\nu}$=3409, 3345, 3217, 2981, 2935, 2097, 1677, 1236, 981 cm^{-1}; HRMS (ESI): calc. for $[C_{10}H_{21}N_4O_4P+Na]^+$: *m/z* 315.1193 $[M+Na]^+$; found: *m/z* 315.1195; ^{31}P NMR (161.98 MHz, $CDCl_3$) of (*R*)-**86** with chiral solvating agent: δ=96.48 [∫10.75, chiral solvating agent], 19.16 [∫0.005, complex of chiral solvating agent with (*S*)-**86**], 19.12 ppm [∫0.995, complex of chiral solvating agent with (*R*)-**86**]; *ee* ≥ 99.0 %.

(*R*)-(1,4-diamino-4-oxobutyl)-phosphonate, (*R*)-phosphaglutamine [(*R*)-69]: (*R*)-Azidophosphonate **86** (47 mg, 0.16 mmol), can be transformed to (*R*)-phosphaglutamine [(*R*)-**69**] by **general procedure F**. In this case, 1 drop of a diluted ethanolic HCl solution (prepared from 1 drop conc. HCl + 2 mL EtOH) is added instead of one drop of conc. HCl. Hydrogenation is followed by stirring of the intermediately obtained aminophosphonate with hydrobromic acid in glacial acetic acid (33% HBr, reaction time 87 h) at room temperature. The crude aminophosphonic acid was purified by ion exchange chromatography according to **general procedure G** (water for elution) to give (*R*)-**69** (17 mg, 58 %) as withe powder; heating was omitted during all synthetic steps; R_f=0.25 [*i*PrOH/H_2O/NH_3 (aq., 25%), 6:3:1]; m.p. 177-179°C; α_D^{23}=+6.5 (*c*=0.49 in D_2O); ^{1}H NMR (400.13 MHz, D_2O): δ=4.03 (s, unknown impurity), 3.34 (ddd, $^3J_{HH}$=6.4, $^3J_{HH}$=7.8 Hz, $^3J_{PH}$=13.8 Hz, 1H, CH-P), 2.69 (t, *J*=7.5 Hz, 2H, C<u>H2</u>-CO_2H), 2.29-2.15(m, 1H, C<u>H2</u>-CH-P), 2.14-1.94 ppm (m, 1H, C<u>H2</u>-CH-P); ^{31}P NMR (161.98 MHz, D_2O): δ=12.60 ppm (s); ^{13}C NMR (100.61 MHz, D_2O): δ=176.92 (s, C=O), 48.35 (d, $^1J_{CP}$=142.3 Hz, CH-P), 30.65 (d, $^2J_{CP}$=8.1 Hz, <u>C</u>H2-CO_2H), 23.72 ppm (d, $^2J_{CP}$=1.3 Hz, <u>C</u>H2-CH-P); IR (ATR): $\tilde{\nu}$=cm^{-1}; HRMS (ESI): calc. for $[C_4H_{11}N_2O_4P+H]^+$: *m/z* 183.0529 $[M+H]^+$; found: *m/z* 183.0529, calc. for $[C_4H_{11}N_2O_4P+Na]^+$: *m/z* 205.0349 $[M+Na]^+$; found: *m/z* 205.0348.

14. Phosphaisoserine (labeled)

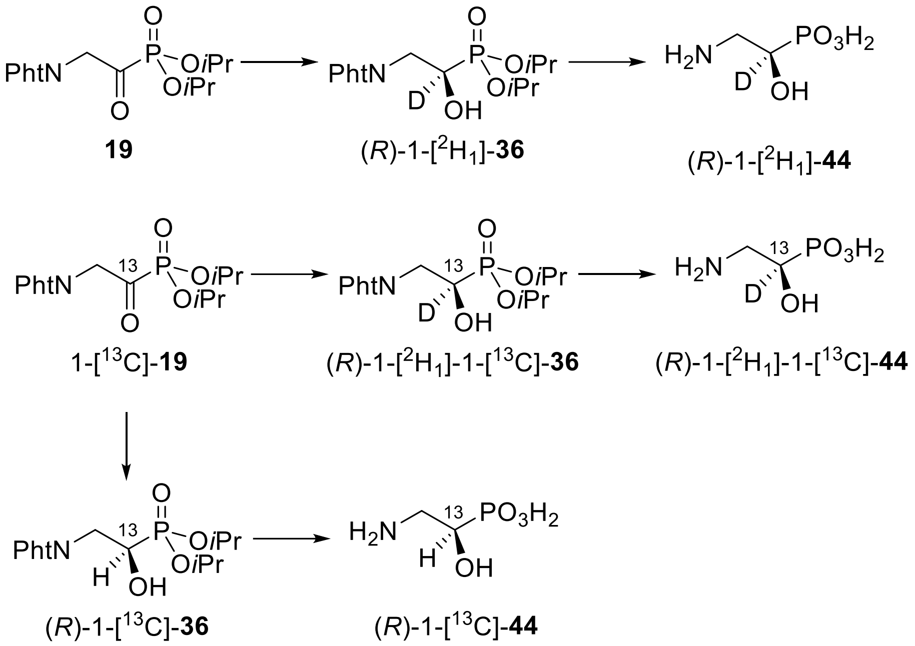

Diisopropyl 1-oxo-2-phthalimidoethyl phosphonate (19): The ketophosphonate **19** (14.5 g, 41 mmol, 82%) was obtained by a literature known procedure;[9] ^{1}H NMR (400.27 MHz, $CDCl_3$): δ=7.88-7.83 (m, 2H, CH arom), 7.75-7.70 (m, 2H, CH arom), 4.88 (d, $^3J_{HP}$=3.6 Hz, 1H, CH_2), 4.82 (sept, $^3J_{HH}$=6.2 Hz, 1H, CH *i*Pr), 4.80 (sept, $^3J_{HH}$=6.0 Hz, 1H, CH *i*Pr), 1.38 (d, $^3J_{HH}$=6.2 Hz, 6H, 2 × CH_3), 1.37 ppm (d, $^3J_{HH}$=6.2 Hz, 6H, 2 × CH_3); ^{31}P NMR (162.03 MHz, $CDCl_3$): δ=−5.84 ppm (s).

Diisopropyl (*R*)-1-[$^2H_1$]-1-hydroxy-2-phthalimido)-ethylphosphonate {(*R*)-1-[$^2H_1$]-36}: (*R*)-1-[$^2H_1$]-**36** (583 mg, 1.63 mmol, 78%) was obtained starting from **19** (742 mg, 2.1 mmol, 1 equiv.) after 18 h as a white powder by following **general procedure D**. Adapted amounts of reagents are used in this case as follows: deuterated formic acid (250 mg, 5.45 mmol, 0.21 mL, 2.6 equiv.), Et_3N (550 mg, 5.45 mmol, 0.76 mL, 2.6 equiv.) and (*S,S*)-**7** (0.04 equiv, see **general procedure C**). The crude product was purified after evaporation of the solvent via MPLC using a solvent gradient (*n*-heptane/EtOAc, 12% to 100% EtOAc); R_f=0.45, (*n*-heptane/EtOAc, 1:1); α_D^{20}=−21.2 (*c*=1.05 in CH_2Cl_2); ^{1}H NMR (400.27 MHz, $CDCl_3$): δ=7.86-7.81 (m, 2H, CH arom), 7.72-7.67 (m, 2H, CH arom), 4.81-4.69 (m, 2H, CH *i*Pr), 4.10 (dd, $^2J_{AB}$=14.5 Hz, $^3J_{AP}$=7.4 Hz, 1H, C$\underline{H_2}$-CD-P), 3.97 (dd, $^2J_{AB}$=14.5 Hz, $^3J_{BP}$=7.0 Hz, 1H, C$\underline{H_2}$-CD-P), 3.26 (s, 1H, OH), 1.35 (d, $^3J_{HH}$=6.2 Hz, 3H, CH_3), 1.314 (d, $^3J_{HH}$=6.2 Hz, 3H, CH_3), 1.312 (d, $^3J_{HH}$=6.2 Hz, 3H, CH_3), 1.29 ppm (d, $^3J_{HH}$=6.2 Hz, 3H, CH_3); ^{31}P NMR (161.98 MHz, $CDCl_3$): δ=18.93 ppm (s); ^{13}C NMR (150.93 MHz, $CDCl_3$): δ=168.5 (s, 2 × CO), 134.1 (s, 2 × CH arom), 132.2 (s, 2 × C arom), 123.5 (s, 2 × CH arom), 72.0 (d, $^2J_{CP}$=7.1 Hz, CH *i*Pr), 71.8 (d, $^2J_{CP}$=7.5 Hz, CH *i*Pr), 66.0 (td, $^1J_{CP}$=161.7 Hz, $^1J_{CD}$=21.8 Hz, CD), 39.9 (d, $^2J_{CP}$=7.6 Hz, CH_2), 24.2 (d,

$^3J_{CP}$=3.3 Hz, CH_3), 24.2 (d, $^3J_{CP}$=3.5 Hz, CH_3), 24.0 (d, $^3J_{CP}$=5.0 Hz, CH_3), 23.9 ppm (d, $^3J_{CP}$=5.0 Hz, CH_3); IR (ATR): $\tilde{\nu}$=3263, 2980, 1707, 1392, 1219, 1085, 981, 710 cm^{-1}; HRMS (ESI): calc. for $[C_{16}H_{21}DNO_6P+Na]^+$: *m/z* 379.1140 $[M+Na]^+$; found: *m/z* 379.1140; degree of deuteration: ^{1}H-NMR: δ=4.23-4.20 ppm (m, CH-P, non-deuterated **36**, ∫0.0087), 4.02 ppm (ABP-system deuterated **36**, CH_2, ∫2.0000); *ee* ≥ 99%.

(*R*)-1-[^{2}H]-2-amino-1-hydroxyethylphosphonic acid, (*R*)-1-[^{2}H]-phosphaisoserine {(*R*)-1-[^{2}H]-44}: (*R*)-1-[^{2}H]-**36** (855 mg, 2.42 mmol) was dissolved in 30 mL HCl (6M) and refluxed for 18 h. After removing excess HCl, the residue was taken up in an aqueous NH_3 solution (20 mL, 25%) and stirred at 60°C for 24 h. Ammonia was removed *in vacuo* and the residue was purified by cation exchange chromatography over Dowex 50W × 8 H^+ with water as eluent. The ninhydrin positive fractions were pooled and concentrated *in vacuo.* The remaining solid was crystallized from water/EtOH to yield (*R*)- 1-[$^2H_1$]-2-amino-1-hydroxyethylphosphonic acid ((*R*)-1-[^{2}H]-**44**, 0.327 mg, 2.30 mmol, 95%) as colorless needles; R_f (*i*PrOH/H_2O/NH_3(25%w/w) 6:3:2)=0.15; m.p. 262-267°C (decomp.); α_D^{20}=−33.6 (*c*=0.92 in water), lit. value of <u>non-deuterated</u> compound: α_D^{20}=−36.8 (*c* =0.99 in water);[9] ^{1}H NMR (400.27 MHz, D_2O): δ=3.32 ppm (dd, $^2J_{AB}$=13.4 Hz, $^3J_{AP}$=6.6 Hz, 1H, C<u>H</u>$_2$-CD-P) 3.12 (dd, $^2J_{AB}$=13.4 Hz, $^3J_{BP}$=6.5 Hz, 1H, C<u>H</u>$_2$-CD-P); ^{31}P NMR (162.03 MHz, D_2O): δ=15.00 ppm (s); ^{13}C NMR (D_2O, 100.65 MHz): *δ*=64.9 (dt, $^1J_{CP}$=155.2 Hz, $^1J_{CD}$=21.3 Hz, C(OH)D-P), 41.4 ppm (d, $^2J_{CP}$=8.9 Hz, CH_2); elemental analysis calc. for $C_2H_7DNO_4P$ (%): C 16.91, H 5.67, N 9.86, O 45.05, P 21.80; found: C 16.84, H 5.75, N 9.69, O 44.78, P 21.98; degree of deuteration: ^{1}H NMR: δ=3.96 ppm (td, CH-P, non-deuterated compound, ∫0.0128), 3.26 (ABP-system deuterated compound, CH_2, ∫2.0000); ee≥ 99%.

Diisopropyl 1-[^{13}C]-1-oxo-2-phthalimidoethyl phosphonate {1-[^{13}C]-19}: 1-[^{13}C]-**19** (3.45 g, 9.7 mmol, 78%) can be obtained similarly to **19** starting from 1-[^{13}C]-glycine (990 mg, 13.02 mmol); ^{1}H NMR (400.27 MHz, $CDCl_3$): δ=7.89-7.82 (m, 2H, CH arom), 7.75-7.70 (m, 2H, CH arom), 4.88 (dd, $^3J_{HP}$=$^2J_{H\text{-}13C}$=3.8 Hz, 1H, CH_2), 4.81 (sept, $^3J_{HH}$=6.2 Hz, 1H, CH *i*Pr), 4.80 (sept, $^3J_{HH}$=6.2 Hz, 1H, CH *i*Pr), 1.39 (d, $^3J_{HH}$=6.2 Hz, 6H, 2 × CH_3), 1.38 ppm (d, $^3J_{HH}$=6.2 Hz, 6H, 2 × CH_3); ^{31}P NMR (162.03 MHz, $CDCl_3$): δ=−5.85 ppm (d, $^1J_{13C\text{-}P}$=177.8 Hz).

Diisopropyl (*R*)-1-[^{13}C]-1-hydroxy-2-phthalimido-ethylphosphonate {(*R*)-1-[^{13}C]-36}: (*R*)-1-[^{13}C]-**36** (1.792 g, 5.03 mmol, 79%) was obtained following **general procedure D** within 18 h starting from 1-[^{13}C]-**19** (2.26 g, 6.37 mmol) using (*S,S*)-**7** (0.01 equiv., **see general procedure C**) as catalyst. The crude product was purified via MPLC using a solvent gradient (*n*-heptane/EtOAc, 12% to 100% EtOAc); R_f=0.46, (*n*-heptane/EtOAc, 1:1); ^{1}H NMR (400.27 MHz, $CDCl_3$): δ=7.88-7.80 (m, 2H, 2 × CH arom), 7.74-7.66 (m, 2H, 2 × CH arom), 4.84-4.68 (m, 2H, 2 × CH *i*Pr), 4.41-4.30 (m, 0.5 H, ^{13}C(OH)<u>H</u>-P), 4.17-4.04 (m, 1H, C<u>H</u>$_2$-^{13}CD-P), 4.04-3.93 (m, 1H, C<u>H</u>$_2$-^{13}CD-P), 4.04-3.93 (m, 0.5 H, ^{13}C(OH)<u>H</u>-P), 1.36 (d, $^3J_{HH}$=6.2 Hz, 3H, CH_3),

1.32 (d, $^{3}J_{HH}$=6.2 Hz, 3H, CH_3), 1.32 (d, $^{3}J_{HH}$=6.2 Hz, 3H, CH_3), 1.30 ppm (d, $^{3}J_{HH}$=6.2 Hz, 3H, CH_3); ^{31}P NMR (162.03 MHz, $CDCl_3$): δ=19.04 ppm (d, $^{1}J_{13CP}$ =162.2 Hz).

(*R*)-1-[^{13}C]-2-amino-1-hydroxyethylphosphonic acid, (*R*)-1-[^{13}C]-phosphaisoserine {(*R*)-1-[^{13}C]-44}: (*R*)-1-[^{13}C]-**44** (851 mg, 5.99 mmol, 98%) can be obtained similarly starting from (*R*)-1-[^{13}C]-**36** (2.18 g, 6.11 mmol); R_f (*i*PrOH/H_2O/NH_3(25%w/w) 6:3:2)=0.16; m.p. 263-268°C (decomp.); α_D^{20}=−31.2 (*c*=1.04 in water), lit. value of [^{12}C]-compound: α_D^{20}=−36.8 (*c* =0.99 in water);[9] ^{1}H NMR (600.27 MHz, D_2O): δ=3.96 (dddd, $^{2}J_{HP}$=$^{3}J_{HB}$=10.0 Hz, $^{3}J_{AH}$=3.3 Hz, $^{1}J_{H13C}$=140.0 Hz, 1H, C(OH)<u>H</u>-P), 3.36 (ddd, $^{2}J_{AB}$=13.2 Hz, $^{3}J_{AP}$=6.2 Hz, $^{3}J_{AH}$=3.3 Hz, 1H, C<u>H</u>$_2$-^{13}C(OH)H-P), 3.16 ppm (m, 1H, C<u>H</u>$_2$-^{13}C(OH)H-P); ^{31}P NMR (242.99 MHz, D_2O): δ=17.53 ppm (d, $^{1}J_{CP}$=155.6 Hz); ^{13}C NMR (D_2O, 150.93 MHz): *δ*=67.8 (d, $^{1}J_{CP}$=155.6 Hz, C(OH)H-P), 43.9 ppm (dd, $^{1}J_{CC}$=36.6 Hz, $^{2}J_{CP}$=9.2 Hz, CH_2); elemental analysis calc. (%) for $C_2H_8NPO_4$: C 16.91, H 5.68, N 9.86; found: C 17.13, H 5.69, N 9.64.

Diisopropyl (*R*)-1-[^{2}H]-1-[^{13}C]-1-hydroxy-2-phthalimido)-ethylphosphonate {(*R*)-1-[^{2}H]-1-[^{13}C]-36}: (*R*)-1-[^{13}C]-1-[^{2}H]-**36** (515 mg, 1.44 mmol, 72%) was obtained starting from (*R*)-1-[^{13}C]-**19** (713 mg, 2.01 mmol) after 18 h as a white powder by following **general procedure D**. Adapted amounts of reagents are used in this case as follows: deuterated formic acid (167 mg, 4.02 mmol, 0.16 mL, 2.8 equiv.) was added to Et_3N (407 mg, 4.02 mmol, 0.56 mL, 2.8 equiv.) and (*S,S*)-**7** (0.03 equiv., see **general procedure C**). The crude product was purified via MPLC using a solvent gradient (*n*-heptane/EtOAc, 12% to 100% EtOAc); R_f=0.45 (*n*-heptane/EtOAc, 1:1); α_D^{20}=−19.4 (*c*=0.54 in CH_2Cl_2); ^{1}H NMR (600.27 MHz, $CDCl_3$): δ=7.86-7.82 (m, 2H, CH arom), 7.73-7.69 (m, 2H, CH arom), 4.76 (symm. m, 2H, CH *i*Pr), 4.11 (ddd, $^{2}J_{AB}$=14.6 Hz, $^{3}J_{AP}$=6.8 Hz; $^{2}J_{AH}$=4.9 Hz, 1H, CH_2-^{13}CD-P), 3.98 (dd, $^{2}J_{AB}$=14.6 Hz, $^{3}J_{BP}$=7.4 Hz, 1H, C<u>H</u>$_2$-^{13}CD-P), 3.01 (dd, $^{3}J_{HP}$=5.5 Hz, J_{H13C} = 2.7 Hz, 1H, OH), 1.36 (d, $^{3}J_{HH}$=6.2 Hz, 3H, CH_3), 1.33 (d, $^{3}J_{HH}$=6.1 Hz, 3H, CH_3), 1.32 (d, $^{3}J_{HH}$=6.2 Hz, 3H, CH_3), 1.31 ppm (d, $^{3}J_{HH}$=6.2 Hz, 3H, CH_3); ^{31}P NMR (162.03 MHz, $CDCl_3$): δ=18.93 ppm (d, $^{1}J_{CP}$=161.5 Hz); ^{13}C NMR (150.93 MHz, $CDCl_3$): δ=168.8 (s, 2 × C=O), 134.4 (s, 2 × CH arom), 132.2 (s, 2 × C arom), 123.7 (s, 2 × CH arom), 72.1 (d, $^{2}J_{CP}$=7.2 Hz, CH *i*Pr of C(OH)<u>D</u>-P), 72.0 (d, $^{2}J_{CP}$=7.3 Hz, CH *i*Pr of C(OH)D-P), 70.7 (d, $^{2}J_{CP}$=7.5 Hz, CH *i*Pr of C(OH)H-P), 69.6 (d, $^{2}J_{CP}$=7.5 Hz, CH *i*Pr of C(OH)H-P), 67.1 (d, $^{1}J_{CP}$=161.7 Hz, C(OH)H-P), 66.7 (td, $^{1}J_{CD}$=22.2 Hz, $^{1}J_{CP}$=161.5 Hz, C(OH)<u>D</u>-P), 40.2 (dd, $^{2}J_{CP}$=7.2 Hz, $^{2}J_{CD}$=37.9 Hz, CH_2) 24.34 (d, $^{3}J_{CP}$=7.0 Hz, CH_3), 24.32 (d, $^{3}J_{CP}$=7.1 Hz, CH_3), 24.2 (d, $^{3}J_{CP}$=4.9 Hz, CH_3), 24.1 ppm (d, $^{3}J_{CP}$=4.8 Hz, CH_3); HRMS (ESI): calc. for $[C_{15}[^{13}C]H_{21}DPO_6+Na]^+$: *m/z* 380.1174 [M+Na$^+$]; found: *m/z* 380.1165; *ee* determination by chiral stationary phase HPLC (Lux-Cellulose 1 (Chiracel OD-H), 250 × 4.6 mm, *n*-heptane + 0.1% *i*PrOH/*i*PrOH, 85:15; R_T ((*R*)-1-[^{13}C]-1-[^{2}H]-**36**) = 7.24 min (∫99.44), R_T ((*S*)-1-[^{13}C]-1-[^{2}H]-**36**) = 8.68 min (∫0.60); *ee* ≥ 98.8 %, chemical purity ≥99 %; degree of deuteration δ=4.31-4.27

ppm (m, ^{13}CH-P, non-deuterated compound, ∫0.0099), 4.04 ppm (ABXP-system deuterated compound, CH_2, ∫2.0000); *ee* ≥ 99%.

(*R*)-1-[^{2}H]-1-[^{13}C]-2-amino-1-hydroxyethylphosphonic acid, (*R*)-1-[^{2}H]-1-[^{13}C]-phosphaisoserine {(*R*)-1-[^{2}H]-1-[^{13}C]-44}: (*R*)-1-[^{2}H]-[^{13}C]-**44** (185 mg, 1.29 mmol, 90%) can be obtained similarly starting from (*R*)-1-[^{2}H]-[^{13}C]-**36** (514 mg, 1.43 mmol); R_f (*i*PrOH/H_2O/NH_3(25%w/w) 6:3:2)=0.12; m.p. 264-269°C (decomp.); α_D^{20}=−33.2 (*c*=0.78 in water), lit. value of unlabelled-compound: α_D^{20}=−36.8 (*c*=0.99 in water);[9] ^{1}H NMR (600.27 MHz, D_2O): δ=3.87 (tdd, $^3J_{HH}$=10.3 Hz, $^2J_{HP}$=3.6 Hz, $^1J_{H13C}$=140.2 Hz, 0.027 H, ^{13}C(OH)H-P), 3.37 (dd, $^2J_{AB}$=13.3 Hz, $^3J_{AP}$=6.5 Hz, 1H, CH_2-^{13}C(OH)D-P), 3.17 (ddd, $^2J_{AB}$=13.3 Hz, $^3J_{BP}$=4.5 Hz, $^3J_{B13C}$=6.3 Hz, 1H, CH_2-^{13}C(OH)D-P); ^{31}P NMR (162.03 MHz, D_2O): δ=15.5 ppm (d, $^1J_{CP}$=155.6 Hz); ^{13}C NMR (D_2O, 150.93 MHz): *δ*=65.22 (d, $^1J_{CP}$=155.6 Hz, -C(OH)H-P), 64.94 (td, $^1J_{CP}$=155.6 Hz, $^1J_{CD}$=21.6 Hz, -C(OH)D-P), 41.30 ppm (dd, $^1J_{CC}$=36.8 Hz, $^2J_{CP}$=8.9 Hz, CH_2); HRMS (ESI): calc. for $[C[^{13}C]H_7DNPO_4+H]^+$: *m/z* 144.0360 $[M+H^+]$; found: *m/z* 144.0360; elemental analysis calc. (%) for $C[^{13}C]H_7DNPO_4$: C 16.79, H 5.63, O 44.73, P 21.65; found: C 16.72, H 5.80, O 44.72, P 21.82; degree of deuteration: ^{1}H-NMR: δ=3.87 (tdd, ∫0.027, ^{13}C(OH)H-P), 3.27 ppm (ABXP-system deuterated compound, CH_2, ∫2.0000); *ee* ≥ 98.6%.

15. 4-Amino-1-hydroxybutylphosphonic acid

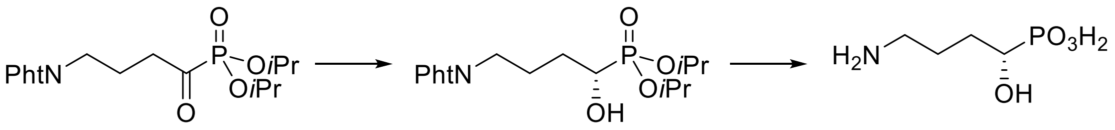

20 (*S*)-**37** (*S*)-**87**

Diisopropyl (4-(1,3-dioxoisoindolin-2-yl)butanoyl)phosphonate (20): Ketophosphonate **20** (4.570 g, 11.97 mmol, 75%) was obtained as yellowish liquid starting from 4-phthalimidobutanoyl chloride[10] (1.035 g, 3.34 mmol) by following **general procedure A** within 1 h at room temperature. The crude product was used without further purification for the next step; ^{31}P NMR ($CDCl_3$, 162.03 MHz): *δ*=9.58 (s, unknown impurity, ∫0.03), 4.43 (s, HP(O)(O*i*Pr)$_2$, ∫0.10), −3.38 (s, unknown impurity, ∫0.07), −4.54 (s, **20**, ∫0.80).

(*S*)-Diisopropyl (4-(1,3-dioxoisoindolin-2-yl)-1-hydroxybutylphosphonate [(*S*)-37]: (*S*)-**37** was obtained from crude keto-phosphonate **20** (approx. 0.8 mmol[1]) following **general procedure D** within 16 h using (*R*,*R*)-**7** (0.02 equiv., see **general procedure C**) as catalyst. Purification was performed by column chromatography (*n*-heptane/EtOAc, 1:1) to furnish the desired product as colorless crystals (0.226 g, 0.59 mmol, 74%); R_f=0.45 (EtOAc); α_D^{20}=−11.7 (*c*=1.03 in acetone); m.p. 92-93°C; ^{1}H NMR (400.27 MHz, $CDCl_3$): *δ*=7.84-7.78 (m, 2H, 2 × CH arom), 7.72-7.66 (m, 2H, 2 × CH arom), 4.78-4.65 (m, 2H, 2 × CH *i*Pr), 3.80 (ddd, $^2J_{PH}$=9.8 Hz,

$^3J_{HH}$=4.9 Hz, $^3J_{HH}$=3.8 Hz, 1H, CH-P), 3.79-3.67 (m, 2H, CH_2-N), 2.40 (broad s, 1H, OH), 2.05-1.92 (m, 2H, CH_2), 1.88-1.61 (m, 2H, CH_2), 1.30 ppm (d, $^3J_{HH}$=6.2 Hz, 12H, 4 × CH_3 *i*Pr); ^{31}P NMR (162.03 MHz, $CDCl_3$): *δ*=22.64 ppm (s); ^{13}C NMR (100.65 MHz, $CDCl_3$): *δ*=168.4 (s, 2 × C=O), 133.9 (s, 2 × CH arom), 132.1 (s, C arom), 123.2 (s, 2 × CH arom), 71.3 (d, $^2J_{CP}$=7.3 Hz, CH *i*Pr), 71.2 (d, $^2J_{CP}$=7.3 Hz, CH *i*Pr), 67.9 (d, $^1J_{CP}$=161.7 Hz, CH-P), 37.6 (s, CH_2-N), 28.5 (s, <u>C</u>H2-CH-P), 25.2 (d, $^3J_{CP}$=13.2 Hz, <u>C</u>H2-CH_2-N), 24.1 (d, $^3J_{CP}$=2.9 Hz, CH_3 *i*Pr), 24.1 (d, $^3J_{CP}$=3.7 Hz, CH_3 *i*Pr), 24.0 (d, $^3J_{CP}$=2.2 Hz, CH_3 *i*Pr), 24.0 (d, $^3J_{CP}$=2.9 Hz, CH_3 *i*Pr); IR (ATR): $\tilde{\nu}$=3223, 1711, 1394, 1216, 983 cm^{-1}; elemental analysis calc. (%) for $C_{18}H_{26}NO_6P$: C 56.39, H 6.84, N 3.65; found: C 56.36, H 7.07, N 3.64; *ee* determination by chiral stationary phase HPLC [Chiralpak® OH-QD-AX], 150 × 4 mm, *n*-heptane + 0.1% *i*PrOH/*i*PrOH, 9:1; R_T [(*S*)-**37**] = 18.43 min (∫99.7), R_T [(*R*)-**37**] = 15.98 min (∫0.3); *ee* ≥ 99.4%.

(*S*)-4-Amino-1-hydroxybutylphosphonic acid [(*S*)-87]: A mixture of (*S*)-**37** (0.664 g, 1.73 mmol) and aqueous HCl (12 mL, 6 M) was refluxed for 18 h. After cooling to room temperature, HCl was removed under reduced pressure. The residue was dissolved in a mixture of hydrazine hydrate (0.692 g, 13.8 mmol) and EtOH (10 mL) and refluxed for 18 h, cooled to room temperature and concentrated under reduced pressure. The residue was purified by ion exchange chromatography (Dowex 50W, H^+-form, water as eluent) followed by crystallization (H_2O/*i*PrOH) to give free phosphonic acid **87** (0.191 g, 1.13 mmol, 65%) as colorless crystals. α_D^{20}=−25.19 (*c*=1.02 in water); m.p. 260-261°C; ^{1}H NMR (400.27 MHz, D_2O): *δ*=3.83-3.70 (m, 1H, CH-P), 3.18-3.03 (m, 2H, CH_2-N), 2.05-1.67 ppm (m, 4H, 2 × CH_2); ^{31}P NMR (D_2O, 162.03 MHz): *δ*=19.52 ppm (s); ^{13}C NMR (100.65 MHz, D_2O): *δ*=68.2 (d, $^1J_{CP}$=157.0 Hz, CH-P), 39.3 (CH_2-N), 28.4 (<u>C</u>H2-CH-P), 23.8 (d, $^3J_{CP}$=13.3 Hz, <u>C</u>H2-CH_2-N); IR (ATR): $\tilde{\nu}$=3442, 1630, 1554, 1069, 1038, 912 cm^{-1}; elemental analysis calc. (%) for $C_4H_{12}NO_4P$: C 28.41, H 7.15, N 8.28; found: C 28.33, H 7.36, N 8.22.

16. Phosphacysteine

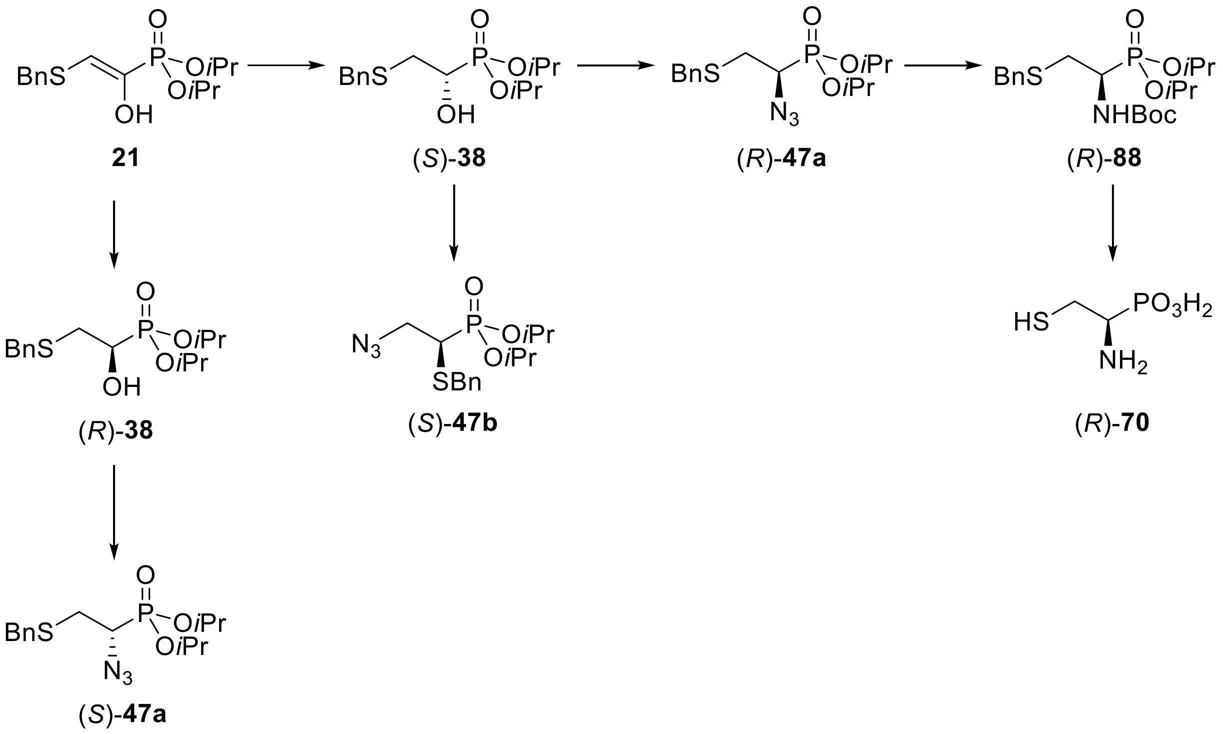

Diisopropyl (2-(benzylthio)acetyl)phosphonate (21): Ketophosphonate **21** can be obtained by **general procedure A** starting from benzylmercaptoacetic acid (910 mg, 5 mmol). Acyl chloride formation is complete within 5 h at 35°C. The obtained crude acyl chloride was purified by bulb-to-bulb distillation (b.p. 80-100°C, 0.3 mbar), before ketophosphonate **21** (1.5 g, 4.5 mmol, 91%) formation (3 h, 0°C). A small analytical sample of **21** was purified by crystallization from diisopropyl ether for characterization purposes, after which mainly the enol-form was obtained; ^{1}H NMR (400.27 MHz, $CDCl_3$): δ=7.38-7.22 (m, 5H, 5 × CH arom), 6.05 (d, $^{3}J_{HP}$=9.2 Hz 1H, C<u>H</u>-C-P), 5.41 (d, $^{3}J_{HP}$=18.7 Hz, OH), 4.64- 4.52 (m, 2H, 2 × CH *i*Pr), 3.89 (s, 2H, CH_2 Bn), 1.32 (d, $^{3}J_{HH}$=6.2, 6H, 2 × CH_3 *i*Pr), 1.23 ppm (d, $^{3}J_{HH}$=6.2, 6H, 2 × CH_3 *i*Pr); ^{31}P NMR (162.03 MHz, $CDCl_3$: δ=9.80 (s, unknown impurity, ∫0.03), 6.01 (s, enol-form of **21**, ∫0.96), 1.31 ppm (s, unknown impurity, ∫0.01).

Diisopropyl (*S*)-(2-(benzylthio)-1-hydroxyethyl)phosphonate [(*S*)-38]: (*S*)-**38** was obtained from crude keto-phosphonate **21** (approx. 3.8 mmol[1]) following **general procedure D** within 24 h using (*R,R*)-**7** (0.05 equiv., see **general procedure C**) as catalyst. The residue was purified via MPLC using a solvent gradient (*n*-heptane/EtOAc, 12% to 100% EtOAc), to yield hydroxyphosphonate (*S*)-**38** (900 mg, 2.7 mmol, 73%) as slightly reddish oil; R_f=0.24, (*n*-heptane/EtOAc, 1:1); α_D^{20}=+35.8 (*c*=0.76 in CH_2Cl_2); ^{1}H NMR (400.27 MHz, $CDCl_3$): δ=7.34-7.19 (m, 5H, 5 × CH arom), 4.79- 4.66 (m, 2H, 2 × CH *i*Pr), 3.81 (ddd, $^{3}J_{AH}$=2.8 Hz, $^{3}J_{BH}$=10.7 Hz, $^{2}J_{HP}$=6.8 Hz, 1H, CH-P), 3.77 ($^{2}J_{AB}$=13.5 Hz, 2H, CH_2 Bn), 2.86 (ddd, $^{2}J_{AB}$=14.3 Hz, $^{3}J_{AH}$=2.8 Hz, $^{3}J_{AP}$=4.9 Hz, 1H, C<u>H</u><u>2</u>-CH-P), 2.71 (ddd, $^{2}J_{AB}$=14.3 Hz, $^{3}J_{BH}$=10.7 Hz, $^{3}J_{BP}$=10.8

Hz, 1H, C$\underline{H}_2$-CH-P), 1.30 (d, $^3J_{HH}$=6.1 Hz, 3H, CH_3 *i*Pr), 1.29 (d, $^3J_{HH}$=6.2, 6H, 2 × CH_3 *i*Pr), 1.27 ppm (d, $^3J_{HH}$=6.8, 3H, CH_3 *i*Pr); ^{31}P NMR (162.03 MHz, $CDCl_3$): δ=20.25 ppm (s); ^{13}C NMR (150.93 MHz, $CDCl_3$): δ=137.8 (s, C arom), 129.2 (s, 2C, 2 × CH arom), 128.8 (s, 2C, 2 × CH arom), 127.5 (s, CH arom), 71.8 (d, $^2J_{CP}$=7.0 Hz, CH *i*Pr), 71.8 (d, $^2J_{CP}$=7.3 Hz, CH *i*Pr), 66.9 (d, $^1J_{CP}$=162.8 Hz, CH-P), 36.4 (s, CH_2 Bn), 33.9 (d, $^2J_{CP}$=2.2 Hz, $\underline{C}H_2$-CH-P), 24.1 (d, $^3J_{CP}$=8.8 Hz, CH_3 *i*Pr), 24.3 (d, $^3J_{CP}$=8.8 Hz, CH_3 *i*Pr), 24.20 (d, $^3J_{CP}$=7.2 Hz, CH_3 *i*Pr), 24.17 ppm (d, $^3J_{CP}$=7.3 Hz, CH_3 *i*Pr); IR (ATR): $\tilde{\nu}$=2980, 2374, 2208, 1454, 1232, 988 cm^{-1}; ^{31}P NMR of (*S*)-**38** with chiral solvating agent(162.03 MHz, $CDCl_3$): δ=96.24 (s, ∫1.14, chiral solvating agent), 20.42 [s, ∫0.99, complex of chiral solvating agent with (*S*)-**38**], 20.24 ppm [s, ∫0.01, complex of chiral solvating agent with (*R*)-**38**]; *ee* ≥ 98%.

Diisopropyl (*R*)-(1-azido-2-(benzylthio)ethyl)phosphonate [(*R*)-47a] and diisopropyl (*S*)-(2-azido-1-(benzylthio)ethyl)phosphonate [(*S*)-47b]: Substitution of the hydroxyl group by an azide was accomplished following **general procedure E**, starting from (*S*)-**38** (700 mg, 2.1 mmol), dissolved in dry CH_2Cl_2, and using DIAD (610 mg, 3.04 mmol, 0.59 mL) as azoester component. Completion of the reaction was observed after 18 h at room temperature. The residue was purified by MPLC with a solvent gradient (CH_2Cl_2/Et_2O, from 7% to 20% Et_2O) to yield azide (*R*)-**47a** (420 mg, 1.2 mmol, 57%) in admixture with its constitutional isomer (*S*)-**47b** (180 mg, 0.5 mmol, 24%) as colorless oil. The isomers can be separated by two subsequent MPLC runs using a solvent gradient (*n*-heptane/Et_2O, 2% to 10% Et_2O). By using CH_2Cl_2 instead of THF as the solvent in the above described reaction, the product ratio can be shifted to mainly obtain (*S*)-**47b** (52%) in admixture with (*R*)-**47a** (20%);

(*R*)-**47a**: R_f=0.53 (CH_2Cl_2/Et_2O 19:1); α_D^{20}=−59.3 (*c*=0.93 in $CHCl_3$); ^{1}H NMR (400.27 MHz, $CDCl_3$): δ=7.37-7.21 (m, 5H, 5 × CH arom), 4.79- 4.65 (m, 2H, 2 × CH *i*Pr), 3.78 ($^2J_{AB}$=13.6 Hz, 2H, CH_2 Bn), 3.41 (ddd, $^3J_{AH}$=2.7 Hz, $^3J_{BH}$=11.7 Hz, $^2J_{HP}$=12.5 Hz, 1H, CH-P), 2.87 (ddd, $^2J_{AB}$=14.3 Hz, $^3J_{AH}$=2.7 Hz, $^3J_{AP}$=4.8 Hz, 1H, C$\underline{H}_2$-CH-P), 2.68 (ddd, $^2J_{AB}$=14.3 Hz, $^3J_{BH}$=11.7 Hz, $^3J_{BP}$=6.4 Hz; 1H, C$\underline{H}_2$-CH-P), 1.31 (d, $^3J_{HH}$=6.1 Hz, 3H, CH_3 *i*Pr), 1.30 (d, $^3J_{HH}$=6.2 Hz, 3H, CH_3 *i*Pr), 1.29 (d, $^3J_{HH}$=6.0 Hz, 3H, CH_3 *i*Pr), 1.28 ppm (d, $^3J_{HH}$=6.2 Hz, 3H, CH_3 *i*Pr); ^{31}P NMR (162.03 MHz, $CDCl_3$): δ=17.43 ppm (s); ^{13}C NMR (150.93 MHz, $CDCl_3$): δ=138.0 (s, C arom), 129.2 (s, 2 × CH arom), 128.9 (s, 2 × CH arom), 127.5 (s, CH arom), 72.4 (d, $^2J_{CP}$=6.7 Hz, CH *i*Pr), 72.4 (d, $^2J_{CP}$=7.0 Hz, CH *i*Pr), 58.8 (d, $^1J_{CP}$=152.9 Hz, CH-P), 37.0 (s, CH_2 Bn), 31.3 (d, $^2J_{CP}$=2.3 Hz, $\underline{C}H_2$-CH-P), 24.4 (d, $^3J_{CP}$=3.7 Hz, CH_3 *i*Pr), 24.3 (d, $^3J_{CP}$=3.6 Hz, CH_3 *i*Pr), 24.2 (d, $^3J_{CP}$=5.1 Hz, CH_3 *i*Pr), 24.1 ppm (d, $^3J_{CP}$=5.0 Hz, CH_3 *i*Pr); IR (ATR): $\tilde{\nu}$=2979, 2098, 1454, 1385, 1302, 1247, 1104, 982 cm^{-1}; HRMS (ESI): calc. for $[C_{15}H_{24}N_3O_3PS+Na]^+$: *m/z* 380.1169 $[M+Na]^+$; found: *m/z* 380.1168.

(*S*)-**47b:** R_f=0.44 (CH_2Cl_2/Et_2O 19:1); α_D^{20}=−28.8 (*c*=0.97 in acetone); ^{1}H NMR (700.40 MHz, $CDCl_3$): δ=7.38-7.37 (m, 2H, 2 × CH arom), 7.34-7.31 (m, 2H, 2 × CH arom), 7.28-7.25 (m, 1H,

2 × CH arom), 4.77 (symm. m, 2H, 2 × CH *i*Pr), 4.06 (dd, $^2J_{AB}$=13.0 Hz, $^4J_{AP}$=1.3 Hz, 1H, CH_2 Bn), 3.94 (d, $^2J_{AB}$=13.0 Hz, 1H, CH_2 Bn), 3.65 (ddd, $^3J_{HA}$=13.0 Hz, $^2J_{HP}$=11.4 Hz, $^3J_{HB}$=4.5 Hz, 1H, CH-P), 3.42 (ddd, $^3J_{AH}$=13.0 Hz, $^2J_{AB}$=$^3J_{AP}$=8.5 Hz, 1H, C$\underline{H}_2$-CH-P), 2.74 (ddd, $^3J_{BP}$=17.2 Hz, $^2J_{AB}$=8.5 Hz, $^3J_{BH}$=4.5 Hz, 2H, C$\underline{H}_2$-CH-P), 1.34 (d, $^3J_{HH}$=6.1 Hz, 3H, CH_3), 1.33 (d, $^3J_{HH}$=6.6 Hz, 3H, CH_3), 1.32 (d, $^3J_{HH}$=6.8 Hz, 3H, CH_3), 1.31 ppm (d, $^3J_{HH}$=6.2 Hz, 3H, CH_3); ^{31}P NMR (161.98 MHz, $CDCl_3$): δ=20.09 ppm (s); ^{13}C NMR (176.12 MHz, $CDCl_3$): δ=137.1 (s, C arom), 129.5 (s, 2C, 2 × CH arom), 128.8 (s, 2C, 2 × CH arom), 127.59 (s, CH arom), 72.27 (d, $^2J_{CP}$=7.3 Hz, CH *i*Pr), 71.86 (d, $^2J_{CP}$=7.3 Hz, CH *i*Pr), 51.8 (d, $^2J_{CP}$=2.9 Hz, $\underline{C}H_2$-CH-P), 40.6 (d, $^1J_{CP}$=148.3 Hz, CH-P), 37.1 (d, $^3J_{CP}$=3.0 Hz, CH_2 Bn), 24.4 (d, $^3J_{CP}$=3.2 Hz, CH_3), 24.3 (d, $^3J_{CP}$=3.7 Hz, CH_3), 24.1 (d, $^3J_{CP}$=5.2 Hz, CH_3), 24.00 ppm (d, $^3J_{CP}$=5.6 Hz, CH_3); IR (ATR): $\tilde{\nu}$=2979, 2931, 2097, 1380, 1245, 1105, 978, 704 cm^{-1}; HRMS (ESI): calc. for $[C_{15}H_{24}N_3O_3PS+Na]^+$: *m/z* 380.1169 $[M+Na]^+$; found: *m/z* 380.1172; ^{31}P NMR (161.98 MHz, *d*$_8$-toluene) with chiral solvating agent: δ=95.31 [∫0.54, chiral solvating agent], 20.21 ppm [∫0.46, complex of chiral solvating agent with (*S*)-**47b**], [peak from complex of chiral solvating agent with (*R*)-**47b** not detected]; *ee* ≥ 99%.

Diisopropyl (*S*)-(1-azido-2-(benzylthio)ethyl)phosphonate [(*S*)-47a]: [(*S*)-**47a**] can be obtained similarly starting from hydroxyphosphonate (*R*)-**38**; (*S*)-**47a:** α_D^{20}=+56.1 (*c*=1.00 in $CHCl_3$); all other data were identical to those reported for (*R*)-**47a**.

***tert*-Butyl (*R*)-(2-(benzylthio)-1-(diisopropoxyphosphoryl)ethyl)carbamate [(*R*)-88]:** A mixture of the azide (*R*)-**47a** (200 mg, 0.56 mmol, 1.0 equiv.) with Pd/C (180 mg) and di-*tert*-butyl-dicarbonate (160 mg, 0.73 mmol, 1.3 equiv.) was dissolved in ethanol (30 mL) and the resulting reaction mixture was degassed. The reaction mixture was hydrogenated using a H_2-filled balloon for 24 h under continuous stirring. The catalyst was removed by filtration over Celite® and the solvent was evaporated *in vacuo* to obtain the carbamate (*R*)-**88** (200 mg, 0.46 mmol, 83%) after purification by MPLC using a solvent gradient (CH_2Cl_2/Et_2O, 7% to 40% Et_2O) as a colorless oil; R_f= 0.58 (CH_2Cl_2/Et_2O 4:1); ^{1}H NMR (400.27 MHz, $CDCl_3$): δ=7.36-7.17 (m, 5H, 5 × CH arom), 4.77-4.58 (m, 3H, NH, 2 × CH *i*Pr), 4.22-4.07 (m, 1H, CH-P), 3.73 (s, 2H, CH_2 Bn), 2.89 (ddd, $^2J_{AB}$=14.1 Hz, $^3J_{AH}$=3.8 Hz, $^3J_{AP}$=7.1 Hz, 1H, C$\underline{H}_2$-CH-P), 2.51 (ddd, $^2J_{AB}$=14.1 Hz, $^3J_{BH}$=10.8 Hz, $^3J_{BP}$=7.7 Hz; 1H, C$\underline{H}_2$-CH-P), 1.45 (s, 9H, 3 × CH_3 Boc), 1.30 (d, $^3J_{HH}$=6.2, 3H, CH_3 *i*Pr), 1.27 (d, $^3J_{HH}$=5.8 Hz, 3H, CH_3 *i*Pr), 1.26 (d, $^3J_{HH}$=5.9 Hz, 3H, CH_3 *i*Pr), 1.23 ppm (d, $^3J_{HH}$=6.2 Hz, 3H, CH_3 *i*Pr); ^{31}P NMR (162.03 MHz, $CDCl_3$): δ=21.13 ppm (s); ^{13}C NMR (150.93 MHz, $CDCl_3$): δ=155.7 (d, $^3J_{CP}$=7.8 Hz, C=O), 137.9 (s, C arom), 129.2 (s, 2 × CH arom), 128.7 (s, 2 × CH arom), 127.2 (s, CH arom), 80.2 (s, C Boc), 71.9 (d, $^2J_{CP}$=7.2 Hz, CH *i*Pr), 71.5 (d, $^2J_{CP}$=7.2 Hz, CH *i*Pr), 46.6 (d, $^1J_{CP}$=156.5 Hz, CH-P), 35.9 (s, CH_2 Bn), 32.4 (d, $^2J_{CP}$=7.5 Hz, $\underline{C}H_2$-CH-P), 28.5 (s, 3 × CH_3 Boc), 24.4 (d, $^3J_{CP}$=3.2 Hz, CH_3 *i*Pr), 24.2 (d,

$^3J_{CP}$=3.4 Hz, CH_3 *i*Pr), 24.0 (d, $^3J_{CP}$=4.7 Hz, CH_3 *i*Pr), 23.9 ppm (d, $^3J_{CP}$=4.7 Hz, CH_3 *i*Pr); HRMS (ESI): calc. for $[C_{20}H_{34}NO_5PS+Na]^+$: *m/z* 454.1788 $[M+Na]^+$, found; *m/z* 454.1793.

(*R*)-(1-amino-2-mercaptoethyl)phosphonic acid, phosphacysteine [(*R*)-70]:Error! Bookmark not defined. The carbamate (*R*)-**86** (285 mg, 0.66 mmol, 1.0 equiv.) was transformed to (*R*)-phosphacysteine (68 mg, 0.43 mmol, 62%) over two steps as follows: The starting material was dissolved in dry Et_2O (3 mL), and added to liquid NH_3 (25 mL) at −30°C. Sodium metal (42 mg, 1.83 mmol, 1.8 equiv.) was added in portions until a deep blue colour persisted. The reaction was quenched by addition of NH_4Cl (87 mg, 1.64 mmol) whereupon it became colorless. The reaction mixture was allowed to slowly come to room temperature in a fume hood. This gave the intermediate mercaptocarbamate in sufficient purity for the next step. ^1H NMR (400.27 MHz, d_4-methanol): δ=4.77-4.63 (m, 2H, 2 × CH *i*Pr), 4.02-3.88 (m, 1H, CH-P), 2.97-2.87 (m, 1H, $C\underline{H_2}$-CH-P), 2.73-2.61 (m, 1H, $C\underline{H_2}$-CH-P), 1.47 (s, 9H, 3 × CH_3 Boc), 1.36-1.30 ppm (m, 12 H, 4 × CH_3); ^{31}P NMR (162.03 MHz, d_4-methanol): δ=20.96 ppm (s). The residue was treated as described in **general procedure G** (reaction time 8 h, water as eluent for ion exchange chromatography). The solid residue was crystallized from hot water (60°C) by addition of EtOH to give (*R*)-**70**; m.p. 245-248°C (decomp.); α_D^{20}=−58.5 (*c*=0.55 in water); ^1H NMR (600.25 MHz, D_2O): δ=3.35 (ddd, $^3J_{AH}$=3.9 Hz, $^3J_{BH}$=10.3 Hz, $^2J_{HP}$=14.1, 1H, CH-P), 3.22 (ddd, $^2J_{AB}$=14.9 Hz, $^3J_{AH}$=3.9 Hz $^3J_{AP}$=8.0 Hz, 2H, $C\underline{H}_2$-CH-P), 2.81 ppm (ddd, $^2J_{AB}$=14.9 Hz, $^3J_{BH}$=10.3 Hz, $^3J_{BP}$=6.9 Hz, 2H, $C\underline{H}_2$-CH-P); ^{31}P NMR (162.03 MHz, D_2O): δ=10.58 ppm (s); ^{13}C NMR (150.93 MHz, D_2O): δ=51.56 (d, $^1J_{CP}$=136.9 Hz, CH-P), 23.15 ppm (s, $\underline{C}H_2$-CH-P); HRMS (ESI): calc. for $[C_2H_7NO_3PS-H]^-$: *m/z* 155.9889 $[M-H]^-$; found: *m/z* 155.9893.

Alternatively, a mixture of the constitutional isomers (*R*)-**47a** and (*S*)-**47b**, as obtained by **general procedure E** (before chromatographic separation) can be directly reacted as outlined above to finally produce a mixture of (*R*)-phosphacysteine and (*S*)-phosphaisocysteine. This mixture is easily separable by ion exchange chromatography: water for elution of (*R*)-**70**, followed by aqueous acetic acid (1 v/v%) for elution of (*S*)-phosphaisocysteine (Dowex 50W, H^+-form).

17. Phosphamethionine (intermediate)

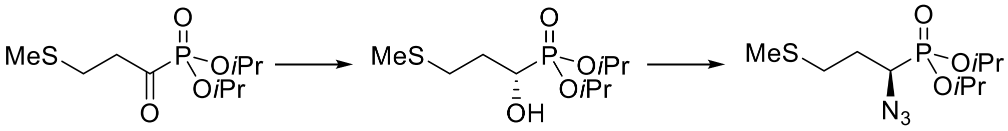

21 (*S*)-**39** (*R*)-**89**

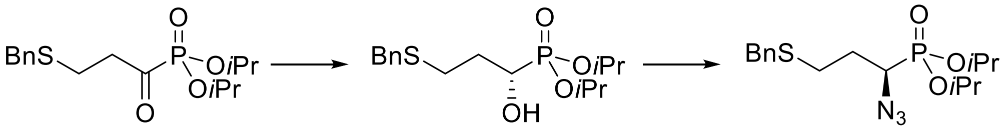

23 (*S*)-**40** (*R*)-**53**

Diisopropyl 1-oxo-3-(methylthio)propylphosphonate (22): Ketophosphonate **22** can be obtained by **general procedure A** starting from 3-(methylthio)-propionic acid (500 mg, 4.7 mmol). Acyl chloride formation is complete within 5 h at 35°C. Ketophosphonate **22** was formed within 3 h at 0°C. No further purification was attempted; ^{31}P NMR (162.03 MHz, $CDCl_3$: δ=139.48 (s, (O*i*Pr)$_3$P, ∫0.10), 18.12 (1 s, unknown impurities, ∫0.04), 17.74 and −2.54 (2 × d, J_{PP}=25.4 Hz, unknown impurity, ∫0.09), 9.25 (s, unknown impurity, ∫0.02), 5.74 (s, unknown impurity, ∫0.04), 4.41 (s, HP(O)(O*i*Pr)$_2$, ∫0.03), −3.35 (s, unknown impurity, ∫0.03), −3.64 ppm (s, **22**, ∫0.65).

Diisopropyl (*S*)-1-hydroxy-3-(methylthio)propylphosphonate [(*S*)-39]: (*R*)-**39** was obtained from crude keto-phosphonate **22** (approx. 3.5 mmol[1]) following **general procedure D** within 18 h using (*R,R*)-**7** (0.05 equiv., see **general procedure C**) as catalyst. The residue was purified via MPLC using a solvent gradient (*n*-heptane/EtOAc, 24% to 100% EtOAc), to yield hydroxyphosphonate (*S*)-**39** (897 mg, 3.3 mmol, 94%) as orange oil; R_f=0.35, (EtOAc); α_D^{20}=+30.0 (*c*=0.49 in acetone); ^{1}H NMR (400.27 MHz, $CDCl_3$): δ=4.81-4.68 (m, 2H, 2 × CH *i*Pr), 4.03-3.94 (m, 1H, CH-P), 2.74 (dddd, $^2J_{AB}$=13.2 Hz, $^3J_{AH}$=7.7 Hz, $^3J_{AH}$=5.3 Hz, $^3J_{AH}$=0.8 Hz, 1H, CH_2), 2.65 (dt, $^2J_{AB}$=13.2 Hz, $^3J_{BH}$=$^3J_{PH}$=7.7 Hz, 1H, CH_2), 2.63-2.52 (m, 1H, OH), 2.09 (s, 3H, CH_3-S), 2.07-1.89 (m, 2H, C$\underline{H_2}$-CH-P), 1.33 (d, $^3J_{HH}$=6.2 Hz, 3H, CH_3 *i*Pr), 1.33 (d, $^3J_{HH}$=6.2, 3H, CH_3 *i*Pr), 1.32 ppm (d, $^3J_{HH}$=6.2, 6H, 2 × CH_3 *i*Pr); ^{31}P NMR (162.03 MHz, $CDCl_3$): δ=23.28 (s, unknown impurity, ∫0.02), 22.70 ppm (s, **39**, ∫0.98); ^{13}C NMR (150.93 MHz, $CDCl_3$): δ=71.6 (d, $^2J_{CP}$=7.1 Hz, CH *i*Pr), 71.4 (d, $^2J_{CP}$=7.3 Hz, CH *i*Pr), 67.1 (d, $^1J_{CP}$=162.7 Hz, CH-P), 30.7 (d, $^3J_{CP}$=1.2 Hz,CH_2-S), 30.7 (d, $^2J_{CP}$=15.2 Hz, $\underline{C}H_2$-CH-P), 24.4 (d, $^3J_{CP}$=2.6 Hz, CH_3 *i*Pr), 24.3 (d, $^3J_{CP}$=2.8 Hz, CH_3 *i*Pr), 24.3 ppm (d, $^3J_{CP}$=4.7 Hz, 2 × CH_3 *i*Pr); IR (ATR): $\tilde{\nu}$=3297, 2979, 2919, 1682, 1386, 1223, 1106 cm^{-1}; HRMS (ESI): calc. for $[C_{10}H_{23}O_4PS+Na]^+$: *m/z* 293.0947 $[M+Na]^+$; found: *m/z* 293.0949; ^{31}P NMR (162.03 MHz, $CDCl_3$) of (*S*)-**39** with chiral solvating agent: δ=97.93 (s, ∫3.74 chiral solvating agent), 22.87 [s, ∫0.10 complex of chiral

solvating agent with (*R*)-**39**], 22.70 ppm [s, ∫0.90, complex of chiral solvating agent with (*S*)-**39**]; *ee* ≥ 80.0%.

(*R*)-Diisopropyl (1-azido-3-(methylthio)-propylphosphonate [(*R*)-89]: Substitution of the hydroxyl group by an azide was accomplished following **general procedure E**, starting from (*S*)-**39** (104 mg, 0.38 mmol), dissolved in dry CH_2Cl_2, and using DEAD as azoester component. The reaction was stopped after 18 h at 30°C. The residue was purified by MPLC with a solvent gradient (CH_2Cl_2/acetone, 0% to 3% acetone) to yield azide (*R*)-**89** (61 mg, 0.21 mmol, 54%) as colorless oil; R_f=0.43 (CH_2Cl_2/acetone, 39:1); α_D^{20}=−57.0 (*c*=0.96 in acetone), lit. for (*R*)-**89**: α_D^{20}=−83.8 (*c*=1.1 in acetone);[2] ^{1}H NMR (400.27 MHz, $CDCl_3$): δ=4.87-4.72 (m, 2H, 2 × CH *i*Pr), 3.68 (td, $^3J_{HH}$=3.4 Hz, $^2J_{HP}$=$^3J_{HH}$=11.4 Hz, 1H, CH-P), 2.74, (dddd, $^2J_{AB}$=13.2 Hz, $^3J_{AH}$=7.6 Hz, $^3J_{AH}$=4.5 Hz, $^4J_{AP}$=1.3 Hz, 1H, CH_2-S), 2.65-2.54 (m, 1H, CH_2-S), 2.10 (s, 3H, CH_3-S), 2.14-2.00 (m, 2H, C$\underline{H}_2$-CH-P), 1.97-1.82 (m, 1H, C$\underline{H}_2$-CH-P), 1.37 ppm (broad d, $^3J_{HH}$=6.3, 12H, 4 × CH_3 *i*Pr); ^{31}P NMR (162.03 MHz, $CDCl_3$): δ=20.10 (s, unknown impurity, ∫0.04), 19.93 ppm (s, **89**, ∫96.0); ^{1}H NMR (400.27 MHz, $CDCl_3$) of (*R*)-**89** with chiral solvating agent: δ=3.73-3.70 (most low-field part of td, ∫0.46, complex of chiral solvating agent with (*R*)-**89**], 3.64-3.61 (most high-field part of td, ∫0.05, complex of chiral solvating agent with (*S*)-**89**]; *ee* ≥ 80%; all other analytical data were in agreement to the literature.[2]

Diisopropyl 1-oxo-3-(benzylthio)-propylphosphonate (23): Ketophosphonate **23** can be obtained by **general procedure A** starting from 3-(benzylthio)-propionic acid (5.00 g, 25.5 mmol). Acyl chloride formation is complete within 20 h at 30°C. Ketophosphonate **23** was formed within 4 h at 25°C. No further purification was attempted; ^{31}P NMR (162.03 MHz, $CDCl_3$): δ=17.98 (s, unknown impurity, ∫0.02), 17.68 and −2.57 (2 × d, J_{PP}=26.1 Hz, unknown impurity, ∫0.06), 9.07 (s, unknown impurity, ∫0.05,), 5.59 (s, unknown impurity, ∫0.03), 4.45 (s, HP(O)(O*i*Pr)$_2$, ∫0.07), 3.33 (s, unknown impurity, ∫0.03), −4.70 ppm (s, **23**, ∫0.74).

(*S*)-Diisopropyl [3-(benzylthio)-1-hydroxypropyl]phosphonate [(*S*)-40]: (*S*)-**40** was obtained from crude keto-phosphonate **23** (approx. 1.02 mmol[1]) following **general procedure D** within 18 h using (*R,R*)-**7** (0.05 equiv., see **general procedure C**) as catalyst. The residue was purified via MPLC using a solvent gradient (*n*-heptane/EtOAc, 18% to 100% EtOAc), to yield hydroxyphosphonate (*S*)-**40** (346 mg, 1.00 mmol, 98%) as colorless oil; R_f=0.38 (*n*-heptane:EtOAc, 1:3); α_D^{20}=+29.6 (*c* 0.85, CH_2Cl_2); ^{1}H NMR (600.25 MHz, $CDCl_3$): δ = 7.32-7.22 (m, 5H, 5 × CH arom), 4.75 (sept, $^3J_{HH}$=6.2 Hz, 1H, CH *i*Pr), 4.73 (sept, $^3J_{HH}$=6.2 Hz, 1H, CH *i*Pr), 3.95 (ddd, $^3J_{HH}$=4.5 Hz, $^3J_{HH}$=3.4 Hz, $^2J_{HP}$=9.7 Hz, 1H, CH-P), 3.72 (J_{AB}=13.8 Hz, 2H, CH_2 Bn), 2.76 (broad s, 1H, OH), 2.68 (ddd, $^2J_{AB}$=13.2 Hz, $^3J_{AH}$=5.1 Hz, $^3J_{AH}$=7.7 Hz, 1H, CH_2-S), 2.58 (ddd, $^2J_{AB}$=13.2 Hz, $^3J_{BH}$=15.6 Hz, $^3J_{BH}$=7.9 Hz, 1H, CH_2-S), 2.01-1.88 (m, 2H, C$\underline{H}_2$-CH(OH)-P), 1.33 (d, $^3J_{HH}$=6.2 Hz, 6H, 2 × CH_3 *i*Pr), 1.33 ppm (d, $^3J_{HH}$=6.2 Hz, 6H, 2 × CH_3 *i*Pr);

^{31}P NMR (161.98 MHz, $CDCl_3$): δ=22.71 ppm (s); ^{13}C NMR (150.93 MHz, $CDCl_3$): δ=138.4 (s, C arom), 129.0 (s, 2 × CH arom), 128.7 (s, 2 × CH arom), 127.2 (s, CH arom), 71.5 (d, $^2J_{CP}$=7.2 Hz, CH *i*Pr), 71.4 (d, $^2J_{CP}$=7.3 Hz, CH *i*Pr), 67.0 (d, $^1J_{CP}$=163.0 Hz, CH-P), 36.2 (s, CH_2 Bn), 30.9 (d, $^3J_{CP}$=1.4 Hz, $\underline{C}H_2$-S), 27.7 (d, $^2J_{CP}$=15.4 Hz, $\underline{C}H_2$-CH-P), 24.3 (d, $^3J_{CP}$=3.5 Hz, CH_3 *i*Pr), 24.3 (d, $^3J_{CP}$=3.6 Hz, CH_3 *i*Pr), 24.2 (d, $^3J_{CP}$=4.7 Hz, CH_3 *i*Pr), 24.2 ppm (d, $^3J_{CP}$=4.8 Hz, CH_3 *i*Pr); IR (ATR): $\tilde{v}$=3276, 2979, 2929, 1726, 1380, 1220, 986, 629 cm^{-1} ; HRMS (ESI): calc. for $[C_{16}H_{27}O_4PS+Na]^+$: *m/z* 369.1260 $[M+Na]^+$; found: *m/z* 369.1241; ^{31}P NMR (161.98 MHz, d_8-toluene) od (*S*)-**40** with chiral solvating agent: δ=95.95 [s, ∫0.73, chiral solvating agent], 23.30 [s, ∫0.27, complex of chiral solvating agent with (*S*)-**40**], 23.48 ppm [s, ∫0.002, complex of chiral solvating agent with (*R*)-**40**]; *ee* ≥ 98.5%.

(*R*)-Diisopropyl [1-azido-3-(benzylthio)propyl]phosphonate [(*R*)-53]: Substitution of the hydroxyl group by an azide was accomplished following **general procedure E**, starting from (*S*)-**40** (260 mg, 0.75 mmol), dissolved in dry CH_2Cl_2, and using DIAD as azoester component. The reaction was stopped after 18 h at 30°C. The residue was purified by MPLC (CH_2Cl_2/Et_2O, 19:1) to yield azide (*R*)-**53** (209 mg, 0.56 mmol, 75%) as colorless oil; R_f=0.81 (CH_2Cl_2:Et_2O, 4:1); α_D^{20}=−73.9 (*c*=1.10 in CH_2Cl_2); ^1H NMR (600.25 MHz, $CDCl_3$): δ=7.34-7.23 (m, 5H, 5 × CH arom), 4.79 (sept, $^3J_{HH}$=6.2 Hz, 1H, CH *i*Pr), 4.77 (sept, $^3J_{HH}$=6.2 Hz, 1H, CH *i*Pr), 3.71 ($^2J_{AB}$=13.7 Hz, 2H, CH_2 Bn), 3.63 (td, $^3J_{HH}$=$^2J_{HP}$=11.4 Hz, $^3J_{HH}$=3.2 Hz, 1H, CH-P), 2.66 (dddd, $^2J_{AB}$=13.4 Hz, $^3J_{AH}$=7.6 Hz, $^3J_{AH}$=4.6 Hz, $^2J_{HP}$=1.3 Hz, 1H, CH_2S), 2.54-2.47, (m, 1H, CH_2-S), 2.05-1.98 (m, 1H, $C\underline{H_2}$-CH-P), 1.88-1.80 (m; 1H, $C\underline{H_2}$-CH-P), 1.36 (d, $^3J_{HH}$=4.3 Hz, 3H, CH_3 *i*Pr), 1.36 (d, $^3J_{HH}$=3.3 Hz, 3H, CH_3 *i*Pr), 1.36 (d, $^3J_{HH}$=4.5 Hz, 3H, CH_3 *i*Pr), 1.35 ppm (d, $^3J_{HH}$=3.1 Hz, 3H, CH_3 *i*Pr); ^{31}P NMR (161.98 MHz, $CDCl_3$,): δ=19.85 ppm (s); ^{13}C NMR (150.93 MHz, $CDCl_3$): δ=138.1 (s, C arom), 129.0 (s, 2 × CH arom), 128.7 (s, 2 × CH arom), 127.3 (s, CH arom), 72.0 (d, $^2J_{CP}$=7.3 Hz, CH *i*Pr), 72.0 (d, $^2J_{CP}$=7.0 Hz, CH *i*Pr), 56.4 (d, $^1J_{CP}$=157.7 Hz, CH-P), 36.1 (s, CH_2 Bn), 28.3 (s, $\underline{C}H_2$-S), 28.1 (d, $^2J_{CP}$=15.1 Hz, $\underline{C}H_2$-CH-P), 24.3 (d, $^3J_{CP}$=3.3 Hz, CH_3 *i*Pr), 24.3 (d, $^3J_{CP}$=3.5 Hz, CH_3 *i*Pr), 24.2 ppm (d, $^3J_{CP}$=4.7 Hz, 2 × CH_3 *i*Pr); IR (ATR): $\tilde{v}$=2979, 2929, 2097, 1740, 1380, 1250, 984, 628 cm^{-1}; HRMS (ESI): calc. for $[C_{16}H_{26}N_3O_3PS+Na]^+$: *m/z* 394.1325 $[M+Na]^+$; found: *m/z* 394.1329; ^{31}P NMR (161.98 MHz, d_8-toluene) (*R*)-**53** with chiral solvating agent: δ=95.60 [∫0.63, chiral solvating agent], 19.95 [∫0.360, complex of chiral solvating agent with (*R*)-**53**], 19.91 ppm [∫0.003, complex of chiral solvating agent with (*S*)-**53**]; *ee* ≥ 98.3%.

18. Phosphaproline and Phosphaornithine (precursor)

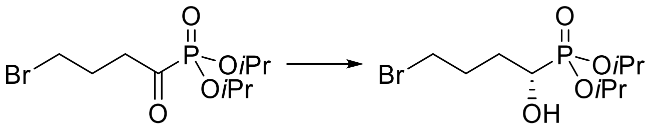

24 (*S*)-**41**

Diisopropyl 1-oxo-4-bromobutylphosphonate (24): Ketophosphonate **24** (93 mg, 0.30 mmol, 68% over two steps) was obtained by **general procedure B** starting from diisopropyltrimethylsilyl phosphite (105 mg, 0.44 mmol).[11] The resulting intermediate hydroxyphosphonate (±)-**41** [R_f=0.41 (EtOAc)] was purified by MPLC using a solvent gradient (*n*-heptane/EtOAc, 24% to 100% EtOAc) before addition of DMP; ^{1}H NMR (600.25 MHz, $CDCl_3$): δ=4.80 (sept, $^3J_{HH}$=6.2 Hz, 1H, CH *i*Pr), 4.79 (sept, $^3J_{HH}$=6.2 Hz, 1H, CH *i*Pr), 3.44 (td, $^3J_{HH}$=6.8 Hz, $^5J_{HP}$=0.8 Hz, 2H, CH_2-Br), 3.03 (td, $^3J_{HH}$=6.8 Hz, $^3J_{HP}$=1.5 Hz, 2H, $C\underline{H}_2$-C(O)), 2.18 (quin, $^3J_{HH}$=6.8 Hz, $C\underline{H}_2$-CH_2-C(O)), 1.38 ppm (broad d, $^3J_{HH}$=6.2 Hz, 12H, 4 × CH_3 *i*Pr); ^{31}P NMR (242.99 MHz, $CDCl_3$): δ=22.18 (s, ∫0.03, residual (±)-**41**), −4.57 ppm (s, ∫0.97, **24**).

(*S*)-Diisopropyl 1-hydroxy-4-bromobutylphosphonate [(*S*)-41]: (*R*)-**41** was obtained from crude keto-phosphonate **24** (approx. 0.81 mmol[1]) following **general procedure D** within 1 h using (*R,R*)-**7** (0.01 equiv., see **general procedure C**) as catalyst. The crude residue was purified by MPLC using a solvent gradient (*n*-heptane/EtOAc, 24 to100% EtOAc) to give the desired product (*S*)-**41** (182 mg, 0.57 mmol, 71%) in an inseparable admixture with diisopropyl (tetrahydrofuran-2-yl)phosphonate[12] (8 mg, 0.03 mmol, 4%) as colorless oil (evaporation of the solvent at 25°C!); R_f =0.31 (EtOAc); ^{1}H NMR (400.27 MHz, $CDCl_3$): δ=4.83-4.69 (m, 2H, 2 × CH *i*Pr), 3.83-3.74 (m, 1H, CH-P), 3.46 (broad t; $^3J_{HH}$=6.8 Hz, 2H, CH_2-Br), 2.47 (symm m, 1H, OH), 2.25-2.10 (m, 1H, $C\underline{H}_2$-CH-P), 2.07-1.86 (m, 2H, 1 × $C\underline{H}_2$-CH-P + 1 × $C\underline{H}_2$-CH_2-Br), 1.86-1.73 (m, 1H, $C\underline{H}_2$-CH_2-Br), 1.36 (d, $^3J_{HH}$=6.3 Hz, 3H, CH_3 *i*Pr), 1.34 (d, $^3J_{HH}$=6.4 Hz, 3H, CH_3 *i*Pr), 1.34 ppm (d, $^3J_{HH}$=6.3 Hz, 6H, 2 × CH_3 *i*Pr); ^{31}P NMR (162.03 MHz, $CDCl_3$): δ=22.58 (s, **41**, ∫0.92), 22.16 ppm (s, diisopropyl (tetrahydrofuran-2-yl)phosphonate, ∫0.08); ^{31}P NMR (162.03 MHz, $CDCl_3$) of (*S*)-**41** with chiral solvating agent: δ=97.09 (s, ∫2.01, chiral solvating agent), 22.46 [s, ∫0.89 complex of chiral solvating agent with (*S*)-**41**], 21.97 ppm [s, ∫0.11, diisopropyl (tetrahydrofuran-2-yl)phosphonate], [complex of chiral solvating agent with (*R*)-**41** not detected]; *ee* ≥ 99%.

References

[1] Drescher, M.; Felsinger, S.; Hammerschmidt, F.; Kählig, H.; Schmidt, S.; Wuggenig, F. *Phosphorus, Sulfur* **1998**, 79-93.

[2] Hammerschmidt, F.; Wuggenig, F. *Tetrahedron: Asymmtery* **1999**, *10*, 1709-1721.

[3] Wuggenig, F.; Schweifer, A.; Meireiter, K.; Hammerschmidt F. *Eur. J. Org. Chem.* **2011**, *10*, 1870-1879.

[4] Hammerschmidt, F.; Kvaternik, H.; Schweifer, A.; Mereiter, K.; Aigner, R. M. *Synthesis* **2012**, *44*, 3387–3391.

[5] Boularot, A.; Giglione, G.; Petit, S.; Duroc, Y.; Alves de Sousa, R:; Larue, V.; Cresteil, T.; Dardel, F.; Artaud, I.; Meinnel, T. *J. Med. Chem.*, **2007**, *50*, 10-20.

[6] Hammerschmidt, F.; Völlenkle, H. *Liebigs Ann. Chem.* **1989**, 577–583.

[7] Hammerschmidt, F.; Lindner, W.; Wuggenig, F.; Zarbl, E. *Tetrahedron: Asymmetry* **2000**, *11(14)*, 2955-2964.

[8] Lejczak, B.; Kafarski, P.; Mastalerz, P. *J. Chromatography* **1985**, *324*, 455–461.

[9] van Staalduinen, L. M.; McSorley, F. R.; Schiessl, K.; Séguin, J.; Wyatt, P. B.; Hammerschmidt, F.; Zechel, D. L.; Jia, Z. *Proc. Natl. Acad. Sci. U. S. A.* **2014**, *111(14)*, 5171-5176.

[10] Guénin, E.; Monteil, M;. Bouchemal, N.; Prangé, T.; Lecouvey, M. *Eur. J. Org. Chem.* **2007**, 3380-3391.

[11] Gama, S R.; Yan Lo, B. S.; Seguin, J.; Pallitsch, K.; Hammerschmidt, F.; Zechel, D. L. *Biochemistry* **2019**, *58*, 5271−5280.

[12] Wuggenig, F., Schweifer, A. Mereiter, K.; Hammerschmidt, F. *Eur. J. Org. Chem.* **2011**, 1870-1879.
